# A novel thermostable TP-84 capsule depolymerase: a method for rapid polyethyleneimine processing of a bacteriophage-expressed proteins

**DOI:** 10.1186/s12934-023-02086-2

**Published:** 2023-04-25

**Authors:** Beata Łubkowska, Edyta Czajkowska, Aleksandra Stodolna, Michał Sroczyński, Agnieszka Zylicz-Stachula, Ireneusz Sobolewski, Piotr M. Skowron

**Affiliations:** 1 Faculty of Health and Life Sciences, Division of Biochemistry, Gdansk University of Physical Education and Sport, Gorskiego 1, 80-336 Gdansk, Poland; 2 Faculty of Chemistry, Department of Molecular Biotechnology, University of Gdansk, Wita Stwosza 63, 80-308 Gdansk, Poland; 3 BioGel Sp. z o.o. (Ltd.), ul. Promienista 83, 60-141 Poznań, Poland

**Keywords:** Capsule, Capsule, Thermophile, Polysaccharide, *Geobacillus stearothermophilus*, Biotechnology, Cloning, Glycosylase, Glycosyl hydrolase, Glicoside hydrolase, Purification, Polyethyleneimine

## Abstract

**Background:**

In spite of the fact that recombinant enzymes are preferably biotechnologically obtained using recombinant clones, the purification of proteins from native microorganisms, including those encoded by bacteriophages, continues. The native bacteriophage protein isolation is often troubled by large volumes of the infected bacterial cell lysates needed to be processed, which is highly undesired in scaled-up industrial processing. A well-known ammonium sulphate fractionation is often a method of choice during purification of the native bacteriophage protein. However, this method is time-consuming and cumbersome, and requires large amounts of the relatively expensive reagent. Thus, other effective and inexpensive methods of reversible protein precipitation are highly desirable. We have previously characterized thermophilic TP-84 bacteriophage, defined a new genus *TP84virus* within *Siphoviridae* family, conducted the TP-84 genome annotation and proteomic analysis. The longest Open Reading Frame (ORF) identified in the genome is TP84_26. We have previously annotated this ORF as a hydrolytic enzyme depolymerizing the thick polysaccharides host’s capsule.

**Results:**

The TP84_26 ‘capsule depolymerase’ (depolymerase) is a large, 112 kDa protein, biosynthesized by the infected *Geobacillus stearothermophilus* 10 (*G. stearothermophilus* 10) cells. The TP84_26 protein biosynthesis was confirmed by three approaches: (i) purification of the protein of the expected size; (ii) mass spectrometry (LC–MS) analysis and (iii) detection of the enzymatic activity toward *G. stearothermophilus* polysaccharide capsules. Streptomycin-resistant mutant of the host was generated and microbiological aspects of both the TP-84 and *G. stearothermophilus* 10 were determined. A new variant of polyethyleneimine (PEI)-mediated purification method was developed, using the novel TP-84 depolymerase as a model. The enzyme was characterized. Three depolymerase forms were detected: soluble, unbound proteins in the bacteriophage/cells lysate and another integrated into the TP-84 virion.

**Conclusions:**

The novel TP-84 depolymerase was purified and characterized. The enzyme exists in three forms. The soluble, unbound forms are probably responsible for the weakening of the capsules of the uninfected bacterial cells. The form integrated into virion particles may generate a local passage for the invading TP-84. The developed PEI purification method appears well suited for the scaled-up or industrial production of bacteriophage proteins.

**Supplementary Information:**

The online version contains supplementary material available at 10.1186/s12934-023-02086-2.

## Introduction

Biotechnological production of enzymes requires optimization of the maximum possible number of parameters, exemplified by: microbial cultivation conditions, protein biosynthesis, expression in the case of recombinant proteins, purification, enzymatic assays, and scaling-up. We have recently published papers concerning improving recombinant toxic proteins expression and purification methodology, including: (i) in vivo transient locking of maturation/quenching toxicity of recombinant bacterial alkaline phosphatase, followed by reactivation in vitro [1], (ii) evaluating the crucial effect of codon composition on translation kinetics leading to activation of expressed thermostable protein [2] and (iii) development of an AT-rich codon-biased gene optimization method [3]. Here we have developed a specialized method of removal of nucleic acids with PEI and protein purification in a novel format, dedicated to obtaining proteins from highly diluted samples, such as those bacteriophage-coded obtained from non-recombinant, native microorganism upon infection. Such protein isolations are often troubled by large volumes of infected cell lysates needed to be processed, which is undesired with scaled-up scientific or industrial processing. Thus efficient and reasonable reversible protein precipitation methods can solve those issues. Well-known ammonium sulfate fractionation uses large amounts of the relatively expensive reagent. This technique is also time-consuming and unwieldy. Since we have been studying the molecular biology of the TP-84 bacteriophage, infecting *G. stearothermophilus* [4] and we intend to establish it as a model thermophilic bacteriophage, we have selected a novel depolymerase, encoded by TP-84, as a model protein for this method. We have previously sequenced the TP-84 genome, established 81 coding DNA sequences (CDSs), confirmed biosynthesis of 31 proteins and defined a new genus *TP84virus* [4]. The TP-84 bacteriophage employs at least three lytic proteins that facilitate bacterial infection. These enzymes are responsible for degradation of the bacterial capsule, cell wall and cytoplasmic membrane. The genes encoding three TP-84 lytic proteins are grouped in a cluster in the middle of the TP-84 genome [4]. The cluster includes: (i) TP84_26 gene (encoding depolymerase), (ii) TP84_27 gene (encoding holin) and (iii) TP84_28 gene (encoding endolysin). The TP84_26 gene codes for a novel, 112.2 kDa depolymerase, which is biosynthesized by the TP-84-infected *G. stearothermophilus* cells. The TP84_26 ORF was annotated as ‘glycosylase’, using HHpred depolymeraseserver for protein homology detection [4, 5]. Initially, the HHpred sequence similarity to putative sporulation-specific glycosylase YdhD from *Bacillus subtilis* was detected [4]. Moreover, several homologs of the TP84_26 encoded protein were identified among *Bacillus* and *Geobacillus* glycosyl/glycoside hydrolases family 18, and glycoside hydrolases from the *Geobacillus* phage GBK2 (GenBank YP_009010491) [4, 6]. A few hypotheses can be made regarding the function of TP84_26 protein, including: (i) the enzyme is needed for glycosylation of a TP-84 protein or DNA; (ii) it is a tail fiber-associated protein exhibiting enzymatic activity, allowing TP-84 penetration of thick *G. stearothermophilus* capsule and (iii) like TP84_28 (endolysin) it is involved in lysis or both (ii) and (iii). The rather remote possibility (i) can be excluded with regard to DNA, as no modified bases by a TP-84-coded enzyme in TP-84 genome were detected. Only a minor GATC adenine methylation was found, apparently caused by the hosts’ Dam system (to be published elsewhere). Research into various depolymerases is of increasing interest due to their potential biomedical applications. For example, depolymerases can be potentially used for degradation of capsule-mediated microbial biofilms, including those caused by pathogenic bacteria in humans, such as *Klebsiella pneumoniae* or *Acinetobacter baumannii* [7, 8]. The recent renaissance of interest in bacteriophages and their enzymes, including depolymerases, showed the substantial potential of depolymerases as antibacterial agents. Such an approach can lead to the development of targeted bacteriophage therapies. Nonetheless, bacteriophage-encoded depolymerases target only a limited number of bacterial strains and have relatively narrow specificity when compared to broad-spectrum antibiotics. Therefore, searching for novel depolymerases is a promising approach. In this work, we have identified, isolated, and characterized native depolymerase, encoded by the TP84_26 gene. Further, we have developed a new method of PEI co-precipitation/purification. The technique is particularly suitable for protein purification from diluted post-infection cell lysates, with general use in biotechnological research.

## Materials and methods

### Bacterial strains, media and reagents

*G. stearothermophilus* strain 10 and TP-84 bacteriophage were originally obtained from Epstein and Campbell [9]. *G. stearothermophilus* NUB3621 (BGSCID 9A5) was from Bacillus Genetic Stock Center (BGSC) (USA) [10]. For preparative scale bacteria were grown with vigorous aeration at 55 °C, using TYM rich medium [11] containing calcium, magnesium ions and fructose, known to stimulate TP-84 yield (Pepton K, 20 g/L (BTL, Poland), yeast extract at 4 g/L (BTL), upon sterilization/cooling CaCl_2_, MgCl_2_, fructose and streptomycin were added to 5 mM, 10 mM, 0.5% and 50 μg/ml, respectively. For solid media, bottom TYM agar contained 2% agar and top TYM agar contained 0.6%. Streptomycin-resistant mutant of *G. stearothermophilus* 10 str^R^ was constructed by serial passaging on solid TYM media with increasing concentrations of streptomycin: 5 μg/ml, 10 μg/ml, 20 μg/ml, 50 μg/ml using 300 μl of dense, overnight (17 h) culture (app. 10^8^ cells) of *G. stearothermophilus* 10. Protein standards: PageRuler™ Plus Prestained Protein Ladder (cat. nr 26619), LMW SDS Marker Kit (cat. nr 17044601) and Coomassie™ Brilliant Blue (cat. nr 20279) were from GE Healthcare (USA). DNA markers were from Thermo Fisher Scientific (USA) and GE Healthcare Bio-Sciences AB (USA). Q- Sepharose™ Fast Flow (cat. 17–0510-01), Blue-agarose™ Fast Flow (cat. 17–0948-01), Heparin-Sepharose™ 6 Fast Flow (cat. 17–0998-01) chromatographic resins were from GE Healthcare Bio-Sciences AB. SIGMAFAST™ Vivaspin Turbo 15 PES 10000 MWCO PES 48 pc (cat. no VS15T02), VivaSpin^®^ Turbo 15 RC (100 kDa cutt-off, cat. No VS15T41) devices were from Sartorius Stedim Biotech GmbH (Germany). Protease Inhibitor Cocktail Tablets, EDTA-Free (cat. no S8820), Sephacryl S300 HR and all the other chemicals were from Sigma-Aldrich (USA).

### TP-84 bacteriophage propagation and purification

Bacteriophage TP-84 was propagated by infection of bacterial host strain *G. stearothermophilus* 10 str^R^ or *G. stearothermophilus* NUB3621 [10] at various cell densities and M.O.I., depending on the purpose of cultivation. Mostly the conditions included OD_600nm_ range 0.3–0.4 and M.O.I = 1–10. 30 min (min) prior infection, bacterial culture was supplemented again with 0.5% fructose and terminated when OD_600nm_ dropped to 0.1 or less by addition of 3 ml of chloroform per 1 L and shaking for 20 min at 55 °C. Bacterial debris was removed by centrifugation and bacteriophage was precipitated with PEG 8000 and NaCl at final concentrations of 10% (w/v) and 0.5 M, respectively and stirred overnight. TP-84 particles precipitate was collected by centrifugation at 6000 x g for 20 min at 4 °C and resuspended in 3 ml of TMC buffer pH 8.0 (50 mM Tris–HCl pH 8.0 at 20 °C; 10 mM MgCl_2_, 5 mM CaCl_2_), the volume was adjusted to 5 ml and layered onto CsCl step gradients (1.7 g/ml, 1.5 g/ml and 1.45 g/ml) in clear polypropylene centrifuge tubes (PA Thinwall tube, PKG 50, Thermo Fisher Scientific). After centrifugation at 80000 x g (Thermo Fisher Scientific™ Sorvall™ WX80 + Floor Ultracentrifuges, Rotor Sur Spin 630/17 Thermo Scientific (17 mL), Germany) for 2 h at 10 °C, the bacteriophage was dialyzed twice at 4 °C overnight against TMC buffer pH 7.5 and titrated at 55 °C, using 200 µl of a fresh culture with OD_600nm_ = 0.3 [9, 12].

### Effect of temperature on the bacteriophage depolymerase production

To investigate the effect of temperature on TP-84 production, 50 ml of liquid medium of *G. stearothermophilus* 10 str^R^ (OD_600nm_ = 0.22) were infected at M.O.I of 1. TP-84-infected cultures were carried out in liquid TYM medium and on plates with solid TYM medium. A wide temperature range was tested, from mesophilic (31 °C) to extremophilic (82 °C). The temperature was evaluated in 3 °C increasements (31, 34, 37, 40, 43, 46, 49, 52, 55, 58, 61, 64, 67, 70, 73, 76, 79, 82) and the bacteriophage titers measured [11]. Plates for titers counting were incubated at optimal temperature of 55 °C for 24 h.

### Novel variant of PEI fractionation method: depolymerase purification Protocol 1

The native TP-84 depolymerase was purified, using a novel protocol developed in this work, which included: (*i*) co-precipitation of nucleic acids and depolymerase with limiting concentration of PEI directly from TP-84 / *G. stearothermophilus* 10 str^R^ lysates, followed by selective back-extraction; (*ii*) Phosphocellulose P11 chromatography; (*iii*) Q-Sepharose chromatography; (*iv*) Heparin-agarose chromatography; (*v*) Size fractionation and concentration by centrifugation in the Vivaspin membrane-spin devices with 100 kDa cut-off, removing smaller than depolymerase contaminants.

### ***TP-84 / G. stearothermophilus 10 str***^***R***^*** cells lysate preparation***

For the purpose of depolymerase production, *G. stearothermophilus* 10 str^R^ was grown at 55 °C for overnight and 5 ml used to inoculate 1 L TYM medium and cultivated at 55 °C with vigorous aeration for app. 4 h to OD_600nm_ of app. 0.4. Then fructose was again added to 0.5% and the culture was continued for 30 min. The host strain is somewhat picky and older inoculums, stored at 4 °C, do not work well. The cells in liquid culture were infected with 6.2 × 10^10^ TP-84 particles (M.O.I. app. 10) for 10 min without shaking, then cultivation was continued at 55 °C for 7.5 h. When OD_600nm_ dropped to 0.1, PMSF stock solution in isopropanol (2.5 ml, 0.2 M) was added to 0.5 mM to inhibit TP-84 and hosts proteases as well as 3 ml of chloroform and left for overnight at room temperature to complete cell lysis. Cell debris was spun down and the supernatant was supplemented with 20 mM EDTA and 80 mM NaCl to remove weakly bound proteins from PEI complexes.

#### PEI fractionation scheme 1

5 ml samples of the cleared lysate were subjected to titration with 1% (v/v) PEI solution (pH 7.5), using (μl): 5, 10, 20, 35, 50, 70, 100, 150, 200, 300 with rapid mixing, to select the amount needed to generate the most intense turbidity, while avoiding excess of the reagent. The samples were shaken for 5 s and incubated for 1 h at 4 °C, and OD_600nm_ was determined. For preparative depolymerase purification, 40 ml of 1% PEI was added with rapid mixing to 1 L of cleared lysate, stirred for 1 h at 4 °C and centrifuged for 20 min at 6,000 × g to remove PEI complexes with nucleic acids and acidic proteins. The supernatant was diluted 1: 1 with distilled water and spun down again to remove remaining portion of the complexes. Both centrifuged complexes were combined, resuspend in 20 ml DEAE buffer (30 mM Tris–HCl, pH 8.0 at 20 °C, 0.5 mM EDTA, 20 mM NaCl, 0.01% Triton X-100, 0.01% Tween 20, 5% glycerol, 5 mM β-mercaptoethanol (βMe), 0.1 mM PMSF) and incubated for overnight at 4 °C. After incubation, 6.75 ml of 0.5 M NaCl in DEAE buffer was added (final concentration 220 mM) to the resuspension (27 ml), mixed, and the back-extracted complexes were spun down (20 min, 6000 × g, 4 °C). The supernatant (30 ml) was dialyzed against DEAE buffer. Aa an alternative back extraction buffer variant low pH phosphate-based was used [50 mM K/PO_4_, pH 6.0, 0.5 M NaCl].

#### Phosphocellulose P11 chromatography

The dialyzed supernatant was loaded onto Phosphocellulose P11 column (1.8 ml), equilibrated in DEAE buffer (pH 8.0). This column served as negative step, with depolymerase eluting in flow-through. The preparation was dialyzed against DEAE buffer, supplemented with 100 mM NaCl.

#### Q-Sepharose chromatography

The dialyzed depolymerase solution was loaded onto Q-Sepharose column (1.8 ml), equilibrated in DEAE buffer, supplemented with 100 mM NaCl. The column was washed with 10 ml DEAE buffer and eluted with 2 steps of increased NaCl concentration (250 and 500 mM) in DEAE buffer. Fraction were analyzed using 10% SDS-PAGE and those containing maximum amounts of depolymerase (4 ml) were pooled and diluted with 20 ml of DEAE buffer.

#### Heparin-agarose chromatography

Diluted fractions were applied onto Heparin-agarose column (2.5 ml), equilibrated in DEAE buffer. This column served as negative step, with depolymerase eluting in flow-through fractions.

#### Membrane size fractionation and concentration

The DP preparation (23 ml) was concentrated and simultaneously used to remove small proteins by centrifugation in VivaSpin^®^ Turbo 15 RC (100 kDa cut-off) device down to 6 ml, diluted with DEAE buffer and concentrated again, obtaining finally app. 1 ml solution, which was dialyzed against storage buffer S (50 mM Tris–HCl pH 8.0 (at 23 °C), 100 mM NaCl, 2.5 mM MgCl_2_, 2.5 mM CaCl_2_, 0.01% Triton X-100, 0, 50% glycerol). To the resulting 0.6 ml final preparation of depolymerase, reducing agent TCEP was added to 1 mM.

### Alternative partial purification of depolymerase (Protocol 2)

The protocol included direct purification of depolymerase along with soluble bacteriophage-encoded proteins from TP-84/*G. stearothermophilus* lysates using (*i*) selective polyethylene glycol 8000 (PEG)/NaCl precipitation and removal of TP-84 particles and (*ii*) Q-Sepharose chromatography. Several variants of this protocol were developed, example Protocol 2 is shown here.

#### PEG/NaCl precipitation

2 L of cleared TP-84 lysate was prepared as in the Protocol 1. NaCl was added to 0.5 M concentration, upon dissolving, PEG was gradually added to final 10% with slow mixing and the precipitation was allowed for overnight at 4 °C. The precipitate was centrifuged (4 °C, 60 min at 7000 × g) and the supernatant was dialyzed against 2 changes of 10 L of DEAE buffer (pH 8.0).

#### Q-Sepharose chromatography

depolymerase solution from the previous step was loaded onto Q-Sepharose (44 ml), equilibrated in DEAE buffer pH 8.0, the column was washed with 100 ml of DEAE buffer (without glycerol) and depolymerase was eluted with steps of NaCl concentrations in DEAE buffer (pH 7.5): 50, 100, 150, 200, 250, 300, 500, 800 mM (50 ml each). The fractions containing depolymerase were pooled and dialyzed against the storage buffer S.

#### LC–MS, electrophoresis, western blotting, quaternary composition analysis

LC–MS analysis of TP-84 bacteriophage particles, infected *G. stearothermophilus* 10 cells lysates and chromatography columns fractions were conducted in various configurations: (*i*) PEG/CsCl purified intact TP-84 was dialyzed against TMC buffer pH 7.5 (50 mM Tris–HCl pH 7.5 at 22 °C, 10 mM MgCl_2_, 5 mM CaCl_2_) and subjected to SDS/PAGE in Tris–glycine buffer using various resolving gel concentrations: 6%, 8%, 10%, 15%, 20% for analysis of well separated bands in various MW ranges. Coomassie Brilliant Blue-stained bands were excised and subjected to in-gel digestion with trypsin, followed by LC–MS analysis, as we described previously [4]; (ii) approach as in (i), except that Tricine buffer was used for resolution of the smallest TP-84 proteins; (iii) partially purified or completely purified TP-84 proteins. The amino acid (aa) sequences as Mascot Reports obtained from LC–MS analysis were further analyzed using software SnapGene 6.1 (GSL Biotech LLC, USA). Native electrophoresis of purified depolymerase was conducted in 6% polyacrylamide gels, casted in TMC pH 7.5 buffer in MiniProtean® Tetra Vertical Electrophoresis Cell (BioRad, USA), maintaining moderate in-gel temperatures. Samples (20 μl) were prepared by addition of 2.85 μl of native loading buffer, 8X concentrated (62.5 mM Tris–HCl pH 6.8, 40% glycerol, 0.01% bromophenol blue). The yield of the depolymerase protein was determined by densitometric analysis of bands on SDS-PAGE gels using UN-SCAN-IT gel 7.1 software (Silk Scientific Inc., USA). Samples for the TP-84 virion protein electrophoresis were prepared using the bacteriophage preparation purified by CsCl density gradient, and dialyzed against buffer DEAE pH 8.0. SDS-PAGE samples from diluted solutions, such as TP-84 lysates, were prepared by precipitation with trichloroacetic acid (TCA) in the presence of sodium deoxycholate [13]. Western blotting was conducted in Trans-Blot® Turbo™ Transfer System (Bio-Rad, USA) Onto PVDF GE 0.2 µm membrane Amersham™ Hybond® P Western blotting PVDF GE10600021 (Merck, Germany), soaked in methanol and transferred to Towbin buffer supplemented with SDS and methanol (25 mM Tris-192 mM glycine pH 8.3, 20% methanol, 0.04% SDS). The membrane was blocked with EveryBlot Blocking Buffer (BioRad, cat. no 12010020). The primary rabbit anti-TP-84 antibodies were developed at Davids Biotechnologie (Germany) using 28-day immunization protocol, and whole IgG fraction was affinity purified on Protein A chromatography. The secondary HRP-conjugated anti-rabbit antibodies (cat. no 31460) were obtained from Thermo Fisher Scientific. The working solutions of antibodies were prepared in TBS-T buffer (50 mM Tris–HCl pH 7.5, 150 mM NaCl, 0.05% Tween 20), at a concentration of 20 µg/ml (primary antibodies) and at dilution of 1: 1000 (secondary antibodies).

Molecular sieving was conducted on Sephacryl S300 HR using 2.5 × 120 cm column, equilibrated in DEAE buffer pH 8.0, at a flow rate of 24 ml/h.

### Alcian blue enzymatic assay

A substrate for the depolymerase was prepared from whole *G. stearothermophilus* 10 str^R^ cells by conducting early log phase culture in TYM medium, supplemented with streptomycin, till OD_600nm_ = 0.3. The cells were centrifuged, resuspended in equal volume of TMC buffer, centrifuged again and resuspended in 1/10 of the culture volume. Digestions were conducted by addition of 1 µg of depolymerase to app. 3 × 10^6^ cells (16 µl of the prepared substrate) at 55 °C with gentle shaking. Upon reaction completion, samples were SDS/heat denatured, run on SDS-PAGE and gels were stained with Alcian Blue procedure [14].

## Results

### Microbiology aspects of G. stearothermophilus 10 str.^R^ infection by TP-84

**Fig. 1:**
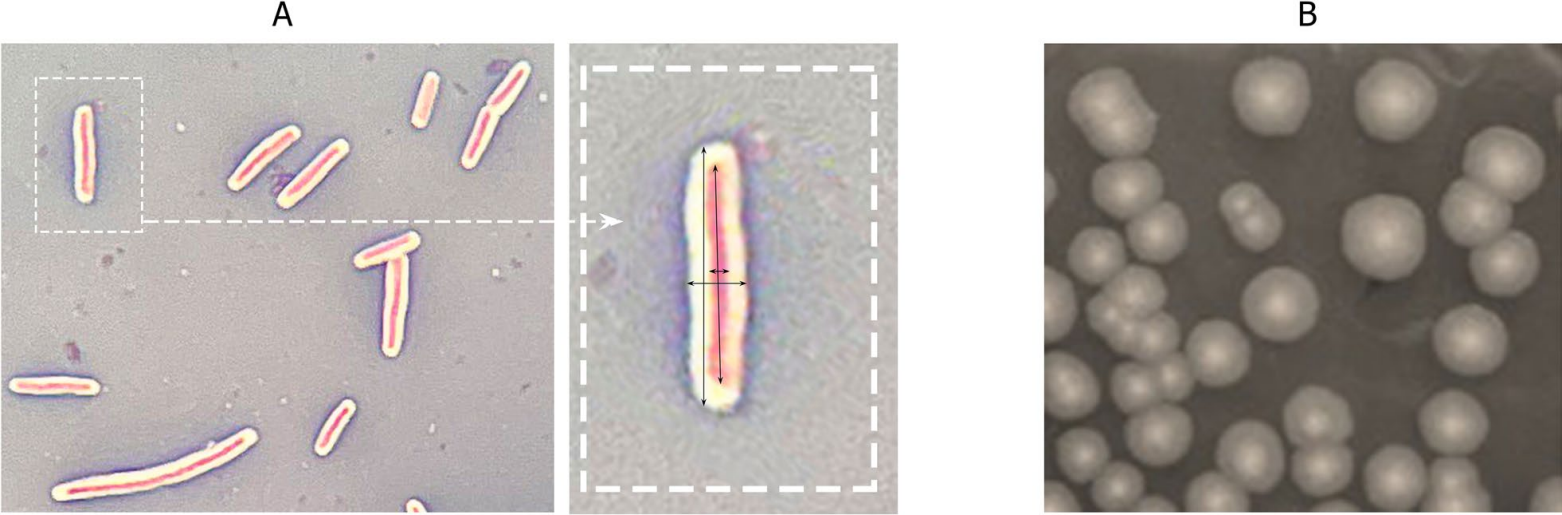
*G. stearothermophilus* 10 str^R^ cell and colonies morphology. Panel **a**. Maneval test, magnification 1000X, 18 h cultivation; →—enlarged window, showing *G. stearothermophilus* 10 str^R^ size measurements. Panel **b**. *C*olonies on solid TYM medium after 24 h incubation at 55 °C

Aminoglycoside antibiotic—streptomycin [15, 16] as relatively temperature resistant, was selected for the use with *G. stearothermophilus 10*. Indirected progressive selection pressure was used to select several spontaneous resistant colonies from app. 10^8^ cells reaching high streptomycin resistance to the concentration of 50 µg/ml. This *G. stearothermophilus* 10 str^R^ mutant was used for further studies. Morphology of the cells, colonies (Fig. 1), media requirements and sensitivity to the TP-84 were not changed. The bacteria synthesize and secrete large amounts of extracellular substances that form a compact layer of polymers (capsules) surrounding—*G. stearothermophilus* 10 str^R^ cells [17]. The bacteria produce thick capsules both in liquid cultures and on agar plates which is favored by high sugar content in the TYM medium. The capsules were stained with Maneval Test [18] (Fig. 1) showing that *G. stearothermophilus* 10 str^R^ capsule typically contain one or two cells. Measurements of the bacterial cells and capsules relative dimensions on indicated that the volume of the capsule is vastly exceeding the cell volume on average by app. 20 times (Fig. 1). Further evaluation revealed the presence of highly resistant endospores [19–21] (Fig. 1). Under the culture conditions used, the Gram( +) *G. stearothermophilus* 10 str^R^ produces spores from early log phase, in spite of the presence of sugars, which are known inhibitors of spores formation. High spores production was observed in the late logarithmic phase (Additional file 1, 2), thus above OD_600nm_ of 0.7 TP-84 lower yields or even no proliferation was observed (not shown). Using standardized conditions [22, 23], *G. stearothermophilus* 10 str^R^ was grown on solid TYM medium with 2% agar and analyzed after 24-h incubation at 55 °C. The colonies morphology features included: round shape, slightly raised, with a wavy edge, gray, opalescent color with light beige centers and smooth, shiny surface. The color environment of the colony did not change (Fig. 1).

**Fig. 2:**
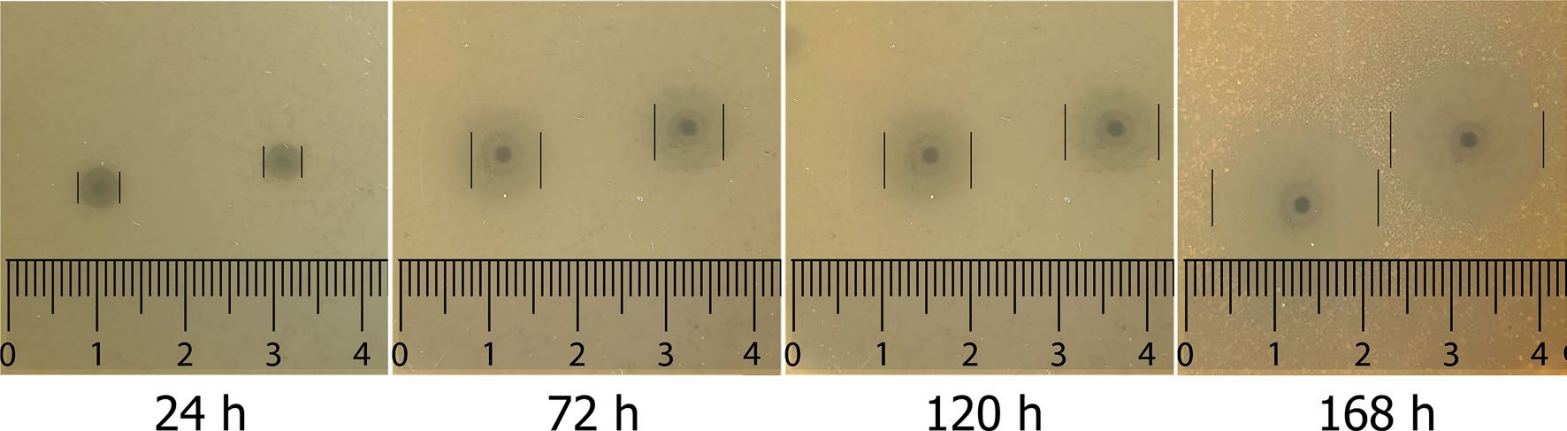
Time course of the increase in the size of hazy rings (‘haloes’) surrounding the TP-84 plaques, obtained on *G. stearothermophilus* 10 str^R^ bacterial lawn

The *G. stearothermophilus* bacterial cells were reported as rods of app. 2–3.5 μm in length and 0.6–1 μm in width [24], which is consistent with our observations (not shown). The growth and infection kinetics of bacteriophage TP-84/*G. stearothermophilus* 10 str^R^ expressed as OD_600nm_, CFU and PFU / ml, shows essentially complete lysis, yielding clear lysates with small amount of bacterial debris, characteristic for a robust, lytic bacteriophage (Additional file 3, 4) [25]. In the culture/infection conditions tested, an alternative TP-84 host, *G. stearothermophilus* NUB3621 [10], reported as thermophilic cloning strain of *G. stearothermophilus* [26], yielded lysates titers of app. 8 × 10^5^ PFU /ml only. We did not observed the ‘halo’ generated around the bacteriophage plaques, when NUB3621 strain was used as a host (not shown). In contrast, TP-84 plagues generated on the *G. stearothermophilus* 10 str^R^ lawn are surrounded by the ‘halo’, which is large and increases in size with incubation time (Fig. 2).

### TP-84 depolymerase biosynthesis in vivo and localization

**Fig. 3:**
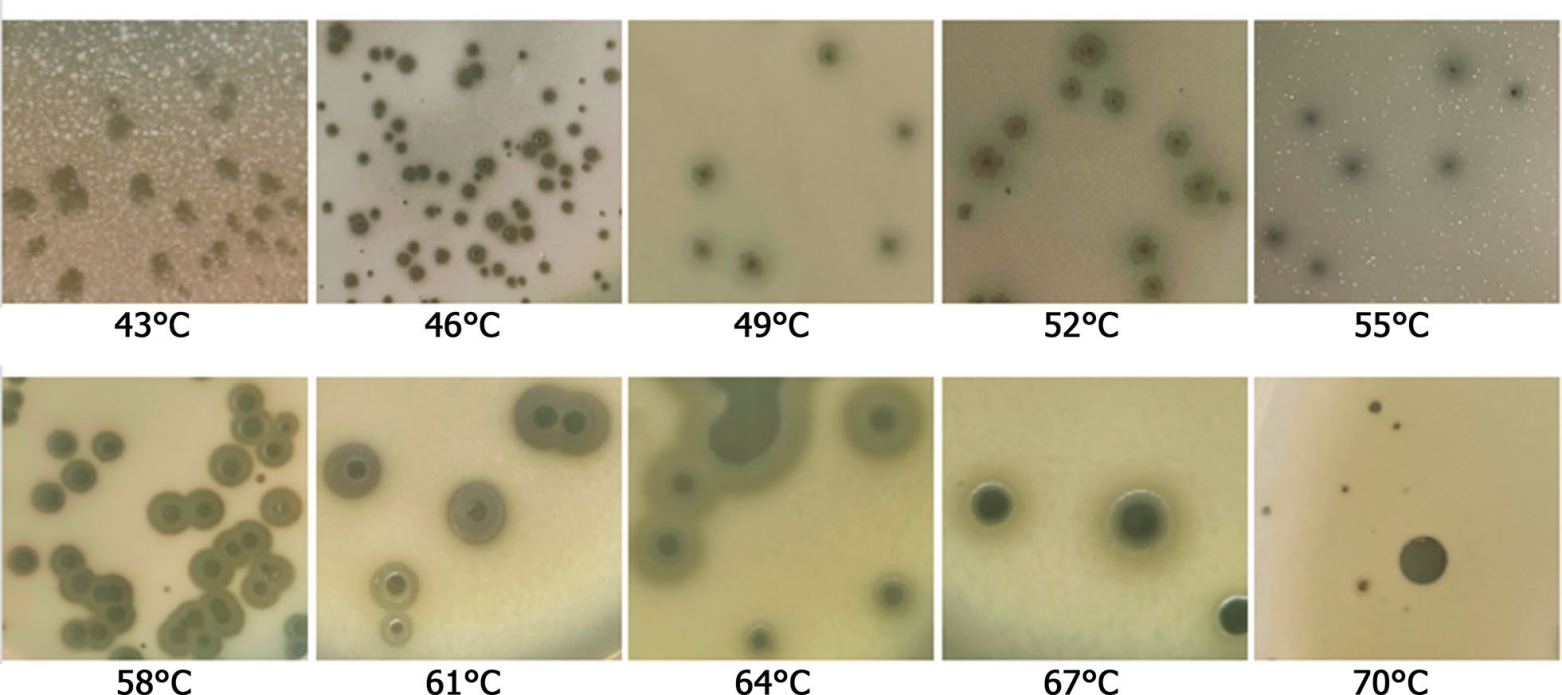
Morphology of TP-84 plaques obtained on *G. stearothermophilus* 10 str^R^ at various temperatures after 24 h incubation

Besides obligatory Ca^2+^ and Mg^2+^ requirements for high production of TP-84, as for other bacteriophages [25, 27, 28], high TP-84 yield is also sugar-dependent. On the TYM medium, supplemented with glucose and/or fructose, TP-84 infection is stimulated hundredfolds, as well the increased host’s capsules [11, this work]. Further we evaluated depolymerase activity in vivo in relation to the host’s temperature growth range, TP-84 life cycle and the presence or absence of the ‘halo’: (i) on solid media in the temperature range of 43–70 °C. It was not possible to reliably observe plaques outside this range due to too high/low humidity in plates, very slow growth, agar deformation, drying and (ii) in liquid cultures at 31–43 °C and 70 °C to 79 °C. The ‘halo’ was observed in the range of 46 °C to 67 °C. The 70 °C temperature was the cut-off point, at which depolymerase activity ceased, only plaques without ‘halo’ were observed (Fig. 3). In the temperature range of 49–58 °C, the bacteriophage plaques are medium to large, typical in appearance, single, well separated. From 61 °C the plaques tend to merge to form big irregular shapes, apparently due to high humidity caused by water evaporation from agar surface. Furthermore, other differences in plaque morphology were observed: they were becoming significantly larger and multilayered.

**Fig. 4:**
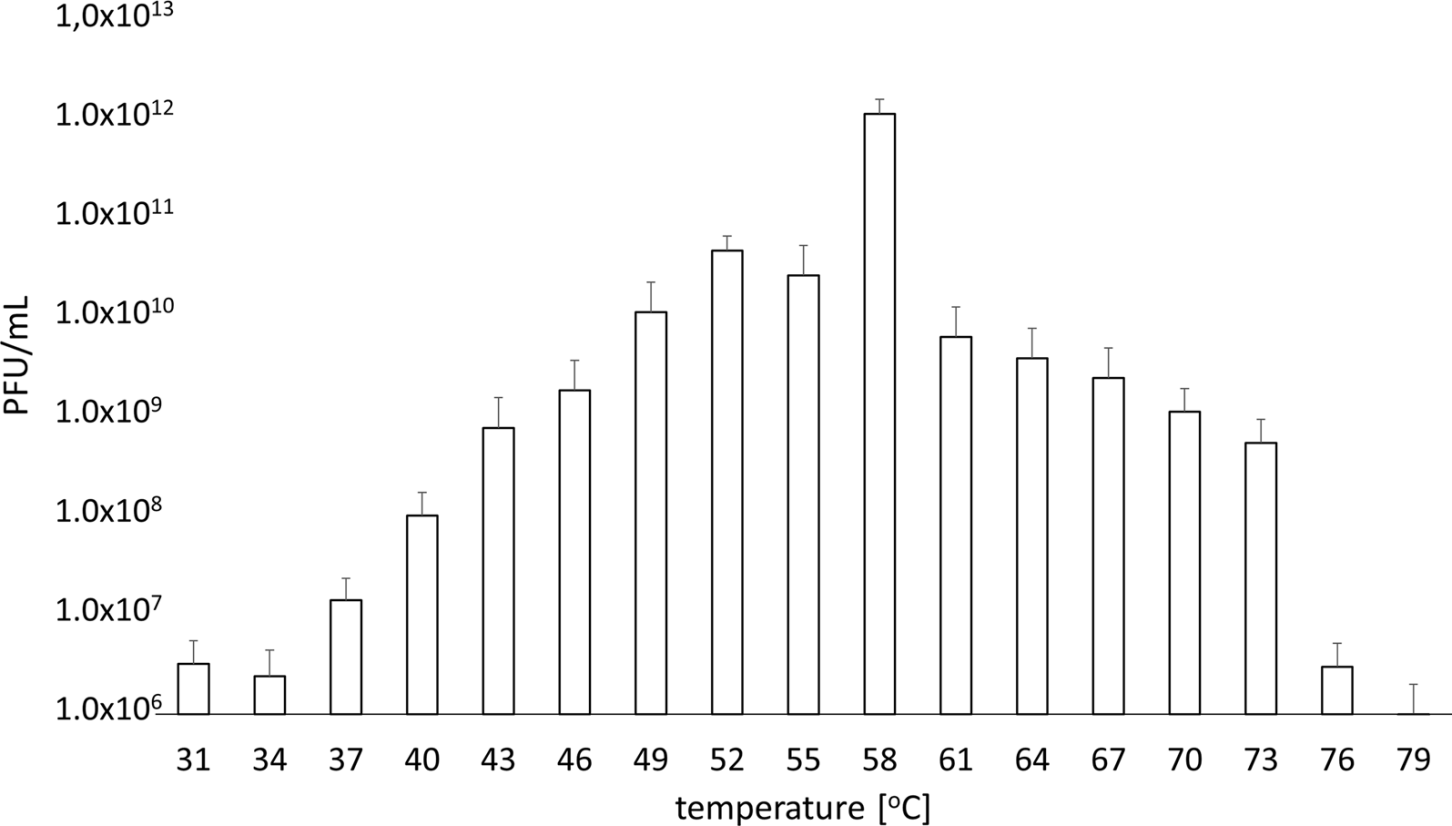
Dependence of bacteriophage TP-84 proliferation yields (PFU/ml) on cultivation temperature

**Fig. 5:**
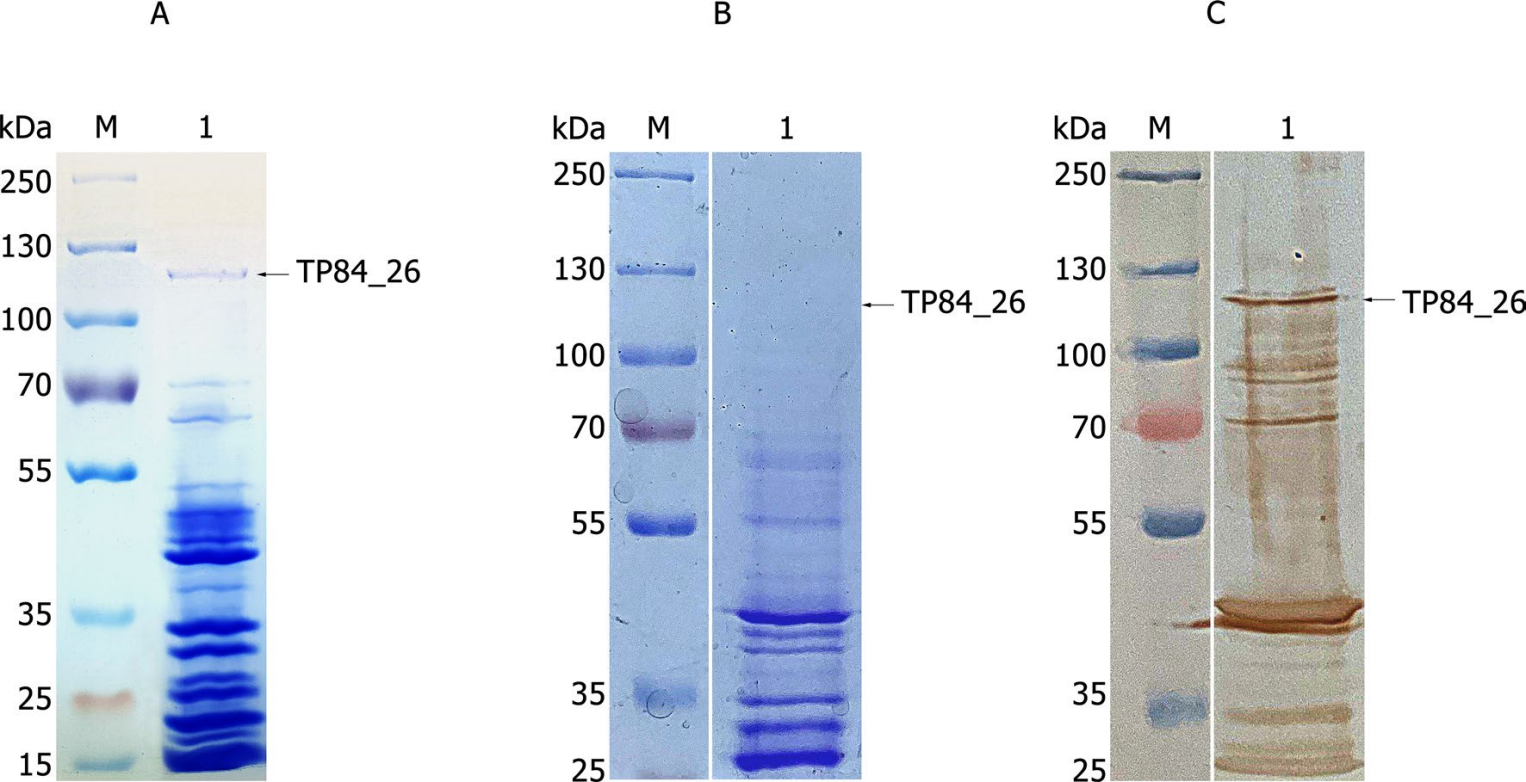
Determination of the depolymerase presence in TP-84 virions. Panel **a** SDS-PAGE of structural TP-84 proteins in 10% gel (concentrated virions purified by CsCl density gradient; after single ultracentrifugation). Lane M, PageRuler™ Plus Prestained Protein Ladder, 10 to 250 kDa; Lane 1, TP-84 bacteriophage, app. 10^11^ virions. Panel **b** SDS-PAGE of structural TP-84 proteins in 8% gel (CsCl density gradient; after double ultracentrifugation). Lane M, PageRuler™ Plus Prestained Protein Ladder, 10 to 250 kDa; Lane 1, TP-84 bacteriophage, app. 4 × 10^10^ virions. Panel **c** As in panel **c** after Western blotting and immunodetection with anti-TP-84 antibodies

Figure 4 shows TP-84 growth and yields (PFU/ml) at various temperatures, resulting in the highest yield at 58 °C. This is correlated with most distinct ‘halo’ on the agar plates. In addition to the range of detectable depolymerase ‘halo’ activity at 46–67 °C. The TP-84/*G. stearothermophilus* 10 system developed at different speed at various temperatures outside the optimum. In the range between 31 °C and 43 °C, a longer incubation, up to 3–4 days, was needed to see TP-84 plaques, while at high temperatures, plaques could be counted after less than 20 h (Fig. 4, Additional file 5). The capsule barrier penetration problem optimally would be solved by a dual function depolymerase: (i) soluble form present in the medium, weakening uninfected bacteria capsules prior to TP-84 invasion (seen as ‘halo’) and (ii) local digestion by depolymerase form associated as structural protein in the region of tail fibers and baseplates, acting precisely at the point of the TP-84-capsule contact. To examine the possibility (ii) we have raised antibodies against purified TP-84, which were used in Western blotting detection of depolymerase in CsCl-purified TP-84 particles, subjected to SDS-PAGE. As shown in Fig. 5a, a faint but clearly visible protein band is present. MW of this protein corresponds to 112 kDa, theoretically predicted for the depolymerase. Figure 5b, c show the results of SDS-PAGE and immunoblotting of the sample containing lower concentration of virions, obtained after double-ultracentrifugation. As can be seen in all panels of the Fig. 5, numerous bands (corresponding to TP84 virion proteins) appeared. Nevertheless, the depolymerase band was detected in all three analysis.

### Novel variant of PEI fractionation: depolymerase purification

#### PEI-mediated depolymerase purification

**Fig. 6:**
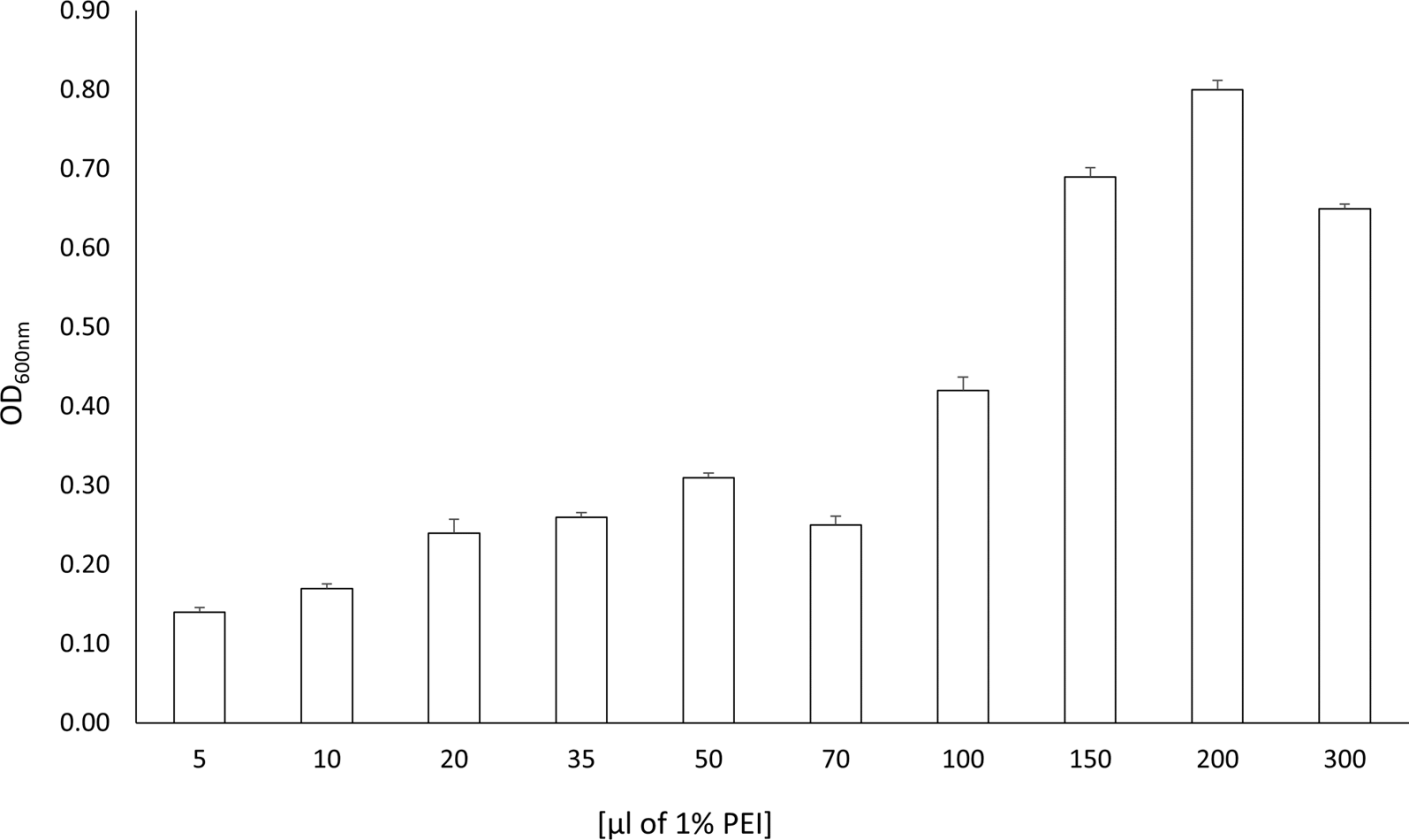
TP-84/*G. stearothermophilus* 10 str^R^ lysates titration with PEI. The graph shows amounts of PEI added as 1% solution to 5 ml lysate samples. For viewing clarity, the scale is not proportional

FAs a stock solution a 1% buffered PEI was used to allow precise addition of small amounts of the reagent. TP-84 / *G. stearothermophilus* 10 str^R^ lysate was titrated with PEI and the concentration yielding the highest turbidity was selected for scaled up purification (Fig. 6, Additional file 6). This purification stage turned out to be very efficient, as naturally expressed in minute amounts TP-84 depolymerase was quantitatively co-precipitated and recovered by back-extraction with high salt buffer. The obtained preparation contained app. 50% of depolymerase protein.

**Fig. 7:**
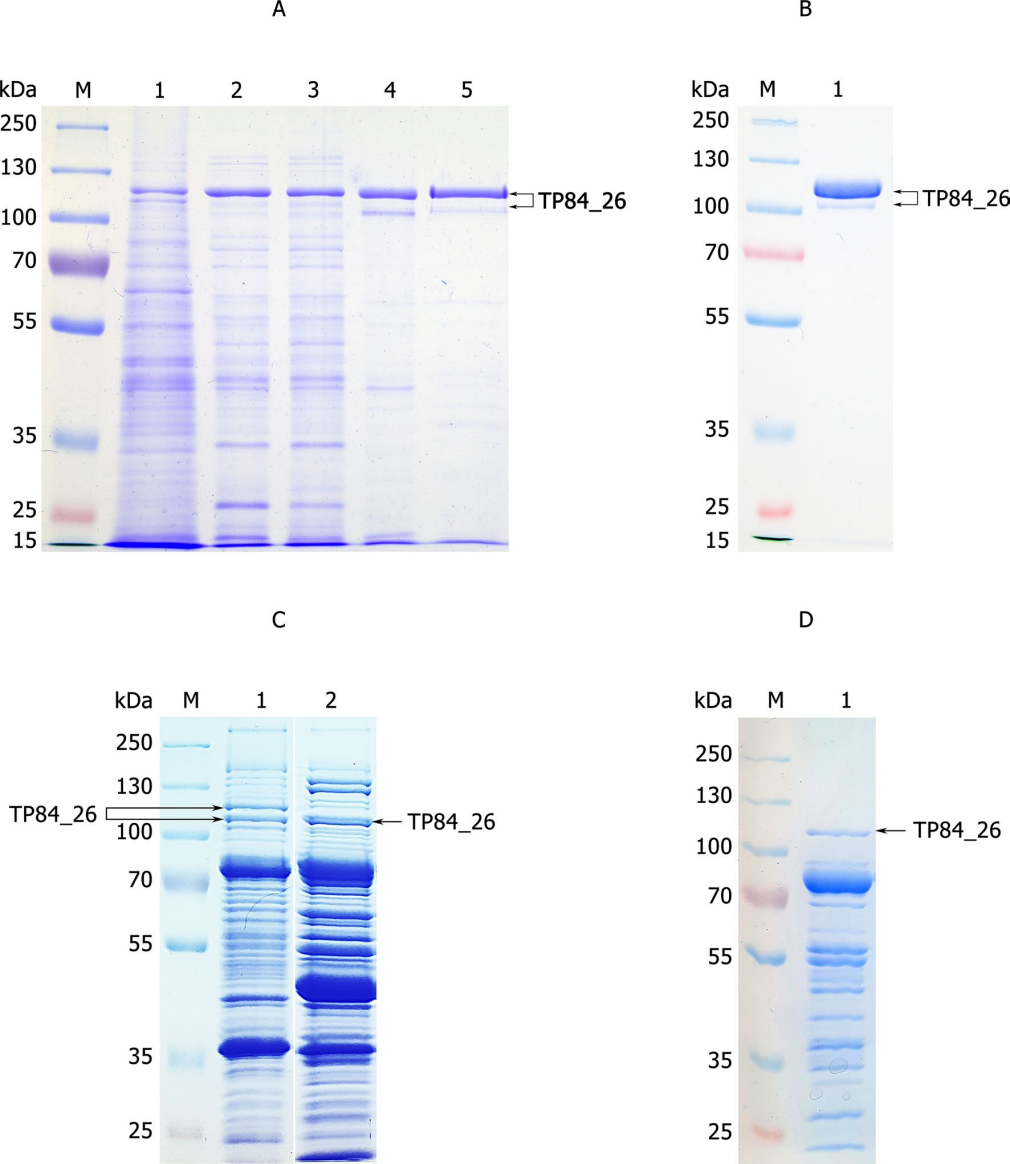
Purification of the depolymerase and other soluble TP-84 proteins from TP-84/*G. stearothermophilus* str^R^ lysates. Protein bands were cut-out and subjected to LC–MS. Bands, corresponding to the depolymerase are marked with arrows. Panel **a**. 10% SDS-PAGE of purification stages (Protocol 1). Lane M, PageRuler™ Plus Prestained Protein Ladder, 10 to 250 kDa; Lane 1, TP-84 TCA-precipitated from 350 μl lysate; Lane 2, depolymerase back-extracted from PEI complexes; Lane 3, flow-through from Phosphocellulose P11*;* Lane 4, combined peak fractions from Q-Sepharose; Lane 5, flow-through from Heparin-Sepharose. Panel **b**. Final depolymerase size fractionation and concentration (Protocol 1). Lane M, PageRuler™ Plus Prestained Protein Ladder, 10 to 250 kDa; Lane 1, TP84_26 depolymerase after VivaSpin® Turbo 15 RC concentration. Panel **c**. Purification of TP-84 proteins using Protocol 2. Lane M, PageRuler™ Plus Prestained Protein Ladder, 10 to 250 kDa; Lane 1, Q-Sepharose chromatography—fraction 100 mM; Lane 2, 200 mM. Panel **d**. Purification of TP-84 proteins using modified Protocol 2, containing CM-Sepharose (negative step), used before Q-Sepharose. Lane M, PageRuler™ Plus Prestained Protein Ladder, 10 to 250 kDa; Lane 1, Q-Sepharose—fraction 500 mM

**Fig. 8:**
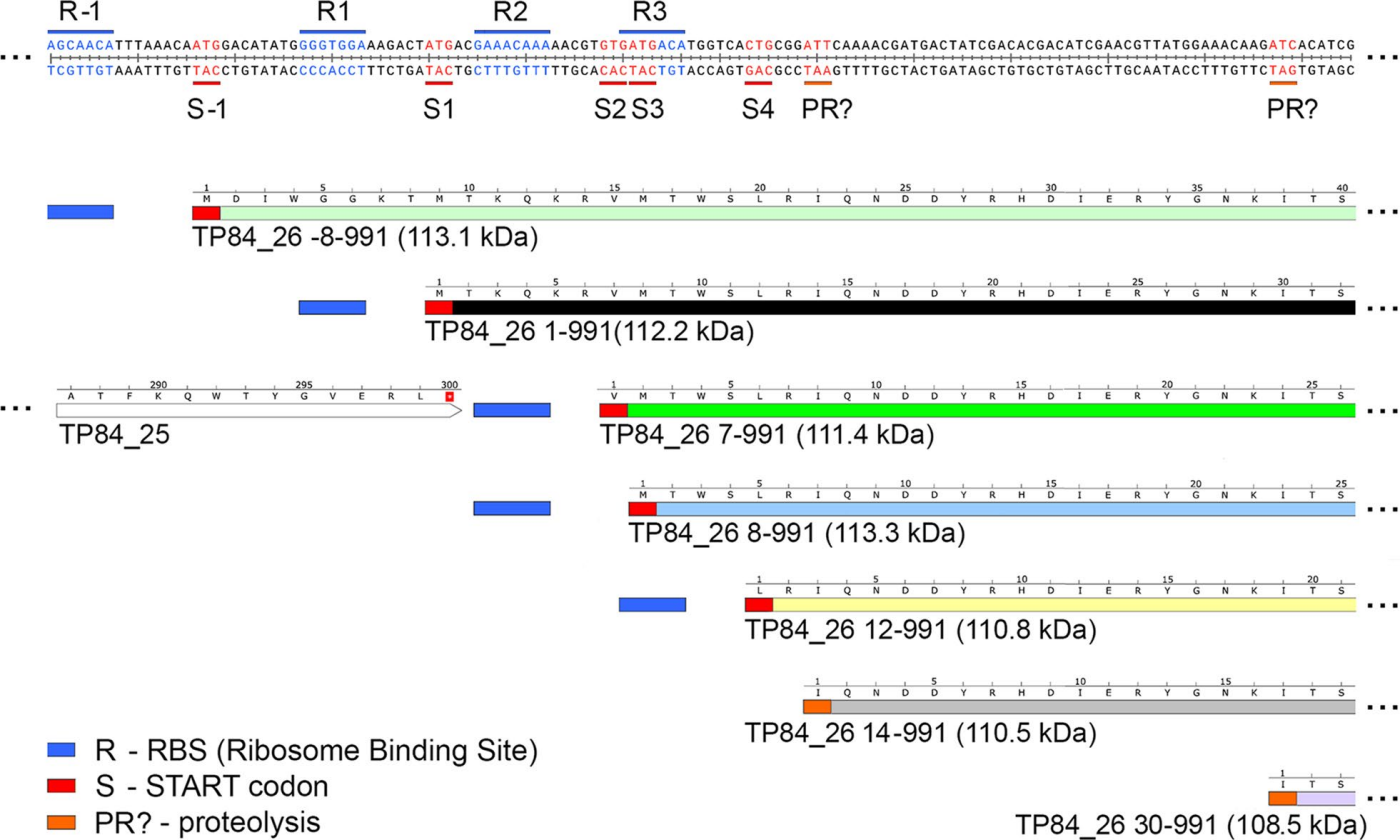
TP84_26 depolymerase coding DNA 5ʹ region nucleotide sequence, translation initiation signals, putative proteolysis cut sites and N-terminal depolymerase protein detected and potential variants aa sequences

**Fig. 9:**
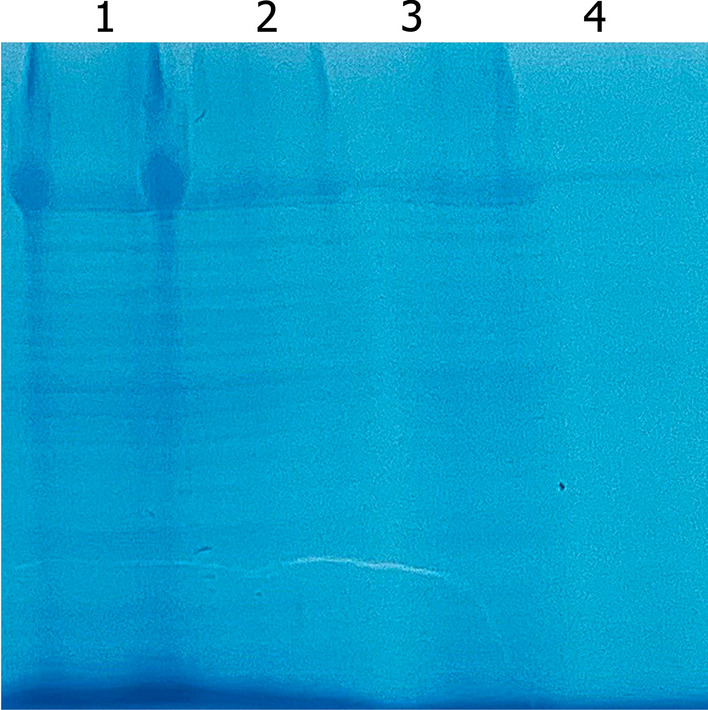
Capsules polysaccharides digestion by depolymerase, detected by Alcian Blue staining assay. Whole *G. stearothermophilus* 10 str^R^ cells were incubated with purified depolymerase. Lane 1, incubation of app. 3 × 10^6^ cells with 1 μg depolymerase for 0 min (control); lane 2, as in lane 1, digestion for 5 min; lane 3, 15 min; lane 4, 45 min

**Fig. 10:**
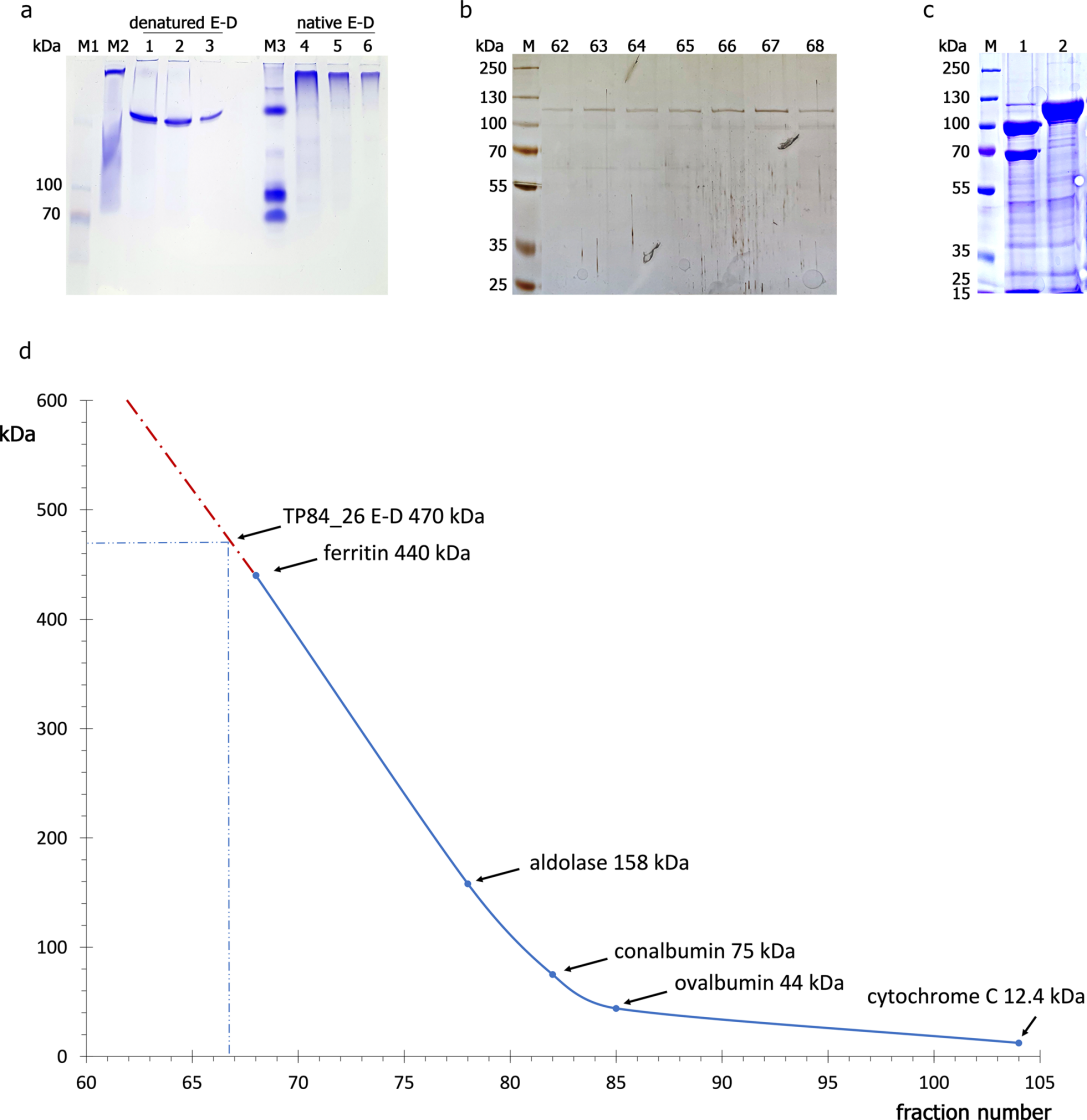
Analysis of TP84_26 depolymerase quaternary composition. Panel **a**. Purified depolymerase electrophoresis (6% PAGE) under native buffer conditions (non-denaturing). Lane M1, PageRuler™ Plus Prestained Protein Ladder; lane 1, TP84_26 SDS-denatured, 24 μg; lane 2, 12 μg; lane 3, 6 μg; Lane M2, GE Healthcare LMW SDS Marker Kit (not heated); lane 4, TP84_26 native (not denatured), 24 μg; lane 5, 12 μg; lane 6, 6 μg. Panel **b**. SDS-PAGE of depolymerase peak fractions from Sephacryl S300 HR column, silver stained. Lane M, PageRuler™ Plus Prestained Protein Ladder, 10 to 250 kDa;  lanes 62 to 68, consecutive Sephacryl S300 HR fractions. Panel **c**. Spontaneous degradation of depolymerase upon changing pH from 6.0 to 8.0. Panel **d**. Molecular sieving of depolymerase on Sephacryl S300, calibrated with 5 mg each of the native markers: ferritin, aldolase, conalbumin, ovalbumin, cytochrome C

The depolymerase production by TP-84 was estimated at app. 1 mg/L, as based on obtained homogeneous preparation (0.4 ml, 2.03 mg/ml) and estimated losses at app. 30–40%. Furthermore, smaller band, a putative truncated depolymerase, was also observed (Fig. 7b). Both bands were cut-out from the gels and subjected to LC–MS, which has shown that the major band (44.1% tryptic peptides coverage) has missing 6 aa from N-terminus (aa 7–991, 111.4 kDa), while the smaller molecular weight band (43.9% coverage) has missing 29 aa residues at the N-terminus (aa 30–991,108.5 kDa). In both variants C-termini are covered completely (Figs. 8 and 9, Additional file 7, 8). It is possible that depolymerase is present in more variants, including the full length, 1–991 aa, 112.2 kDa (Table 1). To confirm that the TP84_26 encoded protein is indeed an enzymatically active depolymerase, the homogeneous preparation was subjected to enzymatic assay targeting polysaccharides, and the reaction products were stained with polysaccharide-specific dye Alcian Blue [14]. Figure 10 shows decrease of Alcian Blue-stainable material correlating with increasing reaction time, thus indicating depolymerase digestion activity toward the *G. stearothermophilus* 10 str^R^ capsules polysaccharides.

**Table 1:** Bacteriophage TP-84_26 potential gene variants and encoded depolymerases properties

Depolymerase variant	CDS length (bp) (including STOP)	Location in the genome (bp)^a^	Polypeptide length (aa)	Predicted polypeptide MW (kDa)	Experimentally determined polypeptide MW (kDa)	Quaternary structure^c^	Predicted isoelectric point
Depolymerase (+ 8 aa)	3000	20155–23154	999	113.1	ND	Tetramer	5.47
Depolymerase full length	2976	20179–23154	991	112.2	115 ± 5^b^	Tetramer	5.49
Depolymerase (-6 aa)	2958	20197–23154	985	111.4	115 ± 5^b^	Tetramer	5.23
Depolymerase (-7 aa)	2955	20200–23154	984	111.3	115 ± 5^b^	Tetramer	5.23
Depolymerase (-11 aa)	2943	20212–23154	980	110.8	ND	Tetramer	5.23
Depolymerase (-13 aa)	2937	20218–23154	978	110.5	ND	Tetramer	5.16
Depolymerase (-29 aa)	2889	20 266–23154	962	108.5	110 ± 5	Tetramer	5.14

^a^as based on several LC–MS analysis, it is conceived that the depolymerase C-terminal sequence is not clipped off for any of the potential variants

^b^resolution of those variants on polyacrylamide gels is within error range of SDS-PAGE

^c^this experiment was conducted using a mixture of depolymerase variants, as obtained from purification procedure

#### Depolymerase partial purification for LC–MS and bioinformatics analysis

For the purposes of continued evaluation of TP-84 proteomics [4], including depolymerase, the soluble TP-84 proteins partial purifications were conducted. The protocols, exemplified by Protocol 2, were devised for use in LC–MS analysis and included several alternatives (not involving PEI) (Fig. 7). It resulted in detection of variants of depolymerase and a number of other previously unconfirmed TP-84 proteins (to be published elsewhere). Depending on the purification scheme one or two bands were visible as located on SDS-PAGE at the expected 112.2 kDa (1–991 aa) molecular weight of depolymerase position (Fig. 7). Bands were cut-out from the gels and subjected to LC–MS, which has shown that the larger molecular weight band (44.1% tryptic peptides coverage) has missing 6 aa from N-terminus (aa 7–991, 111.4 kDa), while the smaller molecular weight band (43.9% coverage) has missing 29 aa residues at the N-terminus (aa 30–991,108.5 kDa). Furthermore, depolymerase was also detected in TP-84 particles, subjected to LC–MS and resulted in determination of another variant with missing 13 aa at the N-terminus: aa 114–991, 110.5 kDa (31% coverage). In all those variants C-termini are covered completely (Fig. 8, Additional files 7, Additional file 8, 9, Table 1). Further, there were detected 2 putative proteolytic forms of molecular weight 110.5 kDa and 108.5 kDa (Table 1). The nucleotide sequence of TP84_26 gene and its upstream region shows potential additional 3 RBSs and 4 START codons in-frame with originally detected [4] TP-84_26 ORF (Fig. 8, Additional file 8). These include: (i) 5ʹ-AGCAACA-3ʹ, located upstream, starting at -9 from potential START codon (ATG) -33 from original ATG codon [4], which would result in translation of larger + 8 aa, 113.1 kDa depolymerase; (ii) 5ʹ- GAAACAAA-3ʹ, at -5 bp from potential internal START codon GTG or -8 from following ATG, which would yield depolymerase variants shorter by 6 aa (111.4 kDa) or 7 aa (111.3 kDa), respectively; (iii) 5ʹ-GATGACA-3ʹ at -7 bp from potential internal START codon CTG, which would yield depolymerase variants shorter by 11 aa (110.8 kDa) (Additional file 10). The stable C-terminus exhibits similarities to C-terminal catalytic domain of glycosyl hydrolases family 18 (GH18, class EC 3.2.1). Thus, based on recent BLASTP and HHpred [5] analysis, containing new data since our original TP-84 analysis [(4)], the closest current annotation of depolymerase would be glycosyl hydrolase or glicoside hydrolase (Additional file 11, 12, 13). Furthermore, putative conserved domains (Yaah superfamily) were detected in the depolymerase protein (Additional file 12, 13), which are associated with *Bacillus subtilis* sporulation and cell wall interaction [29].

#### Depolymerase quaternary structure

The possibility of multimers formation by depolymerase was initially evaluated by running native PAGE, where native and SDS-denatured depolymerase samples exhibited completely different migration behavior (Fig. 10a). Denatured depolymerase migrated deeply into the gel, while not denatured depolymerase barely entered the gel. While this is not a definite proof, such a large difference suggests multimeric depolymerase structure. Further, more precise data came from molecular sieving. The calibrated Sephacryl S300 HR column with a range covering essentially linearly the molecular weight range of depolymerase monomer to oligomers, as one of the high molecular weight markers used was ferritin (440 kDa), anticipated to elute closely to putative depolymerase tetramer. As shown in Fig. 10.b.d.—depolymerase has eluted at 470 kDa tetramer as compared to theoretical 448.8 kDa value for depolymerase tetramer, which is within app. 4.7% error, less than expected for the molecular sieving. Further, during molecular sieving sample preparation using variants of PEI back extraction technique atypical pH instability of depolymerase was observed. Very efficient recovery of depolymerase was seen, when decreasing pH to 6.0 using phosphate elution buffer. Such preparation was stable over month, when stored at 4 °C. Upon adjusting pH to 8, using dialysis or centrifugal dilutions/concentrations, a quantitative splitting of the protein into well-defined two major fragments of molecular weight app. 100 kDa and 68 kDa and a number of smaller bands of less than app. 15 kDa was observed (Fig. 10c, lane 1). To the contrary, protein preparations obtained through the entire purification process conducted in buffers of pH 7.5–8 were stable.

## Discussion

TP-84/*G. stearothermophilus* 10 is an unusual bacteriophage-host system, tolerating temperatures extremes exceptionally well: it exhibits astonishingly wide temperature proliferation range of 31 °C to 79 °C, with indications of growth even beyond this range. Thus the *G. stearothermophilus* 10 and TP-84 form probably the most temperature-universal bacteriophage-host system known thus far. Further, TP-84 proliferation perfectly matches the growth range of the host. This may indicate long history of the TP-84 adjustment to the host’s physiology. Nevertheless, the bacteriophage yields and morphology of its plaques varies with changes in temperature and humidity. To avoid contaminations as well as for a future scaled-up biotechnology applications, the streptomycin-resistant mutant [30] *G. stearothermophilus* 10 was generated. Bacterial colonies, cells morphology, culture growth rate, metabolism did not show any differences and the mutant was used for the studies. As shown in the Fig. 3, small, large, even exceptionally large, multi-layered plaques with or without an iridescent ‘halo’ are observed. Putative TP84_26 depolymerase activity or ‘halo’ activity is either produced or the enzyme retains activity from 46 to 67 °C. Somewhat surprisingly, the range of depolymerase ‘halo’-generating activity is clearly narrower than the growth range of TP-84. Below and above these temperatures, only plaques with no ‘halo’ are observed. The lack of ‘halo’ and apparent substantial decrease in depolymerase activity raises question how much the bacteriophage relies on the enzyme to reach the host’s cell wall? The presence of extensive capsules poses a problem for TP-84 penetration to the host’s cell, nevertheless the capsule is apparently at least partially digested upon infection. There are two temperatures: 46 and 58 °C (Fig. 7), at which the bacteriophage forms plaques with the highest efficiency, which may indicate maxima of the bacteriophage adsorption to the host’s cells. This is correlated with the highest ‘halo’ production. Outside this range TP-84 yields drop 100-fold to thousand folds, which points to important role of depolymerase in TP-84 life cycle. The TP-84 infection course is affected by a number of factors, including: (i) the presence of mono sugars and divalent cation Ca^2+^ and Mg^2+^, which absence can decrease yield hundred folds; (ii) production of very thick capsules—app. 20 times (!) the vegetative cell volume, which is favored by the high sugar content in the medium. Thus, the *G. stearothermophilus* 10 bacteria vegetative cell surrounded by the plasma membrane is actually a small part of the entire microorganism, protected by two additional barriers: thick Gram(+) cell wall and extensive capsule; (iii) the minimum amount of spores, as spores do not support TP-84 infection, this parameter can greatly affect the bacteriophage proliferation kinetics. Furthermore, the presence of the spores adds to the OD_600nm_ readings, which is misleading in M.O.I. calculations and assessment of a culture condition. Thus, we have observed that infections at OD_600nm_ of 0.7 or higher resulted in lower yields, incomplete lysis and often inhibition of the bacteriophage proliferation (not shown). Another strain tested—*G. stearothermophilus* strain NUB3621 [10] was not as efficient host as *G. stearothermophilus* 10 str^R^, however, on this strain recombinant work was shown to be possible [26], thus we have initially evaluated the strain for the purpose of a future recombinant TP-84 constructions. Considering that the capsule is tens of times thicker than the TP-84 particles length, it is interesting how TP-84 manages to reach the host’s cell wall and to inject its genome into the cytoplasm. The precise chemical nature of the capsule (i.e. polysaccharide, polysaccharide-peptide, peptide) was not determined. Nevertheless, depolymerase is currently annotated as glycosyl hydrolase (EC 3.2.1) and exhibits homology to sporulation proteins YdhD and Yaah, which possess conservative motif, associated with proteins interacting with bacterial cell wall and bacteriophage proteins, responsible for cell lysis [29]. Thus, considering the capsules formation-stimulating effect of the sugars in the medium, sensitivity to digestion by TP84_26 depolymerase, annotated as a glycosyl hydrolase, and stainability with polysaccharides-specific dye—Alcian Blue, strongly suggests that indeed polysaccharides form the capsule. Apparently, there exists a mechanism, which allows local capsule disruption to allow TP-84 passage. Since we have purified soluble depolymerase from TP-84/*G. stearothermophilus* 10 str^R^ lysates and since no other TP-84 protein was bioinformatically detected as exhibiting similarities to an enzyme, which might be involved in an capsule depolymerization, we hypothesize that the TP84_26 depolymerase has dual function: (i) it exists as soluble form, released into the medium upon infected host’s cells lysis or is secreted from infected cells. Such depolymerase variant would weaken uninfected bacteria capsules from outside, preparing them for the TP-84 invasions and (ii) the enzyme is also integrated with TP-84 virion in the region of tail fibers and baseplates allowing for direct perforation of the *G. stearothermophilus* capsule at capsule contact point. The hypothesis was verified by Western blotting, using antibodies raised to entire TP-84 virions, which detected depolymerase in TP-84 virions. Besides purification of depolymerase, further confirmation of the function (i) comes from plaques ‘halo’ behavior, as their size increases with incubation time, while clear plaque zone size remains the same for over 7 days. Thus the ‘halo’ must have been caused by a factor diffusing faster (thus being of much smaller molecular weight) through agar than the TP-84 particles. As there is no other candidate coded, it can be concluded that the responsible enzyme is TP84_26 depolymerase. Furthermore, there is an intertwined correlation between maximum ‘halo’ production, sugars addition to the medium, which stimulates both TP-84 yield and capsule biosynthesis, optimal temperature and TP-84 yield. Thus a question arises—perhaps in the course of evolution TP-84 developed a mechanism to use bacteria-protecting capsule to its own advantage, enhancing proliferation and correct hosts selection by means of using capsules as an initial’molecular anchor’ through the virion-associated depolymerase? To verify depolymerase biosynthesis and bioinformatically assigned function and properties, it needed to be purified. Since a bacteriophage proteins are present in cells lysates as very dilute solutions, corresponding to initial culture volume, we have developed a novel approach to isolation of bacteriophage-expressed proteins by reversible PEI precipitation directly from TP-84-infected *G. stearothermophilus* 10 str^R^ lysates. Since some bacteriophages produce nucleases degrading DNA or it undergoes degradation by released host’s nucleases, the amount of PEI added needs to be precisely titrated to obtain co-precipitation with a protein of interest. PEI, being long aliphatic chain polymer with charged amino and imino groups is uniquely suited to formation of insoluble salts with DNA, RNA and acidic proteins. As PEI is not harmful to proteins, it is being used in procedures that remove DNA, RNA and acidic proteins from bacterial lysates in two variants, which are PEI concentration and ionic strength dependent [31]. In low salt and limited PEI concentrations mixed precipitates form, comprised of DNA, RNA, DNA- and RNA-interacting proteins and those, which have low isoelectric point. In higher salt solutions it is possible to precipitate DNA and RNA only, while leaving proteins in supernatant for further processing. Those two variants are thus far used on highly concentrated, disintegrated cells lysates of manyfold lower volume than original microbial culture. To the contrary, depolymerase was isolated and concentrated from large volumes of the bacteriophage lysates using PEI, followed by chromatography. Furthermore, this PEI purification variant does not require further ammonium sulfate protein precipitation, used in standard protocols for removal of remaining soluble PEI, which interferes with a subsequent chromatography. Highly pure preparation was obtained after PEI stage, containing app. 50% of depolymerase. Such efficiency is rarely achievable in purification of native proteins (non-recombinant). Thus the proposed specialized PEI procedure can be useful for more general applications in scientific and industrial scaled-up protein purification. Furthermore, the PEI procedure for a bacteriophage lysates can be also used in ‘negative’ mode, by precipitation of nucleic acids in the presence of higher salt concentration, while leaving a bacteriophage protein of interest in supernatant. SDS-PAGE, LC–MS of the homogeneous depolymerase and TP84_26 gene nucleotide sequence analysis revealed that the enzyme may be present in several forms, shorter or longer as compared to the bioinformatically assigned full length enzyme (1–991 aa) [4] (Figs. 8, 9, Table 1, Additional file 7, 8). There are 5 in-frame potential START codons and 4 ribosome binding sites around the coding region of putative N-terminus of depolymerase. In the absence of N-terminal sequencing data, the lack of complete N-termini maybe a result of not complete coverage with tryptic peptides during LC–MS sample preparation. On the other hand, complete coverage of C-termini indicates that there is no protein processing in this location. However, LC–MS results are matching SDS-PAGE-detected the smaller molecular weight band 30–991 aa (108.6 kDa), which suggests that it may be a product of proteolytic processing at the N-terminus. Either alternative translation initiation at downstream GTG START codon or proteolytic processing is possible in the case of the larger band, exhibiting missing 6 aa at the N-terminus in LC–MS. Furthermore, LC–MS analysis of entire TP-84 virions has shown missing 13 aa from the N-terminus of detected depolymerase. The significance of the variants remains to be determined, nevertheless it is possible that a maturation processes are involved, depending on the enzyme destination. Further question concerning depolymerase functionality in vivo arises: how TP-84 controls diffusion of soluble depolymerase to ensure effective local enzyme concentration to digest capsules in the closest neighborhood, prior to the bacteriophage attachment? Too fast diffusion would dilute the depolymerase enzyme below its biologically functional level. Observing the kinetics of ‘halo’ expansion, one can conclude that this diffusion is relatively slow, even though agar has large gel pores. We hypothesize that TP-84 uses depolymerase multimerization as a mechanism of slowing down diffusion by increasing the native molecular size of the depolymerase, which already is the largest TP-84 protein. Indeed, as shown in Fig. 10 the tetramer of depolymerase is present as the only species detected during molecular sieving and native gel electrophoresis. We further hypothesize that this mechanism, allowing for initial pre-digestion of large capsules of uninfected host’s cells, located in the neighborhood of uninfected cells, is enhancing sequential TP-84 invasions. Further size increase may come from complexing with other proteins. Nevertheless, even such multimer or complex would diffuse faster than TP-84 bacteriophage of molecular weight in the range of 50 MDa, thus ‘halo’ is being generated. Additional unexpected observation was made that overall stable depolymerase protein, while stored at 4 °C, rapidly degrades to defined fragments upon pH buffer change from 6.0 to 8.0. While this phenomenon has not been further explored, it may suggest that depolymerase has a fragile polypeptide/domain structure.

## Conclusions

1. Novel, efficient variant of PEI-mediated proteins purification from diluted samples has been developed, suitable for biotechnology scale production.
2. TP84_26 depolymerase has been purified to homogeneity and characterized.
3. The native depolymerase forms a tetramer of app. 470 kDa apparent molecular weight. We hypothesize that this a mechanism, allowing for initial prdigestion of uninfected host’s cells capsules, located in the neighborhood of infected cells.
4. TP84_26 polypeptide depolymerase apparently exists in several forms, trimmed at the N-terminus, which may be responsible for 2 functions: soluble form for weakening capsules of uninfected cells, while virion-bound form for making TP-84 passage to the host’s cell wall. TP-84 is an emerging model thermophilic bacteriophage, exhibiting extraordinary wide temperature range of proliferation of 31 °C—79 °C.
5. The efficient host—streptomycin-resistant mutant of *G. stearothermophilus* 10 was constructed, yielding TP-84 PFUs exceeding 10^12^ / ml.
6. Microbiological aspects of the TP-84-host interaction have been determined.
7. The TP-84 yield is critically dependent on the presence of mono sugars, divalent cation Ca^2+^, Mg^2+^ and on minimizing amount of the host’s spores in infected culture.

### Supplementary Information

**Additional file 1:** Time course of G. stearothermophilus 10 strR bacteria growth in liquid culture.

**Additional file 2:** Growth curve of G. stearothermophilus 10 strR in TYM medium supplemented with 50 µg/ml streptomycin, at 55oC.

**Additional file 3:** Time course of G. stearothermophilus 10 strR bacteria growthin liquid TYM medium.

**Additional file 4:** The growth curve comparison of the uninfected and the TP-84-infected G. stearothermophilus 10 strR cultures.

**Additional file 5:** TP-84 bacteriophage titers determination upon infections of G. stearothermophilus 10 strR, at various temperatures in the range of 31-79oC.

**Additional file 6:** TP-84 / G. stearothermophilus 10 strR cell lysates titration with 1% buffered PEI solution.

**Additional file 7:** Combined results of depolymerase LC-MS analysis.

**Additional file 8:** Detected and putative depolymerase N-termini variants.

**Additional file 9:** Combined LC-MS analysis of depolymerase.

**Additional file 10:** LC-MS peptides coverage.

**Additional file 11:** Results of the protein similarity search using BlastP.

**Additional file 12:** Putative conserved domains detected for the capsuldepolymeraseepolymerase.

**Additional file 13:** Results of homology detection and structure prediction the TP-84 capsuldepolymeraseepolymerase, performed by HMM-HMM comparison.

## Data Availability

All the data needed to repeat experiments are present in the manuscript and Additional files.
