# Correction: A novel thermostable TP-84 capsule depolymerase: a method for rapid polyethyleneimine processing of a bacteriophage-expressed proteins

**DOI:** 10.1186/s12934-023-02157-4

**Published:** 2023-08-03

**Authors:** Beata Łubkowska, Edyta Czajkowska, Aleksandra Stodolna, Michał Sroczyński, Agnieszka Zylicz-Stachula, Ireneusz Sobolewski, Piotr M. Skowron

**Affiliations:** 1 Faculty of Health and Life Sciences, Division of Biochemistry, Gdansk University of Physical Education and Sport, Gorskiego 1, Gdansk, 80-336 Poland; 2 Faculty of Chemistry, Department of Molecular Biotechnology, University of Gdansk, Wita Stwosza 63, Gdansk, 80-308 Poland; 3 BioGel Sp. z o.o. (Ltd.), ul. Promienista 83, Poznań, 60-141 Poland

**Microbial Cell Factories (2023) 22:80**

10.1186/s12934-023-02086-2

In the original publication of the article, the given and family names of the authors were swapped. The original article has been corrected.

