# Supplementary material for: A novel thermostable TP-84 capsule depolymerase: a method for rapid polyethyleneimine processing of a bacteriophage-expressed proteins
Source: Microb Cell Fact. 2023 Apr 25;22:80. doi: 10.1186/s12934-023-02086-2 (PMC10131341; doi:10.1186/s12934-023-02086-2)
Supplement: Supplementary file 1 — Additional file 1: Time course of G. stearothermophilus 10 strR bacteria growth in liquid culture. [file 12934_2023_2086_MOESM1_ESM.docx]

**Additional file 1**

Time course of *G. stearothermophilus* 10 str^R^ bacteria growth in liquid culture in TYM medium, supplemented with 50 µg/ml streptomycin, at 55^o^C with vigorous aeration.

| **Time [h]** | **Turbidity OD_600nm_** | **Viable count**  **[cells/mL]** |
| --- | --- | --- |
| 1 | 0.003 | 8*10^4^ |
| 2 | 0.007 | 9*10^4^ |
| 3 | 0.03 | 3*10^5^ |
| 4 | 0.1 | 2.4*10^6^ |
| 4,5 | 0.3 | 1.8* 10^7^ |
| 5 | 0.6 | 2.8*10^7^ |
| 5,5 | 0.9 | 5.3*10^7^ |
| 6,5 | 1.5 | 9*10^7^ |
| 7 | 1.9 | 4*10^8^ |
| 7,5 | 2.3 | 3.7*10^8^ |
| 8 | 2.5 | 1.6*10^8^ |
| 9 | 2.5 | 1.5*10^8^ |
| 10 | 2.5 | 1.5*10^8^ |
| 11 | 2.0 | 1.4*10^8^ |
