# Supplementary material for: A novel thermostable TP-84 capsule depolymerase: a method for rapid polyethyleneimine processing of a bacteriophage-expressed proteins
Source: Microb Cell Fact. 2023 Apr 25;22:80. doi: 10.1186/s12934-023-02086-2 (PMC10131341; doi:10.1186/s12934-023-02086-2)
Supplement: Supplementary file 3 — Additional file 3: Time course of G. stearothermophilus 10 strR bacteria growthin liquid TYM medium. [file 12934_2023_2086_MOESM3_ESM.docx]

**Additional file 3**

| **Time [h]** | **Turbidity of uninfected culture, OD_600nm_** | **Turbidity of**  **TP-84-infected culture,**  **OD_600nm_** |
| --- | --- | --- |
| 0 | 0.002 | 0.089 |
| 1 | 0.012 | 0.115 |
| 2 | 0.063 | 0.193 |
| 3 | 0.123 | 0.239 |
| **4** | 0.481 | **0.535** |
| 5 | 0.769 | 0.140 |
| 6 | 1.071 | 0.111 |

Time course of *G. stearothermophilus* 10 str^R^ bacteria growth (uninfected and infected with TP-84 bacteriophage at M.O.I = 1) in liquid TYM medium, supplemented with 50 µg/ml streptomycin, at 55^o^C with vigorous aeration. Marked red: TP-84 infection.
