# Supplementary material for: A novel thermostable TP-84 capsule depolymerase: a method for rapid polyethyleneimine processing of a bacteriophage-expressed proteins
Source: Microb Cell Fact. 2023 Apr 25;22:80. doi: 10.1186/s12934-023-02086-2 (PMC10131341; doi:10.1186/s12934-023-02086-2)
Supplement: Supplementary file 4 — Additional file 4: The growth curve comparison of the uninfected and the TP-84-infected G. stearothermophilus 10 strR cultures. [file 12934_2023_2086_MOESM4_ESM.docx]

**Additional file 4**


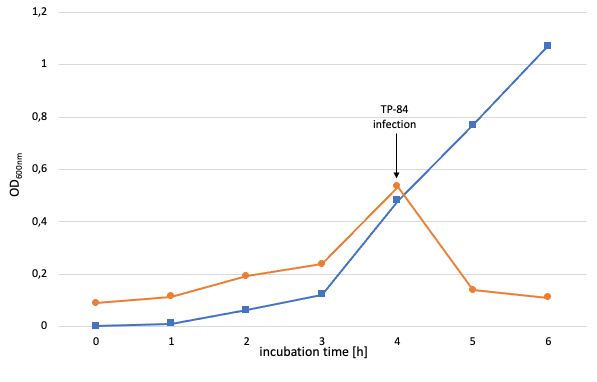


Logarithmic growth curves of uninfected and TP-84-infected (M.O.I=1) *G. stearothermophilus* 10 str^R^. Cultures grown in TYM medium supplemented with 50 µg/ml streptomycin at 55^o^C with vigorous shaking. Data recorded by UV/Visible Spectrophotometer JENWAY^®^ 7205 converted to OD_600nm_ at 30-min and 60-min intervals.
