# Supplementary material for: A novel thermostable TP-84 capsule depolymerase: a method for rapid polyethyleneimine processing of a bacteriophage-expressed proteins
Source: Microb Cell Fact. 2023 Apr 25;22:80. doi: 10.1186/s12934-023-02086-2 (PMC10131341; doi:10.1186/s12934-023-02086-2)
Supplement: Supplementary file 5 — Additional file 5: TP-84 bacteriophage titers determination upon infections of G. stearothermophilus 10 strR, at various temperatures in the range of 31-79oC. [file 12934_2023_2086_MOESM5_ESM.docx]

**Additional file 5**

| **Incubation temperature [°C]** | **Dilution at which the plaques were counted** | **Number of plaques** | **TP-84 count [PFU/ml]** |
| --- | --- | --- | --- |
| 31 | 10^-3^ | 16 | 3.2x10^6^ |
| 34 | 10^-3^ | 12 | 2.4x10^6^ |
| 37 | 10^-4^ | 7 | 1.4x10^7^ |
| 40 | 10^-5^ | 5 | 1.0x10^8^ |
| 43 | 10^-6^ | 38 | 7.6x10^8^ |
| 46 | 10^-6^ | 92 | 1.8x10^9^ |
| 49 | 10^-7^ | 58 | 1.1x10^10^ |
| 52 | 10^-7^ | 23 | 4.6x10^10^ |
| 55 | 10^-7^ | 13 | 2.6x10^10^ |
| 58 | 10^-8^ | 55 | 1.1x10^12^ |
| 61 | 10^-7^ | 31 | 6.2x10^9^ |
| 64 | 10^-7^ | 19 | 3.8x10^9^ |
| 67 | 10^-7^ | 12 | 2.4x10^9^ |
| 70 | 10^-6^ | 55 | 1.1x10^9^ |
| 73 | 10^-5^ | 27 | 5.4x10^8^ |
| 76 | 10^-3^ | 15 | 3.0x10^6^ |
| 79 | 10^-3^ | 4 | 8.0x10^5^ |

TP-84 bacteriophage titers determination upon infections of *G. stearothermophilus* 10 str^R^, at various temperatures in the range of 31-79^o^C. Cultures were grown in TYM liquid media, supplemented with 50 µg/ml streptomycin, with vigorous aeration. Serial dilutions were plated on TYM solid media supplemented with 50 µg/ml streptomycin and incubated at 55^o^C.
