# Supplementary material for: A novel thermostable TP-84 capsule depolymerase: a method for rapid polyethyleneimine processing of a bacteriophage-expressed proteins
Source: Microb Cell Fact. 2023 Apr 25;22:80. doi: 10.1186/s12934-023-02086-2 (PMC10131341; doi:10.1186/s12934-023-02086-2)
Supplement: Supplementary file 6 — Additional file 6: TP-84 / G. stearothermophilus 10 strR cell lysates titration with 1% buffered PEI solution. [file 12934_2023_2086_MOESM6_ESM.docx]

**Additional file 6**

| **Sample number** | **Amount of 1% PEI solution added [µl]** | **Turbidity [OD_600nm_]** |
| --- | --- | --- |
| 1 | 5 | 0.14 |
| 2 | 10 | 0.17 |
| 3 | 20 | 0.24 |
| 4 | 35 | 0.26 |
| 5 | 50 | 0.31 |
| 6 | 70 | 0.25 |
| 7 | 100 | 0.42 |
| 8 | 150 | 0.69 |
| **9** | **200** | **0.80** |
| 10 | 300 | 0.65 |

TP-84 / *G. stearothermophilus* 10 str^R^ cell lysates titration with 1% buffered PEI solution. Marked bold: the amount of 1% PEI solution selected for the E-D purification.
