# Supplementary material for: A novel thermostable TP-84 capsule depolymerase: a method for rapid polyethyleneimine processing of a bacteriophage-expressed proteins
Source: Microb Cell Fact. 2023 Apr 25;22:80. doi: 10.1186/s12934-023-02086-2 (PMC10131341; doi:10.1186/s12934-023-02086-2)
Supplement: Supplementary file 10 — Additional file 10: LC-MS peptides coverage. [file 12934_2023_2086_MOESM10_ESM.docx]

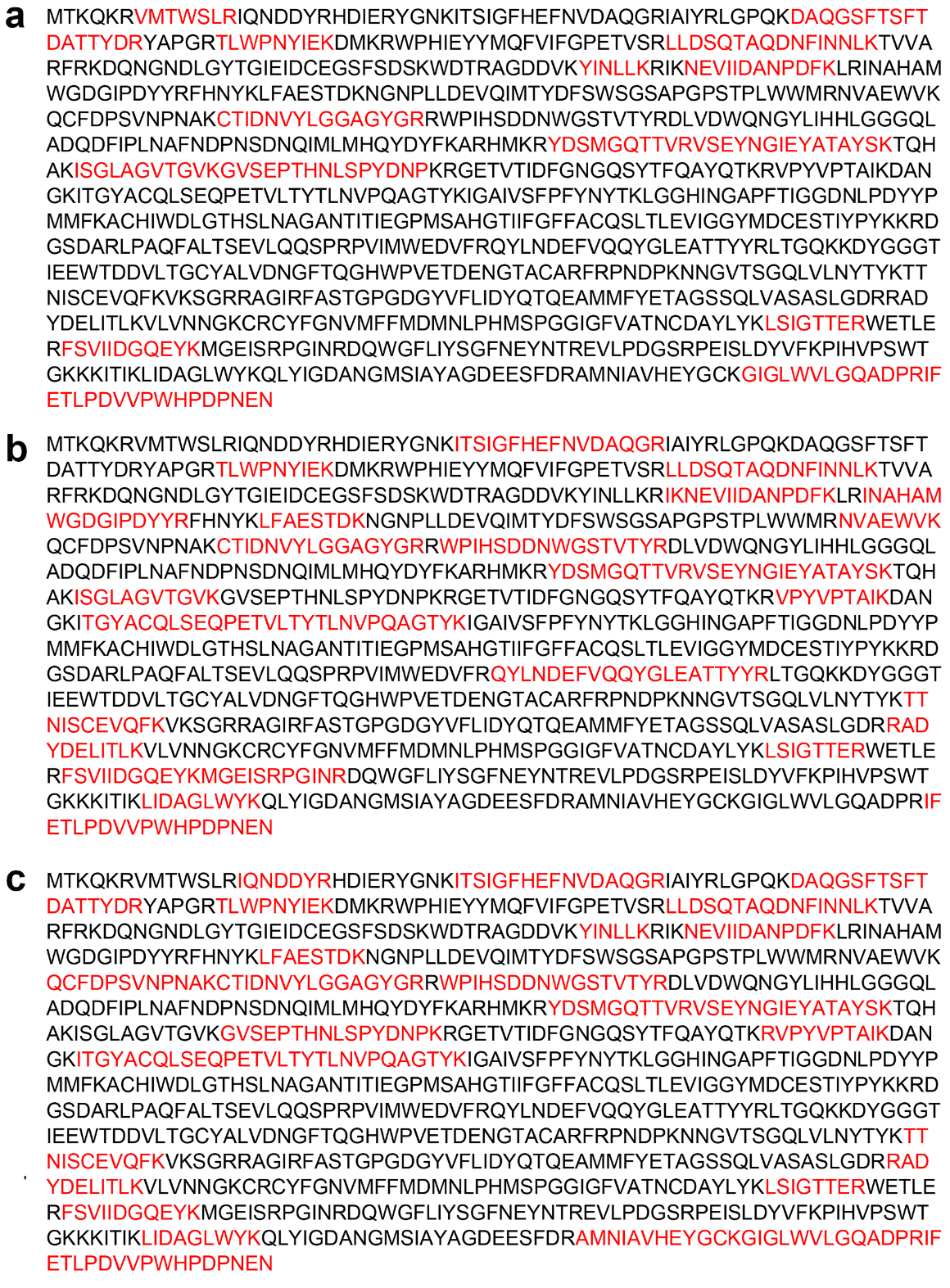


Comparative LC-MS analysis of depolymerase variants. Panel A. Tryptic peptides distribution of the largest molecular weight depolymerase protein variant band on SDS-PAGE, eluted from Q-Sepharose, using Protocol 2 overlayed on bioinformatically detected full-length depolymerase aa sequence [4]. Panel B. As in Panel A, smaller molecular weight depolymerase protein variant band analysis. Panel C. Tryptic peptides distribution of the depolymerase variant band from SDS-PAGE of CsCl-purified TP-84 virions.
