# Supplementary material for: A novel thermostable TP-84 capsule depolymerase: a method for rapid polyethyleneimine processing of a bacteriophage-expressed proteins
Source: Microb Cell Fact. 2023 Apr 25;22:80. doi: 10.1186/s12934-023-02086-2 (PMC10131341; doi:10.1186/s12934-023-02086-2)
Supplement: Supplementary file 11 — Additional file 11: Results of the protein similarity search using BlastP. [file 12934_2023_2086_MOESM11_ESM.pdf]

BLAST ® » **blastp suite** » results for RID-TZTH2RN301N

Your results are filtered to match records with percent identity between 30 and 100.

Job Title	Protein Sequence ...
RID	TZTH2RN301N Search expires on 12-20 11:18 am
Program	BLASTP
Database	nr
Query ID	lcl|Query_478613
Description	unnamed protein product ...
Molecule type	amino acid
Query Length	1000

Descriptions

Description	Scientific Name	Max Score	Total Score	Query Cover	E value	Per. Ident	Acc. Len	Accession
glycosylase [Geobacillus phage TP-84]	Geobacillus phage TP-84	2085	2085	99%	0.0	100.00%	991	YP_009600071.1
glycosyl hydrolase [Geobacillus phage vB_GthS_PK2.1]	Geobacillus phage vB_GthS_PK2.1	2007	2007	99%	0.0	95.36%	991	UYL93853.1
putative glycosyl hydrolase [Bacillus phage vB_BpsM-61]	Bacillus phage vB_BpsM-61	1200	1200	98%	0.0	57.52%	1040	AYP68839.1
hypothetical protein [Bacillus andreraoultii]	Bacillus andreraoultii	707	707	98%	0.0	39.13%	1003	WP_033829088.1
hypothetical protein [Bacillus cereus group]	Bacillus cereus group	680	680	98%	0.0	39.84%	984	WP_205173220.1
hypothetical protein [Bacillus cereus]	Bacillus cereus	677	677	98%	0.0	39.65%	984	WP_071740026.1
hypothetical protein [Bacillus mobilis]	Bacillus mobilis	674	674	98%	0.0	39.55%	984	WP_229133922.1
hypothetical protein [Bacillus cereus group sp. BfR-BA-01489]	Bacillus cereus group sp. BfR-BA-01489	674	674	98%	0.0	39.55%	984	WP_242317101.1
hypothetical protein [Alkaliphilus peptidifermentans]	Alkaliphilus peptidifermentans	649	649	98%	0.0	38.34%	953	WP_091545053.1
hypothetical protein [Marinococcus halophilus]	Marinococcus halophilus	577	577	97%	0.0	34.94%	1089	WP_094907759.1
hypothetical protein [Halobacillus ihumii]	Halobacillus ihumii	566	566	97%	0.0	34.46%	1089	WP_163530905.1
glycoside hydrolase [Geobacillus phage GBK2]	Geobacillus phage GBK2	451	451	96%	2e-138	33.46%	988	YP_009010491.1
glycoside hydrolase [Bacillus wiedmannii]	Bacillus wiedmannii	447	447	98%	1e-136	31.68%	1038	PHE70510.1
glycosyl hydrolase family 18 protein [Bacillus wiedmannii]	Bacillus wiedmannii	444	444	95%	2e-135	32.33%	1030	WP_180236340.1
glycoside hydrolase [Halalkalibacterium halodurans]	Halalkalibacterium halodurans	441	441	95%	5e-135	32.86%	954	WP_053432347.1
glycoside hydrolase [Halalkalibacterium halodurans]	Halalkalibacterium halodurans	439	439	95%	2e-134	32.76%	954	WP_134229696.1
glycoside hydrolase [Halalkalibacterium halodurans]	Halalkalibacterium halodurans	438	438	95%	6e-134	32.79%	954	WP_010896201.1
glycosyl hydrolase family 18 protein [Bacillus wiedmannii]	Bacillus wiedmannii	439	439	95%	2e-133	32.02%	1028	WP_098822181.1
glycoside hydrolase [Priestia megaterium]	Priestia megaterium	436	436	95%	1e-132	31.58%	1020	WP_098896642.1
glycosyl hydrolase family 18 protein [Bacillus wiedmannii]	Bacillus wiedmannii	436	436	95%	2e-132	31.92%	1028	WP_098128560.1
glycosyl hydrolase family 18 protein [Corallococcus sp. AB032C]	Corallococcus sp. AB032C	433	433	95%	8e-132	33.64%	967	WP_121781250.1
glycoside hydrolase [Siminovitchia thermophila]	Siminovitchia thermophila	426	426	97%	5e-129	30.25%	996	WP_205180204.1
glycoside hydrolase [Geobacillus thermodenitrificans]	Geobacillus thermodenitrificans	424	424	97%	3e-128	31.31%	975	WP_236934189.1
glycosyl hydrolase family 18 protein [Halalkalibacterium halodurans]	Halalkalibacterium halodurans	421	421	95%	2e-127	31.98%	957	WP_010899642.1
glycoside hydrolase [Geobacillus thermodenitrificans]	Geobacillus thermodenitrificans	420	420	97%	7e-127	31.36%	980	WP_100660665.1
glycoside hydrolase [Geobacillus sp. 47C-IIb]	Geobacillus sp. 47C-IIb	419	419	97%	1e-126	31.36%	961	WP_190300669.1
hypothetical protein [Geobacillus sp. 47C-IIb]	Geobacillus sp. 47C-IIb	419	419	97%	2e-126	31.36%	980	WP_081157451.1
hypothetical protein APP_29760 [Aeribacillus pallidus]	Aeribacillus pallidus	417	417	95%	4e-126	31.88%	956	BBU40684.1
glycosyl hydrolase family 18 protein [Geobacillus sp. LEMMY01]	Geobacillus sp. LEMMY01	417	417	95%	7e-126	32.39%	963	WP_079936620.1
glycoside hydrolase [Phage Altai3]	Phage Altai3	416	416	95%	2e-125	31.71%	957	QBX91082.1
glycoside hydrolase [Caldibacillus debilis]	Caldibacillus debilis	415	415	95%	5e-125	31.57%	976	REJ18637.1
glycosyl hydrolase family 18 protein [Geobacillus stearothermophilus]	Geobacillus stearothermophilus	415	415	95%	5e-125	32.65%	963	WP_053532686.1
glycosyl hydrolase family 18 protein [Geobacillus icigianus]	Geobacillus icigianus	414	414	95%	1e-124	32.42%	963	WP_033020082.1
glycosyl hydrolase family 18 protein [Geobacillus sp. PA-3]	Geobacillus sp. PA-3	414	414	95%	1e-124	32.25%	963	WP_060476140.1
glycosyl hydrolase family 18 protein [Geobacillus thermodenitrificans]	Geobacillus thermodenitrificans	414	414	95%	1e-124	32.25%	963	WP_011888047.1
glycosyl hydrolase family 18 protein [Geobacillus sp. MR]	Geobacillus sp. MR	414	414	95%	1e-124	32.25%	963	WP_171355563.1
glycosyl hydrolase family 18 protein [Geobacillus sp. 46C-IIa]	Geobacillus sp. 46C-IIa	414	414	95%	1e-124	32.25%	963	WP_081207182.1
glycosyl hydrolase family 18 protein [Geobacillus thermodenitrificans]	Geobacillus thermodenitrificans	414	414	95%	1e-124	32.25%	963	WP_099233472.1
glycosyl hydrolase family 18 protein [Geobacillus sp. C56-T3]	Geobacillus sp. C56-T3	413	413	95%	2e-124	32.39%	963	WP_013144109.1
glycosyl hydrolase family 18 protein [Geobacillus stearothermophilus]	Geobacillus stearothermophilus	413	413	95%	3e-124	32.24%	963	WP_193442733.1
glycosyl hydrolase family 18 protein [Geobacillus stearothermophilus]	Geobacillus stearothermophilus	412	412	96%	4e-124	32.03%	963	WP_121678181.1
glycosyl hydrolase family 18 protein [Geobacillus stearothermophilus]	Geobacillus stearothermophilus	411	411	95%	1e-123	32.28%	963	WP_095859845.1
glycosyl hydrolase family 18 protein [Geobacillus stearothermophilus]	Geobacillus stearothermophilus	411	411	95%	1e-123	32.59%	963	WP_049626260.1

Description	Scientific Name	Max Score	Total Score	Query Cover	E value	Per. Ident	Acc. Len	Accession
glycosyl hydrolase family 18 protein [Geobacillus stearothermophilus]	Geobacillus stearothermophilus	409	409	95%	1e-122	32.11%	963	**WP_061567232.1**
glycosyl hydrolase family 18 protein [Geobacillus zalihae]	Geobacillus zalihae	408	408	95%	2e-122	32.25%	963	**WP_081132806.1**
glycosyl hydrolase family 18 protein [Alkalihalobacillus lehensis]	Alkalihalobacillus lehensis	387	387	95%	1e-114	30.23%	949	**WP_244877264.1**
glycosyl hydrolase family 18 protein [Alkalihalobacillus miscanthi]	Alkalihalobacillus miscanthi	385	385	95%	3e-114	30.03%	947	**WP_144560553.1**
glycoside hydrolase [Bacillus sp. C1-1]	Bacillus sp. C1-1	385	385	95%	3e-114	30.03%	947	**RQW19191.1**
glycoside hydrolase [Shouchella plakortidis]	Shouchella plakortidis	382	382	95%	5e-113	30.03%	947	**KQL57742.1**
glycosyl hydrolase family 18 protein [Bacillaceae]	Bacillaceae	382	382	95%	5e-113	30.03%	949	**WP_236702391.1**
