# Supplementary material for: A novel thermostable TP-84 capsule depolymerase: a method for rapid polyethyleneimine processing of a bacteriophage-expressed proteins
Source: Microb Cell Fact. 2023 Apr 25;22:80. doi: 10.1186/s12934-023-02086-2 (PMC10131341; doi:10.1186/s12934-023-02086-2)
Supplement: Supplementary file 12 — Additional file 12: Putative conserved domains detected for the capsuldepolymeraseepolymerase. [file 12934_2023_2086_MOESM12_ESM.pdf]

Conserved domains on [lcl|Query_159537]

View Standard Results

Local query sequence

Graphical summary Zoom to residue level show extra options »

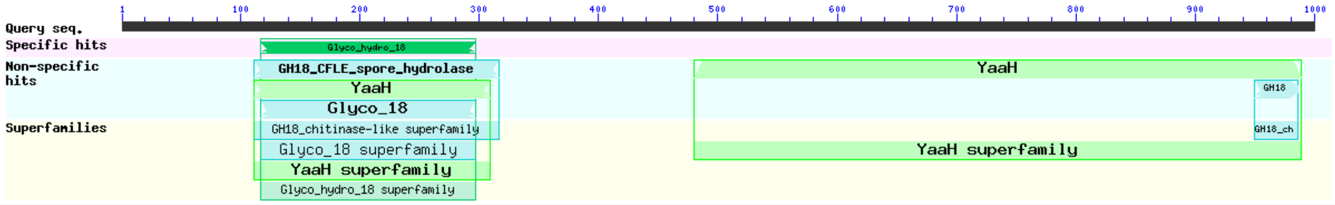

Search for similar domain architectures | Refine search

List of domain hits

Name	Accession	Description	Interval	E-value
GH18_CFLE_spore_hydrolase	cd02874	Cortical fragment-lytic enzyme (CFLE) is a peptidoglycan hydrolase involved in bacterial ...	111-317	1.25e-15
YaaH	COG3858	Spore germination protein YaaH [Cell cycle control, cell division, chromosome partitioning];	480-989	8.24e-12
YaaH	COG3858	Spore germination protein YaaH [Cell cycle control, cell division, chromosome partitioning];	111-309	1.17e-10
Glyco_hydro_18	pfam00704	Glycosyl hydrolases family 18;	117-297	4.00e-05
Glyco_18	smart00636	Glyco_18 domain;	117-297	4.02e-04
GH18_CFLE_spore_hydrolase	cd02874	Cortical fragment-lytic enzyme (CFLE) is a peptidoglycan hydrolase involved in bacterial ...	950-986	8.48e-03

Blast search parameters

Data Source: Live blast search **RID** = TZTH2MRS01N

User Options: **Database:** CDSEARCH/cdd **Low complexity filter:** yes **Composition Based Adjustment:** yes **E-value threshold:** 0.01 **Maximum number of hits:** 500

References:

- Marchler-Bauer A et al. (2017), *"CDD/SPARCLE: functional classification of proteins via subfamily domain architectures."*, **Nucleic Acids Res.45**(D)200-3.
- Marchler-Bauer A et al. (2015), *"CDD: NCBI's conserved domain database."*, **Nucleic Acids Res.43**(D)222-6.
- Marchler-Bauer A et al. (2011), *"CDD: a Conserved Domain Database for the functional annotation of proteins."*, **Nucleic Acids Res.39**(D)225-9.
- Marchler-Bauer A, Bryant SH (2004), *"CD-Search: protein domain annotations on the fly."*, **Nucleic Acids Res.32**(W)327-331.
