# Supplementary material for: A novel thermostable TP-84 capsule depolymerase: a method for rapid polyethyleneimine processing of a bacteriophage-expressed proteins
Source: Microb Cell Fact. 2023 Apr 25;22:80. doi: 10.1186/s12934-023-02086-2 (PMC10131341; doi:10.1186/s12934-023-02086-2)
Supplement: Supplementary file 13 — Additional file 13: Results of homology detection and structure prediction the TP-84 capsuldepolymeraseepolymerase, performed by HMM-HMM comparison. [file 12934_2023_2086_MOESM13_ESM.pdf]

Sign In

HHpred

Job ID: 8363653_2, Parent Job ID: 8363653_1, Created: 3 minutes ago

Input | Parameters | Results | Raw Output | Probability Plot | Query Template MSA | Query MSA

Vis Hits Aln | Select All Forward Forward Query A3M Model using selection Download HHR Color Seqs Wrap Seqs

Number of Hits: **81**
Query MSA diversity (Neff): **1**

Note: your query alignment consists of only 1 sequence(s).

Visualization

Resubmit Section

11 317

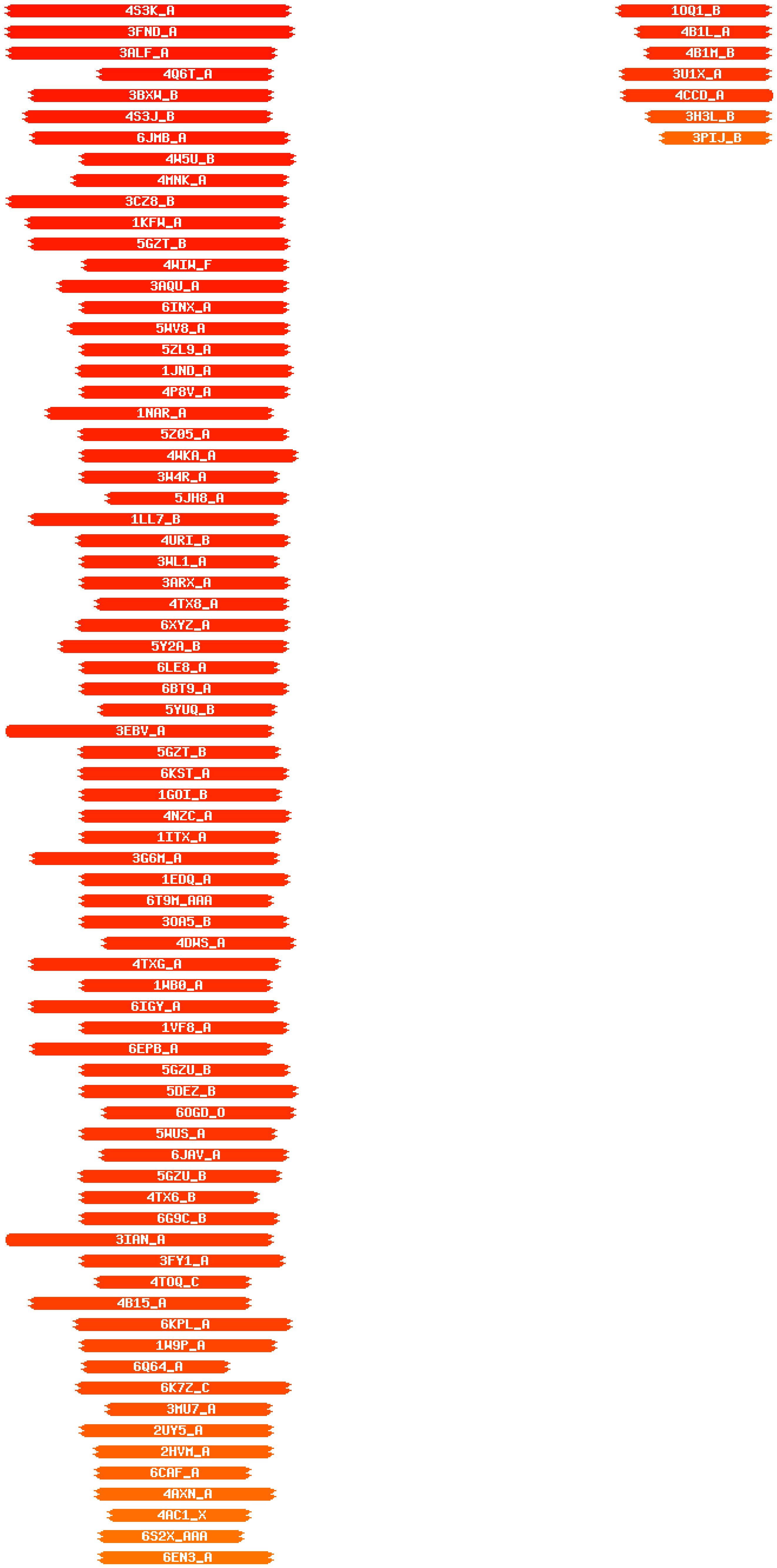

Hitlist

Show 25 Entries

Search:

Nr	Hit	Name	Probability	E-value	Score	SS	Aligned cols	Target Length
1	4S3K_A	Spore germination protein YaaH; TIM Barrel, N-acetylglucosaminidase, hydrolase; HET: SO4; 1.7A {Bacillus megaterium}	98.32	0.000024	71.57	14.3	238	436
2	3FND_A	Chitinase; Chitinase, Tim-barrel, 11092m, Structural Genomics, PSI-2, Protein Structure Initiative, New York SGX Researc	97.97	0.00008	65.43	9.8	238	312
3	3ALF_A	Chitinase, class V; chitinase, HYDROLASE; HET: PO4, EDO; 1.2A {Nicotiana tabacum}	97.95	0.00002	70.04	6	221	353
4	4Q6T_A	Glycosyl hydrolase, family 18; structural genomics, PSI-Biology, Protein Structure Initiative, Midwest Center for Struct	97.95	0.000027	70.3	6.9	150	351
5	3BXW_B	Chitinase domain-containing protein 1; TIM barrel, Lysosome, Secreted, HYDROLASE; HET: SO4; 2.7A {Homo sapiens}	97.86	0.000096	67.84	8.9	204	393
6	4S3J_B	Cortical-lytic enzyme; TIM Barrel, N-acetylglucosaminidase, Spore cortex, HYDROLASE; HET: EDO; 1.6A {Bacillus cereus}	97.83	0.00035	62.81	11.7	199	433
7	6JMB_A	ofchtiv-allosamidin; chitinase, group IV, allosamidin, HYDROLASE; HET: AO3; 1.389A {Ostrinia furnacalis}	97.82	0.00034	64.58	11.7	223	393
8	4W5U_B	Chitinase; TIM barrel, temperature adaptation, hydrolase; HET: MLI; 2.771A {Streptomyces thermoviolaceus}	97.73	0.00011	66.57	6.9	202	408
9	4MNK_A	Chitinase A; Chitinase, Hydrolase, carbohydrate; HET: B3P, NAG; 1.29A {Cycas revoluta}	97.68	0.00023	62.99	8.1	199	348
10	3CZ8_B	Putative sporulation-specific glycosylase ydhD; STRUCTURAL GENOMICS, UNCHARACTERIZED PROTEIN, PROTEIN STRUCTURE INITIATI	97.62	0.0047	53.77	15	229	319
11	1KFW_A	chitinase B; TIM barrel, HYDROLASE; HET: GOL; 1.74A {Arthrobacter sp.} SCOP: c.1.8.5, d.26.3.1	97.6	0.0013	64.81	13	257	435
12	5GZT_B	Chitinase; HYDROLASE; HET: FMT; 2.1A {Paenibacillus sp. FPU-7}	97.57	0.00053	74.19	10.4	247	1136
13	4WIW_F	Glycoside hydrolase family 18; Structural Genomics, PSI-Biology, Midwest Center for Structural Genomics, MCSG, glycoside	97.5	0.0013	59.03	10.4	189	349
14	3AQU_A	At4g19810; stress response, TIM barrel, Hydrolase, chitin; HET: FLC; 2.01A {Arabidopsis thaliana}	97.49	0.00064	59.47	8	214	356
15	6INX_A	N-acetylglucosaminidase; N-acetylglucosaminidase, GH18, hydrolase, STRUCTURAL PROTEIN; 1.429A {Paenibacillus barengoltzi	97.41	0.00068	60.27	7.4	194	345
16	5WV8_A	Chitinase; Ostrinia furnacalis, chitinase, three-dimensional structure, chitin metabolism, HYDROLASE; 2.042A {Ostrinia f	97.34	0.001	65.21	8.4	198	482
17	5ZL9_A	Chitinase AB; Chitinase, engineered protein, HYDROLASE; HET: GOL; 2.6A {Serratia marcescens}	97.23	0.001	67.13	7.1	193	618
18	1JND_A	Imaginal disc growth factor-2; IDGF, imaginal disc, growth factor, chitinase, insulin receptor, heparin, HORMONE-GROWTH	97.21	0.0029	61.27	9.8	196	420
19	4P8V_A	Chitinase-3-like protein 2; Chitinase 3-like protein 2, human YKL-39, Family-18 chitinase, SUGAR BINDING PROTEIN; HET: S	97.21	0.0017	58.46	7.6	184	371
20	1NAR_A	NARBONIN; PLANT SEED PROTEIN; 1.8A {Vicia narbonensis} SCOP: c.1.8.5	97.14	0.0045	56.21	9.7	193	290
21	5Z05_A	Chitinase-3-like protein 1; SIGNALING PROTEIN; HET: NAG; 1.49A {Bubalus bubalis}	97.1	0.0039	55.47	8.7	184	361
22	4WKA_A	Chitotriosidase-1; CHIT1, GH18 chitinase, protonation states, hydrolysis, catalytic mechanism, hydrolase; HET: TLA; 0.95	97.09	0.002	58.03	7	204	377

Nr	Hit	Name	Probability	E-value	Score	SS	Aligned cols	Target Length
23	3W4R_A	Chitinase; insect, glycosyl hydrolase, chitin, HYDROLASE; HET: NAG; 1.7A {Ostrinia furnacalis}	97.07	0.0023	63.35	7.7	180	554
24	5JH8_A	Probable chitinase; HYDROLASE; HET: HIS, 6KY, MLY, MLZ, MSE, EDO; 1.018A {Chromobacterium violaceum}	97.03	0.0031	55.76	7.4	155	317
25	1LL7_B	CHITINASE 1; BETA-ALPHA BARREL, HYDROLASE; 2.0A {Coccidioides immitis} SCOP: c.1.8.5, d.26.3.1	96.96	0.01	54.81	10.4	224	392

Displaying 1 to 25 of 81 hits

« ‹ 1 2 3 4 › »

Alignments

Template alignment | Template 3D Structure | PDBe

1. 4S3K_A Spore germination protein YaaH; TIM Barrel, N-acetylglucosaminidase, hydrolase; HET: SO4; 1.7A {Bacillus megaterium}

Probability: 98.32%, E-value: 0.000024, Score: 71.57, Aligned cols: 238, Identities: 18%, Similarity: 0.332, Template Neff: 11.6

```
Q ss_pred                  cceeeEEEEEEeecC---chHhhHHHhcceeeEEEeeecccCCCccEEEEEecCCCCCCCcccccccCccccCCCCCcc
Q Q_8363653_2        11   KQKRVMTWSLRIQND---DYRHDIERYGNKITSIGFHEFNVDAQGRIAIYRLGPQKDAQGSFTSFTDATTYDRYAPGRTL   87 (999)
Q Consensus          11   kqkrvmtwslriqnd---dyrhdierygnkitsigfhefnvdaqgriaiyrlgpqkdaqgsftsftdattydryapgrtl   87 (999)
                           +.++++.|...-...   .-...+..+...++.|-+.-+.++..|.+.+.                            .
T Consensus         105   ~~~~~~g~~~~~~~~~~~~~~~~~~~~~~~~~~v~~~~~~~~~~~~g~~~~~-----------------------------~  155 (436)
T 4S3K_A            105   RNGEFLGYVETSNRKITPQTEKMINQNAKYLTYLGPANFEVQKDGSLKAP-----------------------------P  155 (436)
T ss_dssp                 EEEEEEEEEECTTCCCCHHHHHHHHHHGGGCSEEEEECEEECTTSCEECC-----------------------------C
T ss_pred                 CCcceeeeEeecCCCCCChhhHHHHHHHHhhhccEEEceeEEECCCCCccCC-----------------------------C
```

```
Q ss_pred                  CcchHhhhhhcCccccceeeEeee-----eCHHHHHHHhccchhhhHHHHhHHHHHHHHhhccCCCCccceeeeEEeccccc
Q Q_8363653_2        88   WPNYIEKDMKRWPHIEYYMQFVI-----FGPETVSRLLDSQTAQDNFINNLKTVVARFRKDQNGNDLGYTGIEIDCEGSF   162 (999)
Q Consensus          88   wpnyiekdmkrwphieyymqfvi-----fgpetvsrlldsqtaqdnfinnlktvvarfrkdqngndlgytgieidcegsf   162 (999)
                           ..+.++. ++. ..+.-.+..-.     +.++..+.++.+...++.|++++..++..+          |+.||+||+|..-
T Consensus         156   ~~~~~~~-~~~-~g~~v~~~i~~~~~~~~~~~~~~~~~~~~~~~~~~~~~~~~~~~~~---------~~dGvdid~e~~~   224 (436)
T 4S3K_A            156   LNNLGSI-AKE-NDVIFLMVLANIENGAFSDEVGRAILNNKDVQDTLLNNIVKTAKEQ---------NFRDIHFDFEFLR   224 (436)
T ss_dssp                 CTTHHHH-HHH-TTCEEEEEEECEETTEECHHHHHHHHTCHHHHHHHHHHHHHHHHHHH---------TEEEEEEECCSCC
T ss_pred                 cccHHHH-HHH-CCCEEEEEEeccCCCCCCHHHHHHHHCCHHHHHHHHHHHHHHHHHHc---------CCCeEEEEccCCC
```

```
Q ss_pred                  cccccccCCCCcchhHcHHHHhcceeeeeCCCCcceEEeehhccCCC-CCchhheecceeeeeecCCCCCccccceeEEEE
Q Q_8363653_2       163   SDSKWDTRAGDDVKYINLLKRIKNEVIIDANPDFKLRINAHAMWGDG-IPDYYRFHNYKLFAESTDKNGNPLLDEVQIMT   241 (999)
Q Consensus         163   sdskwdtragddvkyinllkrikneviidanpdfklrinahamwgdg-ipdyyrfhnyklfaestdkngnplldevqimt   241 (999)
                                   ..+.-.|.++++.++...- ..  ...|.+..-+..... -..++...+++.         -..++|.+.+|+
T Consensus         225   --------~~~~~~~~~~~~~~~~i~~l~~~l~-~~--~~~l~~~~~~~~~~~~~~~~~~~~~~~~--------l~~~~D~v~~~~   285 (436)
T 4S3K_A            225   --------PADKEAYIAFLQKAKKRLQ-DE--QLLMSVALAPKTSRDQKGKWYEAHDYKA--------IGEIANFVVPMT   285 (436)
T ss_dssp                 --------GGGHHHHHHHHHHHHHHHH-HT--TCEEEEEECCCSSTTCCSTTTTTCCHHH--------HHHHSSEEEECC
T ss_pred                 --------HHHHHHHHHHHHHHHHHHHc-cc--CcEEEEEeCCCCCcCCCCCcccccCHHH--------HHHHcCEEEEec
```

```
Q ss_pred                  eecccccCCCCCCCCChHHHhcHHHHHHHHhcCCCCCCCCccceEeeEEeCCCccccCCCCCC--CCCCCceeehhHhccc
Q Q_8363653_2       242   YDFSWSGSAPGPSTPLWWMRNVAEWVKQCFDPSVNPNAKCTIDNVYLGGAGYGRRWPIHS--DDNWGSTVTYRDLVDW   317 (999)
Q Consensus         242   ydfswsgsapgpstplwwmrnvaewvkqcfdpsvnpnakctidnvylggagygrrwpihs--ddnwgstvtyrdlvdw   317 (999)
                           |++.+....+++..|+-|+..+.+|+..-+++          ..+.+|-..||+.|..-.  .......++|.+..+.
T Consensus         286   y~~~~~~~~~~~~~~~~~~~~~~~~~~~~~~~~~----------~ki~~gi~~~g~~~~~~~~~~~~~~~~~~~~~~~~~~~   353 (436)
T 4S3K_A            286   YEWGYSGGPPMAVSPIGPVRDVLEYAVSEIPS----------SKIIMGQNLYGYDWTLPYKPGGEYAKAISPQRAIEL   353 (436)
T ss_dssp                 CC----CCCSCCSSCHHHHHHHHHHHHHHSCG----------GGBEEEEESCEEEEESSCCTTSCCCEEECHHHHHHH
T ss_pred                 ccccCCCCCCCCCCCcHHHHHHHHHHHHhhCCH----------HHeeecccccccccCCCCCCCCccccCHHHHHHH
```

Template alignment | Template 3D Structure | PDBe

2. 3FND_A Chitinase; Chitinase, Tim-barrel, 11092m, Structural Genomics, PSI-2, Protein Structure Initiative, New York SGX Research Center for Structural; HET: MSE; 1.9A {Bacteroides thetaiotaomicron}

Probability: 97.97%, E-value: 0.00008, Score: 65.43, Aligned cols: 238, Identities: 12%, Similarity: 0.094, Template Neff: 11.5

```
Q ss_pred                  cceeeEEEEEEeecCchHhhHHHhcceeeEEEeeecccCCCccEEEEEecCCCCCCCcccccccCccccCCCCCccCcc
Q Q_8363653_2        11   KQKRVMTWSLRIQNDDYRHDIERYGNKITSIGFHEFNVDAQGRIAIYRLGPQKDAQGSFTSFTDATTYDRYAPGRTLWPN   90 (999)
Q Consensus          11   kqkrvmtwslriqnddyrhdierygnkitsigfhefnvdaqgriaiyrlgpqkdaqgsftsftdattydryapgrtlwpn   90 (999)
                           +.+++++|+..+.........-......+++|.+.++.++.+|.+...                           +......+
T Consensus           2   ~~~~v~gy~~~~~~~~~~~~~~~~~~~~~~~t~i~~~~~~~~~~~~~~~~~~~~-------------------------~~~~~~~~   56 (312)
T 3FND_A              2   SLKVVIGYLALDDWEFESLFPTIEWKYLTHINASFARVKADGTLNIN-------------------------PVRKRIES   56 (312)
T ss_dssp                 CCCEEEEEEEETTCTTHHHHGGGCCGGGCSEEEEEEEEECTTSCEECT-------------------------TTTTTHHH
T ss_pred                 CcceEEEeecCCcccccccCCCCCHHHCCEEEEEceEECCCCcEeeC-------------------------chHHhHHH
```

```
Q ss_pred                  hHhhhhhcCccccceeeEeeeeCHHHHHHHhccchhhhHHHHhHHHHHHHHhhccCCCCccceeeeEEecccccccccccC
Q Q_8363653_2        91   YIEKDMKRWPHIEYYMQFVIFGPETVSRLLDSQTAQDNFINNLKTVVARFRKDQNGNDLGYTGIEIDCEGSFSDSKWDTR   170 (999)
Q Consensus          91   yiekdmkrwphieyymqfvifgpetvsrlldsqtaqdnfinnlktvvarfrkdqngndlgytgieidcegsfsdskwdtr   170 (999)
                           +++. +++ ++++++++++++++....+..++.+++.++.|++++.++++++         ++|||+||+|.....
T Consensus          57   ~~~~-~~~-~~~~~~~~~~i~~~~~~~~~~~~~~~~~~~~~~~~~~~~~~~~~~---------~~dGi~id~e~~~~~~------   119 (312)
T 3FND_A             57   VRET-AHK-HNVKILISLAKNSPGEFTTAINDPKARKELIQQIIAFTKEY---------KLDGFDIDYEEYDNW------   119 (312)
```

```
T ss_dssp                    HHHH-HHH-TTCEEEEEEEEESSTTHHHHHHHHSHHHHHHHHHHHHHHHHHHT---------TCSEEEECCCCCTTH------
T ss_pred                    HHHH-HHH-CCCEEEEEecCCCCcchHHHhcCHHHHHHHHHHHHHHHHHh---------CCCcEEeCCCCCCch------


Q ss_pred                     CCCcchhHcHHHHhcceeeeCCCCcceEEeehhccCCCCCchhheecceeeeecCCCCCccccceeEEEEeecccCCC
Q Q_8363653_2       171     AGDDVKYINLLKRIKNEVIIDANPDFKLRINAHAMWGDGIPDYYRFHNYKLFAESTDKNGNPLLDEVQIMTYDFSWSGSA   250 (999)
Q Consensus         171     agddvkyinllkrikneviidanpdfklrinahamwgdgipdyyrfhnyklfaestdkngnplldevqimtydfswsgsa   250 (999)
                              ..+...+..+++++.+...  .  ++.|++.+++.....         ...        +.+.+|+|.+|+|++......
T Consensus         120     ~~~~~~~~~~l~~~~~~~~~--~--~~~l~~~~~~~~~~~---------~~~--------~~~~~D~v~i~~y~~~~~~~~   178 (312)
T 3FND_A            120     DKNFPSLLVFARGLYLAKE--K--NMLMTCAVNSRWLNY---------GTE--------WEQYFDYINLMSYDRGAFTDK   178 (312)
T ss_dssp                    HHHHHHHHHHHHHHHHHHSC--T--TCEEEEEECCSSSCC---------TTT--------SGGGCSEEEECCCCTTCSSSS
T ss_pred                    hhchHHHHHHHHHHHHHhcc--c--CceEEEEeecchhcc---------Cch--------HHHhCCEEEEeCCCCCCCCC


Q ss_pred                     CCCCCChHHHhcHHHHHHH--hcCCCCCCCCcceEeeEEeCCCccccCCCCCC--CCCCCceeehhHhccccccE
Q Q_8363653_2       251     PGPSTPLWWMRNVAEWVKQ--CFDPSVNPNAKCTIDNVYLGGAGYGRRWPIHS--DDNWGSTVTYRDLVDWQNGY   321 (999)
Q Consensus         251     pgpstplwwmrnvaewvkq--cfdpsvnpnakctidnvylggagygrrwpihs--ddnwgstvtyrdlvdwqngy   321 (999)
                              +++..+..++....+++..  ++++          +||++|+++||..|....  ........+|.++++..+..
T Consensus         179     ~~~~~~~~~~~~~~~~~~~~~g~~~----------~ki~~gi~~~g~~~~~~~~~~~~~~~~~~~~~~~~~~~~~   243 (312)
T 3FND_A            179     PVQHASYDDFVKDLKYWNEQCRASK----------SKIVGGLPFYGYSWEESLQGAVDDVRGIRYSGILKHLGNE   243 (312)
T ss_dssp                    CCCSSCHHHHHHHHHCCCCCSCCCG----------GGEEEEEESEEEECCGGGTTSSCTTSEEEHHHHHHHHCGG
T ss_pred                    CCCCCCHHHHHHHHHHHHHHhcCCCH----------HHEEEeecccccccccccccccCcchhhHHHHhcCc
```

Template alignment | Template 3D Structure | PDBe

3. 3ALF_A Chitinase, class V; chitinase, HYDROLASE; HET: PO4, EDO; 1.2A {Nicotiana tabacum}

Probability: 97.95%, E-value: 0.00002, Score: 70.04, Aligned cols: 221, Identities: 15%, Similarity: 0.155, Template Neff: 11.6

```
Q ss_pred                     ceeeEEEEEEeecCchHhhHHHhcceeeEEEeeecccCCCCccEEEEEecCCCCCCCccccccCccccCCCCCCccCcch
Q Q_8363653_2        12     QKRVMTWSLRIQNDDYRHDIERYGNKITSIGFHEFNVDAQGRIAIYRLGPQKDAQGSFTSFTDATTYDRYAPGRTLWPNY   91 (999)
Q Consensus          12     qkrvmtwslriqnddyrhdierygnkitsigfhefnvdaqgriaiyrlgpqkdaqgsftsftdattydryapgrtlwpny   91 (999)
                              .++++||+..  |... ....+....|+||.+.+..++.++.......                       .....+..+
T Consensus           2     ~~~~~gy~~~--~~~~-~~~~~~~~~~~t~i~~~~~~~~~~~~~~~~~~-----------------------~~~~~~~~~   55 (353)
T 3ALF_A              2     QNVKGGYWFK--DSGL-ALNNIDSTLFTHLFCAFADLNPQLNQLIISP-----------------------ENQDSFRQF   55 (353)
T ss_dssp                    CCEEEEEEEG--GGCC-CGGGCCGGGCSEEEEEEEEEETTTTEEECCH-----------------------HHHHHHHHH
T ss_pred                    CCccceeecc--CCCC-CHHHCChhhccEEEEEeeEecCcccCccceeCC-----------------------CchhHHHHH


Q ss_pred                     Hhhhh--hcCccccceeeEeeee--CHHHHHHHhccchhhhHHHHhHHHHHHHHhhccCCCCccceeeeEEecccccccc
Q Q_8363653_2        92     IEKDM--KRWPHIEYYMQFVIF--GPETVSRLLDSQTAQDNFINNLKTVVARFRKDQNGNDLGYTGIEIDCEGSFSDSKW   167 (999)
Q Consensus          92     iekdm--krwphieyymqfvif--gpetvsrlldsqtaqdnfinnlktvvarfrkdqngndlgytgieidcegsfsdskw   167 (999)
                              ++. +  +. ++++++++++++  ....+...+.++..++.+++++.++++++         +||||+||+|.+..
T Consensus          56     ~~~-lk~~~-~~~~v~lsigg~~~~~~~~~~~~~~~~~~~~~~~~~i~~~~~~~~---------~~dGidld~e~~~~----   120 (353)
T 3ALF_A             56     TST-VQRKN-PSVKTFLSIAGGRANSTAYGIMARQPNSRKSFIDSSIRLARQL---------GFHGLDLDWEYPLS----   120 (353)
T ss_dssp                    HHH-HHHHC-TTCEEEEEEECTTSCHHHHHHHHHSHHHHHHHHHHHHHHHHHHH---------TCSEEEEECCCCS----
T ss_pred                    HHH-HHHHC-CCCCEEEEEccCCCcCChhHHHHHhcCHHHHHHHHHHHHHHHHHHHh---------CCCcEEEeecCCCC----


Q ss_pred                     ccCCCCcchhHcHHHHhcceeee------eCCCCcceEEeehhccCCCCCchhheecceeeeecCCCCCccccceeEEEE
Q Q_8363653_2       168     DTRAGDDVKYINLLKRIKNEVII------DANPDFKLRINAHAMWGDGIPDYYRFHNYKLFAESTDKNGNPLLDEVQIMT   241 (999)
Q Consensus         168     dtragddvkyinllkrikneviii------danpdfklrinahamwgdgipdyyrfhnyklfaestdkngnplldevqimt   241 (999)
                                 ......+..++++++.....      ..  .+.+++.++......    ...++...        +...+|++.+|+
T Consensus         121     ---~~~~~~~~~~~~l~~l~~~l~~~~~~~~~~~~--~~~l~~~~~~~~~~~~----~~~~~~~~~--------~~~~~D~~~i~~   183 (353)
T 3ALF_A            121     ---AADMTNLGTLLNEWRTAINTEARNSGRA--ALLLTAAVSNSPRVN----GLNYPVES--------LARNLDWINLMA   183 (353)
T ss_dssp                    ---HHHHHHHHHHHHHHHHHHHHHHHHHHHCSC--CCEEEEEEESSSEET----TEECCHHH--------HHHHCSEEEEEC
T ss_pred                    ---HHHHhcHHHHHHHHHHHHHHHHHHHHHcCCc--eEEEEEEcCCChhhc----cCCCCHHH--------HHHHCCEEEEec


Q ss_pred                     eeccccCCCCC----------CCCChHHHhcHHHHHHH-hcCCCCCCCCcceEeeEEeCCCccccCCCCCC
Q Q_8363653_2       242     YDFSWSGSAPG----------PSTPLWWMRNVAEWVKQ-CFDPSVNPNAKCTIDNVYLGGAGYGRRWPIHS   301 (999)
Q Consensus         242     ydfswsgsapg----------pstplwwmrnvaewvkq-cfdpsvnpnakctidnvylggagygrrwpihs   301 (999)
                              |++......+.          +.....+ ...+..+.. ++++          +||++|+++||..|....
T Consensus         184     y~~~~~~~~~~~~~~~~~~~~~~~~~-~~~~~~~~~~~~~~~~----------~ki~~g~~~~g~~~~~~~~   243 (353)
T 3ALF_A            184     YDFYGPNWSPSQTNSHAQLFDPVNHVSG-SDGINAWIQAGVPT----------KKLVLGIPFYGYAWRLVN   243 (353)
T ss_dssp                    CCSSCTTTSTTBCCCSSCSCCTTTCCSH-HHHHHHHHHTTCCG----------GGEEEEEESEEEEEEESC
T ss_pred                    ccccCCCCCCCcCCCCchhcCCccCCCh-HHHHHHHHHcCCCH----------HHeEEEeeeceEEEEecC
```

Template alignment | Template 3D Structure | PDBe

4. 4Q6T_A Glycosyl hydrolase, family 18; structural genomics, PSI-Biology, Protein Structure Initiative, Midwest Center for Structural Genomics, MCSG, HYDROLASE; HET: MSE; 1.4A {Pseudomonas protegens}

Probability: 97.95%, E-value: 0.000027, Score: 70.3, Aligned cols: 150, Identities: 23%, Similarity: 0.425, Template Neff: 11.2

```
Q ss_pred                     eCHHHHHHHhccchhhhHHHHhHHHHHHHHhhccCCCCccceeeeEEeccccccccccCCCCcchhHcHHHHhcceeee
Q Q_8363653_2       111     FGPETVSRLLDSQTAQDNFINNLKTVVARFRKDQNGNDLGYTGIEIDCEGSFSDSKWDTRAGDDVKYINLLKRIKNEVII   190 (999)
Q Consensus         111     fgpetvsrlldsqtaqdnfinnlktvvarfrkdqngndlgytgieidcegsfsdskwdtragddvkyinllkrikneviii   190 (999)
                              +.++..+.++.+.+..++|++++..++..+         |+.||+||.|..        ...+...+..+++.++...-
T Consensus          83     ~~~~~~~~~~~~~~~~~~~~~~~i~~~~~~~~---------~~DGidid~e~~--------~~~~~~~~~~~~~i~~l~~~l~-   144 (351)
T 4Q6T_A             83     FDPAISHSILNDRALSAGTVKQLVKLAKEG---------GFAGINLDFEKV--------EPRNRAAFCAFVKTLGNALH-   144 (351)
T ss_dssp                    ECHHHHHHHTCHHHHHHHHHHHHHHHHHH---------TCSEEEEECCCC--------CGGGHHHHHHHHHHHHHHHHH-
T ss_pred                    CCHHHHHHHhcCHHHHHHHHHHHHHHHHHHc---------CCCeEEEEcCCC--------CHHHHHHHHHHHHHHHHHHHHH-


Q ss_pred                     eCCCCcceEEeehhccCCCCCchhheecceeeeecCCCCCccccceeEEEEeecccCCC---------CCCCCChHHHh
Q Q_8363653_2       191     DANPDFKLRINAHAMWGDGIPDYYRFHNYKLFAESTDKNGNPLLDEVQIMTYDFSWSGSA---------PGPSTPLWWMR   261 (999)
Q Consensus         191     danpdfklrinahamwgdgipdyyrfhnyklfaestdkngnplldevqimtydfswsgsa---------pgpstplwwmr   261 (999)
                                ....++.+..........++....+++..++         .+|.+.+|+|++......         +++.++.-|.+
T Consensus         145     --~~~~~~~i~~~~~~~~~~~~~~~~~~~~~~~~~--------~~d~~~~~~y~~~~~~~~~~~~~~~~~~~~~~~~~~~~~   214 (351)
```

```
T 4Q6T_A         145    --ASNKKLIISIPPKLSDTEPEYLQGYDYKALGA--------AVDYFQVMTYDQVGPGWSSGGFHNEAWPGPESGFDWQQ   214 (351)
T ss_dssp               --HTTCEEEEEECCCSSSSSSGGGTTCCHHHHHH--------HCSEEEECCCCSEETTEECTTCGGGEESCCSSCHHHHH
T ss_pred               --hcCCEEEEEeCCCCCCCChhhccCCHHHHHH--------HCCEEEEEcCCccCCCcccCCCCCCCCCCCCCCChHHHH


Q ss_pred               cHHHHHHHhcCCCCCCCCcceEeeEEeCCCcccccCCC
Q Q_8363653_2     262    NVAEWVKQCFDPSVNPNAKCTIDNVYLGGAGYGRRWP   298 (999)
Q Consensus       262    nvaewvkqcfdpsvnpnakctidnvylggagygrrwp   298 (999)
                        .+.+|+.+.+.+          ..+.+|-..||+.|.
T Consensus       215    ~~~~~~~~~~~~----------~kl~igl~~yg~~~~   241 (351)
T 4Q6T_A         215    ALLSYAVSRVPA----------SKVLAGLPTYGQDYS   241 (351)
T ss_dssp               HHHHHHHHHSCG----------GGEEEEEESEEEETT
T ss_pred               HHHHHHHhcCCH----------HHeEEEeecceeecc
```

Template alignment | Template 3D Structure | PDBe

5. 3BXW_B Chitinase domain-containing protein 1; TIM barrel, Lysosome, Secreted, HYDROLASE; HET: SO4; 2.7A {Homo sapiens}

Probability: 97.86%, E-value: 0.000096, Score: 67.84, Aligned cols: 204, Identities: 19%, Similarity: 0.29, Template Neff: 11.2

```
Q ss_pred               ceeeEEEeeecccCCCccEEEEEeCCCCCCCcccccccCccccCCCCCccCcchHhhhhhcCccccee--eEeeeeCH
Q Q_8363653_2      36    NKITSIGFHEFNVDAQGRIAIYRLGPQKDAQGSFTSFTDATTYDRYAPGRTLWPNYIEKDMKRWPHIEYY--MQFVIFGP   113 (999)
Q Consensus        36    nkitsigfhefnvdaqgriaiyrlgpqkdaqgsftsftdattydryapgrtlwpnyiekdmkrwphieyy--mqfvifgp   113 (999)
                        +.++.|-+.-+.++..|.+.....+......                        +.+..-.++.+++.-.  +.+--++.
T Consensus       101    ~~~t~i~~~~~~~~~~~~~~~~~~~~~~~~~-----------------------~~~~~~~~~~~~~kv~~~l~~gg~~~   157 (393)
T 3BXW_B         101    SKFTQISPVWLQLKRRGREMFEVTGLHDVDQ-----------------------GWMRAVRKHAKGLHIVPRLLFEDWTY   157 (393)
T ss_dssp               GGCSEEEECCEEEEEEETTEEEEECGGGCCH-----------------------HHHHHHHHHSSSCEECCEEEECSCCH
T ss_pred               hhCCEEeeEEEEEeeCCceEEEEcccccCCH-----------------------HHHHHHHHHcCCCcEEEEEEecCCCH


Q ss_pred               HHHHHHhccchhhhHHHHhHHHHHHHHhhccCCCCccceeeeEEe-ccccccccccCCCCcchhHcHHHHhcceeeeeC
Q Q_8363653_2     114    ETVSRLLDSQTAQDNFINNLKTVVARFRKDQNGNDLGYTGIEID-CEGSFSDSKWDTRAGDDVKYINLLKRIKNEVIIDA   192 (999)
Q Consensus       114    etvsrlldsqtaqdnfinnlktvvarfrkdqngndlgytgieid-cegsfsdskwdtragddvkyinllkrikneviida   192 (999)
                        .....++.+.+..++|++++...+...          |+.||.|| -|..         ...+.-.+..+++.++.+.--.
T Consensus       158    ~~~~~~~~~~~~~~~~~~~~~~~~~~~---------~~dGidid~~e~~--------~~~~~~~~~~~~~~~l~~~l~~~-   219 (393)
T 3BXW_B         158    DDFRNVLDSEDEIEELSKTVVQVAKNQ---------HFDGFVVEVWNQL--------LSQKRVGLIHMLTHLAEALHQA-   219 (393)
T ss_dssp               HHHHHHHTCHHHHHHHHHHHHHHHHHH---------TCCEEEEECGGGC--------CS-CHHHHHHHHHHHHHHHHHT-
T ss_pred               HHHHHHhCCHHHHHHHHHHHHHHHHHC---------CCCEEEEEeHHHh--------cHHhHHHHHHHHHHHHHHHHHC-


Q ss_pred               CCCcceEEeehhccCCCCCchhheecceeeeeecCCCCCcccceeEEEEeeccccCCCCCCCCChHHHhcHHHHHHHHhcC
Q Q_8363653_2     193    NPDFKLRINAHAMWGDGIPDYYRFHNYKLFAESTDKNGNPLLDEVQIMTYDFSWSGSAPGPSTPLWWMRNVAEWVKQCFD   272 (999)
Q Consensus       193    npdfklrinahamwgdgipdyyrfhnyklfaestdkngnplldevqimtydfswsgsapgpstplwwmrnvaewvkqcfd   272 (999)
                        +....+-+.+....+......+...++.-.+..        +|.+.+|+||+. .+..++|..|+.|+....++...
T Consensus       220    ~~~l~~~~~~~~~~~~~~~~~~~~~~~~~~~~~--------~D~i~~~~yd~~-~~~~~~~~~~~~~~~~~~~~~~~~---   287 (393)
T 3BXW_B         220    RLLALLVIPPAITPGTDQLGMFTHKEFEQLAPV--------LDGFSLMTYDYS-TAHQPGPNAPLSWVRACVQVLDP---   287 (393)
T ss_dssp               TCEEEEEECCSBCTTTSSBCSSCHHHHHHHTTT--------CSEEEECCCCC-BTTBCCCSSCHHHHHHHHHHHST---
T ss_pred               CCEEEEEeCCCCCCCCCCCCCCCCHHHHHHHHHH--------CCEEEEecCCCC-CCCCCCCCCCHHHHHHHHHHHCc---


Q ss_pred               CCCCCCCcceEeeEEeCCCcccccCCC
Q Q_8363653_2     273    PSVNPNAKCTIDNVYLGGAGYGRRWP   298 (999)
Q Consensus       273    psvnpnakctidnvylggagygrrwp   298 (999)
                              ..+.-+.+.+|-..||+.|+
T Consensus       288    ------~g~~~~ki~~gl~~~g~~~~   307 (393)
T 3BXW_B         288    ------KSKWRSKILLGLNFYGMDYA   307 (393)
T ss_dssp               ------TCSSGGGEEEEEESSEEEEE
T ss_pred               ------CCCcHHhEEEEeecccccc
```

Template alignment | Template 3D Structure | PDBe

6. 4S3J_B Cortical-lytic enzyme; TIM Barrel, N-acetylglucosaminidase, Spore cortex, HYDROLASE; HET: EDO; 1.6A {Bacillus cereus}

Probability: 97.83%, E-value: 0.00035, Score: 62.81, Aligned cols: 199, Identities: 20%, Similarity: 0.377, Template Neff: 12.1

```
Q ss_pred               hHHHhcceeeEEEeeecccCCCccEEEEEeCCCCCCCcccccccCccccCCCCCccCcchHhhhhhcCcccceeeEee
Q Q_8363653_2      30    DIERYGNKITSIGFHEFNVDAQGRIAIYRLGPQKDAQGSFTSFTDATTYDRYAPGRTLWPNYIEKDMKRWPHIEYYMQFV   109 (999)
Q Consensus        30    dierygnkitsigfhefnvdaqgriaiyrlgpqkdaqgsftsftdattydryapgrtlwpnyiekdmkrwphieyymqfv   109 (999)
                        .++.....++.|-+.-+.++++|.+..-.                             ...-.....+..++...+.+.
T Consensus       125    ~~~~~~~~~t~~~~~~~~~~~~g~~~~~~------------------------------~~~~~~~~~~~~~~~~~~~~~~~i~   174 (433)
T 4S3J_B         125    ATRAINPFLTYLAYFSFEAKRDGTLKEPT------------------------------ETAKIANIATQGQTIPMLVIT   174 (433)
T ss_dssp               HHHHHGGGCSEEEEEEEEECTTSCEECCT------------------------------THHHHHHHHHTTCEEEEEEE
T ss_pred               HHHHHhhcCCEEEeEEEEeCCCCCcCCCC------------------------------CcHHHHHHHHhcCceeEEEEE


Q ss_pred               ee-----CHHHHHHHhccchhhhHHHHhHHHHHHHHhhccCCCCccceeeeEEecccccccccccCCCCcchhHcHHHHh
Q Q_8363653_2     110    IF-----GPETVSRLLDSQTAQDNFINNLKTVVARFRKDQNGNDLGYTGIEIDCEGSFSDSKWDTRAGDDVKYINLLKRI   184 (999)
Q Consensus       110    if-----gpetvsrlldsqtaqdnfinnlktvvarfrkdqngndlgytgieidcegsfsdskwdtragddvkyinllkri   184 (999)
                        .+      ..+....++.+.....+.|++++...+..+          |+.||.||.|..-         ..+...+..+++.+
T Consensus       175    ~~~~~~~~~~~~~~~~~~~~~~~~~~~~~~~~~~---------~~dGid~d~e~~~--------~~~~~~~~~~~~~~~l   237 (433)
T 4S3J_B         175    NIENGNFSADLTSVILRDATIQNKFITNILQTAEKY---------GMRDIHFDFESVA--------PEDREAYNRFLRNV   237 (433)
T ss_dssp               CEETTEECHHHHHHHHHCHHHHHHHHHHHHHHHHHH---------TCCEEEEECCSCC--------GGGHHHHHHHHHHH
T ss_pred               eccCCCCCHHHHHHHhcCHHHHHHHHHHHHHHHHHc---------CCCEEEEECCCCC--------HHHHHHHHHHHHHH


Q ss_pred               cceeeeeCCCCcceEEeehhc-cCCCCChhheecceeeeeecCCCCCcccceeEEEEeeccccCCCCCCCCChHHHhcH
Q Q_8363653_2     185    KNEVIIDANPDFKLRINAHAM-WGDGIPDYYRFHNYKLFAESTDKNGNPLLDEVQIMTYDFSWSGSAPGPSTPLWWMRNV   263 (999)
Q Consensus       185    kneviidanpdfklrinaham-wgdgipdyyrfhnyklfaestdkngnplldevqimtydfswsgsapgpstplwwmrnv   263 (999)
                        +.+.    .+.+.+-+...+. ++.....++...++.-.+        ..+|.+.+|+|++.+..+.+++..|.-|+..+
T Consensus       238    ~~~l----~~~~~~~~~~~~~~~~~~~~~~~~~~~~~~~--------~~vd~~~~~~y~~~~~~~~~~~~~~~~~~~~~~~   305 (433)
```

```
T 4S3J_B          238    KIRL----PSGYTLSTTLVPKTSSNQKGKFFEAHDYKAQG--------QIVDFVVIMTYDWGWQGGPPMAISPIGPVKEV   305 (433)
T ss_dssp                HHTC----CTTCEEEEEECCCSCTTCCSTTTTTCCHHHHH--------HHCSEEEECCCCSSCTTSCSSCSSCHHHHHHH
T ss_pred                HHHC----CCCCEEEEEeCCCCCCCCCCCceeccCHHHHH--------HHCCEEEEecccCccCCCCCCCCCChHHHHHH
```

```
Q ss_pred                HHHHHHhcCCCCCCCCcceEeeEEeCCCccccCC
Q Q_8363653_2      264    AEWVKQCFDPSVNPNAKCTIDNVYLGGAGYGRRW   297 (999)
Q Consensus       264    aewvkqcfdpsvnpnakctidnvylggagygrrw   297 (999)
                         ..|+.+-..|+          .+.+|-.-||+.|
T Consensus       306    ~~~~~~~~~~~----------ki~~g~~~~g~~~   329 (433)
T 4S3J_B          306    LQYAKSQMPPQ----------KIMMGQNLYGFDW   329 (433)
T ss_dssp                HHHHHHHSCGG----------GEEEEEECCEEEE
T ss_pred                HHHHHhhCCHH----------HEEEeceeceecc
```

Template alignment | Template 3D Structure | PDBe

7. 6JMB_A ofchtiv-allosamidin; chitinase, group IV, allosamidin, HYDROLASE; HET: AO3; 1.389A {Ostrinia furnacalis}

Probability: 97.82%, E-value: 0.00034, Score: 64.58, Aligned cols: 223, Identities: 17%, Similarity: 0.251, Template Neff: 11.1

```
Q ss_pred                eeEEEeeecccCCCccEEEEEecCCCCCCCcccccccCcccccCCCCCccCcchHhhhhhcCccccceeeEeeee--CHHH
Q Q_8363653_2       38    ITSIGFHEFNVDAQGRIAIYRLGPQKDAQGSFTSFTDATTYDRYAPGRTLWPNYIEKDMKRWPHIEYYMQFVIF--GPET   115 (999)
Q Consensus        38    itsigfhefnvdaqgriaiyrlgpqkdaqgsftsftdattydryapgrtlwpnyiekdmkrwphieyymqfvif--gpet   115 (999)
                         +|.|-+.-+.++..|.+.                     +.+.......-+..-+.+-.++.|+++.+..+--.  +...
T Consensus        33    ~t~i~~~~~~~~~~~~~~---------------------~~~~~~~~~~~~~~~~~~~~~~~~~~kv~~svg~~~~~~~~   91 (393)
T 6JMB_A           33    CTHLVYAFIGINAEGTAL---------------------ALDPELDVERGNFKQFTSLKEKNPNLKTLVAVGGWSEGSAQ   91 (393)
T ss_dssp                CSEEEEEEEEECTTCCEE---------------------ESSHCCCCCTCHHHHHHGGGGTCTTCEEEEEEECGGGCSHH
T ss_pred                CcEEEEEEeEECCCCCee---------------------cCChhcchhcCchHHHHHHHHHCCCCeEEEEeCCCcccCChh
```

```
Q ss_pred                HHHHhccchhhhHHHHhHHHHHHHHhhccCCCCccceeeeEEeccccccccccCCCCCcchhHcHHHHhcceeeeeCCCC
Q Q_8363653_2      116    VSRLLDSQTAQDNFINNLKTVVARFRKDQNGNDLGYTGIEIDCEGSFSDSKWDTRAGDDVKYINLLKRIKNEVIIDANPD   195 (999)
Q Consensus       116    vsrlldsqtaqdnfinnlktvvarfrkdqngndlgytgieidcegsfsdskwdtragddvkyinllkrikneviidanpd   195 (999)
                         .+.++.+...+++|++++..++.++         ++.||.||.|..-.... .....+..+++.++++++++.- ..+-.
T Consensus        92    ~~~~~~~~~~~~~~~i~~~~~~~~~---------~~dGi~ld~e~~~~~~~~-~~~~~~~~~~~~~~~~l~~~l~-~~~~~   160 (393)
T 6JMB_A           92    YSIMAAEPEYRQNFIQTSLAMILEY---------NFDGLDVDWEYPNRRDT-VHGEDDIEQFSTLLKELREEFD-NYGLL   160 (393)
T ss_dssp                HHHHHHCHHHHHHHHHHHHHHHHHH---------TCCEEEEECSCTTSTTC-SSTTHHHHHHHHHHHHHHHHHH-TTTCE
T ss_pred                HHHHhcCHHHHHHHHHHHHHHHHHh---------CCCcEEecccCCCCCCC-CCChHHHHHHHHHHHHHHHHhc-ccCcE
```

```
Q ss_pred                cceEEeehhccCCCCCchhheecceeeeeecCCCCCcccceeEEEEeecccc-CCCCCCCCCh--------------HHH
Q Q_8363653_2      196    FKLRINAHAMWGDGIPDYYRFHNYKLFAESTDKNGNPLLDEVQIMTYDFSWS-GSAPGPSTPL--------------WWM   260 (999)
Q Consensus       196    fklrinahamwgdgipdyyrfhnyklfaestdkngnplldevqimtydfsws-gsapgpstpl--------------wwm   260 (999)
                         +.+.+..........++-....+              +|-+.+|+||+.+. ...+++..|+              +|+
T Consensus       161    ~~~~~~~~~~~~~~~~~~l~~~--------------vD~~~i~~yd~~~~~~~~~~~~~l~~~~~~~~~~~~~~~~~~~   226 (393)
T 6JMB_A          161    LTVAVSAVEEAAVQSYDVPSVAKY--------------VDYIGVMTYDMHGAWDSVTGHNAPLFISEGESAEQESTLYNV   226 (393)
T ss_dssp                EEEEECCSHHHHHHHCCHHHHHHH--------------SSEEEECCCCSSCTTSSBCCCSSCSSCCTTCCSTTGGGSCCH
T ss_pred                EEEEecCChHHHhhcCCHHHHHHH--------------CCEEEeecccCCCCccccCCcCCCCCCCCCCCchhhhhccccH
```

```
Q ss_pred                hcHHHHHHHh-cCCCCCCCCcceEeeEEeCCCccccCCCCCCCCCCC------------------ceeehhHhcc
Q Q_8363653_2      261    RNVAEWVKQC-FDPSVNPNAKCTIDNVYLGGAGYGRRWPIHSDDNWG------------------STVTYRDLVD   316 (999)
Q Consensus       261    rnvaewvkqc-fdpsvnpnakctidnvylggagygrrwpihsddnwg------------------stvtyrdlvd   316 (999)
                         ....+++... +++          +.+.+|-.-||+.|...+...-.                  ..++|+++.+
T Consensus       227    ~~~~~~~~~~~~~~----------~Ki~~gi~~~g~~~~~~~~~~~~~~~~~~~~~~~~~~~~~~~~~~~~~~~~~~~   291 (393)
T 6JMB_A          227    NNAVQYWLSAGCPP----------EKLVMGVPFYGRTFQLSDPSVNAPNSPSNGAGLAGPYTAESGYVGYNEFCY   291 (393)
T ss_dssp                HHHHHHHHHTTCCG----------GGEEEEEESEEEEEEESSTTCCSTTCBEEEECCCCTTTCCTTEEEHHHHHH
T ss_pred                HHHHHHHHHcCCCH----------HHeEEeeecCeeEEEcCCCCCCCCCCCCCCCCCCCCCcccCCceeeHHHHHH
```

Template alignment | Template 3D Structure | PDBe

8. 4W5U_B Chitinase; TIM barrel, temperature adaptation, hydrolase; HET: MLI; 2.771A {Streptomyces thermoviolaceus}

Probability: 97.73%, E-value: 0.00011, Score: 66.57, Aligned cols: 202, Identities: 22%, Similarity: 0.272, Template Neff: 11.7

```
Q ss_pred                HhhhhhcCccccceeeEeeeeCHHHHHHHhccchhhhHHHHhHHHHHHHHhhccCCCCccceeeeEEeccccccccccCC
Q Q_8363653_2       92    IEKDMKRWPHIEYYMQFVIFGPETVSRLLDSQTAQDNFINNLKTVVARFRKDQNGNDLGYTGIEIDCEGSFSDSKWDTRA   171 (999)
Q Consensus        92    iekdmkrwphieyymqfvifgpetvsrlldsqtaqdnfinnlktvvarfrkdqngndlgytgieidcegsfsdskwdtra   171 (999)
                         +....++-|++...+.  |.|.+..+.+........+|++++...+..+.++.     |+.||+||.| ...+...+...
T Consensus       123    ~~~~~~~~~~~~~~~~--igg~~~~~~~~~~~~~~~~~~~~~~~~~~~~~~-----~~dGidld~e-~~~~~~~~~~~~~   194 (408)
T 4W5U_B          123    LRQLKAKYPHIKVLWS--FGGWTWSGGFADAAKDPQGFAQSCYNLVHDPRWDG-----VFDGIDIDWE-YPNACGLTCDS   194 (408)
T ss_dssp                HHHHHHHSTTCEEEEE--EETTGGGTTHHHHHHSHHHHHHHHHHHHTCTTTTT-----SCSEEEEECS-CTTSBSSSBCC
T ss_pred                HHHHHHHCCCCEEEEE--ECCCcccccchHHHhcCHHHHHHHHHHHhcccccC-----CCCeEEEccc-CCCcCCCCCCC
```

```
Q ss_pred                CCcchhHcHHHHhcceeeeeCCCCcceEEeehhccCCCCCchhheecceeeeeecCCCCCcccceeEEEEeeccccCCCC
Q Q_8363653_2      172    GDDVKYINLLKRIKNEVIIDANPDFKLRINAHAMWGDGIPDYYRFHNYKLFAESTDKNGNPLLDEVQIMTYDFSWSGSAP   251 (999)
Q Consensus       172    gddvkyinllkrikneviidanpdfklrinahamwgdgipdyyrfhnyklfaestdkngnplldevqimtydfswsgsap   251 (999)
                         .+...++.++++++...-   .....+.+.++...+.....+.-            +.=...+|.+.||+||+..+.+.+
T Consensus       195    ~~~~~~~~~l~~l~~~~~---~~~l~~~v~~~~~~~~~~~~~~~------------~~l~~~vD~v~i~~y~~~~~~~~~~   259 (408)
T 4W5U_B          195    SGPDAFRNLMAALRSTFG---DELVTAAVTADGTPGGKIEATDY------------AGAAQYVDWYNVMTYDFFGAWDAQ   259 (408)
T ss_dssp                SCTTHHHHHHHHHHHHHT---TSEEEEEECCCCSTTSHHHHSCH------------HHHHTTCSEEEECCCCSSCTTSTT
T ss_pred                CCHHHHHHHHHHHHHHHC---chheeEEEecCCCCCccccccCH------------HHHHHHCCEEEeEccccCCCCcCC
```

```
Q ss_pred                CCCCChHHHhc----------HHHHHHHhcCCCCCCCCcceEeeEEeCCCccccCCCCCCCCCCCceeehhHhccccccE
Q Q_8363653_2      252    GPSTPLWWMRN----------VAEWVKQCFDPSVNPNAKCTIDNVYLGGAGYGRRWPIHSDDNWGSTVTYRDLVDWQNGY   321 (999)
Q Consensus       252    gpstplwwmrn----------vaewvkqcfdpsvnpnakctidnvylggagygrrwpihsddnwgstvtyrdlvdwqngy   321 (999)
                         ++..+..|+..          +.+-++...+..+.+      +.+.+|-.-||+.|....+...+..........++.++
T Consensus       260    ~~~~~~~~~~~~~~~~~~~~~~~~~~~~~~~~~~~~g~~~------~kl~~g~~~~g~~~~~~~~~~~~~~~~~~~~~~~~~~   333 (408)
```

```
T 4W5U_B           260     GPTAPHSPLTSYDGIPKQGFTSADAIAAFKAQGVPA------DKLLLGIGFYGRGWTGVTQDAPGGTATGPAAGTWEQGI   333 (408)
T ss_dssp                  CCCCCSSCSSCCTTCSSTTCSHHHHHHHHHHHTCCG------GGEEEEEESEEEEEESCSSSSTTSCCSEECCCSSSTTE
T ss_pred                  CCCCCCCCCCCCCCCCCCCCCHHHHHHHHHHcCCCH------HHEEEeeecceeeeeCCCCCCCCCCCCCCCCCCcccce


Q ss_pred                  E
Q Q_8363653_2      322     L   322 (999)
Q Consensus        322     l   322 (999)
                           .
T Consensus        334     ~   334 (408)
T 4W5U_B           334     E   334 (408)
T ss_dssp                  E
T ss_pred                  e
```

Template alignment | Template 3D Structure | PDBe

9. 4MNK_A Chitinase A; Chitinase, Hydrolase, carbohydrate; HET: B3P, NAG; 1.29A {Cycas revoluta}

Probability: 97.68%, E-value: 0.00023, Score: 62.99, Aligned cols: 199, Identities: 15%, Similarity: 0.262, Template Neff: 11.7

```
Q ss_pred                  CCCCccCcchHhhhhhcCccccceeeEee---eeCHHHHHHHhccchhhhHHHHhHHHHHHHHhccCCCCccceeeeEEec
Q Q_8363653_2       82     APGRTLWPNYIEKDMKRWPHIEYYMQFV---IFGPETVSRLLDSQTAQDNFINNLKTVVARFRKDQNGNDLGYTGIEIDC   158 (999)
Q Consensus         82     apgrtlwpnyiekdmkrwphieyymqfv---ifgpetvsrlldsqtaqdnfinnlktvvarfrkdqngndlgytgieidc   158 (999)
                           ......+...+..-.++-|++..++.+-   ....+..+.++.+....++|+.++..++..+          |+.||+||.
T Consensus         49     ~~~~~~~~~~~~~~~~~~~~~~~~lsi~g~~~~~~~~~~~~~~~~~~~~~~~~i~~~~~~~~---------~~dGi~ld~   119 (348)
T 4MNK_A            49     PGSEKTAEDFTPTVRRLNPSVKTLISIGGGGSEVRDNFAKLNSDASARQRFVKSSIALARRY---------GFHGLDLDY   119 (348)
T ss_dssp                  TTCHHHHHHHHHHHHTTCTTCEEEEEEEEESSHHHHHHHHHHHTCHHHHHHHHHHHHHHHHHHH---------TCSEEEEEE
T ss_pred                  CCCccchhhhhHHHHHHCCCCcEEEEeCCCCcchhHhHHHHHhCCHHHHHHHHHHHHHHHHHHh---------CCCcEEEec


Q ss_pred                  ccccccccccCCCCcchhHcHHHHhcceeeeeCC----CCcceEEeehhccCCCCCchhheecceeeeeecCCCCCccc
Q Q_8363653_2      159     EGSFSDSKWDTRAGDDVKYINLLKRIKNEVIIDAN----PDFKLRINAHAMWGDGIPDYYRFHNYKLFAESTDKNGNPLL   234 (999)
Q Consensus        159     egsfsdskwdtragddvkyinllkrikneviidan----pdfklrinahamwgdgipdyyrfhnyklfaestdkngnpll   234 (999)
                           |..-++       .+...+..+++.++...--...    +.+.+-+............+...++...+..        +
T Consensus        120     e~~~~~-------~~~~~~~~~f~~~l~~~l~~~~~~~~~~~~~~~~s~~~~~~~~~~~~~~~~~~~~~~~~~--------~   184 (348)
T 4MNK_A           120     EYPEPQ-------LEMENFVKLVSELTAAIREEARTSGKPRLLLTEAVYFHQKLFPWEVVTEYPVQFIAAG--------L   184 (348)
T ss_dssp                  SCCCSH-------HHHHHHHHHHHHHHHHHHHHHHHHHHCSCCCEEEEEEESSSEECTTSCSEECCHHHHHHH--------C
T ss_pred                  cCCCCh-------HHHHhHHHHHHHHHHHHHHHHHHHHHcCCCcEEEEEEeecccccCCccccCCCHHHHHHh--------C


Q ss_pred                  ceeEEEEeeccccCCC--------CCCCCChHHHhcHHHHHHHhcCCCCCCCCCcceEeeEEeCCCccccCCCCCCCCCCC
Q Q_8363653_2      235     DEVQIMTYDFSWSGSA--------PGPSTPLWWMRNVAEWVKQCFDPSVNPNAKCTIDNVYLGGAGYGRRWPIHSDDNWG   306 (999)
Q Consensus        235     devqimtydfswsgsa--------pgpstplwwmrnvaewvkqcfdpsvnpnakctidnvylggagygrrwpihsddnwg   306 (999)
                           |-+.+|+|++.+....         .++..+.++...+..|+....+++          .+.+|-.-||+.|+.......+
T Consensus        185     D~~~i~~y~~~~~~~~~~~~~~~~~~~~~~~~~~~~~~~~~~~~~~i~~~----------kl~~g~~~~g~~~~~~~~~~~~~   254 (348)
T 4MNK_A           185     DWVNVMAYDFHGSWENFTGAPAALRDPNSKFTASVGIESFLAAGMPPE----------KLVLGIPLFGRSWLLKNNNEVG   254 (348)
T ss_dssp                  SEEEEECCCSSCTTSSBCCCTTCSCC--CCCSHHHHHHHHHHHTCCGG----------GEEEEEESEEEEEEESCTTCCS
T ss_pred                  CEEEEEeccccCCCCCCCCCCcccCCCCCCCcHHHHHHHHHHCCCCHH----------HeEEeeecCeEEEEccCCCCCC


Q ss_pred                  c------------eeehhHh
Q Q_8363653_2      307     S------------TVTYRDL   314 (999)
Q Consensus        307     s------------tvtyrdl   314 (999)
                           .            .++|+++
T Consensus        255     ~~~~~~~~~~~~~~~~~~~e~   274 (348)
T 4MNK_A           255     IGAPAVGAGPVDGALSFSEI   274 (348)
T ss_dssp                  TTCBEEEECTTTTEEEHHHH
T ss_pred                  CCCCCcCCCCCCCcccHHHH
```

Template alignment | Template 3D Structure | PDBe

10. 3CZ8_B Putative sporulation-specific glycosylase ydhD; STRUCTURAL GENOMICS, UNCHARACTERIZED PROTEIN, PROTEIN STRUCTURE INITIATIVE, PSI-2, New York SGX Research Center for Structural Genomics; HET: GOL; 2.2A {Bacillus subtilis subsp. subtilis str. 168}

Probability: 97.62%, E-value: 0.0047, Score: 53.77, Aligned cols: 229, Identities: 23%, Similarity: 0.349, Template Neff: 11.9

```
Q ss_pred                  ceeeEEEEEEeecCchHhhHHHhcceeeEEEeeecccCCCccEEEEEecCCCCCCCcccccccCcccccCCCCCccCcch
Q Q_8363653_2       12     QKRVMTWSLRIQNDDYRHDIERYGNKITSIGFHEFNVDAQGRIAIYRLGPQKDAQGSFTSFTDATTYDRYAPGRTLWPNY   91 (999)
Q Consensus         12     qkrvmtwslriqnddyrhdierygnkitsigfhefnvdaqgriaiyrlgpqkdaqgsftsftdattydryapgrtlwpny   91 (999)
                           +++|..|.-.-..+.....+.+..+++.+.+..+.+++.|.+.+-.                              .+-
T Consensus          7     ~~~v~~y~~~~~~~~~~~~~~~~~~~~t~~~~~~~~~~~~~~~g~~~~~~------------------------------~~~   56 (319)
T 3CZ8_B             7     IAGTLSFYVLRNPDLDRELINDYAPYSSSISIFEYHIAPNGDIANQL------------------------------NDA   56 (319)
T ss_dssp                  CCEEEEEEEEEECGGGC----------CCEEEEEEEEEBCTTSCBCCCC------------------------------CCH
T ss_pred                  ceeEEEEEecCCCccccHHHHHHHhhhccEEEeEEEEeCCCCCCccccC------------------------------ChH


Q ss_pred                  HhhhhhcCccccceeeEee-----eeCHHHHHHHhccchhhhHHHHhHHHHHHHHhccCCCCccceeeeEEecccccccccc
Q Q_8363653_2       92     IEKDMKRWPHIEYYMQFV-----IFGPETVSRLLDSQTAQDNFINNLKTVVARFRKDQNGNDLGYTGIEIDCEGSFSDSK   166 (999)
Q Consensus         92     iekdmkrwphieyymqfv-----ifgpetvsrlldsqtaqdnfinnlktvvarfrkdqngndlgytgieidcegsfsdsk   166 (999)
                           -.....+-+.+...+.+.     -+.......++.+.+..+.|++++...+.++.          +.||.+|.|..
T Consensus         57     ~~~~~~~~~~~~~~~~~~i~~~~~~~~~~~~~~~~~~~~~~~~~~~~~~~~~~~~~---------~dGi~ld~~~~-----   122 (319)
T 3CZ8_B            57     AAIETTWQRRVTPLATITNLTSGGFSTEIVHQVLNNPTARTNLVNNIYDLVSTRG---------YGGVTIDFEQV-----   122 (319)
T ss_dssp                  HHHHHHHHTTCEEEEEEEECEETTEECHHHHHHHHHSHHHHHHHHHHHHHHHHHHHT---------CSEEEEECCSC-----
T ss_pred                  HHHHHHHHCCCeeEEEEEccCCCCCCHHHHHHHHcCHHHHHHHHHHHHHHHHHHhcC---------CCEEEEeCCCC-----


Q ss_pred                  cccCCCCcchhHcHHHHhcceeeeeCCCCcceEEeehhccCC-----CCCchhheecceeeeeecCCCCCCcccceeEEEE
Q Q_8363653_2      167     WDTRAGDDVKYINLLKRIKNEVIIDANPDFKLRINAHAMWGD-----GIPDYYRFHNYKLFAESTDKNGNPLLDEVQIMT   241 (999)
Q Consensus        167     wdtragddvkyinllkrikneviidanpdfklrinahamwgd-----gipdyyrfhnyklfaestdkngnplldevqimt   241 (999)
                              ...+...+..+++.++.+..-..   ..+.+...+.+.+     ...++-....+              +|.+.+|+
```

```
T Consensus      123   ---~~~~~~~~~~~~l~~l~~~~---~~~~~~~~~~~~~~~~~~~~~~~~~~~~~~-------------vd~v~~~~   182 (319)
T 3CZ8_B         123   ---SAADRDLFTGFLRQLRDRLQAGG---YVLTIAVPAKTSDNIPWLRGYDYGGIAV-------------VNYMFIMA   182 (319)
T ss_dssp              ---CGGGHHHHHHHHHHHHHHHHHTT---CEEEEEEECCSCSCCGGGTTCCHHHHHHH-------------SSEEEEEC
T ss_pred              ---CHHHHHHHHHHHHHHHHHHHhhCC---CEEEEEecCcCCCCCcccccCCHHHHHHh-------------CCEEEEee


Q ss_pred              eeccccCCCCCCCCChHHHhcHHHHHHHhcCCCCCCCCcceEeeEEeCCCcccccCCCCCCCCC-CCceeehhHh
Q Q_8363653_2     242   YDFSWSGSAPGPSTPLWWMRNVAEWVKQCFDPSVNPNAKCTIDNVYLGGAGYGRRWPIHSDDN-WGSTVTYRDL   314 (999)
Q Consensus       242   ydfswsgsapgpstplwwmrnvaewvkqcfdpsvnpnakctidnvylggagygrrwpihsddn-wgstvtyrdl   314 (999)
                       |++....+.+++..|+-|.....+++.....+          +.+.+|-..||+.|....... ....++|.+.
T Consensus      183   ~~~~~~~~~~~~~~~~~~~~~~~~~~~~~~~~----------~ki~~gl~~~g~~~~~~~~~~~~~~~~~~~~~~~   246 (319)
T 3CZ8_B         183   YDWHHAGSEPGPVAPITEIRRTIEFTIAQVPS----------RKIIIGVPLYGYDWIIPYQPGTVASAISNQNA   246 (319)
T ss_dssp              CCSSCTTSCSCCSSCHHHHHHHHHHHHTTSCG----------GGEEEECCSCEEEEESSCCTTCCCEEECHHHH
T ss_pred              cCCcCCCCCCCCCCCHHHHHHHHHHHHhcCCH----------HHEEEEeecceeeccCCCCCCCcceeecHHHH
```

Template alignment | Template 3D Structure | PDBe

11. 1KFW_A chitinase B; TIM barrel, HYDROLASE; HET: GOL; 1.74A {Arthrobacter sp.} SCOP: c.1.8.5, d.26.3.1

Probability: 97.6%, E-value: 0.0013, Score: 64.81, Aligned cols: 257, Identities: 17%, Similarity: 0.173, Template Neff: 9.6

```
Q ss_pred              HhcceeeEEEeeecccCCCccEEEEEecCC-CCCCCcccccccCccc--------------ccCCCCCccCcchHhhhhh
Q Q_8363653_2     33   RYGNKITSIGFHEFNVDAQGRIAIYRLGPQ-KDAQGSFTSFTDATTY--------------DRYAPGRTLWPNYIEKDMK   97 (999)
Q Consensus       33   rygnkitsigfhefnvdaqgriaiyrlgpq-kdaqgsftsftdatty--------------dryapgrtlwpnyiekdmk   97 (999)
                       +....+|.|-+.-++++..+-..+-.-++| +.++|++........+              +-+.-...-.-+-+..-.+
T Consensus      34   ~~~~~~thii~~~~~i~~~~~~~~~~~~~~~~~~~~~~~~~~~~~~~~~~~~~~~~~~~~~~~~~~~~~~~~~~~~~~~lk~   113 (435)
T 1KFW_A         34   GTAKNLTHINYSFGNINNQTLTCFMANKAQGTGPNGSDGAGDAWADFGMGYAADKSVSGKADTWDQPLAGSFNQLKQLKA   113 (435)
T ss_dssp              SCGGGCSEEEEEEECBCTTTCSBCCCEECCCSSTTTTTTCEEHCCCCCCCCCTTTSSSSSCCCTTCSCCHHHHHHHHHHT
T ss_pred              ccHHhCcEEEEEeeeecCCcceEEeccccCCCCCCCCCCCCCCchHHhcCcccccccCCCCCCCCCcccccHHHHHHHHH


Q ss_pred              cCccccceeeEeeeeC-HHHHHHHhccchhhhHHHHhHHHHHHHHhh-------ccCCCCcccceeeeEEecccccccccccc
Q Q_8363653_2     98   RWPHIEYYMQFVIFG-PETVSRLLDSQTAQDNFINNLKTVVARFR-------KDQNGNDLGYTGIEIDCEGSFSDSKWDT   169 (999)
Q Consensus       98   rwphieyymqfvifg-petvsrlldsqtaqdnfinnlktvvarfr-------kdqngndlgytgieidcegsfsdskwdt   169 (999)
                       +.|++..++.+--+. ....+.++.+...+..|++++..++..+.       .-+.-..-++.||+||-|.....+.-..
T Consensus      114   ~~~~~kvllsigg~~~~~~~~~~~~~~~~~~~~~~~fi~~i~~~l~~~~~~~~~~~~~~~~~~~~~~~~~~~~fDGvdld~e~~~~~~~~~~~~~~   193 (435)
T 1KFW_A        114   KNPKLKVMISLGGWTWSKNFSKAAATEASRQKLVSSCIDLYIKGNLPNFEGRGGAGAAAGIFDGIDIDWEWPGTNSGLAG   193 (435)
T ss_dssp              TCTTCEEEEEEEECSSSCTTHHHHTSSHHHHHHHHHHHCCCCCSCCEEEETTEEETTTTTTTCCEEEEECSCTTSSCSSTT
T ss_pred              HCCCCEEEEEEcCccccchhHHHHHhcCHHHHHHHHHHHHHHHHHhCCCcccCCCCCcchhccccCeEEEcccCCCCCCCCCC


Q ss_pred              CC----CCcchhHcHHHHhcceeeeC---CCCcceEEeehhccCCCCCchhheecceeeeeecCCCCCccccceeEEEEe
Q Q_8363653_2    170   RA----GDDVKYINLLKRIKNEVIIDA---NPDFKLRINAHAMWGDGIPDYYRFHNYKLFAESTDKNGNPLLDEVQIMTY   242 (999)
Q Consensus      170   ra----gddvkyinllkrikneviida---npdfklrinahamwgdgipdyyrfhnyklfaestdkngnplldevqimty   242 (999)
                       ..    .+...++.++++++.+.--..   ++.+.|-+...+......--..|.....              ...+|-+.+|+|
T Consensus      194   ~~~~~~~~~~~~~~~~~~f~~~Lr~~l~~~~~~~~~~~~~~~~~~~~~~~~~~~~~~~~~~~~l-----------~~~vD~v~i~~y   262 (435)
T 1KFW_A        194   NGVDTVNDRANFKALLAEFRKQLDAYGSTNNKKYVLSAFLPANPADIDAGGWDDPAN-----------FKSLDFGSIQGY   262 (435)
T ss_dssp              CCCCTTTHHHHHHHHHHHHHHHHHHHHHHHHHTTCCCEEEEEECSSHHHHHHHTTTCGGG-----------GGTCSEEEECCS
T ss_pred              CCCCChhHHHHHHHHHHHHHHHHHHHHHHcCcccCCceEEEEEccCChHhhccCCCCChHH-----------hhcCCEEEEeee


Q ss_pred              ecc--ccCCCCCCCCh--------------HHHhcHHHHHHHhcCCCCCCCCcceEeeEEeCCCcccccCCCCCCCCCCC
Q Q_8363653_2    243   DFS--WSGSAPGPSTPL--------------WWMRNVAEWVKQCFDPSVNPNAKCTIDNVYLGGAGYGRRWPIHSDDNWG   306 (999)
Q Consensus      243   dfs--wsgsapgpstpl--------------wwmrnvaewvkqcfdpsvnpnakctidnvylggagygrrwpihsddnwg   306 (999)
                       ++.  |.+..+++..|+              |....+..|+++.+.|              ..+.+|-..||+.|.........
T Consensus      263   d~~~~~~~~~~~~~~~~~~l~~~~~~~~~~~~~~~~~~~~~~~~~~~~~~~~~gv~~----------~Kivlgl~~~g~~~~~~~~~~~~~~~~   332 (435)
T 1KFW_A        263   DLHGAWNPTLTGHQANLYDDPADPRAPSKKFSADKAVKKYLAAGIDP----------KQLGLGLAAYGRGWTGAKNVSPW   332 (435)
T ss_dssp              CSSCTTSTTBCCCSSCSSCCTTCCSCGGGCCCHHHHHHHHHHTTCCG----------GGEEEEEESEEEEEESCCCSSSS
T ss_pred              cCCCCCCcCcCCCCCcccCCCCCCCCCCcccccHHHHHHHHHHCCCCH----------HHeeEEccceeeeeecCCCCCCC


Q ss_pred              ceee
Q Q_8363653_2    307   STVT   310 (999)
Q Consensus      307   stvt   310 (999)
                       ....
T Consensus      333   ~~~~   336 (435)
T 1KFW_A        333   GPAT   336 (435)
T ss_dssp              CBCS
T ss_pred              CCCC
```

Template alignment | Template 3D Structure | PDBe

12. 5GZT_B Chitinase; HYDROLASE; HET: FMT; 2.1A {Paenibacillus sp. FPU-7}

Probability: 97.57%, E-value: 0.00053, Score: 74.19, Aligned cols: 247, Identities: 18%, Similarity: 0.25, Template Neff: 10.3

```
Q ss_pred              ceeeEEEeeecccCCCccEEEEEecCCCCCCCCcccccccCccccccCCCCCccCcchHhhhhhcCccccceeeEeeeeCHHH
Q Q_8363653_2     36   NKITSIGFHEFNVDAQGRIAIYRLGPQKDAQGSFTSFTDATTYDRYAPGRTLWPNYIEKDMKRWPHIEYYMQFVIFGPET   115 (999)
Q Consensus       36   nkitsigfhefnvdaqgriaiyrlgpqkdaqgsftsftdattydryapgrtlwpnyiekdmkrwphieyymqfvifgpet   115 (999)
                       .+.|.|-+.-..++..+.+.+..-.+-.+..    .++....+.+....-.-+...+..-.++.|++.-.+.+--.....
T Consensus      299   ~~~thi~yaf~~~~~~~~~~~~~~~~~~~~~~~~~~----~~~~~~~~~~~~~~~~~~~~~~~~~~~~~lk~~~~~~~kvllsiGG~~~~~   374 (1136)
T 5GZT_B        299   TKFTHLYYAFGRINNGKVVTIKEDAKWTEDP----TITEADRIKRRNNPDESNLAYLTGLKAKNPNLKVLVSIGGWEAEG   374 (1136)
T ss_dssp              GGCSEEEEEEEEEEETTEEEEGGGCHHHHTCT----TSCHHHHHHHHHTCCHHHHHHHHHGGGGTCTTCEEEEEEECTTCCC
T ss_pred              HHCcEEEEEEEEEEeCCcEEEeCCCcccCCCC----ccchhHHhHhcCCCCcccHHHHHHHHHHCCCCeEEEEEcCCCCcCc


Q ss_pred              HHHHhccchhhhHHHHhHHHHHHHHhhccCCCCcccceeeeEEecc--ccccccccccCCCCCcchhHcHHHHhcceeeeeCC
Q Q_8363653_2    116   VSRLLDSQTAQDNFINNLKTVVARFRKDQNGNDLGYTGIEIDCE--GSFSDSKWDTRAGDDVKYINLLKRIKNEVIIDAN   193 (999)
Q Consensus      116   vsrlldsqtaqdnfinnlktvvarfrkdqngndlgytgieidce--gsfsdskwdtragddvkyinllkrikneviidan   193 (999)
                       .+.++.+......+|++++..++         |+.||+||-|  +....+.......|...|+.+|+.++...--...
```

```
T Consensus      375   f~~~~~~~~~~~~f~~~i~~~~~~~---------gfDGvdiDwe~p~~~~~~~~~~~~~~~~~~~~~l~~Lr~~l~~~~~   445 (1136)
T 5GZT_B         375   FSDAALTPESREVFANSALDFMNKY---------NLDGIDLDWEYPVYGAWGVIKSRPEDKANFTALLKLLREKLDAQST   445 (1136)
T ss_dssp              HHHHTSSHHHHHHHHHHHHHHHHHHT---------TCSEEEEECSCTTTHHHHTSCCCTTHHHHHHHHHHHHHHHHHHHHHH
T ss_pred              hHHHhcCHHHHHHHHHHHHHHHHHHc---------CCCCcEeeccCCCCCcCCCCCCCHHHHHHHHHHHHHHHHHHHHhccc


Q ss_pred              CCcc-eEEeehhccCCCCCchhheecceeeeeecCCCCCccccceeEEEEeecccc-CCCCCCCCchH--------HHhcH
Q Q_8363653_2    194   PDFK-LRINAHAMWGDGIPDYYRFHNYKLFAESTDKNGNPLLDEVQIMTYDFSWS-GSAPGPSTPLW--------WMRNV   263 (999)
Q Consensus      194   pdfk-lrinahamwgdgipdyyrfhnyklfaestdkngnplldevqimtydfsws-gsapgpstplw--------wmrnv   263 (999)
                       ..-+ +.+...+.......+.|.+...              ...+|-+.+|+||+... ....++..|++         +-..+
T Consensus      446   ~~~~~~~l~~~~~~~~~~~~~~~~~~~~~l-----------~~~vD~i~vm~Yd~~~~~~~~~~~~~~~~~~~~~~~~~l~~~~~~~~   514 (1136)
T 5GZT_B         446   TTNKYYELAIAAGASKTYTDSVELTKI-----------TPYLDYINLMTYDLHGGWDPATSHHTAVYSATNNQLSVDSTV   514 (1136)
T ss_dssp              HHCCCCEEEEEECCSHHHHTTSCHHHH-----------GGGCSEEEECCCCSSCTTSSBCCCSSCSSCCSTTCCCHHHHH
T ss_pred              ccCCeEEEEEEecCCchhcccCCHHHH-----------HHhccEEEEEEeecCCCCCCCCCCCCCCCCCCCCCCCCCHHHHH


Q ss_pred              HHHHHHhcCCCCCCCCcceEeeEEeCCCccccCCCC------------CCCCCCCceeehhHhcc
Q Q_8363653_2    264   AEWVKQCFDPSVNPNAKCTIDNVYLGGAGYGRRWPI------------HSDDNWGSTVTYRDLVD   316 (999)
Q Consensus      264   aewvkqcfdpsvnpnakctidnvylggagygrrwpi------------hsddnwgstvtyrdlvd   316 (999)
                       ..|+++-+.++            .+.||-..|||.|..              .........++|.++..
T Consensus      515   ~~~~~~g~p~~----------KlvlGlp~yg~~~~~~~~~~~~~~~~~~~~~~~~g~~~~~~i~~   569 (1136)
T 5GZT_B         515   KLYLNNGVPAE----------KLMVGGAFYSRVWQNVENKGTGLSEKAGSQAGSPGTIVYSELVN   569 (1136)
T ss_dssp              HHHHHTTCCGG----------GEEEEEESEEEEECSBCSSTTSTTCBBCCCTTCCSEEEHHHHHH
T ss_pred              HHHHHCCCCHH----------HEEEeccceeEeeecCCCCCCCCccccccCCCCCceeeHHHHHH
```

Template alignment | Template 3D Structure | PDBe

13. **4WIW_F Glycoside hydrolase family 18; Structural Genomics, PSI-Biology, Midwest Center for Structural Genomics, MCSG, glycoside hydrolase family 18, HYDROLASE; HET: CA, MSE; 2.637A {Desulfitobacterium hafniense}**

Probability: 97.5%, E-value: 0.0013, Score: 59.03, Aligned cols: 189, Identities: 22%, Similarity: 0.36, Template Neff: 11.4

```
Q ss_pred              hhhhcCccccceeeEeeeeCHHHHHHHhccchhhhHHHHhHHHHHHHHhhccCCCCccceeeeEEecccccccc-cccCCC
Q Q_8363653_2     94   KDMKRWPHIEYYMQFVIFGPETVSRLLDSQTAQDNFINNLKTVVARFRKDQNGNDLGYTGIEIDCEGSFSDSK-WDTRAG   172 (999)
Q Consensus       94   kdmkrwphieyymqfvifgpetvsrlldsqtaqdnfinnlktvvarfrkdqngndlgytgieidcegsfsdsk-wdtrag   172 (999)
                       ...++.+++...+-+- ...+....++.+....+.|++++...+....           +.||+||.|..-.+.. ....+.
T Consensus       66   ~~~~~~~~~~~k~~~~ig-~~~~~~~~~~~~~~~~~~~~~~~~i~~~~~~~~~~---------~dGvdid~e~~~~~~~~~~~~~~~~~   135 (349)
T 4WIW_F          66   TNMAHGLGMKVLPLIN-GSGATLNTLLKSPAAREKLIGEIVVLLKNTN---------ADGVVIDFETPLDYGDVKDPYDG   135 (349)
T ss_dssp              HHHHHHTTCEECCEEE-ECHHHHHHHHHCHHHHHHHHHHHHHHHHHHHC---------CCEEEEEECSCCCCCCSSCSSCS
T ss_pred              HHHHHhcCCEEEEEEc-CCCchHHHHHCCHHHHHHHHHHHHHHHHHHcC---------CCeEEEecCCCccccCCCCCCCcc


Q ss_pred              CcchhHcHHHHhcceeeeeCCCCcceEEeehhccCCCCCchhheecceeeeeecCCCCCccccceeEEEEeeccccCCCCC
Q Q_8363653_2    173   DDVKYINLLKRIKNEVIIDANPDFKLRINAHAMWGDGIPDYYRFHNYKLFAESTDKNGNPLLDEVQIMTYDFSWSGSAPG   252 (999)
Q Consensus      173   ddvkyinllkrikneviidanpdfklrinahamwgdgipdyyrfhnyklfaestdkngnplldevqimtydfswsgsapg   252 (999)
                       +-..++++++.++...      .+..+.-+-+-.......+.+....+++..           .+.+|.+.+|+|++.+..+.++
T Consensus      136   ~~~~~~~~~i~~l~~~l----~~~~~~~~~~~~~~~~~~~~~~~~~~~~~~~~l--------~~~vD~~~i~~y~~~~~~~~~~   203 (349)
T 4WIW_F         136   VRNDLTAFMESLHSEL----QSMNKLVVMAVMPRMSSSQYWLDAYDYEAL--------SHAVDYLHVMTYDHHYRTSAPG   203 (349)
T ss_dssp              SHHHHHHHHHHHHHHH----HHTTCEEEEEECCCCSTTCGGGTTCCHHHH--------HHSCSEEEECCCCSSCTTSCSC
T ss_pred              hhHHHHHHHHHHHHHH----HhcCCeeEEEeecCCCCccccchhcCCHHHH--------HHHCCEEEeecCCCCCCCCCCC


Q ss_pred              CCCChHHHhcHHHHHHHh-cCCCCCCCCcceEeeEEeCCCccccCCCCCCCCCCC------ceeehhHh
Q Q_8363653_2    253   PSTPLWWMRNVAEWVKQC-FDPSVNPNAKCTIDNVYLGGAGYGRRWPIHSDDNWG------STVTYRDL   314 (999)
Q Consensus      253   pstplwwmrnvaewvkqc-fdpsvnpnakctidnvylggagygrrwpihsddnwg------stvtyrdl   314 (999)
                       +..|+.|.....++.... +.++            .+.+|=.-||+.|......-+           ..++|.++
T Consensus      204   ~~~~~~~~~~~~~~~~~~~g~~~~----------klv~gi~~~g~~~~~~~~~~~~~~~~~~~~~~~~~~~~~   262 (349)
T 4WIW_F         204   PIAPYPWIKQVLTYIQGQGVDMS----------KVLMGIPYYGRDWVVDGKDANGNPTYNSTAFGYSKA   262 (349)
T ss_dssp              CSSCHHHHHHHHHHHHTTCCGG----------GEEECCBSCEEEEEEEECTTSCEEEEEEECHHHH
T ss_pred              CCCCchHHHHHHHHHHhcCCCHH----------HEEEEccCCcccceeeCCCCCCCCcccccCCchHHH
```

Template alignment | Template 3D Structure | PDBe

14. **3AQU_A At4g19810; stress response, TIM barrel, Hydrolase, chitin; HET: FLC; 2.01A {Arabidopsis thaliana}**

Probability: 97.49%, E-value: 0.00064, Score: 59.47, Aligned cols: 214, Identities: 18%, Similarity: 0.299, Template Neff: 12.1

```
Q ss_pred              CcccccccCccccCCCCCCccCcchHhhhhhcCccccceeeEeee--eCHHHHHHHhccchhhhHHHHhHHHHHHHHhhccC
Q Q_8363653_2     67   GSFTSFTDATTYDRYAPGRTLWPNYIEKDMKRWPHIEYYMQFVI--FGPETVSRLLDSQTAQDNFINNLKTVVARFRKDQ   144 (999)
Q Consensus       67   gsftsftdattydryapgrtlwpnyiekdmkrwphieyymqfvi--fgpetvsrlldsqtaqdnfinnlktvvarfrkdq   144 (999)
                       ..+....+........+....+...+..--++.|++...+.+.-   ........+++.+....++|++++...+..+
T Consensus       32   ~~~~~~~~~~~~~~~~~~~~~~~~~~~~~~~~~~~~~~~~~~~~~~~~igg~~~~~~~~~~~~~~~~~~~~~~~~~~~~~~~~~~~----   107 (356)
T 3AQU_A          32   AFADLNSQTNQVTVSSANQPKFSTFTQTVQRRNPSVKTLLSIGGGIADKTAYASMASNPTSRKSFIDSSIRVARSY----   107 (356)
T ss_dssp              EEEEEETTTTEEECCTTTHHHHHHHHHHHTTTCTTCEEEEEEECTTSCHHHHHHHHHSHHHHHHHHHHHHHHHHHHHH----
T ss_pred              EeeeecCCCCceecCcccCcchhHHHHHHHHHCCCCcEEEEECCCccccchHHHhcCHHHHHHHHHHHHHHHHHHHh----


Q ss_pred              CCCccceeeeEEecccccccccccCCCCcchhHcHHHHhcceeeeeCCCCcceEEeehhccCCCCCchhheecceeeee
Q Q_8363653_2    145   NGNDLGYTGIEIDCEGSFSDSKWDTRAGDDVKYINLLKRIKNEVIIDANPDFKLRINAHAMWGDGIPDYYRFHNYKLFAE   224 (999)
Q Consensus      145   ngndlgytgieidcegsfsdskwdtragddvkyinllkrikneviidanpdfklrinahamwgdgipdyyrfhnyklfae   224 (999)
                           ++.||.||.|       +.....+...+++.++....-..-...+-.+..-..............+++..++
T Consensus      108   -----~~dGidid~~-------~~~~~~~~~~~~~~~~l~~l~~~~l~~~~~~~~~~~~~~~~~~~~~~~~~~~~~~~~~~~   175 (356)
T 3AQU_A         108   -----GFHGLDLDWE-------YPSSATEMTNFGTLLREWRSAVVAEASSSGKPRLLLAAAVFYSNNYYSVLYPVSAVAS   175 (356)
T ss_dssp              -----TCSEEEEECS-------CCCSHHHHHHHHHHHHHHHHHHHHHHHHHHCSCCCEEEEEEESSSEETTEECCHHHHHH
T ss_pred              -----CCCcEEEecc-------CCCCHHHHhHHHHHHHHHHHHHHHHHHHHHhcCCCeEEEEEEEccCCcchhcCCCHHHHHH


Q ss_pred              ecCCCCCccccceeEEEEeecccc--CCCCCCCCCh--------HHHhcHHHHHHHhcCCCCCCCCcceEeeEEeCCCcccc
Q Q_8363653_2    225   STDKNGNPLLDEVQIMTYDFSWS--GSAPGPSTPL--------WWMRNVAEWVKQCFDPSVNPNAKCTIDNVYLGGAGYG   294 (999)
Q Consensus      225   stdkngnplldevqimtydfsws--gsapgpstpl--------wwmrnvaewvkqcfdpsvnpnakctidnvylggagyg   294 (999)
```

```
                                   .        +|.+.+|+||+.+.  ...+++..|+        ..-..+..|++.-..+          +.+.+|-.-||
T Consensus        176             ~---------D~v~~~~y~~~~~~~~~~~~~~~l~~~~~~~~~~~~~~~~~~~~g~~~-----------~kl~~gi~~~g  237 (356)
T 3AQU_A           176             S--------LDWVNLMAYDFYGPGWSRVTGPPAALFDPSNAGPSGDAGTRSWIQAGLPA----------KKAVLGFPYYG  237 (356)
T ss_dssp                            H--------CSEEEEECCCCCTTTCSBCCCTTCSCCTTCSSCCHHHHHHHHHHTTCCG----------GGEEEEEESEE
T ss_pred                            H--------CCEEEeEecccCCCcccCCCCCCccCCCCCCCCChHHHHHHHHHcCCCH----------HhEEEeeccce


Q ss_pred                            cCCCCCCCCCCC-------------ceeehhHh
Q Q_8363653_2       295             RRWPIHSDDNWG-------------STVTYRDL   314 (999)
Q Consensus        295             rrwpihsddnwg-------------stvtyrdl   314 (999)
                                   +.|...+....+             ..++|.++
T Consensus        238             ~~~~~~~~~~~~~~~~~~~~~~~~~~~~~~~~~~~   270 (356)
T 3AQU_A           238             YAWRLTNANSHSYYAPTTGAAISPDGSIGYGQI   270 (356)
T ss_dssp                            EEEEESCTTCCSTTCBEEEECSSTTCEEEHHHH
T ss_pred                            EEeEccCCCCCCCCCCCcCCCCCCCCceeHHHH
```

Template alignment | Template 3D Structure | PDBe

15. 6INX_A N-acetylglucosaminidase; N-acetylglucosaminidase, GH18, hydrolase, STRUCTURAL PROTEIN; 1.429A {Paenibacillus barengoltzii}

Probability: 97.41%, E-value: 0.00068, Score: 60.27, Aligned cols: 194, Identities: 16%, Similarity: 0.262, Template Neff: 11.6

```
Q ss_pred                            HhhhhhcCccccceeeEeeeeCHHHHHHHhccchhhhHHHHhHHHHHHHHhhccCCCCccceeeeEEecc-cccccccccC
Q Q_8363653_2        92             IEKDMKRWPHIEYYMQFVIFGPETVSRLLDSQTAQDNFINNLKTVVARFRKDQNGNDLGYTGIEIDCE-GSFSDSKWDTR   170 (999)
Q Consensus         92             iekdmkrwphieyymqfvifgpetvsrlldsqtaqdnfinnlktvvarfrkdqngndlgytgieidce-gsfsdskwdtr   170 (999)
                                   +..-.++.|++...+.+--+..+..+.++.+...+..|++++..++.++         ++.||+||.| ...++......
T Consensus         53             ~~~~~~~~~~~~k~~~~igg~~~~~~~~~~~~~~~~~~~~~~~~~~~~~i~~~~~~~----------~~DGv~id~e~~~~~~~~~~~~~~   123 (345)
T 6INX_A            53             LPELKRENPDLTILLSVGGWSAGGFSEAASTEAGRQAMAESAVRAVTEY---------ALDGVDLDWEYPCYAEAGIAAS   123 (345)
T ss_dssp                            HHHHHHHCTTCEEEEEEECTTCCCHHHHHTSHHHHHHHHHHHHHHHHHHS---------SCCEEEEECSCTTCCTTTSCCC
T ss_pred                            HHHHHHHCCCCEEEEEEcCcccccHHHHhcCHHHHHHHHHHHHHHHHHHc---------CCCcEEEcccCCCcccCCCCC


Q ss_pred                            CCCcchhHcHHHHhcceeeeeCCCCcc-eEEeehhccCCCCCchhheecceeeeeecCCCCCccccceeEEEEeecccc-C
Q Q_8363653_2       171             AGDDVKYINLLKRIKNEVIIDANPDFK-LRINAHAMWGDGIPDYYRFHNYKLFAESTDKNGNPLLDEVQIMTYDFSWS-G   248 (999)
Q Consensus        171             agddvkyinllkrikneviidanpdfk-lrinahamwgdgipdyyrfhnyklfaestdkngnplldevqimtydfsws-g   248 (999)
                                   ..+...+..+++.+++..--...+..+ +.+.+-..........+.+...             ...+|-+.+|+|++.+. .
T Consensus        124             ~~~~~~~~~~~~~~~~~~l~~~l~~~~~~~~~~~~~~~~ls~~~~~~~~~~~~~~~~~~~~~~~~-----------~~~~D~v~~~~y~~~~~~~~~   192 (345)
T 6INX_A           124             PDDKANFTLLLRTMREALDRQGERDGRHYWLTIAAGADQYYIDGTEMAEV-----------QRYLDFVQLMTYDMRGGFQ   192 (345)
T ss_dssp                            TTHHHHHHHHHHHHHHHHHHHHHHHHHHTSCCEEEEEEECSHHHHHTSCHHHH-----------GGGCSEEEEECSSSSSTTC
T ss_pred                            hHHHHHHHHHHHHHHHHHHHHHHcccccCCeEEEEEEecCChhhccCCCHHHH-----------HHHCCEEEeeccccCCCCc


Q ss_pred                            CCCCCCCChH----------HHhcHHHHHHHHhcCCCCCCCCcceEeeEEeCCCccccCCCCCCC----------CCCCce
Q Q_8363653_2       249             SAPGPSTPLW----------WMRNVAEWVKQCFDPSVNPNAKCTIDNVYLGGAGYGRRWPIHSD----------DNWGST   308 (999)
Q Consensus        249             sapgpstplw----------wmrnvaewvkqcfdpsvnpnakctidnvylggagygrrwpihsd----------dnwgst   308 (999)
                                   ..+++..|++          |-+.+..|+...++++          .+.+|-..||+.|+....          ++.+..
T Consensus        193             ~~~~~~~~~~~~~~~~~~~~~~~~~~~~~~~~~~~~~~~----------kl~~g~~~~g~~~~~~~~~~~~~~~~~~~~~~~~~~~~~   262 (345)
T 6INX_A           193             TLTGHHTNLYTGTGDLFRISVDASVNLFVRAGVPKE----------KIVIGAAFYSRMWKDVPNVNRGLYQMSPGSGGYG   262 (345)
T ss_dssp                            SBCCCSSCSSCCTTCSCCCCHHHHHHHHHHTTCCGG----------GEEEEEESEEEEECSCCSSGGGTTCBCSSCCEEE
T ss_pred                            CccCCCCCCCCCCCCchhcCHHHHHHHHHHcCCCHH----------HEEEeeecceeeeCCCCccCCccccCCCCCCCC


Q ss_pred                            eehhHhc
Q Q_8363653_2       309             VTYRDLV   315 (999)
Q Consensus        309             vtyrdlv   315 (999)
                                   .+|.+.+
T Consensus        263             ~~~~~~~~   269 (345)
T 6INX_A           263             PDFTELA   269 (345)
T ss_dssp                            EEHHHCC
T ss_pred                            CChHHHH
```

Template alignment | Template 3D Structure | PDBe

16. 5WV8_A Chitinase; Ostrinia furnacalis, chitinase, three-dimensional structure, chitin metabolism, HYDROLASE; 2.042A {Ostrinia furnacalis}

Probability: 97.34%, E-value: 0.001, Score: 65.21, Aligned cols: 198, Identities: 18%, Similarity: 0.277, Template Neff: 10.2

```
Q ss_pred                            ccCCCCCccCcchHhhhhhcCccccceeeEeeeeC--HHHHHHHhccchhhhHHHHhHHHHHHHHhhccCCCCccceeeeEE
Q Q_8363653_2        79             DRYAPGRTLWPNYIEKDMKRWPHIEYYMQFVIFG--PETVSRLLDSQTAQDNFINNLKTVVARFRKDQNGNDLGYTGIEI   156 (999)
Q Consensus         79             dryapgrtlwpnyiekdmkrwphieyymqfvifg--petvsrlldsqtaqdnfinnlktvvarfrkdqngndlgytgiei   156 (999)
                                   +.......-+...|..--.+-|++...+.+--..  ++..+.++.+.....+|++++..++..+         |+.||+|
T Consensus        144             ~~~~~~~~~~~~~~~~i~~lk~~~~~~~kv~lsiGG~~~~~~~~~~~~~~~~~~~~~~~~~~~f~~~~i~~~~~~~~----------~~DGidl   214 (482)
T 5WV8_A           144             DETKDGKTGLYDRINALKKANPKLKTLLAIGGWSFGTQKFKEMSATRYARQTFIYSAIPYLRDR---------NFDGLDI   214 (482)
T ss_dssp                            GSCBTTBCCHHHHHHTHHHHCTTCEEEEEEECTTTCSHHHHHHHSSHHHHHHHHHHHHHHHHHHT---------TCCEEEE
T ss_pred                            CcccCccccHHHHHHHHHHHCCCCeEEEEECCCccCChhhHHHhcCHHHHHHHHHHHHHHHHHHC---------CCCcEEe


Q ss_pred                            ecccccccccccCCCCcchhHcHHHHhcceeee------eCCCCcceEEeehhccCCCCCchhheecceeeeeecCCCC
Q Q_8363653_2       157             DCEGSFSDSKWDTRAGDDVKYINLLKRIKNEVII------DANPDFKLRINAHAMWGDGIPDYYRFHNYKLFAESTDKNG   230 (999)
Q Consensus        157             dcegsfsdskwdtragddvkyinllkrikneviI------danpdfklrinahamwgdgipdyyrfhnyklfaestdkng   230 (999)
                                   |.|..-.       ..+...++.++++++...--      ..+....+.+.+.+.........++-.+..+
T Consensus        215             D~E~~~~~-------~~~~~~~~~~~~~l~~~Lr~~l~~~~~~~~~~~~~~~~~~~ls~~~~~~~~~~~~~~~~~~~~~~~~~~~~~~~~~~-----------   276 (482)
T 5WV8_A           215             DWLYPKG-------GDDKKNYVLLLKELREAFEAEAQEVKKPRLLLTAAVPVGPDNIKSGYDVPAVASY-----------   276 (482)
T ss_dssp                            ECSCCCS-------HHHHHHHHHHHHHHHHHHHHHHHHHHHHHTSCCCEEEEEECCCHHHHHHHCCHHHHHHH-----------
T ss_pred                            CCcCCCC-------ccchhhHHHHHHHHHHHHHHHHHHHHHHHHHHcCCCCeEEEEEeCCHHHHhcCCCHHHHHHH-----------


Q ss_pred                            CccccceeEEEEeecccc-CCCCCCCCChH-------------HHhcHHHHHHHHhcCCCCCCCCcceEeeEEeCCCCcccC
Q Q_8363653_2       231             NPLLDEVQIMTYDFSWS-GSAPGPSTPLW-------------WMRNVAEWVKQCFDPSVNPNAKCTIDNVYLGGAGYGRR   296 (999)
Q Consensus        231             nplldevqimtydfsws-gsapgpstplw-------------wmrnvaewvkqcfdpsvnpnakctidnvylggagygrr   296 (999)
```

```
                          +|-+.+|+||+..+ ...+++..|+.              .-..+..|+++.+++          +.+.+|-..||+.
T Consensus       277    ---vD~v~vm~Yd~~~~~~~~~~~~~a~l~~~~~~~~~~~~~~~~~~~~~~~~~~~~g~~~-----------~KlvlG~p~yg~~   343 (482)
T 5WV8_A          277    ---LDFINLMAYDFHGKWERETGHNAPLYAPSSDSEWRKQLSVDHAAHLWVKLGAPK----------EKLIIGMPTYGRT   343 (482)
T ss_dssp               ---CSEEEECCCCSSCTTSSBCCCSSCSSCCTTSCTTGGGCSHHHHHHHHHHTTCCG----------GGEEEEEESEEEE
T ss_pred               ---CCEEEEEcCCcCCCCCcccCCCCCCCCCCCChhHHhcCHHHHHHHHHHcCCCH----------HHEEEEEecceeE


Q ss_pred                CCCCCCC------------------CCCceeehhHhcc
Q Q_8363653_2      297    WPIHSDD------------------NWGSTVTYRDLVD   316 (999)
Q Consensus        297    wpihsdd------------------nwgstvtyrdlvd   316 (999)
                         |.....+                  .....++|+++..
T Consensus       344    ~~~~~~~~~~~~~~~~~~~~~~~~~~~~~~~~~g~~~~~~~~~~~   381 (482)
T 5WV8_A          344    FTLSNPNNFKVNSPASGGGKAGEYTKESGFLAYYEVCE   381 (482)
T ss_dssp               EEESSTTCCSTTCBEEEECCCCTTTCCTTEEEHHHHHH
T ss_pred               EEcCCCCCCCCCCCCCCCCCCCCcccCCccccHHHHHH
```

Template alignment | Template 3D Structure | PDBe

17. 5ZL9_A Chitinase AB; Chitinase, engineered protein, HYDROLASE; HET: GOL; 2.6A {Serratia marcescens}

Probability: 97.23%, E-value: 0.001, Score: 67.13, Aligned cols: 193, Identities: 18%, Similarity: 0.267, Template Neff: 10.3

```
Q ss_pred                HhhhhhcCccccceeeEeeeeCHHHHHHHhccchhhhHHHHhHHHHHHHHhhccCCCCccc-eeeeEEecc--ccccccccc
Q Q_8363653_2      92     IEKDMKRWPHIEYYMQFVIFGPETVSRLLDSQTAQDNFINNLKTVVARFRKDQNGNDLG-YTGIEIDCE--GSFSDSKWD   168 (999)
Q Consensus        92     iekdmkrwphieyymqfvifgpetvsrlldsqtaqdnfinnlktvvarfrkdqngndlg-ytgieidce--gsfsdskwd   168 (999)
                         |.+-.++.|++.-.+.+--........+.+....+.|++++..++.+          .+ +.||+||.|  +......--
T Consensus       233    i~~lk~~~pg~kvllSiGG~~~~~~~~~~~~~~~~~~~~~~f~~~i~~~v~~----------~g~~DGiDiD~E~~~~~~~~~~~~~~   303 (618)
T 5ZL9_A          233    LMALKQAHPDLKILPSIGGWTLSDPFFFMGDKVKRDRFVGSVKEFLQT---------WKFFDGVDIDWEFPGGKGANPNL   303 (618)
T ss_dssp                HHHHHHHCTTCEEEEEEEEESSSCGGGGGTTSHHHHHHHHHHHHHHHHH---------CTTCCEEEEEESCTTSCSSCTTC
T ss_pred                HHHHHHHCCCCeEEEEeCCCcccCCchhhcCCHHHHHHHHHHHHHHHHH---------cCCCCeEEEeccCCCCCCCCCCC


Q ss_pred                cCCCCcchhHcHHHHhcceeeeeC---CCCcceEEeehhccCCCCCchhheec--ceeeeeecCCCCCcccceeEEEEee
Q Q_8363653_2      169    TRAGDDVKYINLLKRIKNEVIIDA---NPDFKLRINAHAMWGDGIPDYYRFHN--YKLFAESTDKNGNPLLDEVQIMTYD   243 (999)
Q Consensus        169    tragddvkyinllkrikneviida---npdfklrinahamwgdgipdyyrfhn--yklfaestdkngnplldevqimtyd   243 (999)
                         ....+...|..++++++...--..   ++.+.|-+-..+.     +.+.....  ++..++         .+|.|.+|+||
T Consensus       304    ~~~~~~~~~~~~~~l~~Lr~~l~~~~~~~~~~~~~~~~~ls~a~~~~~~----~~~~~~~~~~~~~~~l~~~---------~vD~v~vm~yd   370 (618)
T 5ZL9_A          304    GSPQDGETYVLLMKELRAMLDQLSVETGRKYELTSAISAG-----KDKIDKFYSFVNVAQN--------SMDHIFLMSYD   370 (618)
T ss_dssp                CCTHHHHHHHHHHHHHHHHHHHHHHHHCCCCEEEEEEESS-----STTHHHHHHHHHHHGG--------GCSEEEEECCC
T ss_pred                CCcchHHHHHHHHHHHHHHHHHHHhhhhCCeEEEEEEeecC-----HHHHHHHHHHHHHHHH--------hCCEEEEEccc


Q ss_pred                ccccCCCC-------------CCCCChHHHhcHHHHHHHHhcCCCCCCCCcceEeeEEeCCCcccccCCCCCCCCCCC----
Q Q_8363653_2      244    FSWSGSAP-------------GPSTPLWWMRNVAEWVKQCFDPSVNPNAKCTIDNVYLGGAGYGRRWPIHSDDNWG----   306 (999)
Q Consensus        244    fswsgsap-------------gpstplwwmrnvaewvkqcfdpsvnpnakctidnvylggagygrrwpihsddnwg----   306 (999)
                         |..+.+.+             .+..+..|...+..|+++.+.++          .+.+|-..|||.|...+...-+
T Consensus       371    ~~~~~~~~~~~~~~~~~l~~~~~~~~~~~~~~~~~~~~~~~~~~~gv~~~----------Kl~lGlp~yg~~~~~~~~~~~~~~~~~~   440 (618)
T 5ZL9_A          371    FYGAFDLKNLGHQTALNAPAWKPDTAYTTVNGVNALLAQGVKPG----------KIVVGTAMYGRGWTGVNGYQNNIPFT   440 (618)
T ss_dssp                SSCTTCSSSCCCSSCSSCCTTCTTCSCCHHHHHHHHHHTTCCGG----------GEEEEEESEEEEEESCBSCSTTCGGG
T ss_pred                ccCCcccccCCCCCCCCCCCCCCCcccHHHHHHHHHHCCCCHH----------HEEEEecceeeeeeCCCCCCCCCCCcc


Q ss_pred                -------------ceeehhHhcc
Q Q_8363653_2      307    -------------STVTYRDLVD   316 (999)
Q Consensus        307    -------------stvtyrdlvd   316 (999)
                                      ..++|+++..
T Consensus       441    ~~~~~~~~~~~~~~~g~~~~y~ei~~   463 (618)
T 5ZL9_A          441    GTATGPVKGTWENGIVDYRQIAG   463 (618)
T ss_dssp                SBCSEECCCSSSTTEEEHHHHHH
T ss_pred                ccCCCCCCCCccCceeeHHHHHH
```

Template alignment | Template 3D Structure | PDBe

18. 1JND_A Imaginal disc growth factor-2; IDGF, imaginal disc, growth factor, chitinase, insulin receptor, heparin, HORMONE-GROWTH FACTOR COMPLEX; HET: BMA, MAN, NAG; 1.3A {Drosophila melanogaster} SCOP: c.1.8.5, d.26.3.1

Probability: 97.21%, E-value: 0.0029, Score: 61.27, Aligned cols: 196, Identities: 18%, Similarity: 0.313, Template Neff: 10.1

```
Q ss_pred                CcchHhhhhhcCccccceeeEeeeeC-------HHHHHHHhccchhhhHHHHhHHHHHHHHhhccCCCCccceeeeEEeccc
Q Q_8363653_2      88     WPNYIEKDMKRWPHIEYYMQFVIFG-------PETVSRLLDSQTAQDNFINNLKTVVARFRKDQNGNDLGYTGIEIDCEG   160 (999)
Q Consensus        88     wpnyiekdmkrwphieyymqfvifg-------petvsrlldsqtaqdnfinnlktvvarfrkdqngndlgytgieidceg   160 (999)
                         +-..+..-.++.|++..++.+--.+       .+-.+.++.+...+.|++++...+..+          |+.||.||.|.
T Consensus       63     ~~~~~~~~l~~~~~p~~k~~lsigg~~~~~~~~~~~~~~~~~~~~~~~~~~~~~~~~~~~~~~~~~~~~~~~~----------~~dGidid~e~   133 (420)
T 1JND_A          63     QFSEVTSLKRKYPHLKVLLSVGGDHDIDPDHPNKYIDLLEGEKVRQIGFIRSAYELVKTY---------GFDGLDLAYQF   133 (420)
T ss_dssp                HHHHHHGGGGTSTTCEEEEEEETTCCCCTTSTTHHHHHHTCCHHHHHHHHHHHHHHHHHHT---------TCSEEEEECCC
T ss_pred                hHHHHHHHHHHHCCCCeEEEEECCCcccCCCCCchHHHHHcccHHHHHHHHHHHHHHHHHHHH---------CCCcEEEeccC


Q ss_pred                ccccccccc-----------------------CCCCcchhHcHHHHhcceeeeeCCCCcceEEeehhccCCCCC-chhhe
Q Q_8363653_2      161    SFSDSKWDT-----------------------RAGDDVKYINLLKRIKNEVIIDANPDFKLRINAHAMWGDGIP-DYYRF   216 (999)
Q Consensus        161    sfsdskwdt-----------------------ragddvkyinllkrikneviidanpdfklrinahamwgdgip-dyyrf   216 (999)
                         ......=..                       ...+..+|..+++.++++.--..   +.+.+.+...+.+..... ++-+.
T Consensus       134    ~~~~~~~~~~~~~~~~~~~~~~~~~~~~~~~~~~~~~~~~~~~~~~~~~~~~~~~~~~~l~~~l~~~~--~~l~~~~~~~~~~~~~~~~~~l   210 (420)
T 1JND_A          134    PKNKPRKVHGDLGLAWKSIKKLFTGDFIVDPHAALHKEQFTALVRDVKDSLRADG---FLLSLTVLPNVNSTWYFDIPAL   210 (420)
T ss_dssp                CCCCCCCC-------------------CCCTTHHHHHHHHHHHHHHHHHHHHHTTT---CEEEEECTTCCHHHHCCHHHH
T ss_pred                CCCCCCccccCccchHHHHHHHHHhcCCCcccCCCChhhcHHHHHHHHHHHHHhhcCC---cEEEEEeCCCCccccCCCCCCHHHH


Q ss_pred                ecceeeeeecCCCCCcccceeEEEEeeccccCC---CCCCCCChHHHhc-------------HHHHHHHhcCCCCCCCCCc
Q Q_8363653_2      217    HNYKLFAESTDKNGNPLLDEVQIMTYDFSWSGS---APGPSTPLWWMRN-------------VAEWVKQCFDPSVNPNAK   280 (999)
```

```
Q Consensus      217   hnyklfaestdkngnplldevqimtydfswsgs---apgpstplwwmrn-------------vaewvkqcfdpsvnpnak   280 (999)
                       ..+              +|-+.+|+|++.....   ..++..|+++...             +..|+.+.+++
T Consensus      211   ~~~--------------vD~~~i~~y~~~~~~~~~~~~~~~~~~~~~~~~~~~~~~~~~~~~~~~~~~~~~~~~~~~~~~gi~~-------   269 (420)
T 1JND_A         211   NGL--------------VDFVNLATFDFLTPARNPEEADYSAPIYHPDGSKDRLAHLNADFQVEYWLSQGFPS-------   269 (420)
T ss_dssp              HTT--------------CSEEEECCCCSSCTTTCTTCBCCSSCSSCCTTSTTCCTTCSHHHHHHHHHHTTCCG-------
T ss_pred              HHh--------------CCEEEEEecccCCCCCCCCccCCCCcCCCCCCCCcchhHhccHHHHHHHHHHCCCCH-------


Q ss_pred              ceEeeEEeCCCccccCCCCCCCCC---------------------CCceeehhHhccccc
Q Q_8363653_2     281   CTIDNVYLGGAGYGRRWPIHSDDN---------------------WGSTVTYRDLVDWQN   319 (999)
Q Consensus      281   ctidnvylggagygrrwpihsddn---------------------wgstvtyrdlvdwqn   319 (999)
                          +.+.+|-..||++|......+                     -....+|+++.....
T Consensus      270   ---~Kl~lgi~~yg~~~~~~~~~~~~~~~~~~~~~~~~~~~~~~~~~~~~~~~~~~~~~~~~~~~~~~~~~~~~ei~~~~~   326 (420)
T 1JND_A         270   ---NKINLGVATYGNAWKLTKDSGLEGVPVVPETSGPAPEGFQSQKPGLLSYAEICGKLS   326 (420)
T ss_dssp              ---GGEEEEEESEEEEEECCGGGCSCCSSCBCSCCSBCCCCTTTCCTTEEEHHHHHHHBC
T ss_pred              ---HHEEEEeeeceeEEEecCCCCCCCCccccCCCCCCCCCcccCCCceEeHHHHhhhcc
```

Template alignment | Template 3D Structure | PDBe

19. 4P8V_A Chitinase-3-like protein 2; Chitinase 3-like protein 2, human YKL-39, Family-18 chitinase, SUGAR BINDING PROTEIN; HET: SO4, NAG; 1.64A {Homo sapiens}

Probability: 97.21%, E-value: 0.0017, Score: 58.46, Aligned cols: 184, Identities: 16%, Similarity: 0.299, Template Neff: 11.6

```
Q ss_pred              HhhhhhcCccccceeeEeeee--CHHHHHHHhccchhhhHHHHHhHHHHHHHHhccCCCCccceeeeEEecccccccccc
Q Q_8363653_2      92   IEKDMKRWPHIEYYMQFVIF--GPETVSRLLDSQTAQDNFINNLKTVVARFRKDQNGNDLGYTGIEIDCEGSFSDSKWDT   169 (999)
Q Consensus       92   iekdmkrwphieyymqfvif--gpetvsrlldsqtaqdnfinnlktvvarfrkdqngndlgytgieidcegsfsdskwdt   169 (999)
                       |.+-.++.|.+..++-+--.  +.+..+.++.+.....+|++++..++..+         ++.||.||.|..
T Consensus       66   i~~~~~~~~~~~~~v~~sigg~~~~~~~~~~~~~~~~~~~~~~~~~~~~~~~~~~~~~~~~~~~~i~~~~~~~~~---------~~dGidld~e~~--------   128 (371)
T 4P8V_A          66   INSLKTKNPKLKILLSIGGYLFGSKGFHPMVDSSTSRLEFINSIILFLRNH---------NFDGLDVSWIYP--------   128 (371)
T ss_dssp              HHHHHHHCTTCEEEEEEEETTTTTGGGTTGGGSHHHHHHHHHHHHHHHHHT---------TCCEEEEEESCC--------
T ss_pred              HHHHHHHCCCCeEEEEECCCccCccccHHHhcCHHHHHHHHHHHHHHHHHC---------CCCCeeeCCCCC--------


Q ss_pred              CCCCcchhHcHHHHhcceeeee------CCCCcceEEeehhccCCCCCchhheecceeeeeecCCCCCcccceeEEEEee
Q Q_8363653_2     170   RAGDDVKYINLLKRIKNEVIID------ANPDFKLRINAHAMWGDGIPDYYRFHNYKLFAESTDKNGNPLLDEVQIMTYD   243 (999)
Q Consensus      170   ragddvkyinllkrikneviid------anpdfklrinahamwgdgipdyyrfhnyklfaestdkngnplldevqimtyd   243 (999)
                       ...+...|..+++++++..--.      .+-.+.+.+.+.....+..+.-.+                ...+|.+.+|+|+
T Consensus      129   ~~~~~~~~~~~~~~~~l~~~~~~~~~~~~~~~~~~~~~~l~~~~~~~~~~~~~~~~~~~~~~~~~~~~~~~~--------------~~~~D~~~~~~~y~   194 (371)
T 4P8V_A         129   DQKENTHFTVLIHELAEAFQKDFTKSTKERLLLTVGVSAGRQMIDNSYQVEKL--------------AKDLDFINLLSFD   194 (371)
T ss_dssp              HHHHHHHHHHHHHHHHHHHHHHHHTCSSCCCEEEEEEECCHHHHHHHCCHHHH--------------HHHCSEEEEECCC
T ss_pred              CCCCCCcHHHHHHHHHHHHhHhccccCcceeEEEEEEeCCHHHHhcCCCHHHH--------------HHhcCEeEeeccc


Q ss_pred              ccccCCCC----------------CCCCChHHHhcHHHHHHHhcCCCCCCCCcceEeeEEeCCCccccCCC---------
Q Q_8363653_2     244   FSWSGSAP----------------GPSTPLWWMRNVAEWVKQCFDPSVNPNAKCTIDNVYLGGAGYGRRWP---------   298 (999)
Q Consensus      244   fswsgsap----------------gpstplwwmrnvaewvkqcfdpsvnpnakctidnvylggagygrrwp---------   298 (999)
                       +......|                +++.+.++...+..|+++.+.+          ..+.+|-..||+.|.
T Consensus      195   ~~~~~~~~~~~~~~~~~~~~~~l~~~~~~~~~~~~~~~~~~~~~~~~~~~~~~~~~~~~~~~g~~~----------~kl~~g~~~~~~~~~~~~~~~~~~~~~~~~   264 (371)
T 4P8V_A         195   FHGSWEKPLITGHNSPLSKGWQDRGPSSYYNVEYAVGYWIHKGMPS----------EKVVMGIPTYGHSFTLASAETTVG   264 (371)
T ss_dssp              SSCTTCSSCBCCCSSCSSCCTTCCGGGGGCSHHHHHHHHHHTTCCG----------GGEEEEEESEEEEEEESSSCCSTT
T ss_pred              ccCCCCCCccccCCCCCCCCCCCCCCCccccHHHHHHHHHHcCCCH----------HHEEEEEecceEEEEeCCCCCCCC


Q ss_pred              --------CCCCCCCCceeehhHhcc
Q Q_8363653_2     299   --------IHSDDNWGSTVTYRDLVD   316 (999)
Q Consensus      299   --------ihsddnwgstvtyrdlvd   316 (999)
                               ..........++|+++..
T Consensus      265   ~~~~~~~~~~~~~~~~~~~~~~~~~~~~~~~~~~~~~~~~~~~~~~~~   290 (371)
T 4P8V_A         265   APASGPGAAGPITESSGFLAYYEICQ   290 (371)
T ss_dssp              CBEEEECCCCTTTCCTTEEEHHHHHH
T ss_pred              CCCCCCCCCCccccCCceeeHHHHHH
```

Template alignment | Template 3D Structure | PDBe

20. 1NAR_A NARBONIN; PLANT SEED PROTEIN; 1.8A {Vicia narbonensis} SCOP: c.1.8.5

Probability: 97.14%, E-value: 0.0045, Score: 56.21, Aligned cols: 193, Identities: 17%, Similarity: 0.219, Template Neff: 10.5

```
Q ss_pred              EEEEEecCCCC----CCCcccccccCccccccCCCCCccCcchHhhhhhcCccccceeeEeeeeCHHHHHHHhccchhhhHH
Q Q_8363653_2      54   IAIYRLGPQKD----AQGSFTSFTDATTYDRYAPGRTLWPNYIEKDMKRWPHIEYYMQFVIFGPETVSRLLDSQTAQDNF   129 (999)
Q Consensus       54   iaiyrlgpqkd----aqgsftsftdattydryapgrtlwpnyiekdmkrwphieyymqfvifgpetvsrlldsqtaqdnf   129 (999)
                       +.+....+..+    ..+.+..+.+.....         ...+..-.++.|++...+.+--.+.......+.+....++|
T Consensus       35   vi~~f~~~~~~~~~~~~~~~~~~~~~~~~~~~~~~~~~~~~~~~~~~~~~~~~~~~~~~~~~~---------~~~~~~~~lk~~~~~~~~~kvllsigg~~~~~~~~~~~~~~~~~~~~~~~~~~~~~~~~~~~~~~~   105 (290)
T 1NAR_A          35   YILGFAIESYYESGKGTGTFEESWDVELFG---------PEKVKNLKRRHPEVKVVISIGGRGVNTPFDPAEENVWVSNA   105 (290)
T ss_dssp              EEEEEEEEEECTTSCEEEEEEEECSCHHHHS---------HHHHHHHHHHCTTCEEEEEEEEESSTTSCBCSCHHHHHHHH
T ss_pred              EEEEEeeeccccCCCceeEEeceechhhCC---------HHHHHHHHHHCCCCEEEEEecCCCCCCCCChhcCHHHHHHHH


Q ss_pred              HHhHHHHHHHHhhccCCCCccc--eeeeEEecccccccccccCCCCcchhHcHHHHhcceeeeeCCCCcceEEeehhccC
Q Q_8363653_2     130   INNLKTVVARFRKDQNGNDLG--YTGIEIDCEGSFSDSKWDTRAGDDVKYINLLKRIKNEVIIDANPDFKLRINAHAMWG   207 (999)
Q Consensus      130   innlktvvarfrkdqngndlg--ytgieidcegsfsdskwdtragddvkyinllkrikneviidanpdfklrinahamwg   207 (999)
                       ++++..++.++.     ...|  +.||+||.|....+..          +..++++++.+  ........+.+..-+...
T Consensus      106   ~~~~~~~~~~~~~~~~~~~-----~~~~~~~~~~dGidld~e~~~~~~~~~----------~~~~l~~l~~~--~~~~~~~~~~~~~~~~~~~~~~~~~~~~~~~~   168 (290)
T 1NAR_A         106   KESLKLIIQKYS-----DDSGNLIDGIDIHYEHIRSDEP----------FATLMGQLITE--LKKDDDLNINVVSIAPSE   168 (290)
T ss_dssp              HHHHHHHHHHSE-----ETTEECCCEEEEEESCBCSSTT----------HHHHHHHHHHH--HHHCTTSCCCEEEECCCT
T ss_pred              HHHHHHHHHHHh-----ccCCCCccEEEEeCCCCCCCHH----------HHHHHHHHHHH--hccCCCCeEEEEeeCCCC


Q ss_pred              CCCCchhheecceeeeeecCCCCCccccceeEEEEeeccccCCCCCCCCChHHHhcHHHHHHHhcCCCCCCCCcceEeeEE
Q Q_8363653_2     208   DGIPDYYRFHNYKLFAESTDKNGNPLLDEVQIMTYDFSWSGSAPGPSTPLWWMRNVAEWVKQCFDPSVNPNAKCTIDNVY   287 (999)
```

```
Q Consensus      208   dgipdyyrfhnyklfaestdkngnplldevqimtydfswsgsapgpstplwwmrnvaewvkqcfdpsvnpnakctidnvy   287 (999)
                       .....|...              ....+|.+.+|+||+.++   +++.....|....-.+.++.++|+          .+.
T Consensus      169   ~~~~~~~~~--------------~~~~~d~v~~~~y~~~~~~---~~~~~~~~~~~~~~~~~~~~~~~~~----------kl~   222 (290)
T 1NAR_A         169   NNSSHYQKL-------------YNAKKDYINWVDYQFSNQ---QKPVSTDDAFVEIFKSLEKDYHPH----------KVL   222 (290)
T ss_dssp              TTHHHHHHH-------------HHHHTTTCCEEEEEGGGC---SSCCCSHHHHHHHHHHHHHHSCTT----------CEE
T ss_pred              CCChhhHHH-------------HHhhCCEEEEEecccCCC---CCCCCCHHHHHHHHHHHHhcCCCHH----------HEE


Q ss_pred              eCCCccccC--CC
Q Q_8363653_2      288   LGGAGYGRR--WP   298 (999)
Q Consensus      288   lggagygrr--wp   298 (999)
                       +|-..||+.  |.
T Consensus      223   ~g~~~~~~~~~~~~   235 (290)
T 1NAR_A         223   PGFSTDPLDTKHN   235 (290)
T ss_dssp              EEEECCHHHHHHC
T ss_pred              EEEECCCCCCCcC
```

Template alignment | Template 3D Structure | PDBe

21. 5Z05_A Chitinase-3-like protein 1; SIGNALING PROTEIN; HET: NAG; 1.49A {Bubalus bubalis}

Probability: 97.1%, E-value: 0.0039, Score: 55.47, Aligned cols: 184, Identities: 15%, Similarity: 0.255, Template Neff: 11.8

```
Q ss_pred              chHhhhhhcCccccceeeEeeeeC--HHHHHHHhcccchhhhHHHHhHHHHHHHHHhccCCCCccceeeeEEeccccccccc
Q Q_8363653_2      90   NYIEKDMKRWPHIEYYMQFVIFG--PETVSRLLDSQTAQDNFINNLKTVVARFRKDQNGNDLGYTGIEIDCEGSFSDSKW   167 (999)
Q Consensus       90   nyiekdmkrwphieyymqfvifg--petvsrlldsqtaqdnfinnlktvvarfrkdqngndlgytgieidcegsfsdskw   167 (999)
                       ..|.+--++-|++..++.+-...   .+..+.++.+...++.|++++..++.++          ++.||.||+|..-.+
T Consensus       57   ~~i~~~~~~~~~~~~~~~~~sig~~~~~~~~~~~~~~~~~~~~~~~~~~~~l~~~~~~~~~~----------~~dGi~id~e~~~~~~~---   124 (361)
T 5Z05_A         57   DTLNTLKNRNPKLKTLLSVGGWNFGSQRFSKIASKTQSRRTFIKSVPPFLRTH----------GFDGLDLAWLYPGWR---   124 (361)
T ss_dssp              HHHHHGGGTCTTCEEEEEEECTTSCHHHHHHHTSHHHHHHHHHHHHHHHHH----------TCSEEEEECSCCCGG---
T ss_pred              HHHHHHHHHCCCCcEEEEeCCcccCChhhHHHhcCHHHHHHHHHHHHHHHHHh----------CCCcEEEccCCCCCC---


Q ss_pred              ccCCCCcchhHcHHHHhcceeee-----eCCCCcceEEeehhccCCCCCchhheecceeeeeeCCCCCCccccceeEEEEe
Q Q_8363653_2     168   DTRAGDDVKYINLLKRIKNEVII-----DANPDFKLRINAHAMWGDGIPDYYRFHNYKLFAESTDKNGNPLLDEVQIMTY   242 (999)
Q Consensus      168   dtragddvkyinllkriknevii-----danpdfklrinahamwgdgipdyyrfhnyklfaestdkngnplldevqimty   242 (999)
                            +.-.+..++++++....-      ..+-...+.+.+...+.+...+.-...++               +|.+-+|+|
T Consensus      125   -----~~~~~~~~~~~~l~~~l~~~~~~~~~~~~~~~l~~~~~~~~~~~~~~~~~~~~~~~----------------~~d~~~~~y   185 (361)
T 5Z05_A        125   -----DKRHLTTLVKEMKAEFVREAQAGTEQLLLSAAVPAGKIAIDRGYDIAQISRH--------------LDFISLLTY   185 (361)
T ss_dssp              -----GHHHHHHHHHHHHHHHHHHHHHHTTSSCCEEEEEEECCHHHHHHHCCHHHHHHH--------------CSEEEEETT
T ss_pred              -----ccccHHHHHHHHHHHHHHHHHhCCCeEEEEEEecCChHHHHcCCCHHHHHHh--------------CCEEEEecC


Q ss_pred              eccc-CCCCCCCCCh-----------HHHhcHHHHHHHh-cCCCCCCCCCcceEeeEEeCCCcccccCCCCCCC-------
Q Q_8363653_2     243   DFSWS-GSAPGPSTPL-----------WWMRNVAEWVKQC-FDPSVNPNAKCTIDNVYLGGAGYGRRWPIHSD-------   302 (999)
Q Consensus      243   dfsws-gsapgpstpl-----------wwmrnvaewvkqc-fdpsvnpnakctidnvylggagygrrwpihsd-------   302 (999)
                       ++.+. ...+++..|+           .++....++....  ++++            .+.+|-.-||++|+..+.
T Consensus      186   ~~~~~~~~~~~~~~~~~~~~~~~~~~~~~~~~~~~~~~~~~~g~~~----------kl~~gi~~~g~~~~~~~~~~~~~~~~~~~   255 (361)
T 5Z05_A        186   DFHGAWRQTVGHHSPLFRGQEDASSRFSNADYAVSYMLRLGAPAN----------KLVMGIPTFGKSYTLASSKTDVGAP   255 (361)
T ss_dssp              CSCCTTSSBCCCSSCSSCCCSSCCCTTSSHHHHHHHHHHTTCCGG----------GEEEEEESSEEEEESSSCCSTTCB
T ss_pred              CCCCccccccCCCCCCCCCCCCCcccccccHHHHHHHHHHcCCCHH----------HEEEEeecccceEEecCCCCCCCCC


Q ss_pred              ----------CCCCceeehhHh
Q Q_8363653_2     303   ----------DNWGSTVTYRDL   314 (999)
Q Consensus      303   ----------dnwgstvtyrdl   314 (999)
                                 ......++|.++
T Consensus      256   ~~~~~~~~~~~~~~~~~~y~~~   277 (361)
T 5Z05_A        256   ISGPGIPGQFTKEKGILAYYEI   277 (361)
T ss_dssp              EEEECCCCTTTCCTTEECHHHH
T ss_pred              CCCCCCCcccccccceeeHHHH
```

Template alignment | Template 3D Structure | PDBe

22. 4WKA_A Chitotriosidase-1; CHIT1, GH18 chitinase, protonation states, hydrolysis, catalytic mechanism, hydrolase; HET: TLA; 0.95A {Homo sapiens}

Probability: 97.09%, E-value: 0.002, Score: 58.03, Aligned cols: 204, Identities: 20%, Similarity: 0.314, Template Neff: 11.6

```
Q ss_pred              HhhhhhcCccccceeeEeeee--CHHHHHHHhcccchhhhHHHHhHHHHHHHHHhccCCCCccceeeeEEecccccccccccc
Q Q_8363653_2      92   IEKDMKRWPHIEYYMQFVIF--GPETVSRLLDSQTAQDNFINNLKTVVARFRKDQNGNDLGYTGIEIDCEGSFSDSKWDT   169 (999)
Q Consensus       92   iekdmkrwphieyymqfvif--gpetvsrlldsqtaqdnfinnlktvvarfrkdqngndlgytgieidcegsfsdskwdt   169 (999)
                       +..-.++.|++..+.+--.  +.+....++.+.+.+.+|+.++..++.+          ++.||+||.|......   .
T Consensus       59   i~~l~~~~~~kv~~sv~g~~~~~~~~~~~~~~~~~~~~~~~~~~~~~~~i~~~~~~~~~~~~~~~~----------~~DGidid~e~~~~~~~---~   126 (377)
T 4WKA_A         59   FNGLKKMNPKLKTLLAIGGWNFGTQKFTDMVATANNRQTFVNSAIRFLRKY----------SFDGLDLDWEYPGSQG---S   126 (377)
T ss_dssp              HHHHHHHCTTCEEEEEECTTTCSHHHHHHHTSHHHHHHHHHHHHHHHHHH----------TCSEEEEECSCTTSTT---C
T ss_pred              HHHHHHHCCCCeEEEEECCCccCCccCCchHHHhcCHHHHHHHHHHHHHHHh----------CCCCeeCCCCCCCCCC---C


Q ss_pred              CCCCcchhHcHHHHhcceeeeeCCCCcceEEeehhccCCCCCchhheecceeeeeeCCCCCCccccceeEEEEeecccc--
Q Q_8363653_2     170   RAGDDVKYINLLKRIKNEVIIDANPDFKLRINAHAMWGDGIPDYYRFHNYKLFAESTDKNGNPLLDEVQIMTYDFSWS--   247 (999)
Q Consensus      170   ragddvkyinllkrikneviidanpdfklrinahamwgdgipdyyrfhnyklfaestdkngnplldevqimtydfsws--   247 (999)
                       ...+.-.++.++++....--......+-.+.........+..-+.+.+....         ...+|-+.+|+|++...
T Consensus      127   ~~~~~~~~~~~~l~~~l~~~l~~~~~~~~~~~~~~~~~~~~~~~~~~~~~~~~~~~~~~--------~~~~D~~~~~y~~~~~~~~~   198 (377)
T 4WKA_A        127   PAVDKERFTTLVQDLANAFQQEAQTSGKERLLLSAAVPAGQTYVDAGYEVDKI--------AQNLDFVNLMAYDFHGSWE   198 (377)
T ss_dssp              CTTHHHHHHHHHHHHHHHHHHHHHHHHCSCCCEEEEEEECSHHHHHHCCHHHH--------HTTCSEEEEECCCSSCTTS
T ss_pred              CcccHHHHHHHHHHHHHHHHHHHHHHhcCCceEEEEEecCChHHHhcCCCHHHH--------HHhCCEEEEEccccccCCcc


Q ss_pred              ------------CCCCCCCChHHHhcHHHHHHHhcCCCCCCCCCcceEeeEEeCCCcccccCCCCCCC------------
Q Q_8363653_2     248   ------------GSAPGPSTPLWWMRNVAEWVKQCFDPSVNPNAKCTIDNVYLGGAGYGRRWPIHSD------------   302 (999)
```

```
Q Consensus     248   ------------gsapgpstplwwmrnvaewvkqcfdpsvnpnakctidnvylggagygrrwpihsd-------------   302 (999)
                                  ...+++..+.-|...+..|+++.++++          .+.+|-.-||+.|.....
T Consensus     199   ~~~~~~~~~~~~~~~~~~~~~~~~~~~~~~~~~~~~~~~----------ki~~gi~~~g~~~~~~~~~~~~~~~~~~~~~   268 (377)
T 4WKA_A        199   KVTGHNSPLYKRQEESGAAASLNVDAAVQQWLQKGTPAS----------KLILGMPTYGRSFTLASSSDTRVGAPATGSG   268 (377)
T ss_dssp            SBCCCSSCSSCCTTCCGGGGGCSHHHHHHHHHHTTCCGG----------GEEEEEESEEEEEEESCTTCCSTTCBEEEEC
T ss_pred            cccCCCCCCCCCcccCCccccCCHHHHHHHHHHcCCCHH----------HEEEEEecceeeEEccCCCCCCCCCCCcCCC


Q ss_pred            -----CCCCceeehhHhccccccEEEEe
Q Q_8363653_2   303   -----DNWGSTVTYRDLVDWQNGYLIHH   325 (999)
Q Consensus     303   -----dnwgstvtyrdlvdwqngylihh   325 (999)
                           .......+|.++..+........
T Consensus     269   ~~~~~~~~~~~~~~~~~~~~~~~~~~~~   296 (377)
T 4WKA_A        269   TPGPFTKEGGMLAYYEVCSWKGATKQRI   296 (377)
T ss_dssp            CCCTTTCCTTEEEHHHHTTCTTCEEEEE
T ss_pred            CCCCCCcccCceEeheehhcccCCceeee
```

Template alignment | Template 3D Structure | PDBe

23. 3W4R_A Chitinase; insect, glycosyl hydrolase, chitin, HYDROLASE; HET: NAG; 1.7A {Ostrinia furnacalis}

Probability: 97.07%, E-value: 0.0023, Score: 63.35, Aligned cols: 180, Identities: 15%, Similarity: 0.321, Template Neff: 10.5

```
Q ss_pred            HhhhhhcCcccceeeEeeee--CHHHHHHHhccchhhhHHHHhHHHHHHHHhhccCCCCccceeeeEEeccccccccccc
Q Q_8363653_2    92   IEKDMKRWPHIEYYMQFVIF--GPETVSRLLDSQTAQDNFINNLKTVVARFRKDQNGNDLGYTGIEIDCEGSFSDSKWDT   169 (999)
Q Consensus      92   iekdmkrwphieyymqfvif--gpetvsrlldsqtaqdnfinnlktvvarfrkdqngndlgytgieidcegsfsdskwdt   169 (999)
                      +.+-.++.|++...+.+--.  +.+..+.++.+.....+|++++..++.++.         +.||+||.|.. .......
T Consensus      88   ~~~lk~~~p~~kvllsigg~~~~~~~~~~~~~~~~~~~~~r~~f~~~i~~~~~~~~~~~---------fDGidiD~E~~-~~~~~~~~   157 (554)
T 3W4R_A        88   FTSLRSKHPGVKFTVAVGGWAEGGSKYSKMVAAKSTRMAFVRSVVDFLNKYN---------FDGLDLDWEYP-GAADRGG   157 (554)
T ss_dssp            HHGGGGTCTTSEEEEEEECSTTCSHHHHHHHTSHHHHHHHHHHHHHHHHHHHT---------CSEEEEECSCT-TCGGGTC
T ss_pred            HHHHHHHCCCCEEEEEECCcccchHHHHHHcCHHHHHHHHHHHHHHHHHHHcC---------CCcEEEcccCC-CCccCCC


Q ss_pred            CCCCcchhHcHHHHhcceeeeeCCCCcceEEeehhccCCCCCchhheecceeeeeecCCCCCcccceeEEEEeecccc-C
Q Q_8363653_2   170   RAGDDVKYINLLKRIKNEVIIDANPDFKLRINAHAMWGDGIPDYYRFHNYKLFAESTDKNGNPLLDEVQIMTYDFSWS-G   248 (999)
Q Consensus     170   ragddvkyinllkrikneviidanpdfklrinahamwgdgipdyyrfhnyklfaestdkngnplldevqimtydfsws-g   248 (999)
                      ...|...|+.+++.++++.--. ++.+.|-+...+........|.-            +.-.+.+|-+.||+||+.+. .
T Consensus     158   ~~~~~~~~~~~~~l~~lr~~l~~~-~~~~~lt~a~~~~~~~~~~~~~~~~~~~------------~~~~~~~D~i~vm~yd~~~~~~   224 (554)
T 3W4R_A       158   SFSDKDKFLYLVQELRRAFIRE-GKGWELTAAVPLANFRLMEGYHV------------PELCQELDAIHVMSYDLRGNWA   224 (554)
T ss_dssp            CTTHHHHHHHHHHHHHHHHHHH-TSCCEEEEEECCCHHHHHHHCCH------------HHHHHHCSEEEECCCCSSCGGG
T ss_pred            ChhHHHHHHHHHHHHHHHHHHh-CCceEEEEEeeccccchhhcCCCH------------HHHHHHCCEEEEEecccCCCCc


Q ss_pred            CCCCCCCChH-------------HHhcHHHHHHHhcCCCCCCCCcceEeeEEeCCCccccCCCCCCCCC
Q Q_8363653_2   249   SAPGPSTPLW-------------WMRNVAEWVKQCFDPSVNPNAKCTIDNVYLGGAGYGRRWPIHSDDN   304 (999)
Q Consensus     249   sapgpstplw-------------wmrnvaewvkqcfdpsvnpnakctidnvylggagygrrwpihsddn   304 (999)
                      ...++..|++             ....+..|+++.+++          ..+.+|-..|||.|..-+..+
T Consensus     225   ~~~~~~~~l~~~~~~~~~~~~~~~~~~~~~~~~~~~g~~~----------~KlvlG~p~yg~~~~~~~~~~~   283 (554)
T 3W4R_A       225   GFADVHSPLYKRPHDQWAYEKLNVNDGLQLWEDKGCPT----------NKLVVGIPFYGRSFTLSSGNN   283 (554)
T ss_dssp            TBCCCSSCSSCCTTCCGGGTTCSHHHHHHHHHHTTCCG----------GGEEEEEESEEEEEEBCTTCC
T ss_pred            CCCCCCCCccCCCCcchHhhccCHHHHHHHHHHcCCCH----------HHEEEEEeecceeeeccCCCC
```

Template alignment | Template 3D Structure | PDBe

24. 5JH8_A Probable chitinase; HYDROLASE; HET: HIS, 6KY, MLY, MLZ, MSE, EDO; 1.018A {Chromobacterium violaceum}

Probability: 97.03%, E-value: 0.0031, Score: 55.76, Aligned cols: 155, Identities: 21%, Similarity: 0.376, Template Neff: 11.5

```
Q ss_pred            hccchhhhHHHHhHHHHHHHHhhccCCCCccceeeeEEeccccccccccccCCCCcchhHcHHHHhcceeeeeCCCCcceE
Q Q_8363653_2   120   LDSQTAQDNFINNLKTVVARFRKDQNGNDLGYTGIEIDCEGSFSDSKWDTRAGDDVKYINLLKRIKNEVIIDANPDFKLR   199 (999)
Q Consensus     120   ldsqtaqdnfinnlktvvarfrkdqngndlgytgieidcegsfsdskwdtragddvkyinllkrikneviidanpdfklr   199 (999)
                      +.+....+.|++++..++..+         ++.||+||.|..-        ..+...++.++++++...--. +..+.+.
T Consensus      83   ~~~~~~~~~~~~~~~~~i~~~~~~~~~---------~~dGv~id~e~~~--------~~~~~~~~~~~~~~~~~~l~~~~~~~~~-~~~~~~~~   144 (317)
T 5JH8_A        83   AVSTSAQSQAVANLVKFAQDK---------RFSGINVDFEAVA--------QGDRNNFSHFIQVLGRALHAK-GLKLIVS   144 (317)
T ss_dssp            HHHTTTHHHHHHHHHHHHHHHH---------TCSEEEEECCSCC--------GGGHHHHHHHHHHHHHHHHHHHTT-TCEEEEE
T ss_pred            hcChHHHHHHHHHHHHHHHHHC---------CCCeEEEEcccCC--------hHHHHHHHHHHHHHHHHHHHHHHC-CCEEEEE


Q ss_pred            EeehhccCCCCCchhheecceeeeeecCCCCCcccceeEEEEeeccccCCCCCCCCCChHHhcHHHHHHHhcCCCCCCCC
Q Q_8363653_2   200   INAHAMWGDGIPDYYRFHNYKLFAESTDKNGNPLLDEVQIMTYDFSWSGSAPGPSTPLWWMRNVAEWVKQCFDPSVNPNA   279 (999)
Q Consensus     200   inahamwgdgipdyyrfhnyklfaestdkngnplldevqimtydfswsgsapgpstplwwmrnvaewvkqcfdpsvnpna   279 (999)
                      +.........+....+.--.+          ...+|-+.+|+|++..+...|.|..+..|++.+.+++.+.+++
T Consensus     145   ~~~~~~~~~~~~~~~~~~~~~~~~~~~---------~~~~d~~~~~~~y~~~~~~~~~~~~~~~~~~~~~~~~~~~~~~~~~~~~~~~~~~~------   209 (317)
T 5JH8_A       145   VPAFSAKDENHPANYGYDLRAL---------GAAADYLQIMSYDEAIPAWDPGPVAGSDWMEDDLDYAVERVPA------   209 (317)
T ss_dssp            EECCCGGGTTSTTTTTCCHHHH---------HHHSSEEEEECCCSSCTTSCCCCSSCHHHHHHHHHHHHHHSCG------
T ss_pred            EccccCCCCCCCCcccCCHHHH---------HHHCCEEEEEecCCCCCCCCCCCCCCChHHHHHHHHHHHHHCCH------


Q ss_pred            cceEeeEEeCCCccccCCCCCCCCCCCceeehhHh
Q Q_8363653_2   280   KCTIDNVYLGGAGYGRRWPIHSDDNWGSTVTYRDL   314 (999)
Q Consensus     280   kctidnvylggagygrrwpihsddnwgstvtyrdl   314 (999)
                          ..+.+|-.-||+.|+.-++   +..++|.+.
T Consensus     210   ----~kl~~g~~~~g~~~~~~~~~---~~~~~~~~~~   237 (317)
T 5JH8_A       210   ----AKILNGIPAYGYDWKRPGD---GGMLYWKDT   237 (317)
T ss_dssp            ----GGEEEEEESEEEEEEETTE---EEEEEGGGH
T ss_pred            ----HHEEEEEeccccccCCCC---CCeeeHHHH
```

Template alignment | Template 3D Structure | PDBe

25. 1LL7_B CHITINASE 1; BETA-ALPHA BARREL, HYDROLASE; 2.0A {Coccidioides immitis} SCOP: c.1.8.5, d.26.3.1

Probability: 96.96%, E-value: 0.01, Score: 54.81, Aligned cols: 224, Identities: 17%, Similarity: 0.231, Template Neff: 11.2

```
Q ss_pred                    ceeeEEEeeecccCCCccEEEEEecCCCCCCCcccccccCccccCCCCCccCcchHhhhhhcCccccceeeEeeeeC-HH
Q Q_8363653_2         36     NKITSIGFHEFNVDAQGRIAIYRLGPQKDAQGSFTSFTDATTYDRYAPGRTLWPNYIEKDMKRWPHIEYYMQFVIFG-PE   114 (999)
Q Consensus           36     nkitsigfhefnvdaqgriaiyrlgpqkdaqgsftsftdattydryapgrtlwpnyiekdmkrwphieyymqfvifg-pe   114 (999)
                             +.++.|-+.-+.++..|.+.+       .+........-....++.-......-.-+-++...++-|.+...+.+--.. ..
T Consensus           27     ~~~t~l~~~~~~~~~~~g~~~~------~~~~~~~~~~~~~~~~~~~~~~~~~~~~~~~~~~~~~~~~~~~~~kv~~~~~g~~~~~   100 (392)
T 1LL7_B              27     DQFTHILYAFANIRPSGEVYL------SDTWADTDKHYPGDKWDEPGNNVYGCIKQMYLLKKNNRNLKTLLSIGGWTYSP   100 (392)
T ss_dssp                     GGCSEEEEEEEEECTTSCEEE------SCHCCCCCCCCTTCCSSCSSCCCCHHHHHHHHHHHHCTTCEEEEEEEHHHHGG
T ss_pred                     HHCCEEEEEeeeeCCCCcEEE------cCccccccCCCCCCCCCCCCCCchhhhHHHHHHHHHhCCCCeEEEEECCcccCc

Q ss_pred                    HHHHHhcccchhhhHHHHhHHHHHHHHhhccCCCCcccceeeeEEeccccccccccccCCCCccchhHcHHHHhcceeee----
Q Q_8363653_2         115    TVSRLLDSQTAQDNFINNLKTVVARFRKDQNGNDLGYTGIEIDCEGSFSDSKWDTRAGDDVKYINLLKRIKNEVII----   190 (999)
Q Consensus           115    tvsrlldsqtaqdnfinnlktvvarfrkdqngndlgytgieidcegsfsdskwdtragddvkyinllkriknevii----   190 (999)
                             ..+.++.....+..|++++...+.+.            ++.||.||.|..-+          ..+...+..++++++...--
T Consensus           101    ~~~~~~~~~~~~~~~~~~~i~~~~~~~~~----------~~dGi~ld~e~~~~~-------~~~~~~~~~~~~~~~l~~l~~~l~~~~~~~   164 (392)
T 1LL7_B              101    NFKTPASTEEGRKKFADTSLKLMKDL---------GFDGIDIDWQYPED-------EKQANDFVLLLKACREALDAYSAK   164 (392)
T ss_dssp                     GSHHHHTSHHHHHHHHHHHHHHHHHHH---------TCSEEEEECSCCCS-------HHHHHHHHHHHHHHHHHHHHHHHHHT
T ss_pred                     cchhhcCCHHHHHHHHHHHHHHHHHHh---------CCCcEEEeeeCCCC-------HHhHHHHHHHHHHHHHHHHHHHHHhhc

Q ss_pred                    -eCCCCcceEEeehhccCCCCCchhheecceeeeeecCCCCCcccceeEEEEeeccc------------cCCCCCCCCC
Q Q_8363653_2         191    -DANPDFKLRINAHAMWGDGIPDYYRFHNYKLFAESTDKNGNPLLDEVQIMTYDFSW------------SGSAPGPSTP   256 (999)
Q Consensus           191    -danpdfklrinahamwgdgipdyyrfhnyklfaestdkngnplldevqimtydfsw------------sgsapgpstp   256 (999)
                              ..++.+.+.+.+...+     .+..+...+++..+        ..+|.+.+|+|++.+              +.+...+..+
T Consensus           165    ~~~~~~~~~~l~~~~~~~~~-----~~~~~~~~~~~~~~~~~l~~---------~~vD~v~i~~y~~~~~~~~~~~~~~~~~~~~~~~~l~~~~~~~~~~~~~~~   231 (392)
T 1LL7_B              165    HPNGKKFLLTIASPA-----GPQNYNKLKLAEMD--------KYLDFWNLMAYDFSGSWDKVSGHMSNVFPSTTKPESTP   231 (392)
T ss_dssp                     STTSCCCEEEEEEEEC-----SHHHHTTSCHHHHH--------TTCSEEEEECCCSSSTTSSBCCCSSCSSCCSSCGGGCS
T ss_pred                     CCCCCeEEEEEEEcCC-----ChhhhhccCHHHHH--------HhCCEEEEecCCCCCCCcccccCCCCCCCCCCCCCCCCC

Q ss_pred                    hHHHHhcHHHHHHHHhcCCCCCCCCCcceEeeEEeCCCcccccCCCCCCCCCC
Q Q_8363653_2         257    LWWMRNVAEWVKQCFDPSVNPNAKCTIDNVYLGGAGYGRRWPIHSDDN   304 (999)
Q Consensus           257    lwwmrnvaewvkqcfdpsvnpnakctidnvylggagygrrwpihsddn   304 (999)
                             .|+...+..|+...+.++           .+.+|-..||+.|...++..
T Consensus           232    ~~~~~~~~~~~~~~~~g~~~~~----------kl~lgl~~~g~~~~~~~~~~~~~   269 (392)
T 1LL7_B              232    FSSDKAVKDYIKAGVPAN----------KIVLGMPLYGRAFASTDGIG   269 (392)
T ss_dssp                     CCHHHHHHHHHHTTCCGG----------GEEEEEESEEEEECSCSSTT
T ss_pred                     CCHHHHHHHHHHcCCCHH----------HEEEeeecccceeeecCCCCC
```

Template alignment | Template 3D Structure | PDBe

26. 4URI_B CHITINASE-RELATED AGGLUTININ; HYDROLASE, CHITINASE-LIKE PROTEIN, GLYCOSIDE HYDROLASE FAMILY GH18, PLANT LECTIN, AGGLUTININ; HET: MPD; 1.85A {ROBINIA PSEUDOACACIA}

Probability: 96.95%, E-value: 0.0056, Score: 52.55, Aligned cols: 195, Identities: 19%, Similarity: 0.32, Template Neff: 12.4

```
Q ss_pred                    CcchHhhhhhcCccccceeeEeee----eCHHHHHHHHhccchhhhHHHHhHHHHHHHHhhccCCCCccceeeeEEeccccccc
Q Q_8363653_2         88     WPNYIEKDMKRWPHIEYYMQFVI----FGPETVSRLLDSQTAQDNFINNLKTVVARFRKDQNGNDLGYTGIEIDCEGSFS   163 (999)
Q Consensus           88     wpnyiekdmkrwphieyymqfvi----fgpetvsrlldsqtaqdnfinnlktvvarfrkdqngndlgytgieidcegsfs   163 (999)
                             +...+...-++.|++...+.+-.    +......+++.+...+..|++++...+.+..          +.||.||.|..-.
T Consensus           50     ~~~~~~~~~~~~~~~~~~~~~~ig~~~~~~~~~~~~~~~~~~~~~~~~~~~~~~~~~~~~~~~~~~~~~~~~~~~~~~~~---------~dGi~id~e~~~~~   120 (337)
T 4URI_B              50     FSTFTQTVRAKNPSVKTLLSIGGGGGRALAAVFANMASQASRRKSFIDSSIQLARRNN---------FHGLDLDWEYPSS   120 (337)
T ss_dssp                     HHHHHHHHHHHCTTCEEEEEEECSCHHHHHHHHHHHHTSHHHHHHHHHHHHHHHHHTT---------CSEEEEECSCCCS
T ss_pred                     HHHHHHHHHHHCCCCeEEEEEcCCCccchHHHHHHHhcCHHHHHHHHHHHHHHHHHCC---------CCcEEEeCCCCCC

Q ss_pred                    ccccccCCCCcchhHcHHHHhcceeeeeCCCCcce-EEeehhccCCCCCchhheecceeeeeecCCCCCcccceeEEEEe
Q Q_8363653_2         164    DSKWDTRAGDDVKYINLLKRIKNEVIIDANPDFKL-RINAHAMWGDGIPDYYRFHNYKLFAESTDKNGNPLLDEVQIMTY   242 (999)
Q Consensus           164    dskwdtragddvkyinllkrikneviidanpdfkl-rinahamwgdgipdyyrfhnyklfaestdkngnplldevqimty   242 (999)
                             .       .+.-.+..+++.++.+.--......+- .+-.-++.........++.+++..+.         .+|.+.+|+|
T Consensus           121    ~-------~~~~~~~~~~~~~~~~~~~l~~~~l~~~~~~~~~~~~~~~~~~~~~~~~~~~~~~~~~~~~~~~~~l~~---------~~d~i~i~~y   185 (337)
T 4URI_B              121    D-------IDKTNFASLIREWRAAVATESSTSGTPALLLSAAVGGSDQITPLKYYPGEAIAN--------NLDWVNVMTY   185 (337)
T ss_dssp                     H-------HHHHHHHHHHHHHHHHHHHHHHHHHTSCCCEEEEEEEECSTTTSCGGGSCHHHHHH--------HCSEEEEECC
T ss_pred                     h-------hhHhcHHHHHHHHHHHHHHHhhcccCCCCceEEEEEecCCCCcccccccCCHHHHHH--------hCCEEEeecc

Q ss_pred                    eccccCCCCCCCCCChHHH----------hcHHHHHHHHhcCCCCCCCCcceEeeEEeCCCcccccCCCCCCCCCC-------
Q Q_8363653_2         243    DFSWSGSAPGPSTPLWWM----------RNVAEWVKQCFDPSVNPNAKCTIDNVYLGGAGYGRRWPIHSDDNW-------   305 (999)
Q Consensus           243    dfswsgsapgpstplwwm----------rnvaewvkqcfdpsvnpnakctidnvylggagygrrwpihsddnw-------   305 (999)
                             ++.+..+.+....|..+.           ..+..|++..+++++            +.+.+|-.-||+.|........
T Consensus           186    ~~~~~~~~~~~~~~~~~~~~~~~~~~~~~~~~~~~~~~~~~~~~~~~~~----------~kl~~gi~~~g~~~~~~~~~~~~~~~~~~~~~~~~~~~~~   255 (337)
T 4URI_B              186    DLYTSDSYPTLTQPPAPLFYPRGIFSADEGITKWIQSGVPE----------SKLALGLPFYGFKWRLSDPNNHGLFAPAT   255 (337)
T ss_dssp                     CSSCTTTSCSBCCCSSCSCCTTSSCCHHHHHHHHHHTTCCG----------GGEEEEEESEEEEEEESCTTCCSTTCBEE
T ss_pred                     cccCCCCCCCCCCCCCCccCCCCCCCCHHHHHHHHHHcCCCH----------HHeEEeeeccceEEEeCCCCCCCCCCccc

Q ss_pred                    --CceeehhHhcc
Q Q_8363653_2         306    --GSTVTYRDLVD   316 (999)
Q Consensus           306    --gstvtyrdlvd   316 (999)
                               ....+|.++..
T Consensus           256    ~~~~~~~~~~~~~~~~~   268 (337)
T 4URI_B              256    QGLGAVKYKDIVN   268 (337)
T ss_dssp                     EEEEEEEHHHHHH
T ss_pred                     CCCCcccHHHHhc
```

Template alignment | Template 3D Structure | PDBe

27. 3WL1_A Chitinase; glycosyl hydrolase, insect, Ostrinia furnacalis, HYDROLASE; HET: NAG; 1.772A {Ostrinia furnacalis}

Probability: 96.92%, E-value: 0.0042, Score: 57.31, Aligned cols: 180, Identities: 15%, Similarity: 0.314, Template Neff: 11.2

```
Q ss_pred                  HhhhhhcCcccceeeEeeee--CHHHHHHHhccchhhhHHHHhHHHHHHHHhccCCCCccceeeeEEecccccccccccc
Q Q_8363653_2      92     IEKDMKRWPHIEYYMQFVIF--GPETVSRLLDSQTAQDNFINNLKTVVARFRKDQNGNDLGYTGIEIDCEGSFSDSKWDT   169 (999)
Q Consensus        92     iekdmkrwphieyymqfvif--gpetvsrlldsqtaqdnfinnlktvvarfrkdqngndlgytgieidcegsfsdskwdt   169 (999)
                          +..-.++.|++..++.+--.  +++..+.++.+....+.|++++..++..+         ++.||.||.|.........
T Consensus        70     ~~~~~~~~~~~~v~~sv~~~~~~~~~~~~~~~~~~~~~~~~~~~~~i~~~~~~~~---------~~dGv~id~e~~~~~~~~~~~-   139 (395)
T 3WL1_A           70     FTSLRSKHPGVKFTVAVGGWAEGGSKYSKMVAAKSTRMAFVRSVVDFLNKY---------NFDGLDLDWEYPGAADRGG-   139 (395)
T ss_dssp                   HHGGGTTCTTSEEEEEEECGGGCSHHHHHHHTSHHHHHHHHHHHHHHHHHH---------TCSEEEEECSCTTCGGGTC-
T ss_pred                   HHHHHHHCCCCEEEEEECCCcccchHHHHHHcCHHHHHHHHHHHHHHHHHc---------CCCcEEEeeeCCCccCCCC-


Q ss_pred                  CCCCcchhHcHHHHhcceeeeeCCCCcceEEeehhccCCCCCchhheecceeeeeecCCCCCcccceeEEEEeecccc-C
Q Q_8363653_2      170    RAGDDVKYINLLKRIKNEVIIDANPDFKLRINAHAMWGDGIPDYYRFHNYKLFAESTDKNGNPLLDEVQIMTYDFSWS-G   248 (999)
Q Consensus        170    ragddvkyinllkrikneviidanpdfklrinahamwgdgipdyyrfhnyklfaestdkngnplldevqimtydfsws-g   248 (999)
                          ...+...+..++++++...--. ++.+.+-+....-...-...|..              +.-...+|.+.+|+|++.++ .
T Consensus        140    ~~~~~~~~~~~l~~l~~~l~~~-~~~~~l~~~~~~~~~~~~~~~~~~~~~~------------~~l~~~~D~~~~~~y~~~~~~~~   206 (395)
T 3WL1_A           140    SFSDKDKFLYLVQELRRAFIRE-GKGWELTAAVPLANFRLMEGYHV------------PELCQELDAIHVMSYDLRGNWA   206 (395)
T ss_dssp                   CTTHHHHHHHHHHHHHHHHHHH-TSCCEEEEEECCCHHHHHHHCCH------------HHHHHHCSEEEECCCCSSCGGG
T ss_pred                   CcchHHHHHHHHHHHHHHHHHh-CCceEEEEEEecchhhhhcCCCH------------HHHHHHCCEEEEEeCcCcCCCC


Q ss_pred                  CCCCCCCChH-------------HHhcHHHHHHHhcCCCCCCCCcceEeeEEeCCCccccCCCCCCCCC
Q Q_8363653_2      249    SAPGPSTPLW-------------WMRNVAEWVKQCFDPSVNPNAKCTIDNVYLGGAGYGRRWPIHSDDN   304 (999)
Q Consensus        249    sapgpstplw-------------wmrnvaewvkqcfdpsvnpnakctidnvylggagygrrwpihsddn   304 (999)
                          ..+++..|+|             +-..+..|.+..+++           ..+.+|-..||+.|...+...
T Consensus        207    ~~~~~~~~~~~~~~~~~~~~~~~~~~~~~~~~~~g~~~----------~kl~lg~~~~g~~~~~~~~~~~   265 (395)
T 3WL1_A           207    GFADVHSPLYKRPHDQWAYEKLNVNDGLQLWEDKGCPT----------NKLVVGIPFYGRSFTLSSGNN   265 (395)
T ss_dssp                   TBCCCSSCSSCCTTCCGGGTTCSHHHHHHHHHHTTCCG----------GGEEEEEESEEEEEEBCTTCC
T ss_pred                   ccCCCCCcccCCCcchhHhhcCCHHHHHHHHHHcCCCH----------HHEEEEeecceeEEEecCCCC
```

Template alignment | Template 3D Structure | PDBe

28. 3ARX_A Chitinase A; TIM BARREL, INHIBITOR COMPLEX, GLYCOSIDASE, HYDROLASE, HYDROLASE-HYDROLASE INHIBITOR complex; HET: POY; 1.16A {Vibrio harveyi}

Probability: 96.88%, E-value: 0.0057, Score: 61.97, Aligned cols: 191, Identities: 20%, Similarity: 0.267, Template Neff: 10

```
Q ss_pred                  HhhhhhcCcccceeeEeeeeCHHHHHHHhccchhhhHHHHhHHHHHHHHhccCCCCccc-eeeeEEeccccccccccccC
Q Q_8363653_2      92     IEKDMKRWPHIEYYMQFVIFGPETVSRLLDSQTAQDNFINNLKTVVARFRKDQNGNDLG-YTGIEIDCEGSFSDSKWDTR   170 (999)
Q Consensus        92     iekdmkrwphieyymqfvifgpetvsrlldsqtaqdnfinnlktvvarfrkdqngndlg-ytgieidcegsfsdskwdtr   170 (999)
                          +..-.+++|++.-++-+--.........+.+.....+|++++..++..+         + |.||+||-|..-...-....
T Consensus        235    l~~lk~~~p~~k~llsigg~~~~~~~~~~~~~~~~~~~~~~f~~~i~~~~~~~~---------~~fDGidld~e~~~~~~~~~~~~~   305 (584)
T 3ARX_A           235    LMALKQRNPDLKIIPSIGGWTLSDPFYDFVDKKNRDTFVASVKKFLKTW---------KFYDGVDIDWEFPGGGGAAADK   305 (584)
T ss_dssp                   HHHHHHHCTTCEEEEEEEEESSSCGGGGGGGSHHHHHHHHHHHHHHHHC---------TTCCEEEEEESCTTSCSSCTTC
T ss_pred                   HHHHHHHCCCCeEEEEECCCcCCccHHHhcCHHHHHHHHHHHHHHHHHc---------CCCCcEEEeccCCCCCCccccC


Q ss_pred                  C---CCcchhHcHHHHhcceeee-----eCCCCcceEEeehhccCCCCCchhheecceeeeeecCCCCCcccceeEEEEe
Q Q_8363653_2      171    A---GDDVKYINLLKRIKNEVII-----DANPDFKLRINAHAMWGDGIPDYYRFHNYKLFAESTDKNGNPLLDEVQIMTY   242 (999)
Q Consensus        171    a---gddvkyinllkriknevii-----danpdfklrinahamwgdgipdyyrfhnyklfaestdkngnplldevqimty   242 (999)
                          .   .+..+|..++|+++...-.     ..+.-..+.+.+....... -++-+...+                +|.+.+|+|
T Consensus        306    ~~~~~~~~~~~~~~~~l~~~l~~~~~~~~~~~~~~ls~~~~~~~~~~~~~~~~-~~~~~~~~~~~--------------vD~v~i~~y   370 (584)
T 3ARX_A           306    GDPVNDGPAYIALMRELRVMLDELEAETGRTYELTSAIGVGYDKIED-VDYADAVQY--------------MDYIFAMTY   370 (584)
T ss_dssp                   CCTTTHHHHHHHHHHHHHHHHHHHHHHSCCCEEEEEEECCSHHHHTT-SCHHHHGGG--------------CSEEEECCC
T ss_pred                   CCccccHHHHHHHHHHHHHHHHHHHHHhCCeEEEEEEecCCchhhcc-CCHHHHHHH--------------CCEEEEEec


Q ss_pred                  ecccc-CCCCCCCCCh----------------------------HHHhcHHHHHHHhcCCCCCCCCcceEeeEEeCCCcc
Q Q_8363653_2      243    DFSWS-GSAPGPSTPL----------------------------WWMRNVAEWVKQCFDPSVNPNAKCTIDNVYLGGAGY   293 (999)
Q Consensus        243    dfsws-gsapgpstpl----------------------------wwmrnvaewvkqcfdpsvnpnakctidnvylggagy   293 (999)
                          ++...  ...+++..|+                            ++-..+..|++..+.+          +.+.||-..|
T Consensus        371    ~~~~~~~~~~~~~~pl~~~~~~~~~~~~~~~~~~~~~~~~~~~~~~~~~~~~~~~~~~~~g~p~----------~Klvlgl~~~   440 (584)
T 3ARX_A           371    DFYGGWNNVPGHQTALYCGSFMRPGQCDGGGVDENGEPYKGPAYTADNGIQLLLAQGVPA----------NKLVLGTAMY   440 (584)
T ss_dssp                   CSSCTTSSCCCCSSCSSCCTTSCTTTTTSCSBCTTSCBCCSCCSCHHHHHHHHHHTTCCG----------GGEEEEEESE
T ss_pred                   cccCccCCCCCCCCcccCCCCCCCCCCCCCCCCCCCCCCCCCCCCHHHHHHHHHHCCCCH----------HHEEEecccc


Q ss_pred                  ccCCCCCCCC-------------------------CCCceeehhHhcc
Q Q_8363653_2      294    GRRWPIHSDD-------------------------NWGSTVTYRDLVD   316 (999)
Q Consensus        294    grrwpihsdd-------------------------nwgstvtyrdlvd   316 (999)
                          |+.|......                         .-+..++|+++..
T Consensus        441    g~~~~~~~~~~~~~~~~~~~~~~~~~~~~~~~~~~~~~~~~~~~~~y~ei~~   488 (584)
T 3ARX_A           441    GRGWEGVTPDTLTDPNDPMTGTATGKLKGSTAQGVWEDGVIDYKGIKS   488 (584)
T ss_dssp                   EEEEECCCGGGCSSTTCGGGSCCSEECCCCGGGTCSBTTEEEHHHHCC
T ss_pred                   ceeecCCCcccCCCCCCCCCccCCCCcCCCcccccccCCeecHHHHHH
```

Template alignment | Template 3D Structure | PDBe

29. 4TX8_A Probable chitinase A; Chitinase Family 18 Glycoside Hydrolase, HYDROLASE; HET: NAG, FLC; 2.17A {Chromobacterium violaceum}

Probability: 96.88%, E-value: 0.0015, Score: 62.35, Aligned cols: 176, Identities: 16%, Similarity: 0.197, Template Neff: 10.6

```
Q ss_pred                  eeeeCHHHHHHHhccchhhhHHHHhHHHHHHHHhccCCCCccceeeeEEeccccccccccccCCCCcchhHcHHHHhcce
Q Q_8363653_2      108    FVIFGPETVSRLLDSQTAQDNFINNLKTVVARFRKDQNGNDLGYTGIEIDCEGSFSDSKWDTRAGDDVKYINLLKRIKNE   187 (999)
Q Consensus        108    fvifgpetvsrlldsqtaqdnfinnlktvvarfrkdqngndlgytgieidcegsfsdskwdtragddvkyinllkrikne   187 (999)
                          .+-+|-..-+.++.+.+..++|++++..++.++         ++.||+||.|..   ...+....+.-.++.+|+.++.+
```

```
T Consensus        178   ~~sigG~~~~~~~~~~~~~~~~~~~~~~~~i~~~---------~~dGiDiD~E~~---~~~~~~~~~~~~~~~~l~~l~~~   245 (439)
T 4TX8_A           178   VLSLGGQNGSVTLNNATQVQNFVNSLYGILTQY---------GFDGIDLDLESG---SGIVVGAPVVSNLVSAVKQLKAK   245 (439)
T ss_dssp                EEEEESTTCCCCCCSHHHHHHHHHHHHHHHHHHH---------TCCEEEEECTTT---TTCCTTCHHHHHHHHHHHHHHHH
T ss_pred                 EEEECCCCCccccCCHHHHHHHHHHHHHHHHHHh---------CCCEEEEeCCCC---CccCCChHHHHHHHHHHHHHHHH


Q ss_pred                 eeeeCCCCcceEEeehhccCCCCCchhheecceeeeeeCCCCCcccceeEEEEeecccCCCCCC-----CCChHHHhc
Q Q_8363653_2      188   VIIDANPDFKLRINAHAMWGDGIPDYYRFHNYKLFAESTDKNGNPLLDEVQIMTYDFSWSGSAPGP-----STPLWWMRN   262 (999)
Q Consensus        188   viidanpdfklrinahamwgdgipdyyrfhnyklfaestdkngnplldevqimtydfswsgsapgp-----stplwwmrn   262 (999)
                         .    .+.+.|-+...+.+.++-...+...........+-...  +|-|.+|+||+....+...+     .++..|...
T Consensus        246   ~----~~~~ls~~~~~~~~~~~~~~~~~~~~~~~~~~~~~~~~~~--vD~i~vm~yd~~~~~~~~~~~~~~~~~~~~~~~~   319 (439)
T 4TX8_A           246   I----GPNFYLSMAPEHPYVQGGFVAYGGNWGAYLPIIDGLRDD--LSVIHVQYYNNGGLYTPYSTGVLAEGSADMLVGG   319 (439)
T ss_dssp                H----CTTCEEEECCBGGGTGGGGTCCBTTBTTTHHHHHHTTTT--CSEEEEECSSSCCBCCTTSSSCBCTTCHHHHHHH
T ss_pred                 H----CCCeEEEECCCChhhcCCcccccccchhhhHHHHHHHHHc--CcEEEeecCCCCCCCCCCCCCccCCCCcceeeehh


Q ss_pred                 HHHHHHHhcCC--------CCCCCCcceEeeEEeCCCccccCCCCCCCCCCCceeehhHh
Q Q_8363653_2      263   VAEWVKQCFDP--------SVNPNAKCTIDNVYLGGAGYGRRWPIHSDDNWGSTVTYRDL   314 (999)
Q Consensus        263   vaewvkqcfdp--------svnpnakctidnvylggagygrrwpihsddnwgstvtyrdl   314 (999)
                         +..++++...-         .+.++       .+.+|-..|++.|.        +..++|.++
T Consensus        320   ~~~~~~~~~~~~~~~~~~~g~~~~------klvlG~p~~~~~~~~-------~g~~~~~~~   366 (439)
T 4TX8_A           320   SKMLIEGFPIANGASGSFKGLRPD------QVAFGVPSGRSSAN-------SGFVTADTV   366 (439)
T ss_dssp                HHHHHHEEECCCSSCCEEECCCGG------GEEEEEESSTTSCS-------SCCCCHHHH
T ss_pred                 hchhhcCccccCCCCCCCCCCChH------HEEEecCCCCCCc-------CCcCCHHHH
```

Template alignment | Template 3D Structure | PDBe

30. 6XYZ_A Candidate chitinase Glycoside hydrolase family 18; Chitinase GH18 Chitin ChiB, HYDROLASE; HET: EDO; 1.63A {Flavobacterium johnsoniae (strain ATCC 17061 / DSM 2064 / UW101)}

Probability: 96.8%, E-value: 0.011, Score: 52.73, Aligned cols: 179, Identities: 15%, Similarity: 0.192, Template Neff: 11.4

```
Q ss_pred                 CcchHhhhhhcCccccceeeEeeeeCH-----HHHHHHhccchhhhHHHHhHHHHHHHHhhccCCCCcccceeeeEEecccc
Q Q_8363653_2       88   WPNYIEKDMKRWPHIEYYMQFVIFGP-----ETVSRLLDSQTAQDNFINNLKTVVARFRKDQNGNDLGYTGIEIDCEGSF   162 (999)
Q Consensus         88   wpnyiekdmkrwphieyymqfvifgp-----etvsrlldsqtaqdnfinnlktvvarfrkdqngndlgytgieidcegsf   162 (999)
                         +.+.+..-.++.|++.-++-+--...     +..+.++.+......|++++..++.+.          +.||.||.|...
T Consensus         61   ~~~~~~~~~~~~~~~~~v~~~v~~~~~~~~~~~~~~~~~~~~~~~~~~~~l~~~~~~~~~~---------~dGi~ld~e~~~   131 (325)
T 6XYZ_A            61   IDAVTKYVRSVNSNIVISISLAGGVISTEQAANWSLLIDKPENRPAFMQNISKFVTDHN---------LDGVDVDLEWDA   131 (325)
T ss_dssp                HHHHHHHHHHHCTTCEEEEEEECSCCCHHHHHHHHHHSSGGGHHHHHHHHHHHHHHTT---------CSEEEEECCGGG
T ss_pred                 HHHHHHHHHHhCCCcEEEEEEecCCCChhhhhcHHHHhhChhHHHHHHHHHHHHHHHHcC---------CCcEEeecCccc


Q ss_pred                 ccccccCCCCccchhHcHHHHhcceeeeeCCCCcceEEeehhccCCCCCchhheecceeeeeeCCCCCCcccceeEEEEe
Q Q_8363653_2      163   SDSKWDTRAGDDVKYINLLKRIKNEVIIDANPDFKLRINAHAMWGDGIPDYYRFHNYKLFAESTDKNGNPLLDEVQIMTY   242 (999)
Q Consensus        163   sdskwdtragddvkyinllkrikneviidanpdfklrinahamwgdgipdyyrfhnyklfaestdkngnplldevqimty   242 (999)
                         +           ...+..+++.++...    ....+.+.+..........                 -+.+.-...+|-+-+|+|
T Consensus        132   ~----------~~~~~~~~~~~~~~l~~~l-----------~~~~~~~~~~~~~~~~~~~~~~~~~~~~~~~~~~~~~~D~i~i~~y   183 (325)
T 6XYZ_A           132   V----------TSGYSGFVVELRKEL---TDRKKLLTAALPNNTRFVN---------------INSEALNAFDFINIMAY   183 (325)
T ss_dssp                C----------CTTHHHHHHHHHHHH---HHTTCEEEEEEESSCCCTT---------------SCHHHHHHSSEEEEECC
T ss_pred                 c----------ccchhHHHHHHHHHh---hhccceEeEEccCCccccC---------------CCHHHHHhCCEEEEecC


Q ss_pred                 ecc--ccCCCCCCCCChHHHhcHHHHHHHhcCCCCCCCCcceEeeEEeCCCcccccCCCCCCCCCCceeehhHhcc
Q Q_8363653_2      243   DFS--WSGSAPGPSTPLWWMRNVAEWVKQCFDPSVNPNAKCTIDNVYLGGAGYGRRWPIHSDDNWGSTVTYRDLVD   316 (999)
Q Consensus        243   dfs--wsgsapgpstplwwmrnvaewvkqcfdpsvnpnakctidnvylggagygrrwpihsddnwgstvtyrdlvd   316 (999)
                         ++.   |+...+++..|+.|+....+.+.+          ..+.-..+.+|-.-||+.|..-.     ...++|.++.+
T Consensus        184   ~~~~~~~~~~~~~~~~~~~~~~~~~~~~~~---------~g~~~~ki~~gi~~~g~~~~~~~~~~~~~~~~~~~~~~~   246 (325)
T 6XYZ_A           184   DSTGPWSPNKIEQHSSFEFAKEGVEFWKK---------QNVPSEKLTLGVPFYGYNFTYPE----VTSSTFGEIIQ   246 (325)
T ss_dssp                CSSCTTSTTCCSCSSCHHHHHHHHHHHHHH---------TTCCGGGEEEEEESEEEECSSSS----CEEEEHHHHHH
T ss_pred                 CCCCCCCCCCCCCCChHHHHHHHHHHHHHH---------CCCCHHHeEEEEeeccccCCCCc----ccccCHHHHHh
```

Template alignment | Template 3D Structure | PDBe

31. 5Y2A_B insect group II chitinase; GH18 chitinase, HYDROLASE; HET: NAG, MAN; 1.9A {Ostrinia furnacalis}

Probability: 96.79%, E-value: 0.0062, Score: 55.53, Aligned cols: 213, Identities: 15%, Similarity: 0.212, Template Neff: 11.4

```
Q ss_pred                 ccccccccCccccccCCCCCcccCcccchHhhhhhcCccccceeeEeeeeC-----HHHHHHHhccchhhhHHHHhHHHHHHHHhhc
Q Q_8363653_2       68   SFTSFTDATTYDRYAPGRTLWPNYIEKDMKRWPHIEYYMQFVIFG-----PETVSRLLDSQTAQDNFINNLKTVVARFRK   142 (999)
Q Consensus         68   sftsftdattydryapgrtlwpnyiekdmkrwphieyymqfvifg-----petvsrlldsqtaqdnfinnlktvvarfrk   142 (999)
                         .|..+....+.........-+..-+.+.+++...-.-.+...|.|     ++..++++.+.+..+.|++++..++.++
T Consensus         38   ~~~~~~~~~~~~~~~~~~~~~~~~~~~~~~~~i~~~~~~~~~~v~~sigg~~~~~~~~~~~~~~~~~~~~~~~~~~~~~i~~~~~~~--   115 (383)
T 5Y2A_B            38   AFAVLDKEELVIKSHDIWLDVENKFYEKVTALKSHGVKVLLGLGGWDDSAGDKYSRLVNNVSARKFVVHAVDFLEQY--   115 (383)
T ss_dssp                EEEEECTTTCSEECSCHCCCCCTCHHHHHHGGGGGTCEEEEEEECTGGGSSSHHHHHHTCHHHHHHHHHHHHHHHHHH--
T ss_pred                 EEeEecCCceeEeecccccccchhhHHHHHHHHhCCCeEEEEEecCCccccCccchhHhcCCHHHHHHHHHHHHHHHHHh--


Q ss_pred                 cCCCCccceeeeEEeccccccccccc--CCCCcchhHcHHHHhcceeeeeCCCCcceEEeehhccCCCCCchhheecce
Q Q_8363653_2      143   DQNGNDLGYTGIEIDCEGSFSDSKWDT--RAGDDVKYINLLKRIKNEVIIDANPDFKLRINAHAMWGDGIPDYYRFHNYK   220 (999)
Q Consensus        143   dqngndlgytgieidcegsfsdskwdt--ragddvkyinllkrikneviidanpdfklrinahamwgdgipdyyrfhnyk   220 (999)
                               |+.||.||.|..-.....-+.   ...+...++.++++++...-.  +..+.+.+.++..........++-.+..+
T Consensus        116   -------~~dGidid~e~~~~~~~~~~~~~~~~~~~~~~~~~~l~~~l~~~~~~l~~~~~~~~~~~~~~~~~~~~~~~~~~-   186 (383)
T 5Y2A_B           116   -------GFDGLDLDWEYPKCWQVECEKGPDSDKQGFADLVKELRKAFNRR-GMLLSAAVSASKRVIDYAYNVPALSMN-   186 (383)
T ss_dssp                -------TCCEEEEECSCSSSBTBTTTSCTTHHHHHHHHHHHHHHHHTT-TCEEEEEECCCHHHHHHHCCHHHHHHH-
T ss_pred                 -------CCCCEEEcccCCCccccccCCCChhhHHHHHHHHHHHHHHHhc-CcEEEEEeCCChHHHhhccCHHHHHHh-


Q ss_pred                 eeeeecCCCCCccccceeEEEEeecccc-CCCCCCCCChH-----------HHhcHHHHHHHhcCCCCCCCCCcceEeeEEe
Q Q_8363653_2      221   LFAESTDKNGNPLLDEVQIMTYDFSWS-GSAPGPSTPLW-----------WMRNVAEWVKQCFDPSVNPNAKCTIDNVYL   288 (999)
Q Consensus        221   lfaestdkngnplldevqimtydfsws-gsapgpstplw-----------wmrnvaewvkqcfdpsvnpnakctidnvyl   288 (999)
```

```
                                      +|.+.+|+|++.++ +..+++..|+-           ....+..|++.-++++          .+.+
T Consensus         187     -------------vD~v~i~~y~~~~~~~~~~~~~~~~~~~~~~~~~~~~~~~~~~~~~~~~g~~~~----------kl~~  243 (383)
T 5Y2A_B            187     -------------LDWISLMTYDYHGQWDKKTGHVAPMYVHDKDTDNTFNVNFTVNYWINKGADRK----------KLVV  243 (383)
T ss_dssp                   -------------CSEEEECCCCSSCGGGSBCCCSSCCSCCTTCSCTTCSHHHHHHHHHHTTCCGG----------GEEE
T ss_pred                   -------------CCEEEEecccccCCCccCCCCCCCCCCCCCCCCCceeHHHHHHHHHHcCCCHH----------HEEE


Q ss_pred                   CCCccccCCCCCCCCCCCce------------------eehhHh
Q Q_8363653_2       289     GGAGYGRRWPIHSDDNWGST------------------VTYRDL  314 (999)
Q Consensus         289     ggagygrrwpihsddnwgst------------------vtyrdl  314 (999)
                            |-.-||+.|......+-+..                  ++|+++
T Consensus         244     g~~~~g~~~~~~~~~~~~~~~~~~~~~~~~~~~~~~~~~~~~~~  287 (383)
T 5Y2A_B            244     GVPFYGQSFSVVEGAGTGLGAPTYAGGEAGDETRARGFLSFYEI  287 (383)
T ss_dssp                   EEESEEEEEEEECTTSCSSTTCBEEEECCCCTTTCCTTEEEHHHH
T ss_pred                   EEEcceEEEEeccCCCCCCCCccCCCCCCccccccccceecHHHH
```

Template alignment | Template 3D Structure | PDBe

32. **1OQ1_B Protein yesU; Structural genomics, Singleton, PSI, Protein Structure Initiative, Midwest Center for Structural Genomics, MCSG, UNKNOWN FUNCTION; HET: MSE, GOL, ACY; 1.7A {Bacillus subtilis} SCOP: b.29.1.17**

Probability: 96.76%, E-value: 0.25, Score: 39.62, Aligned cols: 158, Identities: 14%, Similarity: 0.131, Template Neff: 12.6

```
Q ss_pred                   cCCCCCCCCceeeC-----CCCCeeeeeCCCCcCcCCCccceEEEEeeEec-CcEEEEEEEEeeCC-cceeeEEeecCC-
Q Q_8363653_2       675     DNGFTQGHWPVETD-----ENGTACARFRPNDPKNNGVTSGQLVLNYTYKT-TNISCEVQFKVKSG-RRAGIRFASTGP-  746 (999)
Q Consensus         675     dngftqghwpvetd-----engtacarfrpndpknngvtsgqlvlnytykt-tniscevqfkvksg-rragirfastgp-  746 (999)
                            +++-...+|.....     ++|..+..-.+         ..+...+-..... .+..+|+.++..++ ..+++-|...++
T Consensus          17     ~~~~~~~~W~~~~~~~~~~~~~g~l~~~~~~--------~~~~~~~~~~~~~~~~i~~~~~~~~~~~~~~~~~~~~~~~~~~~~  88 (223)
T 1OQ1_B             17     RSKSDVKDWRMEGGGQISFDDHSLHLSHVQ--------DEAHFVFWCPETFPDGIIVTWDFSPIEQPGLCMLFFAAAGIR  88 (223)
T ss_dssp                   CSGGGGTTCEEEESEEEECTTSSEEEEESS--------TTCCEEEECSSCBCSSEEEEEEEEEEECSEEEEEEEEEEETT
T ss_pred                   CCcccccCEEEecceeEEEcCCeEEEEecC--------CCCeEEEEeCCccCCcEEEEEEEEeCCCCCeEEEEEEEecCCC


Q ss_pred                   -----------------------CCcEEEEEEcccCeEeeeeec------cCcceeeEeeeccccccChhheEEEEEeee
Q Q_8363653_2       747     -----------------------GDGYVFLIDYQTQEAMMFYET------AGSSQLVASASLGDRRADYDELITLKVLVN  797 (999)
Q Consensus         747     -----------------------gdgyvflidyqtqeammfyet------agssqlvasaslgdrradydelitlkvlvn  797 (999)
                                               ..+|.+.++-........|..      .+...+.....--.....-++-.++++.++
T Consensus          89     ~~~~~~~~~~~~~~~~~~~~~~~~~~~~~~~~~~~~~~~~~~~~~~~~~~~~~~~~~~~~~~~~~~~~~~~~~~~~~~~~~i~~~  168 (223)
T 1OQ1_B             89     GEDLFDPSLRKRTGTYPEYHSGDINALHLSYFRRKYAEERAFRTCNLRKSRGFHLAAMGADPLPSPDDADSPYRMKLIKD  168 (223)
T ss_dssp                   SCCTTCTTSCCCCSCTHHHHSSSEEEEEEEEEEEESSHHHHTSCEEEEEEETTTEEEEEEECCCCGGGCCSCEEEEEEEE
T ss_pred                   CcccCCcchhcccCCcccccccceeeEEEEEecccchhhhcccceeccccCCCceEeeecCCCCCCCCCCCCcEEEEEEEE


Q ss_pred                   CCeeeEEeccEEEEEecCC-----CCCCCCceeEEEec-cceEEEEecc
Q Q_8363653_2       798     NGKCRCYFGNVMFFMDMNL-----PHMSPGGIGFVATN-CDAYLYKLSI  840 (999)
Q Consensus         798     ngkcrcyfgnvmffmdmnl-----phmspggigfvatn-cdaylyklsi  840 (999)
                            .+..++|..+...+--...     +...+|.+||.+.. +.+.+-.+.|
T Consensus         169     g~~~~~~vng~~v~~~~~~~~~~~~~~~~~~g~igl~~~~~~~~~~~~~i~i  217 (223)
T 1OQ1_B            169     KGYVHFSINGLPILEWMDDGSTYGPVLTKGKIGFRQMAPMKAVYRDFAV  217 (223)
T ss_dssp                   TTEEEEEETTEEEEEEECCSSSSSSCCCSBEEEEEEETTCEEEEEEEEE
T ss_pred                   CCEEEEEECCEEEEEEEcCCCCCCCCCCcEEEEEEecCeEEEEEeEEE
```

Template alignment | Template 3D Structure | PDBe

33. **6LE8_A Probable endochitinase; Nematode family I chitinase, HYDROLASE, inhibitor, GH18; HET: BTB, EAF; 1.39909326225A {Caenorhabditis elegans}**

Probability: 96.75%, E-value: 0.0065, Score: 55.15, Aligned cols: 179, Identities: 23%, Similarity: 0.35, Template Neff: 11.5

```
Q ss_pred                   HhhhhhcCcccceeeEee--eeCHHHHHHHhccchhhhHHHHhHHHHHHHhhccCCCCcccceeeeEEeccccccccccc
Q Q_8363653_2        92     IEKDMKRWPHIEYYMQFV--IFGPETVSRLLDSQTAQDNFINNLKTVVARFRKDQNGNDLGYTGIEIDCEGSFSDSKWDT  169 (999)
Q Consensus          92     iekdmkrwphieyymqfv--ifgpetvsrlldsqtaqdnfinnlktvvarfrkdqngndlgytgieidcegsfsdskwdt  169 (999)
                            +..--++.|++..++.+-  .++.+..+.++.+.+....|++++..++.++         ++.||+||.|..-.
T Consensus          68     i~~~~~~~~~~~~~v~~~i~g~~~~~~~~~~~~~~~~~~~~~~~~~~~~~i~~~~~~~~~---------~~dGv~ld~~~~~~~------  132 (380)
T 6LE8_A             68     VNKLKVTDTQLKTLLSFGGWSFGTALFQGMAASSASRKVFIDSAITFVRTW---------GFDGIDIDWEYPSG------  132 (380)
T ss_dssp                   HHTHHHHCTTCEEEEEEECTTTCSHHHHHHTSSHHHHHHHHHHHHHHHHHHH---------TCSEEEEECSCCCS------
T ss_pred                   HHHHhhhCCCCeEEEEEECCCccCchhHHHHcCCHHHHHHHHHHHHHHHHHH---------CCCcEEEEEeCCCC------


Q ss_pred                   CCCCcchhHcHHHHhcceeeeeCCCCcceEEeehhccCCCCCchhheecceeeeeecCCCCCcccceeEEEEeecccc-C
Q Q_8363653_2       170     RAGDDVKYINLLKRIKNEVIIDANPDFKLRINAHAMWGDGIPDYYRFHNYKLFAESTDKNGNPLLDEVQIMTYDFSWS-G  248 (999)
Q Consensus         170     ragddvkyinllkrikneviidanpdfklrinahamwgdgipdyyrfhnyklfaestdkngnplldevqimtydfsws-g  248 (999)
                             ..+...++.++++++..--...-..+-..................++++.+         ...+|.+.+|+|++... .
T Consensus         133     -~~~~~~~~~~~~~~l~~~l~~~~~~~~~~~~~~~~l~~~~~~~~~~~~~~~~~~~~~l--------~~~~D~~~i~~y~~~~~~~  203 (380)
T 6LE8_A            133     -ATDMANYVALVKELKAACESEAGSTGKDRLLVTAAVAAGPATIDAGYDIPNL--------APNFDFILLMSYDFFGAWA  203 (380)
T ss_dssp                   -HHHHHHHHHHHHHHHHHHHHHHHHHHCSCCCEEEEEECCSHHHHHHHCCHHHH--------GGGCSEEEECCCCSSCTTS
T ss_pred                   -hhhHHHHHHHHHHHHHHHHHHHhhcCCCceEEEEEEEcCCHHHHhcCCCHHHH--------HHhCCEEEEEcccccCccc


Q ss_pred                   CCCCCCCCh-------------HHHhcHHHHHHHHhcCCCCCCCCcceEeeEEeCCCccccCCCCCCCCC
Q Q_8363653_2       249     SAPGPSTPL-------------WWMRNVAEWVKQCFDPSVNPNAKCTIDNVYLGGAGYGRRWPIHSDDN  304 (999)
Q Consensus         249     sapgpstpl-------------wwmrnvaewvkqcfdpsvnpnakctidnvylggagygrrwpihsddn  304 (999)
                            ..+++..|+               ++...+..|.+..+++           +.+.+|-..||..|.......
T Consensus         204     ~~~~~~~~~~~~~~~~~~~~~~~~~~~~~~~~~~~~~~~----------~kl~~~~~~~~g~~~~~~~~~~~~  262 (380)
T 6LE8_A            204     SLVGFNSPLYATAELPAEWNGWNVDSSARYWNQKGMPK----------EKIIVGMPTYGRGWTLNNASA  262 (380)
T ss_dssp                   SBCCCSSCSSCCTTSCGGGTTCSHHHHHHHHHHTTCCG----------GGEEEEEESEEEEEEESSTTC
T ss_pred                   cccCCCCCCCCCCCCChhhcCCcHHHHHHHHHHcCCCH----------HHEEEeecccceeEECCCCcC
```

Template alignment | Template 3D Structure | PDBe

34. 6BT9_A Chitinase; HYDROLASE; 2.26A {Bacillus thuringiensis}

Probability: 96.68%, E-value: 0.014, Score: 57.93, Aligned cols: 190, Identities: 20%, Similarity: 0.351, Template Neff: 11.2

```
Q ss_pred                HhhhhhcCcccceeeEeeeeC-HHHHHHHhccchhhhHHHHhHHHHHHHhhccCCCCccceeeeEEecc--cccccccc
Q Q_8363653_2      92    IEKDMKRWPHIEYYMQFVIFG-PETVSRLLDSQTAQDNFINNLKTVVARFRKDQNGNDLGYTGIEIDCE--GSFSDSKWD   168 (999)
Q Consensus        92    iekdmkrwphieyymqfvifg-petvsrlldsqtaqdnfinnlktvvarfrkdqngndlgytgieidce--gsfsdskwd   168 (999)
                         +.+..++.|++...+.+--..  ......++.+......|++++..++..+          ++.||+||.|  +......+.
T Consensus       148    ~~~l~~~~~~~kv~~sigg~~~~~~~~~~~~~~~~~~~~~~~~i~~~~~~---------~~dGidid~e~~~~~~~~~~~   218 (672)
T 6BT9_A          148    LKRLKAKYPHLKTIISVGGWTWSNRFSDMAADEKTRKVFAESTVAFLRAY---------GFDGVDLDWEYPGVETIPGGS   218 (672)
T ss_dssp                HHHHHHHSTTCEEEEEEEETTTCTTHHHHHSSHHHHHHHHHHHHHHHHHH---------TCSEEEEEESCTTSCSSTTCC
T ss_pred                HHHHHHHCCCCEEEEEeCCCccchhHHHHhcCHHHHHHHHHHHHHHHHHHh---------CCCeEEEecCCCCCCCCCCCC


Q ss_pred                cCCCCcchhHcHHHHhcceeeeeC---CCCcceEEeehhccCCCCC-chhheecceeeeeecCCCCCcccceeEEEEeec
Q Q_8363653_2     169    TRAGDDVKYINLLKRIKNEVIIDA---NPDFKLRINAHAMWGDGIP-DYYRFHNYKLFAESTDKNGNPLLDEVQIMTYDF   244 (999)
Q Consensus       169    tragddvkyinllkrikneviida---npdfklrinahamwgdgip-dyyrfhnyklfaestdkngnplldevqimtydf   244 (999)
                         ....+...+.++++.++++.--..   .+.+.|.+....-...... ++-++..+                +|.+.+|+||+
T Consensus       219    ~~~~~~~~~~~~l~~l~~~l~~~~~~~~~~~~~~~~l~~~~~~~~~~~~~~~~~~~~l~~~--------------vD~v~v~~yd~   284 (672)
T 6BT9_A          219    YRPEDKQNFTLLLQDVRNALNKAGAEDGKQYLLTIASGASQRYADHTELKKISQI--------------LDWINIMTYDF   284 (672)
T ss_dssp                CCTTHHHHHHHHHHHHHHHHHHHHHHHTCCCEEEEEEESSHHHHHTSCHHHHHHH--------------SSEEEEECCCS
T ss_pred                CCHHHHHHHHHHHHHHHHHHHHHcccccCCCceEEEEEecCChhhhcccCHHHHHHH--------------CcEEEEecCcc


Q ss_pred                cc--------------cCCCCCCCCChHHHhcHHHHHHHhcCCCCCCCCcceEeeEEeCCCccccCCCCCCCCC-----
Q Q_8363653_2     245    SW--------------SGSAPGPSTPLWWMRNVAEWVKQCFDPSVNPNAKCTIDNVYLGGAGYGRRWPIHSDDNW-----   305 (999)
Q Consensus       245    sw--------------sgsapgpstplwwmrnvaewvkqcfdpsvnpnakctidnvylggagygrrwpihsddnw-----   305 (999)
                         .+              ....|.+....+....+..|+++...+          +.+.+|-.-||+.|........
T Consensus       285    ~~~~~~~~~~~~~l~~~~~~~~~~~~~~~~~~~~~~~~~~g~~~----------~ki~~g~~~~g~~~~~~~~~~~~~~~~~~   354 (672)
T 6BT9_A          285    HGGWEATSNHNAALYKDPNDPAANTNFYVDGAINVYTNEGVPV----------DKLVLGVPFYGRGWKSCGKENNGQYQP   354 (672)
T ss_dssp                SCTTSSBCCCSSCCSCCTTSTTGGGTCSHHHHHHHHHHTTCCG----------GGEEEEEESEEEEESEECCGGGGTTCB
T ss_pred                cCCccccCCCCCcccCCCCCcccccCCcHHHHHHHHHHcCCCH----------HHEEEeeeeceEEeecCCCccCCcccc


Q ss_pred                ----------------------CceeehhHh
Q Q_8363653_2     306    ----------------------GSTVTYRDL   314 (999)
Q Consensus       306    ----------------------gstvtyrdl   314 (999)
                                             ...++|.++
T Consensus       355    ~~~~~~~~~~~~~~~~~~~~~~~~~~~~~y~ei   385 (672)
T 6BT9_A          355    CKPGSDGKLASKGTWDDYSTGDTGVYDYGDL   385 (672)
T ss_dssp                EECCTTSCCBCCCTTCCTTTCCCCEEEHHHH
T ss_pred                CCCCCCCcCCCCCcccCcCCCCceeeeHHHH
```

Template alignment | Template 3D Structure | PDBe

35. 5YUQ_B Chintase; Chitinase, Rhizomucor miehei, HYDROLASE; 1.56A {Rhizomucor miehei}

Probability: 96.67%, E-value: 0.0098, Score: 52.63, Aligned cols: 164, Identities: 21%, Similarity: 0.284, Template Neff: 12.1

```
Q ss_pred                CHHHHHHHhccchhhhHHHHhHHHHHHHhhccCCCCccceeeeEEecccc---ccccccccCCCCcchhHcHHHHhccee
Q Q_8363653_2     112    GPETVSRLLDSQTAQDNFINNLKTVVARFRKDQNGNDLGYTGIEIDCEGS---FSDSKWDTRAGDDVKYINLLKRIKNEV   188 (999)
Q Consensus       112    gpetvsrlldsqtaqdnfinnlktvvarfrkdqngndlgytgieidcegs---fsdskwdtragddvkyinllkriknev   188 (999)
                         .......++.+.....+|++++..++.++          ++.||.||.|..   -.........+...+..+++.++...
T Consensus        69    ~~~~~~~~~~~~~~~~~~~~~~~~~~~~~~~---------~~dGi~ld~~~~~~~~~~~~~~~~~~~~~~~~~~~~~~~l~~~~   139 (378)
T 5YUQ_B           69    GSLYFSDLLKSSSSFDNFVSNLVDVVKEY---------DLDGLNLDWEYPNSPNGVACNSKDENDTANYLKLFKALREKL   139 (378)
T ss_dssp                GCSSHHHHHHSHHHHHHHHHHHHHHHHHHH---------TCSEEEEECSCTTCSSSSTTCCCCTTHHHHHHHHHHHHHHHH
T ss_pred                CchhHHHHhcCHHHHHHHHHHHHHHHHHHc---------CCCcEEEcccCCCCCCCCCCCCCCCcchHHHHHHHHHHHHHHH


Q ss_pred                eeeCCCCcceEEeehhccCCCCCchhheecceeeeeecCCCCCcccceeEEEEeeccccCC-CCCCCCChHH--------
Q Q_8363653_2     189    IIDANPDFKLRINAHAMWGDGIPDYYRFHNYKLFAESTDKNGNPLLDEVQIMTYDFSWSGS-APGPSTPLWW--------   259 (999)
Q Consensus       189    iidanpdfklrinahamwgdgipdyyrfhnyklfaestdkngnplldevqimtydfswsgs-apgpstplww--------   259 (999)
                             .+...+-+...+..............++..         ...+|.+.+|+|||.+..+ .+++..|+.|
T Consensus       140    ----~~~~~~~~~~~~~~~~~~~~~~~~~~~~~~~~--------~~~vD~v~~~~yd~~~~~~~~~~~~~~~~~l~~~~~~~~~~~~~~   207 (378)
T 5YUQ_B          140    ----GSKTILTTAVPTAPFNDENQQPSTKLDDNW--------ASTVDAFYIMAYDVNGIRDKNAGANAPLYYSPKVTGVE   207 (378)
T ss_dssp                ----CSSSEEEEEECSSCCBCTTSCBCSSCCTHH--------HHHCSEEEECCCSCSSTTSSBCCCSSCSCCCTTCCSSC
T ss_pred                ----CCCcEEEEEeeCCcccCCCCCCCcccchhH--------HHHCceeeeeccccCCCCCccCCCCCCCCCCCCCCCCC


Q ss_pred                HhcHHHHHHHhcCCCCCCCCcceEeeEEeCCCccccCCCCCCC
Q Q_8363653_2     260    MRNVAEWVKQCFDPSVNPNAKCTIDNVYLGGAGYGRRWPIHSD   302 (999)
Q Consensus       260    mrnvaewvkqcfdpsvnpnakctidnvylggagygrrwpihsd   302 (999)
                         ...+...++......+.+      +.+.+|-.-||+.|.....
T Consensus       208    ~~~~~~~~~~~~~~~~g~~~------~kl~~gl~~~g~~~~~~~~~   244 (378)
T 5YUQ_B          208    PTSGNDAVKAWIAAGIPA------EQLVLGVPFYGRVSKTLEP   244 (378)
T ss_dssp                SCCHHHHHHHHHHTTCCG------GGEEEEEESEEEEEEBSSC
T ss_pred                CCCHHHHHHHHHHcCCCH------HHEEEEeecccceEeeccCc
```

Template alignment | Template 3D Structure | PDBe

36. 3EBV_A Chinitase A; Chitinase A, ChiA, Glycosidase, structural genomics, UNKNOWN FUNCTION, Hydrolase, PSI-2, Protein Structure Initiative, New York SGX; HET: MSE; 1.5A {Streptomyces coelicolor}

Probability: 96.67%, E-value: 0.037, Score: 49.73, Aligned cols: 230, Identities: 14%, Similarity: 0.127, Template Neff: 11

```
Q ss_pred                cccceeeEEEEEEeecCchHhhHHHhcceeeEEEeeecccCCC-ccEEEEEecCCCCCCCcccccccCcccccCCCCCcc
Q Q_8363653_2       9    MTKQKRVMTWSLRIQNDDYRHDIERYGNKITSIGFHEFNVDAQ-GRIAIYRLGPQKDAQGSFTSFTDATTYDRYAPGRTL   87 (999)
Q Consensus         9    mtkqkrvmtwslriqnddyrhdierygnkitsigfhefnvdaq-griaiyrlgpqkdaqgsftsftdattydryapgrtl   87 (999)
                         |.+++|++.+--.-........++.....++.|-+--..+++. |.+.+......                      ....
T Consensus         1    ~~~~~~v~gy~~~~~~~~~~~~~~~~~~~~t~v~~~~~~~~~~~~~~~~~~~~~~~~~~~---------------------~~~~   59 (302)
```

```
T 3EBV_A            1       MSLKHAVTGYWQNFNNGATVQKISDVPSAYDIIAVAFADATTTPGAVTFNLDSAG--------------------LGGY   59 (302)
T ss_dssp                   -CCSSEEEEEEEESSCSSSCCCCGGGSCTTCSEEEEEEEEEECSSTTCEECCCCTTT--------------------TTSC
T ss_pred                   CCCCceEEEeecCCCCCCCCccHHHCCCCCCEEEEEEeecCCCCCcEEecccccc--------------------CCCC
```

```
Q ss_pred                   CcchHhhhhhcCccccceeeEeeeeCHHHHHHHhccchhhhHHHHhHHHHHHHHhhccCCCCccceeeeEEecccccccccc
Q Q_8363653_2       88      WPNYIEKDMKRWPHIEYYMQFVIFGPETVSRLLDSQTAQDNFINNLKTVVARFRKDQNGNDLGYTGIEIDCEGSFSDSKW   167 (999)
Q Consensus         88      wpnyiekdmkrwphieyymqfvifgpetvsrlldsqtaqdnfinnlktvvarfrkdqngndlgytgieidcegsfsdskw   167 (999)
                            -...+...+++. +..-..-++-+|-...+..+.+++.++.|++++...+.++.         +.||+||.|.
T Consensus         60      ~~~~~~~~i~~~-~~~~~~v~lsigg~~~~~~~~~~~~~~~~~~~~~i~~~~~~~~~---------~dGidld~e~-------   122 (302)
T 3EBV_A            60      TVDQFKADVRAK-QAAGKKVIISVGGEKGTVSVNSSASATNFANSVYSVMREYG---------FDGVDIDLEN-------   122 (302)
T ss_dssp                   CHHHHHHHHHHH-HHTTCEEEEEEEETTCCCCCCSHHHHHHHHHHHHHHHHHHHT---------CCEEEEEECS-------
T ss_pred                   CHHHHHHHHHHH-HHCCCEEEEEeCCCcCcccCCChHHHHhHHHHHHHHHHHHhC---------CCeEEEeCCC-------
```

```
Q ss_pred                   ccCCCCcchhHcHHHHhcceeeeeCCCCcceEEeehhccCCCCCchhheecceeeeeecCCCCCcccceeEEEEeecccc
Q Q_8363653_2       168     DTRAGDDVKYINLLKRIKNEVIIDANPDFKLRINAHAMWGDGIPDYYRFHNYKLFAESTDKNGNPLLDEVQIMTYDFSWS   247 (999)
Q Consensus         168     dtragddvkyinllkrikneviidanpdfklrinahamwgdgipdyyrfhnyklfaestdkngnplldevqimtydfsws   247 (999)
                               ..+...+.++|+.++...    ++++.+-+...+-+.+.....+......+...         +|-+.+|+|+....
T Consensus         123     ---~~~~~~~~~~~l~~l~~~~~~----~~~~~~it~~~~~~~~~~~~~~~~~~~~~~~~~~~~---------~D~i~~~~~y~~~~~~   186 (302)
T 3EBV_A            123     ---GLNPTYMTQALRALSAKA----GPDMILTMAPQTIDMQSTQGGYFQTALNVKDI---------LTVVNMQYYNSGTM   186 (302)
T ss_dssp                   ---CCCHHHHHHHHHHHHHHHH----CTTCEEEECCBGGGSSTTSHHHHHHHHTGGG---------CCEEEEECSSCCCE
T ss_pred                   ---CCCHHHHHHHHHHHHHHHH----CCCcEEEEcCCCcccccCCccHHHHHHHHhhh---------ccEEEEeccCCCCc
```

```
Q ss_pred                   CCCCCCCCCChHHHhcHHHHHHHhcCCCCCCCCcceEeeEEeCCCccccCCC
Q Q_8363653_2       248     GSAPGPSTPLWWMRNVAEWVKQCFDPSVNPNAKCTIDNVYLGGAGYGRRWP   298 (999)
Q Consensus         248     gsapgpstplwwmrnvaewvkqcfdpsvnpnakctidnvylggagygrrwp   298 (999)
                            ...++|..+.-+..............+.|.       .+.+|-..||+.|.
T Consensus         187     ~~~~~~~~~~~~~~~~~~~~~~~~~~~~~~~~~~~------kl~~gl~~~g~~~~   231 (302)
T 3EBV_A            187     LGCDGKVYAQGTVDFLTALACIQLEGGLAPS------QVGLGLPASTRAAG   231 (302)
T ss_dssp                   ECTTSCEECTTSHHHHHHHHHHHHTTTCCGG------GEEEEEESSTTSCS
T ss_pred                   cCCCCccccCCCccHHHHHHHHHHHcCCCCHH------HEEEeecCCCCccC
```

Template alignment | Template 3D Structure | PDBe

37. 5GZT_B Chitinase; HYDROLASE; HET: FMT; 2.1A {Paenibacillus sp. FPU-7}

Probability: 96.59%, E-value: 0.0056, Score: 66.49, Aligned cols: 192, Identities: 19%, Similarity: 0.263, Template Neff: 10.3

```
Q ss_pred                   chHhhhhhcCccccceeeEeeeeCHHHHHHHhccchhhhHHHHhHHHHHHHHhhccCCCCccceeeeEEecc--cccccccc
Q Q_8363653_2       90      NYIEKDMKRWPHIEYYMQFVIFGPETVSRLLDSQTAQDNFINNLKTVVARFRKDQNGNDLGYTGIEIDCE--GSFSDSKW   167 (999)
Q Consensus         90      nyiekdmkrwphieyymqfvifgpetvsrlldsqtaqdnfinnlktvvarfrkdqngndlgytgieidce--gsfsdskw   167 (999)
                            +++..--++.|++...+.+--+.....++++.+...+++|++++..++.++         |+.||+||.|  +.-.....
T Consensus         835     ~~~~~lk~~~~~~~k~llsigg~~~~~~~~~~~~~~~~~~~~~~~~r~~fi~~i~~~~~~~~~~---------~fDGvdid~e~p~~~~~~~~~~~   905 (1136)
T 5GZT_B            835     AYLKTLKQQNPSLKVLVSIGGWAAEGFSDAALTPETREELANSAIAFMHQY---------GFDGIDLDWEYPVYGAFGVI   905 (1136)
T ss_dssp                   HHHHHHHHHSTTCEEEEEEECTTCCCHHHHTSSHHHHHHHHHHHHHHHHHHT---------TCSEEEEECSCTTTHHHHTS
T ss_pred                   HHHHHHHHHCCCCEEEEEECCCcccchHHHhCCHHHHHHHHHHHHHHHHHHH---------CCCcEEEeccCCCCcCCCCCC
```

```
Q ss_pred                   ccCCCCcchhHcHHHHhcceeeeeCCCCcceEEeehhccCCCCCchhheecceeeeeecCCCCCcccceeEEEEeecccc
Q Q_8363653_2       168     DTRAGDDVKYINLLKRIKNEVIIDANPDFKLRINAHAMWGDGIPDYYRFHNYKLFAESTDKNGNPLLDEVQIMTYDFSWS   247 (999)
Q Consensus         168     dtragddvkyinllkrikneviidanpdfklrinahamwgdgipdyyrfhnyklfaestdkngnplldevqimtydfsws   247 (999)
                            .....|.-.|..+++.+++..--....-..+-.+-.-+..  ..+.+....+++..++.         +|.+.+|+|||..+
T Consensus         906     ~~~~~~~~~~~~~~~~~~l~~L~~~l~~~~~~~~~~~~~~~~~~~~~~--~~~~~~~~~~~~~~~~~l~~~--------vD~v~l~~yd~~~~~   975 (1136)
T 5GZT_B            906     KSRPEDKQNFTALLKLFREKLDVEGALHGKYYELAIASA--AAPIYINSVELDKIHQY--------LDYMSVMTYDYHGS   975 (1136)
T ss_dssp                   CCCTTHHHHHHHHHHHHHHHHHHHHHHHHHHTSCCEEEEEEC--CSHHHHHHSCHHHHGGG--------CSEEEECCCCCSCT
T ss_pred                   CCCHHHHHHHHHHHHHHHHHHHHHhhhhhhcCCeEEEEEEec--CCcccccCCHHHHHHH--------CCEEEEEcCCCCCC
```

```
Q ss_pred                   -CCCCCCCCChHHH------hcHHHHHHHhcCCCCCCCCcceEeeEEeCCCccccCCCCCCCCCCCC
Q Q_8363653_2       248     -GSAPGPSTPLWWM------RNVAEWVKQCFDPSVNPNAKCTIDNVYLGGAGYGRRWPIHSDDNWG   306 (999)
Q Consensus         248     -gsapgpstplwwm------rnvaewvkqcfdpsvnpnakctidnvylggagygrrwpihsddnwg   306 (999)
                             .+.+++..|++..      .++..+++......+.+      +.+.||-.-||+.|...+....+
T Consensus         976     ~~~~~~~~~~~~l~~~~~~~~~~~~~~~~~~~~~~~~~~g~p~------~KlvlGip~yg~~~~~~~~~~~~~~~~   1035 (1136)
T 5GZT_B            976     WESKTAHQASVYTSALSPGDFSADSVLTAYRKQGVPA------SKLVIGGAFYARGWVNVPNINHG   1035 (1136)
T ss_dssp                   TSSBCCCSSCSSCCTTSTTCCCHHHHHHHHHHTTCCG------GGEEEEEESBCEEESSCCSSGGG
T ss_pred                   CcCCCCCCCCCCCCCCCCCCCCHHHHHHHHHHcCCCH------HHEEEEEecceEeeeCCCccccCC
```

Template alignment | Template 3D Structure | PDBe

38. 6KST_A Family 18 chitinase; Chitin, Chitinase, Chitinolytic enzyme, Family 18 glycoside hydrolase (GH18), N-acetylglucosamine (GlcNAc), Hydrolysis, Transglycosylation, HYDROLASE; HET: MXE, SO4, GOL; 1.25A {Chitiniphilus shinanonensis}

Probability: 96.56%, E-value: 0.015, Score: 52.54, Aligned cols: 195, Identities: 17%, Similarity: 0.293, Template Neff: 11.6

```
Q ss_pred                   chHhhhhhcCccccceeeEeeeeC-HHHHHHHhccchhhhHHHHhHHHHHHHHhhccCCCCccceeeeEEecc--ccccccc
Q Q_8363653_2       90      NYIEKDMKRWPHIEYYMQFVIFG-PETVSRLLDSQTAQDNFINNLKTVVARFRKDQNGNDLGYTGIEIDCE--GSFSDSK   166 (999)
Q Consensus         90      nyiekdmkrwphieyymqfvifg-petvsrlldsqtaqdnfinnlktvvarfrkdqngndlgytgieidce--gsfsdsk   166 (999)
                            ..+.+--+..|++..++.+--.. .+....++.+.+.++.|++++..++..+         ++.||.||.|  +......
T Consensus         64      ~~~~~~~~~~~~p~~~~~~~~~~~g~~~~~~~~~~~~~~~~~~~~~~~~~i~~~~~~~~~---------~~dGi~id~e~~~~~~~~~~~~   134 (372)
T 6KST_A            64      TALRKAKKAHPHLRNLISVGGWSWSSGFSDAAATPEARKRFADSAVAFIRKY---------GFDGVDIDWEYPVEGGAEN   134 (372)
T ss_dssp                   HHHHHHHHHCTTCEEEEEEECSSSCTTHHHHTSSHHHHHHHHHHHHHHHHHHH---------TCSEEEEECSCSSSCSCTT
T ss_pred                   HHHHHHHHHCCCceEEEEECCCcccchHHHhcCHHHHHHHHHHHHHHHHHHh---------CCCcEEEcccCCCcCCccc
```

```
Q ss_pred                   cccCCCCcchhHcHHHHhcceeeeeCCCCcc-eEEeehhccCCCCCchhheecceeeeeecCCCCCcccceeEEEEeecc
Q Q_8363653_2       167     WDTRAGDDVKYINLLKRIKNEVIIDANPDFK-LRINAHAMWGDGIPDYYRFHNYKLFAESTDKNGNPLLDEVQIMTYDFS   245 (999)
Q Consensus         167     wdtragddvkyinllkrikneviidanpdfk-lrinahamwgdgipdyyrfhnyklfaestdkngnplldevqimtydfs   245 (999)
                            ......+...+..++++++...--......+ +.+.+...........|.+...           .+.+|.+.+|+|++.
```

```
T Consensus         135   ~~~~~~~~~~~~~~~~~l~~~l~~~~~~~~~~~~l~~~~~~~~~~~~~~~~~~~~-----------~~~~d~~~~~~y~~~   203 (372)
T 6KST_A            135   MKHRPEDKQNYTLLTRSLREALDTAGKADGKYYELTTAVWGNDKFIANTEMDKV-----------SRDFDFINVMSYDFN   203 (372)
T ss_dssp                 CCCCTTHHHHHHHHHHHHHHHHHHHHHHTSCCEEEEEEEESSTHHHHTSCHHHH-----------GGGCSEEEEECCCSS
T ss_pred                 cCCChhHHHHHHHHHHHHHHHHHHhcccCCceEEEEEeccCChHHHhccCHHHH-----------HHhCCEEEEEccccC


Q ss_pred                 ccCC----------------CCCCCCChHHHhcHHHHHHHHhcCCCCCCCCcceEeeEEeCCCccccCCCCCCC-------
Q Q_8363653_2       246   WSGS----------------APGPSTPLWWMRNVAEWVKQCFDPSVNPNAKCTIDNVYLGGAGYGRRWPIHSD-------   302 (999)
Q Consensus         246   wsgs----------------apgpstplwwmrnvaewvkqcfdpsvnpnakctidnvylggagygrrwpihsd-------   302 (999)
                          .+..                .|+|..++.|...+..|+++-..++          .+.+|=.-||+.|.....
T Consensus         204   ~~~~~~~~~~~~l~~~~~~~~~~~~~~~~~~~~~~~~~~~~g~~~~----------ki~~gi~~~g~~~~~~~~~~~~~~~~~   273 (372)
T 6KST_A            204   GTWNKFSGHNAPFVNDPAYDKPGIGKTFNVVSAVEAYLKAGVPAD----------KLVVGVPLYGYSWKGCAAGERNGEY   273 (372)
T ss_dssp                 CTTSSBCCCSSCSSCCTTCCCTTCCTTCSHHHHHHHHHHTTCCGG----------GEEEEEESEEEEESSCCCTTTTTTT
T ss_pred                 CCCCCCCCCCCCCCCCCCCCCCCCCCCCcHHHHHHHHHHcCCCHH----------HEEEceecceeeeecCCcCCCCCcc


Q ss_pred                 ---------CCCCceeehhHh
Q Q_8363653_2       303   ---------DNWGSTVTYRDL   314 (999)
Q Consensus         303   ---------dnwgstvtyrdl   314 (999)
                                     ..-+..++|+++
T Consensus         274   ~~~~~~~~~~~~~~~~~~~~~   294 (372)
T 6KST_A            274   QDCNGKGRGTWEDGNLDFTDI   294 (372)
T ss_dssp                 CBCCSBCCCSSBTTEEEHHHC
T ss_pred                 ccCCCCCCCcCCCceecHHHH
```

Template alignment | Template 3D Structure | PDBe

39. 4B1L_A LEVANASE; HYDROLASE, LEVAN; HET: FRU; 1.65A {BACILLUS SUBTILIS}

Probability: 96.53%, E-value: 0.27, Score: 36.98, Aligned cols: 141, Identities: 15%, Similarity: 0.261, Template Neff: 12.9

```
Q ss_pred                 eeCCCCcCcCCCccceEEEEeeEecCcEEEEEEEEeeCCcc---eeeEEee-cCCCCcEEEEEEcccCeEeeeeeccCcc
Q Q_8363653_2       696   RFRPNDPKNNGVTSGQLVLNYTYKTTNISCEVQFKVKSGRR---AGIRFAS-TGPGDGYVFLIDYQTQEAMMFYETAGSS   771 (999)
Q Consensus         696   rfrpndpknngvtsgqlvlnytykttniscevqfkvksgrr---agirfas-tgpgdgyvflidyqtqeammfyetagss   771 (999)
                          .+...+........+.-.+-......+...|+.++...+..   +|+.|.. .....+|.+.++.............+..
T Consensus         19    ~w~~~~~~~~~~~~~~~~~~~~~~~~~~~~~~~~~~~~~~~~~~~~~~~~~~~~~~~~~~~~~~~~~~~~~~~~~~~~~~   98 (165)
T 4B1L_A            19    TWADTIEGKQGRSDGDSFILSSASGSDFTYESDITIKDGNGRGAGALMFRSDKDAKNGYLANVDAKHDLVKFFKFENGAA   98 (165)
T ss_dssp                 EEEEETTEEEEEEEEEEEEEEEEEEESSEEEEEEEEEECSSTTCCEEEEEEEEECTTSSSEEEEEEEETTTTEEEEEEEEETTEE
T ss_pred                 EEEeeeCceecccCCceEEEEecCCCCEEEEEEEEEECCCCCceEEEEEEecCCCCceEEEEEeCCCCEEEEEEeeCCeE


Q ss_pred                 eeeEeeecccccCChhheEEEEEeeeCCeeeEEeccEEEEEecCCCCCCCCceeEEEecccceEEEEecc
Q Q_8363653_2       772   QLVASASLGDRRADYDELITLKVLVNNGKCRCYFGNVMFFMDMNLPHMSPGGIGFVATNCDAYLYKLSI   840 (999)
Q Consensus         772   qlvasaslgdrradydelitlkvlvnngkcrcyfgnvmffmdmnlphmspggigfvatncdaylyklsi   840 (999)
                          ..++.....   ..-++..+|++.+.+++.++|..+...+--... ...+|.+||.+.++.+.+-.+.+
T Consensus         99    ~~~~~~~~~~~---~~~~~~~~~~~~v~~~~~~~~~~~~~~~g~~~~~~~~~~-~~~~g~~g~~~~~~~~~~~~~~~~v   163 (165)
T 4B1L_A            99    SVIAEYKTP---IDVNKKYHLKTEAEGDRFKIYLDDRLVIDAHDS-VFSEGQFGLNVWDATAVFQNVTK   163 (165)
T ss_dssp                 EEEEEEECC---CCTTCEEEEEEEEETTEEEEEETTEEEEEEECC-SCCCEEEEEEEEEEEEEEEEEEE
T ss_pred                 EEEeeeecC---CCCCCceEEEEEEECCEEEEEECCEEEEEEecC-ccCCceeEEEEcCcEEEEEeEEE
```

Template alignment | Template 3D Structure | PDBe

40. 1GOI_B CHITINASE B; CHITIN DEGRADATION, HYDROLASE, GLYCOSIDASE; HET: SO4, GOL; 1.45A {SERRATIA MARCESCENS} SCOP: d.26.3.1, b.72.2.1, c.1.8.5

Probability: 96.5%, E-value: 0.0083, Score: 57.19, Aligned cols: 183, Identities: 19%, Similarity: 0.214, Template Neff: 11.2

```
Q ss_pred                 HhhhhhcCccccceeeEeeeeCH--------HHHHHHhccchhhhHHHHhHHHHHHHHhhccCCCCccceeeeEEeccccc
Q Q_8363653_2       92    IEKDMKRWPHIEYYMQFVIFGP--------ETVSRLLDSQTAQDNFINNLKTVVARFRKDQNGNDLGYTGIEIDCEGSFS   163 (999)
Q Consensus         92    iekdmkrwphieyymqfvifgp--------etvsrlldsqtaqdnfinnlktvvarfrkdqngndlgytgieidcegsfs   163 (999)
                          +..-.++.|++...+.+--...        .....++.+......|++++..++.++         ++.||+||.|..
T Consensus         78    ~~~lk~~~~~~~kv~~sigg~~~~~~~~~~~~~~~~~~~~~~~~~~~~~~~~~~~~~~~~~~---------~~DGidid~e~~--   146 (499)
T 1GOI_B            78    LTALKAHNPSLRIMFSIGGWYYSNDLGVSHANYVNAVKTPASRAKFAQSCVRIMKDY---------GFDGVNIDWEYP--   146 (499)
T ss_dssp                 HHGGGGGCTTCEEEEEEECHHHHSTTSTTHHHHHHHTSSHHHHHHHHHHHHHHHHH---------TCSEEEEECSCC--
T ss_pred                 HHHHHHHCCCCeEEEEEcCccccCCcccccchhHhcCCHHHHHHHHHHHHHHHHHH---------CCCeEEeCCcCC--


Q ss_pred                 ccccccCCCCcchhHcHHHHhcceeeee------CCCCcceEEeehhccCCCCCchhheecceeeeeecCCCCCccccee
Q Q_8363653_2       164   DSKWDTRAGDDVKYINLLKRIKNEVIID------ANPDFKLRINAHAMWGDGIPDYYRFHNYKLFAESTDKNGNPLLDEV   237 (999)
Q Consensus         164   dskwdtragddvkyinllkrikneviid------anpdfklrinahamwgdgipdyyrfhnyklfaestdkngnplldev   237 (999)
                                ...+...+..+++.++...--.      ..+.+.|.+...+........++.+....-.              +|.|
T Consensus         147   ------~~~~~~~~~~~~l~~l~~~l~~~~~~~~~~~~~~~~~ls~~~~~~~~~~~~~~~~~~~~~~~~~~-----------~D~v   209 (499)
T 1GOI_B            147   ------QAAEVDGFIAALQEIRTLLNQQTITDGRQALPYQLTIAGAGGAFFLSRYYSKLAQIVAP-----------LDYI   209 (499)
T ss_dssp                 ------CTTHHHHHHHHHHHHHHHHHHHHHTTCTTSCCEEEEEEESSHHHHTTTGGGHHHHHTT-----------CSEE
T ss_pred                 ------ChhhcCCHHHHHHHHHHHHHHHHhccccccccCCeeEEEEccccHHHHHHhHhHHHHHhhhc-----------CCEE


Q ss_pred                 EEEEeecccc-CCCCCCCCChHH------------------------------HhcHHHHHHHHhcCCCCCCCCcceEee
Q Q_8363653_2       238   QIMTYDFSWS-GSAPGPSTPLWW------------------------------MRNVAEWVKQCFDPSVNPNAKCTIDN   285 (999)
Q Consensus         238   qimtydfsws-gsapgpstplww------------------------------mrnvaewvkqcfdpsvnpnakctidn   285 (999)
                          .+|+||+...  ....++..||+-                               ..++...+++.....-.|..|
T Consensus         210   ~v~~yd~~~~~~~~~~~~~~~~~l~~~~~~~~~~~~~~~~~~~~~~~~~~~~~~~~~~~~~~~~~~~~~~~~~g~~~~K-----   284 (499)
T 1GOI_B            210   NLMTYDLAGPWEKVTNHQAALFGDAAGPTFYNALREANLGWSWEELTRAFPSPFSLTVDAAVQQHLMMEGVPSAK-----   284 (499)
T ss_dssp                 EEECCCSSCTTSSSCCCTTCSSBCTTSCCBCCGGGGSSCCCCHHHHHHHSCSSBCCCHHHHHHHHHHSTTCCGGG-----
T ss_pred                 EEEecCCCCCcccCCCcccccCCCCCchHHHHHHHhcCCCCHHHHHHhhCCCCcccCHHHHHHHHHHHHcCCCHHH-----


Q ss_pred                 EEeCCCccccCCCCCCCCCCCCc
Q Q_8363653_2       286   VYLGGAGYGRRWPIHSDDNWGS   307 (999)
Q Consensus         286   vylggagygrrwpihsddnwgs   307 (999)
                          +.||-.-|||.|..-+..+.+.
```

```
T Consensus       285      l~lg~~~yg~~~~~~~~~~~~~   306 (499)
T 1GOI_B          285      IVMGVPFYGRAFKGVSGGNGGQ   306 (499)
T ss_dssp                  EEEEEESEEEEEESCCTTTTTT
T ss_pred                  EEEeeeeceeEeeCCCCCCCCC
```

Template alignment | Template 3D Structure | PDBe

41. 4NZC_A Glycoside hydrolase family 18; Chitinase D, TIM barrel, Transglycosylation, hydrolase; HET: GOL, OMT; 1.45A {Serratia proteamaculans}

Probability: 96.49%, E-value: 0.023, Score: 51.14, Aligned cols: 191, Identities: 19%, Similarity: 0.223, Template Neff: 11.9

```
Q ss_pred                  HhhhhhcCcccceeeEeeeeCHHHHHHHhccchhhhHHHHhHHHHHHHhhccCCCCccceeeeEEecc--cccccccccc
Q Q_8363653_2      92      IEKDMKRWPHIEYYMQFVIFGPETVSRLLDSQTAQDNFINNLKTVVARFRKDQNGNDLGYTGIEIDCE--GSFSDSKWDT   169 (999)
Q Consensus        92      iekdmkrwphieyymqfvifgpetvsrlldsqtaqdnfinnlktvvarfrkdqngndlgytgieidce--gsfsdskwdt   169 (999)
                           +.+..++.|+++..+-+--++......++...+....|+.++..++.+.         ++.||.+|.|  +......+..
T Consensus       78      ~~~~k~~~~~~~~~~~~~~igg~~~~~~~~~~~~~~~~~~~~~~~~~~~~~~~~~~~~~~~~~~---------~~dGv~~d~~~~~~~~~~~~~~~~~~~   148 (398)
T 4NZC_A          78      LPVLRKQNPELKVLLSVGGWGARGFSGAAATAESRAVFIRSVQQVIKQY---------HLDGIDLDWEYPVNGAWGLVES   148 (398)
T ss_dssp                  HHHHHHHCTTCEEEEEEECTTCCCHHHHHSSHHHHHHHHHHHHHHHHHH---------TCSEEEEECSCTTTHHHHTSCC
T ss_pred                  HHHHHHHCCCCEEEEEeCCCcccchHHHhCCHHHHHHHHHHHHHHHHHc---------CCCcEEEcCCCCCCCCCCCCCC
```

```
Q ss_pred                  CCCCcchhHcHHHHhcceeeeeCCCCcceEEeehhccCCCCCchhheecceeeeeecCCCCCccccceeEEEEeec-----
Q Q_8363653_2      170     RAGDDVKYINLLKRIKNEVIIDANPDFKLRINAHAMWGDGIPDYYRFHNYKLFAESTDKNGNPLLDEVQIMTYDF-----   244 (999)
Q Consensus        170     ragddvkyinllkrikneviidanpdfklrinahamwgdgipdyyrfhnyklfaestdkngnplldevqimtydf-----   244 (999)
                           ...+...++++++.++...    .+++.+.+...+......+.|            .-+.-...+|.+.+|+|++
T Consensus       149     ~~~~~~~~~~~~~~~~l~~~~----~~~~~~l~~~~~~~~~~~~~~~~~~~------------~~~~l~~~vD~v~v~~y~~~~~~~~   212 (398)
T 4NZC_A          149     QPADRANFTLLLAELHKAL----DKGKLLTIAVGANVKSPQEWV------------DVKGIAPYLDYINLMTYDMAYGTQ   212 (398)
T ss_dssp                  CTTHHHHHHHHHHHHHHHHS----CTTCEEEEEECSSTHCCCCCC------------CHHHHGGGCSEEEECCCCCCSTTC
T ss_pred                  ChhHHHHHHHHHHHHHHHHh----cCCCcEEEEeecCCCCccccC------------CHHHHHHHCCEEEEEcccccCCcc
```

```
Q ss_pred                  --------cccCCCCCCCCChHHHhcHHHHHHHhcCCCCCCCCcceEeeEEeCCCccccCCCCCCCCC------------
Q Q_8363653_2      245     --------SWSGSAPGPSTPLWWMRNVAEWVKQCFDPSVNPNAKCTIDNVYLGGAGYGRRWPIHSDDN------------   304 (999)
Q Consensus        245     --------swsgsapgpstplwwmrnvaewvkqcfdpsvnpnakctidnvylggagygrrwpihsddn------------   304 (999)
                                   .-....+.+....+....+..|++.-+.++          .+.+|-.-||+.|.+.++..
T Consensus       213     ~~~~~~~~~~~~~~~~~~~~~~~~~~~~~~~~~~~~~~~~~~~~~~~~~~~~~~~~~~~~~~g~~~~----------kl~~gl~~~g~~~~~~~~~~~~~~~~~~~~~~~~~~~~~~~~~~~~   282 (398)
T 4NZC_A          213     YFNSNLYDSKQWPTVAAADRYSANFVVDNYLAAGLKPA----------QLNLGIGFYGRVPKRATEPGIDWDKADAAKNP   282 (398)
T ss_dssp                  CCSSCSSCCSSSCCCSGGGCCCHHHHHHHHHHTTCCGG----------GEEEEEESEEECCGGGTSCBCCTTSTTGGGSC
T ss_pred                  cccccccccCCCCcchhhcccCHHHHHHHHHHCCCCHH----------HEEEcceeeccccccCCCCCCcccccccccCC
```

```
Q ss_pred                  ----------------------CCceeehhHhccc
Q Q_8363653_2      305     ----------------------WGSTVTYRDLVDW   317 (999)
Q Consensus        305     ----------------------wgstvtyrdlvdw   317 (999)
                                                 -...++|.++...
T Consensus       283     ~~~~~~~~~~~~~~~~~~~~~~~~~~~~~~~~~~~~~~~~~~~~~~~~~~~~~~~~y~~i~~~   317 (398)
T 4NZC_A          283     VTQPYFTARETAVFKAMGLDLTKDSYFKYNDIVSK   317 (398)
T ss_dssp                  SBCCCCCHHHHHHHHHTTCCTTTCCEEEHHHCCCC
T ss_pred                  CCCCccccccchhhhhcCCCCCCCccccHHHHHHH
```

Template alignment | Template 3D Structure | PDBe

42. 1ITX_A Glycosyl Hydrolase; Alpha-Beta (TIM) Barrel, HYDROLASE; HET: GOL; 1.1A {Bacillus circulans} SCOP: c.1.8.5, d.26.3.1

Probability: 96.33%, E-value: 0.017, Score: 54.39, Aligned cols: 175, Identities: 18%, Similarity: 0.299, Template Neff: 10.9

```
Q ss_pred                  HhhhhhcCcccceeeEeeeeC-HHHHHHHhccchhhhHHHHhHHHHHHHhhccCCCCccceeeeEEecccccc--ccccc
Q Q_8363653_2      92      IEKDMKRWPHIEYYMQFVIFG-PETVSRLLDSQTAQDNFINNLKTVVARFRKDQNGNDLGYTGIEIDCEGSFS--DSKWD   168 (999)
Q Consensus        92      iekdmkrwphieyymqfvifg-petvsrlldsqtaqdnfinnlktvvarfrkdqngndlgytgieidcegsfs--dskwd   168 (999)
                           +..--++.|++..++.+--.. ....+.++.+....+.|++++..++..+         ++.||+||-|....  ..-..
T Consensus       113     ~~~lk~~~p~~kvl~sigg~~~~~~~~~~~~~~~~~~~~~~~~~~~~~~~~~~~~~~~~~~~~~~~~~~~~~~~~~~~~~~~~~~~~~~~~~~~~~~~~~~~~~~~i~~~~~~~---------~~DGvdld~e~~~~~~~~~~~   183 (419)
T 1ITX_A          113     LNKLKQTNPNLKTIISVGGWTWSNRFSDVAATAATREVFANSAVDFLRKY---------NFDGVDLDWEYPVSGGLDGNS   183 (419)
T ss_dssp                  HHHHHHHSTTCEEEEEEECSSSCTTHHHHHTSHHHHHHHHHHHHHHHHHH---------TCSEEEEECSCSSSCSCTTSC
T ss_pred                  HHHHHHHCCCCeEEEEECCCccchhHHHHhCCHHHHHHHHHHHHHHHHHc---------CCCcEEEcccCCCcCCCCCCC
```

```
Q ss_pred                  cCCCCcchhHcHHHHhcce-----------eeeeCCCCcceEEeehhccCCCCCchhheecceeeeeecCCCCCcccee
Q Q_8363653_2      169     TRAGDDVKYINLLKRIKNE-----------VIIDANPDFKLRINAHAMWGDGIPDYYRFHNYKLFAESTDKNGNPLLDEV   237 (999)
Q Consensus        169     tragddvkyinllkrikne-----------viidanpdfklrinahamwgdgipdyyrfhnyklfaestdkngnplldev   237 (999)
                           ....+...|..++++++++           ++|...+...........+..-                     .+.+|-+
T Consensus       184     ~~~~~~~~~~~~~~~~l~~~l~~~~~~~~~~~~~~~~~~l~~~~~~~~~~~~~~~~~~~~~~~~~l---------------------~~~~D~v   242 (419)
T 1ITX_A          184     KRPEDKQNYTLLLSKIREKLDAAGAVDGKKYLLTIASGASATYAANTELAKI---------------------AAIVDWI   242 (419)
T ss_dssp                  CCTTHHHHHHHHHHHHHHHHHHHHHHHTSCCEEEEEECCSHHHHHTSCHHHH---------------------HHHSSEE
T ss_pred                  CCHHHHHHHHHHHHHHHHHHHHHHccccCCCeEEEEEEccCChhhcccCCHHHH---------------------HHHCcEE
```

```
Q ss_pred                  EEEEeecccc-CCCCCCCCCh-----------------HHHhcHHHHHHHhcCCCCCCCCcceEeeEEeCCCccccCCCC
Q Q_8363653_2      238     QIMTYDFSWS-GSAPGPSTPL-----------------WWMRNVAEWVKQCFDPSVNPNAKCTIDNVYLGGAGYGRRWPI   299 (999)
Q Consensus        238     qimtydfsws-gsapgpstpl-----------------wwmrnvaewvkqcfdpsvnpnakctidnvylggagygrrwpi   299 (999)
                           .+|+|++.+. ...+++..|+                 +....+..|++...+++          .+.+|-.-||+.|..
T Consensus       243     ~~~~y~~~~~~~~~~~~~~~~~~~~~~~~~l~~~~~~~~~~~~~~~~~~~~~~~~~~~~~~~~~~~~~~~~~~~~~~~~g~~~~----------Klv~gl~~~g~~~~~   312 (419)
T 1ITX_A          243     NIMTYDFNGAWQKISAHNAPLNYDPAASAAGVPDANTFNVAAGAQGHLDAGVPAA----------KLVLGVPFYGRGWDG   312 (419)
T ss_dssp                  EECCCCSSCTTSSBCCCSSCSSCCHHHHHTTCTTTTTCSHHHHHHHHHHHTCCGG----------GEEEEEESEEEEEES
T ss_pred                  EEecccccCCcccccCCCCccCCChhhhcCCCCCcccccHHHHHHHHHHcCCCHH----------HeeEecccceeeeec
```

```
Q ss_pred                  CCCCCCC
Q Q_8363653_2      300     HSDDNWG   306 (999)
Q Consensus        300     hsddnwg   306 (999)
                           .+....+
```

```
T Consensus     313     ~~~~~~~   319 (419)
T 1ITX_A        313     CAQAGNG   319 (419)
T ss_dssp               CCSGGGG
T ss_pred               CCCCCCC
```

Template alignment | Template 3D Structure | PDBe

43. 3G6M_A Chitinase; chitinase CrChi1, inhibitor, caffeine, Glycosidase, HYDROLASE-HYDROLASE INHIBITOR complex; HET: CFF; 1.65A {Bionectria ochroleuca}

Probability: 96.32%, E-value: 0.047, Score: 51.31, Aligned cols: 222, Identities: 17%, Similarity: 0.268, Template Neff: 10.9

```
Q ss_pred               eeEEEeeecccCCCccEEEEEecCCCCCCCcccccccCccccCCCCCCccCcchHhhhhhcCccccceeEeeeeC-HHHH
Q Q_8363653_2    38     ITSIGFHEFNVDAQGRIAIYRLGPQKDAQGSFTSFTDATTYDRYAPGRTLWPNYIEKDMKRWPHIEYYMQFVIFG-PETV   116 (999)
Q Consensus      38     itsigfhefnvdaqgriaiyrlgpqkdaqgsftsftdattydryapgrtlwpnyiekdmkrwphieyymqfvifg-petv   116 (999)
                        +|.|-+..+.++..|.+.      .......-........+.-....-.-+...|..-.++.|.+..++.+--.. .+..
T Consensus      47     ~t~i~~~~~~~~~~~~~~~~------~~~~~~~~~~~~~~~~~~~~~~~~~~~~~~i~~lk~~~~~kvl~si~g~~~~~~~~   120 (406)
T 3G6M_A         47     ILHVLYSFMNLRVDGTVY------SGDTYADLEKHYSDDSWNDIGTNAYGCVKQLYKLKKANRSLKIMLSIGGWTWSTNF   120 (406)
T ss_dssp               CSEEEEEEEEECTTSCEE------ESCHCCCCCCCCTTCCSCCSSSCCCHHHHHHHHHHHHCTTCEEEEEEECSSSCTTH
T ss_pred               CCEEEEeccEecCCCeEE------cCCchHHHHhhcCCCCccccCccccchHHHHHHHHHHCCCCeEEEEEcCcccccch


Q ss_pred               HHHhccchhhhHHHHhHHHHHHHHhhccCCCCccceeeeEEecccccccccccCCCCcchhHcHHHHhcceeee-----e
Q Q_8363653_2   117     SRLLDSQTAQDNFINNLKTVVARFRKDQNGNDLGYTGIEIDCEGSFSDSKWDTRAGDDVKYINLLKRIKNEVII-----D   191 (999)
Q Consensus     117     srlldsqtaqdnfinnlktvvarfrkdqngndlgytgieidcegsfsdskwdtragddvkyinllkriknevii-----d   191 (999)
                        +.++.++..++.|++++..++..         .++.||.||.|..       ....+...+..++++++...--      .
T Consensus     121     ~~~~~~~~~~~~~~~~i~~~~~~~---------~~~DGi~ld~e~~-------~~~~~~~~~~~~~~l~~l~~~l~~~~~~~~~~   184 (406)
T 3G6M_A        121     PAAASTEATRATFAKTAVEFMKD---------WGFDGIDVDWEYP-------ASETDANNMVLLLQRVRQELDSYSATYA   184 (406)
T ss_dssp               HHHTSSHHHHHHHHHHHHHHHHH---------HTCSEEEEECSCC-------CSHHHHHHHHHHHHHHHHHHHHHHHHHS
T ss_pred               HHHhCCHHHHHHHHHHHHHHHHH---------hCCCcEEEeCcCC-------CCccchhhHHHHHHHHHHHHHHhhhhhc


Q ss_pred               CCCCcceEEeehhccCCCCCchhheecceeeeeecCCCCCcccceeEEEEeeccc-------------cCCCCCCCCChH
Q Q_8363653_2   192     ANPDFKLRINAHAMWGDGIPDYYRFHNYKLFAESTDKNGNPLLDEVQIMTYDFSW-------------SGSAPGPSTPLW   258 (999)
Q Consensus     192     anpdfklrinahamwgdgipdyyrfhnyklfaestdkngnplldevqimtydfsw-------------sgsapgpstplw   258 (999)
                        .++.+.|.+....     -+..+...++...++.        +|.+.+|+|++.+                ..+...+..+.|
T Consensus     185     ~~~~~~l~~~~~~~-----~~~~~~~~~~~~~~l~~~~--------~D~v~i~~y~~~~~~~~~~~~~~~~~~~~~l~~~~~~~~~~~~~~~~~   251 (406)
T 3G6M_A        185     NGYHFQLSIAAPA-----GPSHYNVLKLAQLGSV--------LDNINLMAYDYAGSWDSVSGHQTNLYPSTSNPSSTPFS   251 (406)
T ss_dssp               TTCCCEEEEEEEC-----SHHHHTTSCHHHHHHH--------CSEEEEECCCCSSTTSSSCCCSSCSSCCSSCGGGCSCC
T ss_pred               CCceeEEEEEeCC-----ChhhhcccCHHHHHHh--------CCEEEEEccCCCCCccccCCCCCCCCCCCCCCCCCCCCC


Q ss_pred               HHhcHHHHHHHhcCCCCCCCCcceEeeEEeCCCccccCCCCCCCC
Q Q_8363653_2   259     WMRNVAEWVKQCFDPSVNPNAKCTIDNVYLGGAGYGRRWPIHSDDN   304 (999)
Q Consensus     259     wmrnvaewvkqcfdpsvnpnakctidnvylggagygrrwpihsddn   304 (999)
                        +...+..|++..+++           ..+.+|-..||+.|.......
T Consensus     252     ~~~~~~~~~~~gi~~----------~Klv~gi~~~g~~~~~~~~~~~~~   287 (406)
T 3G6M_A        252     TKAAVDAYIAAGVPA----------SKIILGMPIYGRAFVGTDGPG   287 (406)
T ss_dssp               HHHHHHHHHHTTCCG----------GGEEEEEESEEEEEESCSSTT
T ss_pred               HHHHHHHHHHCCCCH----------HHEEEeccccEEEeCCCCCC
```

Template alignment | Template 3D Structure | PDBe

44. 1EDQ_A CHITINASE A; BETA-ALPHA (TIM) BARREL, HYDROLASE; 1.55A {Serratia marcescens} SCOP: c.1.8.5, b.1.18.2, d.26.3.1

Probability: 96.3%, E-value: 0.018, Score: 56.75, Aligned cols: 192, Identities: 18%, Similarity: 0.257, Template Neff: 10.6

```
Q ss_pred               HhhhhhcCccccceeeEeeeeCHHHHHHHhccchhhhHHHHhHHHHHHHHhhccCCCCccc-eeeeEEecccccccccc-
Q Q_8363653_2    92     IEKDMKRWPHIEYYMQFVIFGPETVSRLLDSQTAQDNFINNLKTVVARFRKDQNGNDLG-YTGIEIDCEGSFSDSKWDT-   169 (999)
Q Consensus      92     iekdmkrwphieyymqfvifgpetvsrlldsqtaqdnfinnlktvvarfrkdqngndlg-ytgieidcegsfsdskwdt-   169 (999)
                        |..--++.|++...+.+--.........+.+...++.|++++..++..+         | +.||+||.|..-...-...
T Consensus     233     i~~lk~~~~p~~kvl~sigg~~~~~~~~~~~~~~~~~~~~~~~~~~~~~~~~~~~---------~~~DGidlD~e~~~~~~~~~~~~~~~   303 (540)
T 1EDQ_A        233     LMALKQAHPDLKILPSIGGWTLSDPFFFMGDKVKRDRFVGSVKEFLQTW---------KFFDGVDIDWEFPGGKGANPNL   303 (540)
T ss_dssp               HHHHHHHCTTCEEEEEEECSSSCGGGGGTTSHHHHHHHHHHHHHHHHHHC---------TTCCEEEEECSCTTSCSSCTTC
T ss_pred               HHHHHHHCCCCEEEEEeCCCccCCChHhcCCHHHHHHHHHHHHHHHHHHc---------CCCCeEEEeeeCCCCCCCCCCC


Q ss_pred               -CCCCcchhHcHHHHhcceeeeeC---CCCcceEEeehhccCCCCC-chhheecceeeeeecCCCCCcccceeEEEEeec
Q Q_8363653_2   170     -RAGDDVKYINLLKRIKNEVIIDA---NPDFKLRINAHAMWGDGIP-DYYRFHNYKLFAESTDKNGNPLLDEVQIMTYDF   244 (999)
Q Consensus     170     -ragddvkyinllkriknevviida---npdfklrinahamwgdgip-dyyrfhnyklfaestdkngnplldevqimtydf   244 (999)
                         ...+...+..+++.+++..--..   ++.+.|.+...+-....-. ++-....+                 +|-+.+|+||+
T Consensus     304     ~~~~~~~~~~~~~~l~~l~~~~l~~~~~~~~~~~~~~~~~~~~~~~~~~~~~ls~~~~~~~~~~~~~~~~~~--------------vD~i~~~~~y~~   369 (540)
T 1EDQ_A        304     GSPQDGETYVLLMKELRAMLDQLSVETGRKYELTSAISAGKDKIDKVAYNVAQNS--------------MDHIFLMSYDF   369 (540)
T ss_dssp               CCTTHHHHHHHHHHHHHHHHHHHHHHHTCCCEEEEEEECSHHHHTTSCHHHHGGG--------------CSEEEEECCCS
T ss_pred               CCccHHHHHHHHHHHHHHHHHHHHHhhCCeEEEEEEEecCChhHhhhccHHHHHhh--------------CCEEEEEeccc


Q ss_pred               cccCCCC-------------CCCCChHHHhcHHHHHHHhcCCCCCCCCcceEeeEEeCCCccccCCCCCCCCCCCce---
Q Q_8363653_2   245     SWSGSAP-------------GPSTPLWWMRNVAEWVKQCFDPSVNPNAKCTIDNVYLGGAGYGRRWPIHSDDNWGST---   308 (999)
Q Consensus     245     swsgsap-------------gpstplwwmrnvaewvkqcfdpsvnpnakctidnvylggagygrrwpihsddnwgst---   308 (999)
                        .+.-...             +|..+.++...+..|+++.+.+             ..+.+|-..||+.|...+...-+..
T Consensus     370     ~~~~~~~~~~~~~~~~~~~~~~~~~~~~~~~~~~~~~~~~~~~~~~~~~~gi~~----------~Klv~g~~~yg~~~~~~~~~~~~~~~~~~~~~~   439 (540)
T 1EDQ_A        370     YGAFDLKNLGHQTALNAPAWKPDTAYTTVNGVNALLAQGVKP----------GKIVVGTAMYGRGWTGVNGYQNNIPFTG   439 (540)
T ss_dssp               SCTTCSSSCCCSSCSSCCTTCTTCSCCHHHHHHHHHHHTCCG----------GGEEEEEESEEEEEESCBSCSTTCGGGS
T ss_pred               cCCCCccccCCCCCCCCCCCCCCCcccHHHHHHHHHHCCCCH----------HHEEEeeecceeeeecCCCCCCCCcCCC


Q ss_pred               --------------eehhHhcc
Q Q_8363653_2   309     --------------VTYRDLVD   316 (999)
Q Consensus     309     --------------vtyrdlvd   316 (999)
                                      .+|+++..
```

```
T Consensus        440     ~~~~~~~~~~~~~~~~~~ei~~   461 (540)
T 1EDQ_A           440     TATGPVKGTWENGIVDYRQIAG   461 (540)
T ss_dssp                  BCSEECCCSSBTTEEEHHHHHH
T ss_pred                  CCCCCCCCccccCCeeeHHHHHH
```

Template alignment | Template 3D Structure | PDBe

45. 6T9M_AAA Peroxiredoxin; Spore Coat, Chitinase, Colonisation Factor, STRUCTURAL PROTEIN; HET: PEG, 1PE; 1.3A {Peptoclostridium difficile (strain 630)}

Probability: 96.3%, E-value: 0.021, Score: 53.66, Aligned cols: 172, Identities: 20%, Similarity: 0.289, Template Neff: 10.7

```
Q ss_pred                  HhhhhhcCcccceeeEeeeeCHHHHHHHhccchhhhHHHHhHHHHHHHHhccCCCCccceeeeEEecc-ccccccccccC
Q Q_8363653_2      92      IEKDMKRWPHIEYYMQFVIFGPETVSRLLDSQTAQDNFINNLKTVVARFRKDQNGNDLGYTGIEIDCE-GSFSDSKWDTR   170 (999)
Q Consensus        92      iekdmkrwphieyymqfvifgpetvsrlldsqtaqdnfinnlktvvarfrkdqngndlgytgieidce-gsfsdskwdtr   170 (999)
                           +....++.|.+...+.+--+......+++.+.+..+.|++++..++..+         |+.||+||.| ..........
T Consensus        100     ~~~l~~~~~~~kvllsigg~~~~~~~~~~~~~~~~~~~~~~i~~~~~~~---------~~DGidid~e~~~~~~~~~~~~   170 (386)
T 6T9M_AAA         100     LLALKGEKPSLKVIVAIGGWGAEGFSDAALTPTSRYNFARQVNQMINEY---------ALDGIDIDWEYPGSSASGITSR   170 (386)
T ss_dssp                  HHHGGGTCTTCEEEEEEECTTCCCHHHHTSSHHHHHHHHHHHHHHHHHH---------TCSEEEEECSCTTCCTTSCCCC
T ss_pred                  HHHHHhCCCCEEEEEECCCcccchhHHcCCHHHHHHHHHHHHHHHHHHc---------CCCeEEEEEeCCCCCCCCCCCC

Q ss_pred                  CCCcchhHcHHHHhcceeeeeCCCCcceEEeehhccCCCCCchhheecceeeeeecCCCCCccccceeEEEEeeccccCCC
Q Q_8363653_2      171     AGDDVKYINLLKRIKNEVIIDANPDFKLRINAHAMWGDGIPDYYRFHNYKLFAESTDKNGNPLLDEVQIMTYDFSWSGSA   250 (999)
Q Consensus        171     agddvkyinllkrikneviidanpdfklrinahamwgdgipdyyrfhnyklfaestdkngnplldevqimtydfswsgsa   250 (999)
                           ..+.-.+..+++.++...    .+...|.+...+.-..-...|              ....-...+|-+.+|+|++.+....
T Consensus        171     ~~~~~~~~~~l~~l~~~l----~~~~~l~~~~~~~~~~~~~~~~~------------~~~~l~~~vD~v~i~~y~~~~~~~~   234 (386)
T 6T9M_AAA         171     PQDRENFTLLLTAIRDVI----GDDKWLSVAGTGDRGYINSSA------------EIDKIAPIIDYFNLMSYDFTAGETG   234 (386)
T ss_dssp                  TTHHHHHHHHHHHHHHHH----CTTSEEEEEECCSHHHHHHTC------------CHHHHTTTCSEEEECCCCTTTTCCG
T ss_pred                  HHHHHHHHHHHHHHHHHH----CCCceEEEEEeCCcccccCcc------------cHHHHHHHCCEEEEEeCccCCCCCC

Q ss_pred                  CCCCC------------ChHHHhcHHHHHHHh-cCCCCCCCCcceEeeEEeCCCccccCCC
Q Q_8363653_2      251     PGPST------------PLWWMRNVAEWVKQC-FDPSVNPNAKCTIDNVYLGGAGYGRRWP   298 (999)
Q Consensus        251     pgpst------------plwwmrnvaewvkqc-fdpsvnpnakctidnvylggagygrrwp   298 (999)
                           ++...            +..++....+.+... +++          +.+.+|-..||+.|.
T Consensus        235     ~~~~~~~~~~l~~~~~~~~~~~~~~~~~~~~~~~~~gi~~----------~Kiv~gi~~~g~~~~   285 (386)
T 6T9M_AAA         235     PNGRKHQANLFDSDLSLPGYSVDAMVRNLENAGMPS----------EKILLGIPFYGRLGA   285 (386)
T ss_dssp                  GGGSSCSSCSSCCTTSCTTCSHHHHHHHHHHTTCCG----------GGEEEEEESEEEETT
T ss_pred                  CCCCcCCcccCCCCCCCCCCcHHHHHHHHHHCCCCH----------HHEEEEEecccccCC
```

Template alignment | Template 3D Structure | PDBe

46. 3OA5_B Chi1; Tim Barrel, Chitinase, HYDROLASE; HET: 2PE, GOL; 1.74A {Yersinia}

Probability: 96.28%, E-value: 0.021, Score: 59.68, Aligned cols: 190, Identities: 18%, Similarity: 0.239, Template Neff: 9

```
Q ss_pred                  HhhhhhcCcccceeeEeeeeC-HHHHHHHhccchhhhHHHHhHHHHHHHHhccCCCCccceeeeEEecccccccccccc-
Q Q_8363653_2      92      IEKDMKRWPHIEYYMQFVIFG-PETVSRLLDSQTAQDNFINNLKTVVARFRKDQNGNDLGYTGIEIDCEGSFSDSKWDT-   169 (999)
Q Consensus        92      iekdmkrwphieyymqfvifg-petvsrlldsqtaqdnfinnlktvvarfrkdqngndlgytgieidcegsfsdskwdt-   169 (999)
                           +.+--++.|++.-.+-+-=..  .+..+.++.+....++|++++..++.++.        ++.||+||.|..-...   .
T Consensus        228     l~~lk~~~p~lkvllSiGG~~~s~~f~~~~~~~~~~~~r~~f~~si~~~l~~~~--------~fDGIDlDwE~p~~~~---~~   296 (574)
T 3OA5_B          228     FRLLHEADKELEFSLSIGGWSMSGLFSEIAKDEILRTNFVEGIKDFFQRFP--------MFSHLDIDWEYPGSIG---AG   296 (574)
T ss_dssp                  HHHHHHHCTTCEEEEEEECGGGCTTHHHHHHCHHHHHHHHHHHHHHHHHCT--------TCCEEEEECSCTTSCT---TT
T ss_pred                  HHHHHHHCCCCEEEEEEeCcccccchHHHHhcCHHHHHHHHHHHHHHHHHHcC--------CCCeEEEEccCCCCCC---CC

Q ss_pred                  ---CCCCcchhHcHHHHhcceeeeeCCCCcceEEeehhccCCCCCchhheecceeeeeecCCC-CCccccceeEEEEeecc
Q Q_8363653_2      170     ---RAGDDVKYINLLKRIKNEVIIDANPDFKLRINAHAMWGDGIPDYYRFHNYKLFAESTDKN-GNPLLDEVQIMTYDFS   245 (999)
Q Consensus        170     ---ragddvkyinllkrikneviidanpdfklrinahamwgdgipdyyrfhnyklfaestdkn-gnplldevqimtydfs   245 (999)
                              ...+...|..|++.++...--   +++.|-+...+.             .........-.. =...+|.+.+|+|||.
T Consensus        297     ~~~~~~d~~~~~~li~eLr~~l~~---~~~~Lsiav~~~-------------~~~~~~~~~~~~l~~~vD~i~vmtYd~~   360 (574)
T 3OA5_B          297     NPNSPDDGANFAILIQQITDAKIS---NLKGISIASSAD-------------PAKIDAANIPALMDAGVTGINLMTYDFF   360 (574)
T ss_dssp                  CCCCTTHHHHHHHHHHHHHHTCCT---TCCEEEEEECSS-------------HHHHHHTTHHHHHTTTCCEEEECCCCCC
T ss_pred                  CCCChHHHHHHHHHHHHHHHHhhh---cCCeEEEEecCC-------------HHHHhhccchHHHHhcCCEEEEEecccc

Q ss_pred                  cc-CCCCCCCCChHHHhc-------HHHHHHHhcC-CCCCCCCcceEeeEEeCCCccccCCCCCCCCCCC----------
Q Q_8363653_2      246     WS-GSAPGPSTPLWWMRN-------VAEWVKQCFD-PSVNPNAKCTIDNVYLGGAGYGRRWPIHSDDNWG----------   306 (999)
Q Consensus        246     ws-gsapgpstplwwmrn-------vaewvkqcfd-psvnpnakctidnvylggagygrrwpihsddnwg----------   306 (999)
                           ..  ....++.+||..-..       +...|....+ ..+.++      .+.+|-+.|||.|.........
T Consensus        361     g~~~~~~~~~~s~L~~~~~~~~~~~~~~si~~~v~~~~~~~~~gvp~~------KlvlGvp~yg~~~~~~~~~~~~~~~~~~~~~g~~   434 (574)
T 3OA5_B          361     TLGDGKLSHHTNIYRDPSDVYSKYSIDDAVTHLIDEKKVDPK------AIFIGYAGYTRNAKNATITTSIPSEEALKGTY   434 (574)
T ss_dssp                  CSSSSBCCCSSCSCCCTTCSSSCCCHHHHHHCCCCCTCCCGG------GEEEEEESBCEEESSEEECCSSTTTSCCCEEE
T ss_pred                  CCCCCCcccCCCCCCCccccccCCCHHHHHHHHHHhcCCCHH------hEEEEeeecccccccCccccCCCchHhhcCcc

Q ss_pred                  ------------ceeehhHh
Q Q_8363653_2      307     ------------STVTYRDL   314 (999)
Q Consensus        307     ------------stvtyrdl   314 (999)
                                       ..++|.++
T Consensus        435     ~~~~~~~~~~t~~~g~~~y~ei   454 (574)
T 3OA5_B          435     TDANQTLGSFEYSVLEWTDI   454 (574)
T ss_dssp                  SCCTTCCBSSBTTBCBHHHC
T ss_pred                  cCcccccccccchhhHHHH
```

Template alignment | Template 3D Structure | PDBe

47. 4B1M_B LEVANASE; HYDROLASE, CBM66; HET: FRU; 1.1A {BACILLUS SUBTILIS}

Probability: 96.27%, E-value: 0.52, Score: 37.55, Aligned cols: 131, Identities: 17%, Similarity: 0.301, Template Neff: 12.1

```
Q ss_pred                    CCccceEEEEeeEecCcEEEEEEEEEeeCC---cceeeEEee-cCCCCcEEEEEEcccCeEeeeeeccCccceeeEeeeccc
Q Q_8363653_2      706       GVTSGQLVLNYTYKTTNISCEVQFKVKSG---RRAGIRFAS-TGPGDGYVFLIDYQTQEAMMFYETAGSSQLVASASLGD   781 (999)
Q Consensus        706       gvtsgqlvlnytykttniscevqfkvksg---rragirfas-tgpgdgyvflidyqtqeammfyetagssqlvasaslgd   781 (999)
                             ....+...+.......+...++.++...+   ..+|+.|.. .....+|.+.++............+....+++...
T Consensus        49        ~~~~~~~~~~~~~~~~~~~~~~~~~~~~~~~~~~~gi~~~~~~~~~~~~~~~~~~~~~~~~~~~~~~~~g~~~~~~~~~~~~--   126 (185)
T 4B1M_B           49        GRSDGDSFILSSASGSDFTYESDITIKDGNGRGAGALMFRSDKDAKNGYLANVDAKHDLVKFFKFENGAASVIAEYKT--   126 (185)
T ss_pred                    cCCCCceEEEecCCCCcEEEEEEEEEeCCCCCceEEEEEEeCCCCCCeEEEEEECCCCEEEEEEEeCCeeEEeeeeec--
```

```
Q ss_pred                    cccChhheEEEEEeeeCCeeeEEeccEEEEEecCCCCCCCCceeEEEeccceEEEEecc
Q Q_8363653_2      782       RRADYDELITLKVLVNNGKCRCYFGNVMFFMDMNLPHMSPGGIGFVATNCDAYLYKLSI   840 (999)
Q Consensus        782       rradydelitlkvlvnngkcrcyfgnvmffmdmnlphmspggigfvatncdaylyklsi   840 (999)
                              .....+..+|++.+.++..++|....  ...+...+...+|.+||.+.+....+-.+.+
T Consensus        127       -~~~~~~~~~~l~i~~~g~~i~~~vng~-~~~~~~~~~~~~~~g~~gl~~~~~~~~~~~~~i~v   183 (185)
T 4B1M_B           127       -PIDVNKKYHLKTEAEGDRFKIYLDDR-LVIDAHDSVFSEGQFGLNVWDATAVFQNVTK   183 (185)
T ss_pred                    -CCCCCCeEEEEEEEEECCEEEEEEECCE-EEEEEEcCCCCCCeeEEEEeCCeEEEEeEEE
```

Template alignment | Template 3D Structure | PDBe

48. 4DWS_A **Chi2; TIM barrel, Chitinase, Reductive methylation, SUGAR BINDING PROTEIN; HET: M3L, GOL, MLZ, MLY; 1.8A {Yersinia entomophaga}**

Probability: 96.22%, E-value: 0.018, Score: 58.52, Aligned cols: 177, Identities: 19%, Similarity: 0.206, Template Neff: 9.7

```
Q ss_pred                    HHHHhccchhhhHHHHhHHHHHHHHhhccCCCCcccceeeeEEecccccccccccc--CCCCcchhHcHHHHhcceeee--e
Q Q_8363653_2      116       VSRLLDSQTAQDNFINNLKTVVARFRKDQNGNDLGYTGIEIDCEGSFSDSKWDT--RAGDDVKYINLLKRIKNEVII--D   191 (999)
Q Consensus        116       vsrlldsqtaqdnfinnlktvvarfrkdqngndlgytgieidcegsfsdskwdt--ragddvkyinllkriknevii--d   191 (999)
                             .+.++.+.....+|++++...+.++         -++.||+||.|..-....-..   ...+...+..|++.++.+.--  .
T Consensus        227       ~~~~~~~~~~r~~fi~si~~~l~~~--------~~fDGIdidwe~~~~~~~~~~~~~~~~~~~~~~~~li~~Lr~~l~~~~~~   298 (546)
T 4DWS_A           227       FSALAENPDERRVFVASVVDFFVRF--------PMFSCVDIDWEYPGGGGDEGNISSDKDGENYVLLIKELRSALDSRFG   298 (546)
T ss_dssp                    HHHHHHCHHHHHHHHHHHHHHHHHHC--------TTEEEEEEECSCTTSCSCTTSCCCTTHHHHHHHHHHHHHHHHHHHHHT
T ss_pred                    HHHHhCCHHHHHHHHHHHHHHHHHHC--------CCCCeEEEEecCCCCCCCCCCCCCCChhHHHHHHHHHHHHHHHHHHhcC
```

```
Q ss_pred                    CCCCcceEEeehhccCCCCCchhheecceeeeeeCCCCCccccceeEEEEeecc--ccCCCCCCCCChH-----------
Q Q_8363653_2      192       ANPDFKLRINAHAMWGDGIPDYYRFHNYKLFAESTDKNGNPLLDEVQIMTYDFS--WSGSAPGPSTPLW-----------   258 (999)
Q Consensus        192       anpdfklrinahamwgdgipdyyrfhnyklfaestdkngnplldevqimtydfs--wsgsapgpstplw-----------   258 (999)
                             ..+++.|-+...+.     +.+..-.+++-.++..         +|.+.+|+|||.  +....+++.+||+
T Consensus        299       ~~~~~~lsia~~~~-----~~~~~~~~~~~~~~~l~~~~-------vD~i~v~~yd~~~~~~~~~~~~~~~~~~s~l~~~~~~~~~~~~~~   366 (546)
T 4DWS_A           299       YSNRKEISIACSGV-----KAKLKKSNIDQLVANG-------LDNIYLMSYDFFGTIWADYIGHHTNLYSPKDPGEQELF   366 (546)
T ss_dssp                    TTSCCEEEEEEEESS-----HHHHGGGCHHHHHHTT-------CCEEEEECCCSSCTTTCSSCCCSSCSSCCSSCTTCSSS
T ss_pred                    CCCCeEEEEEEecc-----hHHhccCCHHHHHHcC-------CCEEEEEcccccCccccchhccccCCCCCCCCCCCcchhh
```

```
Q ss_pred                    ---HHhcHHHHHHHH-hcCCCCCCCCcceEeeEEeCCCccccCCCCCCCCCCCCceeehhHhccccccEE
Q Q_8363653_2      259       ---WMRNVAEWVKQ-CFDPSVNPNAKCTIDNVYLGGAGYGRRWPIHSDDNWGSTVTYRDLVDWQNGYL   322 (999)
Q Consensus        259       ---wmrnvaewvkq-cfdpsvnpnakctidnvylggagygrrwpihsddnwgstvtyrdlvdwqngyl   322 (999)
                                +...+..|++. -++++            .+.+|-+.|||.|......+......-.-......|.+
T Consensus        367       ~~s~~~~~v~~~~~~~~~gvp~~----------KlvlGip~yg~~~~~~~~~~~~~~~~~~~~~~~~~~~~~~~g~~   424 (546)
T 4DWS_A           367       DLSAEAAIDYLHNELGIPME----------KIHLGYANYGRSAVGGDLTTRQYTKNGPALGTMENGAP   424 (546)
T ss_dssp                    SCCHHHHHHCCCCCSCCCGG----------GEEEEEESBCEEESSCCTTTCCCCTTSCCCBSSSTTCC
T ss_pred                    cCCHHHHHHHHHHHHHhCCCHH----------HEEEccccccceecCCCCCCCcccccCCCCCCCCCCCCCc
```

Template alignment | Template 3D Structure | PDBe

49. 4TXG_A **Chitinase; Chitinase Family GH18 Chitinase, HYDROLASE; HET: CS; 1.75A {Chromobacterium violaceum}**

Probability: 96.2%, E-value: 0.022, Score: 62.28, Aligned cols: 241, Identities: 16%, Similarity: 0.262, Template Neff: 8.6

```
Q ss_pred                    ceeeEEEeeecccCCCccEEEEEecCCCCCCCccccccCcccccCCCCCcccCcchHhhhhhcCccccceeeEeeee----
Q Q_8363653_2      36        NKITSIGFHEFNVDAQGRIAIYRLGPQKDAQGSFTSFTDATTYDRYAPGRTLWPNYIEKDMKRWPHIEYYMQFVIF----   111 (999)
Q Consensus        36        nkitsigfhefnvdaqgriaiyrlgpqkdaqgsftsftdattydryapgrtlwpnyiekdmkrwphieyymqfvif----   111 (999)
                             .++|.|-+--..++..|++.+-.-.+...+.+.--.-......+.-.+.+..+.. +.+--++.|++.-.+.+--.
T Consensus        181       ~~~ThI~yaFa~~~~~g~i~~~~~~~~~~~~~~~~~~~~~~~~~~~~~~~~~~~~~~~~-l~~lk~~~pg~KvllSiGG~~~s~   259 (793)
T 4TXG_A           181       SKLTHINYAFAHVDGSNKLSVNETAPGNPATDMSWPGVAGAEMDASLPYKGHFNL-LTQYKRKYPGVKTLISVGGWAETG   259 (793)
T ss_dssp                    GGCSEEEEEEEEEECTTSCEECCTTSTTCTTTSCCCTTCTTCCCCTTSSCCHHHHH-HHHHHHHSTTCEEEEEEEHHHHSS
T ss_pred                    HHCcEEEEEeeeeCCCCcEEeCCCCCCCCCcCCCCCCCCCccccCCCCCCcccchHHH-HHHHHHHCCCCEEEEEECCchhcc
```

```
Q ss_pred                    ----------CHHHHHHHhc------cchhhhHHHHhHHHHHHHHhhccCCCCcccceeeeEEecccccccccccc------
Q Q_8363653_2      112       ----------GPETVSRLLD------SQTAQDNFINNLKTVVARFRKDQNGNDLGYTGIEIDCEGSFSDSKWDT------   169 (999)
Q Consensus        112       ----------gpetvsrlld------sqtaqdnfinnlktvvarfrkdqngndlgytgieidcegsfsdskwdt------   169 (999)
                                       ..+....++.      +....++|++++..++.++.         +.||+||.|..-+...-..
T Consensus        260       ~~~~~~~~~~~s~~f~~~~~~~~~~~~~~~~~~~r~~f~~s~v~~l~~~g---------fDGvDID~E~p~~~~~~~~~~~~~d~~~   330 (793)
T 4TXG_A           260       GYFDANGKRVASGGFYSMTVNADGTVNQAGINAFSDSAVAFLRKYG---------FDGVDIDFEYPTSMNNAGNPLDWTF   330 (793)
T ss_dssp                    CEECTTSCEECCCHHHHHHBCTTSSBCHHHHHHHHHHHHHHHHHHHT---------CSEEEEECSCCCSTTCSCGGGHHH
T ss_pred                    CccccCCCeeecCCcceeeecCCCCCCHHHHHHHHHHHHHHHHHHhC---------CCcEEEEeeCCCccCCCCCCCCchhh
```

```
Q ss_pred                    ----CCCCcchhHcHHHHhcceeeeeC---CCCcceEEe-ehhccCCCCCchhheecceeeeeeCCCCCccccceeEEEE
Q Q_8363653_2      170       ----RAGDDVKYINLLKRIKNEVIIDA---NPDFKLRIN-AHAMWGDGIPDYYRFHNYKLFAESTDKNGNPLLDEVQIMT   241 (999)
Q Consensus        170       ----ragddvkyinllkrikneviida---npdfklrin-ahamwgdgipdyyrfhnyklfaestdkngnplldevqimt   241 (999)
                                 +..+...|..|+|.++++.-...   .+.+.|-+- ...-|...--+......|               +|-|.||+
T Consensus        331       ~~~~~~~~~~~~~~~~~~ll~~Lr~~l~~~~~~~~~g~~~~Lt~a~~~~~~~~~~~~~~~~~~~~~~l~~~--------------lD~vnvmt   396 (793)
T 4TXG_A           331       SNARLGSLNKGYVALLQTLRDRLDRAAAQDGRYYQITAAVPASGYLLRGMETFQGLKY--------------LDFVNVMS   396 (793)
T ss_dssp                    HTTSTTTHHHHHHHHHHHHHHHHHHHHHHHHTSCCEEEEEEECCHHHHTTSTTCGGGGG--------------CSEEEEEC
T ss_pred                    cccchhhHHHHHHHHHHHHHHHHHHHHHHhhCCeEEEEEEEecCCchhhcCCcHHHHHHH--------------CCEEEEec
```

```
Q ss_pred                 eecccc-CCCCCCCCChHHHh---------------------cHHHHHHHhcCCCCCCCCcceEeeEEeCCCccccCCCC
Q Q_8363653_2      242    YDFSWS-GSAPGPSTPLWWMR---------------------NVAEWVKQCFDPSVNPNAKCTIDNVYLGGAGYGRRWPI   299 (999)
Q Consensus        242    ydfsws-gsapgpstplwwmr---------------------nvaewvkqcfdpsvnpnakctidnvylggagygrrwpi   299 (999)
                          |||..+ ....++..||+...                    .-.+|+.+.+.-.+.+      ..+.||-.-|||.|..
T Consensus        397    YD~~g~w~~~~g~~spL~~~~~~~~~~~~~~~~~~~~~~~~~~~~~~~~~~~~~~~gvp~------~KivlGip~Ygr~~~~   470 (793)
T 4TXG_A           397    YDLHGAWNRFVGPNAALYDDGKDAELAFWNVYSTPQYGNIGYLNTDWAYHYYRGGLPA------SRVNMGVPYYTRGWKN   470 (793)
T ss_dssp                  CCSSCTTSSBCCCSSCSSBCSCCHHHHHTTTTTCGGGTTCCCSSHHHHHHHHTTTSCG------GGEEEEEESBCEEESS
T ss_pred                  CCCCCCcccCCCCCCcccCCCCChhHhhhccCCCCccCCccccHHHHHHHHhcCCCH------HHEEEccccceeeeec


Q ss_pred                 CCCCCCC
Q Q_8363653_2      300    HSDDNWG   306 (999)
Q Consensus        300    hsddnwg   306 (999)
                          -+....|
T Consensus        471    ~~~~~~g   477 (793)
T 4TXG_A           471    VSGGSNG   477 (793)
T ss_dssp                  CBSSBTT
T ss_pred                  CCCCCCC
```

Template alignment | Template 3D Structure | PDBe

50. **1WB0_A CHITOTRIOSIDASE 1; CYCLOPENTAPEPTIDE INHIBITORS, CHITINASE INHIBITORS, CARBOHYDRATE METABOLISM, CHITIN DEGRADATION, CHITIN-BINDING, GLYCOSIDASE, HYDROLASE-HYDROLASE INHIBITOR COMPLEX; HET: VR0, SO4, MEA, IAS; 1.65A {HOMO SAPIENS} SCOP: c.1.8.5, d.26.3.1**

Probability: 96.15%, E-value: 0.0087, Score: 55.75, Aligned cols: 176, Identities: 22%, Similarity: 0.332, Template Neff: 11.4

```
Q ss_pred                 HhhhhhcCcccceeeEee--eeCHHHHHHHhccchhhhHHHHhHHHHHHHHhhccCCCCccceeeeEEeccccccccccc
Q Q_8363653_2       92    IEKDMKRWPHIEYYMQFV--IFGPETVSRLLDSQTAQDNFINNLKTVVARFRKDQNGNDLGYTGIEIDCEGSFSDSKWDT   169 (999)
Q Consensus         92    iekdmkrwphieyymqfv--ifgpetvsrlldsqtaqdnfinnlktvvarfrkdqngndlgytgieidcegsfsdskwdt   169 (999)
                          +..-.++.|++.-++.+-  -++.+..++++.+....+.|++++..++.++          ++.||+||.|..-++   ..
T Consensus         59    ~~~l~~~~~~~~~kv~lsigg~~~~~~~~~~~~~~~~~~~~~~~~~~~i~~~~~~~---------~~DGvdld~e~~~~~---~~   126 (445)
T 1WB0_A            59    FNGLKKMNPKLKTLLAIGGWNFGTQKFTDMVATANNRQTFVNSAIRFLRKY---------SFDGLDLDWEYPGSQ---GS   126 (445)
T ss_dssp                  HHHGGGTCTTCEEEEEEECTTTCSHHHHHHHTSHHHHHHHHHHHHHHHHHHT---------TCCEEEEECSCTTST---TC
T ss_pred                  HHHHHHHCCCCcEEEEECCcccCChhHHHHhcCHHHHHHHHHHHHHHHHHHh---------CCCCeEeCccCCCCC---CC


Q ss_pred                 CCCCcchhHcHHHHhcceeeeeCCCCcceEEeehhccCCCCCchhheecceeeeeecCCCCCccccceeEEEEeecccc--
Q Q_8363653_2      170    RAGDDVKYINLLKRIKNEVIIDANPDFKLRINAHAMWGDGIPDYYRFHNYKLFAESTDKNGNPLLDEVQIMTYDFSWS--   247 (999)
Q Consensus        170    ragddvkyinllkrikneviidanpdfklrinahamwgdgipdyyrfhnyklfaestdkngnplldevqimtydfsws--   247 (999)
                          ...+.-.|..++++++.+.--......+-....-+.-.-.....-...+.+-.++.          +|-+.+|+||+...
T Consensus        127    ~~~~~~~~~~~~~~~l~~~~~~~~~~~~~~~~~~~ls~~~~~~~~~~~~~~~~~~~~~~~--------~D~~~v~~y~~~~~~~~   198 (445)
T 1WB0_A           127    PAVDKERFTTLVQDLANAFQQEAQTSGKERLLLSAAVPAGQTYVDAGYEVDKIAQN--------LDFVNLMAYDFHGSWE   198 (445)
T ss_dssp                  CTTHHHHHHHHHHHHHHHHHHHHHHHHHCSCCCEEEEEECCCHHHHHHHCCHHHHHHH--------CSEEEECCCCSSCTTS
T ss_pred                  ChhhHHHHHHHHHHHHHHHHHHHHHHHhCCccEEEEEEecCCHHHHhcCCCHHHHHHh--------cCEEEeecccccCCcc


Q ss_pred                 ------------CCCCCCCCChHHHhcHHHHHHHhcCCCCCCCCcceEeeEEeCCCccccCC
Q Q_8363653_2      248    ------------GSAPGPSTPLWWMRNVAEWVKQCFDPSVNPNAKCTIDNVYLGGAGYGRRW   297 (999)
Q Consensus        248    ------------gsapgpstplwwmrnvaewvkqcfdpsvnpnakctidnvylggagygrrw   297 (999)
                                        ...+++..+.-+...+..|+++-..++           .+.+|-..||+.|
T Consensus        199    ~~~~~~~~~~~~~~~~~~~~~~~~~~~~~~~~~~~g~~~~----------Kl~~g~~~~g~~~~   250 (445)
T 1WB0_A           199    KVTGHNSPLYKRQEESGAAASLNVDAAVQQWLQKGTPAS----------KLILGMPTYGRSF   250 (445)
T ss_dssp                  SBCCCSSCSSCCTTCCGGGGGCSHHHHHHHHHHTTCCGG----------GEEEEEESEEEEE
T ss_pred                  cccCCCCCcCCcccccccccCCHHHHHHHHHHcCCCHH----------HEEEEeecceEEE
```

If you use HHpred on our Toolkit for your research, please cite as appropriate:

A Completely Reimplemented MPI Bioinformatics Toolkit with a New HHpred Server at its Core.
Zimmermann L, Stephens A, Nam SZ, Rau D, Kübler J, Lozajic M, Gabler F, Söding J, Lupas AN, Alva V. J Mol Biol. 2018 Jul 20. S0022-2836(17)30587-9.

Protein Sequence Analysis Using the MPI Bioinformatics Toolkit.
Gabler F, Nam SZ, Till S, Mirdita M, Steinegger M, Söding J, Lupas AN, Alva V. Curr Protoc Bioinformatics. 2020 Dec;72(1):e108. doi: 10.1002/cpbi.108.

Protein homology detection by HMM-HMM comparison.
Söding J. Bioinformatics. 2005 Apr 1;21(7):951-60.

Fast and accurate automatic structure prediction with HHpred.
Hildebrand A, Remmert M, Biegert A, Söding J. Proteins. 2009;77 Suppl 9:128-32.

Automatic Prediction of Protein 3D Structures by Probabilistic Multi-template Homology Modeling.
Meier A, Söding J. PLoS Comput Biol. 2015 Oct 23;11(10):e1004343.

UniProt: the universal protein knowledgebase in 2021.
UniProt Consortium. Nucleic Acids Res. 2021;49(D1):D480-D489.

RCSB Protein Data Bank: powerful new tools for exploring 3D structures of biological macromolecules for basic and applied research and education in fundamental biology, biomedicine, biotechnology, bioengineering and energy sciences.
Burley SK, Bhikadiya C, Bi C, et al. Nucleic Acids Res. 2021;49(D1):D437-D451.

ECOD: an evolutionary classification of protein domains.
Cheng H, Schaeffer RD, Liao Y, et al. PLoS Comput Biol. 2014;10(12):e1003926.

SCOPe: improvements to the structural classification of proteins - extended database to facilitate variant interpretation and machine learning.

Chandonia JM, Guan L, Lin S, Yu C, Fox NK, Brenner SE. Nucleic Acids Res. 2022;50(D1):D553-D559.

PHROG: families of prokaryotic virus proteins clustered using remote homology.
Terzian P, Olo Ndela E, Galiez C, et al. NAR Genom Bioinform. 2021;3(3):lqab067.

CATH: increased structural coverage of functional space.
Sillitoe I, Bordin N, Dawson N, et al. Nucleic Acids Res. 2021;49(D1):D266-D273.

© 2008-2022, Dept. of Protein Evolution, Max Planck Institute for Biology, Tübingen
