# Supplementary material for: A novel thermostable TP-84 capsule depolymerase: a method for rapid polyethyleneimine processing of a bacteriophage-expressed proteins
Source: Microb Cell Fact. 2023 Apr 25;22:80. doi: 10.1186/s12934-023-02086-2 (PMC10131341; doi:10.1186/s12934-023-02086-2)
Supplement: Supplementary file 9 — Additional file 9: Combined LC-MS analysis of depolymerase. [file 12934_2023_2086_MOESM9_ESM.pdf]

Table 1. TP-84 genes/proteins numbering legend

previous TP-84 genes/proteins numbering (as shown on MS results below)*	current genes/proteins numbering (this work, Table 1)	molecular weight (kDa)
25	53	38,5
26	54	38,8
29	57	30,4
32	60	26,4
38	66	10,4
41	69	20,1
44	72	19,3
46	74	21,3
56	01	22,4
57	02	70,0
58	03	26,3
61	06	62,3
63	08	78,0
65	10	31,6
66	11	14,0
67	12	37,8
68	13	7,2
71	16	18,7
73	18	20,4
74	19	19,3
75	20	14,7
76	21	93,1
77	22	27,9
78	23	99,4
80	25	34,4
81	26	112,2
83	28	44,2

* previous genes/proteins numbering was present in the database, constructed prior TP-84 analysis manuscript submission (*PLOS ONE* 2018, **13**: e0195449). Upon submission revised version of the Manuscript the numbering has been changed to follow convention of assigning a terminase gene as no 1.

MASCOT Search Results

User :
E-mail :
Search title : 200420 592 Czajkowska
MS data file : O:\jasio\MGF\Czajkowska 20200525\00418592czaj_1.mgf
Database : TP84 20170731 (83 sequences; 14,773 residues)
Timestamp : 26 May 2020 at 10:22:37 GMT

Re-search ◉ All ○ Non-significant ○ Unassigned [help] Export As XML

Not what you expected? Try the select summary.

Search parameters
Score distribution
Modification statistics for all protein families
Legend

Protein Family Summary

Format	Significance threshold p<	0.000152	Max. number of families	AUTO
	Target FDR (overrides sig. threshold)	(not set)	FDR type	PSM
	Display non-sig. matches	☐	Min. number of sig. unique sequences	1
			Dendrograms cut at	0
	Preferred taxonomy	All entries		

[help]

Sensitivity and FDR (reversed protein sequences)

Proteins (21) | Report Builder | Unassigned (10190) | § permalink

Protein families 11–20 (out of 21)

10 per page Previous 1 2 3 Next Expand all Collapse all

Accession contains Find Clear

**11**	**65.**	187	65.
**12**	**63.**	178	63.
**13**	**25.**	163	25.
14	**81.**	108	81.

		Score	Mass	Matches	Sequences	emPAI	
**14.1**	81.	108	112893	2 (2)	2 (2)	0.08	81.

2 peptide matches (2 non-duplicate, 0 duplicate)

☑ Auto-fit to window

Query	Dupes	Observed	Mr(expt)	Mr(calc)	ppm	M	Score	Expect	Rank	U	Peptide
3624		587.2637	1172.5128	1172.5132	-0.35	0	70	9.4e-08	1	U	R.YDSMGQTTVR.V + Oxidation (M)
7727		941.9061	1881.7976	1881.8017	-2.17	0	76	2.5e-08	1	U	K.DAQGSFTSFTDATTYDR.Y

**15**	**38.**	105	38.
**16**	**75.**	99	75.
**17**	**58.**	96	58.
**18**	**82.**	50	82.
**19**	**56.**	47	56.
**20**	**80.**	43	80.

10 per page Previous 1 2 3 Next Expand all Collapse all

Not what you expected? Try the select summary.

MASCOT Search Results

User :
E-mail :
Search title : 200420 592 Czajkowska
MS data file : O:\jasio\MGF\Czajkowska 20200426\00418592czaj_1.mgf
Database : TP84 20170731 (83 sequences; 14,773 residues)
Timestamp : 29 Apr 2020 at 16:19:20 GMT

Re-search ◉ All ○ Non-significant ○ Unassigned [help] Export As XML

Not what you expected? Try the select summary.

Search parameters

Score distribution

Modification statistics for all protein families

Legend

Protein Family Summary

Format	Significance threshold p<	0.000152	Max. number of families	AUTO	[help]
	Target FDR (overrides sig. threshold)	(not set)	FDR type	PSM	
	Display non-sig. matches	☐	Min. number of sig. unique sequences	2	
			Dendrograms cut at	0	
	Preferred taxonomy	All entries			

Sensitivity and FDR (reversed protein sequences)

Proteins (16) | Report Builder | Unassigned (10189) | § permalink

Protein families 11–16 (out of 16)

10 per page Previous 1 2 Expand all Collapse all

Accession contains Find Clear

**11**	**32.**	201	32.
**12**	**63.**	182	63.
**13**	**25.**	165	25.
14	**81.**	122	81.

		Score	Mass	Matches	Sequences	emPAI	
**14.1**	81.	122	112893	2 (2)	2 (2)	0.08	81.

2 peptide matches (2 non-duplicate, 0 duplicate)

☑ Auto-fit to window

Query	Dupes	Observed	Mr(expt)	Mr(calc)	ppm	M	Score	Expect	Rank	U	Peptide
3624		587.2655	1172.5164	1172.5132	2.72	0	70	9.4e-08	1	U	R.YDSMGQTTVR.V + Oxidation (M)
7727		941.9115	1881.8084	1881.8017	3.52	0	90	9.5e-10	1	U	K.DAQGSFTSFTDATTYDR.Y

**15**	**38.**	105	38.
**16**	**58.**	101	58.

10 per page Previous 1 2 Expand all Collapse all

Not what you expected? Try the select summary.

MASCOT Search Results

User :
E-mail :
Search title : 200420 592 Czajkowska
MS data file : O:\jasio\MGF\Czajkowska 20200426\00418593czaj_2.mgf
Database : TP84 20170731 (83 sequences; 14,773 residues)
Timestamp : 29 Apr 2020 at 16:22:22 GMT

Re-search ◉ All ○ Non-significant ○ Unassigned [help] Export As XML

Not what you expected? Try the select summary.

▸ **Search parameters**
▸ **Score distribution**
▸ **Modification statistics for all protein families**
▸ **Legend**

Protein Family Summary

Format	Significance threshold p<	0.00338	Max. number of families	AUTO [help]
	Target FDR (overrides sig. threshold)	(not set)	FDR type	PSM
	Display non-sig. matches	☐	Min. number of sig. unique sequences	2
			Dendrograms cut at	0
	Preferred taxonomy	All entries		

▸ **Sensitivity and FDR (reversed protein sequences)**

Proteins (8) | Report Builder | Unassigned (13175) | § permalink

Protein families 1–8 (out of 8)

10 per page 1 Expand all Collapse all

Accession contains Find Clear

▸**1** **78.** 3739 78.
▸**2** **61.** 3564 61.
▾***3*** **81.** 2175 81.

		Score	Mass	Matches	Sequences	emPAI	
**3.1**	81.	2175	112893	45 (45)	27 (27)	1.85	81.

▾***45 peptide matches (32 non-duplicate, 13 duplicate)***

☑ Auto-fit to window

Query	Dupes	Observed	Mr(expt)	Mr(calc)	ppm	M	Score	Expect	Rank	U	Peptide
337		382.2397	762.4649	762.4639	1.31	0	44	4.4e-05	1	U	K.YINLLK.R
1110	1	438.7434	875.4723	875.4712	1.18	0	58	1.6e-06	1	U	K.LSIGTTER.W
1317	1	455.7304	909.4462	909.4444	2.06	0	64	4.1e-07	1	U	K.LFAESTDK.N
1411	1	462.2147	922.4149	922.4144	0.53	0	38	0.00017	1	U	R.IQNDDYR.H
1979		494.2982	986.5818	986.5801	1.73	0	51	7.9e-06	1	U	R.VPYVPTAIK.D
2093	1	501.3040	1000.5934	1000.5917	1.69	0	84	4.1e-09	1	U	K.ISGLAGVTGVK.G
2832	1	539.8013	1077.5881	1077.5859	2.10	0	79	1.4e-08	1	U	K.LIDAGLWYK.Q
3511		381.9014	1142.6824	1142.6812	1.10	1	30	0.00097	1	U	K.RVPYVPTAIK.D
3628		579.2672	1156.5198	1156.5183	1.28	0	81	8.4e-09	1	U	R.YDSMGQTTVR.V
3669		582.3098	1162.6050	1162.6022	2.34	0	50	1.1e-05	1	U	R.TLWPNYIEK.D
3786		587.2649	1172.5153	1172.5132	1.75	0	71	7.5e-08	1	U	R.YDSMGQTTVR.V + Oxidation (M)
4593		415.8847	1244.6323	1244.6295	2.25	0	26	0.0025	1	U	K.MGEISRPGINR.D + Oxidation (M)
5019	2	649.8362	1297.6579	1297.6554	1.90	0	97	1.8e-10	1	U	R.FSVIIDGQEYK.M
5276		663.8239	1325.6332	1325.6286	3.49	0	73	5.2e-08	1	U	K.TTNISCEVQFK.V
5379	1	668.8607	1335.7068	1335.7034	2.57	1	69	1.3e-07	1	U	R.RADYDELITLK.V
5747		687.8485	1373.6825	1373.6827	-0.10	0	64	4.3e-07	1	U	K.NEVIIDANPDFK.L
5758		688.8172	1375.6198	1375.6190	0.52	0	88	1.7e-09	1	U	K.QCFDPSVNPNAK.C
5805	1	691.3845	1380.7544	1380.7514	2.19	0	68	1.8e-07	1	U	K.GIGLWVLGQADPR.I
6072		704.8213	1407.6281	1407.6275	0.45	0	62	6.3e-07	1	U	R.AMNIAVHEYGCK.G + Oxidation (M)
6073		470.2168	1407.6285	1407.6275	0.74	0	47	1.9e-05	1	U	R.AMNIAVHEYGCK.G + Oxidation (M)
7470		808.3808	1614.7470	1614.7460	0.60	0	86	2.3e-09	1	U	K.CTIDNVYLGGAGYGR.R
8106		847.9006	1693.7866	1693.7835	1.84	0	129	1.3e-13	1	U	R.VSEYNGIEYATAYSK.T
8506		585.6175	1753.8305	1753.8271	1.95	0	67	2.1e-07	1	U	K.GVSEPTHNLSPYDNPK.R
8507		877.9226	1753.8306	1753.8271	1.98	0	108	1.6e-11	1	U	K.GVSEPTHNLSPYDNPK.R
8715		895.9465	1789.8785	1789.8748	2.11	0	151	8.3e-16	1	U	K.ITSIGFHEFNVDAQGR.I
8717	1	597.6338	1789.8795	1789.8748	2.65	0	85	2.8e-09	1	U	K.ITSIGFHEFNVDAQGR.I
9068		917.4721	1832.9297	1832.9268	1.58	0	125	3.1e-13	1	U	R.LLDSQTAQDNFINNLK.T
9352	1	941.9091	1881.8037	1881.8017	1.06	0	120	9.4e-13	1	U	K.DAQGSFTSFTDATTYDR.Y
9513	1	645.3004	1932.8795	1932.8755	2.05	0	43	5.3e-05	1	U	R.WPIHSDDNWGSTVTYR.D

▸**4** **67.** 1571 67.
▸**5** **76.** 1206 76.
▸**6** **63.** 1121 63.
▸**7** **65.** 386 65.
▸**8** **74.** 70 74.

10 per page 1 Expand all Collapse all

Not what you expected? Try the select summary.

MASCOT Search Results

User :
E-mail :
Search title : 200420 592 Czajkowska
MS data file : O:\jasio\MGF\Czajkowska 20200525\00418593czaj_2.mgf
Database : TP84 20170731 (83 sequences; 14,773 residues)
Timestamp : 26 May 2020 at 10:23:06 GMT

Re-search ◉ All ○ Non-significant ○ Unassigned [help] Export As XML

Not what you expected? Try the select summary.

Search parameters
Score distribution
Modification statistics for all protein families
Legend

Protein Family Summary

Format	Significance threshold p<	0.00338	Max. number of families	AUTO	[help]
	Target FDR (overrides sig. threshold)	(not set)	FDR type	PSM	
	Display non-sig. matches	☐	Min. number of sig. unique sequences	1	
			Dendrograms cut at	0	
	Preferred taxonomy	All entries			

Sensitivity and FDR (reversed protein sequences)

Proteins (9) | Report Builder | Unassigned (13186) | § permalink

Protein families 1–9 (out of 9)

10 per page 1 Expand all Collapse all

Accession contains Find Clear

1	78.	3648	78.
2	61.	3366	61.
3	81.	2091	81.

		Score	Mass	Matches	Sequences	emPAI	
3.1	81.	2091	112893	44 (44)	26 (26)	1.75	81.

44 peptide matches (31 non-duplicate, 13 duplicate)

☑ Auto-fit to window

Query	Dupes	Observed	Mr(expt)	Mr(calc)	ppm	M	Score	Expect	Rank	U	Peptide
337		382.2390	762.4635	762.4639	-0.54	0	44	4.4e-05	1	U	K.YINLLK.R
1110	1	438.7426	875.4706	875.4712	-0.71	0	58	1.6e-06	1	U	K.LSIGTTER.W
1317	1	455.7295	909.4445	909.4444	0.17	0	64	4.1e-07	1	U	K.LFAESTDK.N
1411	1	462.2139	922.4132	922.4144	-1.37	0	38	0.00017	1	U	R.IQNDDYR.H
1979		494.2972	986.5799	986.5801	-0.19	0	51	7.9e-06	1	U	R.VPYVPTAIK.D
2093	1	501.3030	1000.5915	1000.5917	-0.24	0	81	7.9e-09	1	U	K.ISGLAGVTGVK.G
2832	1	539.8003	1077.5860	1077.5859	0.15	0	79	1.4e-08	1	U	K.LIDAGLWYK.Q
3511		381.9007	1142.6803	1142.6812	-0.76	1	29	0.0013	1	U	K.RVPYVPTAIK.D
3628		579.2660	1156.5175	1156.5183	-0.69	0	79	1.2e-08	1	U	R.YDSMGQTTVR.V
3669		582.3086	1162.6027	1162.6022	0.37	0	50	1.1e-05	1	U	R.TLWPNYIEK.D
3786		587.2637	1172.5129	1172.5132	-0.22	0	71	7.5e-08	1	U	R.YDSMGQTTVR.V + Oxidation (M)
5019	2	649.8349	1297.6553	1297.6554	-0.098	0	97	1.8e-10	1	U	R.FSVIIDGQEYK.M
5276		663.8225	1325.6305	1325.6286	1.48	0	71	8e-08	1	U	K.TTNISCEVQFK.V
5379	1	668.8593	1335.7041	1335.7034	0.55	1	65	3.3e-07	1	U	R.RADYDELITLK.V
5747		687.8472	1373.6798	1373.6827	-2.13	0	64	4.3e-07	1	U	K.NEVIIDANPDFK.L
5758		688.8158	1375.6170	1375.6190	-1.51	0	86	2.6e-09	1	U	K.QCFDPSVNPNAK.C
5805	1	691.3831	1380.7516	1380.7514	0.16	0	68	1.8e-07	1	U	K.GIGLWVLGQADPR.I
6072		704.8199	1407.6253	1407.6275	-1.58	0	58	1.5e-06	1	U	R.AMNIAVHEYGCK.G + Oxidation (M)
6073		470.2159	1407.6258	1407.6275	-1.17	0	43	4.7e-05	1	U	R.AMNIAVHEYGCK.G + Oxidation (M)
7470		808.3791	1614.7436	1614.7460	-1.49	0	85	3.2e-09	1	U	K.CTIDNVYLGGAGYGR.R
8106		847.8988	1693.7830	1693.7835	-0.27	0	129	1.3e-13	1	U	R.VSEYNGIEYATAYSK.T
8506		877.9207	1753.8269	1753.8271	-0.14	0	108	1.6e-11	1	U	K.GVSEPTHNLSPYDNPK.R
8507		585.6163	1753.8271	1753.8271	-0.013	0	66	2.7e-07	1	U	K.GVSEPTHNLSPYDNPK.R
8715		895.9446	1789.8747	1789.8748	-0.026	0	131	8.9e-14	1	U	K.ITSIGFHEFNVDAQGR.I
8717	1	597.6326	1789.8760	1789.8748	0.67	0	85	2.8e-09	1	U	K.ITSIGFHEFNVDAQGR.I
9068		917.4702	1832.9258	1832.9268	-0.57	0	122	5.7e-13	1	U	R.LLDSQTAQDNFINNLK.T
9352	1	941.9071	1881.7997	1881.8017	-1.10	0	120	9.4e-13	1	U	K.DAQGSFTSFTDATTYDR.Y
9513	1	645.2991	1932.8756	1932.8755	0.051	0	41	8.5e-05	1	U	R.WPIHSDDNWGSTVTYR.D
10272		707.0088	2118.0046	2118.0058	-0.56	0	31	0.00086	1	U	R.IFETLPDVVPWHPDPNEN.-

4	67.	1431	67.
5	76.	1114	76.
6	63.	1067	63.
7	65.	381	65.
8	32.	80	32.
9	74.	68	74.

10 per page 1 Expand all Collapse all

Not what you expected? Try the select summary.

MASCOT Search Results

User :
E-mail :
Search title : 200518 704 Czajkowska
MS data file : O:\jasio\MGF\Czajkowska 20200526\00518704czaj_500_2_BL_1.mgf
Database : TP84 20170731 (83 sequences; 14,773 residues)
Timestamp : 26 May 2020 at 07:35:10 GMT

Re-search ◉ All ○ Non-significant ○ Unassigned [help] Export As XML

Not what you expected? Try the select summary.

- **Search parameters**
- **Score distribution**
- **Modification statistics for all protein families**
- **Legend**

Protein Family Summary

Format	Significance threshold p<	0.05	Max. number of families	AUTO	[help]
	Target FDR (overrides sig. threshold)	(not set)	FDR type	PSM	
	Ions score or expect cut-off	0.05	Min. number of sig. unique sequences	1	
			Dendrograms cut at	0	
	Preferred taxonomy	All entries			

Sensitivity and FDR (reversed protein sequences)

Proteins (3) | Report Builder | Unassigned (12472) | § permalink

Protein families 1–3 (out of 3)

10 per page 1 Expand all Collapse all

Accession contains Find Clear

1 **81.** 789 81.

		Score	Mass	Matches	Sequences	emPAI	
1.1	81.	789	112893	14 (14)	13 (13)	0.63	81.

14 peptide matches (14 non-duplicate, 0 duplicate)

☑ Auto-fit to window

Query	Dupes	Observed	Mr(expt)	Mr(calc)	ppm	M	Score	Expect	Rank	U	Peptide
584		417.2115	832.4085	832.4079	0.71	0	16	0.027	1	U	R.WETLER.F
827		437.2323	872.4501	872.4504	-0.39	0	18	0.015	1	U	R.FRPNDPK.N
1684		501.3033	1000.5920	1000.5917	0.29	0	71	8.4e-08	1	U	K.ISGLAGVTGVK.G
2916		572.3481	1142.6816	1142.6812	0.36	1	35	0.0003	1	U	K.RVPYVPTAIK.D
3218		587.2634	1172.5122	1172.5132	-0.82	0	78	1.7e-08	1	U	R.YDSMGQTTVR.V + Oxidation (M)
4357		649.8354	1297.6563	1297.6554	0.67	0	46	2.7e-05	1	U	R.FSVIIDGQEYK.M
4631		663.8216	1325.6286	1325.6286	0.0098	0	42	5.8e-05	1	U	K.TTNISCEVQFK.V
6984		808.4383	1614.8620	1614.8617	0.22	1	104	3.6e-11	1	U	R.IKNEVIIDANPDFK.L
6995		810.4263	1618.8381	1618.8395	-0.89	0	56	2.8e-06	1	U	K.IGAIVSFPFYNYTK.L
7772		877.9201	1753.8257	1753.8271	-0.84	0	70	1.1e-07	1	U	K.GVSEPTHNLSPYDNPK.R
7773		585.6165	1753.8276	1753.8271	0.27	0	82	5.8e-09	1	U	K.GVSEPTHNLSPYDNPK.R
7999		895.9450	1789.8754	1789.8748	0.33	0	147	1.8e-15	1	U	K.ITSIGFHEFNVDAQGR.I
8197		917.4703	1832.9261	1832.9268	-0.40	0	125	3.3e-13	1	U	R.LLDSQTAQDNFINNLK.T
9525		1060.0094	2118.0043	2118.0058	-0.72	0	68	1.7e-07	1	U	R.IFETLPDVVPWHPDPNEN.-

2 **26.** 22 26.

3 **31.** 14 31.

10 per page 1 Expand all Collapse all

Not what you expected? Try the select summary.

MASCOT Search Results

User :
E-mail :
Search title : 201104 1950 Czajkowska bacteria
MS data file : O:\jasio\MGF\Czajkowska 20201104\010301950czaj_dial16_8_BL.mgf
Database : TP84 20170731 (83 sequences; 14,773 residues)
Timestamp : 4 Nov 2020 at 12:20:37 GMT

Re-search ◉ All ○ Non-significant ○ Unassigned [help] Export As XML

Not what you expected? Try the select summary.

- **Search parameters**
- **Score distribution**
- **Modification statistics for all protein families**
- **Legend**

Protein Family Summary

Format	Significance threshold p<	0.03296	Max. number of families	AUTO	[help]
	Target FDR (overrides sig. threshold)	(not set)	FDR type	PSM	
	Display non-sig. matches	☐	Min. number of sig. unique sequences	1	
			Dendrograms cut at	0	
	Preferred taxonomy	All entries			

Sensitivity and FDR (reversed protein sequences)

Proteins (7) | Report Builder | Unassigned (5480) | § permalink

Protein families 1–7 (out of 7)

10 per page 1 Expand all Collapse all

Accession contains Find Clear

1 **81.** 1154 81.

		Score	Mass	Matches	Sequences	emPAI	
**1.1**	81.	1154	112893	19 (19)	13 (13)	0.63	81.

19 peptide matches (15 non-duplicate, 4 duplicate)

☑ Auto-fit to window

Query	Dupes	Observed	Mr(expt)	Mr(calc)	ppm	M	Score	Expect	Rank	U	Peptide
163		417.2116	832.4087	832.4079	1.00	0	21	0.0076	1	U	R.WETLER.F
263		462.2141	922.4137	922.4144	-0.86	0	44	3.7e-05	1	U	R.IQNDDYR.H
600		539.8001	1077.5856	1077.5859	-0.27	0	83	5.1e-09	1	U	K.LIDAGLWYK.Q
804		572.3480	1142.6814	1142.6812	0.17	1	23	0.0046	1	U	K.RVPYVPTAIK.D
853		582.3064	1162.5983	1162.6022	-3.36	0	38	0.00016	1	U	R.TLWPNYIEK.D
914		590.8065	1179.5985	1179.6023	-3.25	0	66	2.8e-07	1	U	R.ADYDELITLK.V
1221		649.8339	1297.6533	1297.6554	-1.61	0	100	9.8e-11	1	U	R.FSVIIDGQEYK.M
2011		539.2949	1614.8629	1614.8617	0.75	1	83	5e-09	1	U	R.IKNEVIIDANPDFK.L
2012		808.4396	1614.8646	1614.8617	1.77	1	95	3.2e-10	1	U	R.IKNEVIIDANPDFK.L
2019	1	810.4268	1618.8390	1618.8395	-0.35	0	105	3.5e-11	1	U	K.IGAIVSFPFYNYTK.L
2428	1	877.9200	1753.8254	1753.8271	-0.98	0	78	1.7e-08	1	U	K.GVSEPTHNLSPYDNPK.R
2429	1	585.6163	1753.8270	1753.8271	-0.088	0	80	9.8e-09	1	U	K.GVSEPTHNLSPYDNPK.R
2530		597.6315	1789.8727	1789.8748	-1.13	0	92	5.9e-10	1	U	K.ITSIGFHEFNVDAQGR.I
2808		941.9069	1881.7992	1881.8017	-1.34	0	129	1.2e-13	1	U	K.DAQGSFTSFTDATTYDR.Y
3236	1	1060.0059	2117.9972	2118.0058	-4.06	0	72	6.1e-08	1	U	R.IFETLPDVVPWHPDPNEN.-

**2**	**76.**	1009	76.
**3**	**78.**	981	78.
**4**	**61.**	549	61.
**5**	**67.**	476	67.
**6**	**66.**	138	66.
**7**	**65.**	79	65.

10 per page 1 Expand all Collapse all

Not what you expected? Try the select summary.

Mascot: http://www.matrixscience.com/

MATRIX SCIENCE Mascot Search Results

```
User                   : Asia Zebrowska
Email                  : asia.zebrowska87@gmail.com
Search title           : Copy of Zebrowska cal viruses sheep
MS data file           : \\212.87.29.243\Dane\USERS\Agata\tmp\00229311zebr_BL_depol-Q1.mgf
Database 1             : Sprot 2020_01 (561911 sequences; 202173710 residues)
Taxonomy 1             : Viruses (16712 sequences)
Database 2             : Trembl 2019_11 (179250561 sequences; 60319985647 residues)
Taxonomy 2             : Viruses (4300387 sequences)
Timestamp              : 11 Mar 2020 at 13:08:53 GMT
Enzyme                 : Trypsin
Fixed modifications    : Carbamidomethyl (C)
Variable modifications : Oxidation (M)
Mass values            : Monoisotopic
Protein Mass           : Unrestricted
Peptide Mass Tolerance : ± 5.02 ppm
Fragment Mass Tolerance: ± 0.01 Da
Max Missed Cleavages   : 1
Instrument type        : HCD
Number of queries      : 4822
Protein hits           : 2::A0A1U9WQS0 >tr|A0A1U9WQS0|A0A1U9WQS0_9CAUD Glycosylase OS=Geobacillus phage TP-84 OX=1965361 PE=4 SV=1
                         2::W6LP63     >tr|W6LP63|W6LP63_9CAUD Enolase OS=Streptococcus phage 20617 OX=1392231 GN=eno PE=3 SV=1
                         2::A0A1U9WQN1 >tr|A0A1U9WQN1|A0A1U9WQN1_9CAUD Thymidylate synthase OS=Geobacillus phage TP-84 OX=1965361 PE=4 SV=1
                         2::A0A1U9WQP5 >tr|A0A1U9WQP5|A0A1U9WQP5_9CAUD Capsid portal protein OS=Geobacillus phage TP-84 OX=1965361 PE=4 SV=1
                         2::A0A1U9WQR1 >tr|A0A1U9WQR1|A0A1U9WQR1_9CAUD Tail protein OS=Geobacillus phage TP-84 OX=1965361 PE=4 SV=1
                         2::A0A499SQI6 >tr|A0A499SQI6|A0A499SQI6_9CAUD Iron-sulfur cluster assembly protein OS=Staphylococcus phage phiSP44-1 OX=2491321 PE=4 SV=1
                         2::A0A2D1GPX2 >tr|A0A2D1GPX2|A0A2D1GPX2_9CAUD DNA recombinase OS=Mycobacterium phage Kumao OX=2041344 GN=88 PE=3 SV=1
                         2::A0A0B5CY24 >tr|A0A0B5CY24|A0A0B5CY24_9HIV1 Pol protein (Fragment) OS=Human immunodeficiency virus 1 OX=11676 GN=pol PE=3 SV=1
                         2::A0A1U9WQR2 >tr|A0A1U9WQR2|A0A1U9WQR2_9CAUD Uncharacterized protein OS=Geobacillus phage TP-84 OX=1965361 PE=4 SV=1
                         2::A0A0G2Y0A6 >tr|A0A0G2Y0A6|A0A0G2Y0A6_MIMIV Heat shock protein 70-like protein OS=Acanthamoeba polyphaga mimivirus OX=212035 PE=4 SV=1
                         2::A0A3T0IGV1 >tr|A0A3T0IGV1|A0A3T0IGV1_9CAUD dCMP deaminase OS=Acinetobacter phage AbTZA1 OX=2500827 PE=4 SV=1
                         2::A0A218MLN2 >tr|A0A218MLN2|A0A218MLN2_9VIRU Putative structural protein OS=uncultured virus OX=340016 PE=4 SV=1
                         2::A0A0H4INX8 >tr|A0A0H4INX8|A0A0H4INX8_9CAUD Uncharacterized protein OS=Pseudoalteromonas phage H101 OX=1654919 PE=4 SV=1
                         2::A0A0A8J9R3 >tr|A0A0A8J9R3|A0A0A8J9R3_9CAUD Uncharacterized protein OS=Klebsiella phage K64-1 OX=1439894 PE=4 SV=1
                         2::G0T543     >tr|G0T543|G0T543_IRV9 TFIIS-type domain-containing protein OS=Wiseana iridescent virus OX=68347 PE=4 SV=1
                         2::A0A1V0SDT3 >tr|A0A1V0SDT3|A0A1V0SDT3_9VIRU Uncharacterized protein OS=Indivirus ILV1 OX=1977633 GN=Indivirus_4_44 PE=4 SV=1
                         2::A0A2H4UZZ7 >tr|A0A2H4UZZ7|A0A2H4UZZ7_9ABAC ORF97 protein OS=Operophtera brumata nucleopolyhedrovirus OX=1046267 PE=4 SV=1
                         2::A0A1V0DZB4 >tr|A0A1V0DZB4|A0A1V0DZB4_9CAUD Replicative DNA helicase OS=Acinetobacter phage WCHABP12 OX=1965454 GN=dnaB PE=4 SV=1
                         2::A0A3Q8J4E7 >tr|A0A3Q8J4E7|A0A3Q8J4E7_9GAMA Glycoprotein B OS=Vombatid gammaherpesvirus 1 OX=2052651 GN=ORF8 PE=4 SV=1
                         2::A0A059UHZ7 >tr|A0A059UHZ7|A0A059UHZ7_9RETR Pol protein (Fragment) OS=Feline immunodeficiency virus OX=11673 GN=pol PE=3 SV=1
                         2::A0A1L2CVK2 >tr|A0A1L2CVK2|A0A1L2CVK2_9CAUD Uncharacterized protein OS=Pectobacterium phage CBB OX=1792242 GN=CBB_463 PE=4 SV=1
                         2::A0A0P0KQU0 >tr|A0A0P0KQU0|A0A0P0KQU0_9HEPC Genome polyprotein (Fragment) OS=Hepatitis C virus subtype 1a OX=31646 PE=4 SV=1
                         2::A0A1B1IVR3 >tr|A0A1B1IVR3|A0A1B1IVR3_9VIRU Excinuclease ABC subunit A OS=uncultured Mediterranean phage OX=1868660 PE=3 SV=1
                         2::A0A0A0Y6T0 >tr|A0A0A0Y6T0|A0A0A0Y6T0_WSSVS Wsv216 OS=White spot syndrome virus (isolate Shrimp/China/Tongan/1996) OX=654913 PE=4 SV=1
                         2::A0A482MLD2 >tr|A0A482MLD2|A0A482MLD2_9CAUD Uncharacterized protein OS=Caudovirales GX15bay OX=2530034 PE=4 SV=1
                         2::G8XUC3     >tr|G8XUC3|G8XUC3_9BETA Large tegument protein OS=Aotine betaherpesvirus 1 OX=50290 GN=UL48 PE=3 SV=1
                         2::A0A3G4ZY70 >tr|A0A3G4ZY70|A0A3G4ZY70_9VIRU Uncharacterized protein OS=Gaeavirus sp. OX=2487767 GN=Gaeavirus1_3 PE=4 SV=1
                         2::A0A075Q9S7 >tr|A0A075Q9S7|A0A075Q9S7_9HIV1 Protein Nef OS=Human immunodeficiency virus 1 OX=11676 GN=nef PE=2 SV=1
                         2::A0A0K2CZZ1 >tr|A0A0K2CZZ1|A0A0K2CZZ1_9CAUD Putative phosphoadenosine phosphosulfate reductase OS=Bacillus phage TsarBomba OX=1690456 GN=TSARBOMBA_18 PE=4 SV=1
                         2::A0A223W084 >tr|A0A223W084|A0A223W084_9CAUD Uncharacterized protein OS=Agrobacterium phage Atu_ph08 OX=2024265 PE=4 SV=1
                         2::A0A516LXB0 >tr|A0A516LXB0|A0A516LXB0_9VIRU Uncharacterized protein OS=Prokaryotic dsDNA virus sp. OX=2591644 GN=Unbinned175contig1000_19 PE=4 SV=1
                         2::A0A1B1IS87 >tr|A0A1B1IS87|A0A1B1IS87_9VIRU Uncharacterized protein OS=uncultured Mediterranean phage OX=1868660 PE=4 SV=1
                         2::A0A1D8MBQ4 >tr|A0A1D8MBQ4|A0A1D8MBQ4_9VIRU Polyprotein (Fragment) OS=Endornavirus-like virus OX=1906952 PE=4 SV=1
                         2::H2BCQ0     >tr|H2BCQ0|H2BCQ0_9CAUD Uncharacterized protein OS=Synechococcus phage S-CBS4 OX=756275 GN=S-CBS4_gp009 PE=4 SV=1
                         2::Q75491     >tr|Q75491|Q75491_9HIV1 Envelope glycoprotein (Fragment) OS=Human immunodeficiency virus 1 OX=11676 GN=env PE=4 SV=1
                         2::A0A068EKL1 >tr|A0A068EKL1|A0A068EKL1_9POXV Ankyrin repeat protein OS=Pigeonpox virus OX=10264 GN=fep_016 PE=4 SV=1
                         2::A0A0A0QBC3 >tr|A0A0A0QBC3|A0A0A0QBC3_9CIRC Replication-associated protein (Fragment) OS=Circovirus VS6600032 OX=1525174 PE=4 SV=1
                         2::A0A1V0SKV6 >tr|A0A1V0SKV6|A0A1V0SKV6_9VIRU Uncharacterized protein OS=Klosneuvirus KNV1 OX=1977640 GN=Klosneuvirus_5_30 PE=4 SV=1
                         2::A0A160DAA3 >tr|A0A160DAA3|A0A160DAA3_9ALPC Spike glycoprotein OS=Porcine epidemic diarrhea virus OX=28295 GN=S PE=2 SV=1
                         2::H6WG71     >tr|H6WG71|H6WG71_9CAUD Uncharacterized protein OS=Cyanophage S-TIM5 OX=1137745 PE=4 SV=1
                         1::C1JCT2     Polyprotein-FSD OS=Solenopsis invicta virus 3 OX=631345 PE=1 SV=2
                         2::A0A401IPN0 >tr|A0A401IPN0|A0A401IPN0_9VIRU Wsv514-like protein OS=Sesarmops intermedium nimavirus OX=2133796 PE=4 SV=1
                         2::Q6RU22     >tr|Q6RU22|Q6RU22_9HIV1 Gag protein (Fragment) OS=Human immunodeficiency virus 1 OX=11676 GN=gag PE=4 SV=1
                         2::A0A2D2W767 >tr|A0A2D2W767|A0A2D2W767_9CAUD Uncharacterized protein OS=Pectobacterium phage DU_PP_V OX=2041492 GN=P13BB106kb_p046 PE=4 SV=1
                         2::A0A482MM07 >tr|A0A482MM07|A0A482MM07_9CAUD Uncharacterized protein OS=Burkholderia phage BcepSauron OX=2530033 GN=BcepSauron_325 PE=4 SV=1
                         2::A0A4D6NVF7 >tr|A0A4D6NVF7|A0A4D6NVF7_9VIRU Structural protein (Fragment) OS=Melipona quadrifasciata virus 1b OX=2571177 GN=SP PE=4 SV=1
                         2::A0A023VZE8 >tr|A0A023VZE8|A0A023VZE8_BSMV Beta-B protein OS=Barley stripe mosaic virus OX=12327 PE=4 SV=1
                         2::A0A0M3SUP6 >tr|A0A0M3SUP6|A0A0M3SUP6_9VIRU RNA-dependent RNA polymerase (Fragment) OS=Rhizoctonia solani beny-like virus 1 OX=1708385 PE=4 SV=1
                         2::A0A482JV29 >tr|A0A482JV29|A0A482JV29_9RHAB RNA-directed RNA polymerase L OS=Lampyris noctiluca rhabdo-like virus 1 OX=2552994 PE=3 SV=1
                         2::A0A023NH97 >tr|A0A023NH97|A0A023NH97_9CAUD Putative ribonucleoside-diphosphate redutase OS=Dinoroseobacter phage DFL12phi1 OX=1477404 GN=DFL12P1_0034 PE=4 SV=1
                         2::A0A1E1GE69 >tr|A0A1E1GE69|A0A1E1GE69_9CAUD Putative primase OS=Vibrio phage CKB-S1 OX=1903259 PE=4 SV=1
                         2::A0A1W6RAJ7 >tr|A0A1W6RAJ7|A0A1W6RAJ7_9ENTO Capsid protein VP1 (Fragment) OS=Echovirus E16 OX=47504 PE=4 SV=1
                         2::A0A1W6JEN0 >tr|A0A1W6JEN0|A0A1W6JEN0_9BETA Uncharacterized protein OS=Human betaherpesvirus 6 OX=10368 PE=4 SV=1
                         2::A0A097BY06 >tr|A0A097BY06|A0A097BY06_9CAUD Minor tail protein OS=Mycobacterium phage Power OX=1552733 GN=PBI_POWER_31 PE=4 SV=1
                         2::D7SGS7     >tr|D7SGS7|D7SGS7_9MONO Polymerase (Fragment) OS=Paramyxovirus bat/GH47/2008 OX=589524 GN=L PE=4 SV=1
                         2::A0A088B1A8 >tr|A0A088B1A8|A0A088B1A8_FRG3V Uncharacterized protein OS=Chinese giant salamander iridovirus OX=1213990 PE=4 SV=1
                         2::A0A0A7RU81 >tr|A0A0A7RU81|A0A0A7RU81_9CAUD Tail fiber protein OS=Clostridium phage phiCT19406B OX=1567010 GN=phiCT19406B_53 PE=4 SV=1
                         2::A0A0F6WEX4 >tr|A0A0F6WEX4|A0A0F6WEX4_9CAUD DnaB helicase OS=Mycobacterium phage Mindy OX=1647311 GN=80 PE=4 SV=1
                         2::A0A0M5M5R9 >tr|A0A0M5M5R9|A0A0M5M5R9_9CAUD SsDNA-binding protein OS=Citrobacter phage Margaery OX=1701810 GN=CPT_Margaery222 PE=4 SV=1
                         2::A0A0D3MVZ4 >tr|A0A0D3MVZ4|A0A0D3MVZ4_9ADEN Ig-like domain-containing protein OS=Duck adenovirus 1 OX=130329 PE=4 SV=1
                         2::A0A1D8EZ70 >tr|A0A1D8EZ70|A0A1D8EZ70_9CAUD Lysin A OS=Mycobacterium phage Badger OX=1881140 GN=SEA_BADGER_8 PE=4 SV=1
                         2::A0A516LNF5 >tr|A0A516LNF5|A0A516LNF5_9VIRU Peptidase S74 domain-containing protein OS=Prokaryotic dsDNA virus sp. OX=2591644 GN=GOVbin3762_66 PE=4 SV=1
                         2::A0A076JU09 >tr|A0A076JU09|A0A076JU09_9ALPH Portal protein OS=Equid alphaherpesvirus 1 OX=10326 PE=3 SV=1
                         2::A0A4D5ZFG5 >tr|A0A4D5ZFG5|A0A4D5ZFG5_9VIRU Uncharacterized protein OS=Streptococcus satellite phage Javan147 OX=2558541 GN=JavanS147_0011 PE=4 SV=1
                         2::A0A160DHM9 >tr|A0A160DHM9|A0A160DHM9_9CAUD Uncharacterized protein OS=Gordonia phage Jeanie OX=1838068 GN=3 PE=4 SV=1
                         2::A0A4D6BFQ7 >tr|A0A4D6BFQ7|A0A4D6BFQ7_9CAUD Major capsid protein OS=Streptococcus phage Javan570 OX=2548265 GN=Javan570_0040 PE=4 SV=1
                         2::A0A3G8FE45 >tr|A0A3G8FE45|A0A3G8FE45_9CAUD SPP1 gp7 family head morphogenesis protein OS=Phage NGI4 OX=2489188 PE=4 SV=1
                         2::A0A3S7SGD4 >tr|A0A3S7SGD4|A0A3S7SGD4_9CAUD Uncharacterized protein OS=Aerococcus phage vB_AviM_AVP OX=2495576 GN=AVP_16 PE=4 SV=1
                         2::A0A0K0KVG1 >tr|A0A0K0KVG1|A0A0K0KVG1_9CAUD Putative neck protein OS=Prochlorococcus phage P-TIM68 OX=1542477 PE=4 SV=1
                         2::A0A345MNE4 >tr|A0A345MNE4|A0A345MNE4_9CAUD Helicase domain protein OS=Myoviridae sp. OX=2202564 PE=4 SV=1
                         2::A0A0G2SSE8 >tr|A0A0G2SSE8|A0A0G2SSE8_9CAUD Tail protein/adaptor protein OS=Proteus phage vB_PmiP_Pm5460 OX=1636249 GN=Pm5460_41 PE=4 SV=1
                         2::A0A0S1M392 >tr|A0A0S1M392|A0A0S1M392_9PICO Genome polyprotein OS=Hedgehog hepatovirus OX=1745249 PE=4 SV=1
                         2::C8CJF1     >tr|C8CJF1|C8CJF1_9RHAB RNA-directed RNA polymerase L OS=Drosophila obscura sigmavirus 10A OX=666962 GN=L PE=3 SV=1
                         2::A0A0F6R7E5 >tr|A0A0F6R7E5|A0A0F6R7E5_9CAUD Uncharacterized protein OS=Escherichia coli O157 typing phage 8 OX=1508683 GN=ECTP8_01430 PE=4 SV=1
                         2::A0A385IRN7 >tr|A0A385IRN7|A0A385IRN7_9CAUD Putative colanic acid-degrading protein OS=Escherichia phage vB_EcoM_IME392 OX=2301649 PE=4 SV=1
                         2::A0A1Q1G599 >tr|A0A1Q1G599|A0A1Q1G599_9HIV1 Pol protein (Fragment) OS=Human immunodeficiency virus 1 OX=11676 GN=pol PE=4 SV=1
                         2::A0A1Z1LZM8 >tr|A0A1Z1LZM8|A0A1Z1LZM8_9CAUD Uncharacterized protein OS=Bacillus phage Deep-Purple OX=1873341 GN=DeepPurple_gp035 PE=4 SV=1
                         2::A0A140G5X8 >tr|A0A140G5X8|A0A140G5X8_9CAUD Putative scaffold protein OS=Enterobacteria phage ECGD1 OX=1784948 GN=ECGD1_130 PE=4 SV=1
                         2::L0H229     >tr|L0H229|L0H229_9HIV1 Envelope glycoprotein (Fragment) OS=Human immunodeficiency virus 1 OX=11676 GN=env PE=4 SV=1
                         2::E5E431     >tr|E5E431|E5E431_9CAUD Uncharacterized protein OS=Acinetobacter phage Acj61 OX=760732 GN=Acj61p050 PE=4 SV=1
                         2::A0A223LF67 >tr|A0A223LF67|A0A223LF67_9CAUD Tail sheath protein OS=Aeromonas phage AS-gz OX=2026082 PE=4 SV=1
                         2::A0A5C1K3U7 >tr|A0A5C1K3U7|A0A5C1K3U7_9MONO Large structural protein OS=Guadeloupe mosquito mononega-like virus OX=2607732 GN=orf6 PE=4 SV=1
                         2::V9VL70     >tr|V9VL70|V9VL70_9GAMC Nucleocapsid protein (Fragment) OS=Infectious bronchitis virus OX=11120 GN=N PE=4 SV=1
                         2::Q27VW2     >tr|Q27VW2|Q27VW2_9HIV1 Envelope glycoprotein (Fragment) OS=Human immunodeficiency virus 1 OX=11676 GN=env PE=4 SV=1
                         2::A0A1U8ZS54 >tr|A0A1U8ZS54|A0A1U8ZS54_9VIRU Nucleoprotein OS=Lassa mammarenavirus OX=11620 GN=N PE=3 SV=1
                         2::A0A516LSU6 >tr|A0A516LSU6|A0A516LSU6_9VIRU Putative 60 kDa chaperonin OS=Prokaryotic dsDNA virus sp. OX=2591644 GN=GOVbin1782_22 PE=4 SV=1
                         2::A0A1D6Y7D3 >tr|A0A1D6Y7D3|A0A1D6Y7D3_9VIRU Putative mRNA-capping enzyme OS=Golden Marseillevirus OX=1720526 GN=GMAR_ORF208 PE=4 SV=1
                         2::A0A2H4UVE2 >tr|A0A2H4UVE2|A0A2H4UVE2_9VIRU Serine/threonine-protein kinase OS=Bodo saltans virus OX=2024608 GN=BMW23_0847 PE=4 SV=1
                         2::A0A0R6PJK4 >tr|A0A0R6PJK4|A0A0R6PJK4_9CAUD Haemagg_act domain-containing protein OS=Moraxella phage Mcat25 OX=1647541 PE=4 SV=1
                         2::A0A482MH24 >tr|A0A482MH24|A0A482MH24_9CAUD Uncharacterized protein OS=Serratia phage Serbin OX=2562181 GN=CPT_Serbin_039 PE=4 SV=1
                         2::A0A1U7ET45 >tr|A0A1U7ET45|A0A1U7ET45_9VIRU 126 kDa protein OS=Odontoglossum ringspot virus OX=12238 PE=4 SV=1
                         2::A0A0U2UDM8 >tr|A0A0U2UDM8|A0A0U2UDM8_9HIV1 Pol protein (Fragment) OS=Human immunodeficiency virus 1 OX=11676 PE=3 SV=1
```

	Target	Decoy	False discovery rate
Peptide matches above identity threshold	180	116	64.44 %
Peptide matches above homology or identity threshold	270	189	70.00 %

Select Summary Report

Format As [Select Summary (protein hits)] Help Help

Significance threshold p< [0.05] Max. number of hits [AUTO] Show Percolator scores ☐

Standard scoring ○ MudPIT scoring ◉ Ions score or expect cut-off [40] Show sub-sets [0]

Show pop-ups ○ Suppress pop-ups ◉ Require bold red ☑

Preferred taxonomy [All entries]

Re-Search ◉ All queries ○ Unassigned ○ Below homology threshold ○ Below identity threshold

1. 2::A0A1U9WQS0 **Mass:** 112893 **Score:** 1406 **Matches:** 25(25) **Sequences:** 17(17) **emPAI:** 0.96
>tr|A0A1U9WQS0|A0A1U9WQS0_9CAUD Glycosylase OS=Geobacillus phage TP-84 OX=1965361 PE=4 SV=1

Query	Observed	Mr(expt)	Mr(calc)	ppm	Miss	Score	Expect	Rank	Unique	Peptide
70	382.2394	762.4643	762.4639	0.53	0	40	0.027	1	U	K.YINLLK.R
285	438.7433	875.4721	875.4712	1.00	0	77	7.8e-06	1	U	K.LSIGTTER.W 286
359	454.7371	907.4597	907.4586	1.22	0	44	0.0095	1	U	R.VMTWSLR.I
365	455.7288	909.4429	909.4444	-1.54	0	68	3e-05	1	U	K.LFAESTDK.N
617	501.3031	1000.5917	1000.5917	-0.03	0	115	6.6e-10	1	U	K.ISGLAGVTGVK.G 618
879	539.8011	1077.5876	1077.5859	1.58	0	51	0.0025	1	U	K.LIDAGLWYK.Q
1193	579.2658	1156.5171	1156.5183	-1.00	0	(78)	2.9e-06	1	U	R.YDSMGQTTVR.V
1215	582.3091	1162.6037	1162.6022	1.22	0	55	0.0018	1	U	R.TLWPNYIEK.D
1257	587.2636	1172.5125	1172.5132	-0.56	0	85	2.5e-07	1	U	R.YDSMGQTTVR.V
1740	649.8350	1297.6554	1297.6554	-0.03	0	52	0.0024	1	U	R.FSVIIDGQEYK.M 1739
2053	687.8500	1373.6854	1373.6827	2.01	0	83	1.6e-06	1	U	K.NEVIIDANPDFK.L 2054
2080	691.3820	1380.7495	1380.7514	-1.39	0	61	0.00015	1	U	K.GIGLWVLGQADPR.I
2645	808.3812	1614.7478	1614.7460	1.06	0	110	5.3e-09	1	U	K.CTIDNVYLGGAGYGR.R
2891	847.8991	1693.7836	1693.7835	0.08	0	117	5.4e-10	1	U	R.VSEYNGIEYATAYSK.T
3078	877.9213	1753.8280	1753.8271	0.50	0	110	2.4e-09	1	U	K.GVSEPTHNLSPYDNPK.R
3079	585.6171	1753.8295	1753.8271	1.35	0	(90)	1.9e-07	1	U	K.GVSEPTHNLSPYDNPK.R
3327	917.4712	1832.9278	1832.9268	0.53	0	160	3.9e-14	1	U	R.LLDSQTAQDNFINNLK.T 3328
3438	941.9081	1881.8016	1881.8017	-0.07	0	129	5.1e-12	1	U	K.DAQGSFTSFTDATTYDR.Y
3836	1060.0066	2117.9986	2118.0058	-3.43	0	62	0.00028	1	U	R.IFETLPDVVPWHPDPNEN.- 3835

2. 2::W6LP63 **Mass:** 47022 **Score:** 923 **Matches:** 12(12) **Sequences:** 4(4) **emPAI:** 0.56
>tr|W6LP63|W6LP63_9CAUD Enolase OS=Streptococcus phage 20617 OX=1392231 GN=eno PE=3 SV=1

Query	Observed	Mr(expt)	Mr(calc)	ppm	Miss	Score	Expect	Rank	Unique	Peptide
77	386.2055	770.3964	770.3963	0.23	0	54	0.00066	1	U	K.SFYNLK.K
116	403.7296	805.4446	805.4446	-0.04	0	51	0.0044	1	U	K.YNQLLR.I 117
3565	990.9989	1979.9832	1979.9874	-2.13	0	152	1.7e-13	1	U	K.VNQIGTLTETFDAIEMAK.E 3566
3567	661.0024	1979.9855	1979.9874	-0.97	0	(106)	6.9e-09	1	U	K.VNQIGTLTETFDAIEMAK.E 3564
3594	998.9956	1995.9766	1995.9823	-2.83	0	(136)	6.8e-12	1	U	K.VNQIGTLTETFDAIEMAK.E 3597
3595	666.3335	1995.9787	1995.9823	-1.83	0	(76)	8.2e-06	1	U	K.VNQIGTLTETFDAIEMAK.E 3596
3946	1096.0404	2190.0663	2190.0652	0.53	0	154	4.2e-13	1	U	R.SGETEDSTIADIAVATNAGQIK.T

3. 2::A0A1U9WQN1 **Mass:** 38540 **Score:** 403 **Matches:** 6(6) **Sequences:** 5(5) **emPAI:** 0.73
>tr|A0A1U9WQN1|A0A1U9WQN1_9CAUD Thymidylate synthase OS=Geobacillus phage TP-84 OX=1965361 PE=4 SV=1

Query	Observed	Mr(expt)	Mr(calc)	ppm	Miss	Score	Expect	Rank	Unique	Peptide
1133	568.8068	1135.5991	1135.5985	0.46	1	65	0.00011	1	U	K.KGEISGNYIR.D
1968	677.8367	1353.6588	1353.6598	-0.75	0	78	1.4e-05	1	U	K.VAGYEEAIMSLR.M
2136	696.8285	1391.6425	1391.6425	0.03	0	111	2.2e-09	1	U	K.QGMEMLADQIEK.H
3075	584.9219	1751.7440	1751.7461	-1.20	0	57	7.5e-05	1	U	R.YELTDTGEWEHMNK.V
3505	968.9857	1935.9569	1935.9578	-0.44	0	106	9.8e-09	1	U	R.LANIQETVLSEWYQDK.I 3504

4. 2::A0A1U9WQP5 **Mass:** 62259 **Score:** 256 **Matches:** 4(4) **Sequences:** 4(4) **emPAI:** 0.31
>tr|A0A1U9WQP5|A0A1U9WQP5_9CAUD Capsid portal protein OS=Geobacillus phage TP-84 OX=1965361 PE=4 SV=1

Query	Observed	Mr(expt)	Mr(calc)	ppm	Miss	Score	Expect	Rank	Unique	Peptide
599	497.7640	993.5135	993.5131	0.38	0	75	2.6e-05	1	U	R.SELYNLAGK.Q
940	546.2908	1090.5670	1090.5659	1.07	0	63	0.00061	1	U	K.EIFQALQDK.A
1673	638.3201	1274.6256	1274.6255	0.05	0	94	2.9e-07	1	U	K.QDEINWTLTR.N
3783	1042.5467	2083.0789	2083.0797	-0.38	0	134	1.3e-11	1	U	R.LIQEGEITDQIIEGAEVAR.N

5. 2::A0A1U9WQR1 **Mass:** 99414 **Score:** 209 **Matches:** 4(4) **Sequences:** 4(4) **emPAI:** 0.19
>tr|A0A1U9WQR1|A0A1U9WQR1_9CAUD Tail protein OS=Geobacillus phage TP-84 OX=1965361 PE=4 SV=1

Query	Observed	Mr(expt)	Mr(calc)	ppm	Miss	Score	Expect	Rank	Unique	Peptide
808	529.2790	1056.5434	1056.5451	-1.62	0	73	1.5e-05	1	U	K.TEVELNQPK.K
1061	561.2954	1120.5762	1120.5764	-0.17	0	84	1.2e-06	1	U	K.VINYEQVEK.T
1150	571.8194	1141.6242	1141.6244	-0.15	0	65	0.00016	1	U	R.FGSPAAIGPGIR.G
2132	695.9138	1389.8131	1389.8119	0.85	0	67	6.7e-06	1	U	R.LTLTSEPLIIYK.D

6. 2::A0A499SQI6 **Mass:** 52717 **Score:** 116 **Matches:** 2(2) **Sequences:** 2(2) **emPAI:** 0.17
>tr|A0A499SQI6|A0A499SQI6_9CAUD Iron-sulfur cluster assembly protein OS=Staphylococcus phage phiSP44-1 OX=2491321 PE=4 SV=1

Query	Observed	Mr(expt)	Mr(calc)	ppm	Miss	Score	Expect	Rank	Unique	Peptide
564	492.7718	983.5291	983.5287	0.34	0	57	0.00031	1	U	K.LGIPEAEQK.Y
2445	751.3696	1500.7246	1500.7282	-2.44	0	84	4.3e-06	1	U	K.VSEEQLFYLMSR.G

7. 2::A0A2D1GPX2 **Mass:** 35002 **Score:** 104 **Matches:** 2(2) **Sequences:** 1(1) **emPAI:** 0.13
>tr|A0A2D1GPX2|A0A2D1GPX2_9CAUD DNA recombinase OS=Mycobacterium phage Kumao OX=2041344 GN=88 PE=3 SV=1

Query	Observed	Mr(expt)	Mr(calc)	ppm	Miss	Score	Expect	Rank	Unique	Peptide
1689	639.8323	1277.6500	1277.6503	-0.25	0	84	4e-06	1	U	R.IVEIYGPESSGK.T 1690

8. 2::A0A0B5CY24 **Mass:** 39036 **Score:** 103 **Matches:** 2(2) **Sequences:** 1(1) **emPAI:** 0.11
>tr|A0A0B5CY24|A0A0B5CY24_9HIV1 Pol protein (Fragment) OS=Human immunodeficiency virus 1 OX=11676 GN=pol PE=3 SV=1

Query	Observed	Mr(expt)	Mr(calc)	ppm	Miss	Score	Expect	Rank	Unique	Peptide
166	413.2442	824.4738	824.4756	-2.16	0	66	1.2e-05	1	U	K.LSDHLLK.W 167

Proteins matching the same set of peptides:
2::I0CGY4 **Mass:** 37204 **Score:** 103 **Matches:** 2(2) **Sequences:** 1(1)
>tr|I0CGY4|I0CGY4_9HIV1 Pol protein (Fragment) OS=Human immunodeficiency virus 1 OX=11676 GN=pol PE=3 SV=1

9. 2::A0A1U9WQR2 **Mass:** 19335 **Score:** 83 **Matches:** 2(2) **Sequences:** 2(2) **emPAI:** 0.54
>tr|A0A1U9WQR2|A0A1U9WQR2_9CAUD Uncharacterized protein OS=Geobacillus phage TP-84 OX=1965361 PE=4 SV=1

Query	Observed	Mr(expt)	Mr(calc)	ppm	Miss	Score	Expect	Rank	Unique	Peptide
1065	562.2661	1122.5177	1122.5193	-1.44	0	60	0.00043	1	U	K.ESQQFEIDK.I
2259	718.8652	1435.7158	1435.7163	-0.36	0	44	0.014	1	U	R.AMNLLSIMSGIDR.E

10. 2::A0A0G2Y0A6 **Mass:** 107753 **Score:** 77 **Matches:** 2(2) **Sequences:** 2(2) **emPAI:** 0.08
>tr|A0A0G2Y0A6|A0A0G2Y0A6_MIMIV Heat shock protein 70-like protein OS=Acanthamoeba polyphaga mimivirus OX=212035 PE=4 SV=1

Query	Observed	Mr(expt)	Mr(calc)	ppm	Miss	Score	Expect	Rank	Unique	Peptide
327	451.2783	900.5421	900.5393	3.18	0	49	0.01	1	U	K.VILVGGSTR.I
2548	782.9109	1563.8072	1563.8045	1.72	0	60	0.00081	1	U	K.AVITVPAYFNDAQR.Q

Proteins matching the same set of peptides:
2::A0A0G2Y835 **Mass:** 107638 **Score:** 77 **Matches:** 2(2) **Sequences:** 2(2)
>tr|A0A0G2Y835|A0A0G2Y835_MIMIV Heat shock protein 70-like protein OS=Acanthamoeba polyphaga mimivirus OX=212035 PE=4 SV=1
2::A0A0G2YCR2 **Mass:** 107765 **Score:** 77 **Matches:** 2(2) **Sequences:** 2(2)
>tr|A0A0G2YCR2|A0A0G2YCR2_MIMIV Heat shock 70Kd HSP OS=Acanthamoeba polyphaga mimivirus OX=212035 GN=L254 PE=4 SV=1
2::A0A0U2K379 **Mass:** 107765 **Score:** 77 **Matches:** 2(2) **Sequences:** 2(2)
>tr|A0A0U2K379|A0A0U2K379_9VIRU Heat shock 70Kd HSP OS=Niemeyer virus OX=1766765 PE=4 SV=1
2::A0A140E0K5 **Mass:** 107765 **Score:** 77 **Matches:** 2(2) **Sequences:** 2(2)
>tr|A0A140E0K5|A0A140E0K5_MIMIV Heat shock protein 70-like protein OS=Samba virus OX=1461100 PE=4 SV=1
2::A0A165XD09 **Mass:** 107765 **Score:** 77 **Matches:** 2(2) **Sequences:** 2(2)
>tr|A0A165XD09|A0A165XD09_MIMIV Heat shock protein 70-like protein OS=Mimivirus Bombay OX=1835008 PE=4 SV=1
2::A0A1E1EVQ4 **Mass:** 107765 **Score:** 77 **Matches:** 2(2) **Sequences:** 2(2)
>tr|A0A1E1EVQ4|A0A1E1EVQ4_9VIRU Heat shock protein 70-like protein OS=Acanthamoeba castellanii mimivirus OX=1899318 PE=4 SV=1
2::J2YWD0 **Mass:** 107765 **Score:** 77 **Matches:** 2(2) **Sequences:** 2(2)
>tr|J2YWD0|J2YWD0_9VIRU Heat shock 70Kd HSP OS=Acanthamoeba polyphaga lentillevirus OX=1077221 GN=L206 PE=4 SV=1
1::Q5UPU0 **Mass:** 107765 **Score:** 77 **Matches:** 2(2) **Sequences:** 2(2)
Heat shock protein 70 homolog OS=Acanthamoeba polyphaga mimivirus OX=212035 GN=MIMI_L254 PE=3 SV=1
2::V5L598 **Mass:** 107765 **Score:** 77 **Matches:** 2(2) **Sequences:** 2(2)
>tr|V5L598|V5L598_9VIRU 70 kDa heat shock protein-like protein OS=Hirudovirus strain Sangsue OX=1420594 GN=HIRU_S708 PE=4 SV=1
2::G8ECW7 **Mass:** 107765 **Score:** 77 **Matches:** 2(2) **Sequences:** 2(2)
>tr|G8ECW7|G8ECW7_9VIRU Heat shock 70Kd HSP OS=Acanthamoeba castellanii mamavirus OX=554168 GN=MAMA_L321 PE=4 SV=1

---

11. 2::A0A3T0IGV1 **Mass:** 20343 **Score:** 77 **Matches:** 1(1) **Sequences:** 1(1) **emPAI:** 0.23
>tr|A0A3T0IGV1|A0A3T0IGV1_9CAUD dCMP deaminase OS=Acinetobacter phage AbTZA1 OX=2500827 PE=4 SV=1

Query	Observed	Mr(expt)	Mr(calc)	ppm	Miss	Score	Expect	Rank	Unique	Peptide
137	407.7547	813.4949	813.4960	-1.34	0	77	5.6e-06	1	U	K.ILEAAGIK.V

Proteins matching the same set of peptides:
2::A0A4Y1NLK6 **Mass:** 20382 **Score:** 77 **Matches:** 1(1) **Sequences:** 1(1)
>tr|A0A4Y1NLK6|A0A4Y1NLK6_9CAUD dCMP deaminase OS=Acinetobacter phage AM101 OX=2178927 GN=AM101_211 PE=4 SV=1

---

12. 2::A0A218MLN2 **Mass:** 43019 **Score:** 75 **Matches:** 3(3) **Sequences:** 2(2) **emPAI:** 0.22
>tr|A0A218MLN2|A0A218MLN2_9VIRU Putative structural protein OS=uncultured virus OX=340016 PE=4 SV=1

Query	Observed	Mr(expt)	Mr(calc)	ppm	Miss	Score	Expect	Rank	Unique	Peptide
2	351.7042	701.3938	701.3959	-3.11	0	50	0.0048	1	U	K.VEEVVK.E 1
176	415.7523	829.4900	829.4909	-1.03	1	55	0.0012	1	U	K.KVEEVVK.E

---

13. 2::A0A0H4INX8 **Mass:** 22107 **Score:** 70 **Matches:** 2(2) **Sequences:** 1(1) **emPAI:** 0.21
>tr|A0A0H4INX8|A0A0H4INX8_9CAUD Uncharacterized protein OS=Pseudoalteromonas phage H101 OX=1654919 PE=4 SV=1

Query	Observed	Mr(expt)	Mr(calc)	ppm	Miss	Score	Expect	Rank	Unique	Peptide
343	453.2561	904.4976	904.4978	-0.20	0	55	0.0009	1	U	R.TSTIASIGR.G 344

---

14. 2::A0A0A8J9R3 **Mass:** 382428 **Score:** 70 **Matches:** 2(2) **Sequences:** 1(1) **emPAI:** 0.01
>tr|A0A0A8J9R3|A0A0A8J9R3_9CAUD Uncharacterized protein OS=Klebsiella phage K64-1 OX=1439894 PE=4 SV=1

Query	Observed	Mr(expt)	Mr(calc)	ppm	Miss	Score	Expect	Rank	Unique	Peptide
19	361.6948	721.3750	721.3759	-1.30	0	54	0.0016	1	U	K.YGDLVR.T 20

Proteins matching the same set of peptides:
2::H6X3M7 **Mass:** 382912 **Score:** 70 **Matches:** 2(2) **Sequences:** 1(1)
>tr|H6X3M7|H6X3M7_9CAUD Uncharacterized protein OS=Enterobacteria phage vB_KleM-RaK2 OX=1147094 GN=RaK2_0070 PE=4 SV=1

---

15. 2::G0T543 **Mass:** 16464 **Score:** 69 **Matches:** 2(2) **Sequences:** 1(1) **emPAI:** 0.29
>tr|G0T543|G0T543_IRV9 TFIIS-type domain-containing protein OS=Wiseana iridescent virus OX=68347 PE=4 SV=1

Query	Observed	Mr(expt)	Mr(calc)	ppm	Miss	Score	Expect	Rank	Unique	Peptide
528	486.2919	970.5693	970.5699	-0.60	0	54	0.0015	1	U	K.LEEVVAALK.N 529

---

16. 2::A0A1V0SDT3 **Mass:** 94076 **Score:** 69 **Matches:** 2(2) **Sequences:** 1(1) **emPAI:** 0.05
>tr|A0A1V0SDT3|A0A1V0SDT3_9VIRU Uncharacterized protein OS=Indivirus ILV1 OX=1977633 GN=Indivirus_4_44 PE=4 SV=1

Query	Observed	Mr(expt)	Mr(calc)	ppm	Miss	Score	Expect	Rank	Unique	Peptide
926	544.8039	1087.5933	1087.5913	1.78	0	52	0.0013	1	U	K.FINEIIPDK.F 927

---

17. 2::A0A2H4UZZ7 **Mass:** 43281 **Score:** 69 **Matches:** 2(2) **Sequences:** 1(1) **emPAI:** 0.10
>tr|A0A2H4UZZ7|A0A2H4UZZ7_9ABAC ORF97 protein OS=Operophtera brumata nucleopolyhedrovirus OX=1046267 PE=4 SV=1

Query	Observed	Mr(expt)	Mr(calc)	ppm	Miss	Score	Expect	Rank	Unique	Peptide
9	356.7029	711.3913	711.3915	-0.29	0	50	0.00076	1	U	K.SDHLIK.N 8

---

18. 2::A0A1V0DZB4 **Mass:** 48404 **Score:** 68 **Matches:** 2(2) **Sequences:** 1(1) **emPAI:** 0.09
>tr|A0A1V0DZB4|A0A1V0DZB4_9CAUD Replicative DNA helicase OS=Acinetobacter phage WCHABP12 OX=1965454 GN=dnaB PE=4 SV=1

Query	Observed	Mr(expt)	Mr(calc)	ppm	Miss	Score	Expect	Rank	Unique	Peptide
4	351.7053	701.3960	701.3959	0.08	0	54	0.0019	1	U	K.LDIVDK.A 3

Proteins matching the same set of peptides:
2::A0A1X6WG70 **Mass:** 156443 **Score:** 68 **Matches:** 2(2) **Sequences:** 1(1)
>tr|A0A1X6WG70|A0A1X6WG70_9VIRU Uncharacterized protein OS=Pacmanvirus A23 OX=1932881 GN=PACV_169 PE=4 SV=1
2::A0A1X9SFL1 **Mass:** 48404 **Score:** 68 **Matches:** 2(2) **Sequences:** 1(1)
>tr|A0A1X9SFL1|A0A1X9SFL1_9CAUD Replicative DNA helicase OS=Acinetobacter phage WCHABP1 OX=1983409 GN=dnaB PE=4 SV=1
2::A0A220NQM0 **Mass:** 48501 **Score:** 68 **Matches:** 2(2) **Sequences:** 1(1)
>tr|A0A220NQM0|A0A220NQM0_9CAUD Replicative DNA helicase OS=Acinetobacter phage AbP2 OX=2015804 GN=ABP2_079 PE=4 SV=1
2::A0A2L0UZ96 **Mass:** 87135 **Score:** 68 **Matches:** 2(2) **Sequences:** 1(1)
>tr|A0A2L0UZ96|A0A2L0UZ96_9CAUD Zeta_toxin domain-containing protein OS=Agrobacterium phage Atu_ph07 OX=2024264 PE=4 SV=1
2::A0A3G3BXJ7 **Mass:** 48461 **Score:** 68 **Matches:** 2(2) **Sequences:** 1(1)
>tr|A0A3G3BXJ7|A0A3G3BXJ7_9CAUD Replicative DNA helicase OS=Acinetobacter phage vB_KpnM_IME284 OX=2419625 PE=4 SV=1
2::A0A3G3BXU9 **Mass:** 48404 **Score:** 68 **Matches:** 2(2) **Sequences:** 1(1)
>tr|A0A3G3BXU9|A0A3G3BXU9_9CAUD Replicative DNA helicase OS=Acinetobacter phage vB_KpnM_IME512 OX=2419627 PE=4 SV=1
2::A0A482MGD6 **Mass:** 93180 **Score:** 68 **Matches:** 2(2) **Sequences:** 1(1)
>tr|A0A482MGD6|A0A482MGD6_9CAUD Tape measure protein OS=Serratia phage Serbin OX=2562181 GN=CPT_Serbin_021 PE=4 SV=1
2::A0A516LKU8 **Mass:** 7405 **Score:** 68 **Matches:** 2(2) **Sequences:** 1(1)
>tr|A0A516LKU8|A0A516LKU8_9VIRU Uncharacterized protein OS=Prokaryotic dsDNA virus sp. OX=2591644 GN=Tp118DCM00d2C30442581_17 PE=4 SV=1
2::A7DYD4 **Mass:** 35730 **Score:** 68 **Matches:** 2(2) **Sequences:** 1(1)
>tr|A7DYD4|A7DYD4_9CAUD Uncharacterized protein OS=Streptococcus phage M102 OX=372457 PE=4 SV=1

---

19. 2::A0A3Q8J4E7 **Mass:** 91595 **Score:** 68 **Matches:** 2(2) **Sequences:** 1(1) **emPAI:** 0.05
>tr|A0A3Q8J4E7|A0A3Q8J4E7_9GAMA Glycoprotein B OS=Vombatid gammaherpesvirus 1 OX=2052651 GN=ORF8 PE=4 SV=1

Query	Observed	Mr(expt)	Mr(calc)	ppm	Miss	Score	Expect	Rank	Unique	Peptide
11	358.7183	715.4220	715.4228	-1.16	0	58	0.0016	1	U	K.TVIIDR.T 13

Proteins matching the same set of peptides:
2::V5K6X0 **Mass:** 65777 **Score:** 68 **Matches:** 2(2) **Sequences:** 1(1)
>tr|V5K6X0|V5K6X0_9MONO Transmembrane protein OS=Miniopterus schreibersii paramyxovirus OX=1387879 GN=TM PE=4 SV=1

---

20. 2::A0A059UHZ7 **Mass:** 130888 **Score:** 68 **Matches:** 4(3) **Sequences:** 2(1) **emPAI:** 0.03
>tr|A0A059UHZ7|A0A059UHZ7_9RETR Pol protein (Fragment) OS=Feline immunodeficiency virus OX=11673 GN=pol PE=3 SV=1

Query	Observed	Mr(expt)	Mr(calc)	ppm	Miss	Score	Expect	Rank	Unique	Peptide
388	458.2613	914.5080	914.5073	0.79	0	40	0.14	2	U	K.QVADEIIK.K
750	348.5414	1042.6023	1042.6022	0.10	1	47	0.008	1	U	K.QVADEIIKK.C 751
752	522.3097	1042.6047	1042.6022	2.41	1	(46)	0.0085	1	U	K.QVADEIIKK.C

---

21. 2::A0A1L2CVK2 **Mass:** 19336 **Score:** 67 **Matches:** 3(3) **Sequences:** 1(1) **emPAI:** 0.24
>tr|A0A1L2CVK2|A0A1L2CVK2_9CAUD Uncharacterized protein OS=Pectobacterium phage CBB OX=1792242 GN=CBB_463 PE=4 SV=1

Query	Observed	Mr(expt)	Mr(calc)	ppm	Miss	Score	Expect	Rank	Unique	Peptide
31	365.2347	728.4548	728.4545	0.52	0	51	0.0066	1	U	R.TLQVLR.M 30 32

Proteins matching the same set of peptides:
2::K4F7S2 **Mass:** 19272 **Score:** 67 **Matches:** 3(3) **Sequences:** 1(1)
>tr|K4F7S2|K4F7S2_9CAUD Uncharacterized protein OS=Cronobacter phage vB_CsaM_GAP32 OX=1141136 GN=GAP32_442 PE=4 SV=1

---

22. 2::A0A0P0KQU0 **Mass:** 324585 **Score:** 66 **Matches:** 2(2) **Sequences:** 1(1) **emPAI:** 0.01
>tr|A0A0P0KQU0|A0A0P0KQU0_9HEPC Genome polyprotein (Fragment) OS=Hepatitis C virus subtype 1a OX=31646 PE=4 SV=1

Query	Observed	Mr(expt)	Mr(calc)	ppm	Miss	Score	Expect	Rank	Unique	Peptide
28	364.2080	726.4015	726.4024	-1.29	0	51	0.0015	1	U	R.GPELGVR.A 27

Proteins matching the same set of peptides:
2::A0A1B1LUZ0 **Mass:** 331111 **Score:** 66 **Matches:** 2(2) **Sequences:** 1(1)
>tr|A0A1B1LUZ0|A0A1B1LUZ0_9HEPC Genome polyprotein (Fragment) OS=Hepacivirus C OX=11103 PE=4 SV=1
2::A0A1B1LUZ4 **Mass:** 331170 **Score:** 66 **Matches:** 2(2) **Sequences:** 1(1)
>tr|A0A1B1LUZ4|A0A1B1LUZ4_9HEPC Genome polyprotein (Fragment) OS=Hepacivirus C OX=11103 PE=4 SV=1
2::A0A5B9BYM6 **Mass:** 12209 **Score:** 66 **Matches:** 2(2) **Sequences:** 1(1)
>tr|A0A5B9BYM6|A0A5B9BYM6_9HEPC Polyprotein (Fragment) OS=Hepacivirus C OX=11103 GN=POLY PE=4 SV=1
2::A0A5B9BZ95 **Mass:** 12140 **Score:** 66 **Matches:** 2(2) **Sequences:** 1(1)
>tr|A0A5B9BZ95|A0A5B9BZ95_9HEPC Polyprotein (Fragment) OS=Hepacivirus C OX=11103 GN=POLY PE=4 SV=1
2::A0A5B9BZV7 **Mass:** 12233 **Score:** 66 **Matches:** 2(2) **Sequences:** 1(1)
>tr|A0A5B9BZV7|A0A5B9BZV7_9HEPC Polyprotein (Fragment) OS=Hepacivirus C OX=11103 GN=POLY PE=4 SV=1
2::A0A5B9BZW6 **Mass:** 12281 **Score:** 66 **Matches:** 2(2) **Sequences:** 1(1)
>tr|A0A5B9BZW6|A0A5B9BZW6_9HEPC Polyprotein (Fragment) OS=Hepacivirus C OX=11103 GN=POLY PE=4 SV=1
2::L0B3F6 **Mass:** 12737 **Score:** 66 **Matches:** 2(2) **Sequences:** 1(1)
>tr|L0B3F6|L0B3F6_9HEPC Polyprotein (Fragment) OS=Hepacivirus C OX=11103 PE=4 SV=1
2::L0B510 **Mass:** 12713 **Score:** 66 **Matches:** 2(2) **Sequences:** 1(1)
>tr|L0B510|L0B510_9HEPC Polyprotein (Fragment) OS=Hepacivirus C OX=11103 PE=4 SV=1
2::L0B528 **Mass:** 12719 **Score:** 66 **Matches:** 2(2) **Sequences:** 1(1)
>tr|L0B528|L0B528_9HEPC Polyprotein (Fragment) OS=Hepacivirus C OX=11103 PE=4 SV=1
2::L0B5D2 **Mass:** 12755 **Score:** 66 **Matches:** 2(2) **Sequences:** 1(1)
>tr|L0B5D2|L0B5D2_9HEPC Polyprotein (Fragment) OS=Hepacivirus C OX=11103 PE=4 SV=1
2::Q0GRI0 **Mass:** 13394 **Score:** 66 **Matches:** 2(2) **Sequences:** 1(1)
>tr|Q0GRI0|Q0GRI0_9HEPC Core protein (Fragment) OS=Hepacivirus C OX=11103 PE=4 SV=1
2::Q3S7E1 **Mass:** 13504 **Score:** 66 **Matches:** 2(2) **Sequences:** 1(1)
>tr|Q3S7E1|Q3S7E1_9HEPC Core protein (Fragment) OS=Hepacivirus C OX=11103 PE=4 SV=2
2::F4YQA4 **Mass:** 20921 **Score:** 66 **Matches:** 2(2) **Sequences:** 1(1)
>tr|F4YQA4|F4YQA4_9HEPC Core protein (Fragment) OS=Hepacivirus C OX=11103 PE=4 SV=1
2::A5A7Y3 **Mass:** 10190 **Score:** 66 **Matches:** 2(2) **Sequences:** 1(1)
>tr|A5A7Y3|A5A7Y3_9HEPC Core protein (Fragment) OS=Hepatitis C virus subtype 6n OX=378506 PE=2 SV=1
2::A6YCA4 **Mass:** 75640 **Score:** 66 **Matches:** 2(2) **Sequences:** 1(1)
>tr|A6YCA4|A6YCA4_9HEPC Polyprotein (Fragment) OS=Hepacivirus C OX=11103 PE=4 SV=1
2::G9FVM8 **Mass:** 13698 **Score:** 66 **Matches:** 2(2) **Sequences:** 1(1)
>tr|G9FVM8|G9FVM8_9HEPC Core protein (Fragment) OS=Hepacivirus C OX=11103 PE=4 SV=1
2::H9B6W0 **Mass:** 20923 **Score:** 66 **Matches:** 2(2) **Sequences:** 1(1)
>tr|H9B6W0|H9B6W0_9HEPC Core protein (Fragment) OS=Hepacivirus C OX=11103 PE=4 SV=1
2::Q56NC8 **Mass:** 14561 **Score:** 66 **Matches:** 2(2) **Sequences:** 1(1)
>tr|Q56NC8|Q56NC8_9HEPC Core protein (Fragment) OS=Hepacivirus C OX=11103 GN=C PE=4 SV=1

---

23. 2::A0A1B1IVR3 **Mass:** 108030 **Score:** 66 **Matches:** 1(1) **Sequences:** 1(1) **emPAI:** 0.04
>tr|A0A1B1IVR3|A0A1B1IVR3_9VIRU Excinuclease ABC subunit A OS=uncultured Mediterranean phage OX=1868660 PE=3 SV=1

Query	Observed	Mr(expt)	Mr(calc)	ppm	Miss	Score	Expect	Rank	Unique	Peptide
2534	779.3786	1556.7427	1556.7471	-2.83	0	66	6.9e-05	1	U	K.SSLAFDTIYAEGQR.R

---

24. 2::A0A0A0Y6T0 **Mass:** 132333 **Score:** 63 **Matches:** 2(2) **Sequences:** 1(1) **emPAI:** 0.03
>tr|A0A0A0Y6T0|A0A0A0Y6T0_WSSVS Wsv216 OS=White spot syndrome virus (isolate Shrimp/China/Tongan/1996) OX=654913 PE=4 SV=1

Query	Observed	Mr(expt)	Mr(calc)	ppm	Miss	Score	Expect	Rank	Unique	Peptide
150	817.4325	816.4252	816.4229	2.84	0	58	0.00048	1	U	K.VDLVDEK.Y 149

Proteins matching the same set of peptides:
2::A0A0K2JNJ2 **Mass:** 132333 **Score:** 63 **Matches:** 2(2) **Sequences:** 1(1)
>tr|A0A0K2JNJ2|A0A0K2JNJ2_9VIRU Envelope protein OS=White spot syndrome virus OX=342409 PE=4 SV=1
2::A0A0S2E5V1 **Mass:** 132275 **Score:** 63 **Matches:** 2(2) **Sequences:** 1(1)
>tr|A0A0S2E5V1|A0A0S2E5V1_9VIRU Envelope protein OS=White spot syndrome virus OX=342409 PE=4 SV=1
2::A0A0S2E6C0 **Mass:** 132563 **Score:** 63 **Matches:** 2(2) **Sequences:** 1(1)
>tr|A0A0S2E6C0|A0A0S2E6C0_9VIRU Envelope protein OS=White spot syndrome virus OX=342409 PE=4 SV=1
2::A0A0X9LA84 **Mass:** 132299 **Score:** 63 **Matches:** 2(2) **Sequences:** 1(1)
>tr|A0A0X9LA84|A0A0X9LA84_9VIRU Uncharacterized protein OS=White spot syndrome virus OX=342409 PE=4 SV=1
2::A0A1Z2R9G2 **Mass:** 132448 **Score:** 63 **Matches:** 2(2) **Sequences:** 1(1)
>tr|A0A1Z2R9G2|A0A1Z2R9G2_9VIRU Wsv216 OS=White spot syndrome virus OX=342409 PE=4 SV=1
2::A0A2D3I4Q4 **Mass:** 133446 **Score:** 63 **Matches:** 2(2) **Sequences:** 1(1)
>tr|A0A2D3I4Q4|A0A2D3I4Q4_9VIRU ORF1077 OS=White spot syndrome virus OX=342409 PE=4 SV=1
2::A0A2I6SCF1 **Mass:** 17649 **Score:** 63 **Matches:** 2(2) **Sequences:** 1(1)
>tr|A0A2I6SCF1|A0A2I6SCF1_9VIRU WSSV484 OS=White spot syndrome virus OX=342409 PE=4 SV=1
2::A0A2R2XF94 **Mass:** 132373 **Score:** 63 **Matches:** 2(2) **Sequences:** 1(1)
>tr|A0A2R2XF94|A0A2R2XF94_9VIRU Envelope protein VP124 OS=White spot syndrome virus OX=342409 PE=4 SV=1
2::A0A2U9G8U6 **Mass:** 132218 **Score:** 63 **Matches:** 2(2) **Sequences:** 1(1)
>tr|A0A2U9G8U6|A0A2U9G8U6_WSSV Wsv216 OS=White spot syndrome virus OX=92652 GN=216 PE=4 SV=1
2::A0A2U9GE83 **Mass:** 132299 **Score:** 63 **Matches:** 2(2) **Sequences:** 1(1)
>tr|A0A2U9GE83|A0A2U9GE83_WSSV Wsv216 OS=White spot syndrome virus OX=92652 GN=216 PE=4 SV=1
2::A0A2U9GFK6 **Mass:** 132333 **Score:** 63 **Matches:** 2(2) **Sequences:** 1(1)
>tr|A0A2U9GFK6|A0A2U9GFK6_WSSV Wsv216 OS=White spot syndrome virus OX=92652 GN=216 PE=4 SV=1
2::A0A2U9GH22 **Mass:** 111991 **Score:** 63 **Matches:** 2(2) **Sequences:** 1(1)
>tr|A0A2U9GH22|A0A2U9GH22_WSSV Wsv216 OS=White spot syndrome virus OX=92652 GN=216 PE=4 SV=1
2::A0A5A4N7F0 **Mass:** 132505 **Score:** 63 **Matches:** 2(2) **Sequences:** 1(1)
>tr|A0A5A4N7F0|A0A5A4N7F0_9VIRU VP124 OS=Procambarus clarkii virus OX=2488332 GN=ORF81L PE=4 SV=1
2::K7WHI6 **Mass:** 132303 **Score:** 63 **Matches:** 2(2) **Sequences:** 1(1)
>tr|K7WHI6|K7WHI6_9VIRU Wsv216 OS=White spot syndrome virus OX=342409 GN=wssv_02160 PE=4 SV=1
2::Q8QTD8 **Mass:** 132337 **Score:** 63 **Matches:** 2(2) **Sequences:** 1(1)
>tr|Q8QTD8|Q8QTD8_WSSV WSSV271 OS=White spot syndrome virus OX=92652 PE=4 SV=1
2::Q91LD4 **Mass:** 132333 **Score:** 63 **Matches:** 2(2) **Sequences:** 1(1)
>tr|Q91LD4|Q91LD4_9VIRU ORF111 OS=White spot syndrome virus OX=342409 PE=4 SV=1

---

25. 2::A0A482MLD2 **Mass:** 11951 **Score:** 62 **Matches:** 1(1) **Sequences:** 1(1) **emPAI:** 0.41
>tr|A0A482MLD2|A0A482MLD2_9CAUD Uncharacterized protein OS=Caudovirales GX15bay OX=2530034 PE=4 SV=1

Query	Observed	Mr(expt)	Mr(calc)	ppm	Miss	Score	Expect	Rank	Unique	Peptide
145	408.7452	815.4758	815.4752	0.68	1	62	0.00031	1	U	R.ADEILKK.R

26. 2::G8XUC3 **Mass:** 242144 **Score:** 60 **Matches:** 7(3) **Sequences:** 1(1) **emPAI:** 0.02

>tr|G8XUC3|G8XUC3_9BETA Large tegument protein OS=Aotine betaherpesvirus 1 OX=50290 GN=UL48 PE=3 SV=1

Query	Observed	Mr(expt)	Mr(calc)	ppm	Miss	Score	Expect	Rank	Unique	Peptide
922	544.8018	1087.5890	1087.5873	1.54	1	51	0.0065	1	U	K.VEAVSKAAADK.N 914 915 916 920 923 924

27. 2::A0A3G4ZY70 **Mass:** 92750 **Score:** 60 **Matches:** 1(1) **Sequences:** 1(1) **emPAI:** 0.05

>tr|A0A3G4ZY70|A0A3G4ZY70_9VIRU Uncharacterized protein OS=Gaeavirus sp. OX=2487767 GN=Gaeavirus1_3 PE=4 SV=1

Query	Observed	Mr(expt)	Mr(calc)	ppm	Miss	Score	Expect	Rank	Unique	Peptide
527	486.2805	970.5464	970.5447	1.71	0	60	0.00045	1	U	R.ILNDDIIR.E

28. 2::A0A075Q9S7 **Mass:** 23916 **Score:** 59 **Matches:** 2(2) **Sequences:** 1(1) **emPAI:** 0.19

>tr|A0A075Q9S7|A0A075Q9S7_9HIV1 Protein Nef OS=Human immunodeficiency virus 1 OX=11676 GN=nef PE=2 SV=1

Query	Observed	Mr(expt)	Mr(calc)	ppm	Miss	Score	Expect	Rank	Unique	Peptide
162	412.7335	823.4525	823.4552	-3.32	0	46	0.0035	1	U	K.LVPQDPR.E 163

Proteins matching the same set of peptides:

2::A0A075QC14 **Mass:** 23905 **Score:** 59 **Matches:** 2(2) **Sequences:** 1(1)
>tr|A0A075QC14|A0A075QC14_9HIV1 Protein Nef OS=Human immunodeficiency virus 1 OX=11676 GN=nef PE=3 SV=1
2::A0A075QCX5 **Mass:** 23803 **Score:** 59 **Matches:** 2(2) **Sequences:** 1(1)
>tr|A0A075QCX5|A0A075QCX5_9HIV1 Protein Nef OS=Human immunodeficiency virus 1 OX=11676 GN=nef PE=3 SV=1
2::A0A075QEX6 **Mass:** 23901 **Score:** 59 **Matches:** 2(2) **Sequences:** 1(1)
>tr|A0A075QEX6|A0A075QEX6_9HIV1 Protein Nef OS=Human immunodeficiency virus 1 OX=11676 GN=nef PE=3 SV=1
2::A0A075QJB6 **Mass:** 23733 **Score:** 59 **Matches:** 2(2) **Sequences:** 1(1)
>tr|A0A075QJB6|A0A075QJB6_9HIV1 Protein Nef OS=Human immunodeficiency virus 1 OX=11676 GN=nef PE=2 SV=1
2::A0A0S3QMH6 **Mass:** 23860 **Score:** 59 **Matches:** 2(2) **Sequences:** 1(1)
>tr|A0A0S3QMH6|A0A0S3QMH6_9HIV1 Protein Nef OS=Human immunodeficiency virus 1 OX=11676 GN=nef PE=3 SV=1

29. 2::A0A0K2CZZ1 **Mass:** 46128 **Score:** 59 **Matches:** 1(1) **Sequences:** 1(1) **emPAI:** 0.10

>tr|A0A0K2CZZ1|A0A0K2CZZ1_9CAUD Putative phosphoadenosine phosphosulfate reductase OS=Bacillus phage TsarBomba OX=1690456 GN=TSARBOMBA_18 PE=4 SV=1

Query	Observed	Mr(expt)	Mr(calc)	ppm	Miss	Score	Expect	Rank	Unique	Peptide
29	365.2161	728.4177	728.4181	-0.48	0	59	0.002	1	U	K.ALAELGR.D

Proteins matching the same set of peptides:

2::A0A218KDB3 **Mass:** 46227 **Score:** 59 **Matches:** 1(1) **Sequences:** 1(1)
>tr|A0A218KDB3|A0A218KDB3_9CAUD Putative phosphoadenosine phosphosulfate reductase OS=Bacillus phage PBC6 OX=1673872 GN=PBC6_033 PE=4 SV=1
2::A0A2S0CSW2 **Mass:** 46227 **Score:** 59 **Matches:** 1(1) **Sequences:** 1(1)
>tr|A0A2S0CSW2|A0A2S0CSW2_9CAUD Putative phosphoadenosine phosphosulfate reductase OS=Bacillus phage BCP12 OX=1913122 GN=BCP12_239 PE=4 SV=1
2::A0A346TNJ5 **Mass:** 46227 **Score:** 59 **Matches:** 1(1) **Sequences:** 1(1)
>tr|A0A346TNJ5|A0A346TNJ5_9CAUD Putative phosphoadenosine phosphosulfate reductase OS=Bacillus phage BC01 OX=2039884 GN=BC01_038 PE=4 SV=1
2::A0A516M412 **Mass:** 35320 **Score:** 59 **Matches:** 1(1) **Sequences:** 1(1)
>tr|A0A516M412|A0A516M412_9VIRU Putative scaffolding protein OS=Prokaryotic dsDNA virus sp. OX=2591644 GN=Tp185DCM00d2C31949971_37 PE=4 SV=1
2::A0A516M6I5 **Mass:** 35348 **Score:** 59 **Matches:** 1(1) **Sequences:** 1(1)
>tr|A0A516M6I5|A0A516M6I5_9VIRU Putative scaffolding protein OS=Prokaryotic dsDNA virus sp. OX=2591644 GN=Tp1111MES1053591_48 PE=4 SV=1
2::A0A5B9NH97 **Mass:** 46241 **Score:** 59 **Matches:** 1(1) **Sequences:** 1(1)
>tr|A0A5B9NH97|A0A5B9NH97_9CAUD Putative phosphoadenosine phosphosulfate reductase OS=Bacillus phage vB_BspM_MarvelLand OX=2591380 GN=MARVELLAND_54 PE=4 SV=1
2::G9J263 **Mass:** 12881 **Score:** 59 **Matches:** 1(1) **Sequences:** 1(1)
>tr|G9J263|G9J263_9CAUD PAPS_reduct domain-containing protein OS=Bacillus phage BCP78 OX=1126950 GN=BCP78_0036 PE=4 SV=1
2::J9PRK2 **Mass:** 40917 **Score:** 59 **Matches:** 1(1) **Sequences:** 1(1)
>tr|J9PRK2|J9PRK2_9CAUD Putative phosphoadenosine phosphosulfate reductase OS=Bacillus phage BCU4 OX=1126951 GN=BCU4_0028 PE=4 SV=1

30. 2::A0A223W084 **Mass:** 26567 **Score:** 57 **Matches:** 1(1) **Sequences:** 1(1) **emPAI:** 0.17

>tr|A0A223W084|A0A223W084_9CAUD Uncharacterized protein OS=Agrobacterium phage Atu_ph08 OX=2024265 PE=4 SV=1

Query	Observed	Mr(expt)	Mr(calc)	ppm	Miss	Score	Expect	Rank	Unique	Peptide
144	408.7451	815.4756	815.4752	0.39	0	57	0.001	1	U	K.IVAEGLSK.L

31. 2::A0A516LXB0 **Mass:** 188227 **Score:** 56 **Matches:** 2(2) **Sequences:** 1(1) **emPAI:** 0.02

>tr|A0A516LXB0|A0A516LXB0_9VIRU Uncharacterized protein OS=Prokaryotic dsDNA virus sp. OX=2591644 GN=Unbinned175contig1000_19 PE=4 SV=1

Query	Observed	Mr(expt)	Mr(calc)	ppm	Miss	Score	Expect	Rank	Unique	Peptide
415	464.2078	926.4010	926.4021	-1.19	0	41	0.013	1	U	K.FDPEEYK.G 414

32. 2::A0A1B1IS87 **Mass:** 17656 **Score:** 56 **Matches:** 1(1) **Sequences:** 1(1) **emPAI:** 0.27

>tr|A0A1B1IS87|A0A1B1IS87_9VIRU Uncharacterized protein OS=uncultured Mediterranean phage OX=1868660 PE=4 SV=1

Query	Observed	Mr(expt)	Mr(calc)	ppm	Miss	Score	Expect	Rank	Unique	Peptide
518	483.7797	965.5448	965.5433	1.49	0	56	0.00028	1	U	K.YITIELSK.S

33. 2::A0A1D8MBQ4 **Mass:** 76569 **Score:** 55 **Matches:** 1(1) **Sequences:** 1(1) **emPAI:** 0.06

>tr|A0A1D8MBQ4|A0A1D8MBQ4_9VIRU Polyprotein (Fragment) OS=Endornavirus-like virus OX=1906952 PE=4 SV=1

Query	Observed	Mr(expt)	Mr(calc)	ppm	Miss	Score	Expect	Rank	Unique	Peptide
168	413.2759	824.5373	824.5371	0.21	0	55	4.1e-05	1	U	K.LIEIPLK.Q

34. 2::H2BCQ0 **Mass:** 7052 **Score:** 54 **Matches:** 2(1) **Sequences:** 1(1) **emPAI:** 0.76

>tr|H2BCQ0|H2BCQ0_9CAUD Uncharacterized protein OS=Synechococcus phage S-CBS4 OX=756275 GN=S-CBS4_gp009 PE=4 SV=1

Query	Observed	Mr(expt)	Mr(calc)	ppm	Miss	Score	Expect	Rank	Unique	Peptide
557	491.7578	981.5011	981.5018	-0.77	0	47	0.0095	1	U	M.ETLEISYK.E 558

35. 2::Q75491 **Mass:** 13951 **Score:** 54 **Matches:** 1(1) **Sequences:** 1(1) **emPAI:** 0.34

>tr|Q75491|Q75491_9HIV1 Envelope glycoprotein (Fragment) OS=Human immunodeficiency virus 1 OX=11676 GN=env PE=4 SV=1

Query	Observed	Mr(expt)	Mr(calc)	ppm	Miss	Score	Expect	Rank	Unique	Peptide
24	362.7086	723.4027	723.4028	-0.02	0	54	0.00041	1	U	K.IADHLR.E

36. 2::A0A068EKL1 **Mass:** 46526 **Score:** 54 **Matches:** 1(1) **Sequences:** 1(1) **emPAI:** 0.09

>tr|A0A068EKL1|A0A068EKL1_9POXV Ankyrin repeat protein OS=Pigeonpox virus OX=10264 GN=fep_016 PE=4 SV=1

Query	Observed	Mr(expt)	Mr(calc)	ppm	Miss	Score	Expect	Rank	Unique	Peptide
55	376.7125	751.4105	751.4116	-1.37	0	54	0.0012	1	U	K.LSYEIK.Y

Proteins matching the same set of peptides:

2::A0A068EL77 **Mass:** 46093 **Score:** 54 **Matches:** 1(1) **Sequences:** 1(1)
>tr|A0A068EL77|A0A068EL77_9POXV Ankyrin repeat protein OS=Penguinpox virus OX=648998 GN=pepv_016 PE=4 SV=1
2::A0A1X9Q118 **Mass:** 56950 **Score:** 54 **Matches:** 1(1) **Sequences:** 1(1)
>tr|A0A1X9Q118|A0A1X9Q118_9VIRU Putative VP4 OS=Human gut microviridae SH-CHD8 OX=1986033 PE=4 SV=1

37. 2::A0A0A0QBC3 **Mass:** 23537 **Score:** 54 **Matches:** 2(2) **Sequences:** 1(1) **emPAI:** 0.19

>tr|A0A0A0QBC3|A0A0A0QBC3_9CIRC Replication-associated protein (Fragment) OS=Circovirus VS6600032 OX=1525174 PE=4 SV=1

Query	Observed	Mr(expt)	Mr(calc)	ppm	Miss	Score	Expect	Rank	Unique	Peptide
37	365.7259	729.4372	729.4385	-1.74	0	48	0.014	1	U	R.TLDIIR.R 36

38. 2::A0A1V0SKV6 **Mass:** 61089 **Score:** 54 **Matches:** 1(1) **Sequences:** 1(1) **emPAI:** 0.07

>tr|A0A1V0SKV6|A0A1V0SKV6_9VIRU Uncharacterized protein OS=Klosneuvirus KNV1 OX=1977640 GN=Klosneuvirus_5_30 PE=4 SV=1

Query	Observed	Mr(expt)	Mr(calc)	ppm	Miss	Score	Expect	Rank	Unique	Peptide
16	360.7186	719.4227	719.4218	1.31	0	54	0.00068	1	U	R.IPYSLK.Y

Proteins matching the same set of peptides:

2::A0A385I731 **Mass:** 203583 **Score:** 54 **Matches:** 1(1) **Sequences:** 1(1)
>tr|A0A385I731|A0A385I731_9RETR Glycoprotein OS=Saesbyeol virus OX=2320522 PE=4 SV=1
2::A0A5J6VJE5 **Mass:** 45156 **Score:** 54 **Matches:** 1(1) **Sequences:** 1(1)
>tr|A0A5J6VJE5|A0A5J6VJE5_9VIRU Elongation factor Tu GTP binding domain protein OS=Megaviridae environmental sample OX=1737588 PE=4 SV=1

---

39. 2::A0A160DAA3 **Mass:** 154217 **Score:** 54 **Matches:** 1(1) **Sequences:** 1(1) **emPAI:** 0.03
>tr|A0A160DAA3|A0A160DAA3_9ALPC Spike glycoprotein OS=Porcine epidemic diarrhea virus OX=28295 GN=S PE=2 SV=1

Query	Observed	Mr(expt)	Mr(calc)	ppm	Miss	Score	Expect	Rank	Unique	Peptide
35	365.7138	729.4131	729.4133	-0.28	0	54	0.0041	1	U	K.GLNTVAR.A

Proteins matching the same set of peptides:

2::A0A165XVM7 **Mass:** 154063 **Score:** 54 **Matches:** 1(1) **Sequences:** 1(1)
>tr|A0A165XVM7|A0A165XVM7_9ALPC Spike glycoprotein OS=Porcine epidemic diarrhea virus OX=28295 GN=S PE=3 SV=1
2::A0A165XVS3 **Mass:** 154091 **Score:** 54 **Matches:** 1(1) **Sequences:** 1(1)
>tr|A0A165XVS3|A0A165XVS3_9ALPC Spike glycoprotein OS=Porcine epidemic diarrhea virus OX=28295 GN=S PE=3 SV=1
2::A0A165XVY6 **Mass:** 154074 **Score:** 54 **Matches:** 1(1) **Sequences:** 1(1)
>tr|A0A165XVY6|A0A165XVY6_9ALPC Spike glycoprotein OS=Porcine epidemic diarrhea virus OX=28295 GN=S PE=3 SV=1
2::A0A172PC26 **Mass:** 154093 **Score:** 54 **Matches:** 1(1) **Sequences:** 1(1)
>tr|A0A172PC26|A0A172PC26_9ALPC Spike glycoprotein OS=Porcine epidemic diarrhea virus OX=28295 GN=S PE=3 SV=1
2::A0A2H5AC33 **Mass:** 154491 **Score:** 54 **Matches:** 1(1) **Sequences:** 1(1)
>tr|A0A2H5AC33|A0A2H5AC33_9ALPC Spike glycoprotein OS=Porcine epidemic diarrhea virus OX=28295 GN=S PE=3 SV=1
2::A0A2H5AC45 **Mass:** 154543 **Score:** 54 **Matches:** 1(1) **Sequences:** 1(1)
>tr|A0A2H5AC45|A0A2H5AC45_9ALPC Spike glycoprotein OS=Porcine epidemic diarrhea virus OX=28295 GN=S PE=3 SV=1
2::A0A2R4H2R8 **Mass:** 154533 **Score:** 54 **Matches:** 1(1) **Sequences:** 1(1)
>tr|A0A2R4H2R8|A0A2R4H2R8_9ALPC Spike glycoprotein OS=Porcine epidemic diarrhea virus OX=28295 GN=S PE=3 SV=1

---

40. 2::H6WG71 **Mass:** 6463 **Score:** 53 **Matches:** 1(1) **Sequences:** 1(1) **emPAI:** 0.86
>tr|H6WG71|H6WG71_9CAUD Uncharacterized protein OS=Cyanophage S-TIM5 OX=1137745 PE=4 SV=1

Query	Observed	Mr(expt)	Mr(calc)	ppm	Miss	Score	Expect	Rank	Unique	Peptide
57	376.7248	751.4351	751.4341	1.40	0	53	0.00085	1	U	K.IDVLHR.V

---

41. 1::C1JCT2 **Mass:** 392767 **Score:** 53 **Matches:** 1(1) **Sequences:** 1(1) **emPAI:** 0.01
Polyprotein-FSD OS=Solenopsis invicta virus 3 OX=631345 PE=1 SV=2

Query	Observed	Mr(expt)	Mr(calc)	ppm	Miss	Score	Expect	Rank	Unique	Peptide
1027	557.8164	1113.6182	1113.6142	3.61	0	53	0.0031	1	U	K.VIQTGNNLAGK.H

---

42. 2::A0A401IPN0 **Mass:** 255005 **Score:** 51 **Matches:** 2(2) **Sequences:** 1(1) **emPAI:** 0.02
>tr|A0A401IPN0|A0A401IPN0_9VIRU Wsv514-like protein OS=Sesarmops intermedium nimavirus OX=2133796 PE=4 SV=1

Query	Observed	Mr(expt)	Mr(calc)	ppm	Miss	Score	Expect	Rank	Unique	Peptide
129	406.2179	810.4212	810.4236	-2.92	0	45	0.0048	1	U	K.IGEHIDK.F 130

---

43. 2::Q6RU22 **Mass:** 17943 **Score:** 51 **Matches:** 1(1) **Sequences:** 1(1) **emPAI:** 0.26
>tr|Q6RU22|Q6RU22_9HIV1 Gag protein (Fragment) OS=Human immunodeficiency virus 1 OX=11676 GN=gag PE=4 SV=1

Query	Observed	Mr(expt)	Mr(calc)	ppm	Miss	Score	Expect	Rank	Unique	Peptide
590	496.7639	991.5133	991.5127	0.60	0	51	0.0055	1	U	-.DFLNAWVK.V

---

44. 2::A0A2D2W767 **Mass:** 12740 **Score:** 51 **Matches:** 1(1) **Sequences:** 1(1) **emPAI:** 0.38
>tr|A0A2D2W767|A0A2D2W767_9CAUD Uncharacterized protein OS=Pectobacterium phage DU_PP_V OX=2041492 GN=P13BB106kb_p046 PE=4 SV=1

Query	Observed	Mr(expt)	Mr(calc)	ppm	Miss	Score	Expect	Rank	Unique	Peptide
174	414.7514	827.4882	827.4865	2.14	0	51	0.0076	1	U	K.IANLELR.A

---

45. 2::A0A482MM07 **Mass:** 31543 **Score:** 51 **Matches:** 2(0) **Sequences:** 1(0)
>tr|A0A482MM07|A0A482MM07_9CAUD Uncharacterized protein OS=Burkholderia phage BcepSauron OX=2530033 GN=BcepSauron_325 PE=4 SV=1

Query	Observed	Mr(expt)	Mr(calc)	ppm	Miss	Score	Expect	Rank	Unique	Peptide
279	437.7171	873.4197	873.4192	0.59	0	42	0.17	1	U	K.VGELGDER.S 278

---

46. 2::A0A4D6NVF7 **Mass:** 44635 **Score:** 50 **Matches:** 1(1) **Sequences:** 1(1) **emPAI:** 0.10
>tr|A0A4D6NVF7|A0A4D6NVF7_9VIRU Structural protein (Fragment) OS=Melipona quadrifasciata virus 1b OX=2571177 GN=SP PE=4 SV=1

Query	Observed	Mr(expt)	Mr(calc)	ppm	Miss	Score	Expect	Rank	Unique	Peptide
259	435.7561	869.4977	869.4971	0.75	0	50	0.0014	1	U	R.IATVPNQK.H

---

47. 2::A0A023VZE8 **Mass:** 58864 **Score:** 50 **Matches:** 2(2) **Sequences:** 1(1) **emPAI:** 0.07
>tr|A0A023VZE8|A0A023VZE8_BSMV Beta-B protein OS=Barley stripe mosaic virus OX=12327 PE=4 SV=1

Query	Observed	Mr(expt)	Mr(calc)	ppm	Miss	Score	Expect	Rank	Unique	Peptide
121	405.2239	808.4332	808.4330	0.16	0	46	0.008	1	U	R.LASLYDK.H 122

Proteins matching the same set of peptides:

2::A0A0G2RAA1 **Mass:** 56933 **Score:** 50 **Matches:** 2(2) **Sequences:** 1(1)
>tr|A0A0G2RAA1|A0A0G2RAA1_BSMV Beta b protein OS=Barley stripe mosaic virus OX=12327 PE=4 SV=1
2::A0A2K9YRQ0 **Mass:** 57069 **Score:** 50 **Matches:** 2(2) **Sequences:** 1(1)
>tr|A0A2K9YRQ0|A0A2K9YRQ0_BSMV Beta b protein OS=Barley stripe mosaic virus OX=12327 PE=4 SV=1
2::A0A2K9YS21 **Mass:** 56872 **Score:** 50 **Matches:** 2(2) **Sequences:** 1(1)
>tr|A0A2K9YS21|A0A2K9YS21_BSMV Beta b protein OS=Barley stripe mosaic virus OX=12327 PE=4 SV=1
2::A0A2K9YS53 **Mass:** 57104 **Score:** 50 **Matches:** 2(2) **Sequences:** 1(1)
>tr|A0A2K9YS53|A0A2K9YS53_BSMV Beta b protein OS=Barley stripe mosaic virus OX=12327 PE=4 SV=1
2::A0A2K9YS64 **Mass:** 57097 **Score:** 50 **Matches:** 2(2) **Sequences:** 1(1)
>tr|A0A2K9YS64|A0A2K9YS64_BSMV Beta b protein OS=Barley stripe mosaic virus OX=12327 PE=4 SV=1
2::Q5Q1D8 **Mass:** 57255 **Score:** 50 **Matches:** 2(2) **Sequences:** 1(1)
>tr|Q5Q1D8|Q5Q1D8_BSMV Beta-B protein OS=Barley stripe mosaic virus OX=12327 PE=2 SV=1
1::P04867 **Mass:** 58458 **Score:** 50 **Matches:** 2(2) **Sequences:** 1(1)
Movement protein TGB1 OS=Barley stripe mosaic virus OX=12327 PE=1 SV=1
2::Q65722 **Mass:** 56975 **Score:** 50 **Matches:** 2(2) **Sequences:** 1(1)
>tr|Q65722|Q65722_BSMV Beta-b protein OS=Barley stripe mosaic virus OX=12327 PE=2 SV=1
2::Q65723 **Mass:** 56931 **Score:** 50 **Matches:** 2(2) **Sequences:** 1(1)
>tr|Q65723|Q65723_BSMV Beta-b protein OS=Barley stripe mosaic virus OX=12327 PE=2 SV=1
2::Q65725 **Mass:** 57005 **Score:** 50 **Matches:** 2(2) **Sequences:** 1(1)
>tr|Q65725|Q65725_BSMV Beta-b protein OS=Barley stripe mosaic virus OX=12327 PE=2 SV=1

---

48. 2::A0A0M3SUP6 **Mass:** 49172 **Score:** 50 **Matches:** 1(1) **Sequences:** 1(1) **emPAI:** 0.09
>tr|A0A0M3SUP6|A0A0M3SUP6_9VIRU RNA-dependent RNA polymerase (Fragment) OS=Rhizoctonia solani beny-like virus 1 OX=1708385 PE=4 SV=1

Query	Observed	Mr(expt)	Mr(calc)	ppm	Miss	Score	Expect	Rank	Unique	Peptide
10	358.7122	715.4098	715.4116	-2.52	0	50	0.0073	1	U	R.IVEEVK.V

Proteins matching the same set of peptides:

2::A0A127AW26 **Mass:** 20044 **Score:** 50 **Matches:** 1(1) **Sequences:** 1(1)
>tr|A0A127AW26|A0A127AW26_9CAUD Uncharacterized protein OS=Bacillus phage SP-15 OX=1792032 GN=SP15_039 PE=4 SV=1
2::A0A1L3KLR4 **Mass:** 49735 **Score:** 50 **Matches:** 1(1) **Sequences:** 1(1)
>tr|A0A1L3KLR4|A0A1L3KLR4_9VIRU RdRp OS=Wenling partiti-like virus 13 OX=1923519 PE=4 SV=1
2::A0A3G5AHH2 **Mass:** 53785 **Score:** 50 **Matches:** 1(1) **Sequences:** 1(1)
>tr|A0A3G5AHH2|A0A3G5AHH2_9VIRU Uncharacterized protein OS=Sylvanvirus sp. OX=2487774 GN=Sylvanvirus4_2 PE=4 SV=1
2::A0MNA8 **Mass:** 17169 **Score:** 50 **Matches:** 1(1) **Sequences:** 1(1)
>tr|A0MNA8|A0MNA8_9CAUD Uncharacterized protein OS=Thermus phage phiYS40 OX=407392 GN=YS40_090 PE=4 SV=1
2::G0T5E5 **Mass:** 52997 **Score:** 50 **Matches:** 1(1) **Sequences:** 1(1)
>tr|G0T5E5|G0T5E5_IRV9 Uncharacterized protein OS=Wiseana iridescent virus OX=68347 PE=4 SV=1
2::J9PV61 **Mass:** 25120 **Score:** 50 **Matches:** 1(1) **Sequences:** 1(1)
>tr|J9PV61|J9PV61_9CAUD Putative radical activating enzyme OS=Bacillus phage BCD7 OX=1136534 GN=BCD7_0027 PE=3 SV=1

---

49. 2::A0A482JV29 **Mass:** 224784 **Score:** 49 **Matches:** 1(1) **Sequences:** 1(1) **emPAI:** 0.02
>tr|A0A482JV29|A0A482JV29_9RHAB RNA-directed RNA polymerase L OS=Lampyris noctiluca rhabdo-like virus 1 OX=2552994 PE=3 SV=1

Query	Observed	Mr(expt)	Mr(calc)	ppm	Miss	Score	Expect	Rank	Unique	Peptide
308	445.7223	889.4301	889.4294	0.86	0	49	0.0035	1	U	K.EWIATDR.A

---

50. 2::A0A023NH97 **Mass:** 88819 **Score:** 49 **Matches:** 1(1) **Sequences:** 1(1) **emPAI:** 0.05
>tr|A0A023NH97|A0A023NH97_9CAUD Putative ribonucleoside-diphosphate redutase OS=Dinoroseobacter phage DFL12phi1 OX=1477404 GN=DFL12P1_0034 PE=4 SV=1

Query	Observed	Mr(expt)	Mr(calc)	ppm	Miss	Score	Expect	Rank	Unique	Peptide
405	461.2380	920.4615	920.4603	1.23	0	49	0.0062	1	U	K.WVDSSISK.T

Proteins matching the same set of peptides:

2::A0A0A7CHE9 **Mass:** 88820 **Score:** 49 **Matches:** 1(1) **Sequences:** 1(1)
>tr|A0A0A7CHE9|A0A0A7CHE9_9CAUD Putative ribonucleoside-diphosphate redutase OS=Dinoroseobacter phage vBDshPR2C OX=1498169 GN=vBDshPR2C_50 PE=4 SV=1
2::A0A2Z4QFE2 **Mass:** 88352 **Score:** 49 **Matches:** 1(1) **Sequences:** 1(1)
>tr|A0A2Z4QFE2|A0A2Z4QFE2_9CAUD Putative ribonucleoside-diphosphate reductase OS=Ruegeria phage vB_RpoP-V12 OX=2218611 GN=vBRpoPV12_50 PE=4 SV=1
2::A0A2Z4QFG0 **Mass:** 88352 **Score:** 49 **Matches:** 1(1) **Sequences:** 1(1)
>tr|A0A2Z4QFG0|A0A2Z4QFG0_9CAUD Putative ribonucleoside-diphosphate reductase OS=Ruegeria phage vB_RpoP-V21 OX=2218615 GN=vBRpoPV21_47 PE=4 SV=1
2::A0A2Z4QHC9 **Mass:** 89077 **Score:** 49 **Matches:** 1(1) **Sequences:** 1(1)
>tr|A0A2Z4QHC9|A0A2Z4QHC9_9CAUD Ribonucleotide reductase of class II OS=Ruegeria phage vB_RpoP-V13 OX=2218612 GN=vBRpoPV13_42 PE=4 SV=1
2::A0A2Z4QIG9 **Mass:** 88352 **Score:** 49 **Matches:** 1(1) **Sequences:** 1(1)
>tr|A0A2Z4QIG9|A0A2Z4QIG9_9CAUD Putative ribonucleoside-diphosphate reductase OS=Ruegeria phage vB_RpoP-V17 OX=2218614 GN=vBRpoPV17_47 PE=4 SV=1
2::A0A345AYJ0 **Mass:** 88352 **Score:** 49 **Matches:** 1(1) **Sequences:** 1(1)
>tr|A0A345AYJ0|A0A345AYJ0_9CAUD Ribonucleotide reductase of class II OS=Ruegeria phage vB_RpoP-V14 OX=2218613 GN=vBRpoPV14_52 PE=4 SV=1
2::W0NY41 **Mass:** 89169 **Score:** 49 **Matches:** 1(1) **Sequences:** 1(1)
>tr|W0NY41|W0NY41_9CAUD Ribonucleoside-diphosphate reductase OS=Sulfitobacter phage phiCB2047-B OX=754046 GN=SUFG_00031 PE=4 SV=1
2::E3PZ93 **Mass:** 88256 **Score:** 49 **Matches:** 1(1) **Sequences:** 1(1)
>tr|E3PZ93|E3PZ93_9CAUD Hypothetical host-like ribonucleoside diphosphate reductase OS=Roseovarius sp. 217 phage 1 OX=874471 PE=4 SV=1
2::C4NT50 **Mass:** 88352 **Score:** 49 **Matches:** 1(1) **Sequences:** 1(1)
>tr|C4NT50|C4NT50_9CAUD Putative ribonucleoside-diphosphate redutase OS=Silicibacter phage DSS3phi2 OX=490912 PE=4 SV=1
2::C4NTC8 **Mass:** 88160 **Score:** 49 **Matches:** 1(1) **Sequences:** 1(1)
>tr|C4NTC8|C4NTC8_9CAUD Putative ribonucleoside-diphosphate redutase OS=Sulfitobacter phage EE36phi1 OX=490913 PE=4 SV=1
2::K4Q4Y8 **Mass:** 88171 **Score:** 49 **Matches:** 1(1) **Sequences:** 1(1)
>tr|K4Q4Y8|K4Q4Y8_9CAUD Hypothetical host-like protein OS=Roseovarius Plymouth podovirus 1 OX=926474 PE=4 SV=1

---

51. 2::A0A1E1GE69 **Mass:** 88638 **Score:** 49 **Matches:** 1(1) **Sequences:** 1(1) **emPAI:** 0.05
>tr|A0A1E1GE69|A0A1E1GE69_9CAUD Putative primase OS=Vibrio phage CKB-S1 OX=1903259 PE=4 SV=1

Query	Observed	Mr(expt)	Mr(calc)	ppm	Miss	Score	Expect	Rank	Unique	Peptide
576	494.2771	986.5396	986.5397	-0.04	0	49	0.0054	1	U	R.AAVSAGVGDLK.G

---

52. 2::A0A1W6RAJ7 **Mass:** 12732 **Score:** 49 **Matches:** 1(1) **Sequences:** 1(1) **emPAI:** 0.38
>tr|A0A1W6RAJ7|A0A1W6RAJ7_9ENTO Capsid protein VP1 (Fragment) OS=Echovirus E16 OX=47504 PE=4 SV=1

Query	Observed	Mr(expt)	Mr(calc)	ppm	Miss	Score	Expect	Rank	Unique	Peptide
262	435.7735	869.5324	869.5334	-1.13	0	49	0.01	1	U	K.ELVQLIR.K

Proteins matching the same set of peptides:

2::A0A1W6RAM0 **Mass:** 12750 **Score:** 49 **Matches:** 1(1) **Sequences:** 1(1)
>tr|A0A1W6RAM0|A0A1W6RAM0_9ENTO Capsid protein VP1 (Fragment) OS=Echovirus E16 OX=47504 PE=4 SV=1
2::A0A1W6RB04 **Mass:** 12732 **Score:** 49 **Matches:** 1(1) **Sequences:** 1(1)
>tr|A0A1W6RB04|A0A1W6RB04_9ENTO Capsid protein VP1 (Fragment) OS=Echovirus E16 OX=47504 PE=4 SV=1
2::A0A1W6RB18 **Mass:** 12750 **Score:** 49 **Matches:** 1(1) **Sequences:** 1(1)
>tr|A0A1W6RB18|A0A1W6RB18_9ENTO Capsid protein VP1 (Fragment) OS=Echovirus E16 OX=47504 PE=4 SV=1
2::A0A1W6RBG6 **Mass:** 11272 **Score:** 49 **Matches:** 1(1) **Sequences:** 1(1)
>tr|A0A1W6RBG6|A0A1W6RBG6_9ENTO Capsid protein VP1 (Fragment) OS=Echovirus E16 OX=47504 PE=4 SV=1

---

53. 2::A0A1W6JEN0 **Mass:** 48576 **Score:** 48 **Matches:** 1(1) **Sequences:** 1(1) **emPAI:** 0.09
>tr|A0A1W6JEN0|A0A1W6JEN0_9BETA Uncharacterized protein OS=Human betaherpesvirus 6 OX=10368 PE=4 SV=1

Query	Observed	Mr(expt)	Mr(calc)	ppm	Miss	Score	Expect	Rank	Unique	Peptide
537	487.2702	972.5259	972.5240	1.98	0	48	0.008	1	U	R.IESLENLR.N

---

54. 2::A0A097BY06 **Mass:** 77954 **Score:** 48 **Matches:** 1(1) **Sequences:** 1(1) **emPAI:** 0.06
>tr|A0A097BY06|A0A097BY06_9CAUD Minor tail protein OS=Mycobacterium phage Power OX=1552733 GN=PBI_POWER_31 PE=4 SV=1

Query	Observed	Mr(expt)	Mr(calc)	ppm	Miss	Score	Expect	Rank	Unique	Peptide
76	385.2446	768.4746	768.4745	0.10	0	48	0.00096	1	U	R.LPLDALK.T

Proteins matching the same set of peptides:

2::A0A097EWN0 **Mass:** 78208 **Score:** 48 **Matches:** 1(1) **Sequences:** 1(1)
>tr|A0A097EWN0|A0A097EWN0_9CAUD Minor tail protein OS=Mycobacterium phage SweetiePie OX=1555235 GN=31 PE=4 SV=1
2::A0A0M3UL68 **Mass:** 84250 **Score:** 48 **Matches:** 1(1) **Sequences:** 1(1)
>tr|A0A0M3UL68|A0A0M3UL68_9CAUD Minor tail protein OS=Mycobacterium phage LadyBird OX=1718166 GN=SEA_LADYBIRD_31 PE=4 SV=1
2::A0A1C9LZW9 **Mass:** 77898 **Score:** 48 **Matches:** 1(1) **Sequences:** 1(1)
>tr|A0A1C9LZW9|A0A1C9LZW9_9CAUD Minor tail protein OS=Mycobacterium phage DudeLittle OX=1897766 GN=SEA_DUDELITTLE_31 PE=4 SV=1
2::A0A220NSE9 **Mass:** 63313 **Score:** 48 **Matches:** 1(1) **Sequences:** 1(1)
>tr|A0A220NSE9|A0A220NSE9_9CAUD Minor tail protein OS=Mycobacterium phage Heffalump OX=1983575 GN=33 PE=4 SV=1
2::A0A2D1G5J0 **Mass:** 77933 **Score:** 48 **Matches:** 1(1) **Sequences:** 1(1)
>tr|A0A2D1G5J0|A0A2D1G5J0_9CAUD Minor tail protein OS=Mycobacterium phage Anselm OX=2041517 GN=SEA_ANSELM_31 PE=4 SV=1
2::A0A2D1G9Y3 **Mass:** 78257 **Score:** 48 **Matches:** 1(1) **Sequences:** 1(1)
>tr|A0A2D1G9Y3|A0A2D1G9Y3_9CAUD Minor tail protein OS=Mycobacterium phage Dalmatian OX=2041527 GN=SEA_DALMATIAN_31 PE=4 SV=1
2::A0A2D1GJ93 **Mass:** 78238 **Score:** 48 **Matches:** 1(1) **Sequences:** 1(1)
>tr|A0A2D1GJ93|A0A2D1GJ93_9CAUD Minor tail protein OS=Mycobacterium phage SnapTap OX=2041549 GN=SEA_SNAPTAP_31 PE=4 SV=1
2::A0A2R4AP72 **Mass:** 78230 **Score:** 48 **Matches:** 1(1) **Sequences:** 1(1)
>tr|A0A2R4AP72|A0A2R4AP72_9CAUD Minor tail protein OS=Mycobacterium phage Fameo OX=2126943 GN=31 PE=4 SV=1
2::A0A2Z5H0E5 **Mass:** 78208 **Score:** 48 **Matches:** 1(1) **Sequences:** 1(1)
>tr|A0A2Z5H0E5|A0A2Z5H0E5_9CAUD Minor tail protein OS=Mycobacterium phage Crucio OX=2234072 GN=31 PE=4 SV=1
2::A0A385D5H4 **Mass:** 77889 **Score:** 48 **Matches:** 1(1) **Sequences:** 1(1)
>tr|A0A385D5H4|A0A385D5H4_9CAUD Minor tail protein OS=Mycobacterium phage QueenBeesly OX=2301544 GN=31 PE=4 SV=1
2::A0A5J6TLJ6 **Mass:** 78367 **Score:** 48 **Matches:** 1(1) **Sequences:** 1(1)
>tr|A0A5J6TLJ6|A0A5J6TLJ6_9CAUD Minor tail protein OS=Mycobacterium phage SemperFi OX=2599879 GN=33 PE=4 SV=1
2::M4HZ75 **Mass:** 84118 **Score:** 48 **Matches:** 1(1) **Sequences:** 1(1)
>tr|M4HZ75|M4HZ75_9CAUD Minor tail subunit OS=Mycobacterium phage First OX=1245814 GN=First_0031 PE=4 SV=1
2::W8E958 **Mass:** 84119 **Score:** 48 **Matches:** 1(1) **Sequences:** 1(1)
>tr|W8E958|W8E958_9CAUD Minor tail subunit OS=Mycobacterium phage 20ES OX=1458726 GN=20ES_31 PE=4 SV=1

---

55. 2::D7SGS7 **Mass:** 46496 **Score:** 48 **Matches:** 1(1) **Sequences:** 1(1) **emPAI:** 0.09
>tr|D7SGS7|D7SGS7_9MONO Polymerase (Fragment) OS=Paramyxovirus bat/GH47/2008 OX=589524 GN=L PE=4 SV=1

Query	Observed	Mr(expt)	Mr(calc)	ppm	Miss	Score	Expect	Rank	Unique	Peptide
715	516.3036	1030.5927	1030.5910	1.62	0	48	0.0068	1	U	K.VIDLETTLK.T

Proteins matching the same set of peptides:
2::K7XAQ9 **Mass:** 259901 **Score:** 48 **Matches:** 1(1) **Sequences:** 1(1)
>tr|K7XAQ9|K7XAQ9_9MONO RNA-directed RNA polymerase L OS=Achimota virus 1 OX=1261100 GN=L PE=3 SV=1

---

56. 2::A0A088B1A8 **Mass:** 130006 **Score:** 47 **Matches:** 1(1) **Sequences:** 1(1) **emPAI:** 0.03
>tr|A0A088B1A8|A0A088B1A8_FRG3V Uncharacterized protein OS=Chinese giant salamander iridovirus OX=1213990 PE=4 SV=1

Query	Observed	Mr(expt)	Mr(calc)	ppm	Miss	Score	Expect	Rank	Unique	Peptide
40	367.7319	733.4493	733.4486	0.92	0	47	0.0093	1	U	R.LVYALR.V

Proteins matching the same set of peptides:
2::A0A0A0VCF0 **Mass:** 129971 **Score:** 47 **Matches:** 1(1) **Sequences:** 1(1)
>tr|A0A0A0VCF0|A0A0A0VCF0_9VIRU Uncharacterized protein OS=Common midwife toad virus OX=540070 GN=orf65L PE=4 SV=1
2::A0A0D3R3E1 **Mass:** 129898 **Score:** 47 **Matches:** 1(1) **Sequences:** 1(1)
>tr|A0A0D3R3E1|A0A0D3R3E1_9VIRU Uncharacterized protein OS=German gecko ranavirus OX=1620702 GN=GGRV_ORF33R PE=4 SV=1
2::A0A0D3R3P0 **Mass:** 129885 **Score:** 47 **Matches:** 1(1) **Sequences:** 1(1)
>tr|A0A0D3R3P0|A0A0D3R3P0_9VIRU Uncharacterized protein OS=Tortoise ranavirus OX=1585660 GN=ToRV1_ORF53L PE=4 SV=1
2::A0A0D3R3P8 **Mass:** 129855 **Score:** 47 **Matches:** 1(1) **Sequences:** 1(1)
>tr|A0A0D3R3P8|A0A0D3R3P8_9VIRU Uncharacterized protein OS=Testudo hermanni ranavirus OX=89464 GN=CH8/96_ORF50L PE=4 SV=1
2::A0A0U2I088 **Mass:** 130072 **Score:** 47 **Matches:** 1(1) **Sequences:** 1(1)
>tr|A0A0U2I088|A0A0U2I088_9VIRU Orf2-like protein OS=Ambystoma tigrinum virus OX=265294 GN=69R PE=4 SV=1
2::A0A0U2JDA3 **Mass:** 130043 **Score:** 47 **Matches:** 1(1) **Sequences:** 1(1)
>tr|A0A0U2JDA3|A0A0U2JDA3_9VIRU Orf2-like protein OS=Ambystoma tigrinum virus OX=265294 GN=73R PE=4 SV=1
2::A0A0U2K546 **Mass:** 130165 **Score:** 47 **Matches:** 1(1) **Sequences:** 1(1)
>tr|A0A0U2K546|A0A0U2K546_9VIRU Orf2-like protein OS=Ambystoma tigrinum virus OX=265294 GN=76R PE=4 SV=1
2::A0A0U2K5J2 **Mass:** 129928 **Score:** 47 **Matches:** 1(1) **Sequences:** 1(1)
>tr|A0A0U2K5J2|A0A0U2K5J2_9VIRU Orf2-like protein OS=Ambystoma tigrinum virus OX=265294 GN=75R PE=4 SV=1
2::A0A0U2K680 **Mass:** 130103 **Score:** 47 **Matches:** 1(1) **Sequences:** 1(1)
>tr|A0A0U2K680|A0A0U2K680_9VIRU Orf2-like protein OS=Ambystoma tigrinum virus OX=265294 GN=77R PE=4 SV=1
2::A0A0U2Q908 **Mass:** 130056 **Score:** 47 **Matches:** 1(1) **Sequences:** 1(1)
>tr|A0A0U2Q908|A0A0U2Q908_9VIRU Orf2-like protein OS=Ambystoma tigrinum virus OX=265294 GN=69R PE=4 SV=1
2::A0A0U2Q9F2 **Mass:** 129929 **Score:** 47 **Matches:** 1(1) **Sequences:** 1(1)
>tr|A0A0U2Q9F2|A0A0U2Q9F2_9VIRU Orf2-like protein OS=Ambystoma tigrinum virus OX=265294 GN=76R PE=4 SV=1
2::A0A0U2QCY5 **Mass:** 130132 **Score:** 47 **Matches:** 1(1) **Sequences:** 1(1)
>tr|A0A0U2QCY5|A0A0U2QCY5_9VIRU Orf2-like protein OS=Ambystoma tigrinum virus OX=265294 GN=76R PE=4 SV=1
2::A0A0U2RW92 **Mass:** 130025 **Score:** 47 **Matches:** 1(1) **Sequences:** 1(1)
>tr|A0A0U2RW92|A0A0U2RW92_9VIRU Orf2-like protein OS=Ambystoma tigrinum virus OX=265294 GN=85R PE=4 SV=1
2::A0A192GQ44 **Mass:** 129537 **Score:** 47 **Matches:** 1(1) **Sequences:** 1(1)
>tr|A0A192GQ44|A0A192GQ44_9VIRU Uncharacterized protein OS=Short-finned eel ranavirus OX=638660 GN=ORF88 PE=4 SV=1
2::A0A192GQU6 **Mass:** 129914 **Score:** 47 **Matches:** 1(1) **Sequences:** 1(1)
>tr|A0A192GQU6|A0A192GQU6_9VIRU Uncharacterized protein OS=Bohle iridovirus OX=100220 GN=ORF44 PE=4 SV=1
2::A0A1B2ITV2 **Mass:** 129713 **Score:** 47 **Matches:** 1(1) **Sequences:** 1(1)
>tr|A0A1B2ITV2|A0A1B2ITV2_FRG3V Uncharacterized protein OS=Pike perch iridovirus OX=1887315 GN=PPIV_ORF67 PE=4 SV=1
2::A0A1B2IU75 **Mass:** 129936 **Score:** 47 **Matches:** 1(1) **Sequences:** 1(1)
>tr|A0A1B2IU75|A0A1B2IU75_9VIRU Uncharacterized protein OS=Cod iridovirus OX=1887316 PE=4 SV=1
2::A0A1B2IUH1 **Mass:** 129877 **Score:** 47 **Matches:** 1(1) **Sequences:** 1(1)
>tr|A0A1B2IUH1|A0A1B2IUH1_9VIRU Uncharacterized protein OS=Ranavirus maximus OX=1887314 PE=4 SV=1
2::A0A220IGW2 **Mass:** 129865 **Score:** 47 **Matches:** 1(1) **Sequences:** 1(1)
>tr|A0A220IGW2|A0A220IGW2_FRG3V RV orf2-like protein OS=Frog virus 3 OX=10493 GN=orf41R PE=4 SV=1
2::A0A222NTK3 **Mass:** 129817 **Score:** 47 **Matches:** 1(1) **Sequences:** 1(1)
>tr|A0A222NTK3|A0A222NTK3_FRG3V Uncharacterized protein OS=Pelophylax esculentus virus OX=2023775 GN=ORF66 PE=4 SV=1
2::A0A222NTV2 **Mass:** 129927 **Score:** 47 **Matches:** 1(1) **Sequences:** 1(1)
>tr|A0A222NTV2|A0A222NTV2_FRG3V Uncharacterized protein OS=Rana esculenta virus OX=575980 GN=ORF67 PE=4 SV=1
2::A0A223PJ57 **Mass:** 129934 **Score:** 47 **Matches:** 1(1) **Sequences:** 1(1)
>tr|A0A223PJ57|A0A223PJ57_9VIRU Uncharacterized protein OS=Rana catesbeiana virus 2 OX=2026840 GN=ORF60 PE=4 SV=1
2::A0A223PJF3 **Mass:** 130006 **Score:** 47 **Matches:** 1(1) **Sequences:** 1(1)
>tr|A0A223PJF3|A0A223PJF3_9VIRU Uncharacterized protein OS=Rana catesbeiana virus OX=1380865 GN=ORF65 PE=4 SV=1
2::A0A2D0XJ85 **Mass:** 129953 **Score:** 47 **Matches:** 1(1) **Sequences:** 1(1)
>tr|A0A2D0XJ85|A0A2D0XJ85_9VIRU Uncharacterized protein OS=Common midwife toad virus OX=540070 GN=orf65L PE=4 SV=1
2::A0A2D0XMI7 **Mass:** 129869 **Score:** 47 **Matches:** 1(1) **Sequences:** 1(1)
>tr|A0A2D0XMI7|A0A2D0XMI7_9VIRU Uncharacterized protein OS=Common midwife toad virus OX=540070 GN=orf65L PE=4 SV=1
2::A0A2D0XML4 **Mass:** 129985 **Score:** 47 **Matches:** 1(1) **Sequences:** 1(1)
>tr|A0A2D0XML4|A0A2D0XML4_9VIRU Uncharacterized protein OS=Common midwife toad virus OX=540070 GN=orf65L PE=4 SV=1
2::A0A2P1GJH5 **Mass:** 129977 **Score:** 47 **Matches:** 1(1) **Sequences:** 1(1)
>tr|A0A2P1GJH5|A0A2P1GJH5_FRG3V Rrv orf2-like protein OS=Rana nigromaculata ranavirus OX=2044919 GN=ORF44R PE=4 SV=1
2::A0A2U9QGS6 **Mass:** 129952 **Score:** 47 **Matches:** 1(1) **Sequences:** 1(1)
>tr|A0A2U9QGS6|A0A2U9QGS6_FRG3V Uncharacterized protein OS=Terrapene carolina carolina ranavirus OX=2219561 GN=ORF40 PE=4 SV=1
2::A0A2U9QH16 **Mass:** 129952 **Score:** 47 **Matches:** 1(1) **Sequences:** 1(1)
>tr|A0A2U9QH16|A0A2U9QH16_FRG3V Uncharacterized protein OS=Trioceros melleri ranavirus OX=2219562 GN=ORF40 PE=4 SV=1
2::A0A3G2Y5V0 **Mass:** 130564 **Score:** 47 **Matches:** 1(1) **Sequences:** 1(1)
>tr|A0A3G2Y5V0|A0A3G2Y5V0_FRG3V RRV orf2-like protein OS=Frog virus 3 OX=10493 GN=orf43 PE=4 SV=1
2::A0A3Q8UGS1 **Mass:** 129956 **Score:** 47 **Matches:** 1(1) **Sequences:** 1(1)
>tr|A0A3Q8UGS1|A0A3Q8UGS1_9VIRU Uncharacterized protein OS=Zoo ranavirus OX=1419340 GN=ORF041 PE=4 SV=1
2::A0A3Q9T933 **Mass:** 129779 **Score:** 47 **Matches:** 1(1) **Sequences:** 1(1)
>tr|A0A3Q9T933|A0A3Q9T933_9VIRU Uncharacterized protein OS=Lumpfish ranavirus OX=2501771 GN=ORF76 PE=4 SV=1
2::A0A3T0PNK4 **Mass:** 129969 **Score:** 47 **Matches:** 1(1) **Sequences:** 1(1)
>tr|A0A3T0PNK4|A0A3T0PNK4_9VIRU Uncharacterized protein OS=Lumpfish ranavirus OX=2501771 GN=ORF76 PE=4 SV=1
2::A0A482A4A1 **Mass:** 130042 **Score:** 47 **Matches:** 1(1) **Sequences:** 1(1)
>tr|A0A482A4A1|A0A482A4A1_9VIRU Orf2-like protein OS=Ambystoma tigrinum virus OX=265294 GN=80R PE=4 SV=1
2::A0A5B8P2A1 **Mass:** 129962 **Score:** 47 **Matches:** 1(1) **Sequences:** 1(1)
>tr|A0A5B8P2A1|A0A5B8P2A1_FRG3V RRV orf2-like protein OS=Frog virus 3 OX=10493 GN=041 PE=4 SV=1
2::I2BFT3 **Mass:** 129725 **Score:** 47 **Matches:** 1(1) **Sequences:** 1(1)
>tr|I2BFT3|I2BFT3_9VIRU Uncharacterized protein OS=European catfish virus OX=84739 GN=102R PE=4 SV=1
2::Q2WET5 **Mass:** 129968 **Score:** 47 **Matches:** 1(1) **Sequences:** 1(1)
>tr|Q2WET5|Q2WET5_RTRV Uncharacterized protein OS=Rana tigrina ranavirus OX=160691 PE=4 SV=1
2::T2C5J9 **Mass:** 130020 **Score:** 47 **Matches:** 1(1) **Sequences:** 1(1)
>tr|T2C5J9|T2C5J9_FRG3V Uncharacterized protein OS=Andrias davidianus ranavirus OX=1398177 GN=ORF68L PE=4 SV=1
2::C3RWP4 **Mass:** 129964 **Score:** 47 **Matches:** 1(1) **Sequences:** 1(1)
>tr|C3RWP4|C3RWP4_FRG3V Orf2-like protein OS=Soft-shelled turtle iridovirus OX=365144 GN=ORF045R PE=4 SV=1
2::D3TTW0 **Mass:** 129864 **Score:** 47 **Matches:** 1(1) **Sequences:** 1(1)
>tr|D3TTW0|D3TTW0_9VIRU Uncharacterized protein OS=Epizootic haematopoietic necrosis virus OX=100217 PE=4 SV=1
2::V5KX95 **Mass:** 130044 **Score:** 47 **Matches:** 1(1) **Sequences:** 1(1)
>tr|V5KX95|V5KX95_FRG3V Uncharacterized protein OS=Andrias davidianus ranavirus OX=1398177 GN=69L PE=4 SV=1
2::V5N2E5 **Mass:** 130044 **Score:** 47 **Matches:** 1(1) **Sequences:** 1(1)
>tr|V5N2E5|V5N2E5_FRG3V Orf2-like protein OS=Chinese giant salamander iridovirus OX=1213990 PE=4 SV=1
2::H6WEL0 **Mass:** 133042 **Score:** 47 **Matches:** 1(1) **Sequences:** 1(1)
>tr|H6WEL0|H6WEL0_9VIRU Uncharacterized protein OS=Common midwife toad virus OX=540070 PE=4 SV=1
1::Q6GZT5 **Mass:** 129948 **Score:** 47 **Matches:** 1(1) **Sequences:** 1(1)

Uncharacterized protein 041R OS=Frog virus 3 (isolate Goorha) OX=654924 GN=FV3-041R PE=4 SV=1
2::Q6YH54 **Mass:** 130056 **Score:** 47 **Matches:** 1(1) **Sequences:** 1(1)
>tr|Q6YH54|Q6YH54_9VIRU Uncharacterized protein OS=Ambystoma tigrinum stebbensi virus OX=217733 PE=4 SV=1
2::W8SY58 **Mass:** 129934 **Score:** 47 **Matches:** 1(1) **Sequences:** 1(1)
>tr|W8SY58|W8SY58_FRG3V RRV orf2-like protein OS=Frog virus 3 OX=10493 GN=ORF41 PE=4 SV=1
2::H9XFK7 **Mass:** 129974 **Score:** 47 **Matches:** 1(1) **Sequences:** 1(1)
>tr|H9XFK7|H9XFK7_FRG3V Rrv orf2-like protein OS=Rana grylio iridovirus OX=380178 GN=ORF44R PE=4 SV=1
2::Q91R21 **Mass:** 129928 **Score:** 47 **Matches:** 1(1) **Sequences:** 1(1)
>tr|Q91R21|Q91R21_9VIRU Orf2-like protein OS=Regina ranavirus OX=80267 PE=4 SV=1

---

57. 2::A0A0A7RU81 **Mass:** 47134 **Score:** 47 **Matches:** 1(1) **Sequences:** 1(1) **emPAI:** 0.09
>tr|A0A0A7RU81|A0A0A7RU81_9CAUD Tail fiber protein OS=Clostridium phage phiCT19406B OX=1567010 GN=phiCT19406B_53 PE=4 SV=1

Query	Observed	Mr(expt)	Mr(calc)	ppm	Miss	Score	Expect	Rank	Unique	Peptide
275	437.2419	872.4692	872.4715	-2.70	1	47	0.01	1	U	R.AGNLENKK.S

Proteins matching the same set of peptides:
2::A0A0A7S1G0 **Mass:** 47134 **Score:** 47 **Matches:** 1(1) **Sequences:** 1(1)
>tr|A0A0A7S1G0|A0A0A7S1G0_9CAUD Tail fiber protein OS=Clostridium phage phiCTC2B OX=1567016 GN=phiCTC2B_53 PE=4 SV=1

---

58. 2::A0A0F6WEX4 **Mass:** 44333 **Score:** 47 **Matches:** 1(1) **Sequences:** 1(1) **emPAI:** 0.10
>tr|A0A0F6WEX4|A0A0F6WEX4_9CAUD DnaB helicase OS=Mycobacterium phage Mindy OX=1647311 GN=80 PE=4 SV=1

Query	Observed	Mr(expt)	Mr(calc)	ppm	Miss	Score	Expect	Rank	Unique	Peptide
1112	566.2989	1130.5833	1130.5866	-2.93	1	47	0.056	1	U	R.LIELGDRCR.Q

Proteins matching the same set of peptides:
2::A0A0H4TKZ1 **Mass:** 46125 **Score:** 47 **Matches:** 1(1) **Sequences:** 1(1)
>tr|A0A0H4TKZ1|A0A0H4TKZ1_9CAUD DNAB-like helicase OS=Mycobacterium phage Dusk OX=1679524 GN=SEA_DUSK_78 PE=4 SV=1
2::A0A0H4TLN8 **Mass:** 46125 **Score:** 47 **Matches:** 1(1) **Sequences:** 1(1)
>tr|A0A0H4TLN8|A0A0H4TLN8_9CAUD DNAB helicase OS=Mycobacterium phage NelitzaMV OX=1679530 GN=SEA_NELITZAMV_79 PE=4 SV=1
2::A0A0K1LK52 **Mass:** 46093 **Score:** 47 **Matches:** 1(1) **Sequences:** 1(1)
>tr|A0A0K1LK52|A0A0K1LK52_9CAUD DnaB-like helicase OS=Mycobacterium phage NoSleep OX=1673875 GN=SEA_NOSLEEP_82 PE=4 SV=1
2::A0A0K2CLB0 **Mass:** 46155 **Score:** 47 **Matches:** 1(1) **Sequences:** 1(1)
>tr|A0A0K2CLB0|A0A0K2CLB0_9CAUD DsDNA helicase OS=Mycobacterium phage Ukulele OX=1701847 GN=SEA_UKULELE_76 PE=4 SV=1
2::A0A142K6Z7 **Mass:** 46125 **Score:** 47 **Matches:** 1(1) **Sequences:** 1(1)
>tr|A0A142K6Z7|A0A142K6Z7_9CAUD Helicase OS=Mycobacterium phage TeardropMSU OX=1815609 GN=77 PE=4 SV=1
2::A0A1D8ESD2 **Mass:** 46127 **Score:** 47 **Matches:** 1(1) **Sequences:** 1(1)
>tr|A0A1D8ESD2|A0A1D8ESD2_9CAUD DsDNA helicase OS=Mycobacterium phage Pharsalus OX=1897505 GN=SEA_PHARSALUS_78 PE=4 SV=1
2::A0A1D9CA05 **Mass:** 46155 **Score:** 47 **Matches:** 1(1) **Sequences:** 1(1)
>tr|A0A1D9CA05|A0A1D9CA05_9CAUD DsDNA helicase OS=Mycobacterium phage Goldilocks OX=1906944 GN=SEA_GOLDILOCKS_84 PE=4 SV=1
2::A0A1J0GRK0 **Mass:** 46093 **Score:** 47 **Matches:** 1(1) **Sequences:** 1(1)
>tr|A0A1J0GRK0|A0A1J0GRK0_9CAUD DsDNA helicase OS=Mycobacterium phage Tuco OX=1913043 GN=SEA_TUCO_82 PE=4 SV=1
2::A0A1L7DRN2 **Mass:** 46155 **Score:** 47 **Matches:** 1(1) **Sequences:** 1(1)
>tr|A0A1L7DRN2|A0A1L7DRN2_9CAUD DsDNA helicase OS=Mycobacterium phage CrystalP OX=1932894 GN=80 PE=4 SV=1
2::A0A1S5VXU9 **Mass:** 46155 **Score:** 47 **Matches:** 1(1) **Sequences:** 1(1)
>tr|A0A1S5VXU9|A0A1S5VXU9_9CAUD DsDNA helicase OS=Mycobacterium phage Maxxinista OX=1955423 GN=SEA_MAXXINISTA_83 PE=4 SV=1
2::A0A249XPC5 **Mass:** 46155 **Score:** 47 **Matches:** 1(1) **Sequences:** 1(1)
>tr|A0A249XPC5|A0A249XPC5_9CAUD DsDNA helicase OS=Mycobacterium phage MadamMonkfish OX=2027897 GN=SEA_MADAMMONKFISH_81 PE=4 SV=1
2::A0A2D1GBD7 **Mass:** 46155 **Score:** 47 **Matches:** 1(1) **Sequences:** 1(1)
>tr|A0A2D1GBD7|A0A2D1GBD7_9CAUD DsDNA helicase OS=Mycobacterium phage FireRed OX=2041531 GN=SEA_FIRERED_85 PE=4 SV=1
2::A0A2D1GGZ9 **Mass:** 46155 **Score:** 47 **Matches:** 1(1) **Sequences:** 1(1)
>tr|A0A2D1GGZ9|A0A2D1GGZ9_9CAUD DsDNA helicase OS=Mycobacterium phage Murica OX=2041543 GN=SEA_MURICA_84 PE=4 SV=1
2::A0A2D1GIQ7 **Mass:** 46155 **Score:** 47 **Matches:** 1(1) **Sequences:** 1(1)
>tr|A0A2D1GIQ7|A0A2D1GIQ7_9CAUD DsDNA helicase OS=Mycobacterium phage Sassay OX=2041547 GN=SEA_SASSAY_76 PE=4 SV=1
2::A0A2D1GJT9 **Mass:** 46155 **Score:** 47 **Matches:** 1(1) **Sequences:** 1(1)
>tr|A0A2D1GJT9|A0A2D1GJT9_9CAUD DsDNA helicase OS=Mycobacterium phage Terminus OX=2041552 GN=SEA_TERMINUS_84 PE=4 SV=1
2::A0A2D1GKY0 **Mass:** 46155 **Score:** 47 **Matches:** 1(1) **Sequences:** 1(1)
>tr|A0A2D1GKY0|A0A2D1GKY0_9CAUD DsDNA helicase OS=Mycobacterium phage Willez OX=2041556 GN=SEA_WILLEZ_76 PE=4 SV=1
2::A0A2D1GLB8 **Mass:** 46093 **Score:** 47 **Matches:** 1(1) **Sequences:** 1(1)
>tr|A0A2D1GLB8|A0A2D1GLB8_9CAUD DsDNA helicase OS=Mycobacterium phage YassJohnny OX=2041557 GN=SEA_YASSJOHNNY_78 PE=4 SV=1
2::A0A2D2W3B7 **Mass:** 46155 **Score:** 47 **Matches:** 1(1) **Sequences:** 1(1)
>tr|A0A2D2W3B7|A0A2D2W3B7_9CAUD DsDNA helicase OS=Mycobacterium phage MISSy OX=2041541 GN=SEA_MISSY_82 PE=4 SV=1
2::A0A2H4PBM3 **Mass:** 46155 **Score:** 47 **Matches:** 1(1) **Sequences:** 1(1)
>tr|A0A2H4PBM3|A0A2H4PBM3_9CAUD DsDNA helicase OS=Mycobacterium phage Youngblood OX=2047847 GN=SEA_YOUNGBLOOD_84 PE=4 SV=1
2::A0A2L1IY67 **Mass:** 46155 **Score:** 47 **Matches:** 1(1) **Sequences:** 1(1)
>tr|A0A2L1IY67|A0A2L1IY67_9CAUD DsDNA helicase OS=Mycobacterium phage Kimchi OX=2079420 GN=SEA_KIMCHI_85 PE=4 SV=1
2::A0A2P0ZZT7 **Mass:** 46093 **Score:** 47 **Matches:** 1(1) **Sequences:** 1(1)
>tr|A0A2P0ZZT7|A0A2P0ZZT7_9CAUD DsDNA helicase OS=Mycobacterium phage Asriel OX=2081618 GN=80 PE=4 SV=1
2::A0A2P1A073 **Mass:** 46093 **Score:** 47 **Matches:** 1(1) **Sequences:** 1(1)
>tr|A0A2P1A073|A0A2P1A073_9CAUD DsDNA helicase OS=Mycobacterium phage Barbarian OX=2081619 GN=80 PE=4 SV=1
2::A0A2P1A1L4 **Mass:** 46154 **Score:** 47 **Matches:** 1(1) **Sequences:** 1(1)
>tr|A0A2P1A1L4|A0A2P1A1L4_9CAUD DsDNA helicase OS=Mycobacterium phage Gage OX=2081624 GN=83 PE=4 SV=1
2::A0A2P1A3A0 **Mass:** 46155 **Score:** 47 **Matches:** 1(1) **Sequences:** 1(1)
>tr|A0A2P1A3A0|A0A2P1A3A0_9CAUD DsDNA helicase OS=Mycobacterium phage Sotrice96 OX=2081629 GN=82 PE=4 SV=1
2::A0A2P1JVI4 **Mass:** 46155 **Score:** 47 **Matches:** 1(1) **Sequences:** 1(1)
>tr|A0A2P1JVI4|A0A2P1JVI4_9CAUD DnaB-like DNA helicase OS=Mycobacterium phage RiverMonster OX=2108119 GN=81 PE=4 SV=1
2::A0A2P1N4L2 **Mass:** 46155 **Score:** 47 **Matches:** 1(1) **Sequences:** 1(1)
>tr|A0A2P1N4L2|A0A2P1N4L2_9CAUD DsDNA helicase OS=Mycobacterium phage MPhalcon OX=2126787 GN=82 PE=4 SV=1
2::A0A2Z5H8K2 **Mass:** 46093 **Score:** 47 **Matches:** 1(1) **Sequences:** 1(1)
>tr|A0A2Z5H8K2|A0A2Z5H8K2_9CAUD DsDNA helicase OS=Mycobacterium phage Adnama OX=2250354 GN=85 PE=4 SV=1
2::A0A2Z5HB91 **Mass:** 46093 **Score:** 47 **Matches:** 1(1) **Sequences:** 1(1)
>tr|A0A2Z5HB91|A0A2Z5HB91_9CAUD DnaB-like dsDNA helicase OS=Mycobacterium phage DoctorDiddles OX=2250376 GN=80 PE=4 SV=1
2::A0A2Z5HDT0 **Mass:** 46155 **Score:** 47 **Matches:** 1(1) **Sequences:** 1(1)
>tr|A0A2Z5HDT0|A0A2Z5HDT0_9CAUD DnaB-like dsDNA helicase OS=Mycobacterium phage Icee OX=2250380 GN=82 PE=4 SV=1
2::A0A345KX72 **Mass:** 46093 **Score:** 47 **Matches:** 1(1) **Sequences:** 1(1)
>tr|A0A345KX72|A0A345KX72_9CAUD DnaB-like dsDNA helicase OS=Mycobacterium phage IHOP OX=2250301 GN=79 PE=4 SV=1
2::A0A345KZ05 **Mass:** 46093 **Score:** 47 **Matches:** 1(1) **Sequences:** 1(1)
>tr|A0A345KZ05|A0A345KZ05_9CAUD DnaB-like dsDNA helicase OS=Mycobacterium phage Phaja OX=2250306 GN=79 PE=4 SV=1
2::A0A345L0A9 **Mass:** 46155 **Score:** 47 **Matches:** 1(1) **Sequences:** 1(1)
>tr|A0A345L0A9|A0A345L0A9_9CAUD DnaB-like dsDNA helicase OS=Mycobacterium phage ShereKhan OX=2250308 GN=79 PE=4 SV=1
2::A0A345L3N9 **Mass:** 46155 **Score:** 47 **Matches:** 1(1) **Sequences:** 1(1)
>tr|A0A345L3N9|A0A345L3N9_9CAUD DsDNA helicase OS=Mycobacterium phage Glexan OX=2250328 GN=80 PE=4 SV=1
2::A0A345L5R4 **Mass:** 46155 **Score:** 47 **Matches:** 1(1) **Sequences:** 1(1)
>tr|A0A345L5R4|A0A345L5R4_9CAUD DnaB-like dsDNA helicase OS=Mycobacterium phage Simpliphy OX=2250332 GN=80 PE=4 SV=1
2::A0A345L6F9 **Mass:** 46125 **Score:** 47 **Matches:** 1(1) **Sequences:** 1(1)
>tr|A0A345L6F9|A0A345L6F9_9CAUD DnaB-like dsDNA helicase OS=Mycobacterium phage TBrady12 OX=2250334 GN=84 PE=4 SV=1
2::A0A345M4R9 **Mass:** 46155 **Score:** 47 **Matches:** 1(1) **Sequences:** 1(1)
>tr|A0A345M4R9|A0A345M4R9_9CAUD DnaB-like dsDNA helicase OS=Mycobacterium phage Hopey OX=2283255 GN=81 PE=4 SV=1
2::A0A345M5H8 **Mass:** 46125 **Score:** 47 **Matches:** 1(1) **Sequences:** 1(1)
>tr|A0A345M5H8|A0A345M5H8_9CAUD DnaB-like dsDNA helicase OS=Mycobacterium phage Inca OX=2283256 GN=78 PE=4 SV=1
2::A0A345MDC2 **Mass:** 46155 **Score:** 47 **Matches:** 1(1) **Sequences:** 1(1)
>tr|A0A345MDC2|A0A345MDC2_9CAUD Helicase OS=Mycobacterium phage xkcd OX=2283301 GN=84 PE=4 SV=1
2::A0A385DQV3 **Mass:** 46155 **Score:** 47 **Matches:** 1(1) **Sequences:** 1(1)
>tr|A0A385DQV3|A0A385DQV3_9CAUD DnaB-like dsDNA helicase OS=Mycobacterium phage Emmina OX=2301564 GN=81 PE=4 SV=1
2::A0A385DWB4 **Mass:** 46155 **Score:** 47 **Matches:** 1(1) **Sequences:** 1(1)
>tr|A0A385DWB4|A0A385DWB4_9CAUD DnaB-like dsDNA helicase OS=Mycobacterium phage Easy2Say OX=2301705 GN=82 PE=4 SV=1
2::A0A385UHL5 **Mass:** 46127 **Score:** 47 **Matches:** 1(1) **Sequences:** 1(1)
>tr|A0A385UHL5|A0A385UHL5_9CAUD DnaB-like dsDNA helicase OS=Mycobacterium phage Paperbeatsrock OX=2301616 GN=80 PE=4 SV=1
2::A0A386KHI8 **Mass:** 46155 **Score:** 47 **Matches:** 1(1) **Sequences:** 1(1)

>tr|A0A386KHI8|A0A386KHI8_9CAUD DnaB-like dsDNA helicase OS=Mycobacterium phage Gemini OX=2315538 GN=86 PE=4 SV=1
2::A0A3G3LY80 **Mass:** 46155 **Score:** 47 **Matches:** 1(1) **Sequences:** 1(1)
>tr|A0A3G3LY80|A0A3G3LY80_9CAUD DsDNA helicase OS=Mycobacterium phage BaboJay OX=2484208 GN=78 PE=4 SV=1
2::A0A3G3M098 **Mass:** 46127 **Score:** 47 **Matches:** 1(1) **Sequences:** 1(1)
>tr|A0A3G3M098|A0A3G3M098_9CAUD DsDNA helicase OS=Mycobacterium phage Manda OX=2484211 GN=82 PE=4 SV=1
2::A0A3G3M2G3 **Mass:** 46127 **Score:** 47 **Matches:** 1(1) **Sequences:** 1(1)
>tr|A0A3G3M2G3|A0A3G3M2G3_9CAUD DsDNA helicase OS=Mycobacterium phage Pat3 OX=2484214 GN=79 PE=4 SV=1
2::A0A411BUI2 **Mass:** 46155 **Score:** 47 **Matches:** 1(1) **Sequences:** 1(1)
>tr|A0A411BUI2|A0A411BUI2_9CAUD DsDNA helicase OS=Mycobacterium phage Czyszczon1 OX=2502427 GN=82 PE=4 SV=1
2::A0A411BYC4 **Mass:** 46093 **Score:** 47 **Matches:** 1(1) **Sequences:** 1(1)
>tr|A0A411BYC4|A0A411BYC4_9CAUD DsDNA helicase OS=Mycobacterium phage Cookies OX=2502438 GN=78 PE=4 SV=1
2::A0A411CRU3 **Mass:** 46155 **Score:** 47 **Matches:** 1(1) **Sequences:** 1(1)
>tr|A0A411CRU3|A0A411CRU3_9CAUD DnaB-like dsDNA helicase OS=Mycobacterium phage Nimrod OX=2510511 GN=82 PE=4 SV=1
2::A0A481W8J0 **Mass:** 46093 **Score:** 47 **Matches:** 1(1) **Sequences:** 1(1)
>tr|A0A481W8J0|A0A481W8J0_9CAUD DnaB-like dsDNA helicase OS=Mycobacterium phage Moldemort OX=2530188 GN=84 PE=4 SV=1
2::A0A4D6DZN0 **Mass:** 46155 **Score:** 47 **Matches:** 1(1) **Sequences:** 1(1)
>tr|A0A4D6DZN0|A0A4D6DZN0_9CAUD DnaB-like dsDNA helicase OS=Mycobacterium phage HanKaySha OX=2562186 GN=81 PE=4 SV=1
2::A0A4D6T8M2 **Mass:** 46155 **Score:** 47 **Matches:** 1(1) **Sequences:** 1(1)
>tr|A0A4D6T8M2|A0A4D6T8M2_9CAUD DNAB-like dsDNA helicase OS=Mycobacterium phage Lilizi OX=2565512 GN=82 PE=4 SV=1
2::A0A4Y6EG41 **Mass:** 46093 **Score:** 47 **Matches:** 1(1) **Sequences:** 1(1)
>tr|A0A4Y6EG41|A0A4Y6EG41_9CAUD DnaB-like dsDNA helicase OS=Mycobacterium phage ChotaBhai OX=2588139 GN=81 PE=4 SV=1
2::A0A514DEA8 **Mass:** 46155 **Score:** 47 **Matches:** 1(1) **Sequences:** 1(1)
>tr|A0A514DEA8|A0A514DEA8_9CAUD DnaB-like dsDNA helicase OS=Mycobacterium phage Flypotenuse OX=2591220 GN=77 PE=4 SV=1
2::A0A514TYN8 **Mass:** 46093 **Score:** 47 **Matches:** 1(1) **Sequences:** 1(1)
>tr|A0A514TYN8|A0A514TYN8_9CAUD DnaB-like dsDNA helicase OS=Mycobacterium phage Gator OX=2593347 GN=79 PE=4 SV=1
2::A0A514TZM3 **Mass:** 46155 **Score:** 47 **Matches:** 1(1) **Sequences:** 1(1)
>tr|A0A514TZM3|A0A514TZM3_9CAUD DnaB-like helicase OS=Mycobacterium phage Tomaszewski OX=2593354 GN=76 PE=4 SV=1
2::G1D0T1 **Mass:** 46093 **Score:** 47 **Matches:** 1(1) **Sequences:** 1(1)
>tr|G1D0T1|G1D0T1_9CAUD DnaB-like helicase OS=Mycobacterium virus Bask21 OX=1034100 GN=81 PE=4 SV=1
2::G1D2D1 **Mass:** 46155 **Score:** 47 **Matches:** 1(1) **Sequences:** 1(1)
>tr|G1D2D1|G1D2D1_9CAUD DnaB OS=Mycobacterium phage Henry OX=1034105 GN=79 PE=4 SV=1
2::G1D5U5 **Mass:** 46093 **Score:** 47 **Matches:** 1(1) **Sequences:** 1(1)
>tr|G1D5U5|G1D5U5_9CAUD DnaB-like helicase OS=Mycobacterium phage SirDuracell OX=1034116 GN=80 PE=4 SV=1
2::G1DI15 **Mass:** 44333 **Score:** 47 **Matches:** 1(1) **Sequences:** 1(1)
>tr|G1DI15|G1DI15_9CAUD DnaB OS=Mycobacterium phage Toto OX=1035485 GN=79 PE=4 SV=1
2::G1DIK5 **Mass:** 46155 **Score:** 47 **Matches:** 1(1) **Sequences:** 1(1)
>tr|G1DIK5|G1DIK5_9CAUD DnaB OS=Mycobacterium phage Rakim OX=1035483 GN=82 PE=4 SV=1
2::G1JS60 **Mass:** 46155 **Score:** 47 **Matches:** 1(1) **Sequences:** 1(1)
>tr|G1JS60|G1JS60_9CAUD DnaB-like helicase OS=Mycobacterium phage Lilac OX=1070990 GN=83 PE=4 SV=1
2::G1JSY9 **Mass:** 46155 **Score:** 47 **Matches:** 1(1) **Sequences:** 1(1)
>tr|G1JSY9|G1JSY9_9CAUD DnaB-like helicase OS=Mycobacterium phage Elph10 OX=1070989 GN=81 PE=4 SV=1
2::G1JWU9 **Mass:** 46155 **Score:** 47 **Matches:** 1(1) **Sequences:** 1(1)
>tr|G1JWU9|G1JWU9_9CAUD DnaB-like helicase OS=Mycobacterium virus Eureka OX=1074306 GN=79 PE=4 SV=1
2::Q1A0Y9 **Mass:** 46155 **Score:** 47 **Matches:** 1(1) **Sequences:** 1(1)
>tr|Q1A0Y9|Q1A0Y9_9CAUD SF4 helicase domain-containing protein OS=Mycobacterium virus 244 OX=373403 GN=82 PE=4 SV=1
2::T2A998 **Mass:** 46155 **Score:** 47 **Matches:** 1(1) **Sequences:** 1(1)
>tr|T2A998|T2A998_9CAUD DNA B-like helicase OS=Mycobacterium phage Quink OX=1354514 GN=QUINK_84 PE=4 SV=1
2::R4JFG4 **Mass:** 46155 **Score:** 47 **Matches:** 1(1) **Sequences:** 1(1)
>tr|R4JFG4|R4JFG4_9CAUD DnaB-like helicase OS=Mycobacterium phage Phrux OX=1327774 GN=78 PE=4 SV=1
2::R4TP37 **Mass:** 46155 **Score:** 47 **Matches:** 1(1) **Sequences:** 1(1)
>tr|R4TP37|R4TP37_9CAUD DnaB-like helicase OS=Mycobacterium phage Dumbo OX=1327764 GN=81 PE=4 SV=1
2::R4TST2 **Mass:** 46093 **Score:** 47 **Matches:** 1(1) **Sequences:** 1(1)
>tr|R4TST2|R4TST2_9CAUD DnaB-like helicase OS=Mycobacterium phage Phaux OX=1327937 GN=83 PE=4 SV=1
2::R4TTQ4 **Mass:** 46183 **Score:** 47 **Matches:** 1(1) **Sequences:** 1(1)
>tr|R4TTQ4|R4TTQ4_9CAUD DnaB-like helicase OS=Mycobacterium phage Murphy OX=1327939 GN=81 PE=4 SV=1
2::B5A642 **Mass:** 46155 **Score:** 47 **Matches:** 1(1) **Sequences:** 1(1)
>tr|B5A642|B5A642_9CAUD DnaB OS=Mycobacterium virus Porky OX=546185 GN=Porky_80 PE=4 SV=1
2::B5A743 **Mass:** 44303 **Score:** 47 **Matches:** 1(1) **Sequences:** 1(1)
>tr|B5A743|B5A743_9CAUD SF4 helicase domain-containing protein OS=Mycobacterium virus Kostya OX=546183 GN=Kostya_81 PE=4 SV=1
2::S5MCJ4 **Mass:** 46125 **Score:** 47 **Matches:** 1(1) **Sequences:** 1(1)
>tr|S5MCJ4|S5MCJ4_9CAUD DnaB-like helicase OS=Mycobacterium phage ABCat OX=1358124 GN=78 PE=4 SV=1
2::S5XZL6 **Mass:** 46155 **Score:** 47 **Matches:** 1(1) **Sequences:** 1(1)
>tr|S5XZL6|S5XZL6_9CAUD DnaB-like helicase OS=Mycobacterium phage Contagion OX=1340833 GN=77 PE=4 SV=1
2::S5Y7A5 **Mass:** 46155 **Score:** 47 **Matches:** 1(1) **Sequences:** 1(1)
>tr|S5Y7A5|S5Y7A5_9CAUD DnaB-like helicase OS=Mycobacterium phage DrDrey OX=1357669 GN=81 PE=4 SV=1
2::S5Z9L3 **Mass:** 46155 **Score:** 47 **Matches:** 1(1) **Sequences:** 1(1)
>tr|S5Z9L3|S5Z9L3_9CAUD DnaB-like helicase OS=Mycobacterium phage Goku OX=1383057 GN=79 PE=4 SV=1
2::V5R856 **Mass:** 46155 **Score:** 47 **Matches:** 1(1) **Sequences:** 1(1)
>tr|V5R856|V5R856_9CAUD DnaB-like helicase OS=Mycobacterium phage HufflyPuff OX=1430411 GN=84 PE=4 SV=1
2::V5R8G7 **Mass:** 46155 **Score:** 47 **Matches:** 1(1) **Sequences:** 1(1)
>tr|V5R8G7|V5R8G7_9CAUD DnaB-like helicase OS=Mycobacterium phage PhatBacter OX=1391181 GN=85 PE=4 SV=1
2::V5R8I8 **Mass:** 46155 **Score:** 47 **Matches:** 1(1) **Sequences:** 1(1)
>tr|V5R8I8|V5R8I8_9CAUD SF4 helicase domain-containing protein OS=Mycobacterium phage Nala OX=1391180 GN=83 PE=4 SV=1
2::V5R8U5 **Mass:** 46155 **Score:** 47 **Matches:** 1(1) **Sequences:** 1(1)
>tr|V5R8U5|V5R8U5_9CAUD DnaB-like helicase OS=Mycobacterium phage Bruin OX=1391179 GN=78 PE=4 SV=1
2::V5RCC2 **Mass:** 46155 **Score:** 47 **Matches:** 1(1) **Sequences:** 1(1)
>tr|V5RCC2|V5RCC2_9CAUD DnaB-like helicase OS=Mycobacterium phage Mosby OX=1429913 GN=77 PE=4 SV=1
2::C9DCQ0 **Mass:** 46155 **Score:** 47 **Matches:** 1(1) **Sequences:** 1(1)
>tr|C9DCQ0|C9DCQ0_9CAUD DnaB OS=Mycobacterium virus Pumpkin OX=663559 GN=84 PE=4 SV=1
2::Q857T0 **Mass:** 46155 **Score:** 47 **Matches:** 1(1) **Sequences:** 1(1)
>tr|Q857T0|Q857T0_9CAUD SF4 helicase domain-containing protein OS=Mycobacterium virus CJW1 OX=205869 GN=81 PE=4 SV=1

---

59. 2::A0A0M5M5R9 **Mass:** 16061 **Score:** 47 **Matches:** 1(1) **Sequences:** 1(1) **emPAI:** 0.29
>tr|A0A0M5M5R9|A0A0M5M5R9_9CAUD SsDNA-binding protein OS=Citrobacter phage Margaery OX=1701810 GN=CPT_Margaery222 PE=4 SV=1

Query	Observed	Mr(expt)	Mr(calc)	ppm	Miss	Score	Expect	Rank	Unique	Peptide
257	435.2476	868.4806	868.4807	-0.06	0	47	0.0066	1	U	R.LLFYTGR.H

Proteins matching the same set of peptides:
2::A0A1W5N0M8 **Mass:** 16061 **Score:** 47 **Matches:** 1(1) **Sequences:** 1(1)
>tr|A0A1W5N0M8|A0A1W5N0M8_9CAUD Recombination, repair and ssDNA binding protein OS=Cronobacter phage vB_CsaM_leB OX=1885242 GN=B_221 PE=4 SV=1
2::A0A1W5P0W1 **Mass:** 16070 **Score:** 47 **Matches:** 1(1) **Sequences:** 1(1)
>tr|A0A1W5P0W1|A0A1W5P0W1_9CAUD Recombination, repair and ssDNA binding protein OS=Cronobacter phage vB_CsaM_leE OX=1873954 GN=E_226 PE=4 SV=1
2::A0A1X8VCA5 **Mass:** 16061 **Score:** 47 **Matches:** 1(1) **Sequences:** 1(1)
>tr|A0A1X8VCA5|A0A1X8VCA5_9CAUD Recombination, repair and sssDNA binding protein OS=Cronobacter phage vB_CsaM_leN OX=1885245 GN=N_227 PE=4 SV=1
2::A0A3B8DIS1 **Mass:** 16061 **Score:** 47 **Matches:** 1(1) **Sequences:** 1(1)
>tr|A0A3B8DIS1|A0A3B8DIS1_9CAUD UvsY-like protein OS=Citrobacter phage Maroon OX=2315469 GN=CPT_Maroon_218 PE=4 SV=1
2::K4F7S7 **Mass:** 16061 **Score:** 47 **Matches:** 1(1) **Sequences:** 1(1)
>tr|K4F7S7|K4F7S7_9CAUD Recombination, repair and ssDNA binding protein OS=Cronobacter phage vB_CsaM_GAP161 OX=1141138 GN=GAP161_213 PE=4 SV=1

---

60. 2::A0A0D3MVZ4 **Mass:** 20513 **Score:** 46 **Matches:** 1(1) **Sequences:** 1(1) **emPAI:** 0.22
>tr|A0A0D3MVZ4|A0A0D3MVZ4_9ADEN Ig-like domain-containing protein OS=Duck adenovirus 1 OX=130329 PE=4 SV=1

Query	Observed	Mr(expt)	Mr(calc)	ppm	Miss	Score	Expect	Rank	Unique	Peptide
119	404.2221	806.4296	806.4286	1.24	0	46	0.016	1	U	K.LLDYQR.L

Proteins matching the same set of peptides:
2::O11432 **Mass:** 20527 **Score:** 46 **Matches:** 1(1) **Sequences:** 1(1)
>tr|O11432|O11432_9ADEN Ig-like domain-containing protein OS=Duck adenovirus 1 OX=130329 PE=4 SV=1

---

61. 2::A0A1D8EZ70 **Mass:** 56951 **Score:** 46 **Matches:** 1(1) **Sequences:** 1(1) **emPAI:** 0.08
>tr|A0A1D8EZ70|A0A1D8EZ70_9CAUD Lysin A OS=Mycobacterium phage Badger OX=1881140 GN=SEA_BADGER_8 PE=4 SV=1

Query	Observed	Mr(expt)	Mr(calc)	ppm	Miss	Score	Expect	Rank	Unique	Peptide
97	393.7456	785.4767	785.4759	1.02	0	46	0.02	1	U	R.GTLAGVIR.A

Proteins matching the same set of peptides:
2::A0A1J0MAF1 **Mass:** 56951 **Score:** 46 **Matches:** 1(1) **Sequences:** 1(1)
>tr|A0A1J0MAF1|A0A1J0MAF1_9CAUD Lysin A OS=Mycobacterium phage Achebe OX=1916113 GN=8 PE=4 SV=1
2::A0A3S9UMP6 **Mass:** 56951 **Score:** 46 **Matches:** 1(1) **Sequences:** 1(1)
>tr|A0A3S9UMP6|A0A3S9UMP6_9CAUD Lysin A OS=Mycobacterium phage Cici OX=2499034 GN=8 PE=4 SV=1
2::A0A411BUR2 **Mass:** 56951 **Score:** 46 **Matches:** 1(1) **Sequences:** 1(1)
>tr|A0A411BUR2|A0A411BUR2_9CAUD Lysin A OS=Mycobacterium phage Katalie136 OX=2502428 GN=8 PE=4 SV=1
2::A0A411BZ85 **Mass:** 56951 **Score:** 46 **Matches:** 1(1) **Sequences:** 1(1)
>tr|A0A411BZ85|A0A411BZ85_9CAUD Lysin A OS=Mycobacterium phage Datway OX=2502440 GN=8 PE=4 SV=1
2::A0A4Y5P096 **Mass:** 56951 **Score:** 46 **Matches:** 1(1) **Sequences:** 1(1)
>tr|A0A4Y5P096|A0A4Y5P096_9CAUD Lysin A OS=Mycobacterium phage Xena OX=2584499 GN=8 PE=4 SV=1
2::G1BKY1 **Mass:** 56951 **Score:** 46 **Matches:** 1(1) **Sequences:** 1(1)
>tr|G1BKY1|G1BKY1_9CAUD Lysin A OS=Mycobacterium virus Backyardigan OX=1034129 GN=8 PE=4 SV=1
2::G1FN95 **Mass:** 56951 **Score:** 46 **Matches:** 1(1) **Sequences:** 1(1)
>tr|G1FN95|G1FN95_9CAUD Lysin A OS=Mycobacterium phage Wile OX=1056833 GN=8 PE=4 SV=1

---

62. 2::A0A516LNF5 **Mass:** 97581 **Score:** 46 **Matches:** 2(2) **Sequences:** 1(1) **emPAI:** 0.04
>tr|A0A516LNF5|A0A516LNF5_9VIRU Peptidase S74 domain-containing protein OS=Prokaryotic dsDNA virus sp. OX=2591644 GN=GOVbin3762_66 PE=4 SV=1

Query	Observed	Mr(expt)	Mr(calc)	ppm	Miss	Score	Expect	Rank	Unique	Peptide
1162	573.7988	1145.5830	1145.5815	1.25	0	44	0.037	1	U	K.ELSAEVEELK.A 1161

---

63. 2::A0A076JU09 **Mass:** 84694 **Score:** 46 **Matches:** 1(1) **Sequences:** 1(1) **emPAI:** 0.05
>tr|A0A076JU09|A0A076JU09_9ALPH Portal protein OS=Equid alphaherpesvirus 1 OX=10326 PE=3 SV=1

Query	Observed	Mr(expt)	Mr(calc)	ppm	Miss	Score	Expect	Rank	Unique	Peptide
47	373.7064	745.3982	745.3970	1.61	0	46	0.016	1	U	K.TIEDLR.T

Proteins matching the same set of peptides:
2::A0A0A7D9M5 **Mass:** 84568 **Score:** 46 **Matches:** 1(1) **Sequences:** 1(1)
>tr|A0A0A7D9M5|A0A0A7D9M5_9ALPH Portal protein OS=Equid alphaherpesvirus 1 OX=10326 GN=ORF56 PE=3 SV=1
2::A0A0A7DAD3 **Mass:** 84554 **Score:** 46 **Matches:** 1(1) **Sequences:** 1(1)
>tr|A0A0A7DAD3|A0A0A7DAD3_9ALPH Portal protein OS=Equid alphaherpesvirus 1 OX=10326 GN=ORF56 PE=3 SV=1
2::A0A0X9ZHI2 **Mass:** 84184 **Score:** 46 **Matches:** 1(1) **Sequences:** 1(1)
>tr|A0A0X9ZHI2|A0A0X9ZHI2_9ALPH Portal protein OS=Equid alphaherpesvirus 4 OX=10331 GN=ORF56 PE=3 SV=1
2::A0A0Y0A4E8 **Mass:** 84170 **Score:** 46 **Matches:** 1(1) **Sequences:** 1(1)
>tr|A0A0Y0A4E8|A0A0Y0A4E8_9ALPH Portal protein OS=Equid alphaherpesvirus 4 OX=10331 GN=ORF56 PE=3 SV=1
2::A0A1J1E0I6 **Mass:** 84210 **Score:** 46 **Matches:** 1(1) **Sequences:** 1(1)
>tr|A0A1J1E0I6|A0A1J1E0I6_9ALPH Portal protein OS=Equid alphaherpesvirus 4 OX=10331 GN=ORF56 PE=3 SV=1
2::A0A2K9QRI7 **Mass:** 84599 **Score:** 46 **Matches:** 1(1) **Sequences:** 1(1)
>tr|A0A2K9QRI7|A0A2K9QRI7_9ALPH Portal protein OS=Equid alphaherpesvirus 8 OX=39637 GN=ORF56 PE=3 SV=1
2::A0A2K9QRR7 **Mass:** 84666 **Score:** 46 **Matches:** 1(1) **Sequences:** 1(1)
>tr|A0A2K9QRR7|A0A2K9QRR7_9ALPH Portal protein OS=Equid alphaherpesvirus 8 OX=39637 GN=ORF56 PE=3 SV=1
2::A0A2P0N522 **Mass:** 84610 **Score:** 46 **Matches:** 1(1) **Sequences:** 1(1)
>tr|A0A2P0N522|A0A2P0N522_9ALPH Portal protein OS=Equid alphaherpesvirus 1 OX=10326 GN=ORF56 PE=3 SV=1
2::A0A2P0N7P0 **Mass:** 84568 **Score:** 46 **Matches:** 1(1) **Sequences:** 1(1)
>tr|A0A2P0N7P0|A0A2P0N7P0_9ALPH Portal protein OS=Equid alphaherpesvirus 1 OX=10326 GN=ORF56 PE=3 SV=1
2::A0A2P0N8A0 **Mass:** 84525 **Score:** 46 **Matches:** 1(1) **Sequences:** 1(1)
>tr|A0A2P0N8A0|A0A2P0N8A0_9ALPH Portal protein OS=Equid alphaherpesvirus 1 OX=10326 GN=ORF56 PE=3 SV=1
2::A0A516LDA7 **Mass:** 97041 **Score:** 46 **Matches:** 1(1) **Sequences:** 1(1)
>tr|A0A516LDA7|A0A516LDA7_9VIRU Putative DNA ligase OS=Prokaryotic dsDNA virus sp. OX=2591644 GN=GOVbin4206_77 PE=4 SV=1
2::I1V8H0 **Mass:** 77644 **Score:** 46 **Matches:** 1(1) **Sequences:** 1(1)
>tr|I1V8H0|I1V8H0_9ALPH Portal protein OS=Equid alphaherpesvirus 8 OX=39637 GN=ORF56 PE=3 SV=1
2::M4JG74 **Mass:** 157566 **Score:** 46 **Matches:** 1(1) **Sequences:** 1(1)
>tr|M4JG74|M4JG74_9VIRU Capsid protein VP1 OS=Haloarcula hispanica virus PH1 OX=1282967 GN=HhPH1_gp12 PE=4 SV=1
2::B7FEJ8 **Mass:** 77689 **Score:** 46 **Matches:** 1(1) **Sequences:** 1(1)
>tr|B7FEJ8|B7FEJ8_9ALPH Portal protein OS=Equid alphaherpesvirus 9 OX=55744 PE=3 SV=1
2::L8B1V9 **Mass:** 84640 **Score:** 46 **Matches:** 1(1) **Sequences:** 1(1)
>tr|L8B1V9|L8B1V9_9ALPH Portal protein OS=Equid alphaherpesvirus 1 OX=10326 GN=ORF56 PE=3 SV=1
1::P28944 **Mass:** 84568 **Score:** 46 **Matches:** 1(1) **Sequences:** 1(1)
Portal protein OS=Equine herpesvirus 1 (strain Ab4p) OX=31520 GN=56 PE=3 SV=1
2::O39296 **Mass:** 84212 **Score:** 46 **Matches:** 1(1) **Sequences:** 1(1)
>tr|O39296|O39296_EHV4 Portal protein OS=Equine herpesvirus 4 (strain 1942) OX=10333 GN=56 PE=3 SV=1
1::P84458 **Mass:** 84568 **Score:** 46 **Matches:** 1(1) **Sequences:** 1(1)
Portal protein OS=Equine herpesvirus 1 (strain V592) OX=310273 GN=56 PE=3 SV=1

---

64. 2::A0A4D5ZFG5 **Mass:** 22006 **Score:** 46 **Matches:** 2(2) **Sequences:** 1(1) **emPAI:** 0.21
>tr|A0A4D5ZFG5|A0A4D5ZFG5_9VIRU Uncharacterized protein OS=Streptococcus satellite phage Javan147 OX=2558541 GN=JavanS147_0011 PE=4 SV=1

Query	Observed	Mr(expt)	Mr(calc)	ppm	Miss	Score	Expect	Rank	Unique	Peptide
418	464.7764	927.5383	927.5389	-0.71	0	43	0.022	1	U	R.QDLLQAIK.N 419

---

65. 2::A0A160DHM9 **Mass:** 9089 **Score:** 45 **Matches:** 1(1) **Sequences:** 1(1) **emPAI:** 0.56
>tr|A0A160DHM9|A0A160DHM9_9CAUD Uncharacterized protein OS=Gordonia phage Jeanie OX=1838068 GN=3 PE=4 SV=1

Query	Observed	Mr(expt)	Mr(calc)	ppm	Miss	Score	Expect	Rank	Unique	Peptide
161	412.2427	822.4709	822.4712	-0.37	0	45	0.0026	1	U	R.LVADHLR.H

Proteins matching the same set of peptides:
2::A0A160DHP6 **Mass:** 9089 **Score:** 45 **Matches:** 1(1) **Sequences:** 1(1)
>tr|A0A160DHP6|A0A160DHP6_9CAUD Uncharacterized protein OS=Gordonia phage McGonagall OX=1838072 GN=3 PE=4 SV=1
2::A0A4Y6EJ81 **Mass:** 8962 **Score:** 45 **Matches:** 1(1) **Sequences:** 1(1)
>tr|A0A4Y6EJ81|A0A4Y6EJ81_9CAUD Uncharacterized protein OS=Gordonia phage Coeur OX=2571246 GN=3 PE=4 SV=1

---

66. 2::A0A4D6BFQ7 **Mass:** 32236 **Score:** 45 **Matches:** 1(1) **Sequences:** 1(1) **emPAI:** 0.14
>tr|A0A4D6BFQ7|A0A4D6BFQ7_9CAUD Major capsid protein OS=Streptococcus phage Javan570 OX=2548265 GN=Javan570_0040 PE=4 SV=1

Query	Observed	Mr(expt)	Mr(calc)	ppm	Miss	Score	Expect	Rank	Unique	Peptide
326	451.2584	900.5022	900.5029	-0.77	0	45	0.036	1	U	R.AVTAEAIAR.H

Proteins matching the same set of peptides:
2::D2IYW7 **Mass:** 36233 **Score:** 45 **Matches:** 1(1) **Sequences:** 1(1)
>tr|D2IYW7|D2IYW7_9CAUD Uncharacterized protein gp45 OS=Enterococcus phage phiFL1A OX=673832 GN=gp45 PE=4 SV=1
2::D2IZ30 **Mass:** 36233 **Score:** 45 **Matches:** 1(1) **Sequences:** 1(1)
>tr|D2IZ30|D2IZ30_9CAUD Uncharacterized protein gp47 OS=Enterococcus phage phiFL1B OX=673833 GN=gp47 PE=4 SV=1
2::D2IZ99 **Mass:** 36233 **Score:** 45 **Matches:** 1(1) **Sequences:** 1(1)
>tr|D2IZ99|D2IZ99_9CAUD Uncharacterized protein gp53 OS=Enterococcus phage phiFL1C OX=673834 GN=gp53 PE=4 SV=1
2::D2IZG2 **Mass:** 36233 **Score:** 45 **Matches:** 1(1) **Sequences:** 1(1)
>tr|D2IZG2|D2IZG2_9CAUD Uncharacterized protein gp42 OS=Enterococcus phage phiFL2A OX=673835 GN=gp42 PE=4 SV=1
2::D2IZM6 **Mass:** 36233 **Score:** 45 **Matches:** 1(1) **Sequences:** 1(1)
>tr|D2IZM6|D2IZM6_9CAUD Head protein OS=Enterococcus phage phiFL2B OX=673836 GN=gp43 PE=4 SV=1

---

67. 2::A0A3G8FE45 **Mass:** 32169 **Score:** 45 **Matches:** 1(1) **Sequences:** 1(1) **emPAI:** 0.14
>tr|A0A3G8FE45|A0A3G8FE45_9CAUD SPP1 gp7 family head morphogenesis protein OS=Phage NGI4 OX=2489188 PE=4 SV=1

Query	Observed	Mr(expt)	Mr(calc)	ppm	Miss	Score	Expect	Rank	Unique	Peptide
304	443.7531	885.4917	885.4920	-0.27	0	45	0.015	1	U	R.TEIPSALR.R

68. 2::A0A3S7SGD4 **Mass:** 7434 **Score:** 45 **Matches:** 1(1) **Sequences:** 1(1) **emPAI:** 0.72
>tr|A0A3S7SGD4|A0A3S7SGD4_9CAUD Uncharacterized protein OS=Aerococcus phage vB_AviM_AVP OX=2495576 GN=AVP_16 PE=4 SV=1

Query	Observed	Mr(expt)	Mr(calc)	ppm	Miss	Score	Expect	Rank	Unique	Peptide
627	503.2847	1004.5548	1004.5542	0.53	0	45	0.013	1	U	K.LDGLEFALK.T

69. 2::A0A0K0KVG1 **Mass:** 284825 **Score:** 44 **Matches:** 1(1) **Sequences:** 1(1) **emPAI:** 0.01
>tr|A0A0K0KVG1|A0A0K0KVG1_9CAUD Putative neck protein OS=Prochlorococcus phage P-TIM68 OX=1542477 PE=4 SV=1

Query	Observed	Mr(expt)	Mr(calc)	ppm	Miss	Score	Expect	Rank	Unique	Peptide
80	386.7217	771.4289	771.4279	1.33	0	44	0.015	1	U	K.FDIHIK.D

70. 2::A0A345MNE4 **Mass:** 73419 **Score:** 44 **Matches:** 1(1) **Sequences:** 1(1) **emPAI:** 0.06
>tr|A0A345MNE4|A0A345MNE4_9CAUD Helicase domain protein OS=Myoviridae sp. OX=2202564 PE=4 SV=1

Query	Observed	Mr(expt)	Mr(calc)	ppm	Miss	Score	Expect	Rank	Unique	Peptide
2050	687.3994	1372.7842	1372.7814	2.04	0	44	0.003	1	U	K.LISDVTGLTDIVK.V

71. 2::A0A0G2SSE8 **Mass:** 36305 **Score:** 44 **Matches:** 1(1) **Sequences:** 1(1) **emPAI:** 0.12
>tr|A0A0G2SSE8|A0A0G2SSE8_9CAUD Tail protein/adaptor protein OS=Proteus phage vB_PmiP_Pm5460 OX=1636249 GN=Pm5460_41 PE=4 SV=1

Query	Observed	Mr(expt)	Mr(calc)	ppm	Miss	Score	Expect	Rank	Unique	Peptide
909	544.2916	1086.5686	1086.5669	1.52	1	44	0.017	1	U	K.DAVNKGQLDK.V

72. 2::A0A0S1M392 **Mass:** 255098 **Score:** 44 **Matches:** 1(1) **Sequences:** 1(1) **emPAI:** 0.02
>tr|A0A0S1M392|A0A0S1M392_9PICO Genome polyprotein OS=Hedgehog hepatovirus OX=1745249 PE=4 SV=1

Query	Observed	Mr(expt)	Mr(calc)	ppm	Miss	Score	Expect	Rank	Unique	Peptide
5	352.6812	703.3479	703.3500	-3.03	0	44	0.039	1	U	K.SLNENK.I

Proteins matching the same set of peptides:
2::A0A0S1M3C0 **Mass:** 254956 **Score:** 44 **Matches:** 1(1) **Sequences:** 1(1)
>tr|A0A0S1M3C0|A0A0S1M3C0_9PICO Genome polyprotein OS=Hedgehog hepatovirus OX=1745249 PE=4 SV=1
2::A0A0S1M3C5 **Mass:** 255134 **Score:** 44 **Matches:** 1(1) **Sequences:** 1(1)
>tr|A0A0S1M3C5|A0A0S1M3C5_9PICO Genome polyprotein OS=Hedgehog hepatovirus Igel8Erieur2014 OX=1987144 PE=4 SV=1
2::A0A4V1F5G8 **Mass:** 8213 **Score:** 44 **Matches:** 1(1) **Sequences:** 1(1)
>tr|A0A4V1F5G8|A0A4V1F5G8_9VIRU Uncharacterized protein OS=Blackfly microvirus SF02 OX=2576452 PE=4 SV=1

73. 2::C8CJF1 **Mass:** 247398 **Score:** 44 **Matches:** 1(1) **Sequences:** 1(1) **emPAI:** 0.02
>tr|C8CJF1|C8CJF1_9RHAB RNA-directed RNA polymerase L OS=Drosophila obscura sigmavirus 10A OX=666962 GN=L PE=3 SV=1

Query	Observed	Mr(expt)	Mr(calc)	ppm	Miss	Score	Expect	Rank	Unique	Peptide
66	381.2192	760.4238	760.4232	0.77	0	44	0.028	1	U	K.QPTLFR.T

74. 2::A0A0F6R7E5 **Mass:** 12860 **Score:** 43 **Matches:** 1(1) **Sequences:** 1(1) **emPAI:** 0.38
>tr|A0A0F6R7E5|A0A0F6R7E5_9CAUD Uncharacterized protein OS=Escherichia coli O157 typing phage 8 OX=1508683 GN=ECTP8_01430 PE=4 SV=1

Query	Observed	Mr(expt)	Mr(calc)	ppm	Miss	Score	Expect	Rank	Unique	Peptide
98	393.7582	785.5019	785.5011	1.08	0	43	0.0097	1	U	K.IAAAVTIK.V

Proteins matching the same set of peptides:
2::A0A0F6TJL0 **Mass:** 12860 **Score:** 43 **Matches:** 1(1) **Sequences:** 1(1)
>tr|A0A0F6TJL0|A0A0F6TJL0_9CAUD Uncharacterized protein OS=Escherichia coli O157 typing phage 12 OX=1508674 GN=ECTP12_01880 PE=4 SV=1
2::A0A0F6TK27 **Mass:** 12860 **Score:** 43 **Matches:** 1(1) **Sequences:** 1(1)
>tr|A0A0F6TK27|A0A0F6TK27_9CAUD Uncharacterized protein OS=Escherichia coli O157 typing phage 11 OX=1508673 GN=ECTP11_01820 PE=4 SV=1
2::C5H7R7 **Mass:** 12860 **Score:** 43 **Matches:** 1(1) **Sequences:** 1(1)
>tr|C5H7R7|C5H7R7_9CAUD Uncharacterized protein 137 OS=Escherichia phage wV8 OX=576791 GN=137 PE=4 SV=1

75. 2::A0A385IRN7 **Mass:** 95529 **Score:** 43 **Matches:** 1(1) **Sequences:** 1(1) **emPAI:** 0.05
>tr|A0A385IRN7|A0A385IRN7_9CAUD Putative colanic acid-degrading protein OS=Escherichia phage vB_EcoM_IME392 OX=2301649 PE=4 SV=1

Query	Observed	Mr(expt)	Mr(calc)	ppm	Miss	Score	Expect	Rank	Unique	Peptide
48	374.2300	746.4455	746.4439	2.05	0	43	0.028	1	U	R.LFVGGVR.L

76. 2::A0A1Q1G599 **Mass:** 33933 **Score:** 43 **Matches:** 1(1) **Sequences:** 1(1) **emPAI:** 0.13
>tr|A0A1Q1G599|A0A1Q1G599_9HIV1 Pol protein (Fragment) OS=Human immunodeficiency virus 1 OX=11676 GN=pol PE=4 SV=1

Query	Observed	Mr(expt)	Mr(calc)	ppm	Miss	Score	Expect	Rank	Unique	Peptide
1064	561.3033	1120.5920	1120.5876	3.93	1	43	0.03	1	U	K.LFDSRELNK.R

77. 2::A0A1Z1LZM8 **Mass:** 28910 **Score:** 43 **Matches:** 1(1) **Sequences:** 1(1) **emPAI:** 0.16
>tr|A0A1Z1LZM8|A0A1Z1LZM8_9CAUD Uncharacterized protein OS=Bacillus phage Deep-Purple OX=1873341 GN=DeepPurple_gp035 PE=4 SV=1

Query	Observed	Mr(expt)	Mr(calc)	ppm	Miss	Score	Expect	Rank	Unique	Peptide
394	458.7765	915.5385	915.5389	-0.40	1	43	0.018	1	U	M.KNLETAIK.D

78. 2::A0A140G5X8 **Mass:** 41102 **Score:** 43 **Matches:** 1(1) **Sequences:** 1(1) **emPAI:** 0.11
>tr|A0A140G5X8|A0A140G5X8_9CAUD Putative scaffold protein OS=Enterobacteria phage ECGD1 OX=1784948 GN=ECGD1_130 PE=4 SV=1

Query	Observed	Mr(expt)	Mr(calc)	ppm	Miss	Score	Expect	Rank	Unique	Peptide
148	409.2050	816.3955	816.3977	-2.74	0	43	0.02	1	U	K.AEAEELR.K

Proteins matching the same set of peptides:
2::A0A291LB67 **Mass:** 41097 **Score:** 43 **Matches:** 1(1) **Sequences:** 1(1)
>tr|A0A291LB67|A0A291LB67_9CAUD Uncharacterized protein OS=Escherichia phage vB_EcoM_PHB05 OX=2041347 PE=4 SV=1
2::A0A2H4P7E5 **Mass:** 96818 **Score:** 43 **Matches:** 1(1) **Sequences:** 1(1)
>tr|A0A2H4P7E5|A0A2H4P7E5_9CAUD RIIA lysis inhibitor OS=Pseudomonas phage inbricus OX=2048976 GN=CNR35_00002 PE=4 SV=1
2::A0A385IES8 **Mass:** 41116 **Score:** 43 **Matches:** 1(1) **Sequences:** 1(1)
>tr|A0A385IES8|A0A385IES8_9CAUD Putative scaffold protein OS=Escherichia phage vB_vPM_PD114 OX=2316019 PE=4 SV=1
2::A0A386KIX3 **Mass:** 41116 **Score:** 43 **Matches:** 1(1) **Sequences:** 1(1)
>tr|A0A386KIX3|A0A386KIX3_9CAUD Putative scaffold protein OS=Escherichia phage vB_vPM_PD06 OX=2315527 PE=4 SV=1
2::A0A514CUL7 **Mass:** 96774 **Score:** 43 **Matches:** 1(1) **Sequences:** 1(1)
>tr|A0A514CUL7|A0A514CUL7_9CAUD Putative rIIA lysis inhibitor OS=Achromobacter phage vB_AxyP_19-32_Axy13 OX=2591044 GN=Axy13_034 PE=4 SV=1
2::I7HPG3 **Mass:** 41102 **Score:** 43 **Matches:** 1(1) **Sequences:** 1(1)
>tr|I7HPG3|I7HPG3_9CAUD Phi92_gp122 OS=Enterobacteria phage phi92 OX=948870 GN=PHI92_gene_122 PE=4 SV=1

79. 2::L0H229 **Mass:** 38097 **Score:** 42 **Matches:** 1(1) **Sequences:** 1(1) **emPAI:** 0.12
>tr|L0H229|L0H229_9HIV1 Envelope glycoprotein (Fragment) OS=Human immunodeficiency virus 1 OX=11676 GN=env PE=4 SV=1

Query	Observed	Mr(expt)	Mr(calc)	ppm	Miss	Score	Expect	Rank	Unique	Peptide
423	465.2639	928.5133	928.5130	0.22	0	42	0.051	1	U	R.WLEVVQR.T

Proteins matching the same set of peptides:
2::L0H3M9 **Mass:** 37274 **Score:** 42 **Matches:** 1(1) **Sequences:** 1(1)
>tr|L0H3M9|L0H3M9_9HIV1 Envelope glycoprotein (Fragment) OS=Human immunodeficiency virus 1 OX=11676 GN=env PE=4 SV=1

80. 2::E5E431 **Mass:** 16863 **Score:** 42 **Matches:** 1(1) **Sequences:** 1(1) **emPAI:** 0.28
>tr|E5E431|E5E431_9CAUD Uncharacterized protein OS=Acinetobacter phage Acj61 OX=760732 GN=Acj61p050 PE=4 SV=1

Query	Observed	Mr(expt)	Mr(calc)	ppm	Miss	Score	Expect	Rank	Unique	Peptide
140	408.7128	815.4110	815.4137	-3.27	0	42	0.016	1	U	R.AEAQLER.A

81. 2::A0A223LF67 **Mass:** 71904 **Score:** 42 **Matches:** 1(1) **Sequences:** 1(1) **emPAI:** 0.06
>tr|A0A223LF67|A0A223LF67_9CAUD Tail sheath protein OS=Aeromonas phage AS-gz OX=2026082 PE=4 SV=1

Query	Observed	Mr(expt)	Mr(calc)	ppm	Miss	Score	Expect	Rank	Unique	Peptide
206	423.2399	844.4652	844.4654	-0.28	1	42	0.037	1	U	K.VDADGKIK.A

Proteins matching the same set of peptides:
2::A0A2S1PEG1 **Mass:** 71953 **Score:** 42 **Matches:** 1(1) **Sequences:** 1(1)
>tr|A0A2S1PEG1|A0A2S1PEG1_9CAUD Tail sheath protein OS=Aeromonas phage 50AhydR13PP OX=2163978 PE=4 SV=1
2::A0A2S1PG39 **Mass:** 71904 **Score:** 42 **Matches:** 1(1) **Sequences:** 1(1)
>tr|A0A2S1PG39|A0A2S1PG39_9CAUD Tail sheath protein OS=Aeromonas phage 60AhydR15PP OX=2163979 PE=4 SV=1
2::E1A1G3 **Mass:** 28558 **Score:** 42 **Matches:** 1(1) **Sequences:** 1(1)
>tr|E1A1G3|E1A1G3_9CAUD Tail sheath monomer-like protein OS=Aeromonas phage phiAS4 OX=879628 GN=phiAS4_ORF0115 PE=4 SV=1
2::I6ZI77 **Mass:** 71966 **Score:** 42 **Matches:** 1(1) **Sequences:** 1(1)
>tr|I6ZI77|I6ZI77_9CAUD Tail sheath protein OS=Aeromonas phage Aes012 OX=1198014 GN=Aes012_153 PE=4 SV=1
2::J7HXR1 **Mass:** 71881 **Score:** 42 **Matches:** 1(1) **Sequences:** 1(1)
>tr|J7HXR1|J7HXR1_9CAUD Tail sheath monomer OS=Stenotrophomonas phage IME13 OX=1211280 PE=4 SV=1
2::J7KLQ6 **Mass:** 71908 **Score:** 42 **Matches:** 1(1) **Sequences:** 1(1)
>tr|J7KLQ6|J7KLQ6_9CAUD Tail sheath protein OS=Aeromonas phage Aes508 OX=1198013 GN=Aes508_142 PE=4 SV=1
2::Q19CM1 **Mass:** 71950 **Score:** 42 **Matches:** 1(1) **Sequences:** 1(1)
>tr|Q19CM1|Q19CM1_9CAUD Gp18 tail sheath monomer OS=Aeromonas virus 25 OX=233894 GN=18 PE=4 SV=1

---

82. 2::A0A5C1K3U7 **Mass:** 237367 **Score:** 42 **Matches:** 1(1) **Sequences:** 1(1) **emPAI:** 0.02
>tr|A0A5C1K3U7|A0A5C1K3U7_9MONO Large structural protein OS=Guadeloupe mosquito mononega-like virus OX=2607732 GN=orf6 PE=4 SV=1

Query	Observed	Mr(expt)	Mr(calc)	ppm	Miss	Score	Expect	Rank	Unique	Peptide
81	387.2110	772.4075	772.4079	-0.54	0	42	0.036	1	U	K.QLIDER.D

Proteins matching the same set of peptides:
2::H2A0C2 **Mass:** 14915 **Score:** 42 **Matches:** 1(1) **Sequences:** 1(1)
>tr|H2A0C2|H2A0C2_9CAUD Putative peptidase OS=Bacteroides phage B124-14 OX=1105171 GN=B124-14_017 PE=4 SV=1
2::R4ZDA1 **Mass:** 14683 **Score:** 42 **Matches:** 1(1) **Sequences:** 1(1)
>tr|R4ZDA1|R4ZDA1_9POXV Uncharacterized protein OS=Adoxophyes honmai entomopoxvirus 'L' OX=1293540 GN=AHEV_074 PE=4 SV=1
2::K9ME25 **Mass:** 18785 **Score:** 42 **Matches:** 1(1) **Sequences:** 1(1)
>tr|K9ME25|K9ME25_9VIRU Uncharacterized protein OS=Sulfolobus virus STSV2 OX=1123964 GN=STSV2_01 PE=4 SV=1

---

83. 2::V9VL70 **Mass:** 20919 **Score:** 41 **Matches:** 1(0) **Sequences:** 1(0)
>tr|V9VL70|V9VL70_9GAMC Nucleocapsid protein (Fragment) OS=Infectious bronchitis virus OX=11120 GN=N PE=4 SV=1

Query	Observed	Mr(expt)	Mr(calc)	ppm	Miss	Score	Expect	Rank	Unique	Peptide
330	451.7558	901.4970	901.4981	-1.28	0	41	0.086	1	U	K.QGLLAASSR.A

---

84. 2::Q27VW2 **Mass:** 15466 **Score:** 41 **Matches:** 1(1) **Sequences:** 1(1) **emPAI:** 0.31
>tr|Q27VW2|Q27VW2_9HIV1 Envelope glycoprotein (Fragment) OS=Human immunodeficiency virus 1 OX=11676 GN=env PE=4 SV=1

Query	Observed	Mr(expt)	Mr(calc)	ppm	Miss	Score	Expect	Rank	Unique	Peptide
153	409.7064	817.3982	817.3970	1.47	1	41	0.0096	1	U	R.EELYKH.-

---

85. 2::A0A1U8ZS54 **Mass:** 63370 **Score:** 41 **Matches:** 1(1) **Sequences:** 1(1) **emPAI:** 0.07
>tr|A0A1U8ZS54|A0A1U8ZS54_9VIRU Nucleoprotein OS=Lassa mammarenavirus OX=11620 GN=N PE=3 SV=1

Query	Observed	Mr(expt)	Mr(calc)	ppm	Miss	Score	Expect	Rank	Unique	Peptide
391	458.2796	914.5446	914.5437	1.01	0	41	0.02	1	U	K.LIDISLNK.A

Proteins matching the same set of peptides:
2::A0A2P1JNE0 **Mass:** 63292 **Score:** 41 **Matches:** 1(1) **Sequences:** 1(1)
>tr|A0A2P1JNE0|A0A2P1JNE0_9VIRU Nucleoprotein OS=Lassa mammarenavirus OX=11620 GN=NP PE=3 SV=1
2::A0A2P1JNH3 **Mass:** 63353 **Score:** 41 **Matches:** 1(1) **Sequences:** 1(1)
>tr|A0A2P1JNH3|A0A2P1JNH3_9VIRU Nucleoprotein OS=Lassa mammarenavirus OX=11620 GN=NP PE=3 SV=1
2::A0A2P1JNQ6 **Mass:** 63298 **Score:** 41 **Matches:** 1(1) **Sequences:** 1(1)
>tr|A0A2P1JNQ6|A0A2P1JNQ6_9VIRU Nucleoprotein (Fragment) OS=Lassa mammarenavirus OX=11620 GN=NP PE=3 SV=1
2::A0A3G2CGT0 **Mass:** 63298 **Score:** 41 **Matches:** 1(1) **Sequences:** 1(1)
>tr|A0A3G2CGT0|A0A3G2CGT0_9VIRU Nucleoprotein OS=Lassa mammarenavirus OX=11620 GN=NP PE=3 SV=1
2::A0A3Q8QFI5 **Mass:** 63362 **Score:** 41 **Matches:** 1(1) **Sequences:** 1(1)
>tr|A0A3Q8QFI5|A0A3Q8QFI5_9VIRU Nucleoprotein OS=Lassa mammarenavirus OX=11620 GN=NP PE=3 SV=1
2::A0A3Q8QFJ2 **Mass:** 63381 **Score:** 41 **Matches:** 1(1) **Sequences:** 1(1)
>tr|A0A3Q8QFJ2|A0A3Q8QFJ2_9VIRU Nucleoprotein OS=Lassa mammarenavirus OX=11620 GN=NP PE=3 SV=1
2::A0A3Q8QFM0 **Mass:** 63332 **Score:** 41 **Matches:** 1(1) **Sequences:** 1(1)
>tr|A0A3Q8QFM0|A0A3Q8QFM0_9VIRU Nucleoprotein OS=Lassa mammarenavirus OX=11620 GN=NP PE=3 SV=1
2::A0A3S7SYW1 **Mass:** 63409 **Score:** 41 **Matches:** 1(1) **Sequences:** 1(1)
>tr|A0A3S7SYW1|A0A3S7SYW1_9VIRU Nucleoprotein OS=Lassa mammarenavirus OX=11620 GN=N PE=3 SV=1
2::A0A3S8NVJ6 **Mass:** 63331 **Score:** 41 **Matches:** 1(1) **Sequences:** 1(1)
>tr|A0A3S8NVJ6|A0A3S8NVJ6_9VIRU Nucleoprotein OS=Lassa mammarenavirus OX=11620 GN=NP PE=3 SV=1
2::A0A3S8NW48 **Mass:** 57311 **Score:** 41 **Matches:** 1(1) **Sequences:** 1(1)
>tr|A0A3S8NW48|A0A3S8NW48_9VIRU Nucleoprotein OS=Lassa mammarenavirus OX=11620 GN=NP PE=4 SV=1
2::A0A3S8NW88 **Mass:** 63369 **Score:** 41 **Matches:** 1(1) **Sequences:** 1(1)
>tr|A0A3S8NW88|A0A3S8NW88_9VIRU Nucleoprotein OS=Lassa mammarenavirus OX=11620 GN=NP PE=3 SV=1
2::A0A3S8NWD2 **Mass:** 63392 **Score:** 41 **Matches:** 1(1) **Sequences:** 1(1)
>tr|A0A3S8NWD2|A0A3S8NWD2_9VIRU Nucleoprotein OS=Lassa mammarenavirus OX=11620 GN=NP PE=3 SV=1
2::A0A3S8NWH4 **Mass:** 63229 **Score:** 41 **Matches:** 1(1) **Sequences:** 1(1)
>tr|A0A3S8NWH4|A0A3S8NWH4_9VIRU Nucleoprotein OS=Lassa mammarenavirus OX=11620 GN=NP PE=3 SV=1
2::U5KR76 **Mass:** 62585 **Score:** 41 **Matches:** 1(1) **Sequences:** 1(1)
>tr|U5KR76|U5KR76_9VIRU Nucleoprotein (Fragment) OS=Lassa mammarenavirus OX=11620 PE=3 SV=1
2::Q6GWR5 **Mass:** 63302 **Score:** 41 **Matches:** 1(1) **Sequences:** 1(1)
>tr|Q6GWR5|Q6GWR5_9VIRU Nucleoprotein OS=Lassa mammarenavirus OX=11620 GN=N PE=3 SV=1
2::E9K9R3 **Mass:** 63303 **Score:** 41 **Matches:** 1(1) **Sequences:** 1(1)
>tr|E9K9R3|E9K9R3_9VIRU Nucleoprotein OS=Lassa mammarenavirus OX=11620 GN=NP PE=3 SV=1
2::Q9IMH6 **Mass:** 23521 **Score:** 41 **Matches:** 1(1) **Sequences:** 1(1)
>tr|Q9IMH6|Q9IMH6_9VIRU Nucleoprotein (Fragment) OS=Lassa mammarenavirus OX=11620 PE=4 SV=1
2::Q9IMH7 **Mass:** 23497 **Score:** 41 **Matches:** 1(1) **Sequences:** 1(1)
>tr|Q9IMH7|Q9IMH7_9VIRU Nucleoprotein (Fragment) OS=Lassa mammarenavirus OX=11620 PE=4 SV=1
2::Q9IMH8 **Mass:** 23536 **Score:** 41 **Matches:** 1(1) **Sequences:** 1(1)
>tr|Q9IMH8|Q9IMH8_9VIRU Nucleoprotein (Fragment) OS=Lassa mammarenavirus OX=11620 PE=4 SV=1
2::Q9IMH9 **Mass:** 23628 **Score:** 41 **Matches:** 1(1) **Sequences:** 1(1)
>tr|Q9IMH9|Q9IMH9_9VIRU Nucleoprotein (Fragment) OS=Lassa mammarenavirus OX=11620 PE=4 SV=1
2::Q9IMI0 **Mass:** 23612 **Score:** 41 **Matches:** 1(1) **Sequences:** 1(1)
>tr|Q9IMI0|Q9IMI0_9VIRU Nucleoprotein (Fragment) OS=Lassa mammarenavirus OX=11620 PE=4 SV=1
2::Q9IMI1 **Mass:** 23487 **Score:** 41 **Matches:** 1(1) **Sequences:** 1(1)
>tr|Q9IMI1|Q9IMI1_9VIRU Nucleoprotein (Fragment) OS=Lassa mammarenavirus OX=11620 PE=4 SV=1
2::Q9IMI2 **Mass:** 23487 **Score:** 41 **Matches:** 1(1) **Sequences:** 1(1)
>tr|Q9IMI2|Q9IMI2_9VIRU Nucleoprotein (Fragment) OS=Lassa mammarenavirus OX=11620 PE=4 SV=1

---

86. 2::A0A516LSU6 **Mass:** 55218 **Score:** 41 **Matches:** 1(1) **Sequences:** 1(1) **emPAI:** 0.08
>tr|A0A516LSU6|A0A516LSU6_9VIRU Putative 60 kDa chaperonin OS=Prokaryotic dsDNA virus sp. OX=2591644 GN=GOVbin1782_22 PE=4 SV=1

Query	Observed	Mr(expt)	Mr(calc)	ppm	Miss	Score	Expect	Rank	Unique	Peptide
335	451.7733	901.5321	901.5345	-2.67	1	41	0.012	1	U	R.KITSINAR.T

---

87. 2::A0A1D6Y7D3 **Mass:** 87661 **Score:** 41 **Matches:** 1(1) **Sequences:** 1(1) **emPAI:** 0.05
>tr|A0A1D6Y7D3|A0A1D6Y7D3_9VIRU Putative mRNA-capping enzyme OS=Golden Marseillevirus OX=1720526 GN=GMAR_ORF208 PE=4 SV=1

Query	Observed	Mr(expt)	Mr(calc)	ppm	Miss	Score	Expect	Rank	Unique	Peptide
668	509.7624	1017.5102	1017.5131	-2.88	0	41	0.054	1	U	K.IEDLFEPR.L

88. 2::A0A2H4UVE2 **Mass:** 39272 **Score:** 41 **Matches:** 1(1) **Sequences:** 1(1) **emPAI:** 0.11
>tr|A0A2H4UVE2|A0A2H4UVE2_9VIRU Serine/threonine-protein kinase OS=Bodo saltans virus OX=2024608 GN=BMW23_0847 PE=4 SV=1

Query	Observed	Mr(expt)	Mr(calc)	ppm	Miss	Score	Expect	Rank	Unique	Peptide
572	493.7980	985.5815	985.5808	0.73	0	41	0.034	1	U	K.ITNDIVALK.T

89. 2::A0A0R6PJK4 **Mass:** 176372 **Score:** 40 **Matches:** 1(1) **Sequences:** 1(1) **emPAI:** 0.02
>tr|A0A0R6PJK4|A0A0R6PJK4_9CAUD Haemagg_act domain-containing protein OS=Moraxella phage Mcat25 OX=1647541 PE=4 SV=1

Query	Observed	Mr(expt)	Mr(calc)	ppm	Miss	Score	Expect	Rank	Unique	Peptide
1942	672.3630	1342.7114	1342.7133	-1.37	0	40	0.03	1	U	K.IEAIVFDNPTPK.N

90. 2::A0A482MH24 **Mass:** 26615 **Score:** 40 **Matches:** 1(1) **Sequences:** 1(1) **emPAI:** 0.17
>tr|A0A482MH24|A0A482MH24_9CAUD Uncharacterized protein OS=Serratia phage Serbin OX=2562181 GN=CPT_Serbin_039 PE=4 SV=1

Query	Observed	Mr(expt)	Mr(calc)	ppm	Miss	Score	Expect	Rank	Unique	Peptide
15	360.6873	719.3600	719.3602	-0.38	0	40	0.013	1	U	K.YGVEPR.I

91. 2::A0A1U7ET45 **Mass:** 127431 **Score:** 40 **Matches:** 2(2) **Sequences:** 1(1) **emPAI:** 0.03
>tr|A0A1U7ET45|A0A1U7ET45_9VIRU 126 kDa protein OS=Odontoglossum ringspot virus OX=12238 PE=4 SV=1

Query	Observed	Mr(expt)	Mr(calc)	ppm	Miss	Score	Expect	Rank	Unique	Peptide
466	472.2770	942.5394	942.5386	0.89	0	40	0.058	1	U	K.TLDSGLLPK.Q 465

Proteins matching the same set of peptides:
2::A0A1U7ET49 **Mass:** 184952 **Score:** 40 **Matches:** 2(2) **Sequences:** 1(1)
>tr|A0A1U7ET49|A0A1U7ET49_9VIRU 183 kDa readthrough protein OS=Odontoglossum ringspot virus OX=12238 PE=4 SV=1
2::A0A2Z6E953 **Mass:** 127576 **Score:** 40 **Matches:** 2(2) **Sequences:** 1(1)
>tr|A0A2Z6E953|A0A2Z6E953_9VIRU 126 kDa protein OS=Odontoglossum ringspot virus OX=12238 PE=4 SV=1
2::A0A2Z6E9A3 **Mass:** 185097 **Score:** 40 **Matches:** 2(2) **Sequences:** 1(1)
>tr|A0A2Z6E9A3|A0A2Z6E9A3_9VIRU 183 kDa readthrough protein OS=Odontoglossum ringspot virus OX=12238 PE=4 SV=1
2::Q3YFN0 **Mass:** 127551 **Score:** 40 **Matches:** 2(2) **Sequences:** 1(1)
>tr|Q3YFN0|Q3YFN0_9VIRU RNA-dependent RNA polymerase/replicase OS=Odontoglossum ringspot virus OX=12238 GN=RdRp PE=4 SV=1
2::Q3YFN1 **Mass:** 185692 **Score:** 40 **Matches:** 2(2) **Sequences:** 1(1)
>tr|Q3YFN1|Q3YFN1_9VIRU RdRp readthrough protein OS=Odontoglossum ringspot virus OX=12238 PE=4 SV=1
2::W5RYZ3 **Mass:** 127577 **Score:** 40 **Matches:** 2(2) **Sequences:** 1(1)
>tr|W5RYZ3|W5RYZ3_9VIRU 129 kDa replicase protein OS=Odontoglossum ringspot virus OX=12238 PE=4 SV=1
2::W5S2L7 **Mass:** 183302 **Score:** 40 **Matches:** 2(2) **Sequences:** 1(1)
>tr|W5S2L7|W5S2L7_9VIRU 183 kDa replicase protein OS=Odontoglossum ringspot virus OX=12238 PE=4 SV=1
2::Q6PY88 **Mass:** 127535 **Score:** 40 **Matches:** 2(2) **Sequences:** 1(1)
>tr|Q6PY88|Q6PY88_9VIRU RNA-dependent RNA polymerase/replicase OS=Odontoglossum ringspot virus OX=12238 GN=RdRp PE=4 SV=1
2::Q6PY89 **Mass:** 185056 **Score:** 40 **Matches:** 2(2) **Sequences:** 1(1)
>tr|Q6PY89|Q6PY89_9VIRU RNA-dependent RNA polymerase readthrough protein OS=Odontoglossum ringspot virus OX=12238 GN=RdRp PE=4 SV=1
2::O10447 **Mass:** 185188 **Score:** 40 **Matches:** 2(2) **Sequences:** 1(1)
>tr|O10447|O10447_9VIRU Polyprotein OS=Odontoglossum ringspot virus OX=12238 PE=4 SV=1
2::O39641 **Mass:** 127523 **Score:** 40 **Matches:** 2(2) **Sequences:** 1(1)
>tr|O39641|O39641_9VIRU 130k protein OS=Odontoglossum ringspot virus OX=12238 PE=4 SV=2
2::Q84124 **Mass:** 127662 **Score:** 40 **Matches:** 2(2) **Sequences:** 1(1)
>tr|Q84124|Q84124_9VIRU 126kDa replicase OS=Odontoglossum ringspot virus OX=12238 PE=4 SV=1
2::Q84125 **Mass:** 183401 **Score:** 40 **Matches:** 2(2) **Sequences:** 1(1)
>tr|Q84125|Q84125_9VIRU 181 kDa readthrough protein OS=Odontoglossum ringspot virus OX=12238 PE=4 SV=1
1::Q84133 **Mass:** 185075 **Score:** 40 **Matches:** 2(2) **Sequences:** 1(1)
Replicase large subunit OS=Odontoglossum ringspot virus (isolate Singapore 1) OX=138662 PE=3 SV=2
2::Q84134 **Mass:** 127783 **Score:** 40 **Matches:** 2(2) **Sequences:** 1(1)
>tr|Q84134|Q84134_ORSVS 126 kDa replicase OS=Odontoglossum ringspot virus (isolate Singapore 1) OX=138662 PE=4 SV=1
1::P89659 **Mass:** 185133 **Score:** 40 **Matches:** 2(2) **Sequences:** 1(1)
Replicase large subunit OS=Odontoglossum ringspot virus (isolate Korean Cy) OX=138661 PE=3 SV=2
2::P90348 **Mass:** 127392 **Score:** 40 **Matches:** 2(2) **Sequences:** 1(1)
>tr|P90348|P90348_ORSVC 126 kDa protein (Fragment) OS=Odontoglossum ringspot virus (isolate Korean Cy) OX=138661 PE=4 SV=1

92. 2::A0A0U2UDM8 **Mass:** 57459 **Score:** 40 **Matches:** 1(1) **Sequences:** 1(1) **emPAI:** 0.08
>tr|A0A0U2UDM8|A0A0U2UDM8_9HIV1 Pol protein (Fragment) OS=Human immunodeficiency virus 1 OX=11676 PE=3 SV=1

Query	Observed	Mr(expt)	Mr(calc)	ppm	Miss	Score	Expect	Rank	Unique	Peptide
461	471.7713	941.5280	941.5294	-1.47	0	40	0.015	1	U	K.QLVEAVQR.I

Proteins matching the same set of peptides:
2::A0A1L4CTP9 **Mass:** 60997 **Score:** 40 **Matches:** 1(1) **Sequences:** 1(1)
>tr|A0A1L4CTP9|A0A1L4CTP9_9HIV1 Pol protein (Fragment) OS=Human immunodeficiency virus 1 OX=11676 GN=pol PE=3 SV=1
2::A0A1X9G6Y9 **Mass:** 113111 **Score:** 40 **Matches:** 1(1) **Sequences:** 1(1)
>tr|A0A1X9G6Y9|A0A1X9G6Y9_9HIV1 Pol protein (Fragment) OS=Human immunodeficiency virus 1 OX=11676 GN=pol PE=3 SV=1
2::A0A345VTV1 **Mass:** 57215 **Score:** 40 **Matches:** 1(1) **Sequences:** 1(1)
>tr|A0A345VTV1|A0A345VTV1_9HIV1 Pol protein (Fragment) OS=Human immunodeficiency virus 1 OX=11676 GN=pol PE=3 SV=1
2::A0F896 **Mass:** 57637 **Score:** 40 **Matches:** 1(1) **Sequences:** 1(1)
>tr|A0F896|A0F896_9HIV1 Pol protein (Fragment) OS=Human immunodeficiency virus 1 OX=11676 GN=pol PE=3 SV=1
2::A0F897 **Mass:** 57703 **Score:** 40 **Matches:** 1(1) **Sequences:** 1(1)
>tr|A0F897|A0F897_9HIV1 Pol protein (Fragment) OS=Human immunodeficiency virus 1 OX=11676 GN=pol PE=3 SV=1
2::L0AT58 **Mass:** 114387 **Score:** 40 **Matches:** 1(1) **Sequences:** 1(1)
>tr|L0AT58|L0AT58_9HIV1 Pol protein (Fragment) OS=Human immunodeficiency virus 1 OX=11676 GN=pol PE=3 SV=1
2::D3VX84 **Mass:** 97114 **Score:** 40 **Matches:** 1(1) **Sequences:** 1(1)
>tr|D3VX84|D3VX84_9HIV1 Pol protein (Fragment) OS=Human immunodeficiency virus 1 OX=11676 GN=pol PE=4 SV=1
2::G5CFC5 **Mass:** 57199 **Score:** 40 **Matches:** 1(1) **Sequences:** 1(1)
>tr|G5CFC5|G5CFC5_9HIV1 Pol protein (Fragment) OS=Human immunodeficiency virus 1 OX=11676 GN=pol PE=3 SV=1
2::G5DH53 **Mass:** 57287 **Score:** 40 **Matches:** 1(1) **Sequences:** 1(1)
>tr|G5DH53|G5DH53_9HIV1 Pol protein (Fragment) OS=Human immunodeficiency virus 1 OX=11676 GN=pol PE=3 SV=1
2::G5DL30 **Mass:** 57149 **Score:** 40 **Matches:** 1(1) **Sequences:** 1(1)
>tr|G5DL30|G5DL30_9HIV1 Pol protein (Fragment) OS=Human immunodeficiency virus 1 OX=11676 GN=pol PE=3 SV=1
2::I7F9K8 **Mass:** 57238 **Score:** 40 **Matches:** 1(1) **Sequences:** 1(1)
>tr|I7F9K8|I7F9K8_9HIV1 Pol protein (Fragment) OS=Human immunodeficiency virus 1 OX=11676 GN=pol PE=3 SV=1
2::Q08JX9 **Mass:** 113721 **Score:** 40 **Matches:** 1(1) **Sequences:** 1(1)
>tr|Q08JX9|Q08JX9_9HIV1 Pol (Fragment) OS=Human immunodeficiency virus 1 OX=11676 GN=pol PE=3 SV=1
2::Q08JY8 **Mass:** 113724 **Score:** 40 **Matches:** 1(1) **Sequences:** 1(1)
>tr|Q08JY8|Q08JY8_9HIV1 Pol (Fragment) OS=Human immunodeficiency virus 1 OX=11676 GN=pol PE=3 SV=1
2::B9V5X4 **Mass:** 114226 **Score:** 40 **Matches:** 1(1) **Sequences:** 1(1)
>tr|B9V5X4|B9V5X4_9HIV1 Pol protein (Fragment) OS=Human immunodeficiency virus 1 OX=11676 GN=pol PE=3 SV=1

Mascot: http://www.matrixscience.com/

MATRIX SCIENCE Mascot Search Results

User	: Asia Zebrowska
Email	: asia.zebrowska87@gmail.com
Search title	: Copy of Zebrowska cal viruses sheep
MS data file	: \\212.87.29.243\Dane\USERS\Agata\tmp\00229316zebr_BL_depol-Q8.mgf
Database 1	: Sprot 2020_01 (561911 sequences; 202173710 residues)
Taxonomy 1	: Viruses (16712 sequences)
Database 2	: Trembl 2019_11 (179250561 sequences; 60319985647 residues)
Taxonomy 2	: Viruses (4300387 sequences)
Timestamp	: 11 Mar 2020 at 14:30:56 GMT
Enzyme	: Trypsin
Fixed modifications	: Carbamidomethyl (C)
Variable modifications	: Oxidation (M)
Mass values	: Monoisotopic
Protein Mass	: Unrestricted
Peptide Mass Tolerance	: ± 5 ppm
Fragment Mass Tolerance	: ± 0.01 Da
Max Missed Cleavages	: 1
Instrument type	: HCD
Number of queries	: 5242
Protein hits	: 2::A0A1U9WQS0 >tr|A0A1U9WQS0|A0A1U9WQS0_9CAUD Glycosylase OS=Geobacillus phage TP-84 OX=1965361 PE=4 SV=1
	2::A0A1U9WQR1 >tr|A0A1U9WQR1|A0A1U9WQR1_9CAUD Tail protein OS=Geobacillus phage TP-84 OX=1965361 PE=4 SV=1
	2::A0A1U9WQN1 >tr|A0A1U9WQN1|A0A1U9WQN1_9CAUD Thymidylate synthase OS=Geobacillus phage TP-84 OX=1965361 PE=4 SV=1
	2::A0A076FL24 >tr|A0A076FL24|A0A076FL24_9MONO Enhanced green fluorescent protein OS=Measles morbillivirus MeV-eGFP_Edm-tag OX=1524961 GN=eGFP PE=4 SV=1
	2::W6LP63 >tr|W6LP63|W6LP63_9CAUD Enolase OS=Streptococcus phage 20617 OX=1392231 GN=eno PE=3 SV=1
	2::A0A0M4R3J1 >tr|A0A0M4R3J1|A0A0M4R3J1_9CAUD Uncharacterized protein OS=Arthrobacter phage Brent OX=1701798 GN=SEA_BRENT_43 PE=4 SV=1
	2::A0A1U9WQP5 >tr|A0A1U9WQP5|A0A1U9WQP5_9CAUD Capsid portal protein OS=Geobacillus phage TP-84 OX=1965361 PE=4 SV=1
	2::Q66NJ1 >tr|Q66NJ1|Q66NJ1_9HIV1 Reverse transcriptase (Fragment) OS=Human immunodeficiency virus 1 OX=11676 GN=pol PE=4 SV=1
	2::Q6R4V1 >tr|Q6R4V1|Q6R4V1_9CAUD Putative replication protein RepA OS=Vibrio virus VP882 OX=261726 PE=4 SV=1
	2::A0A059UHZ7 >tr|A0A059UHZ7|A0A059UHZ7_9RETR Pol protein (Fragment) OS=Feline immunodeficiency virus OX=11673 GN=pol PE=3 SV=1
	2::A0A0M3SUP6 >tr|A0A0M3SUP6|A0A0M3SUP6_9VIRU RNA-dependent RNA polymerase (Fragment) OS=Rhizoctonia solani beny-like virus 1 OX=1708385 PE=4 SV=1
	2::A0A0M5M5R9 >tr|A0A0M5M5R9|A0A0M5M5R9_9CAUD SsDNA-binding protein OS=Citrobacter phage Margaery OX=1701810 GN=CPT_Margaery222 PE=4 SV=1
	2::A0A0E4G1G1 >tr|A0A0E4G1G1|A0A0E4G1G1_9CAUD Putative phage structural protein OS=Staphylococcus phage PSa3 OX=1319980 GN=PSa3_ORF15c PE=4 SV=1
	2::A0A223W084 >tr|A0A223W084|A0A223W084_9CAUD Uncharacterized protein OS=Agrobacterium phage Atu_ph08 OX=2024265 PE=4 SV=1
	2::A0A3S7SGD4 >tr|A0A3S7SGD4|A0A3S7SGD4_9CAUD Uncharacterized protein OS=Aerococcus phage vB_AviM_AVP OX=2495576 GN=AVP_16 PE=4 SV=1
	2::A0A0K1LLW6 >tr|A0A0K1LLW6|A0A0K1LLW6_9CAUD Uncharacterized protein OS=Caulobacter phage Sansa OX=1675600 GN=CPT_Sansa92 PE=4 SV=1
	2::C0M298 >tr|C0M298|C0M298_BPMS2 Replicase protein (Fragment) OS=Enterobacteria phage MS2 OX=12022 GN=MS2g4 PE=4 SV=1
	2::A0A097BY06 >tr|A0A097BY06|A0A097BY06_9CAUD Minor tail protein OS=Mycobacterium phage Power OX=1552733 GN=PBI_POWER_31 PE=4 SV=1
	2::A0A1V0DZB4 >tr|A0A1V0DZB4|A0A1V0DZB4_9CAUD Replicative DNA helicase OS=Acinetobacter phage WCHABP12 OX=1965454 GN=dnaB PE=4 SV=1
	2::A0A482MLD2 >tr|A0A482MLD2|A0A482MLD2_9CAUD Uncharacterized protein OS=Caudovirales GX15bay OX=2530034 PE=4 SV=1
	2::A0A141HS99 >tr|A0A141HS99|A0A141HS99_9CAUD Uncharacterized protein OS=Bacillus phage vB_BpuM-BpSp OX=1739968 GN=Bp8pS_208 PE=4 SV=1
	2::A0A3G5A6F4 >tr|A0A3G5A6F4|A0A3G5A6F4_9VIRU ANK_REP_REGION domain-containing protein OS=Hyperionvirus sp. OX=2487770 GN=Hyperionvirus2_195 PE=4 SV=1
	2::A0A0A0YVX1 >tr|A0A0A0YVX1|A0A0A0YVX1_9CAUD Uncharacterized protein OS=Pseudomonas phage YH6 OX=1566995 GN=YH6_053 PE=4 SV=1
	2::A0A068EKL1 >tr|A0A068EKL1|A0A068EKL1_9POXV Ankyrin repeat protein OS=Pigeonpox virus OX=10264 GN=fep_016 PE=4 SV=1
	2::A0A060D5Q0 >tr|A0A060D5Q0|A0A060D5Q0_9CAUD Uncharacterized protein OS=Salmonella phage 9NA OX=1113547 GN=9NA_029 PE=4 SV=1
	2::A0A516L0H2 >tr|A0A516L0H2|A0A516L0H2_9VIRU Uncharacterized protein OS=Prokaryotic dsDNA virus sp. OX=2591644 GN=GOVbin2950_25 PE=4 SV=1
	2::A0A290FZR5 >tr|A0A290FZR5|A0A290FZR5_9CAUD Uncharacterized protein OS=Caldibacillus phage CBP1 OX=2036768 PE=4 SV=1
	2::A0A023T3C3 >tr|A0A023T3C3|A0A023T3C3_9POTV Polyprotein (Fragment) OS=Zucchini yellow mosaic virus OX=12232 PE=3 SV=1
	2::A0A1V0SKV6 >tr|A0A1V0SKV6|A0A1V0SKV6_9VIRU Uncharacterized protein OS=Klosneuvirus KNV1 OX=1977640 GN=Klosneuvirus_5_30 PE=4 SV=1
	2::D7SGS7 >tr|D7SGS7|D7SGS7_9MONO Polymerase (Fragment) OS=Paramyxovirus bat/GH47/2008 OX=589524 GN=L PE=4 SV=1
	2::A0A160DAA3 >tr|A0A160DAA3|A0A160DAA3_9ALPC Spike glycoprotein OS=Porcine epidemic diarrhea virus OX=28295 GN=S PE=2 SV=1
	2::A0A0A0YX30 >tr|A0A0A0YX30|A0A0A0YX30_9CAUD Integrase OS=Streptococcus phage SpSL1 OX=1566990 GN=int PE=4 SV=1
	2::A0A482JV29 >tr|A0A482JV29|A0A482JV29_9RHAB RNA-directed RNA polymerase L OS=Lampyris noctiluca rhabdo-like virus 1 OX=2552994 PE=3 SV=1
	2::A0A076JU09 >tr|A0A076JU09|A0A076JU09_9ALPH Portal protein OS=Equid alphaherpesvirus 1 OX=10326 PE=3 SV=1
	2::A8ATK7 >tr|A8ATK7|A8ATK7_9CAUD Gp49 OS=Listeria phage B054 OX=330397 PE=4 SV=1
	2::A0A2I7QYQ0 >tr|A0A2I7QYQ0|A0A2I7QYQ0_9CAUD Uncharacterized protein OS=Vibrio phage 1.086.O._10N.222.51.F8 OX=1881424 GN=NVP1086O_01 PE=4 SV=1
	2::A0A2Z2CKJ5 >tr|A0A2Z2CKJ5|A0A2Z2CKJ5_9VIRU Glycoprotein OS=Pidgey virus OX=1911436 PE=4 SV=1
	2::A0A0K0KVG1 >tr|A0A0K0KVG1|A0A0K0KVG1_9CAUD Putative neck protein OS=Prochlorococcus phage P-TIM68 OX=1542477 PE=4 SV=1
	2::A0A1D8MBQ4 >tr|A0A1D8MBQ4|A0A1D8MBQ4_9VIRU Polyprotein (Fragment) OS=Endornavirus-like virus OX=1906952 PE=4 SV=1
	2::A0A481ZC74 >tr|A0A481ZC74|A0A481ZC74_9VIRU Uncharacterized protein OS=Pithovirus LCPAC401 OX=2506595 GN=LCPAC401_03270 PE=4 SV=1
	2::A0A3G1QTM4 >tr|A0A3G1QTM4|A0A3G1QTM4_9CAUD Virion-associated RNA polymerase OS=Erwinia phage phiEaP-8 OX=2178928 PE=4 SV=1
	2::A0A2I6BQV1 >tr|A0A2I6BQV1|A0A2I6BQV1_9POXV Uncharacterized protein OS=White-tailed deer poxvirus OX=2067573 GN=DPV106 PE=4 SV=1
	2::A0A0G3Y4Q4 >tr|A0A0G3Y4Q4|A0A0G3Y4Q4_OSHV1 ORF67 OS=Ostreid herpesvirus 1 OX=261939 PE=4 SV=1
	2::A0A0F6NAG1 >tr|A0A0F6NAG1|A0A0F6NAG1_9POTV Helper component protein (Fragment) OS=Banana bract mosaic virus OX=45661 PE=4 SV=1
	2::A0A385IRN7 >tr|A0A385IRN7|A0A385IRN7_9CAUD Putative colanic acid-degrading protein OS=Escherichia phage vB_EcoM_IME392 OX=2301649 PE=4 SV=1
	2::A0A2D2W767 >tr|A0A2D2W767|A0A2D2W767_9CAUD Uncharacterized protein OS=Pectobacterium phage DU_PP_V OX=2041492 GN=P13BB106kb_p046 PE=4 SV=1
	2::A0A1W6QMP8 >tr|A0A1W6QMP8|A0A1W6QMP8_9INFB Polymerase basic protein 2 OS=Influenza B virus (B/Minnesota/05/2017) OX=1985038 GN=PB2 PE=3 SV=1
	2::A0A0F6R7E5 >tr|A0A0F6R7E5|A0A0F6R7E5_9CAUD Uncharacterized protein OS=Escherichia coli O157 typing phage 8 OX=1508683 GN=ECTP8_01430 PE=4 SV=1
	2::A0A0N6YRF3 >tr|A0A0N6YRF3|A0A0N6YRF3_9CAUD Uncharacterized protein OS=Dickeya phage phiD3 OX=1542131 GN=HQ80_0150 PE=4 SV=1
	2::A0A1L3KJF4 >tr|A0A1L3KJF4|A0A1L3KJF4_9VIRU RdRp OS=Beihai hepe-like virus 2 OX=1922383 PE=4 SV=1
	2::A0A345MNE4 >tr|A0A345MNE4|A0A345MNE4_9CAUD Helicase domain protein OS=Myoviridae sp. OX=2202564 PE=4 SV=1
	2::A0A3S9UCE2 >tr|A0A3S9UCE2|A0A3S9UCE2_9CAUD CobT-like cobalamin biosynthesis protein OS=Gordonia phage Gray OX=2499024 GN=73 PE=4 SV=1
	2::G8XUC3 >tr|G8XUC3|G8XUC3_9BETA Large tegument protein OS=Aotine betaherpesvirus 1 OX=50290 GN=UL48 PE=3 SV=1
	2::A0A516LXB0 >tr|A0A516LXB0|A0A516LXB0_9VIRU Uncharacterized protein OS=Prokaryotic dsDNA virus sp. OX=2591644 GN=Unbinned175contig1000_19 PE=4 SV=1
	2::A0A160DHM9 >tr|A0A160DHM9|A0A160DHM9_9CAUD Uncharacterized protein OS=Gordonia phage Jeanie OX=1838068 GN=3 PE=4 SV=1
	2::Q75491 >tr|Q75491|Q75491_9HIV1 Envelope glycoprotein (Fragment) OS=Human immunodeficiency virus 1 OX=11676 GN=env PE=4 SV=1
	2::A0A3G4ZXG6 >tr|A0A3G4ZXG6|A0A3G4ZXG6_9VIRU Uncharacterized protein OS=Edafosvirus sp. OX=2487765 GN=Edafosvirus5_27 PE=4 SV=1
	2::A0A2H4UVE2 >tr|A0A2H4UVE2|A0A2H4UVE2_9VIRU Serine/threonine-protein kinase OS=Bodo saltans virus OX=2024608 GN=BMW23_0847 PE=4 SV=1
	2::A0A1D6Y7D3 >tr|A0A1D6Y7D3|A0A1D6Y7D3_9VIRU Putative mRNA-capping enzyme OS=Golden Marseillevirus OX=1720526 GN=GMAR_ORF208 PE=4 SV=1
	2::A0A2Z4QKJ4 >tr|A0A2Z4QKJ4|A0A2Z4QKJ4_9VIRU Putative RNA-dependent RNA polymerase OS=Galbut virus OX=1654579 PE=4 SV=1

	Target	Decoy	False discovery rate
Peptide matches above identity threshold	151	84	55.63 %
Peptide matches above homology or identity threshold	220	144	65.45 %

Select Summary Report

Format As [Select Summary (protein hits)] Help Help

Significance threshold p< [0.05] Max. number of hits [AUTO] Show Percolator scores ☐

Standard scoring ○ MudPIT scoring ◉ Ions score or expect cut-off [40] Show sub-sets [0]

Show pop-ups ○ Suppress pop-ups ◉ Require bold red ☑

Preferred taxonomy [All entries]

Re-Search ◉ All queries ○ Unassigned ○ Below homology threshold ○ Below identity threshold

1. 2::A0A1U9WQS0 **Mass:** 112893 **Score:** 2819 **Matches:** 47(46) **Sequences:** 25(24) **emPAI:** 1.65

>tr|A0A1U9WQS0|A0A1U9WQS0_9CAUD Glycosylase OS=Geobacillus phage TP-84 OX=1965361 PE=4 SV=1

Query	Observed	Mr(expt)	Mr(calc)	ppm	Miss	Score	Expect	Rank	Unique	Peptide
264	423.2297	844.4448	844.4443	0.61	0	44	0.0086	1	U	R.NVAEWVK.Q
340	438.7429	875.4713	875.4712	0.03	0	78	7.4e-06	1	U	K.LSIGTTER.W 339
412	455.7294	909.4443	909.4444	-0.03	0	60	0.0002	1	U	K.LFAESTDK.N
578	494.2972	986.5799	986.5801	-0.19	0	72	0.00011	1	U	R.VPYVPTAIK.D
610	501.3034	1000.5922	1000.5917	0.48	0	96	4.7e-08	1	U	K.ISGLAGVTGVK.G
831	539.8004	1077.5863	1077.5859	0.45	0	80	5e-06	1	U	K.LIDAGLWYK.Q 830
1053	579.2672	1156.5199	1156.5183	1.40	0	87	4.3e-07	1	U	R.YDSMGQTTVR.V
1077	582.3085	1162.6025	1162.6022	0.21	0	55	0.0014	1	U	R.TLWPNYIEK.D 1078
1106	587.2641	1172.5136	1172.5132	0.33	0	(83)	4e-07	1	U	R.YDSMGQTTVR.V
1141	590.8096	1179.6047	1179.6023	2.01	0	87	6.3e-07	1	U	R.ADYDELITLK.V
1382	415.8839	1244.6299	1244.6295	0.26	0	40	0.16	1	U	K.MGEISRPGINR.D
1545	649.8352	1297.6558	1297.6554	0.29	0	105	1.1e-08	1	U	R.FSVIIDGQEYK.M 1544
1643	663.8219	1325.6292	1325.6286	0.50	0	69	3.3e-05	1	U	K.TTNISCEVQFK.V
1673	446.2419	1335.7040	1335.7034	0.44	1	(58)	0.00046	1	U	R.RADYDELITLK.V
1674	668.8597	1335.7048	1335.7034	1.08	1	76	1e-05	1	U	R.RADYDELITLK.V
1822	688.8184	1375.6222	1375.6190	2.26	0	100	1.2e-08	1	U	K.QCFDPSVNPNAK.C 1821
1852	691.3830	1380.7515	1380.7514	0.11	0	83	6.6e-07	1	U	K.GIGLWVLGQADPR.I 1853
1906	696.8231	1391.6317	1391.6326	-0.59	0	92	2.2e-07	1	U	R.AMNIAVHEYGCK.G 1907
1909	464.8858	1391.6357	1391.6326	2.24	0	(66)	9e-05	1	U	R.AMNIAVHEYGCK.G 1908
1969	704.8208	1407.6271	1407.6275	-0.30	0	(89)	1.6e-07	1	U	R.AMNIAVHEYGCK.G 1968

2504	808.3807	1614.7469	1614.7460	0.51	0	99	2e-07	1	U	K.CTIDNVYLGGAGYGR.R
2505	808.4384	1614.8623	1614.8617	0.36	1	95	1.4e-07	1	U	R.IKNEVIIDANPDFK.L
2506	539.2954	1614.8644	1614.8617	1.66	1	(60)	0.00035	1	U	R.IKNEVIIDANPDFK.L
2649	847.8985	1693.7824	1693.7835	-0.62	0	148	3.8e-13	1	U	R.VSEYNGIEYATAYSK.T
2811	877.9205	1753.8265	1753.8271	-0.37	0	121	1.8e-10	1	U	K.GVSEPTHNLSPYDNPK.R
2812	585.6166	1753.8279	1753.8271	0.43	0	(90)	2e-07	1	U	K.GVSEPTHNLSPYDNPK.R 2813
2890	895.9457	1789.8768	1789.8748	1.17	0	131	2.4e-11	1	U	K.ITSIGFHEFNVDAQGR.I 2889
2961	917.4720	1832.9295	1832.9268	1.45	0	167	8e-15	1	U	R.LLDSQTAQDNFINNLK.T 2960
2962	611.9842	1832.9307	1832.9268	2.14	0	(41)	0.028	1	U	R.LLDSQTAQDNFINNLK.T
3042	941.9053	1881.7961	1881.8017	-2.97	0	103	1.8e-09	1	U	K.DAQGSFTSFTDATTYDR.Y
4666	792.4191	3165.6472	3165.6499	-0.84	0	(50)	0.00078	1	U	R.EVLPDGSRPEISLDYVFKPIHVPSWTGK.K 4668 4669
4667	1056.2232	3165.6478	3165.6499	-0.67	0	55	0.00022	1	U	R.EVLPDGSRPEISLDYVFKPIHVPSWTGK.K
4747	1082.5370	3244.5892	3244.5962	-2.15	0	98	7.6e-08	1	U	K.ITGYACQLSEQPETVLTYTLNVPQAGTYK.I

2. 2::A0A1U9WQR1 **Mass:** 99414 **Score:** 828 **Matches:** 17(17) **Sequences:** 13(13) **emPAI:** 0.81
>tr|A0A1U9WQR1|A0A1U9WQR1_9CAUD Tail protein OS=Geobacillus phage TP-84 OX=1965361 PE=4 SV=1

Query	Observed	Mr(expt)	Mr(calc)	ppm	Miss	Score	Expect	Rank	Unique	Peptide
94	378.7188	755.4231	755.4218	1.78	0	44	0.0014	1	U	R.LYLYGK.N
612	501.7721	1001.5296	1001.5294	0.18	1	53	0.0024	1	U	R.IGVWKDER.Y
761	529.2797	1056.5449	1056.5451	-0.21	0	67	6.8e-05	1	U	K.TEVELNQPK.K 762
941	561.2958	1120.5770	1120.5764	0.53	0	75	8.4e-06	1	U	K.VINYEQVEK.T
944	561.7938	1121.5730	1121.5717	1.13	0	80	4.2e-06	1	U	R.GTSQDAFVGIK.V 945
1010	571.8200	1141.6254	1141.6244	0.90	0	87	9.7e-07	1	U	R.FGSPAAIGPGIR.G
1770	683.3587	1364.7029	1364.7010	1.41	0	98	6.8e-08	1	U	K.IMEVQVLDYQK.I 1769
1851	691.3560	1380.6975	1380.6959	1.17	0	(83)	1.5e-06	1	U	K.IMEVQVLDYQK.I
1902	695.9128	1389.8111	1389.8119	-0.59	0	92	2.2e-08	1	U	R.LTLTSEPLIIYK.D
2165	487.9001	1460.6785	1460.6783	0.11	0	76	6.4e-06	1	U	K.VTIHYTEPDSDGK.T
2367	779.3678	1556.7211	1556.7181	1.92	0	57	0.00039	1	U	R.YTIPQNLYDDGMK.M
2387	522.6385	1564.8937	1564.8936	0.04	1	82	7.8e-06	1	U	K.RIELGAEPVELIAR.I
3591	1071.5602	2141.1058	2141.1157	-4.61	0	83	1.5e-06	1	U	K.TLQLQSFVAPFDITAAYTR.S
4910	1176.2001	3525.5784	3525.5784	0.01	0	65	2.1e-05	1	U	K.NFVTGQYNTNLMTWTSPTIDLGQDGFDDFGR.V

3. 2::A0A1U9WQN1 **Mass:** 38540 **Score:** 549 **Matches:** 8(8) **Sequences:** 5(5) **emPAI:** 0.73
>tr|A0A1U9WQN1|A0A1U9WQN1_9CAUD Thymidylate synthase OS=Geobacillus phage TP-84 OX=1965361 PE=4 SV=1

Query	Observed	Mr(expt)	Mr(calc)	ppm	Miss	Score	Expect	Rank	Unique	Peptide
994	379.5395	1135.5968	1135.5985	-1.57	1	(52)	0.0021	1	U	K.KGEISGNYIR.D
995	568.8065	1135.5984	1135.5985	-0.10	1	82	2.5e-06	1	U	K.KGEISGNYIR.D
1723	677.8378	1353.6610	1353.6598	0.86	0	95	3.1e-07	1	U	K.VAGYEEAIMSLR.M
2141	363.6992	1450.7679	1450.7681	-0.14	0	46	0.0052	1	U	K.IQHLVYAVTDHR.G
2739	576.6084	1726.8032	1726.8023	0.55	0	(66)	4.6e-05	1	U	R.GAQDDLDAHAIAFNNR.I
2740	864.4094	1726.8042	1726.8023	1.11	0	126	4.5e-11	1	U	R.GAQDDLDAHAIAFNNR.I
3189	968.9845	1935.9545	1935.9578	-1.72	0	135	1e-11	1	U	R.LANIQETVLSEWYQDK.I 3190

4. 2::A0A076FL24 **Mass:** 27039 **Score:** 230 **Matches:** 4(4) **Sequences:** 3(3) **emPAI:** 0.86
>tr|A0A076FL24|A0A076FL24_9MONO Enhanced green fluorescent protein OS=Measles morbillivirus MeV-eGFP_Edm-tag OX=1524961 GN=eGFP PE=4 SV=1

Query	Observed	Mr(expt)	Mr(calc)	ppm	Miss	Score	Expect	Rank	Unique	Peptide
1473	633.7929	1265.5713	1265.5710	0.23	0	69	2.3e-05	1	U	K.SAMPEGYVQER.T
1502	641.7916	1281.5687	1281.5659	2.12	0	(61)	0.00014	1	U	K.SAMPEGYVQER.T
4366	688.6202	2750.4518	2750.4491	1.00	1	81	1.6e-06	1	U	M.VSKGEELFTGVVPILVELDGDVNGHK.F
4429	725.3791	2897.4875	2897.4845	1.04	1	95	6.3e-08	1	U	-.MVSKGEELFTGVVPILVELDGDVNGHK.F

Proteins matching the same set of peptides:
2::A0A076V611 **Mass:** 27039 **Score:** 230 **Matches:** 4(4) **Sequences:** 3(3)
>tr|A0A076V611|A0A076V611_9VIRU EGFP OS=synthetic recombinant Chikungunya virus strain LS3-GFP OX=1529938 PE=4 SV=1
2::A0A0U3A3D8 **Mass:** 27039 **Score:** 230 **Matches:** 4(4) **Sequences:** 3(3)
>tr|A0A0U3A3D8|A0A0U3A3D8_9MONO EGFP OS=Zaire ebolavirus OX=186538 PE=4 SV=1
2::A0A1V0D974 **Mass:** 27010 **Score:** 230 **Matches:** 4(4) **Sequences:** 3(3)
>tr|A0A1V0D974|A0A1V0D974_9POXV EGFP OS=Vaccinia virus OX=10245 GN=EGFP PE=4 SV=1
2::A0A348GST9 **Mass:** 27039 **Score:** 230 **Matches:** 4(4) **Sequences:** 3(3)
>tr|A0A348GST9|A0A348GST9_9MONO Enhanced green fluorescent protein OS=Measles morbillivirus OX=11234 GN=EGFP PE=4 SV=1
2::A0A3Q8WKS4 **Mass:** 27039 **Score:** 230 **Matches:** 4(4) **Sequences:** 3(3)
>tr|A0A3Q8WKS4|A0A3Q8WKS4_9VIRU Enhanced green fluorescence protein OS=Sacbrood virus OX=89463 PE=4 SV=1
2::A0A5J6XWW3 **Mass:** 27089 **Score:** 230 **Matches:** 4(4) **Sequences:** 3(3)
>tr|A0A5J6XWW3|A0A5J6XWW3_9GAMA YFP OS=Macacine gammaherpesvirus 5 OX=154334 PE=4 SV=1
2::C5MKY7 **Mass:** 27039 **Score:** 230 **Matches:** 4(4) **Sequences:** 3(3)
>tr|C5MKY7|C5MKY7_HCMV Enhanced green fluorescent protein OS=Human cytomegalovirus OX=10359 GN=egfp PE=4 SV=1

5. 2::W6LP63 **Mass:** 47022 **Score:** 181 **Matches:** 3(3) **Sequences:** 3(3) **emPAI:** 0.31
>tr|W6LP63|W6LP63_9CAUD Enolase OS=Streptococcus phage 20617 OX=1392231 GN=eno PE=3 SV=1

Query	Observed	Mr(expt)	Mr(calc)	ppm	Miss	Score	Expect	Rank	Unique	Peptide
119	386.2060	770.3975	770.3963	1.62	0	54	0.00074	1	U	K.SFYNLK.K
177	403.7294	805.4442	805.4446	-0.54	0	44	0.021	1	U	K.YNQLLR.I
3302	990.9999	1979.9852	1979.9874	-1.09	0	150	2.8e-13	1	U	K.VNQIGTLTETFDAIEMAK.E

6. 2::A0A0M4R3J1 **Mass:** 28457 **Score:** 73 **Matches:** 2(2) **Sequences:** 1(1) **emPAI:** 0.16
>tr|A0A0M4R3J1|A0A0M4R3J1_9CAUD Uncharacterized protein OS=Arthrobacter phage Brent OX=1701798 GN=SEA_BRENT_43 PE=4 SV=1

Query	Observed	Mr(expt)	Mr(calc)	ppm	Miss	Score	Expect	Rank	Unique	Peptide
782	532.3359	1062.6572	1062.6550	2.12	0	46	0.00031	1	U	R.LNVLHIAVGK.L 780

Proteins matching the same set of peptides:
2::A0A222Z3H5 **Mass:** 28457 **Score:** 73 **Matches:** 2(2) **Sequences:** 1(1)
>tr|A0A222Z3H5|A0A222Z3H5_9CAUD Uncharacterized protein OS=Arthrobacter phage Franzy OX=2024007 GN=SEA_FRANZY_43 PE=4 SV=1
2::A0A222Z9C1 **Mass:** 26813 **Score:** 73 **Matches:** 2(2) **Sequences:** 1(1)
>tr|A0A222Z9C1|A0A222Z9C1_9CAUD Uncharacterized protein OS=Arthrobacter phage Piccoletto OX=2024282 GN=SEA_PICCOLETTO_43 PE=4 SV=1

7. 2::A0A1U9WQP5 **Mass:** 62259 **Score:** 71 **Matches:** 2(2) **Sequences:** 2(2) **emPAI:** 0.15
>tr|A0A1U9WQP5|A0A1U9WQP5_9CAUD Capsid portal protein OS=Geobacillus phage TP-84 OX=1965361 PE=4 SV=1

Query	Observed	Mr(expt)	Mr(calc)	ppm	Miss	Score	Expect	Rank	Unique	Peptide
1080	582.8077	1163.6008	1163.6009	-0.05	0	45	0.016	1	U	K.TPYIVANVCK.M
1280	406.8557	1217.5454	1217.5465	-0.97	0	41	0.011	1	U	R.DVYYPHPDGR.G

8. 2::Q66NJ1 **Mass:** 24571 **Score:** 70 **Matches:** 2(2) **Sequences:** 1(1) **emPAI:** 0.19
>tr|Q66NJ1|Q66NJ1_9HIV1 Reverse transcriptase (Fragment) OS=Human immunodeficiency virus 1 OX=11676 GN=pol PE=4 SV=1

Query	Observed	Mr(expt)	Mr(calc)	ppm	Miss	Score	Expect	Rank	Unique	Peptide
419	457.7507	913.4868	913.4869	-0.08	0	55	0.00068	1	U	K.IGPEETIR.X 420

9. 2::Q6R4V1 **Mass:** 140130 **Score:** 69 **Matches:** 1(1) **Sequences:** 1(1) **emPAI:** 0.03
>tr|Q6R4V1|Q6R4V1_9CAUD Putative replication protein RepA OS=Vibrio virus VP882 OX=261726 PE=4 SV=1

Query	Observed	Mr(expt)	Mr(calc)	ppm	Miss	Score	Expect	Rank	Unique	Peptide
628	503.7822	1005.5499	1005.5495	0.44	0	69	9.4e-05	1	U	K.ALLEIYER.E

10. 2::A0A059UHZ7 Mass: 130888 Score: 68 Matches: 3(3) Sequences: 2(2) emPAI: 0.07
>tr|A0A059UHZ7|A0A059UHZ7_9RETR Pol protein (Fragment) OS=Feline immunodeficiency virus OX=11673 GN=pol PE=3 SV=1

Query	Observed	Mr(expt)	Mr(calc)	ppm	Miss	Score	Expect	Rank	Unique	Peptide
422	458.2613	914.5081	914.5073	0.85	0	44	0.055	1	U	K.QVADEIIK.K
722	348.5419	1042.6039	1042.6022	1.61	1	(45)	0.011	1	U	K.QVADEIIKK.C
723	522.3093	1042.6041	1042.6022	1.81	1	53	0.0017	1	U	K.QVADEIIKK.C

11. 2::A0A0M3SUP6 Mass: 49172 Score: 63 Matches: 2(2) Sequences: 1(1) emPAI: 0.09
>tr|A0A0M3SUP6|A0A0M3SUP6_9VIRU RNA-dependent RNA polymerase (Fragment) OS=Rhizoctonia solani beny-like virus 1 OX=1708385 PE=4 SV=1

Query	Observed	Mr(expt)	Mr(calc)	ppm	Miss	Score	Expect	Rank	Unique	Peptide
20	358.7123	715.4101	715.4116	-2.04	0	54	0.0029	1	U	R.IVEEVK.V 21

Proteins matching the same set of peptides:
2::A0A127AW26 Mass: 20044 Score: 63 Matches: 2(2) Sequences: 1(1)
>tr|A0A127AW26|A0A127AW26_9CAUD Uncharacterized protein OS=Bacillus phage SP-15 OX=1792032 GN=SP15_039 PE=4 SV=1
2::A0A1L3KLR4 Mass: 49735 Score: 63 Matches: 2(2) Sequences: 1(1)
>tr|A0A1L3KLR4|A0A1L3KLR4_9VIRU RdRp OS=Wenling partiti-like virus 13 OX=1923519 PE=4 SV=1
2::A0A3G5AHH2 Mass: 53785 Score: 63 Matches: 2(2) Sequences: 1(1)
>tr|A0A3G5AHH2|A0A3G5AHH2_9VIRU Uncharacterized protein OS=Sylvanvirus sp. OX=2487774 GN=Sylvanvirus4_2 PE=4 SV=1
2::A0MNA8 Mass: 17169 Score: 63 Matches: 2(2) Sequences: 1(1)
>tr|A0MNA8|A0MNA8_9CAUD Uncharacterized protein OS=Thermus phage phiYS40 OX=407392 GN=YS40_090 PE=4 SV=1
2::G0T5E5 Mass: 52997 Score: 63 Matches: 2(2) Sequences: 1(1)
>tr|G0T5E5|G0T5E5_IRV9 Uncharacterized protein OS=Wiseana iridescent virus OX=68347 PE=4 SV=1
2::J9PV61 Mass: 25120 Score: 63 Matches: 2(2) Sequences: 1(1)
>tr|J9PV61|J9PV61_9CAUD Putative radical activating enzyme OS=Bacillus phage BCD7 OX=1136534 GN=BCD7_0027 PE=3 SV=1

12. 2::A0A0M5M5R9 Mass: 16061 Score: 62 Matches: 2(2) Sequences: 1(1) emPAI: 0.29
>tr|A0A0M5M5R9|A0A0M5M5R9_9CAUD SsDNA-binding protein OS=Citrobacter phage Margaery OX=1701810 GN=CPT_Margaery222 PE=4 SV=1

Query	Observed	Mr(expt)	Mr(calc)	ppm	Miss	Score	Expect	Rank	Unique	Peptide
318	435.2487	868.4828	868.4807	2.44	0	52	0.0011	1	U	R.LLFYTGR.H 317

Proteins matching the same set of peptides:
2::A0A1W5N0M8 Mass: 16061 Score: 62 Matches: 2(2) Sequences: 1(1)
>tr|A0A1W5N0M8|A0A1W5N0M8_9CAUD Recombination, repair and ssDNA binding protein OS=Cronobacter phage vB_CsaM_leB OX=1885242 GN=B_221 PE=4 SV=1
2::A0A1W5P0W1 Mass: 16070 Score: 62 Matches: 2(2) Sequences: 1(1)
>tr|A0A1W5P0W1|A0A1W5P0W1_9CAUD Recombination, repair and ssDNA binding protein OS=Cronobacter phage vB_CsaM_leE OX=1873954 GN=E_226 PE=4 SV=1
2::A0A1X8VCA5 Mass: 16061 Score: 62 Matches: 2(2) Sequences: 1(1)
>tr|A0A1X8VCA5|A0A1X8VCA5_9CAUD Recombination, repair and sssDNA binding protein OS=Cronobacter phage vB_CsaM_leN OX=1885245 GN=N_227 PE=4 SV=1
2::A0A3B8DIS1 Mass: 16061 Score: 62 Matches: 2(2) Sequences: 1(1)
>tr|A0A3B8DIS1|A0A3B8DIS1_9CAUD UvsY-like protein OS=Citrobacter phage Maroon OX=2315469 GN=CPT_Maroon_218 PE=4 SV=1
2::K4F7S7 Mass: 16061 Score: 62 Matches: 2(2) Sequences: 1(1)
>tr|K4F7S7|K4F7S7_9CAUD Recombination, repair and ssDNA binding protein OS=Cronobacter phage vB_CsaM_GAP161 OX=1141138 GN=GAP161_213 PE=4 SV=1

13. 2::A0A0E4G1G1 Mass: 74805 Score: 62 Matches: 1(1) Sequences: 1(1) emPAI: 0.06
>tr|A0A0E4G1G1|A0A0E4G1G1_9CAUD Putative phage structural protein OS=Staphylococcus phage PSa3 OX=1319980 GN=PSa3_ORF15c PE=4 SV=1

Query	Observed	Mr(expt)	Mr(calc)	ppm	Miss	Score	Expect	Rank	Unique	Peptide
193	407.2627	812.5109	812.5120	-1.35	0	62	0.00024	1	U	R.LIEVLAR.R

Proteins matching the same set of peptides:
2::A0A173G9L4 Mass: 74538 Score: 62 Matches: 1(1) Sequences: 1(1)
>tr|A0A173G9L4|A0A173G9L4_9CAUD Putative major teichoic acid biosynthesis protein C OS=Staphylococcus phage SLPW OX=1821228 PE=4 SV=1
2::A0A2I7SBV8 Mass: 74469 Score: 62 Matches: 1(1) Sequences: 1(1)
>tr|A0A2I7SBV8|A0A2I7SBV8_9CAUD Putative major teichoic acid biosynthesis protein C OS=Staphylococcus phage vB_SauP_phiAGO1.3 OX=2074138 GN=phiAGO13_15 PE=4 SV=1
2::A0A2I7SBX4 Mass: 74469 Score: 62 Matches: 1(1) Sequences: 1(1)
>tr|A0A2I7SBX4|A0A2I7SBX4_9CAUD Putative major teichoic acid biosynthesis protein C OS=Staphylococcus phage vB_SauP_phiAGO1.9 OX=2074139 GN=phiAGO19_15 PE=4 SV=1
2::A0A3S7JLM6 Mass: 74716 Score: 62 Matches: 1(1) Sequences: 1(1)
>tr|A0A3S7JLM6|A0A3S7JLM6_9CAUD Minor tail protein OS=Staphylococcus phage CSA13 OX=2108359 GN=phCSA13_006 PE=4 SV=1
2::A0A5J6T7G3 Mass: 74497 Score: 62 Matches: 1(1) Sequences: 1(1)
>tr|A0A5J6T7G3|A0A5J6T7G3_9CAUD Receptor binding protein OS=Staphylococcus phage Portland OX=2650876 GN=CPT_Portland_014 PE=4 SV=1
2::Q4ZE58 Mass: 74751 Score: 62 Matches: 1(1) Sequences: 1(1)
>tr|Q4ZE58|Q4ZE58_9CAUD ORF002 OS=Staphylococcus phage 66 OX=320832 PE=4 SV=1
2::W6E9L3 Mass: 74696 Score: 62 Matches: 1(1) Sequences: 1(1)
>tr|W6E9L3|W6E9L3_9CAUD Putative minor structure protein OS=Staphylococcus phage GRCS OX=1453367 GN=GRCS_0016 PE=4 SV=1
2::G9M949 Mass: 74474 Score: 62 Matches: 1(1) Sequences: 1(1)
>tr|G9M949|G9M949_BPPS4 Structure protein, minor tail protein OS=Staphylococcus phage S24-1 OX=1010614 PE=4 SV=1
2::G9M970 Mass: 74525 Score: 62 Matches: 1(1) Sequences: 1(1)
>tr|G9M970|G9M970_BPPSP Putative minor structure protein OS=Staphylococcus phage S13' OX=1010615 PE=4 SV=1
2::Q859I6 Mass: 74806 Score: 62 Matches: 1(1) Sequences: 1(1)
>tr|Q859I6|Q859I6_9CAUD Minor structural protein OS=Staphylococcus phage P68 OX=204090 PE=4 SV=1
2::Q859K8 Mass: 74806 Score: 62 Matches: 1(1) Sequences: 1(1)
>tr|Q859K8|Q859K8_BP44A Minor structural protein OS=Staphylococcus phage 44AHJD OX=204086 PE=4 SV=1

14. 2::A0A223W084 Mass: 26567 Score: 60 Matches: 1(1) Sequences: 1(1) emPAI: 0.17
>tr|A0A223W084|A0A223W084_9CAUD Uncharacterized protein OS=Agrobacterium phage Atu_ph08 OX=2024265 PE=4 SV=1

Query	Observed	Mr(expt)	Mr(calc)	ppm	Miss	Score	Expect	Rank	Unique	Peptide
199	408.7453	815.4760	815.4752	0.86	0	60	0.00057	1	U	K.IVAEGLSK.L

15. 2::A0A3S7SGD4 Mass: 7434 Score: 59 Matches: 2(2) Sequences: 1(1) emPAI: 0.72
>tr|A0A3S7SGD4|A0A3S7SGD4_9CAUD Uncharacterized protein OS=Aerococcus phage vB_AviM_AVP OX=2495576 GN=AVP_16 PE=4 SV=1

Query	Observed	Mr(expt)	Mr(calc)	ppm	Miss	Score	Expect	Rank	Unique	Peptide
622	503.2835	1004.5524	1004.5542	-1.84	0	48	0.0052	1	U	K.LDGLEFALK.T 623

16. 2::A0A0K1LLW6 Mass: 10749 Score: 59 Matches: 1(1) Sequences: 1(1) emPAI: 0.46
>tr|A0A0K1LLW6|A0A0K1LLW6_9CAUD Uncharacterized protein OS=Caulobacter phage Sansa OX=1675600 GN=CPT_Sansa92 PE=4 SV=1

Query	Observed	Mr(expt)	Mr(calc)	ppm	Miss	Score	Expect	Rank	Unique	Peptide
48	364.7366	727.4586	727.4592	-0.80	0	59	0.00045	1	U	M.IVLEVR.E

17. 2::C0M298 Mass: 54569 Score: 59 Matches: 1(1) Sequences: 1(1) emPAI: 0.08
>tr|C0M298|C0M298_BPMS2 Replicase protein (Fragment) OS=Enterobacteria phage MS2 OX=12022 GN=MS2g4 PE=4 SV=1

Query	Observed	Mr(expt)	Mr(calc)	ppm	Miss	Score	Expect	Rank	Unique	Peptide
126	387.2186	772.4226	772.4232	-0.68	0	59	0.00099	1	U	R.LLADWR.T

Proteins matching the same set of peptides:
2::G4XQQ4 Mass: 29647 Score: 59 Matches: 1(1) Sequences: 1(1)
>tr|G4XQQ4|G4XQQ4_9HIV1 Pol protein (Fragment) OS=Human immunodeficiency virus 1 OX=11676 GN=pol PE=4 SV=2

18. 2::A0A097BY06 Mass: 77954 Score: 59 Matches: 1(1) Sequences: 1(1) emPAI: 0.06
>tr|A0A097BY06|A0A097BY06_9CAUD Minor tail protein OS=Mycobacterium phage Power OX=1552733 GN=PBI_POWER_31 PE=4 SV=1

Query	Observed	Mr(expt)	Mr(calc)	ppm	Miss	Score	Expect	Rank	Unique	Peptide
115	385.2439	768.4733	768.4745	-1.55	0	59	8.4e-05	1	U	R.LPLDALK.T

Proteins matching the same set of peptides:
2::A0A097EWN0 **Mass:** 78208 **Score:** 59 **Matches:** 1(1) **Sequences:** 1(1)
>tr|A0A097EWN0|A0A097EWN0_9CAUD Minor tail protein OS=Mycobacterium phage SweetiePie OX=1555235 GN=31 PE=4 SV=1
2::A0A0M3UL68 **Mass:** 84250 **Score:** 59 **Matches:** 1(1) **Sequences:** 1(1)
>tr|A0A0M3UL68|A0A0M3UL68_9CAUD Minor tail protein OS=Mycobacterium phage LadyBird OX=1718166 GN=SEA_LADYBIRD_31 PE=4 SV=1
2::A0A1C9LZW9 **Mass:** 77898 **Score:** 59 **Matches:** 1(1) **Sequences:** 1(1)
>tr|A0A1C9LZW9|A0A1C9LZW9_9CAUD Minor tail protein OS=Mycobacterium phage DudeLittle OX=1897766 GN=SEA_DUDELITTLE_31 PE=4 SV=1
2::A0A220NSE9 **Mass:** 63313 **Score:** 59 **Matches:** 1(1) **Sequences:** 1(1)
>tr|A0A220NSE9|A0A220NSE9_9CAUD Minor tail protein OS=Mycobacterium phage Heffalump OX=1983575 GN=33 PE=4 SV=1
2::A0A2D1G5J0 **Mass:** 77933 **Score:** 59 **Matches:** 1(1) **Sequences:** 1(1)
>tr|A0A2D1G5J0|A0A2D1G5J0_9CAUD Minor tail protein OS=Mycobacterium phage Anselm OX=2041517 GN=SEA_ANSELM_31 PE=4 SV=1
2::A0A2D1G9Y3 **Mass:** 78257 **Score:** 59 **Matches:** 1(1) **Sequences:** 1(1)
>tr|A0A2D1G9Y3|A0A2D1G9Y3_9CAUD Minor tail protein OS=Mycobacterium phage Dalmatian OX=2041527 GN=SEA_DALMATIAN_31 PE=4 SV=1
2::A0A2D1GJ93 **Mass:** 78238 **Score:** 59 **Matches:** 1(1) **Sequences:** 1(1)
>tr|A0A2D1GJ93|A0A2D1GJ93_9CAUD Minor tail protein OS=Mycobacterium phage SnapTap OX=2041549 GN=SEA_SNAPTAP_31 PE=4 SV=1
2::A0A2R4AP72 **Mass:** 78230 **Score:** 59 **Matches:** 1(1) **Sequences:** 1(1)
>tr|A0A2R4AP72|A0A2R4AP72_9CAUD Minor tail protein OS=Mycobacterium phage Fameo OX=2126943 GN=31 PE=4 SV=1
2::A0A2Z5H0E5 **Mass:** 78208 **Score:** 59 **Matches:** 1(1) **Sequences:** 1(1)
>tr|A0A2Z5H0E5|A0A2Z5H0E5_9CAUD Minor tail protein OS=Mycobacterium phage Crucio OX=2234072 GN=31 PE=4 SV=1
2::A0A385D5H4 **Mass:** 77889 **Score:** 59 **Matches:** 1(1) **Sequences:** 1(1)
>tr|A0A385D5H4|A0A385D5H4_9CAUD Minor tail protein OS=Mycobacterium phage QueenBeesly OX=2301544 GN=31 PE=4 SV=1
2::A0A5J6TLJ6 **Mass:** 78367 **Score:** 59 **Matches:** 1(1) **Sequences:** 1(1)
>tr|A0A5J6TLJ6|A0A5J6TLJ6_9CAUD Minor tail protein OS=Mycobacterium phage SemperFi OX=2599879 GN=33 PE=4 SV=1
2::M4HZ75 **Mass:** 84118 **Score:** 59 **Matches:** 1(1) **Sequences:** 1(1)
>tr|M4HZ75|M4HZ75_9CAUD Minor tail subunit OS=Mycobacterium phage First OX=1245814 GN=First_0031 PE=4 SV=1
2::W8E958 **Mass:** 84119 **Score:** 59 **Matches:** 1(1) **Sequences:** 1(1)
>tr|W8E958|W8E958_9CAUD Minor tail subunit OS=Mycobacterium phage 20ES OX=1458726 GN=20ES_31 PE=4 SV=1

---

19. 2::A0A1V0DZB4 **Mass:** 48404 **Score:** 57 **Matches:** 2(2) **Sequences:** 1(1) **emPAI:** 0.09
>tr|A0A1V0DZB4|A0A1V0DZB4_9CAUD Replicative DNA helicase OS=Acinetobacter phage WCHABP12 OX=1965454 GN=dnaB PE=4 SV=1

Query	Observed	Mr(expt)	Mr(calc)	ppm	Miss	Score	Expect	Rank	Unique	Peptide
7	351.7051	701.3957	701.3959	-0.38	0	51	0.0041	1	U	K.LDIVDK.A 8

Proteins matching the same set of peptides:
2::A0A1X6WG70 **Mass:** 156443 **Score:** 57 **Matches:** 2(2) **Sequences:** 1(1)
>tr|A0A1X6WG70|A0A1X6WG70_9VIRU Uncharacterized protein OS=Pacmanvirus A23 OX=1932881 GN=PACV_169 PE=4 SV=1
2::A0A1X9SFL1 **Mass:** 48404 **Score:** 57 **Matches:** 2(2) **Sequences:** 1(1)
>tr|A0A1X9SFL1|A0A1X9SFL1_9CAUD Replicative DNA helicase OS=Acinetobacter phage WCHABP1 OX=1983409 GN=dnaB PE=4 SV=1
2::A0A220NQM0 **Mass:** 48501 **Score:** 57 **Matches:** 2(2) **Sequences:** 1(1)
>tr|A0A220NQM0|A0A220NQM0_9CAUD Replicative DNA helicase OS=Acinetobacter phage AbP2 OX=2015804 GN=ABP2_079 PE=4 SV=1
2::A0A2L0UZ96 **Mass:** 87135 **Score:** 57 **Matches:** 2(2) **Sequences:** 1(1)
>tr|A0A2L0UZ96|A0A2L0UZ96_9CAUD Zeta_toxin domain-containing protein OS=Agrobacterium phage Atu_ph07 OX=2024264 PE=4 SV=1
2::A0A3G3BXJ7 **Mass:** 48461 **Score:** 57 **Matches:** 2(2) **Sequences:** 1(1)
>tr|A0A3G3BXJ7|A0A3G3BXJ7_9CAUD Replicative DNA helicase OS=Acinetobacter phage vB_KpnM_IME284 OX=2419625 PE=4 SV=1
2::A0A3G3BXU9 **Mass:** 48404 **Score:** 57 **Matches:** 2(2) **Sequences:** 1(1)
>tr|A0A3G3BXU9|A0A3G3BXU9_9CAUD Replicative DNA helicase OS=Acinetobacter phage vB_KpnM_IME512 OX=2419627 PE=4 SV=1
2::A0A482MGD6 **Mass:** 93180 **Score:** 57 **Matches:** 2(2) **Sequences:** 1(1)
>tr|A0A482MGD6|A0A482MGD6_9CAUD Tape measure protein OS=Serratia phage Serbin OX=2562181 GN=CPT_Serbin_021 PE=4 SV=1
2::A0A516LKU8 **Mass:** 7405 **Score:** 57 **Matches:** 2(2) **Sequences:** 1(1)
>tr|A0A516LKU8|A0A516LKU8_9VIRU Uncharacterized protein OS=Prokaryotic dsDNA virus sp. OX=2591644 GN=Tp118DCM00d2C30442581_17 PE=4 SV=1
2::A7DYD4 **Mass:** 35730 **Score:** 57 **Matches:** 2(2) **Sequences:** 1(1)
>tr|A7DYD4|A7DYD4_9CAUD Uncharacterized protein OS=Streptococcus phage M102 OX=372457 PE=4 SV=1

---

20. 2::A0A482MLD2 **Mass:** 11951 **Score:** 56 **Matches:** 1(1) **Sequences:** 1(1) **emPAI:** 0.41
>tr|A0A482MLD2|A0A482MLD2_9CAUD Uncharacterized protein OS=Caudovirales GX15bay OX=2530034 PE=4 SV=1

Query	Observed	Mr(expt)	Mr(calc)	ppm	Miss	Score	Expect	Rank	Unique	Peptide
200	408.7456	815.4767	815.4752	1.81	1	56	0.0014	1	U	R.ADEILKK.R

---

21. 2::A0A141HS99 **Mass:** 104742 **Score:** 56 **Matches:** 2(2) **Sequences:** 1(1) **emPAI:** 0.04
>tr|A0A141HS99|A0A141HS99_9CAUD Uncharacterized protein OS=Bacillus phage vB_BpuM-BpSp OX=1739968 GN=Bp8pS_208 PE=4 SV=1

Query	Observed	Mr(expt)	Mr(calc)	ppm	Miss	Score	Expect	Rank	Unique	Peptide
88	376.6948	751.3750	751.3752	-0.24	0	46	0.0054	1	U	K.EFSLEK.L 87

---

22. 2::A0A3G5A6F4 **Mass:** 24080 **Score:** 54 **Matches:** 4(1) **Sequences:** 1(1) **emPAI:** 0.19
>tr|A0A3G5A6F4|A0A3G5A6F4_9VIRU ANK_REP_REGION domain-containing protein OS=Hyperionvirus sp. OX=2487770 GN=Hyperionvirus2_195 PE=4 SV=1

Query	Observed	Mr(expt)	Mr(calc)	ppm	Miss	Score	Expect	Rank	Unique	Peptide
73	373.7148	745.4151	745.4156	-0.74	0	54	0.01	1	U	K.LVMIDR.V 72
109	381.7120	761.4094	761.4106	-1.56	0	(44)	0.13	1	U	K.LVMIDR.V 110

---

23. 2::A0A0A0YVX1 **Mass:** 18771 **Score:** 54 **Matches:** 1(1) **Sequences:** 1(1) **emPAI:** 0.25
>tr|A0A0A0YVX1|A0A0A0YVX1_9CAUD Uncharacterized protein OS=Pseudomonas phage YH6 OX=1566995 GN=YH6_053 PE=4 SV=1

Query	Observed	Mr(expt)	Mr(calc)	ppm	Miss	Score	Expect	Rank	Unique	Peptide
541	486.7587	971.5029	971.5036	-0.73	0	54	0.0019	1	U	R.QELAGALDR.S

Proteins matching the same set of peptides:
2::A0A0A7NP13 **Mass:** 18863 **Score:** 54 **Matches:** 1(1) **Sequences:** 1(1)
>tr|A0A0A7NP13|A0A0A7NP13_9CAUD Uncharacterized protein OS=Pseudomonas phage RWG OX=1541890 PE=4 SV=1
2::A0A0A7NUJ8 **Mass:** 18863 **Score:** 54 **Matches:** 1(1) **Sequences:** 1(1)
>tr|A0A0A7NUJ8|A0A0A7NUJ8_9CAUD Uncharacterized protein OS=Pseudomonas phage Pa2 OX=1530400 PE=4 SV=1
2::A0A0E3T9S8 **Mass:** 18771 **Score:** 54 **Matches:** 1(1) **Sequences:** 1(1)
>tr|A0A0E3T9S8|A0A0E3T9S8_9CAUD Tail assembly protein OS=Pseudomonas phage YH30 OX=1636189 GN=YH30_58 PE=4 SV=1
2::A0A0F6YQ45 **Mass:** 18771 **Score:** 54 **Matches:** 1(1) **Sequences:** 1(1)
>tr|A0A0F6YQ45|A0A0F6YQ45_9CAUD Uncharacterized protein OS=Pseudomonas phage DL64 OX=1640973 PE=4 SV=1
2::A0A172B2L2 **Mass:** 18785 **Score:** 54 **Matches:** 1(1) **Sequences:** 1(1)
>tr|A0A172B2L2|A0A172B2L2_9CAUD Uncharacterized protein OS=Pseudomonas phage vB_PaeP_MAG4 OX=1639814 GN=vB_PaeP_fi6_051 PE=4 SV=1
2::A0A191ZBJ9 **Mass:** 18863 **Score:** 54 **Matches:** 1(1) **Sequences:** 1(1)
>tr|A0A191ZBJ9|A0A191ZBJ9_9CAUD Uncharacterized protein OS=Pseudomonas phage PEV2 OX=1837850 PE=4 SV=1
2::A0A1X9I8I9 **Mass:** 20007 **Score:** 54 **Matches:** 1(1) **Sequences:** 1(1)
>tr|A0A1X9I8I9|A0A1X9I8I9_9CAUD Uncharacterized protein OS=Pseudomonas phage vB_Pae575P-3 OX=1868829 GN=vB_Pae575P-3_50 PE=4 SV=1
2::A0A1X9I8V5 **Mass:** 20007 **Score:** 54 **Matches:** 1(1) **Sequences:** 1(1)
>tr|A0A1X9I8V5|A0A1X9I8V5_9CAUD Uncharacterized protein OS=Pseudomonas phage vB_Pae1396P-5 OX=1868842 GN=vB_Pae1369P-5_49 PE=4 SV=1
2::A0A2K8HK35 **Mass:** 18863 **Score:** 54 **Matches:** 1(1) **Sequences:** 1(1)
>tr|A0A2K8HK35|A0A2K8HK35_9CAUD Uncharacterized protein OS=Pseudomonas phage vB_PaeP_PYO2 OX=2034342 GN=vBPaePPYO2_00050 PE=4 SV=1
2::A0A2K8IBX6 **Mass:** 18863 **Score:** 54 **Matches:** 1(1) **Sequences:** 1(1)
>tr|A0A2K8IBX6|A0A2K8IBX6_9CAUD Uncharacterized protein OS=Pseudomonas phage vB_PaeP_DEV OX=2034344 GN=vBPaePDEV_00050 PE=4 SV=1
2::A0A2Z4PYZ4 **Mass:** 18805 **Score:** 54 **Matches:** 1(1) **Sequences:** 1(1)
>tr|A0A2Z4PYZ4|A0A2Z4PYZ4_9CAUD Uncharacterized protein OS=Pseudomonas phage LP14 OX=2234081 PE=4 SV=1
2::X5KIM2 **Mass:** 20115 **Score:** 54 **Matches:** 1(1) **Sequences:** 1(1)
>tr|X5KIM2|X5KIM2_9CAUD Uncharacterized protein OS=Pseudomonas phage vB_PaeP_C2-10_Ab09 OX=1476391 GN=ORF44 PE=4 SV=1
2::I7CML0 **Mass:** 18771 **Score:** 54 **Matches:** 1(1) **Sequences:** 1(1)
>tr|I7CML0|I7CML0_9CAUD Uncharacterized protein OS=Pseudomonas phage PA26 OX=1204542 PE=4 SV=1
2::C8ZKR2 **Mass:** 18771 **Score:** 54 **Matches:** 1(1) **Sequences:** 1(1)
>tr|C8ZKR2|C8ZKR2_9CAUD Uncharacterized protein OS=Pseudomonas phage LIT1 OX=655098 PE=4 SV=1

---

24. 2::A0A068EKL1 **Mass:** 46526 **Score:** 54 **Matches:** 1(1) **Sequences:** 1(1) **emPAI:** 0.09
>tr|A0A068EKL1|A0A068EKL1_9POXV Ankyrin repeat protein OS=Pigeonpox virus OX=10264 GN=fep_016 PE=4 SV=1

Query	Observed	Mr(expt)	Mr(calc)	ppm	Miss	Score	Expect	Rank	Unique	Peptide
89	376.7133	751.4120	751.4116	0.56	0	54	0.0012	1	U	K.LSYEIK.Y

Proteins matching the same set of peptides:
2::A0A068EL77 **Mass:** 46093 **Score:** 54 **Matches:** 1(1) **Sequences:** 1(1)
>tr|A0A068EL77|A0A068EL77_9POXV Ankyrin repeat protein OS=Penguinpox virus OX=648998 GN=pepv_016 PE=4 SV=1
2::A0A1X9Q118 **Mass:** 56950 **Score:** 54 **Matches:** 1(1) **Sequences:** 1(1)
>tr|A0A1X9Q118|A0A1X9Q118_9VIRU Putative VP4 OS=Human gut microviridae SH-CHD8 OX=1986033 PE=4 SV=1

---

25. 2::A0A060D5Q0 **Mass:** 30617 **Score:** 53 **Matches:** 1(1) **Sequences:** 1(1) **emPAI:** 0.15
>tr|A0A060D5Q0|A0A060D5Q0_9CAUD Uncharacterized protein OS=Salmonella phage 9NA OX=1113547 GN=9NA_029 PE=4 SV=1

Query	Observed	Mr(expt)	Mr(calc)	ppm	Miss	Score	Expect	Rank	Unique	Peptide
9	351.7147	701.4149	701.4146	0.51	0	53	0.0017	1	U	K.LIMTPK.A

---

26. 2::A0A516L0H2 **Mass:** 13576 **Score:** 52 **Matches:** 1(1) **Sequences:** 1(1) **emPAI:** 0.36
>tr|A0A516L0H2|A0A516L0H2_9VIRU Uncharacterized protein OS=Prokaryotic dsDNA virus sp. OX=2591644 GN=GOVbin2950_25 PE=4 SV=1

Query	Observed	Mr(expt)	Mr(calc)	ppm	Miss	Score	Expect	Rank	Unique	Peptide
364	443.7665	885.5184	885.5171	1.53	0	52	0.0043	1	U	K.ALLEALEK.S

Proteins matching the same set of peptides:
2::A0A516L865 **Mass:** 13665 **Score:** 52 **Matches:** 1(1) **Sequences:** 1(1)
>tr|A0A516L865|A0A516L865_9VIRU Uncharacterized protein OS=Prokaryotic dsDNA virus sp. OX=2591644 GN=Unbinned4311contig1001_30 PE=4 SV=1
2::A0A516LUY4 **Mass:** 13201 **Score:** 52 **Matches:** 1(1) **Sequences:** 1(1)
>tr|A0A516LUY4|A0A516LUY4_9VIRU HTH_8 domain-containing protein OS=Prokaryotic dsDNA virus sp. OX=2591644 GN=Unbinned92contig1004_18 PE=4 SV=1

---

27. 2::A0A290FZR5 **Mass:** 18830 **Score:** 51 **Matches:** 1(1) **Sequences:** 1(1) **emPAI:** 0.25
>tr|A0A290FZR5|A0A290FZR5_9CAUD Uncharacterized protein OS=Caldibacillus phage CBP1 OX=2036768 PE=4 SV=1

Query	Observed	Mr(expt)	Mr(calc)	ppm	Miss	Score	Expect	Rank	Unique	Peptide
593	498.2635	994.5124	994.5123	0.05	1	51	0.0038	1	U	K.KIEWYEK.A

---

28. 2::A0A023T3C3 **Mass:** 57721 **Score:** 50 **Matches:** 1(1) **Sequences:** 1(1) **emPAI:** 0.08
>tr|A0A023T3C3|A0A023T3C3_9POTV Polyprotein (Fragment) OS=Zucchini yellow mosaic virus OX=12232 PE=3 SV=1

Query	Observed	Mr(expt)	Mr(calc)	ppm	Miss	Score	Expect	Rank	Unique	Peptide
114	384.2169	766.4192	766.4225	-4.26	1	50	0.0039	1	U	R.KLYTDK.G

Proteins matching the same set of peptides:
2::A0A023Y915 **Mass:** 338850 **Score:** 50 **Matches:** 1(1) **Sequences:** 1(1)
>tr|A0A023Y915|A0A023Y915_9POTV Genome polyprotein (Fragment) OS=Hardenbergia mosaic virus OX=409486 PE=3 SV=1
2::A0A023Y9B4 **Mass:** 353665 **Score:** 50 **Matches:** 1(1) **Sequences:** 1(1)
>tr|A0A023Y9B4|A0A023Y9B4_9POTV Genome polyprotein OS=Hardenbergia mosaic virus OX=409486 PE=3 SV=1
2::A0A023Y9I8 **Mass:** 353635 **Score:** 50 **Matches:** 1(1) **Sequences:** 1(1)
>tr|A0A023Y9I8|A0A023Y9I8_9POTV Genome polyprotein OS=Hardenbergia mosaic virus OX=409486 PE=3 SV=1
2::A0A023Y9J6 **Mass:** 353572 **Score:** 50 **Matches:** 1(1) **Sequences:** 1(1)
>tr|A0A023Y9J6|A0A023Y9J6_9POTV Genome polyprotein OS=Hardenbergia mosaic virus OX=409486 PE=3 SV=1
2::A0A023Y9S1 **Mass:** 353620 **Score:** 50 **Matches:** 1(1) **Sequences:** 1(1)
>tr|A0A023Y9S1|A0A023Y9S1_9POTV Genome polyprotein OS=Hardenbergia mosaic virus OX=409486 PE=3 SV=1
2::A0A059TCI5 **Mass:** 9373 **Score:** 50 **Matches:** 1(1) **Sequences:** 1(1)
>tr|A0A059TCI5|A0A059TCI5_9POTV NIb replicase (Fragment) OS=Zucchini yellow mosaic virus OX=12232 PE=4 SV=1
2::A0A060KQ18 **Mass:** 36414 **Score:** 50 **Matches:** 1(1) **Sequences:** 1(1)
>tr|A0A060KQ18|A0A060KQ18_9POTV Polyprotein (Fragment) OS=Zucchini yellow mosaic virus [India:Ludhiana:Bitter gourd:2013] OX=1506186 PE=3 SV=1
2::A0A068FLC0 **Mass:** 36414 **Score:** 50 **Matches:** 1(1) **Sequences:** 1(1)
>tr|A0A068FLC0|A0A068FLC0_9POTV Polyprotein (Fragment) OS=Zucchini yellow mosaic virus OX=12232 PE=3 SV=1
2::A0A075IAX5 **Mass:** 19244 **Score:** 50 **Matches:** 1(1) **Sequences:** 1(1)
>tr|A0A075IAX5|A0A075IAX5_9POTV Polyprotein (Fragment) OS=Zucchini yellow mosaic virus OX=12232 PE=4 SV=1
2::A0A075IAY1 **Mass:** 19201 **Score:** 50 **Matches:** 1(1) **Sequences:** 1(1)
>tr|A0A075IAY1|A0A075IAY1_9POTV Polyprotein (Fragment) OS=Zucchini yellow mosaic virus OX=12232 PE=4 SV=1
2::A0A075IAY8 **Mass:** 19201 **Score:** 50 **Matches:** 1(1) **Sequences:** 1(1)
>tr|A0A075IAY8|A0A075IAY8_9POTV Polyprotein (Fragment) OS=Zucchini yellow mosaic virus OX=12232 PE=4 SV=1
2::A0A075IAZ5 **Mass:** 19201 **Score:** 50 **Matches:** 1(1) **Sequences:** 1(1)
>tr|A0A075IAZ5|A0A075IAZ5_9POTV Polyprotein (Fragment) OS=Zucchini yellow mosaic virus OX=12232 PE=4 SV=1
2::A0A075ID69 **Mass:** 19172 **Score:** 50 **Matches:** 1(1) **Sequences:** 1(1)
>tr|A0A075ID69|A0A075ID69_9POTV Polyprotein (Fragment) OS=Zucchini yellow mosaic virus OX=12232 PE=4 SV=1
2::A0A075ID74 **Mass:** 19201 **Score:** 50 **Matches:** 1(1) **Sequences:** 1(1)
>tr|A0A075ID74|A0A075ID74_9POTV Polyprotein (Fragment) OS=Zucchini yellow mosaic virus OX=12232 PE=4 SV=1
2::A0A075ID81 **Mass:** 19172 **Score:** 50 **Matches:** 1(1) **Sequences:** 1(1)
>tr|A0A075ID81|A0A075ID81_9POTV Polyprotein (Fragment) OS=Zucchini yellow mosaic virus OX=12232 PE=4 SV=1
2::A0A075IDE7 **Mass:** 19185 **Score:** 50 **Matches:** 1(1) **Sequences:** 1(1)
>tr|A0A075IDE7|A0A075IDE7_9POTV Polyprotein (Fragment) OS=Zucchini yellow mosaic virus OX=12232 PE=4 SV=1
2::A0A075IDF1 **Mass:** 19201 **Score:** 50 **Matches:** 1(1) **Sequences:** 1(1)
>tr|A0A075IDF1|A0A075IDF1_9POTV Polyprotein (Fragment) OS=Zucchini yellow mosaic virus OX=12232 PE=4 SV=1
2::A0A075IDF5 **Mass:** 19259 **Score:** 50 **Matches:** 1(1) **Sequences:** 1(1)
>tr|A0A075IDF5|A0A075IDF5_9POTV Polyprotein (Fragment) OS=Zucchini yellow mosaic virus OX=12232 PE=4 SV=1
2::A0A075IDF9 **Mass:** 19201 **Score:** 50 **Matches:** 1(1) **Sequences:** 1(1)
>tr|A0A075IDF9|A0A075IDF9_9POTV Polyprotein (Fragment) OS=Zucchini yellow mosaic virus OX=12232 PE=4 SV=1
2::A0A075IFT5 **Mass:** 19201 **Score:** 50 **Matches:** 1(1) **Sequences:** 1(1)
>tr|A0A075IFT5|A0A075IFT5_9POTV Polyprotein (Fragment) OS=Zucchini yellow mosaic virus OX=12232 PE=4 SV=1
2::A0A075IFU0 **Mass:** 19172 **Score:** 50 **Matches:** 1(1) **Sequences:** 1(1)
>tr|A0A075IFU0|A0A075IFU0_9POTV Polyprotein (Fragment) OS=Zucchini yellow mosaic virus OX=12232 PE=4 SV=1
2::A0A075IFU7 **Mass:** 19172 **Score:** 50 **Matches:** 1(1) **Sequences:** 1(1)
>tr|A0A075IFU7|A0A075IFU7_9POTV Polyprotein (Fragment) OS=Zucchini yellow mosaic virus OX=12232 PE=4 SV=1
2::A0A075IFV4 **Mass:** 19201 **Score:** 50 **Matches:** 1(1) **Sequences:** 1(1)
>tr|A0A075IFV4|A0A075IFV4_9POTV Polyprotein (Fragment) OS=Zucchini yellow mosaic virus OX=12232 PE=4 SV=1
2::A0A075IHL7 **Mass:** 19200 **Score:** 50 **Matches:** 1(1) **Sequences:** 1(1)
>tr|A0A075IHL7|A0A075IHL7_9POTV Polyprotein (Fragment) OS=Zucchini yellow mosaic virus OX=12232 PE=4 SV=1
2::A0A075IHM2 **Mass:** 19201 **Score:** 50 **Matches:** 1(1) **Sequences:** 1(1)
>tr|A0A075IHM2|A0A075IHM2_9POTV Polyprotein (Fragment) OS=Zucchini yellow mosaic virus OX=12232 PE=4 SV=1
2::A0A075IHM6 **Mass:** 19172 **Score:** 50 **Matches:** 1(1) **Sequences:** 1(1)
>tr|A0A075IHM6|A0A075IHM6_9POTV Polyprotein (Fragment) OS=Zucchini yellow mosaic virus OX=12232 PE=4 SV=1
2::A0A076JTP5 **Mass:** 35903 **Score:** 50 **Matches:** 1(1) **Sequences:** 1(1)
>tr|A0A076JTP5|A0A076JTP5_9POTV Polyprotein (Fragment) OS=Zucchini yellow mosaic virus OX=12232 PE=3 SV=1
2::A0A076JV30 **Mass:** 35877 **Score:** 50 **Matches:** 1(1) **Sequences:** 1(1)
>tr|A0A076JV30|A0A076JV30_9POTV Polyprotein (Fragment) OS=Zucchini yellow mosaic virus OX=12232 PE=3 SV=1
2::A0A076JYM3 **Mass:** 35903 **Score:** 50 **Matches:** 1(1) **Sequences:** 1(1)
>tr|A0A076JYM3|A0A076JYM3_9POTV Polyprotein (Fragment) OS=Zucchini yellow mosaic virus OX=12232 PE=3 SV=1
2::A0A076JZ29 **Mass:** 35877 **Score:** 50 **Matches:** 1(1) **Sequences:** 1(1)
>tr|A0A076JZ29|A0A076JZ29_9POTV Polyprotein (Fragment) OS=Zucchini yellow mosaic virus OX=12232 PE=3 SV=1
2::A0A076JZZ4 **Mass:** 35877 **Score:** 50 **Matches:** 1(1) **Sequences:** 1(1)
>tr|A0A076JZZ4|A0A076JZZ4_9POTV Polyprotein (Fragment) OS=Zucchini yellow mosaic virus OX=12232 PE=3 SV=1
2::A0A077D1B6 **Mass:** 35705 **Score:** 50 **Matches:** 1(1) **Sequences:** 1(1)
>tr|A0A077D1B6|A0A077D1B6_9POTV Polyprotein (Fragment) OS=Zucchini yellow mosaic virus OX=12232 PE=3 SV=1
2::A0A096ZXG1 **Mass:** 353913 **Score:** 50 **Matches:** 1(1) **Sequences:** 1(1)
>tr|A0A096ZXG1|A0A096ZXG1_9POTV Genome polyprotein OS=Zucchini yellow mosaic virus OX=12232 PE=3 SV=1
2::A0A0A0N129 **Mass:** 55787 **Score:** 50 **Matches:** 1(1) **Sequences:** 1(1)
>tr|A0A0A0N129|A0A0A0N129_9POTV Polyprotein (Fragment) OS=Zucchini yellow mosaic virus OX=12232 PE=3 SV=1
2::A0A0A7DTD7 **Mass:** 353576 **Score:** 50 **Matches:** 1(1) **Sequences:** 1(1)

>tr|A0A0A7DTD7|A0A0A7DTD7_9POTV Genome polyprotein OS=Zucchini yellow mosaic virus OX=12232 PE=3 SV=1
2::A0A0A7DUE2 **Mass:** 353638 **Score:** 50 **Matches:** 1(1) **Sequences:** 1(1)
>tr|A0A0A7DUE2|A0A0A7DUE2_9POTV Genome polyprotein OS=Zucchini yellow mosaic virus OX=12232 PE=3 SV=1
2::A0A0B4VLK2 **Mass:** 249965 **Score:** 50 **Matches:** 1(1) **Sequences:** 1(1)
>tr|A0A0B4VLK2|A0A0B4VLK2_9POTV Genome polyprotein (Fragment) OS=Telosma mosaic virus OX=400394 PE=3 SV=1
2::A0A0M3LAJ6 **Mass:** 353886 **Score:** 50 **Matches:** 1(1) **Sequences:** 1(1)
>tr|A0A0M3LAJ6|A0A0M3LAJ6_9POTV Genome polyprotein OS=Zucchini yellow mosaic virus OX=12232 PE=3 SV=1
2::A0A0S1VVW0 **Mass:** 353472 **Score:** 50 **Matches:** 1(1) **Sequences:** 1(1)
>tr|A0A0S1VVW0|A0A0S1VVW0_9POTV Genome polyprotein OS=Zucchini yellow mosaic virus OX=12232 PE=3 SV=1
2::A0A0U2D8X3 **Mass:** 55384 **Score:** 50 **Matches:** 1(1) **Sequences:** 1(1)
>tr|A0A0U2D8X3|A0A0U2D8X3_9POTY Polyprotein (Fragment) OS=Paris vietnamensis mottle virus OX=1675556 PE=4 SV=1
2::A0A0U2U076 **Mass:** 45417 **Score:** 50 **Matches:** 1(1) **Sequences:** 1(1)
>tr|A0A0U2U076|A0A0U2U076_9POTV Polyprotein (Fragment) OS=Zucchini yellow mosaic virus OX=12232 PE=3 SV=2
2::A0A125SLF8 **Mass:** 353342 **Score:** 50 **Matches:** 1(1) **Sequences:** 1(1)
>tr|A0A125SLF8|A0A125SLF8_9POTV Genome polyprotein OS=Zucchini yellow mosaic virus OX=12232 PE=3 SV=1
2::A0A140DJD3 **Mass:** 370332 **Score:** 50 **Matches:** 1(1) **Sequences:** 1(1)
>tr|A0A140DJD3|A0A140DJD3_9POTV Genome polyprotein OS=East Asian Passiflora virus OX=341167 PE=3 SV=1
2::A0A141NWG3 **Mass:** 18769 **Score:** 50 **Matches:** 1(1) **Sequences:** 1(1)
>tr|A0A141NWG3|A0A141NWG3_9POTV Polyprotein (Fragment) OS=Zucchini yellow mosaic virus OX=12232 PE=4 SV=1
2::A0A141NWG4 **Mass:** 19896 **Score:** 50 **Matches:** 1(1) **Sequences:** 1(1)
>tr|A0A141NWG4|A0A141NWG4_9POTV Polyprotein (Fragment) OS=Zucchini yellow mosaic virus OX=12232 PE=4 SV=1
2::A0A141NWG5 **Mass:** 19896 **Score:** 50 **Matches:** 1(1) **Sequences:** 1(1)
>tr|A0A141NWG5|A0A141NWG5_9POTV Polyprotein (Fragment) OS=Zucchini yellow mosaic virus OX=12232 PE=4 SV=1
2::A0A193H6U8 **Mass:** 54932 **Score:** 50 **Matches:** 1(1) **Sequences:** 1(1)
>tr|A0A193H6U8|A0A193H6U8_9POTV Polyprotein (Fragment) OS=Zucchini yellow mosaic virus OX=12232 PE=3 SV=1
2::A0A1B0YWM4 **Mass:** 353884 **Score:** 50 **Matches:** 1(1) **Sequences:** 1(1)
>tr|A0A1B0YWM4|A0A1B0YWM4_9POTV Genome polyprotein OS=Bean common mosaic necrosis virus OX=43240 PE=3 SV=1
2::A0A1B1W1W3 **Mass:** 353686 **Score:** 50 **Matches:** 1(1) **Sequences:** 1(1)
>tr|A0A1B1W1W3|A0A1B1W1W3_9POTV Genome polyprotein OS=Zucchini yellow mosaic virus OX=12232 PE=3 SV=1
2::A0A1D8PUQ6 **Mass:** 353324 **Score:** 50 **Matches:** 1(1) **Sequences:** 1(1)
>tr|A0A1D8PUQ6|A0A1D8PUQ6_9POTV Genome polyprotein OS=Zucchini yellow mosaic virus OX=12232 PE=3 SV=1
2::A0A1L3KNQ6 **Mass:** 353579 **Score:** 50 **Matches:** 1(1) **Sequences:** 1(1)
>tr|A0A1L3KNQ6|A0A1L3KNQ6_9POTV Genome polyprotein OS=Zucchini yellow mosaic virus OX=12232 PE=3 SV=1
2::A0A1L3KNV2 **Mass:** 353384 **Score:** 50 **Matches:** 1(1) **Sequences:** 1(1)
>tr|A0A1L3KNV2|A0A1L3KNV2_9POTV Genome polyprotein OS=Zucchini yellow mosaic virus OX=12232 PE=3 SV=1
2::A0A1S6KL15 **Mass:** 53618 **Score:** 50 **Matches:** 1(1) **Sequences:** 1(1)
>tr|A0A1S6KL15|A0A1S6KL15_9POTV Polyprotein (Fragment) OS=East Asian Passiflora virus OX=341167 PE=3 SV=1
2::A0A1V0CMI6 **Mass:** 37647 **Score:** 50 **Matches:** 1(1) **Sequences:** 1(1)
>tr|A0A1V0CMI6|A0A1V0CMI6_9POTV Polyprotein (Fragment) OS=Zucchini yellow mosaic virus OX=12232 PE=3 SV=1
2::A0A1V0CMI7 **Mass:** 37647 **Score:** 50 **Matches:** 1(1) **Sequences:** 1(1)
>tr|A0A1V0CMI7|A0A1V0CMI7_9POTV Polyprotein (Fragment) OS=Zucchini yellow mosaic virus OX=12232 PE=3 SV=1
2::A0A1V0CMJ3 **Mass:** 37647 **Score:** 50 **Matches:** 1(1) **Sequences:** 1(1)
>tr|A0A1V0CMJ3|A0A1V0CMJ3_9POTV Polyprotein (Fragment) OS=Zucchini yellow mosaic virus OX=12232 PE=3 SV=1
2::A0A1V0CMJ4 **Mass:** 37647 **Score:** 50 **Matches:** 1(1) **Sequences:** 1(1)
>tr|A0A1V0CMJ4|A0A1V0CMJ4_9POTV Polyprotein (Fragment) OS=Zucchini yellow mosaic virus OX=12232 PE=3 SV=1
2::A0A1V0CMJ5 **Mass:** 37647 **Score:** 50 **Matches:** 1(1) **Sequences:** 1(1)
>tr|A0A1V0CMJ5|A0A1V0CMJ5_9POTV Polyprotein (Fragment) OS=Zucchini yellow mosaic virus OX=12232 PE=3 SV=1
2::A0A1V0CMJ7 **Mass:** 37647 **Score:** 50 **Matches:** 1(1) **Sequences:** 1(1)
>tr|A0A1V0CMJ7|A0A1V0CMJ7_9POTV Polyprotein (Fragment) OS=Zucchini yellow mosaic virus OX=12232 PE=3 SV=1
2::A0A1W5YP76 **Mass:** 353845 **Score:** 50 **Matches:** 1(1) **Sequences:** 1(1)
>tr|A0A1W5YP76|A0A1W5YP76_9POTV Genome polyprotein OS=Bean common mosaic necrosis virus OX=43240 PE=3 SV=1
2::A0A1W5YP77 **Mass:** 353667 **Score:** 50 **Matches:** 1(1) **Sequences:** 1(1)
>tr|A0A1W5YP77|A0A1W5YP77_9POTV Genome polyprotein OS=Bean common mosaic necrosis virus OX=43240 PE=3 SV=1
2::A0A1W5YP86 **Mass:** 353762 **Score:** 50 **Matches:** 1(1) **Sequences:** 1(1)
>tr|A0A1W5YP86|A0A1W5YP86_9POTV Genome polyprotein OS=Bean common mosaic necrosis virus OX=43240 PE=3 SV=1
2::A0A1W6LXG9 **Mass:** 353544 **Score:** 50 **Matches:** 1(1) **Sequences:** 1(1)
>tr|A0A1W6LXG9|A0A1W6LXG9_9POTV Genome polyprotein OS=Zucchini yellow mosaic virus OX=12232 PE=3 SV=1
2::A0A1Y0EVC3 **Mass:** 353939 **Score:** 50 **Matches:** 1(1) **Sequences:** 1(1)
>tr|A0A1Y0EVC3|A0A1Y0EVC3_9POTV Genome polyprotein OS=Hardenbergia mosaic virus OX=409486 PE=3 SV=1
2::A0A1Z1VXG1 **Mass:** 353428 **Score:** 50 **Matches:** 1(1) **Sequences:** 1(1)
>tr|A0A1Z1VXG1|A0A1Z1VXG1_9POTV Genome polyprotein OS=Zucchini yellow mosaic virus OX=12232 PE=3 SV=1
2::A0A219YWS6 **Mass:** 353932 **Score:** 50 **Matches:** 1(1) **Sequences:** 1(1)
>tr|A0A219YWS6|A0A219YWS6_9POTV Genome polyprotein OS=Zucchini yellow mosaic virus OX=12232 PE=3 SV=1
2::A0A240F8S3 **Mass:** 353953 **Score:** 50 **Matches:** 1(1) **Sequences:** 1(1)
>tr|A0A240F8S3|A0A240F8S3_9POTV Genome polyprotein OS=Zucchini yellow mosaic virus OX=12232 PE=3 SV=1
2::A0A240F8S4 **Mass:** 353750 **Score:** 50 **Matches:** 1(1) **Sequences:** 1(1)
>tr|A0A240F8S4|A0A240F8S4_9POTV Genome polyprotein OS=Zucchini yellow mosaic virus OX=12232 PE=3 SV=1
2::A0A240F8S5 **Mass:** 353620 **Score:** 50 **Matches:** 1(1) **Sequences:** 1(1)
>tr|A0A240F8S5|A0A240F8S5_9POTV Genome polyprotein OS=Zucchini yellow mosaic virus OX=12232 PE=3 SV=1
2::A0A240F8S6 **Mass:** 356286 **Score:** 50 **Matches:** 1(1) **Sequences:** 1(1)
>tr|A0A240F8S6|A0A240F8S6_9POTV Genome polyprotein OS=Zucchini yellow mosaic virus OX=12232 PE=3 SV=1
2::A0A240F8S7 **Mass:** 353331 **Score:** 50 **Matches:** 1(1) **Sequences:** 1(1)
>tr|A0A240F8S7|A0A240F8S7_9POTV Genome polyprotein OS=Zucchini yellow mosaic virus OX=12232 PE=3 SV=1
2::A0A240F8S8 **Mass:** 353319 **Score:** 50 **Matches:** 1(1) **Sequences:** 1(1)
>tr|A0A240F8S8|A0A240F8S8_9POTV Genome polyprotein OS=Zucchini yellow mosaic virus OX=12232 PE=3 SV=1
2::A0A240F8S9 **Mass:** 353271 **Score:** 50 **Matches:** 1(1) **Sequences:** 1(1)
>tr|A0A240F8S9|A0A240F8S9_9POTV Genome polyprotein OS=Zucchini yellow mosaic virus OX=12232 PE=3 SV=1
2::A0A240F8T2 **Mass:** 353801 **Score:** 50 **Matches:** 1(1) **Sequences:** 1(1)
>tr|A0A240F8T2|A0A240F8T2_9POTV Genome polyprotein OS=Zucchini yellow mosaic virus OX=12232 PE=3 SV=1
2::A0A2H4V8D2 **Mass:** 355163 **Score:** 50 **Matches:** 1(1) **Sequences:** 1(1)
>tr|A0A2H4V8D2|A0A2H4V8D2_9POTV Genome polyprotein OS=Paris mosaic necrosis virus OX=2058396 PE=3 SV=1
2::A0A2H5CUH8 **Mass:** 63646 **Score:** 50 **Matches:** 1(1) **Sequences:** 1(1)
>tr|A0A2H5CUH8|A0A2H5CUH8_9POTV Polyprotein (Fragment) OS=Zucchini yellow mosaic virus OX=12232 PE=3 SV=1
2::A0A2I7VT90 **Mass:** 16801 **Score:** 50 **Matches:** 1(1) **Sequences:** 1(1)
>tr|A0A2I7VT90|A0A2I7VT90_9POTV Coat protein (Fragment) OS=Zucchini yellow mosaic virus OX=12232 PE=4 SV=1
2::A0A2I7VT96 **Mass:** 16801 **Score:** 50 **Matches:** 1(1) **Sequences:** 1(1)
>tr|A0A2I7VT96|A0A2I7VT96_9POTV Coat protein (Fragment) OS=Zucchini yellow mosaic virus OX=12232 PE=4 SV=1
2::A0A2I7VTA4 **Mass:** 16786 **Score:** 50 **Matches:** 1(1) **Sequences:** 1(1)
>tr|A0A2I7VTA4|A0A2I7VTA4_9POTV Coat protein (Fragment) OS=Zucchini yellow mosaic virus OX=12232 PE=4 SV=1
2::A0A2K9RF70 **Mass:** 370480 **Score:** 50 **Matches:** 1(1) **Sequences:** 1(1)
>tr|A0A2K9RF70|A0A2K9RF70_9POTV Genome polyprotein OS=East Asian Passiflora virus OX=341167 PE=3 SV=1
2::A0A2P1ANH7 **Mass:** 20310 **Score:** 50 **Matches:** 1(1) **Sequences:** 1(1)
>tr|A0A2P1ANH7|A0A2P1ANH7_9POTV Coat protein (Fragment) OS=Zucchini yellow mosaic virus OX=12232 PE=2 SV=1
2::A0A2P1ANI0 **Mass:** 20310 **Score:** 50 **Matches:** 1(1) **Sequences:** 1(1)
>tr|A0A2P1ANI0|A0A2P1ANI0_9POTV Coat protein (Fragment) OS=Zucchini yellow mosaic virus OX=12232 PE=2 SV=1
2::A0A2P1ANI1 **Mass:** 20337 **Score:** 50 **Matches:** 1(1) **Sequences:** 1(1)
>tr|A0A2P1ANI1|A0A2P1ANI1_9POTV Coat protein (Fragment) OS=Zucchini yellow mosaic virus OX=12232 PE=2 SV=1
2::A0A2P1ANI4 **Mass:** 20310 **Score:** 50 **Matches:** 1(1) **Sequences:** 1(1)
>tr|A0A2P1ANI4|A0A2P1ANI4_9POTV Coat protein (Fragment) OS=Zucchini yellow mosaic virus OX=12232 PE=2 SV=1
2::A0A2P1ANI7 **Mass:** 20310 **Score:** 50 **Matches:** 1(1) **Sequences:** 1(1)
>tr|A0A2P1ANI7|A0A2P1ANI7_9POTV Coat protein (Fragment) OS=Zucchini yellow mosaic virus OX=12232 PE=2 SV=1
2::A0A2P1ANJ1 **Mass:** 20337 **Score:** 50 **Matches:** 1(1) **Sequences:** 1(1)
>tr|A0A2P1ANJ1|A0A2P1ANJ1_9POTV Coat protein (Fragment) OS=Zucchini yellow mosaic virus OX=12232 PE=2 SV=1
2::A0A2U7SN92 **Mass:** 341953 **Score:** 50 **Matches:** 1(1) **Sequences:** 1(1)
>tr|A0A2U7SN92|A0A2U7SN92_9POTV Genome polyprotein (Fragment) OS=Bean common mosaic necrosis virus OX=43240 PE=3 SV=1
2::A0A2U7SNA8 **Mass:** 342125 **Score:** 50 **Matches:** 1(1) **Sequences:** 1(1)
>tr|A0A2U7SNA8|A0A2U7SNA8_9POTV Genome polyprotein (Fragment) OS=Bean common mosaic necrosis virus OX=43240 PE=3 SV=1
2::A0A2U7SNC7 **Mass:** 342025 **Score:** 50 **Matches:** 1(1) **Sequences:** 1(1)
>tr|A0A2U7SNC7|A0A2U7SNC7_9POTV Polyprotein (Fragment) OS=Bean common mosaic necrosis virus OX=43240 PE=3 SV=1
2::A0A2U7SSP6 **Mass:** 342175 **Score:** 50 **Matches:** 1(1) **Sequences:** 1(1)

>tr|A0A2U7SSP6|A0A2U7SSP6_9POTV Genome polyprotein (Fragment) OS=Bean common mosaic necrosis virus OX=43240 PE=3 SV=1
2::A0A2U7STH4 **Mass:** 341947 **Score:** 50 **Matches:** 1(1) **Sequences:** 1(1)
>tr|A0A2U7STH4|A0A2U7STH4_9POTV Genome polyprotein (Fragment) OS=Bean common mosaic necrosis virus OX=43240 PE=3 SV=1
2::A0A2U7STW1 **Mass:** 342123 **Score:** 50 **Matches:** 1(1) **Sequences:** 1(1)
>tr|A0A2U7STW1|A0A2U7STW1_9POTV Polyprotein (Fragment) OS=Bean common mosaic necrosis virus OX=43240 PE=3 SV=1
2::A0A2U7SU55 **Mass:** 341750 **Score:** 50 **Matches:** 1(1) **Sequences:** 1(1)
>tr|A0A2U7SU55|A0A2U7SU55_9POTV Genome polyprotein (Fragment) OS=Bean common mosaic necrosis virus OX=43240 PE=3 SV=1
2::A0A2U7SU65 **Mass:** 353875 **Score:** 50 **Matches:** 1(1) **Sequences:** 1(1)
>tr|A0A2U7SU65|A0A2U7SU65_9POTV Polyprotein OS=Bean common mosaic necrosis virus OX=43240 PE=3 SV=1
2::A0A2U7SUG8 **Mass:** 342064 **Score:** 50 **Matches:** 1(1) **Sequences:** 1(1)
>tr|A0A2U7SUG8|A0A2U7SUG8_9POTV Genome polyprotein (Fragment) OS=Bean common mosaic necrosis virus OX=43240 PE=3 SV=1
2::A0A2U7SUG9 **Mass:** 342168 **Score:** 50 **Matches:** 1(1) **Sequences:** 1(1)
>tr|A0A2U7SUG9|A0A2U7SUG9_9POTV Genome polyprotein (Fragment) OS=Bean common mosaic necrosis virus OX=43240 PE=3 SV=1
2::A0A2U7SVA1 **Mass:** 341947 **Score:** 50 **Matches:** 1(1) **Sequences:** 1(1)
>tr|A0A2U7SVA1|A0A2U7SVA1_9POTV Genome polyprotein (Fragment) OS=Bean common mosaic necrosis virus OX=43240 PE=3 SV=1
2::A0A345ZA01 **Mass:** 353404 **Score:** 50 **Matches:** 1(1) **Sequences:** 1(1)
>tr|A0A345ZA01|A0A345ZA01_9POTV Genome polyprotein OS=Zucchini yellow mosaic virus OX=12232 PE=3 SV=1
2::A0A346HHC7 **Mass:** 363827 **Score:** 50 **Matches:** 1(1) **Sequences:** 1(1)
>tr|A0A346HHC7|A0A346HHC7_9POTV Genome polyprotein OS=Telosma mosaic virus OX=400394 PE=3 SV=1
2::A0A385HCU2 **Mass:** 18759 **Score:** 50 **Matches:** 1(1) **Sequences:** 1(1)
>tr|A0A385HCU2|A0A385HCU2_9POTV Coat protein (Fragment) OS=Zucchini yellow mosaic virus OX=12232 PE=4 SV=1
2::A0A385HCU3 **Mass:** 18887 **Score:** 50 **Matches:** 1(1) **Sequences:** 1(1)
>tr|A0A385HCU3|A0A385HCU3_9POTV Coat protein (Fragment) OS=Zucchini yellow mosaic virus OX=12232 PE=4 SV=1
2::A0A385HCU4 **Mass:** 18760 **Score:** 50 **Matches:** 1(1) **Sequences:** 1(1)
>tr|A0A385HCU4|A0A385HCU4_9POTV Coat protein (Fragment) OS=Zucchini yellow mosaic virus OX=12232 PE=4 SV=1
2::A0A385HCU5 **Mass:** 18759 **Score:** 50 **Matches:** 1(1) **Sequences:** 1(1)
>tr|A0A385HCU5|A0A385HCU5_9POTV Coat protein (Fragment) OS=Zucchini yellow mosaic virus OX=12232 PE=4 SV=1
2::A0A385J6S4 **Mass:** 353533 **Score:** 50 **Matches:** 1(1) **Sequences:** 1(1)
>tr|A0A385J6S4|A0A385J6S4_9POTV Genome polyprotein OS=Zucchini yellow mosaic virus OX=12232 PE=3 SV=1
2::A0A385J6S5 **Mass:** 353499 **Score:** 50 **Matches:** 1(1) **Sequences:** 1(1)
>tr|A0A385J6S5|A0A385J6S5_9POTV Genome polyprotein OS=Zucchini yellow mosaic virus OX=12232 PE=3 SV=1
2::A0A385J702 **Mass:** 353480 **Score:** 50 **Matches:** 1(1) **Sequences:** 1(1)
>tr|A0A385J702|A0A385J702_9POTV Genome polyprotein OS=Zucchini yellow mosaic virus OX=12232 PE=3 SV=1
2::A0A386JM83 **Mass:** 40500 **Score:** 50 **Matches:** 1(1) **Sequences:** 1(1)
>tr|A0A386JM83|A0A386JM83_9POTV Polyprotein (Fragment) OS=Bean common mosaic necrosis virus OX=43240 PE=3 SV=1
2::A0A386JM89 **Mass:** 17479 **Score:** 50 **Matches:** 1(1) **Sequences:** 1(1)
>tr|A0A386JM89|A0A386JM89_9POTV Polyprotein (Fragment) OS=Bean common mosaic necrosis virus OX=43240 PE=3 SV=1
2::A0A386QWK2 **Mass:** 353681 **Score:** 50 **Matches:** 1(1) **Sequences:** 1(1)
>tr|A0A386QWK2|A0A386QWK2_9POTV Genome polyprotein OS=Bean common mosaic necrosis virus OX=43240 PE=3 SV=2
2::A0A386QWK6 **Mass:** 353769 **Score:** 50 **Matches:** 1(1) **Sequences:** 1(1)
>tr|A0A386QWK6|A0A386QWK6_9POTV Genome polyprotein OS=Bean common mosaic necrosis virus OX=43240 PE=3 SV=2
2::A0A386QWK9 **Mass:** 353770 **Score:** 50 **Matches:** 1(1) **Sequences:** 1(1)
>tr|A0A386QWK9|A0A386QWK9_9POTV Genome polyprotein OS=Bean common mosaic necrosis virus OX=43240 PE=3 SV=2
2::A0A386QWL9 **Mass:** 353688 **Score:** 50 **Matches:** 1(1) **Sequences:** 1(1)
>tr|A0A386QWL9|A0A386QWL9_9POTV Genome polyprotein OS=Bean common mosaic necrosis virus OX=43240 PE=3 SV=1
2::A0A386QWM4 **Mass:** 353775 **Score:** 50 **Matches:** 1(1) **Sequences:** 1(1)
>tr|A0A386QWM4|A0A386QWM4_9POTV Genome polyprotein OS=Bean common mosaic necrosis virus OX=43240 PE=3 SV=2
2::A0A386QWN5 **Mass:** 353796 **Score:** 50 **Matches:** 1(1) **Sequences:** 1(1)
>tr|A0A386QWN5|A0A386QWN5_9POTV Genome polyprotein OS=Bean common mosaic necrosis virus OX=43240 PE=3 SV=2
2::A0A386QWR5 **Mass:** 353781 **Score:** 50 **Matches:** 1(1) **Sequences:** 1(1)
>tr|A0A386QWR5|A0A386QWR5_9POTV Genome polyprotein OS=Bean common mosaic necrosis virus OX=43240 PE=3 SV=2
2::A0A3G2JU76 **Mass:** 36409 **Score:** 50 **Matches:** 1(1) **Sequences:** 1(1)
>tr|A0A3G2JU76|A0A3G2JU76_9POTV Polyprotein (Fragment) OS=Zucchini yellow mosaic virus OX=12232 PE=3 SV=1
2::A0A3G4YJQ6 **Mass:** 355458 **Score:** 50 **Matches:** 1(1) **Sequences:** 1(1)
>tr|A0A3G4YJQ6|A0A3G4YJQ6_9POTV Genome polyprotein OS=Ugandan Passiflora virus OX=693419 PE=3 SV=1
2::A0A3G4YK52 **Mass:** 353331 **Score:** 50 **Matches:** 1(1) **Sequences:** 1(1)
>tr|A0A3G4YK52|A0A3G4YK52_9POTV Genome polyprotein OS=Zucchini yellow mosaic virus OX=12232 PE=3 SV=1
2::A0A3G9CNF8 **Mass:** 60553 **Score:** 50 **Matches:** 1(1) **Sequences:** 1(1)
>tr|A0A3G9CNF8|A0A3G9CNF8_9POTV Polyprotein (Fragment) OS=Zucchini yellow mosaic virus OX=12232 PE=4 SV=1
2::A0A3Q8QFX4 **Mass:** 256490 **Score:** 50 **Matches:** 1(1) **Sequences:** 1(1)
>tr|A0A3Q8QFX4|A0A3Q8QFX4_9VIRU RNA-directed RNA polymerase L OS=Lassa mammarenavirus OX=11620 GN=L PE=3 SV=1
2::A0A3Q8QG58 **Mass:** 256246 **Score:** 50 **Matches:** 1(1) **Sequences:** 1(1)
>tr|A0A3Q8QG58|A0A3Q8QG58_9VIRU RNA-directed RNA polymerase L OS=Lassa mammarenavirus OX=11620 GN=L PE=3 SV=1
2::A0A3S5XD38 **Mass:** 353562 **Score:** 50 **Matches:** 1(1) **Sequences:** 1(1)
>tr|A0A3S5XD38|A0A3S5XD38_9POTV Genome polyprotein OS=Bean common mosaic necrosis virus OX=43240 PE=3 SV=1
2::A0A3S5XG80 **Mass:** 353579 **Score:** 50 **Matches:** 1(1) **Sequences:** 1(1)
>tr|A0A3S5XG80|A0A3S5XG80_9POTV Genome polyprotein OS=Bean common mosaic necrosis virus OX=43240 PE=3 SV=1
2::A0A3S7XU02 **Mass:** 353462 **Score:** 50 **Matches:** 1(1) **Sequences:** 1(1)
>tr|A0A3S7XU02|A0A3S7XU02_9POTV Genome polyprotein OS=Bean common mosaic necrosis virus OX=43240 PE=3 SV=1
2::A0A3S7XU03 **Mass:** 353532 **Score:** 50 **Matches:** 1(1) **Sequences:** 1(1)
>tr|A0A3S7XU03|A0A3S7XU03_9POTV Genome polyprotein OS=Bean common mosaic necrosis virus OX=43240 PE=3 SV=1
2::A0A3S7XU06 **Mass:** 354027 **Score:** 50 **Matches:** 1(1) **Sequences:** 1(1)
>tr|A0A3S7XU06|A0A3S7XU06_9POTV Genome polyprotein OS=Bean common mosaic necrosis virus OX=43240 PE=3 SV=1
2::A0A3S7XU09 **Mass:** 353705 **Score:** 50 **Matches:** 1(1) **Sequences:** 1(1)
>tr|A0A3S7XU09|A0A3S7XU09_9POTV Polyprotein OS=Bean common mosaic necrosis virus OX=43240 PE=3 SV=1
2::A0A3S8RDM0 **Mass:** 353628 **Score:** 50 **Matches:** 1(1) **Sequences:** 1(1)
>tr|A0A3S8RDM0|A0A3S8RDM0_9POTV Genome polyprotein OS=Zucchini yellow mosaic virus OX=12232 PE=3 SV=1
2::A0A3S9JKW3 **Mass:** 36521 **Score:** 50 **Matches:** 1(1) **Sequences:** 1(1)
>tr|A0A3S9JKW3|A0A3S9JKW3_9POTV Polyprotein (Fragment) OS=Telosma mosaic virus OX=400394 PE=3 SV=1
2::A0A410NSA6 **Mass:** 353146 **Score:** 50 **Matches:** 1(1) **Sequences:** 1(1)
>tr|A0A410NSA6|A0A410NSA6_9POTV Genome polyprotein OS=Zucchini yellow mosaic virus OX=12232 PE=3 SV=1
2::A0A410NSB2 **Mass:** 353721 **Score:** 50 **Matches:** 1(1) **Sequences:** 1(1)
>tr|A0A410NSB2|A0A410NSB2_9POTV Genome polyprotein OS=Zucchini yellow mosaic virus OX=12232 PE=3 SV=1
2::A0A410NSC9 **Mass:** 353571 **Score:** 50 **Matches:** 1(1) **Sequences:** 1(1)
>tr|A0A410NSC9|A0A410NSC9_9POTV Genome polyprotein OS=Zucchini yellow mosaic virus OX=12232 PE=3 SV=1
2::A0A410NSK3 **Mass:** 353605 **Score:** 50 **Matches:** 1(1) **Sequences:** 1(1)
>tr|A0A410NSK3|A0A410NSK3_9POTV Genome polyprotein OS=Zucchini yellow mosaic virus OX=12232 PE=3 SV=1
2::A0A411D738 **Mass:** 42235 **Score:** 50 **Matches:** 1(1) **Sequences:** 1(1)
>tr|A0A411D738|A0A411D738_9POTV Coat protein (Fragment) OS=East Asian Passiflora virus OX=341167 PE=3 SV=1
2::A0A411D739 **Mass:** 42317 **Score:** 50 **Matches:** 1(1) **Sequences:** 1(1)
>tr|A0A411D739|A0A411D739_9POTV Coat protein (Fragment) OS=East Asian Passiflora virus OX=341167 PE=3 SV=1
2::A0A411D740 **Mass:** 42207 **Score:** 50 **Matches:** 1(1) **Sequences:** 1(1)
>tr|A0A411D740|A0A411D740_9POTV Coat protein (Fragment) OS=East Asian Passiflora virus OX=341167 PE=3 SV=1
2::A0A411D746 **Mass:** 42323 **Score:** 50 **Matches:** 1(1) **Sequences:** 1(1)
>tr|A0A411D746|A0A411D746_9POTV Coat protein (Fragment) OS=East Asian Passiflora virus OX=341167 PE=3 SV=1
2::A0A481SS98 **Mass:** 19155 **Score:** 50 **Matches:** 1(1) **Sequences:** 1(1)
>tr|A0A481SS98|A0A481SS98_9POTV Polyprotein (Fragment) OS=Zucchini yellow mosaic virus OX=12232 PE=4 SV=1
2::A0A481SSB0 **Mass:** 19242 **Score:** 50 **Matches:** 1(1) **Sequences:** 1(1)
>tr|A0A481SSB0|A0A481SSB0_9POTV Polyprotein (Fragment) OS=Zucchini yellow mosaic virus OX=12232 PE=4 SV=1
2::A0A481SSB7 **Mass:** 19239 **Score:** 50 **Matches:** 1(1) **Sequences:** 1(1)
>tr|A0A481SSB7|A0A481SSB7_9POTV Polyprotein (Fragment) OS=Zucchini yellow mosaic virus OX=12232 PE=4 SV=1
2::A0A481SSD3 **Mass:** 19242 **Score:** 50 **Matches:** 1(1) **Sequences:** 1(1)
>tr|A0A481SSD3|A0A481SSD3_9POTV Polyprotein (Fragment) OS=Zucchini yellow mosaic virus OX=12232 PE=4 SV=1
2::A0A481SSD6 **Mass:** 19297 **Score:** 50 **Matches:** 1(1) **Sequences:** 1(1)
>tr|A0A481SSD6|A0A481SSD6_9POTV Polyprotein (Fragment) OS=Zucchini yellow mosaic virus OX=12232 PE=4 SV=1
2::A0A481SSF2 **Mass:** 19269 **Score:** 50 **Matches:** 1(1) **Sequences:** 1(1)
>tr|A0A481SSF2|A0A481SSF2_9POTV Polyprotein (Fragment) OS=Zucchini yellow mosaic virus OX=12232 PE=4 SV=1
2::A0A481SSF6 **Mass:** 19155 **Score:** 50 **Matches:** 1(1) **Sequences:** 1(1)
>tr|A0A481SSF6|A0A481SSF6_9POTV Polyprotein (Fragment) OS=Zucchini yellow mosaic virus OX=12232 PE=4 SV=1
2::A0A481SSF7 **Mass:** 19299 **Score:** 50 **Matches:** 1(1) **Sequences:** 1(1)

>tr|A0A481SSF7|A0A481SSF7_9POTV Polyprotein (Fragment) OS=Zucchini yellow mosaic virus OX=12232 PE=4 SV=1
2::A0A481SSG9 **Mass:** 19224 **Score:** 50 **Matches:** 1(1) **Sequences:** 1(1)
>tr|A0A481SSG9|A0A481SSG9_9POTV Polyprotein (Fragment) OS=Zucchini yellow mosaic virus OX=12232 PE=4 SV=1
2::A0A481SSH6 **Mass:** 19313 **Score:** 50 **Matches:** 1(1) **Sequences:** 1(1)
>tr|A0A481SSH6|A0A481SSH6_9POTV Polyprotein (Fragment) OS=Zucchini yellow mosaic virus OX=12232 PE=4 SV=1
2::A0A481SSJ0 **Mass:** 19323 **Score:** 50 **Matches:** 1(1) **Sequences:** 1(1)
>tr|A0A481SSJ0|A0A481SSJ0_9POTV Polyprotein (Fragment) OS=Zucchini yellow mosaic virus OX=12232 PE=4 SV=1
2::A0A481SSK3 **Mass:** 19299 **Score:** 50 **Matches:** 1(1) **Sequences:** 1(1)
>tr|A0A481SSK3|A0A481SSK3_9POTV Polyprotein (Fragment) OS=Zucchini yellow mosaic virus OX=12232 PE=4 SV=1
2::A0A481SSR6 **Mass:** 19224 **Score:** 50 **Matches:** 1(1) **Sequences:** 1(1)
>tr|A0A481SSR6|A0A481SSR6_9POTV Polyprotein (Fragment) OS=Zucchini yellow mosaic virus OX=12232 PE=4 SV=1
2::A0A481SSS7 **Mass:** 19272 **Score:** 50 **Matches:** 1(1) **Sequences:** 1(1)
>tr|A0A481SSS7|A0A481SSS7_9POTV Polyprotein (Fragment) OS=Zucchini yellow mosaic virus OX=12232 PE=4 SV=1
2::A0A481SSU2 **Mass:** 19323 **Score:** 50 **Matches:** 1(1) **Sequences:** 1(1)
>tr|A0A481SSU2|A0A481SSU2_9POTV Polyprotein (Fragment) OS=Zucchini yellow mosaic virus OX=12232 PE=4 SV=1
2::A0A481SSV7 **Mass:** 19255 **Score:** 50 **Matches:** 1(1) **Sequences:** 1(1)
>tr|A0A481SSV7|A0A481SSV7_9POTV Polyprotein (Fragment) OS=Zucchini yellow mosaic virus OX=12232 PE=4 SV=1
2::A0A481STL9 **Mass:** 19224 **Score:** 50 **Matches:** 1(1) **Sequences:** 1(1)
>tr|A0A481STL9|A0A481STL9_9POTV Polyprotein (Fragment) OS=Zucchini yellow mosaic virus OX=12232 PE=4 SV=1
2::A0A481STN3 **Mass:** 19355 **Score:** 50 **Matches:** 1(1) **Sequences:** 1(1)
>tr|A0A481STN3|A0A481STN3_9POTV Polyprotein (Fragment) OS=Zucchini yellow mosaic virus OX=12232 PE=4 SV=1
2::A0A481STN7 **Mass:** 19224 **Score:** 50 **Matches:** 1(1) **Sequences:** 1(1)
>tr|A0A481STN7|A0A481STN7_9POTV Polyprotein (Fragment) OS=Zucchini yellow mosaic virus OX=12232 PE=4 SV=1
2::A0A481STP6 **Mass:** 19225 **Score:** 50 **Matches:** 1(1) **Sequences:** 1(1)
>tr|A0A481STP6|A0A481STP6_9POTV Polyprotein (Fragment) OS=Zucchini yellow mosaic virus OX=12232 PE=4 SV=1
2::A0A481STP7 **Mass:** 19355 **Score:** 50 **Matches:** 1(1) **Sequences:** 1(1)
>tr|A0A481STP7|A0A481STP7_9POTV Polyprotein (Fragment) OS=Zucchini yellow mosaic virus OX=12232 PE=4 SV=1
2::A0A481STR0 **Mass:** 19337 **Score:** 50 **Matches:** 1(1) **Sequences:** 1(1)
>tr|A0A481STR0|A0A481STR0_9POTV Polyprotein (Fragment) OS=Zucchini yellow mosaic virus OX=12232 PE=4 SV=1
2::A0A481STR6 **Mass:** 19225 **Score:** 50 **Matches:** 1(1) **Sequences:** 1(1)
>tr|A0A481STR6|A0A481STR6_9POTV Polyprotein (Fragment) OS=Zucchini yellow mosaic virus OX=12232 PE=4 SV=1
2::A0A481STS2 **Mass:** 19224 **Score:** 50 **Matches:** 1(1) **Sequences:** 1(1)
>tr|A0A481STS2|A0A481STS2_9POTV Polyprotein (Fragment) OS=Zucchini yellow mosaic virus OX=12232 PE=4 SV=1
2::A0A481STT1 **Mass:** 19269 **Score:** 50 **Matches:** 1(1) **Sequences:** 1(1)
>tr|A0A481STT1|A0A481STT1_9POTV Polyprotein (Fragment) OS=Zucchini yellow mosaic virus OX=12232 PE=4 SV=1
2::A0A481STT4 **Mass:** 19242 **Score:** 50 **Matches:** 1(1) **Sequences:** 1(1)
>tr|A0A481STT4|A0A481STT4_9POTV Polyprotein (Fragment) OS=Zucchini yellow mosaic virus OX=12232 PE=4 SV=1
2::A0A481STU5 **Mass:** 19269 **Score:** 50 **Matches:** 1(1) **Sequences:** 1(1)
>tr|A0A481STU5|A0A481STU5_9POTV Polyprotein (Fragment) OS=Zucchini yellow mosaic virus OX=12232 PE=4 SV=1
2::A0A481STV7 **Mass:** 19269 **Score:** 50 **Matches:** 1(1) **Sequences:** 1(1)
>tr|A0A481STV7|A0A481STV7_9POTV Polyprotein (Fragment) OS=Zucchini yellow mosaic virus OX=12232 PE=4 SV=1
2::A0A481STX3 **Mass:** 19155 **Score:** 50 **Matches:** 1(1) **Sequences:** 1(1)
>tr|A0A481STX3|A0A481STX3_9POTV Polyprotein (Fragment) OS=Zucchini yellow mosaic virus OX=12232 PE=4 SV=1
2::A0A481STY7 **Mass:** 19242 **Score:** 50 **Matches:** 1(1) **Sequences:** 1(1)
>tr|A0A481STY7|A0A481STY7_9POTV Polyprotein (Fragment) OS=Zucchini yellow mosaic virus OX=12232 PE=4 SV=1
2::A0A481SU01 **Mass:** 19281 **Score:** 50 **Matches:** 1(1) **Sequences:** 1(1)
>tr|A0A481SU01|A0A481SU01_9POTV Polyprotein (Fragment) OS=Zucchini yellow mosaic virus OX=12232 PE=4 SV=1
2::A0A481SU14 **Mass:** 19299 **Score:** 50 **Matches:** 1(1) **Sequences:** 1(1)
>tr|A0A481SU14|A0A481SU14_9POTV Polyprotein (Fragment) OS=Zucchini yellow mosaic virus OX=12232 PE=4 SV=1
2::A0A481SUC5 **Mass:** 19197 **Score:** 50 **Matches:** 1(1) **Sequences:** 1(1)
>tr|A0A481SUC5|A0A481SUC5_9POTV Polyprotein (Fragment) OS=Zucchini yellow mosaic virus OX=12232 PE=4 SV=1
2::A0A481SUD3 **Mass:** 19355 **Score:** 50 **Matches:** 1(1) **Sequences:** 1(1)
>tr|A0A481SUD3|A0A481SUD3_9POTV Polyprotein (Fragment) OS=Zucchini yellow mosaic virus OX=12232 PE=4 SV=1
2::A0A481SUE6 **Mass:** 19242 **Score:** 50 **Matches:** 1(1) **Sequences:** 1(1)
>tr|A0A481SUE6|A0A481SUE6_9POTV Polyprotein (Fragment) OS=Zucchini yellow mosaic virus OX=12232 PE=4 SV=1
2::A0A481SUF5 **Mass:** 19269 **Score:** 50 **Matches:** 1(1) **Sequences:** 1(1)
>tr|A0A481SUF5|A0A481SUF5_9POTV Polyprotein (Fragment) OS=Zucchini yellow mosaic virus OX=12232 PE=4 SV=1
2::A0A481SUG1 **Mass:** 19155 **Score:** 50 **Matches:** 1(1) **Sequences:** 1(1)
>tr|A0A481SUG1|A0A481SUG1_9POTV Polyprotein (Fragment) OS=Zucchini yellow mosaic virus OX=12232 PE=4 SV=1
2::A0A481SUH5 **Mass:** 19191 **Score:** 50 **Matches:** 1(1) **Sequences:** 1(1)
>tr|A0A481SUH5|A0A481SUH5_9POTV Polyprotein (Fragment) OS=Zucchini yellow mosaic virus OX=12232 PE=4 SV=1
2::A0A481SUL8 **Mass:** 19224 **Score:** 50 **Matches:** 1(1) **Sequences:** 1(1)
>tr|A0A481SUL8|A0A481SUL8_9POTV Polyprotein (Fragment) OS=Zucchini yellow mosaic virus OX=12232 PE=4 SV=1
2::A0A481SUM0 **Mass:** 19299 **Score:** 50 **Matches:** 1(1) **Sequences:** 1(1)
>tr|A0A481SUM0|A0A481SUM0_9POTV Polyprotein (Fragment) OS=Zucchini yellow mosaic virus OX=12232 PE=4 SV=1
2::A0A481XTR2 **Mass:** 35322 **Score:** 50 **Matches:** 1(1) **Sequences:** 1(1)
>tr|A0A481XTR2|A0A481XTR2_9POTV Nib (Fragment) OS=Bean common mosaic necrosis virus OX=43240 PE=4 SV=1
2::A0A482AS15 **Mass:** 354022 **Score:** 50 **Matches:** 1(1) **Sequences:** 1(1)
>tr|A0A482AS15|A0A482AS15_9POTV Genome polyprotein OS=Zucchini yellow mosaic virus OX=12232 PE=3 SV=1
2::A0A482LWJ3 **Mass:** 353770 **Score:** 50 **Matches:** 1(1) **Sequences:** 1(1)
>tr|A0A482LWJ3|A0A482LWJ3_9POTV Genome polyprotein OS=Bean common mosaic necrosis virus OX=43240 PE=3 SV=1
2::A0A482LXM3 **Mass:** 353885 **Score:** 50 **Matches:** 1(1) **Sequences:** 1(1)
>tr|A0A482LXM3|A0A482LXM3_9POTV Genome polyprotein OS=Bean common mosaic necrosis virus OX=43240 PE=3 SV=1
2::A0A482M0K1 **Mass:** 353735 **Score:** 50 **Matches:** 1(1) **Sequences:** 1(1)
>tr|A0A482M0K1|A0A482M0K1_9POTV Genome polyprotein OS=Bean common mosaic necrosis virus OX=43240 PE=3 SV=1
2::A0A499QQX9 **Mass:** 353668 **Score:** 50 **Matches:** 1(1) **Sequences:** 1(1)
>tr|A0A499QQX9|A0A499QQX9_9POTV Genome polyprotein OS=Zucchini yellow mosaic virus OX=12232 PE=3 SV=1
2::A0A499QY99 **Mass:** 353668 **Score:** 50 **Matches:** 1(1) **Sequences:** 1(1)
>tr|A0A499QY99|A0A499QY99_9POTV Genome polyprotein OS=Zucchini yellow mosaic virus OX=12232 PE=3 SV=1
2::A0A499R3N8 **Mass:** 353682 **Score:** 50 **Matches:** 1(1) **Sequences:** 1(1)
>tr|A0A499R3N8|A0A499R3N8_9POTV Genome polyprotein OS=Zucchini yellow mosaic virus OX=12232 PE=3 SV=1
2::A0A4V1DVY1 **Mass:** 353362 **Score:** 50 **Matches:** 1(1) **Sequences:** 1(1)
>tr|A0A4V1DVY1|A0A4V1DVY1_9POTV Genome polyprotein OS=Zucchini yellow mosaic virus OX=12232 PE=3 SV=1
2::A0A4Y6GU17 **Mass:** 353417 **Score:** 50 **Matches:** 1(1) **Sequences:** 1(1)
>tr|A0A4Y6GU17|A0A4Y6GU17_9POTV Genome polyprotein OS=Zucchini yellow mosaic virus OX=12232 PE=3 SV=1
2::A0A510E918 **Mass:** 353430 **Score:** 50 **Matches:** 1(1) **Sequences:** 1(1)
>tr|A0A510E918|A0A510E918_9POTV Genome polyprotein OS=Bean common mosaic necrosis virus OX=43240 PE=3 SV=1
2::A0A5J6NPZ3 **Mass:** 269154 **Score:** 50 **Matches:** 1(1) **Sequences:** 1(1)
>tr|A0A5J6NPZ3|A0A5J6NPZ3_9POTV Polyprotein (Fragment) OS=Telosma mosaic virus OX=400394 PE=4 SV=1
2::A0A5J6T5N7 **Mass:** 7964 **Score:** 50 **Matches:** 1(1) **Sequences:** 1(1)
>tr|A0A5J6T5N7|A0A5J6T5N7_9CAUD Uncharacterized protein OS=Bacillus phage 022DV001 OX=2601655 GN=46 PE=4 SV=1
2::A0A5J6T5Z7 **Mass:** 7964 **Score:** 50 **Matches:** 1(1) **Sequences:** 1(1)
>tr|A0A5J6T5Z7|A0A5J6T5Z7_9CAUD Uncharacterized protein OS=Bacillus phage 055SW001 OX=2601662 GN=46 PE=4 SV=1
2::A0A5J6XVX6 **Mass:** 27185 **Score:** 50 **Matches:** 1(1) **Sequences:** 1(1)
>tr|A0A5J6XVX6|A0A5J6XVX6_9POTV Polyprotein (Fragment) OS=Zucchini yellow mosaic virus OX=12232 PE=4 SV=1
2::A0S0E4 **Mass:** 19395 **Score:** 50 **Matches:** 1(1) **Sequences:** 1(1)
>tr|A0S0E4|A0S0E4_9POTV Polyprotein (Fragment) OS=Zucchini yellow mosaic virus OX=12232 PE=3 SV=1
2::A0T1H0 **Mass:** 19856 **Score:** 50 **Matches:** 1(1) **Sequences:** 1(1)
>tr|A0T1H0|A0T1H0_9POTV Polyprotein (Fragment) OS=Zucchini yellow mosaic virus OX=12232 PE=3 SV=1
2::A0T1H1 **Mass:** 19856 **Score:** 50 **Matches:** 1(1) **Sequences:** 1(1)
>tr|A0T1H1|A0T1H1_9POTV Polyprotein (Fragment) OS=Zucchini yellow mosaic virus OX=12232 PE=3 SV=1
2::C0LQA4 **Mass:** 16903 **Score:** 50 **Matches:** 1(1) **Sequences:** 1(1)
>tr|C0LQA4|C0LQA4_9POTV Polyprotein (Fragment) OS=Zucchini yellow mosaic virus OX=12232 PE=4 SV=1
2::C0LQA5 **Mass:** 16876 **Score:** 50 **Matches:** 1(1) **Sequences:** 1(1)
>tr|C0LQA5|C0LQA5_9POTV Polyprotein (Fragment) OS=Zucchini yellow mosaic virus OX=12232 PE=4 SV=1
2::C0LQA6 **Mass:** 16887 **Score:** 50 **Matches:** 1(1) **Sequences:** 1(1)
>tr|C0LQA6|C0LQA6_9POTV Polyprotein (Fragment) OS=Zucchini yellow mosaic virus OX=12232 PE=4 SV=1
2::C0LQA7 **Mass:** 16935 **Score:** 50 **Matches:** 1(1) **Sequences:** 1(1)
>tr|C0LQA7|C0LQA7_9POTV Polyprotein (Fragment) OS=Zucchini yellow mosaic virus OX=12232 PE=4 SV=1
2::C0LQA8 **Mass:** 16874 **Score:** 50 **Matches:** 1(1) **Sequences:** 1(1)

>tr|C0LQA8|C0LQA8_9POTV Polyprotein (Fragment) OS=Zucchini yellow mosaic virus OX=12232 PE=4 SV=1
2::C0LQA9 **Mass:** 16903 **Score:** 50 **Matches:** 1(1) **Sequences:** 1(1)
>tr|C0LQA9|C0LQA9_9POTV Polyprotein (Fragment) OS=Zucchini yellow mosaic virus OX=12232 PE=4 SV=1
2::C0LQB0 **Mass:** 16841 **Score:** 50 **Matches:** 1(1) **Sequences:** 1(1)
>tr|C0LQB0|C0LQB0_9POTV Polyprotein (Fragment) OS=Zucchini yellow mosaic virus OX=12232 PE=4 SV=1
2::C0LQB1 **Mass:** 16903 **Score:** 50 **Matches:** 1(1) **Sequences:** 1(1)
>tr|C0LQB1|C0LQB1_9POTV Polyprotein (Fragment) OS=Zucchini yellow mosaic virus OX=12232 PE=4 SV=1
2::C0LQB2 **Mass:** 16845 **Score:** 50 **Matches:** 1(1) **Sequences:** 1(1)
>tr|C0LQB2|C0LQB2_9POTV Polyprotein (Fragment) OS=Zucchini yellow mosaic virus OX=12232 PE=4 SV=1
2::C0LQB3 **Mass:** 16887 **Score:** 50 **Matches:** 1(1) **Sequences:** 1(1)
>tr|C0LQB3|C0LQB3_9POTV Polyprotein (Fragment) OS=Zucchini yellow mosaic virus OX=12232 PE=4 SV=1
2::C0LQB4 **Mass:** 16875 **Score:** 50 **Matches:** 1(1) **Sequences:** 1(1)
>tr|C0LQB4|C0LQB4_9POTV Polyprotein (Fragment) OS=Zucchini yellow mosaic virus OX=12232 PE=4 SV=1
2::C0LQB5 **Mass:** 16803 **Score:** 50 **Matches:** 1(1) **Sequences:** 1(1)
>tr|C0LQB5|C0LQB5_9POTV Polyprotein (Fragment) OS=Zucchini yellow mosaic virus OX=12232 PE=4 SV=1
2::C0LQB6 **Mass:** 16841 **Score:** 50 **Matches:** 1(1) **Sequences:** 1(1)
>tr|C0LQB6|C0LQB6_9POTV Polyprotein (Fragment) OS=Zucchini yellow mosaic virus OX=12232 PE=4 SV=1
2::C0LQB7 **Mass:** 16903 **Score:** 50 **Matches:** 1(1) **Sequences:** 1(1)
>tr|C0LQB7|C0LQB7_9POTV Polyprotein (Fragment) OS=Zucchini yellow mosaic virus OX=12232 PE=4 SV=1
2::C0LQB8 **Mass:** 16932 **Score:** 50 **Matches:** 1(1) **Sequences:** 1(1)
>tr|C0LQB8|C0LQB8_9POTV Polyprotein (Fragment) OS=Zucchini yellow mosaic virus OX=12232 PE=4 SV=1
2::C0LQB9 **Mass:** 16875 **Score:** 50 **Matches:** 1(1) **Sequences:** 1(1)
>tr|C0LQB9|C0LQB9_9POTV Polyprotein (Fragment) OS=Zucchini yellow mosaic virus OX=12232 PE=4 SV=1
2::C0LQC0 **Mass:** 16889 **Score:** 50 **Matches:** 1(1) **Sequences:** 1(1)
>tr|C0LQC0|C0LQC0_9POTV Polyprotein (Fragment) OS=Zucchini yellow mosaic virus OX=12232 PE=4 SV=1
2::C0LQC1 **Mass:** 16875 **Score:** 50 **Matches:** 1(1) **Sequences:** 1(1)
>tr|C0LQC1|C0LQC1_9POTV Polyprotein (Fragment) OS=Zucchini yellow mosaic virus OX=12232 PE=4 SV=1
2::C0LQC2 **Mass:** 16875 **Score:** 50 **Matches:** 1(1) **Sequences:** 1(1)
>tr|C0LQC2|C0LQC2_9POTV Polyprotein (Fragment) OS=Zucchini yellow mosaic virus OX=12232 PE=4 SV=1
2::C0LQC3 **Mass:** 16903 **Score:** 50 **Matches:** 1(1) **Sequences:** 1(1)
>tr|C0LQC3|C0LQC3_9POTV Polyprotein (Fragment) OS=Zucchini yellow mosaic virus OX=12232 PE=4 SV=1
2::C0LQC4 **Mass:** 16877 **Score:** 50 **Matches:** 1(1) **Sequences:** 1(1)
>tr|C0LQC4|C0LQC4_9POTV Polyprotein (Fragment) OS=Zucchini yellow mosaic virus OX=12232 PE=4 SV=1
2::G0ZFM0 **Mass:** 16297 **Score:** 50 **Matches:** 1(1) **Sequences:** 1(1)
>tr|G0ZFM0|G0ZFM0_9POTV Polyprotein (Fragment) OS=Zucchini yellow mosaic virus OX=12232 PE=4 SV=1
2::Q0R400 **Mass:** 19436 **Score:** 50 **Matches:** 1(1) **Sequences:** 1(1)
>tr|Q0R400|Q0R400_9POTV Polyprotein (Fragment) OS=Zucchini yellow mosaic virus OX=12232 PE=3 SV=1
2::Q0R401 **Mass:** 19785 **Score:** 50 **Matches:** 1(1) **Sequences:** 1(1)
>tr|Q0R401|Q0R401_9POTV Polyprotein (Fragment) OS=Zucchini yellow mosaic virus OX=12232 PE=3 SV=1
2::Q0R402 **Mass:** 19583 **Score:** 50 **Matches:** 1(1) **Sequences:** 1(1)
>tr|Q0R402|Q0R402_9POTV Polyprotein (Fragment) OS=Zucchini yellow mosaic virus OX=12232 PE=3 SV=1
2::Q0R403 **Mass:** 19941 **Score:** 50 **Matches:** 1(1) **Sequences:** 1(1)
>tr|Q0R403|Q0R403_9POTV Polyprotein (Fragment) OS=Zucchini yellow mosaic virus OX=12232 PE=3 SV=1
2::Q0R404 **Mass:** 20236 **Score:** 50 **Matches:** 1(1) **Sequences:** 1(1)
>tr|Q0R404|Q0R404_9POTV Polyprotein (Fragment) OS=Zucchini yellow mosaic virus OX=12232 PE=3 SV=1
2::Q0R405 **Mass:** 19425 **Score:** 50 **Matches:** 1(1) **Sequences:** 1(1)
>tr|Q0R405|Q0R405_9POTV Polyprotein (Fragment) OS=Zucchini yellow mosaic virus OX=12232 PE=3 SV=1
2::Q0R406 **Mass:** 19556 **Score:** 50 **Matches:** 1(1) **Sequences:** 1(1)
>tr|Q0R406|Q0R406_9POTV Polyprotein (Fragment) OS=Zucchini yellow mosaic virus OX=12232 PE=3 SV=1
2::Q0R407 **Mass:** 353517 **Score:** 50 **Matches:** 1(1) **Sequences:** 1(1)
>tr|Q0R407|Q0R407_9POTV Genome polyprotein OS=Zucchini yellow mosaic virus OX=12232 PE=3 SV=1
2::A1DU45 **Mass:** 353658 **Score:** 50 **Matches:** 1(1) **Sequences:** 1(1)
>tr|A1DU45|A1DU45_9POTV Genome polyprotein OS=Zucchini yellow mosaic virus OX=12232 PE=3 SV=1
2::A1DU46 **Mass:** 353615 **Score:** 50 **Matches:** 1(1) **Sequences:** 1(1)
>tr|A1DU46|A1DU46_9POTV Genome polyprotein OS=Zucchini yellow mosaic virus OX=12232 PE=3 SV=1
2::A1KQQ4 **Mass:** 36178 **Score:** 50 **Matches:** 1(1) **Sequences:** 1(1)
>tr|A1KQQ4|A1KQQ4_9POTV Polyprotein (Fragment) OS=Telosma mosaic virus Pangda12 OX=409912 PE=3 SV=1
2::A1KQQ5 **Mass:** 54697 **Score:** 50 **Matches:** 1(1) **Sequences:** 1(1)
>tr|A1KQQ5|A1KQQ5_9POTV Polyprotein (Fragment) OS=Telosma mosaic virus OX=400394 PE=3 SV=2
2::A1XTA3 **Mass:** 352166 **Score:** 50 **Matches:** 1(1) **Sequences:** 1(1)
>tr|A1XTA3|A1XTA3_BTMV Polyprotein OS=Beet mosaic virus OX=114921 PE=3 SV=1
2::E1Y6T4 **Mass:** 56080 **Score:** 50 **Matches:** 1(1) **Sequences:** 1(1)
>tr|E1Y6T4|E1Y6T4_9POTV Polyprotein (Fragment) OS=Passion fruit woodiness virus OX=31732 PE=3 SV=1
2::E1Y6T5 **Mass:** 56080 **Score:** 50 **Matches:** 1(1) **Sequences:** 1(1)
>tr|E1Y6T5|E1Y6T5_9POTV Polyprotein (Fragment) OS=Passion fruit woodiness virus OX=31732 PE=3 SV=1
2::E1Y6T6 **Mass:** 56050 **Score:** 50 **Matches:** 1(1) **Sequences:** 1(1)
>tr|E1Y6T6|E1Y6T6_9POTV Polyprotein (Fragment) OS=Passion fruit woodiness virus OX=31732 PE=3 SV=1
2::E1Y6T7 **Mass:** 55379 **Score:** 50 **Matches:** 1(1) **Sequences:** 1(1)
>tr|E1Y6T7|E1Y6T7_9POTV Polyprotein (Fragment) OS=Passion fruit woodiness virus OX=31732 PE=3 SV=1
2::G1UGS0 **Mass:** 370484 **Score:** 50 **Matches:** 1(1) **Sequences:** 1(1)
>tr|G1UGS0|G1UGS0_9POTV Genome polyprotein OS=East Asian Passiflora virus OX=341167 PE=3 SV=1
2::T1VXY0 **Mass:** 353814 **Score:** 50 **Matches:** 1(1) **Sequences:** 1(1)
>tr|T1VXY0|T1VXY0_9POTV Genome polyprotein OS=Zucchini yellow mosaic virus OX=12232 PE=3 SV=1
2::T1VXY5 **Mass:** 353945 **Score:** 50 **Matches:** 1(1) **Sequences:** 1(1)
>tr|T1VXY5|T1VXY5_9POTV Genome polyprotein OS=Zucchini yellow mosaic virus OX=12232 PE=3 SV=1
2::T1VYN1 **Mass:** 353971 **Score:** 50 **Matches:** 1(1) **Sequences:** 1(1)
>tr|T1VYN1|T1VYN1_9POTV Genome polyprotein OS=Zucchini yellow mosaic virus OX=12232 PE=3 SV=1
2::T1VYN4 **Mass:** 353936 **Score:** 50 **Matches:** 1(1) **Sequences:** 1(1)
>tr|T1VYN4|T1VYN4_9POTV Genome polyprotein OS=Zucchini yellow mosaic virus OX=12232 PE=3 SV=1
2::B2CZL7 **Mass:** 55202 **Score:** 50 **Matches:** 1(1) **Sequences:** 1(1)
>tr|B2CZL7|B2CZL7_9POTV Polyprotein (Fragment) OS=Zucchini yellow mosaic virus OX=12232 PE=3 SV=1
2::B2CZL8 **Mass:** 55493 **Score:** 50 **Matches:** 1(1) **Sequences:** 1(1)
>tr|B2CZL8|B2CZL8_9POTV Polyprotein (Fragment) OS=Zucchini yellow mosaic virus OX=12232 PE=3 SV=1
2::B2CZL9 **Mass:** 55395 **Score:** 50 **Matches:** 1(1) **Sequences:** 1(1)
>tr|B2CZL9|B2CZL9_9POTV Polyprotein (Fragment) OS=Zucchini yellow mosaic virus OX=12232 PE=3 SV=1
2::D2E410 **Mass:** 56080 **Score:** 50 **Matches:** 1(1) **Sequences:** 1(1)
>tr|D2E410|D2E410_9POTV Polyprotein (Fragment) OS=Ugandan Passiflora virus OX=693419 PE=3 SV=1
2::D2E411 **Mass:** 56080 **Score:** 50 **Matches:** 1(1) **Sequences:** 1(1)
>tr|D2E411|D2E411_9POTV Polyprotein (Fragment) OS=Ugandan Passiflora virus OX=693419 PE=3 SV=1
2::D2E412 **Mass:** 56080 **Score:** 50 **Matches:** 1(1) **Sequences:** 1(1)
>tr|D2E412|D2E412_9POTV Polyprotein (Fragment) OS=Ugandan Passiflora virus OX=693419 PE=3 SV=1
2::D2E413 **Mass:** 56178 **Score:** 50 **Matches:** 1(1) **Sequences:** 1(1)
>tr|D2E413|D2E413_9POTV Polyprotein (Fragment) OS=Ugandan Passiflora virus OX=693419 PE=3 SV=1
2::E2ECE4 **Mass:** 37184 **Score:** 50 **Matches:** 1(1) **Sequences:** 1(1)
>tr|E2ECE4|E2ECE4_9POTV Polyprotein (Fragment) OS=Hardenbergia mosaic virus OX=409486 PE=3 SV=1
2::E2IYA5 **Mass:** 19294 **Score:** 50 **Matches:** 1(1) **Sequences:** 1(1)
>tr|E2IYA5|E2IYA5_9POTV Polyprotein (Fragment) OS=Zucchini yellow mosaic virus OX=12232 PE=4 SV=1
2::E2IYA6 **Mass:** 19368 **Score:** 50 **Matches:** 1(1) **Sequences:** 1(1)
>tr|E2IYA6|E2IYA6_9POTV Polyprotein (Fragment) OS=Zucchini yellow mosaic virus OX=12232 PE=4 SV=1
2::Q2L983 **Mass:** 55620 **Score:** 50 **Matches:** 1(1) **Sequences:** 1(1)
>tr|Q2L983|Q2L983_BTMV Polyprotein (Fragment) OS=Beet mosaic virus OX=114921 PE=3 SV=1
2::Q2MHN5 **Mass:** 353591 **Score:** 50 **Matches:** 1(1) **Sequences:** 1(1)
>tr|Q2MHN5|Q2MHN5_9POTV Genome polyprotein OS=Zucchini yellow mosaic virus OX=12232 PE=3 SV=1
2::Q2MHN6 **Mass:** 353512 **Score:** 50 **Matches:** 1(1) **Sequences:** 1(1)
>tr|Q2MHN6|Q2MHN6_9POTV Genome polyprotein OS=Zucchini yellow mosaic virus OX=12232 PE=3 SV=1
2::B3VCB9 **Mass:** 25018 **Score:** 50 **Matches:** 1(1) **Sequences:** 1(1)
>tr|B3VCB9|B3VCB9_9POTV Polyprotein (Fragment) OS=Hardenbergia mosaic virus OX=409486 PE=2 SV=1
2::I3RGT4 **Mass:** 353874 **Score:** 50 **Matches:** 1(1) **Sequences:** 1(1)
>tr|I3RGT4|I3RGT4_9POTV Genome polyprotein OS=Zucchini yellow mosaic virus OX=12232 PE=3 SV=1
2::I3RGT5 **Mass:** 353093 **Score:** 50 **Matches:** 1(1) **Sequences:** 1(1)

>tr|I3RGT5|I3RGT5_9POTV Genome polyprotein OS=Zucchini yellow mosaic virus OX=12232 PE=3 SV=1
2::I3RGT6 **Mass:** 353869 **Score:** 50 **Matches:** 1(1) **Sequences:** 1(1)
>tr|I3RGT6|I3RGT6_9POTV Genome polyprotein OS=Zucchini yellow mosaic virus OX=12232 PE=3 SV=1
2::I3RGT7 **Mass:** 353532 **Score:** 50 **Matches:** 1(1) **Sequences:** 1(1)
>tr|I3RGT7|I3RGT7_9POTV Genome polyprotein OS=Zucchini yellow mosaic virus OX=12232 PE=3 SV=1
2::I3RGT8 **Mass:** 353328 **Score:** 50 **Matches:** 1(1) **Sequences:** 1(1)
>tr|I3RGT8|I3RGT8_9POTV Genome polyprotein OS=Zucchini yellow mosaic virus OX=12232 PE=3 SV=1
2::I3RGT9 **Mass:** 353096 **Score:** 50 **Matches:** 1(1) **Sequences:** 1(1)
>tr|I3RGT9|I3RGT9_9POTV Genome polyprotein OS=Zucchini yellow mosaic virus OX=12232 PE=3 SV=1
2::I3RGU0 **Mass:** 353795 **Score:** 50 **Matches:** 1(1) **Sequences:** 1(1)
>tr|I3RGU0|I3RGU0_9POTV Genome polyprotein OS=Zucchini yellow mosaic virus OX=12232 PE=3 SV=1
2::I3RGU1 **Mass:** 352973 **Score:** 50 **Matches:** 1(1) **Sequences:** 1(1)
>tr|I3RGU1|I3RGU1_9POTV Genome polyprotein OS=Zucchini yellow mosaic virus OX=12232 PE=3 SV=1
2::I3RGU2 **Mass:** 352407 **Score:** 50 **Matches:** 1(1) **Sequences:** 1(1)
>tr|I3RGU2|I3RGU2_9POTV Genome polyprotein OS=Zucchini yellow mosaic virus OX=12232 PE=3 SV=1
2::I3RGU3 **Mass:** 353489 **Score:** 50 **Matches:** 1(1) **Sequences:** 1(1)
>tr|I3RGU3|I3RGU3_9POTV Genome polyprotein OS=Zucchini yellow mosaic virus OX=12232 PE=3 SV=1
2::I3RGU4 **Score:** 50 **Matches:** 1(1) **Sequences:** 1(1)
>tr|I3RGU4|I3RGU4_9POTV Genome polyprotein OS=Zucchini yellow mosaic virus OX=12232 PE=3 SV=1
2::I3RGU5 **Mass:** 353992 **Score:** 50 **Matches:** 1(1) **Sequences:** 1(1)
>tr|I3RGU5|I3RGU5_9POTV Genome polyprotein OS=Zucchini yellow mosaic virus OX=12232 PE=3 SV=1
2::I3RGU6 **Mass:** 352974 **Score:** 50 **Matches:** 1(1) **Sequences:** 1(1)
>tr|I3RGU6|I3RGU6_9POTV Polyprotein OS=Zucchini yellow mosaic virus OX=12232 PE=3 SV=1
2::I3RGU7 **Mass:** 353455 **Score:** 50 **Matches:** 1(1) **Sequences:** 1(1)
>tr|I3RGU7|I3RGU7_9POTV Genome polyprotein OS=Zucchini yellow mosaic virus OX=12232 PE=3 SV=1
2::I3RGU8 **Mass:** 352978 **Score:** 50 **Matches:** 1(1) **Sequences:** 1(1)
>tr|I3RGU8|I3RGU8_9POTV Genome polyprotein OS=Zucchini yellow mosaic virus OX=12232 PE=3 SV=1
2::I3RGU9 **Mass:** 353815 **Score:** 50 **Matches:** 1(1) **Sequences:** 1(1)
>tr|I3RGU9|I3RGU9_9POTV Genome polyprotein OS=Zucchini yellow mosaic virus OX=12232 PE=3 SV=1
2::I3RGV0 **Mass:** 1533525 **Score:** 50 **Matches:** 1(1) **Sequences:** 1(1)
>tr|I3RGV0|I3RGV0_9POTV Genome polyprotein OS=Zucchini yellow mosaic virus OX=12232 PE=3 SV=1
2::I3RGV1 **Mass:** 353975 **Score:** 50 **Matches:** 1(1) **Sequences:** 1(1)
>tr|I3RGV1|I3RGV1_9POTV Genome polyprotein OS=Zucchini yellow mosaic virus OX=12232 PE=3 SV=1
2::I3RGV2 **Mass:** 353806 **Score:** 50 **Matches:** 1(1) **Sequences:** 1(1)
>tr|I3RGV2|I3RGV2_9POTV Genome polyprotein OS=Zucchini yellow mosaic virus OX=12232 PE=3 SV=1
2::I3RGV3 **Mass:** 943689 **Score:** 50 **Matches:** 1(1) **Sequences:** 1(1)
>tr|I3RGV3|I3RGV3_9POTV Genome polyprotein OS=Zucchini yellow mosaic virus OX=12232 PE=3 SV=1
2::I3RGV4 **Mass:** 353833 **Score:** 50 **Matches:** 1(1) **Sequences:** 1(1)
>tr|I3RGV4|I3RGV4_9POTV Genome polyprotein OS=Zucchini yellow mosaic virus OX=12232 PE=3 SV=1
2::I3RGV5 **Mass:** 353302 **Score:** 50 **Matches:** 1(1) **Sequences:** 1(1)
>tr|I3RGV5|I3RGV5_9POTV Genome polyprotein OS=Zucchini yellow mosaic virus OX=12232 PE=3 SV=1
2::I3RGV6 **Mass:** 353743 **Score:** 50 **Matches:** 1(1) **Sequences:** 1(1)
>tr|I3RGV6|I3RGV6_9POTV Genome polyprotein OS=Zucchini yellow mosaic virus OX=12232 PE=3 SV=1
2::I3RGV7 **Mass:** 352847 **Score:** 50 **Matches:** 1(1) **Sequences:** 1(1)
>tr|I3RGV7|I3RGV7_9POTV Polyprotein OS=Zucchini yellow mosaic virus OX=12232 PE=3 SV=1
2::I3RGV8 **Mass:** 353702 **Score:** 50 **Matches:** 1(1) **Sequences:** 1(1)
>tr|I3RGV8|I3RGV8_9POTV Genome polyprotein OS=Zucchini yellow mosaic virus OX=12232 PE=3 SV=1
2::F4ZLQ9 **Mass:** 351876 **Score:** 50 **Matches:** 1(1) **Sequences:** 1(1)
>tr|F4ZLQ9|F4ZLQ9_9POTV Genome polyprotein OS=Hardenbergia mosaic virus OX=409486 PE=3 SV=1
2::F4ZLR0 **Mass:** 353791 **Score:** 50 **Matches:** 1(1) **Sequences:** 1(1)
>tr|F4ZLR0|F4ZLR0_9POTV Genome polyprotein OS=Hardenbergia mosaic virus OX=409486 PE=3 SV=1
2::Q4F982 **Mass:** 55426 **Score:** 50 **Matches:** 1(1) **Sequences:** 1(1)
>tr|Q4F982|Q4F982_9POTY Polyprotein (Fragment) OS=Siratro 2 virus Y OX=336010 PE=4 SV=1
2::D5LH40 **Mass:** 22622 **Score:** 50 **Matches:** 1(1) **Sequences:** 1(1)
>tr|D5LH40|D5LH40_9POTV Polyprotein (Fragment) OS=Zucchini yellow mosaic virus OX=12232 PE=4 SV=1
2::D5LH41 **Mass:** 22554 **Score:** 50 **Matches:** 1(1) **Sequences:** 1(1)
>tr|D5LH41|D5LH41_9POTV Polyprotein (Fragment) OS=Zucchini yellow mosaic virus OX=12232 PE=4 SV=1
2::D5LH42 **Mass:** 22554 **Score:** 50 **Matches:** 1(1) **Sequences:** 1(1)
>tr|D5LH42|D5LH42_9POTV Polyprotein (Fragment) OS=Zucchini yellow mosaic virus OX=12232 PE=4 SV=1
2::D5LH43 **Mass:** 22554 **Score:** 50 **Matches:** 1(1) **Sequences:** 1(1)
>tr|D5LH43|D5LH43_9POTV Polyprotein (Fragment) OS=Zucchini yellow mosaic virus OX=12232 PE=4 SV=1
2::D5LH44 **Mass:** 22554 **Score:** 50 **Matches:** 1(1) **Sequences:** 1(1)
>tr|D5LH44|D5LH44_9POTV Polyprotein (Fragment) OS=Zucchini yellow mosaic virus OX=12232 PE=4 SV=1
2::D5LH45 **Mass:** 22563 **Score:** 50 **Matches:** 1(1) **Sequences:** 1(1)
>tr|D5LH45|D5LH45_9POTV Polyprotein (Fragment) OS=Zucchini yellow mosaic virus OX=12232 PE=4 SV=1
2::D5LH46 **Mass:** 22574 **Score:** 50 **Matches:** 1(1) **Sequences:** 1(1)
>tr|D5LH46|D5LH46_9POTV Polyprotein (Fragment) OS=Zucchini yellow mosaic virus OX=12232 PE=4 SV=1
2::E5KGC6 **Mass:** 354924 **Score:** 50 **Matches:** 1(1) **Sequences:** 1(1)
>tr|E5KGC6|E5KGC6_9POTV Genome polyprotein OS=Passion fruit woodiness virus OX=31732 PE=3 SV=1
2::E5KWJ0 **Mass:** 353393 **Score:** 50 **Matches:** 1(1) **Sequences:** 1(1)
>tr|E5KWJ0|E5KWJ0_9POTV Genome polyprotein OS=Bean common mosaic necrosis virus OX=43240 PE=3 SV=1
2::E5KWJ1 **Mass:** 353830 **Score:** 50 **Matches:** 1(1) **Sequences:** 1(1)
>tr|E5KWJ1|E5KWJ1_9POTV Genome polyprotein OS=Bean common mosaic necrosis virus OX=43240 PE=3 SV=1
2::E5KWJ2 **Mass:** 353478 **Score:** 50 **Matches:** 1(1) **Sequences:** 1(1)
>tr|E5KWJ2|E5KWJ2_9POTV Genome polyprotein OS=Bean common mosaic necrosis virus OX=43240 PE=3 SV=1
2::M5AN52 **Mass:** 354873 **Score:** 50 **Matches:** 1(1) **Sequences:** 1(1)
>tr|M5AN52|M5AN52_9POTV Genome polyprotein OS=Passion fruit woodiness virus OX=31732 PE=3 SV=1
2::Q5I1Y6 **Mass:** 365549 **Score:** 50 **Matches:** 1(1) **Sequences:** 1(1)
>tr|Q5I1Y6|Q5I1Y6_9POTV Genome polyprotein OS=Bean common mosaic necrosis virus OX=43240 PE=3 SV=1
2::Q5R2E9 **Mass:** 44687 **Score:** 50 **Matches:** 1(1) **Sequences:** 1(1)
>tr|Q5R2E9|Q5R2E9_9POTV Polyprotein (Fragment) OS=Zucchini yellow mosaic virus OX=12232 PE=3 SV=1
2::A6PZ08 **Mass:** 353902 **Score:** 50 **Matches:** 1(1) **Sequences:** 1(1)
>tr|A6PZ08|A6PZ08_9POTV Genome polyprotein OS=Zucchini yellow mosaic virus OX=12232 PE=3 SV=1
2::A6XMW3 **Mass:** 353895 **Score:** 50 **Matches:** 1(1) **Sequences:** 1(1)
>tr|A6XMW3|A6XMW3_9POTV Genome polyprotein OS=Telosma mosaic virus OX=400394 PE=3 SV=1
2::A6XP49 **Mass:** 55003 **Score:** 50 **Matches:** 1(1) **Sequences:** 1(1)
>tr|A6XP49|A6XP49_9POTV Polyprotein (Fragment) OS=Zucchini yellow mosaic virus OX=12232 PE=3 SV=1
2::A6XP53 **Mass:** 54856 **Score:** 50 **Matches:** 1(1) **Sequences:** 1(1)
>tr|A6XP53|A6XP53_9POTV Polyprotein (Fragment) OS=Zucchini yellow mosaic virus OX=12232 PE=3 SV=1
2::D6QSX3 **Mass:** 55914 **Score:** 50 **Matches:** 1(1) **Sequences:** 1(1)
>tr|D6QSX3|D6QSX3_9POTV Polyprotein (Fragment) OS=Zucchini yellow mosaic virus OX=12232 PE=3 SV=1
2::D6QSX4 **Mass:** 55858 **Score:** 50 **Matches:** 1(1) **Sequences:** 1(1)
>tr|D6QSX4|D6QSX4_9POTV Polyprotein (Fragment) OS=Zucchini yellow mosaic virus OX=12232 PE=3 SV=1
2::D6QSX5 **Mass:** 55845 **Score:** 50 **Matches:** 1(1) **Sequences:** 1(1)
>tr|D6QSX5|D6QSX5_9POTV Polyprotein (Fragment) OS=Zucchini yellow mosaic virus OX=12232 PE=3 SV=1
2::D6QSX6 **Mass:** 55861 **Score:** 50 **Matches:** 1(1) **Sequences:** 1(1)
>tr|D6QSX6|D6QSX6_9POTV Polyprotein (Fragment) OS=Zucchini yellow mosaic virus OX=12232 PE=3 SV=1
2::D6QSX7 **Mass:** 55914 **Score:** 50 **Matches:** 1(1) **Sequences:** 1(1)
>tr|D6QSX7|D6QSX7_9POTV Polyprotein (Fragment) OS=Zucchini yellow mosaic virus OX=12232 PE=3 SV=1
2::D6QSX8 **Mass:** 55914 **Score:** 50 **Matches:** 1(1) **Sequences:** 1(1)
>tr|D6QSX8|D6QSX8_9POTV Polyprotein (Fragment) OS=Zucchini yellow mosaic virus OX=12232 PE=3 SV=1
2::E6Y2M2 **Mass:** 56284 **Score:** 50 **Matches:** 1(1) **Sequences:** 1(1)
>tr|E6Y2M2|E6Y2M2_BTMV Polyprotein (Fragment) OS=Beet mosaic virus OX=114921 PE=3 SV=1
2::F6MEN7 **Mass:** 36209 **Score:** 50 **Matches:** 1(1) **Sequences:** 1(1)
>tr|F6MEN7|F6MEN7_9POTV Polyprotein (Fragment) OS=Zucchini yellow mosaic virus OX=12232 PE=3 SV=1
2::I6QAJ3 **Mass:** 353534 **Score:** 50 **Matches:** 1(1) **Sequences:** 1(1)
>tr|I6QAJ3|I6QAJ3_9POTV Genome polyprotein OS=Zucchini yellow mosaic virus OX=12232 PE=3 SV=1
2::Q6DRQ4 **Mass:** 9687 **Score:** 50 **Matches:** 1(1) **Sequences:** 1(1)
>tr|Q6DRQ4|Q6DRQ4_9POTV Polyprotein (Fragment) OS=Zucchini yellow mosaic virus OX=12232 PE=4 SV=1
2::Q6DRQ5 **Mass:** 9703 **Score:** 50 **Matches:** 1(1) **Sequences:** 1(1)

>tr|Q6DRQ5|Q6DRQ5_9POTV Polyprotein (Fragment) OS=Zucchini yellow mosaic virus OX=12232 PE=4 SV=1
2::Q6DRQ6 **Mass:** 9745 **Score:** 50 **Matches:** 1(1) **Sequences:** 1(1)
>tr|Q6DRQ6|Q6DRQ6_9POTV Polyprotein (Fragment) OS=Zucchini yellow mosaic virus OX=12232 PE=4 SV=1
2::Q6DRQ7 **Mass:** 9531 **Score:** 50 **Matches:** 1(1) **Sequences:** 1(1)
>tr|Q6DRQ7|Q6DRQ7_9POTV Polyprotein (Fragment) OS=Zucchini yellow mosaic virus OX=12232 PE=4 SV=1
2::Q6DRQ8 **Mass:** 9703 **Score:** 50 **Matches:** 1(1) **Sequences:** 1(1)
>tr|Q6DRQ8|Q6DRQ8_9POTV Polyprotein (Fragment) OS=Zucchini yellow mosaic virus OX=12232 PE=4 SV=1
2::Q6DRQ9 **Mass:** 9703 **Score:** 50 **Matches:** 1(1) **Sequences:** 1(1)
>tr|Q6DRQ9|Q6DRQ9_9POTV Polyprotein (Fragment) OS=Zucchini yellow mosaic virus OX=12232 PE=4 SV=1
2::Q6DRR1 **Mass:** 9703 **Score:** 50 **Matches:** 1(1) **Sequences:** 1(1)
>tr|Q6DRR1|Q6DRR1_9POTV Polyprotein (Fragment) OS=Zucchini yellow mosaic virus OX=12232 PE=4 SV=1
2::Q6DRR2 **Mass:** 9703 **Score:** 50 **Matches:** 1(1) **Sequences:** 1(1)
>tr|Q6DRR2|Q6DRR2_9POTV Polyprotein (Fragment) OS=Zucchini yellow mosaic virus OX=12232 PE=4 SV=1
2::Q6DRR4 **Mass:** 9745 **Score:** 50 **Matches:** 1(1) **Sequences:** 1(1)
>tr|Q6DRR4|Q6DRR4_9POTV Polyprotein (Fragment) OS=Zucchini yellow mosaic virus OX=12232 PE=4 SV=1
2::Q6GYE7 **Mass:** 36412 **Score:** 50 **Matches:** 1(1) **Sequences:** 1(1)
>tr|Q6GYE7|Q6GYE7_9POTV Polyprotein (Fragment) OS=Zucchini yellow mosaic virus OX=12232 PE=3 SV=1
2::Q6GYE8 **Mass:** 36485 **Score:** 50 **Matches:** 1(1) **Sequences:** 1(1)
>tr|Q6GYE8|Q6GYE8_9POTV Polyprotein (Fragment) OS=Zucchini yellow mosaic virus OX=12232 PE=3 SV=1
2::Q6GYE9 **Mass:** 36283 **Score:** 50 **Matches:** 1(1) **Sequences:** 1(1)
>tr|Q6GYE9|Q6GYE9_9POTV Polyprotein (Fragment) OS=Zucchini yellow mosaic virus OX=12232 PE=3 SV=1
2::Q6GYF1 **Mass:** 36460 **Score:** 50 **Matches:** 1(1) **Sequences:** 1(1)
>tr|Q6GYF1|Q6GYF1_9POTV Polyprotein (Fragment) OS=Zucchini yellow mosaic virus OX=12232 PE=3 SV=1
2::Q6GYF2 **Mass:** 37033 **Score:** 50 **Matches:** 1(1) **Sequences:** 1(1)
>tr|Q6GYF2|Q6GYF2_9POTV Polyprotein (Fragment) OS=Zucchini yellow mosaic virus OX=12232 PE=3 SV=1
2::Q6WN47 **Mass:** 353477 **Score:** 50 **Matches:** 1(1) **Sequences:** 1(1)
>tr|Q6WN47|Q6WN47_9POTV Genome polyprotein OS=Zucchini yellow mosaic virus OX=12232 PE=3 SV=1
2::Q6WN48 **Mass:** 353437 **Score:** 50 **Matches:** 1(1) **Sequences:** 1(1)
>tr|Q6WN48|Q6WN48_9POTV Genome polyprotein OS=Zucchini yellow mosaic virus OX=12232 PE=3 SV=1
1::Q6XW15 **Mass:** 352131 **Score:** 50 **Matches:** 1(1) **Sequences:** 1(1)
Genome polyprotein OS=Beet mosaic virus OX=114921 PE=3 SV=1
2::Q6Y2U7 **Mass:** 353588 **Score:** 50 **Matches:** 1(1) **Sequences:** 1(1)
>tr|Q6Y2U7|Q6Y2U7_9POTV Genome polyprotein OS=Zucchini yellow mosaic virus OX=12232 PE=3 SV=1
2::V6CKQ5 **Mass:** 353496 **Score:** 50 **Matches:** 1(1) **Sequences:** 1(1)
>tr|V6CKQ5|V6CKQ5_9POTV Genome polyprotein OS=Bean common mosaic necrosis virus OX=43240 PE=3 SV=1
2::W6A4S5 **Mass:** 36153 **Score:** 50 **Matches:** 1(1) **Sequences:** 1(1)
>tr|W6A4S5|W6A4S5_BTMV Polyprotein (Fragment) OS=Beet mosaic virus OX=114921 PE=3 SV=1
2::W6A536 **Mass:** 36139 **Score:** 50 **Matches:** 1(1) **Sequences:** 1(1)
>tr|W6A536|W6A536_BTMV Polyprotein (Fragment) OS=Beet mosaic virus OX=114921 PE=3 SV=1
2::W6A584 **Mass:** 36167 **Score:** 50 **Matches:** 1(1) **Sequences:** 1(1)
>tr|W6A584|W6A584_BTMV Polyprotein (Fragment) OS=Beet mosaic virus OX=114921 PE=3 SV=1
2::W6A5F7 **Mass:** 36175 **Score:** 50 **Matches:** 1(1) **Sequences:** 1(1)
>tr|W6A5F7|W6A5F7_BTMV Polyprotein (Fragment) OS=Beet mosaic virus OX=114921 PE=3 SV=1
2::W6AEK2 **Mass:** 36153 **Score:** 50 **Matches:** 1(1) **Sequences:** 1(1)
>tr|W6AEK2|W6AEK2_BTMV Polyprotein (Fragment) OS=Beet mosaic virus OX=114921 PE=3 SV=1
2::W6AEK8 **Mass:** 36185 **Score:** 50 **Matches:** 1(1) **Sequences:** 1(1)
>tr|W6AEK8|W6AEK8_BTMV Polyprotein (Fragment) OS=Beet mosaic virus OX=114921 PE=3 SV=1
2::A7K7E1 **Mass:** 18212 **Score:** 50 **Matches:** 1(1) **Sequences:** 1(1)
>tr|A7K7E1|A7K7E1_9POTV Polyprotein (Fragment) OS=Zucchini yellow mosaic virus OX=12232 PE=4 SV=1
2::A7K7E2 **Mass:** 18184 **Score:** 50 **Matches:** 1(1) **Sequences:** 1(1)
>tr|A7K7E2|A7K7E2_9POTV Polyprotein (Fragment) OS=Zucchini yellow mosaic virus OX=12232 PE=4 SV=1
2::A7K7E3 **Mass:** 18212 **Score:** 50 **Matches:** 1(1) **Sequences:** 1(1)
>tr|A7K7E3|A7K7E3_9POTV Polyprotein (Fragment) OS=Zucchini yellow mosaic virus OX=12232 PE=4 SV=1
2::A7K7E4 **Mass:** 18212 **Score:** 50 **Matches:** 1(1) **Sequences:** 1(1)
>tr|A7K7E4|A7K7E4_9POTV Polyprotein (Fragment) OS=Zucchini yellow mosaic virus OX=12232 PE=4 SV=1
2::A7K7E5 **Mass:** 18184 **Score:** 50 **Matches:** 1(1) **Sequences:** 1(1)
>tr|A7K7E5|A7K7E5_9POTV Polyprotein (Fragment) OS=Zucchini yellow mosaic virus OX=12232 PE=4 SV=1
2::A7K7E6 **Mass:** 18158 **Score:** 50 **Matches:** 1(1) **Sequences:** 1(1)
>tr|A7K7E6|A7K7E6_9POTV Polyprotein (Fragment) OS=Zucchini yellow mosaic virus OX=12232 PE=4 SV=1
2::A7K7E7 **Mass:** 18158 **Score:** 50 **Matches:** 1(1) **Sequences:** 1(1)
>tr|A7K7E7|A7K7E7_9POTV Polyprotein (Fragment) OS=Zucchini yellow mosaic virus OX=12232 PE=4 SV=1
2::A7K7E8 **Mass:** 18193 **Score:** 50 **Matches:** 1(1) **Sequences:** 1(1)
>tr|A7K7E8|A7K7E8_9POTV Polyprotein (Fragment) OS=Zucchini yellow mosaic virus OX=12232 PE=4 SV=1
2::A7K7E9 **Mass:** 18253 **Score:** 50 **Matches:** 1(1) **Sequences:** 1(1)
>tr|A7K7E9|A7K7E9_9POTV Polyprotein (Fragment) OS=Zucchini yellow mosaic virus OX=12232 PE=4 SV=1
2::A7K7F0 **Mass:** 18212 **Score:** 50 **Matches:** 1(1) **Sequences:** 1(1)
>tr|A7K7F0|A7K7F0_9POTV Polyprotein (Fragment) OS=Zucchini yellow mosaic virus OX=12232 PE=4 SV=1
2::A7K7F1 **Mass:** 18252 **Score:** 50 **Matches:** 1(1) **Sequences:** 1(1)
>tr|A7K7F1|A7K7F1_9POTV Polyprotein (Fragment) OS=Zucchini yellow mosaic virus OX=12232 PE=4 SV=1
2::A7K7F2 **Mass:** 18158 **Score:** 50 **Matches:** 1(1) **Sequences:** 1(1)
>tr|A7K7F2|A7K7F2_9POTV Polyprotein (Fragment) OS=Zucchini yellow mosaic virus OX=12232 PE=4 SV=1
2::A7UAK9 **Mass:** 47177 **Score:** 50 **Matches:** 1(1) **Sequences:** 1(1)
>tr|A7UAK9|A7UAK9_9POTV Polyprotein (Fragment) OS=Malaysian Passiflora virus OX=457561 PE=2 SV=1
2::B7S4W8 **Mass:** 55835 **Score:** 50 **Matches:** 1(1) **Sequences:** 1(1)
>tr|B7S4W8|B7S4W8_9POTV Polyprotein (Fragment) OS=Zucchini yellow mosaic virus OX=12232 PE=3 SV=1
2::B7X6I1 **Mass:** 95700 **Score:** 50 **Matches:** 1(1) **Sequences:** 1(1)
>tr|B7X6I1|B7X6I1_9POTV Polyprotein (Fragment) OS=Yambean mosaic virus OX=418827 GN=NIa-NIb-CP PE=3 SV=1
2::C7G101 **Mass:** 10002 **Score:** 50 **Matches:** 1(1) **Sequences:** 1(1)
>tr|C7G101|C7G101_9POTV NIb replicase (Fragment) OS=Zucchini yellow mosaic virus OX=12232 GN=NIb PE=4 SV=1
2::C7G103 **Mass:** 10002 **Score:** 50 **Matches:** 1(1) **Sequences:** 1(1)
>tr|C7G103|C7G103_9POTV NIb replicase (Fragment) OS=Zucchini yellow mosaic virus OX=12232 GN=NIb PE=4 SV=1
2::Q7T908 **Mass:** 353603 **Score:** 50 **Matches:** 1(1) **Sequences:** 1(1)
>tr|Q7T908|Q7T908_9POTV Genome polyprotein OS=Zucchini yellow mosaic virus OX=12232 PE=2 SV=1
2::Q7T910 **Mass:** 53737 **Score:** 50 **Matches:** 1(1) **Sequences:** 1(1)
>tr|Q7T910|Q7T910_9POTV Polyprotein (Fragment) OS=Zucchini yellow mosaic virus OX=12232 PE=2 SV=1
2::Q7T911 **Mass:** 55974 **Score:** 50 **Matches:** 1(1) **Sequences:** 1(1)
>tr|Q7T911|Q7T911_9POTV Polyprotein (Fragment) OS=Zucchini yellow mosaic virus OX=12232 PE=2 SV=1
2::Q7T912 **Mass:** 55998 **Score:** 50 **Matches:** 1(1) **Sequences:** 1(1)
>tr|Q7T912|Q7T912_9POTV Polyprotein (Fragment) OS=Zucchini yellow mosaic virus OX=12232 PE=2 SV=1
2::Q7T914 **Mass:** 353157 **Score:** 50 **Matches:** 1(1) **Sequences:** 1(1)
>tr|Q7T914|Q7T914_9POTV Genome polyprotein OS=Zucchini yellow mosaic virus OX=12232 PE=2 SV=1
2::Q7T915 **Mass:** 353564 **Score:** 50 **Matches:** 1(1) **Sequences:** 1(1)
>tr|Q7T915|Q7T915_9POTV Genome polyprotein OS=Zucchini yellow mosaic virus OX=12232 PE=2 SV=1
2::Q7T916 **Mass:** 55962 **Score:** 50 **Matches:** 1(1) **Sequences:** 1(1)
>tr|Q7T916|Q7T916_9POTV Polyprotein (Fragment) OS=Zucchini yellow mosaic virus OX=12232 PE=2 SV=1
2::Q7T917 **Mass:** 353336 **Score:** 50 **Matches:** 1(1) **Sequences:** 1(1)
>tr|Q7T917|Q7T917_9POTV Genome polyprotein OS=Zucchini yellow mosaic virus OX=12232 PE=2 SV=1
2::A8E0J4 **Mass:** 20478 **Score:** 50 **Matches:** 1(1) **Sequences:** 1(1)
>tr|A8E0J4|A8E0J4_9POTV Polyprotein (Fragment) OS=Bean common mosaic necrosis virus OX=43240 PE=3 SV=1
2::B8YJI9 **Mass:** 46711 **Score:** 50 **Matches:** 1(1) **Sequences:** 1(1)
>tr|B8YJI9|B8YJI9_9POTV Polyprotein (Fragment) OS=Begonia flower breaking virus OX=588752 PE=3 SV=1
2::Q8BA03 **Mass:** 353741 **Score:** 50 **Matches:** 1(1) **Sequences:** 1(1)
>tr|Q8BA03|Q8BA03_9POTV Genome polyprotein OS=Bean common mosaic necrosis virus OX=43240 PE=3 SV=1
2::Q8QKU4 **Mass:** 218697 **Score:** 50 **Matches:** 1(1) **Sequences:** 1(1)
>tr|Q8QKU4|Q8QKU4_9POTV Genome polyprotein (Fragment) OS=Zucchini yellow mosaic virus OX=12232 PE=3 SV=1
2::A9EEY5 **Mass:** 353486 **Score:** 50 **Matches:** 1(1) **Sequences:** 1(1)
>tr|A9EEY5|A9EEY5_9POTV Genome polyprotein OS=Zucchini yellow mosaic virus OX=12232 PE=3 SV=1
2::C9EBA0 **Mass:** 36080 **Score:** 50 **Matches:** 1(1) **Sequences:** 1(1)
>tr|C9EBA0|C9EBA0_9POTV Polyprotein (Fragment) OS=Zucchini yellow mosaic virus OX=12232 PE=3 SV=1
2::D9MNL7 **Mass:** 19308 **Score:** 50 **Matches:** 1(1) **Sequences:** 1(1)

>tr|D9MNL7|D9MNL7_9POTV Polyprotein (Fragment) OS=Zucchini yellow mosaic virus OX=12232 PE=4 SV=1
2::G9F9P9 **Mass:** 352831 **Score:** 50 **Matches:** 1(1) **Sequences:** 1(1)
>tr|G9F9P9|G9F9P9_9POTV Polyprotein (Fragment) OS=Blue squill virus A OX=1072517 PE=3 SV=1
2::G9HZB8 **Mass:** 289284 **Score:** 50 **Matches:** 1(1) **Sequences:** 1(1)
>tr|G9HZB8|G9HZB8_9POTV Genome polyprotein (Fragment) OS=Blue squill virus A OX=1072517 PE=3 SV=1
2::J9XMR3 **Mass:** 43244 **Score:** 50 **Matches:** 1(1) **Sequences:** 1(1)
>tr|J9XMR3|J9XMR3_9POTV Polyprotein (Fragment) OS=Zucchini yellow mosaic virus OX=12232 PE=3 SV=1
2::J9XMR7 **Mass:** 43304 **Score:** 50 **Matches:** 1(1) **Sequences:** 1(1)
>tr|J9XMR7|J9XMR7_9POTV Polyprotein (Fragment) OS=Zucchini yellow mosaic virus OX=12232 PE=3 SV=1
2::J9XMS2 **Mass:** 43263 **Score:** 50 **Matches:** 1(1) **Sequences:** 1(1)
>tr|J9XMS2|J9XMS2_9POTV Polyprotein (Fragment) OS=Zucchini yellow mosaic virus OX=12232 PE=3 SV=1
2::J9XMS5 **Mass:** 43308 **Score:** 50 **Matches:** 1(1) **Sequences:** 1(1)
>tr|J9XMS5|J9XMS5_9POTV Polyprotein (Fragment) OS=Zucchini yellow mosaic virus OX=12232 PE=3 SV=1
2::J9XMS9 **Mass:** 19574 **Score:** 50 **Matches:** 1(1) **Sequences:** 1(1)
>tr|J9XMS9|J9XMS9_9POTV Polyprotein (Fragment) OS=Zucchini yellow mosaic virus OX=12232 PE=4 SV=1
2::J9XMT4 **Mass:** 19625 **Score:** 50 **Matches:** 1(1) **Sequences:** 1(1)
>tr|J9XMT4|J9XMT4_9POTV Polyprotein (Fragment) OS=Zucchini yellow mosaic virus OX=12232 PE=4 SV=1
2::J9XMT9 **Mass:** 19624 **Score:** 50 **Matches:** 1(1) **Sequences:** 1(1)
>tr|J9XMT9|J9XMT9_9POTV Polyprotein (Fragment) OS=Zucchini yellow mosaic virus OX=12232 PE=4 SV=1
2::J9XMU3 **Mass:** 19669 **Score:** 50 **Matches:** 1(1) **Sequences:** 1(1)
>tr|J9XMU3|J9XMU3_9POTV Polyprotein (Fragment) OS=Zucchini yellow mosaic virus OX=12232 PE=4 SV=1
2::J9XMU8 **Mass:** 19574 **Score:** 50 **Matches:** 1(1) **Sequences:** 1(1)
>tr|J9XMU8|J9XMU8_9POTV Polyprotein (Fragment) OS=Zucchini yellow mosaic virus OX=12232 PE=4 SV=1
2::J9XMV2 **Mass:** 43308 **Score:** 50 **Matches:** 1(1) **Sequences:** 1(1)
>tr|J9XMV2|J9XMV2_9POTV Polyprotein (Fragment) OS=Zucchini yellow mosaic virus OX=12232 PE=3 SV=1
2::J9XMV3 **Mass:** 19651 **Score:** 50 **Matches:** 1(1) **Sequences:** 1(1)
>tr|J9XMV3|J9XMV3_9POTV Polyprotein (Fragment) OS=Zucchini yellow mosaic virus OX=12232 PE=4 SV=1
2::J9XMV5 **Mass:** 43262 **Score:** 50 **Matches:** 1(1) **Sequences:** 1(1)
>tr|J9XMV5|J9XMV5_9POTV Polyprotein (Fragment) OS=Zucchini yellow mosaic virus OX=12232 PE=3 SV=1
2::J9XMV8 **Mass:** 19609 **Score:** 50 **Matches:** 1(1) **Sequences:** 1(1)
>tr|J9XMV8|J9XMV8_9POTV Polyprotein (Fragment) OS=Zucchini yellow mosaic virus OX=12232 PE=4 SV=1
2::J9XMW0 **Mass:** 43273 **Score:** 50 **Matches:** 1(1) **Sequences:** 1(1)
>tr|J9XMW0|J9XMW0_9POTV Polyprotein (Fragment) OS=Zucchini yellow mosaic virus OX=12232 PE=3 SV=1
2::J9XMW2 **Mass:** 19653 **Score:** 50 **Matches:** 1(1) **Sequences:** 1(1)
>tr|J9XMW2|J9XMW2_9POTV Polyprotein (Fragment) OS=Zucchini yellow mosaic virus OX=12232 PE=4 SV=1
2::J9XMW5 **Mass:** 19669 **Score:** 50 **Matches:** 1(1) **Sequences:** 1(1)
>tr|J9XMW5|J9XMW5_9POTV Polyprotein (Fragment) OS=Zucchini yellow mosaic virus OX=12232 PE=4 SV=1
2::J9XMW7 **Mass:** 19639 **Score:** 50 **Matches:** 1(1) **Sequences:** 1(1)
>tr|J9XMW7|J9XMW7_9POTV Polyprotein (Fragment) OS=Zucchini yellow mosaic virus OX=12232 PE=4 SV=1
2::J9XMX0 **Mass:** 19638 **Score:** 50 **Matches:** 1(1) **Sequences:** 1(1)
>tr|J9XMX0|J9XMX0_9POTV Polyprotein (Fragment) OS=Zucchini yellow mosaic virus OX=12232 PE=4 SV=1
2::J9XMX2 **Mass:** 19669 **Score:** 50 **Matches:** 1(1) **Sequences:** 1(1)
>tr|J9XMX2|J9XMX2_9POTV Polyprotein (Fragment) OS=Zucchini yellow mosaic virus OX=12232 PE=4 SV=1
2::J9XMX3 **Mass:** 19723 **Score:** 50 **Matches:** 1(1) **Sequences:** 1(1)
>tr|J9XMX3|J9XMX3_9POTV Polyprotein (Fragment) OS=Zucchini yellow mosaic virus OX=12232 PE=4 SV=1
2::J9XMX6 **Mass:** 19639 **Score:** 50 **Matches:** 1(1) **Sequences:** 1(1)
>tr|J9XMX6|J9XMX6_9POTV Polyprotein (Fragment) OS=Zucchini yellow mosaic virus OX=12232 PE=4 SV=1
2::J9XMX7 **Mass:** 19573 **Score:** 50 **Matches:** 1(1) **Sequences:** 1(1)
>tr|J9XMX7|J9XMX7_9POTV Polyprotein (Fragment) OS=Zucchini yellow mosaic virus OX=12232 PE=4 SV=1
2::J9XMY0 **Mass:** 19709 **Score:** 50 **Matches:** 1(1) **Sequences:** 1(1)
>tr|J9XMY0|J9XMY0_9POTV Polyprotein (Fragment) OS=Zucchini yellow mosaic virus OX=12232 PE=4 SV=1
2::J9XMY1 **Mass:** 19639 **Score:** 50 **Matches:** 1(1) **Sequences:** 1(1)
>tr|J9XMY1|J9XMY1_9POTV Polyprotein (Fragment) OS=Zucchini yellow mosaic virus OX=12232 PE=4 SV=1
2::J9XMY2 **Mass:** 19574 **Score:** 50 **Matches:** 1(1) **Sequences:** 1(1)
>tr|J9XMY2|J9XMY2_9POTV Polyprotein (Fragment) OS=Zucchini yellow mosaic virus OX=12232 PE=4 SV=1
2::J9XMY5 **Mass:** 19639 **Score:** 50 **Matches:** 1(1) **Sequences:** 1(1)
>tr|J9XMY5|J9XMY5_9POTV Polyprotein (Fragment) OS=Zucchini yellow mosaic virus OX=12232 PE=4 SV=1
2::J9XMY6 **Mass:** 19651 **Score:** 50 **Matches:** 1(1) **Sequences:** 1(1)
>tr|J9XMY6|J9XMY6_9POTV Polyprotein (Fragment) OS=Zucchini yellow mosaic virus OX=12232 PE=4 SV=1
2::J9XMZ1 **Mass:** 19609 **Score:** 50 **Matches:** 1(1) **Sequences:** 1(1)
>tr|J9XMZ1|J9XMZ1_9POTV Polyprotein (Fragment) OS=Zucchini yellow mosaic virus OX=12232 PE=4 SV=1
2::J9XMZ4 **Mass:** 19653 **Score:** 50 **Matches:** 1(1) **Sequences:** 1(1)
>tr|J9XMZ4|J9XMZ4_9POTV Polyprotein (Fragment) OS=Zucchini yellow mosaic virus OX=12232 PE=4 SV=1
2::J9XMZ8 **Mass:** 19639 **Score:** 50 **Matches:** 1(1) **Sequences:** 1(1)
>tr|J9XMZ8|J9XMZ8_9POTV Polyprotein (Fragment) OS=Zucchini yellow mosaic virus OX=12232 PE=4 SV=1
2::J9XN02 **Mass:** 19653 **Score:** 50 **Matches:** 1(1) **Sequences:** 1(1)
>tr|J9XN02|J9XN02_9POTV Polyprotein (Fragment) OS=Zucchini yellow mosaic virus OX=12232 PE=4 SV=1
2::J9XN06 **Mass:** 19584 **Score:** 50 **Matches:** 1(1) **Sequences:** 1(1)
>tr|J9XN06|J9XN06_9POTV Polyprotein (Fragment) OS=Zucchini yellow mosaic virus OX=12232 PE=4 SV=1
2::J9XN10 **Mass:** 19573 **Score:** 50 **Matches:** 1(1) **Sequences:** 1(1)
>tr|J9XN10|J9XN10_9POTV Polyprotein (Fragment) OS=Zucchini yellow mosaic virus OX=12232 PE=4 SV=1
2::J9XN15 **Mass:** 19639 **Score:** 50 **Matches:** 1(1) **Sequences:** 1(1)
>tr|J9XN15|J9XN15_9POTV Polyprotein (Fragment) OS=Zucchini yellow mosaic virus OX=12232 PE=4 SV=1
2::J9XN20 **Mass:** 19639 **Score:** 50 **Matches:** 1(1) **Sequences:** 1(1)
>tr|J9XN20|J9XN20_9POTV Polyprotein (Fragment) OS=Zucchini yellow mosaic virus OX=12232 PE=4 SV=1
2::J9XPZ3 **Mass:** 43220 **Score:** 50 **Matches:** 1(1) **Sequences:** 1(1)
>tr|J9XPZ3|J9XPZ3_9POTV Polyprotein (Fragment) OS=Zucchini yellow mosaic virus OX=12232 PE=3 SV=1
2::J9XPZ8 **Mass:** 43322 **Score:** 50 **Matches:** 1(1) **Sequences:** 1(1)
>tr|J9XPZ8|J9XPZ8_9POTV Polyprotein (Fragment) OS=Zucchini yellow mosaic virus OX=12232 PE=3 SV=1
2::J9XQ03 **Mass:** 43300 **Score:** 50 **Matches:** 1(1) **Sequences:** 1(1)
>tr|J9XQ03|J9XQ03_9POTV Polyprotein (Fragment) OS=Zucchini yellow mosaic virus OX=12232 PE=3 SV=1
2::J9XQ08 **Mass:** 19698 **Score:** 50 **Matches:** 1(1) **Sequences:** 1(1)
>tr|J9XQ08|J9XQ08_9POTV Polyprotein (Fragment) OS=Zucchini yellow mosaic virus OX=12232 PE=4 SV=1
2::J9XQ10 **Mass:** 19669 **Score:** 50 **Matches:** 1(1) **Sequences:** 1(1)
>tr|J9XQ10|J9XQ10_9POTV Polyprotein (Fragment) OS=Zucchini yellow mosaic virus OX=12232 PE=4 SV=1
2::J9XQ15 **Mass:** 19728 **Score:** 50 **Matches:** 1(1) **Sequences:** 1(1)
>tr|J9XQ15|J9XQ15_9POTV Polyprotein (Fragment) OS=Zucchini yellow mosaic virus OX=12232 PE=4 SV=1
2::J9XQ20 **Mass:** 19541 **Score:** 50 **Matches:** 1(1) **Sequences:** 1(1)
>tr|J9XQ20|J9XQ20_9POTV Polyprotein (Fragment) OS=Zucchini yellow mosaic virus OX=12232 PE=4 SV=1
2::J9XQ23 **Mass:** 19670 **Score:** 50 **Matches:** 1(1) **Sequences:** 1(1)
>tr|J9XQ23|J9XQ23_9POTV Polyprotein (Fragment) OS=Zucchini yellow mosaic virus OX=12232 PE=4 SV=1
2::J9XQ26 **Mass:** 19651 **Score:** 50 **Matches:** 1(1) **Sequences:** 1(1)
>tr|J9XQ26|J9XQ26_9POTV Polyprotein (Fragment) OS=Zucchini yellow mosaic virus OX=12232 PE=4 SV=1
2::J9XQ31 **Mass:** 19638 **Score:** 50 **Matches:** 1(1) **Sequences:** 1(1)
>tr|J9XQ31|J9XQ31_9POTV Polyprotein (Fragment) OS=Zucchini yellow mosaic virus OX=12232 PE=4 SV=1
2::J9XQ36 **Mass:** 19639 **Score:** 50 **Matches:** 1(1) **Sequences:** 1(1)
>tr|J9XQ36|J9XQ36_9POTV Polyprotein (Fragment) OS=Zucchini yellow mosaic virus OX=12232 PE=4 SV=1
2::J9XQ38 **Mass:** 19639 **Score:** 50 **Matches:** 1(1) **Sequences:** 1(1)
>tr|J9XQ38|J9XQ38_9POTV Polyprotein (Fragment) OS=Zucchini yellow mosaic virus OX=12232 PE=4 SV=1
2::J9XQ42 **Mass:** 19638 **Score:** 50 **Matches:** 1(1) **Sequences:** 1(1)
>tr|J9XQ42|J9XQ42_9POTV Polyprotein (Fragment) OS=Zucchini yellow mosaic virus OX=12232 PE=4 SV=1
2::J9XQ46 **Mass:** 19638 **Score:** 50 **Matches:** 1(1) **Sequences:** 1(1)
>tr|J9XQ46|J9XQ46_9POTV Polyprotein (Fragment) OS=Zucchini yellow mosaic virus OX=12232 PE=4 SV=1
2::J9XQ49 **Mass:** 19584 **Score:** 50 **Matches:** 1(1) **Sequences:** 1(1)
>tr|J9XQ49|J9XQ49_9POTV Polyprotein (Fragment) OS=Zucchini yellow mosaic virus OX=12232 PE=4 SV=1
2::J9XQ54 **Mass:** 19639 **Score:** 50 **Matches:** 1(1) **Sequences:** 1(1)
>tr|J9XQ54|J9XQ54_9POTV Polyprotein (Fragment) OS=Zucchini yellow mosaic virus OX=12232 PE=4 SV=1
2::J9XQ58 **Mass:** 19638 **Score:** 50 **Matches:** 1(1) **Sequences:** 1(1)
>tr|J9XQ58|J9XQ58_9POTV Polyprotein (Fragment) OS=Zucchini yellow mosaic virus OX=12232 PE=4 SV=1
2::J9XRU8 **Mass:** 43198 **Score:** 50 **Matches:** 1(1) **Sequences:** 1(1)

>tr|J9XRU8|J9XRU8_9POTV Polyprotein (Fragment) OS=Zucchini yellow mosaic virus OX=12232 PE=3 SV=1
2::J9XRV1 **Mass:** 43256 **Score:** 50 **Matches:** 1(1) **Sequences:** 1(1)
>tr|J9XRV1|J9XRV1_9POTV Polyprotein (Fragment) OS=Zucchini yellow mosaic virus OX=12232 PE=3 SV=1
2::J9XRV4 **Mass:** 43355 **Score:** 50 **Matches:** 1(1) **Sequences:** 1(1)
>tr|J9XRV4|J9XRV4_9POTV Polyprotein (Fragment) OS=Zucchini yellow mosaic virus OX=12232 PE=3 SV=1
2::J9XRV7 **Mass:** 19613 **Score:** 50 **Matches:** 1(1) **Sequences:** 1(1)
>tr|J9XRV7|J9XRV7_9POTV Polyprotein (Fragment) OS=Zucchini yellow mosaic virus OX=12232 PE=4 SV=1
2::J9XRW0 **Mass:** 19574 **Score:** 50 **Matches:** 1(1) **Sequences:** 1(1)
>tr|J9XRW0|J9XRW0_9POTV Polyprotein (Fragment) OS=Zucchini yellow mosaic virus OX=12232 PE=4 SV=1
2::J9XRW4 **Mass:** 19655 **Score:** 50 **Matches:** 1(1) **Sequences:** 1(1)
>tr|J9XRW4|J9XRW4_9POTV Polyprotein (Fragment) OS=Zucchini yellow mosaic virus OX=12232 PE=4 SV=1
2::J9XRW7 **Mass:** 19532 **Score:** 50 **Matches:** 1(1) **Sequences:** 1(1)
>tr|J9XRW7|J9XRW7_9POTV Polyprotein (Fragment) OS=Zucchini yellow mosaic virus OX=12232 PE=4 SV=1
2::J9XRX0 **Mass:** 19709 **Score:** 50 **Matches:** 1(1) **Sequences:** 1(1)
>tr|J9XRX0|J9XRX0_9POTV Polyprotein (Fragment) OS=Zucchini yellow mosaic virus OX=12232 PE=4 SV=1
2::J9XRX4 **Mass:** 19669 **Score:** 50 **Matches:** 1(1) **Sequences:** 1(1)
>tr|J9XRX4|J9XRX4_9POTV Polyprotein (Fragment) OS=Zucchini yellow mosaic virus OX=12232 PE=4 SV=1
2::J9XRX7 **Mass:** 19651 **Score:** 50 **Matches:** 1(1) **Sequences:** 1(1)
>tr|J9XRX7|J9XRX7_9POTV Polyprotein (Fragment) OS=Zucchini yellow mosaic virus OX=12232 PE=4 SV=1
2::J9XRY1 **Mass:** 19639 **Score:** 50 **Matches:** 1(1) **Sequences:** 1(1)
>tr|J9XRY1|J9XRY1_9POTV Polyprotein (Fragment) OS=Zucchini yellow mosaic virus OX=12232 PE=4 SV=1
2::J9XRY5 **Mass:** 19639 **Score:** 50 **Matches:** 1(1) **Sequences:** 1(1)
>tr|J9XRY5|J9XRY5_9POTV Polyprotein (Fragment) OS=Zucchini yellow mosaic virus OX=12232 PE=4 SV=1
2::J9XRY7 **Mass:** 19638 **Score:** 50 **Matches:** 1(1) **Sequences:** 1(1)
>tr|J9XRY7|J9XRY7_9POTV Polyprotein (Fragment) OS=Zucchini yellow mosaic virus OX=12232 PE=4 SV=1
2::J9XRZ1 **Mass:** 19653 **Score:** 50 **Matches:** 1(1) **Sequences:** 1(1)
>tr|J9XRZ1|J9XRZ1_9POTV Polyprotein (Fragment) OS=Zucchini yellow mosaic virus OX=12232 PE=4 SV=1
2::J9XRZ6 **Mass:** 19584 **Score:** 50 **Matches:** 1(1) **Sequences:** 1(1)
>tr|J9XRZ6|J9XRZ6_9POTV Polyprotein (Fragment) OS=Zucchini yellow mosaic virus OX=12232 PE=4 SV=1
2::J9XS00 **Mass:** 19573 **Score:** 50 **Matches:** 1(1) **Sequences:** 1(1)
>tr|J9XS00|J9XS00_9POTV Polyprotein (Fragment) OS=Zucchini yellow mosaic virus OX=12232 PE=4 SV=1
2::J9XS04 **Mass:** 19639 **Score:** 50 **Matches:** 1(1) **Sequences:** 1(1)
>tr|J9XS04|J9XS04_9POTV Polyprotein (Fragment) OS=Zucchini yellow mosaic virus OX=12232 PE=4 SV=1
2::J9XS09 **Mass:** 19639 **Score:** 50 **Matches:** 1(1) **Sequences:** 1(1)
>tr|J9XS09|J9XS09_9POTV Polyprotein (Fragment) OS=Zucchini yellow mosaic virus OX=12232 PE=4 SV=1
2::J9XTU3 **Mass:** 43187 **Score:** 50 **Matches:** 1(1) **Sequences:** 1(1)
>tr|J9XTU3|J9XTU3_9POTV Polyprotein (Fragment) OS=Zucchini yellow mosaic virus OX=12232 PE=3 SV=1
2::J9XTU7 **Mass:** 43259 **Score:** 50 **Matches:** 1(1) **Sequences:** 1(1)
>tr|J9XTU7|J9XTU7_9POTV Polyprotein (Fragment) OS=Zucchini yellow mosaic virus OX=12232 PE=3 SV=1
2::J9XTV0 **Mass:** 43332 **Score:** 50 **Matches:** 1(1) **Sequences:** 1(1)
>tr|J9XTV0|J9XTV0_9POTV Polyprotein (Fragment) OS=Zucchini yellow mosaic virus OX=12232 PE=3 SV=1
2::J9XTV5 **Mass:** 19639 **Score:** 50 **Matches:** 1(1) **Sequences:** 1(1)
>tr|J9XTV5|J9XTV5_9POTV Polyprotein (Fragment) OS=Zucchini yellow mosaic virus OX=12232 PE=4 SV=1
2::J9XTW4 **Mass:** 19548 **Score:** 50 **Matches:** 1(1) **Sequences:** 1(1)
>tr|J9XTW4|J9XTW4_9POTV Polyprotein (Fragment) OS=Zucchini yellow mosaic virus OX=12232 PE=4 SV=1
2::J9XTW9 **Mass:** 19669 **Score:** 50 **Matches:** 1(1) **Sequences:** 1(1)
>tr|J9XTW9|J9XTW9_9POTV Polyprotein (Fragment) OS=Zucchini yellow mosaic virus OX=12232 PE=4 SV=1
2::J9XTX4 **Mass:** 19532 **Score:** 50 **Matches:** 1(1) **Sequences:** 1(1)
>tr|J9XTX4|J9XTX4_9POTV Polyprotein (Fragment) OS=Zucchini yellow mosaic virus OX=12232 PE=4 SV=1
2::J9XTX5 **Mass:** 19567 **Score:** 50 **Matches:** 1(1) **Sequences:** 1(1)
>tr|J9XTX5|J9XTX5_9POTV Polyprotein (Fragment) OS=Zucchini yellow mosaic virus OX=12232 PE=4 SV=1
2::J9XTY0 **Mass:** 19651 **Score:** 50 **Matches:** 1(1) **Sequences:** 1(1)
>tr|J9XTY0|J9XTY0_9POTV Polyprotein (Fragment) OS=Zucchini yellow mosaic virus OX=12232 PE=4 SV=1
2::J9XTY5 **Mass:** 19643 **Score:** 50 **Matches:** 1(1) **Sequences:** 1(1)
>tr|J9XTY5|J9XTY5_9POTV Polyprotein (Fragment) OS=Zucchini yellow mosaic virus OX=12232 PE=4 SV=1
2::J9XTY9 **Mass:** 19639 **Score:** 50 **Matches:** 1(1) **Sequences:** 1(1)
>tr|J9XTY9|J9XTY9_9POTV Polyprotein (Fragment) OS=Zucchini yellow mosaic virus OX=12232 PE=4 SV=1
2::J9XTZ4 **Mass:** 19639 **Score:** 50 **Matches:** 1(1) **Sequences:** 1(1)
>tr|J9XTZ4|J9XTZ4_9POTV Polyprotein (Fragment) OS=Zucchini yellow mosaic virus OX=12232 PE=4 SV=1
2::J9XTZ8 **Mass:** 19639 **Score:** 50 **Matches:** 1(1) **Sequences:** 1(1)
>tr|J9XTZ8|J9XTZ8_9POTV Polyprotein (Fragment) OS=Zucchini yellow mosaic virus OX=12232 PE=4 SV=1
2::J9XU00 **Mass:** 19669 **Score:** 50 **Matches:** 1(1) **Sequences:** 1(1)
>tr|J9XU00|J9XU00_9POTV Polyprotein (Fragment) OS=Zucchini yellow mosaic virus OX=12232 PE=4 SV=1
2::J9XU04 **Mass:** 19523 **Score:** 50 **Matches:** 1(1) **Sequences:** 1(1)
>tr|J9XU04|J9XU04_9POTV Polyprotein (Fragment) OS=Zucchini yellow mosaic virus OX=12232 PE=4 SV=1
2::J9XU08 **Mass:** 19651 **Score:** 50 **Matches:** 1(1) **Sequences:** 1(1)
>tr|J9XU08|J9XU08_9POTV Polyprotein (Fragment) OS=Zucchini yellow mosaic virus OX=12232 PE=4 SV=1
2::J9XU12 **Mass:** 19624 **Score:** 50 **Matches:** 1(1) **Sequences:** 1(1)
>tr|J9XU12|J9XU12_9POTV Polyprotein (Fragment) OS=Zucchini yellow mosaic virus OX=12232 PE=4 SV=1
2::J9XU15 **Mass:** 19639 **Score:** 50 **Matches:** 1(1) **Sequences:** 1(1)
>tr|J9XU15|J9XU15_9POTV Polyprotein (Fragment) OS=Zucchini yellow mosaic virus OX=12232 PE=4 SV=1
2::Q9ILY2 **Mass:** 55365 **Score:** 50 **Matches:** 1(1) **Sequences:** 1(1)
>tr|Q9ILY2|Q9ILY2_BTMV Polyprotein (Fragment) OS=Beet mosaic virus OX=114921 PE=3 SV=1
2::Q9PY73 **Mass:** 43136 **Score:** 50 **Matches:** 1(1) **Sequences:** 1(1)
>tr|Q9PY73|Q9PY73_9POTV Polyprotein (Fragment) OS=Zucchini yellow mosaic virus OX=12232 PE=3 SV=1
2::Q9WAC2 **Mass:** 353787 **Score:** 50 **Matches:** 1(1) **Sequences:** 1(1)
>tr|Q9WAC2|Q9WAC2_9POTV Genome polyprotein OS=Zucchini yellow mosaic virus OX=12232 PE=3 SV=2
2::Q9YJS4 **Mass:** 36221 **Score:** 50 **Matches:** 1(1) **Sequences:** 1(1)
>tr|Q9YJS4|Q9YJS4_9POTV Polyprotein (Fragment) OS=Zucchini yellow mosaic virus OX=12232 PE=3 SV=1
2::Q9YJW7 **Mass:** 79063 **Score:** 50 **Matches:** 1(1) **Sequences:** 1(1)
>tr|Q9YJW7|Q9YJW7_9POTV Polyprotein (Fragment) OS=Bean common mosaic virus OX=12196 PE=4 SV=1
2::Q52NV6 **Mass:** 55696 **Score:** 50 **Matches:** 1(1) **Sequences:** 1(1)
>tr|Q52NV6|Q52NV6_9POTV Polyprotein (Fragment) OS=Zucchini yellow mosaic virus OX=12232 PE=3 SV=1
2::Q80P22 **Mass:** 37775 **Score:** 50 **Matches:** 1(1) **Sequences:** 1(1)
>tr|Q80P22|Q80P22_9POTV Polyprotein (Fragment) OS=Passion fruit woodiness virus OX=31732 PE=3 SV=1
2::Q80S22 **Mass:** 54104 **Score:** 50 **Matches:** 1(1) **Sequences:** 1(1)
>tr|Q80S22|Q80S22_9POTV Polyprotein (Fragment) OS=Bean common mosaic virus OX=12196 PE=3 SV=1
2::Q91S94 **Mass:** 56321 **Score:** 50 **Matches:** 1(1) **Sequences:** 1(1)
>tr|Q91S94|Q91S94_BTMV Polyprotein (Fragment) OS=Beet mosaic virus OX=114921 PE=3 SV=1
2::Q99HQ2 **Mass:** 55882 **Score:** 50 **Matches:** 1(1) **Sequences:** 1(1)
>tr|Q99HQ2|Q99HQ2_9POTV Polyprotein (Fragment) OS=Hibbertia virus Y OX=114892 PE=3 SV=1
2::Q99HQ3 **Mass:** 55837 **Score:** 50 **Matches:** 1(1) **Sequences:** 1(1)
>tr|Q99HQ3|Q99HQ3_9POTV Polyprotein (Fragment) OS=Clitoria virus Y OX=114891 PE=3 SV=1
2::Q153H0 **Mass:** 94572 **Score:** 50 **Matches:** 1(1) **Sequences:** 1(1)
>tr|Q153H0|Q153H0_9POTV Polyprotein (Fragment) OS=Zucchini yellow mosaic virus OX=12232 PE=3 SV=1
2::Q05912 **Mass:** 116795 **Score:** 50 **Matches:** 1(1) **Sequences:** 1(1)
>tr|Q05912|Q05912_9POTV -S RNA (Fragment) OS=Zucchini yellow mosaic virus OX=12232 PE=4 SV=1
1::P18479 **Mass:** 353484 **Score:** 50 **Matches:** 1(1) **Sequences:** 1(1)
Genome polyprotein OS=Zucchini yellow mosaic virus (strain California) OX=117128 PE=1 SV=2
1::O36979 **Mass:** 353831 **Score:** 50 **Matches:** 1(1) **Sequences:** 1(1)
Genome polyprotein OS=Zucchini yellow mosaic virus (strain Singapore) OX=117130 PE=3 SV=2
1::Q65399 **Mass:** 353245 **Score:** 50 **Matches:** 1(1) **Sequences:** 1(1)

Genome polyprotein OS=Bean common mosaic necrosis virus (strain NL-3) OX=500578 PE=2 SV=1
2::Q65400 **Mass:** 39855 **Score:** 50 **Matches:** 1(1) **Sequences:** 1(1)
>tr|Q65400|Q65400_9POTV Polyprotein (Fragment) OS=Bean common mosaic virus OX=12196 PE=3 SV=1
2::Q65412 **Mass:** 34367 **Score:** 50 **Matches:** 1(1) **Sequences:** 1(1)
>tr|Q65412|Q65412_9POTV Polyprotein (Fragment) OS=Bean common mosaic virus OX=12196 PE=3 SV=1
2::O70724 **Mass:** 36475 **Score:** 50 **Matches:** 1(1) **Sequences:** 1(1)
>tr|O70724|O70724_9POTV Polyprotein (Fragment) OS=Zucchini yellow mosaic virus OX=12232 PE=3 SV=1
2::O70725 **Mass:** 36335 **Score:** 50 **Matches:** 1(1) **Sequences:** 1(1)
>tr|O70725|O70725_9POTV Polyprotein (Fragment) OS=Zucchini yellow mosaic virus OX=12232 PE=3 SV=1
2::Q86979 **Mass:** 8473 **Score:** 50 **Matches:** 1(1) **Sequences:** 1(1)
>tr|Q86979|Q86979_9POTV CP (Fragment) OS=Zucchini yellow mosaic virus OX=12232 GN=CP PE=4 SV=1
2::Q86980 **Mass:** 8572 **Score:** 50 **Matches:** 1(1) **Sequences:** 1(1)
>tr|Q86980|Q86980_9POTV CP (Fragment) OS=Zucchini yellow mosaic virus OX=12232 GN=CP PE=4 SV=1
2::Q89329 **Mass:** 52858 **Score:** 50 **Matches:** 1(1) **Sequences:** 1(1)
>tr|Q89329|Q89329_9POTV Polyprotein (Fragment) OS=Zucchini yellow mosaic virus OX=12232 PE=3 SV=1
1::Q89330 **Mass:** 354016 **Score:** 50 **Matches:** 1(1) **Sequences:** 1(1)
Genome polyprotein OS=Zucchini yellow mosaic virus (strain Reunion Island) OX=117129 PE=3 SV=1
2::Q89335 **Mass:** 50697 **Score:** 50 **Matches:** 1(1) **Sequences:** 1(1)
>tr|Q89335|Q89335_9POTV Polyprotein (Fragment) OS=Zucchini yellow mosaic virus OX=12232 PE=3 SV=1
2::Q89336 **Mass:** 39047 **Score:** 50 **Matches:** 1(1) **Sequences:** 1(1)
>tr|Q89336|Q89336_9POTV ZYMV-S coat protein (Fragment) OS=Zucchini yellow mosaic virus OX=12232 PE=2 SV=1
2::A0A1S7D5F2 **Mass:** 352285 **Score:** 50 **Matches:** 1(1) **Sequences:** 1(1)
>tr|A0A1S7D5F2|A0A1S7D5F2_BTMV Polyprotein OS=Beet mosaic virus OX=114921 PE=3 SV=1
2::A1XTA2 **Mass:** 352148 **Score:** 50 **Matches:** 1(1) **Sequences:** 1(1)
>tr|A1XTA2|A1XTA2_BTMV Polyprotein OS=Beet mosaic virus OX=114921 PE=3 SV=1

---

29. 2::A0A1V0SKV6 **Mass:** 61089 **Score:** 50 **Matches:** 1(1) **Sequences:** 1(1) **emPAI:** 0.07
>tr|A0A1V0SKV6|A0A1V0SKV6_9VIRU Uncharacterized protein OS=Klosneuvirus KNV1 OX=1977640 GN=Klosneuvirus_5_30 PE=4 SV=1

Query	Observed	Mr(expt)	Mr(calc)	ppm	Miss	Score	Expect	Rank	Unique	Peptide
27	360.7189	719.4232	719.4218	2.03	0	50	0.0015	1	U	R.IPYSLK.Y

Proteins matching the same set of peptides:
2::A0A385I731 **Mass:** 203583 **Score:** 50 **Matches:** 1(1) **Sequences:** 1(1)
>tr|A0A385I731|A0A385I731_9RETR Glycoprotein OS=Saesbyeol virus OX=2320522 PE=4 SV=1
2::A0A5J6VJE5 **Mass:** 45156 **Score:** 50 **Matches:** 1(1) **Sequences:** 1(1)
>tr|A0A5J6VJE5|A0A5J6VJE5_9VIRU Elongation factor Tu GTP binding domain protein OS=Megaviridae environmental sample OX=1737588 PE=4 SV=1

---

30. 2::D7SGS7 **Mass:** 46496 **Score:** 50 **Matches:** 1(1) **Sequences:** 1(1) **emPAI:** 0.09
>tr|D7SGS7|D7SGS7_9MONO Polymerase (Fragment) OS=Paramyxovirus bat/GH47/2008 OX=589524 GN=L PE=4 SV=1

Query	Observed	Mr(expt)	Mr(calc)	ppm	Miss	Score	Expect	Rank	Unique	Peptide
695	516.3037	1030.5929	1030.5910	1.87	0	50	0.0037	1	U	K.VIDLETTLK.T

Proteins matching the same set of peptides:
2::K7XAQ9 **Mass:** 259901 **Score:** 50 **Matches:** 1(1) **Sequences:** 1(1)
>tr|K7XAQ9|K7XAQ9_9MONO RNA-directed RNA polymerase L OS=Achimota virus 1 OX=1261100 GN=L PE=3 SV=1

---

31. 2::A0A160DAA3 **Mass:** 154217 **Score:** 50 **Matches:** 1(1) **Sequences:** 1(1) **emPAI:** 0.03
>tr|A0A160DAA3|A0A160DAA3_9ALPC Spike glycoprotein OS=Porcine epidemic diarrhea virus OX=28295 GN=S PE=2 SV=1

Query	Observed	Mr(expt)	Mr(calc)	ppm	Miss	Score	Expect	Rank	Unique	Peptide
51	365.7137	729.4128	729.4133	-0.67	0	50	0.0095	1	U	K.GLNTVAR.A

Proteins matching the same set of peptides:
2::A0A165XVM7 **Mass:** 154063 **Score:** 50 **Matches:** 1(1) **Sequences:** 1(1)
>tr|A0A165XVM7|A0A165XVM7_9ALPC Spike glycoprotein OS=Porcine epidemic diarrhea virus OX=28295 GN=S PE=3 SV=1
2::A0A165XVS3 **Mass:** 154091 **Score:** 50 **Matches:** 1(1) **Sequences:** 1(1)
>tr|A0A165XVS3|A0A165XVS3_9ALPC Spike glycoprotein OS=Porcine epidemic diarrhea virus OX=28295 GN=S PE=3 SV=1
2::A0A165XVY6 **Mass:** 154074 **Score:** 50 **Matches:** 1(1) **Sequences:** 1(1)
>tr|A0A165XVY6|A0A165XVY6_9ALPC Spike glycoprotein OS=Porcine epidemic diarrhea virus OX=28295 GN=S PE=3 SV=1
2::A0A172PC26 **Mass:** 154093 **Score:** 50 **Matches:** 1(1) **Sequences:** 1(1)
>tr|A0A172PC26|A0A172PC26_9ALPC Spike glycoprotein OS=Porcine epidemic diarrhea virus OX=28295 GN=S PE=3 SV=1
2::A0A2H5AC33 **Mass:** 154491 **Score:** 50 **Matches:** 1(1) **Sequences:** 1(1)
>tr|A0A2H5AC33|A0A2H5AC33_9ALPC Spike glycoprotein OS=Porcine epidemic diarrhea virus OX=28295 GN=S PE=3 SV=1
2::A0A2H5AC45 **Mass:** 154543 **Score:** 50 **Matches:** 1(1) **Sequences:** 1(1)
>tr|A0A2H5AC45|A0A2H5AC45_9ALPC Spike glycoprotein OS=Porcine epidemic diarrhea virus OX=28295 GN=S PE=3 SV=1
2::A0A2R4H2R8 **Mass:** 154533 **Score:** 50 **Matches:** 1(1) **Sequences:** 1(1)
>tr|A0A2R4H2R8|A0A2R4H2R8_9ALPC Spike glycoprotein OS=Porcine epidemic diarrhea virus OX=28295 GN=S PE=3 SV=1

---

32. 2::A0A0A0YX30 **Mass:** 44330 **Score:** 50 **Matches:** 1(1) **Sequences:** 1(1) **emPAI:** 0.10
>tr|A0A0A0YX30|A0A0A0YX30_9CAUD Integrase OS=Streptococcus phage SpSL1 OX=1566990 GN=int PE=4 SV=1

Query	Observed	Mr(expt)	Mr(calc)	ppm	Miss	Score	Expect	Rank	Unique	Peptide
36	361.7375	721.4604	721.4599	0.77	0	50	0.00027	1	U	R.AILHLR.D

Proteins matching the same set of peptides:
2::A0A126GGK4 **Mass:** 44390 **Score:** 50 **Matches:** 1(1) **Sequences:** 1(1)
>tr|A0A126GGK4|A0A126GGK4_9CAUD Integrase_AP2 domain protein OS=Streptococcus phage phiARI0578 OX=1701832 GN=phiARI0578_34 PE=4 SV=1
2::A0A126GH03 **Mass:** 43221 **Score:** 50 **Matches:** 1(1) **Sequences:** 1(1)
>tr|A0A126GH03|A0A126GH03_9CAUD Integrase (Fragment) OS=Streptococcus phage phiARI0285-2 OX=1701818 GN=phiARI0285-2_42 PE=4 SV=1
2::A0A141DZD4 **Mass:** 44335 **Score:** 50 **Matches:** 1(1) **Sequences:** 1(1)
>tr|A0A141DZD4|A0A141DZD4_9CAUD Phage_integrase domain-containing protein OS=Streptococcus phage phiARI0923 OX=1701842 GN=phiARI0923_36 PE=4 SV=1
2::A0A141E0Q7 **Mass:** 44297 **Score:** 50 **Matches:** 1(1) **Sequences:** 1(1)
>tr|A0A141E0Q7|A0A141E0Q7_9CAUD Int_SAM_3 domain protein OS=Streptococcus phage phiARI0131-2 OX=1701814 GN=phiARI0131-2_38 PE=4 SV=1
2::A0A141E192 **Mass:** 44303 **Score:** 50 **Matches:** 1(1) **Sequences:** 1(1)
>tr|A0A141E192|A0A141E192_9CAUD Integrase OS=Streptococcus phage phiARI0995b OX=1701844 GN=phiARI0995b_34 PE=4 SV=1
2::A0A1S5S862 **Mass:** 44423 **Score:** 50 **Matches:** 1(1) **Sequences:** 1(1)
>tr|A0A1S5S862|A0A1S5S862_9CAUD Integrase OS=Streptococcus phage IPP11 OX=1916152 GN=IPP11_00001 PE=4 SV=1
2::A0A1S5S8M0 **Mass:** 44349 **Score:** 50 **Matches:** 1(1) **Sequences:** 1(1)
>tr|A0A1S5S8M0|A0A1S5S8M0_9CAUD Integrase OS=Streptococcus phage IPP12 OX=1916153 GN=IPP12_00001 PE=4 SV=1
2::A0A1S5S8Y2 **Mass:** 44345 **Score:** 50 **Matches:** 1(1) **Sequences:** 1(1)
>tr|A0A1S5S8Y2|A0A1S5S8Y2_9CAUD Integrase OS=Streptococcus phage IPP17 OX=1916157 GN=IPP17_00001 PE=4 SV=1
2::A0A1S5S994 **Mass:** 44335 **Score:** 50 **Matches:** 1(1) **Sequences:** 1(1)
>tr|A0A1S5S994|A0A1S5S994_9CAUD Integrase OS=Streptococcus phage IPP18 OX=1916158 GN=IPP18_00001 PE=4 SV=1
2::A0A1S5S9I3 **Mass:** 44353 **Score:** 50 **Matches:** 1(1) **Sequences:** 1(1)
>tr|A0A1S5S9I3|A0A1S5S9I3_9CAUD Integrase OS=Streptococcus phage IPP20 OX=1916160 GN=IPP20_00001 PE=4 SV=1
2::A0A1S5S9J0 **Mass:** 44408 **Score:** 50 **Matches:** 1(1) **Sequences:** 1(1)
>tr|A0A1S5S9J0|A0A1S5S9J0_9CAUD Integrase OS=Streptococcus phage IPP19 OX=1916159 GN=IPP19_00001 PE=4 SV=1
2::A0A1S5S9M9 **Mass:** 44380 **Score:** 50 **Matches:** 1(1) **Sequences:** 1(1)
>tr|A0A1S5S9M9|A0A1S5S9M9_9CAUD Integrase OS=Streptococcus phage IPP21 OX=1916161 GN=IPP21_00001 PE=4 SV=1
2::A0A1S5SA16 **Mass:** 44334 **Score:** 50 **Matches:** 1(1) **Sequences:** 1(1)
>tr|A0A1S5SA16|A0A1S5SA16_9CAUD Integrase OS=Streptococcus phage IPP22 OX=1916162 GN=IPP22_00001 PE=4 SV=1
2::A0A1S5SAT1 **Mass:** 44379 **Score:** 50 **Matches:** 1(1) **Sequences:** 1(1)
>tr|A0A1S5SAT1|A0A1S5SAT1_9CAUD Integrase OS=Streptococcus phage IPP29 OX=1916169 GN=IPP29_00001 PE=4 SV=1
2::A0A1S5SAV6 **Mass:** 44394 **Score:** 50 **Matches:** 1(1) **Sequences:** 1(1)
>tr|A0A1S5SAV6|A0A1S5SAV6_9CAUD Integrase OS=Streptococcus phage IPP28 OX=1916168 GN=IPP28_00001 PE=4 SV=1
2::A0A1S5SAZ8 **Mass:** 44349 **Score:** 50 **Matches:** 1(1) **Sequences:** 1(1)
>tr|A0A1S5SAZ8|A0A1S5SAZ8_9CAUD Integrase OS=Streptococcus phage IPP30 OX=1916170 GN=IPP30_00001 PE=4 SV=1
2::A0A1S5SCF9 **Mass:** 44362 **Score:** 50 **Matches:** 1(1) **Sequences:** 1(1)

>tr|A0A1S5SCF9|A0A1S5SCF9_9CAUD Integrase OS=Streptococcus phage IPP41 OX=1916180 GN=IPP41_00001 PE=4 SV=1
2::A0A1S5SCM1 **Mass:** 44337 **Score:** 50 **Matches:** 1(1) **Sequences:** 1(1)
>tr|A0A1S5SCM1|A0A1S5SCM1_9CAUD Integrase OS=Streptococcus phage IPP42 OX=1916181 GN=IPP42_00001 PE=4 SV=1
2::A0A1S5SCV4 **Mass:** 44335 **Score:** 50 **Matches:** 1(1) **Sequences:** 1(1)
>tr|A0A1S5SCV4|A0A1S5SCV4_9CAUD Integrase OS=Streptococcus phage IPP44 OX=1916183 GN=IPP44_00001 PE=4 SV=1
2::A0A1S5SD03 **Mass:** 44303 **Score:** 50 **Matches:** 1(1) **Sequences:** 1(1)
>tr|A0A1S5SD03|A0A1S5SD03_9CAUD Integrase OS=Streptococcus phage IPP45 OX=1916184 GN=IPP45_00001 PE=4 SV=1
2::A0A1S5SD13 **Mass:** 44349 **Score:** 50 **Matches:** 1(1) **Sequences:** 1(1)
>tr|A0A1S5SD13|A0A1S5SD13_9CAUD Integrase OS=Streptococcus phage IPP43 OX=1916182 GN=IPP43_00001 PE=4 SV=1
2::A0A1S5SDZ6 **Mass:** 44337 **Score:** 50 **Matches:** 1(1) **Sequences:** 1(1)
>tr|A0A1S5SDZ6|A0A1S5SDZ6_9CAUD Integrase OS=Streptococcus phage IPP51 OX=1916189 GN=IPP51_00001 PE=4 SV=1
2::A0A1S5SEC4 **Mass:** 44335 **Score:** 50 **Matches:** 1(1) **Sequences:** 1(1)
>tr|A0A1S5SEC4|A0A1S5SEC4_9CAUD Integrase OS=Streptococcus phage IPP57 OX=1916194 GN=IPP57_00001 PE=4 SV=1
2::A0A1S5SEH2 **Mass:** 44335 **Score:** 50 **Matches:** 1(1) **Sequences:** 1(1)
>tr|A0A1S5SEH2|A0A1S5SEH2_9CAUD Integrase OS=Streptococcus phage IPP58 OX=1916195 GN=IPP58_00001 PE=4 SV=1
2::A0A1S5SF65 **Mass:** 44349 **Score:** 50 **Matches:** 1(1) **Sequences:** 1(1)
>tr|A0A1S5SF65|A0A1S5SF65_9CAUD Integrase OS=Streptococcus phage IPP62 OX=1916198 GN=IPP62_00001 PE=4 SV=1
2::A0A1S5SFI5 **Mass:** 44294 **Score:** 50 **Matches:** 1(1) **Sequences:** 1(1)
>tr|A0A1S5SFI5|A0A1S5SFI5_9CAUD Integrase OS=Streptococcus phage IPP63 OX=1916199 GN=IPP63_00001 PE=4 SV=1
2::A0A1S5SFX6 **Mass:** 44313 **Score:** 50 **Matches:** 1(1) **Sequences:** 1(1)
>tr|A0A1S5SFX6|A0A1S5SFX6_9CAUD Integrase OS=Streptococcus phage IPP67 OX=1916203 GN=IPP67_00001 PE=4 SV=1
2::A0A1S5SG51 **Mass:** 44303 **Score:** 50 **Matches:** 1(1) **Sequences:** 1(1)
>tr|A0A1S5SG51|A0A1S5SG51_9CAUD Integrase OS=Streptococcus phage IPP68 OX=1916204 GN=IPP68_00001 PE=4 SV=1
2::A0A4D6A727 **Mass:** 44302 **Score:** 50 **Matches:** 1(1) **Sequences:** 1(1)
>tr|A0A4D6A727|A0A4D6A727_9CAUD Mobile element protein OS=Streptococcus phage Javan331 OX=2548111 GN=Javan331_0055 PE=4 SV=1
2::A0A4D6A7C8 **Mass:** 44372 **Score:** 50 **Matches:** 1(1) **Sequences:** 1(1)
>tr|A0A4D6A7C8|A0A4D6A7C8_9CAUD Mobile element protein OS=Streptococcus phage Javan347 OX=2548117 GN=Javan347_0045 PE=4 SV=1
2::A0A4D6A817 **Mass:** 44377 **Score:** 50 **Matches:** 1(1) **Sequences:** 1(1)
>tr|A0A4D6A817|A0A4D6A817_9CAUD Mobile element protein OS=Streptococcus phage Javan363 OX=2548126 GN=Javan363_0044 PE=4 SV=1
2::A0A4D6B0V3 **Mass:** 44258 **Score:** 50 **Matches:** 1(1) **Sequences:** 1(1)
>tr|A0A4D6B0V3|A0A4D6B0V3_9CAUD Mobile element protein OS=Streptococcus phage Javan348 OX=2548118 GN=Javan348_0001 PE=4 SV=1
2::A0A4D6B218 **Mass:** 38223 **Score:** 50 **Matches:** 1(1) **Sequences:** 1(1)
>tr|A0A4D6B218|A0A4D6B218_9CAUD Mobile element protein OS=Streptococcus phage Javan350 OX=2548121 GN=Javan350_0001 PE=4 SV=1
2::A0A4D6B4S0 **Mass:** 44277 **Score:** 50 **Matches:** 1(1) **Sequences:** 1(1)
>tr|A0A4D6B4S0|A0A4D6B4S0_9CAUD Mobile element protein OS=Streptococcus phage Javan366 OX=2548127 GN=Javan366_0001 PE=4 SV=1
2::Q3BLW3 **Mass:** 44302 **Score:** 50 **Matches:** 1(1) **Sequences:** 1(1)
>tr|Q3BLW3|Q3BLW3_9VIRU Integrase OS=Phage SK137 OX=334523 GN=int PE=4 SV=1
2::C5J953 **Mass:** 44362 **Score:** 50 **Matches:** 1(1) **Sequences:** 1(1)
>tr|C5J953|C5J953_9CAUD Uncharacterized protein OS=Streptococcus phage PH10 OX=644007 PE=4 SV=1
2::E8ZDN6 **Mass:** 44371 **Score:** 50 **Matches:** 1(1) **Sequences:** 1(1)
>tr|E8ZDN6|E8ZDN6_9VIRU Integrase OS=Streptococcus phage 23782 OX=870472 PE=4 SV=1
2::E8ZDT4 **Mass:** 44303 **Score:** 50 **Matches:** 1(1) **Sequences:** 1(1)
>tr|E8ZDT4|E8ZDT4_9VIRU Integrase OS=Streptococcus phage 11865 OX=870473 PE=4 SV=1

---

33. 2::A0A482JV29 **Mass:** 224784 **Score:** 49 **Matches:** 1(1) **Sequences:** 1(1) **emPAI:** 0.02
>tr|A0A482JV29|A0A482JV29_9RHAB RNA-directed RNA polymerase L OS=Lampyris noctiluca rhabdo-like virus 1 OX=2552994 PE=3 SV=1

Query	Observed	Mr(expt)	Mr(calc)	ppm	Miss	Score	Expect	Rank	Unique	Peptide
368	445.7221	889.4295	889.4294	0.20	0	49	0.0042	1	U	K.EWIATDR.A

---

34. 2::A0A076JU09 **Mass:** 84694 **Score:** 49 **Matches:** 1(1) **Sequences:** 1(1) **emPAI:** 0.05
>tr|A0A076JU09|A0A076JU09_9ALPH Portal protein OS=Equid alphaherpesvirus 1 OX=10326 PE=3 SV=1

Query	Observed	Mr(expt)	Mr(calc)	ppm	Miss	Score	Expect	Rank	Unique	Peptide
71	373.7056	745.3967	745.3970	-0.43	0	49	0.0066	1	U	K.TIEDLR.T

Proteins matching the same set of peptides:
2::A0A0A7D9M5 **Mass:** 84568 **Score:** 49 **Matches:** 1(1) **Sequences:** 1(1)
>tr|A0A0A7D9M5|A0A0A7D9M5_9ALPH Portal protein OS=Equid alphaherpesvirus 1 OX=10326 GN=ORF56 PE=3 SV=1
2::A0A0A7DAD3 **Mass:** 84554 **Score:** 49 **Matches:** 1(1) **Sequences:** 1(1)
>tr|A0A0A7DAD3|A0A0A7DAD3_9ALPH Portal protein OS=Equid alphaherpesvirus 1 OX=10326 GN=ORF56 PE=3 SV=1
2::A0A0X9ZHI2 **Mass:** 84184 **Score:** 49 **Matches:** 1(1) **Sequences:** 1(1)
>tr|A0A0X9ZHI2|A0A0X9ZHI2_9ALPH Portal protein OS=Equid alphaherpesvirus 4 OX=10331 GN=ORF56 PE=3 SV=1
2::A0A0Y0A4E8 **Mass:** 84170 **Score:** 49 **Matches:** 1(1) **Sequences:** 1(1)
>tr|A0A0Y0A4E8|A0A0Y0A4E8_9ALPH Portal protein OS=Equid alphaherpesvirus 4 OX=10331 GN=ORF56 PE=3 SV=1
2::A0A1J1E0I6 **Mass:** 84210 **Score:** 49 **Matches:** 1(1) **Sequences:** 1(1)
>tr|A0A1J1E0I6|A0A1J1E0I6_9ALPH Portal protein OS=Equid alphaherpesvirus 4 OX=10331 GN=ORF56 PE=3 SV=1
2::A0A2K9QRI7 **Mass:** 84599 **Score:** 49 **Matches:** 1(1) **Sequences:** 1(1)
>tr|A0A2K9QRI7|A0A2K9QRI7_9ALPH Portal protein OS=Equid alphaherpesvirus 8 OX=39637 GN=ORF56 PE=3 SV=1
2::A0A2K9QRR7 **Mass:** 84666 **Score:** 49 **Matches:** 1(1) **Sequences:** 1(1)
>tr|A0A2K9QRR7|A0A2K9QRR7_9ALPH Portal protein OS=Equid alphaherpesvirus 8 OX=39637 GN=ORF56 PE=3 SV=1
2::A0A2P0N522 **Mass:** 84610 **Score:** 49 **Matches:** 1(1) **Sequences:** 1(1)
>tr|A0A2P0N522|A0A2P0N522_9ALPH Portal protein OS=Equid alphaherpesvirus 1 OX=10326 GN=ORF56 PE=3 SV=1
2::A0A2P0N7P0 **Mass:** 84568 **Score:** 49 **Matches:** 1(1) **Sequences:** 1(1)
>tr|A0A2P0N7P0|A0A2P0N7P0_9ALPH Portal protein OS=Equid alphaherpesvirus 1 OX=10326 GN=ORF56 PE=3 SV=1
2::A0A2P0N8A0 **Mass:** 84525 **Score:** 49 **Matches:** 1(1) **Sequences:** 1(1)
>tr|A0A2P0N8A0|A0A2P0N8A0_9ALPH Portal protein OS=Equid alphaherpesvirus 1 OX=10326 GN=ORF56 PE=3 SV=1
2::A0A516LDA7 **Mass:** 97041 **Score:** 49 **Matches:** 1(1) **Sequences:** 1(1)
>tr|A0A516LDA7|A0A516LDA7_9VIRU Putative DNA ligase OS=Prokaryotic dsDNA virus sp. OX=2591644 GN=GOVbin4206_77 PE=4 SV=1
2::I1V8H0 **Mass:** 77644 **Score:** 49 **Matches:** 1(1) **Sequences:** 1(1)
>tr|I1V8H0|I1V8H0_9ALPH Portal protein OS=Equid alphaherpesvirus 8 OX=39637 GN=ORF56 PE=3 SV=1
2::M4JG74 **Mass:** 157566 **Score:** 49 **Matches:** 1(1) **Sequences:** 1(1)
>tr|M4JG74|M4JG74_9VIRU Capsid protein VP1 OS=Haloarcula hispanica virus PH1 OX=1282967 GN=HhPH1_gp12 PE=4 SV=1
2::B7FEJ8 **Mass:** 77689 **Score:** 49 **Matches:** 1(1) **Sequences:** 1(1)
>tr|B7FEJ8|B7FEJ8_9ALPH Portal protein OS=Equid alphaherpesvirus 9 OX=55744 PE=3 SV=1
2::L8B1V9 **Mass:** 84640 **Score:** 49 **Matches:** 1(1) **Sequences:** 1(1)
>tr|L8B1V9|L8B1V9_9ALPH Portal protein OS=Equid alphaherpesvirus 1 OX=10326 GN=ORF56 PE=3 SV=1
1::P28944 **Mass:** 84568 **Score:** 49 **Matches:** 1(1) **Sequences:** 1(1)
Portal protein OS=Equine herpesvirus 1 (strain Ab4p) OX=31520 GN=56 PE=3 SV=1
2::O39296 **Mass:** 84212 **Score:** 49 **Matches:** 1(1) **Sequences:** 1(1)
>tr|O39296|O39296_EHV4 Portal protein OS=Equine herpesvirus 4 (strain 1942) OX=10333 GN=56 PE=3 SV=1
1::P84458 **Mass:** 84568 **Score:** 49 **Matches:** 1(1) **Sequences:** 1(1)
Portal protein OS=Equine herpesvirus 1 (strain V592) OX=310273 GN=56 PE=3 SV=1

---

35. 2::A8ATK7 **Mass:** 74572 **Score:** 49 **Matches:** 1(1) **Sequences:** 1(1) **emPAI:** 0.06
>tr|A8ATK7|A8ATK7_9CAUD Gp49 OS=Listeria phage B054 OX=330397 PE=4 SV=1

Query	Observed	Mr(expt)	Mr(calc)	ppm	Miss	Score	Expect	Rank	Unique	Peptide
739	526.2746	1050.5346	1050.5345	0.03	1	49	0.0082	1	U	K.KEEIFTER.N

---

36. 2::A0A2I7QYQ0 **Mass:** 26830 **Score:** 49 **Matches:** 1(1) **Sequences:** 1(1) **emPAI:** 0.17
>tr|A0A2I7QYQ0|A0A2I7QYQ0_9CAUD Uncharacterized protein OS=Vibrio phage 1.086.O._10N.222.51.F8 OX=1881424 GN=NVP1086O_01 PE=4 SV=1

Query	Observed	Mr(expt)	Mr(calc)	ppm	Miss	Score	Expect	Rank	Unique	Peptide
502	479.2922	956.5699	956.5655	4.64	0	49	0.0058	1	U	R.IAELVGISR.Q

Proteins matching the same set of peptides:
2::A0A2I7RH50 **Mass:** 26720 **Score:** 49 **Matches:** 1(1) **Sequences:** 1(1)
>tr|A0A2I7RH50|A0A2I7RH50_9CAUD Uncharacterized protein OS=Vibrio phage 1.181.O._10N.286.46.C9 OX=1881433 GN=NVP1181O_02 PE=4 SV=1
2::A0A2I7RK87 **Mass:** 26857 **Score:** 49 **Matches:** 1(1) **Sequences:** 1(1)
>tr|A0A2I7RK87|A0A2I7RK87_9CAUD Uncharacterized protein OS=Vibrio phage 1.191.O._10N.286.52.B4 OX=1881435 GN=NVP1191O_01 PE=4 SV=1
2::A0A2I7RS65 **Mass:** 26692 **Score:** 49 **Matches:** 1(1) **Sequences:** 1(1)
>tr|A0A2I7RS65|A0A2I7RS65_9CAUD Uncharacterized protein OS=Vibrio phage 1.225.O._10N.261.48.B7 OX=1881440 GN=NVP1225O_01 PE=4 SV=1

---

37. 2::A0A2Z2CKJ5 **Mass:** 134247 **Score:** 48 **Matches:** 1(1) **Sequences:** 1(1) **emPAI:** 0.03
>tr|A0A2Z2CKJ5|A0A2Z2CKJ5_9VIRU Glycoprotein OS=Pidgey virus OX=1911436 PE=4 SV=1

Query	Observed	Mr(expt)	Mr(calc)	ppm	Miss	Score	Expect	Rank	Unique	Peptide
69	373.2134	744.4123	744.4130	-0.89	0	48	0.023	1	U	R.EVTQLR.A

Proteins matching the same set of peptides:
2::Q90QL4 **Mass:** 12443 **Score:** 48 **Matches:** 1(1) **Sequences:** 1(1)
>tr|Q90QL4|Q90QL4_9HIV1 Envelope glycoprotein (Fragment) OS=Human immunodeficiency virus 1 OX=11676 GN=env PE=4 SV=1

---

38. 2::A0A0K0KVG1 **Mass:** 284825 **Score:** 46 **Matches:** 1(1) **Sequences:** 1(1) **emPAI:** 0.02
>tr|A0A0K0KVG1|A0A0K0KVG1_9CAUD Putative neck protein OS=Prochlorococcus phage P-TIM68 OX=1542477 PE=4 SV=1

Query	Observed	Mr(expt)	Mr(calc)	ppm	Miss	Score	Expect	Rank	Unique	Peptide
122	386.7212	771.4279	771.4279	-0.06	0	46	0.0095	1	U	K.FDIHIK.D

---

39. 2::A0A1D8MBQ4 **Mass:** 76569 **Score:** 45 **Matches:** 1(1) **Sequences:** 1(1) **emPAI:** 0.06
>tr|A0A1D8MBQ4|A0A1D8MBQ4_9VIRU Polyprotein (Fragment) OS=Endornavirus-like virus OX=1906952 PE=4 SV=1

Query	Observed	Mr(expt)	Mr(calc)	ppm	Miss	Score	Expect	Rank	Unique	Peptide
218	413.2757	824.5368	824.5371	-0.42	0	45	0.00034	1	U	K.LIEIPLK.Q

---

40. 2::A0A481ZC74 **Mass:** 29404 **Score:** 45 **Matches:** 1(1) **Sequences:** 1(1) **emPAI:** 0.15
>tr|A0A481ZC74|A0A481ZC74_9VIRU Uncharacterized protein OS=Pithovirus LCPAC401 OX=2506595 GN=LCPAC401_03270 PE=4 SV=1

Query	Observed	Mr(expt)	Mr(calc)	ppm	Miss	Score	Expect	Rank	Unique	Peptide
208	412.2315	822.4484	822.4487	-0.40	0	45	0.004	1	U	R.TYAEIVK.E

---

41. 2::A0A3G1QTM4 **Mass:** 381032 **Score:** 45 **Matches:** 1(1) **Sequences:** 1(1) **emPAI:** 0.01
>tr|A0A3G1QTM4|A0A3G1QTM4_9CAUD Virion-associated RNA polymerase OS=Erwinia phage phiEaP-8 OX=2178928 PE=4 SV=1

Query	Observed	Mr(expt)	Mr(calc)	ppm	Miss	Score	Expect	Rank	Unique	Peptide
272	424.2495	846.4845	846.4851	-0.66	0	45	0.0087	1	U	K.LFTPEIK.D

---

42. 2::A0A2I6BQV1 **Mass:** 43876 **Score:** 44 **Matches:** 1(1) **Sequences:** 1(1) **emPAI:** 0.10
>tr|A0A2I6BQV1|A0A2I6BQV1_9POXV Uncharacterized protein OS=White-tailed deer poxvirus OX=2067573 GN=DPV106 PE=4 SV=1

Query	Observed	Mr(expt)	Mr(calc)	ppm	Miss	Score	Expect	Rank	Unique	Peptide
136	391.2499	780.4852	780.4858	-0.69	0	44	0.0016	1	U	R.HLIGLTK.D

Proteins matching the same set of peptides:
2::A0A3S7SW56 **Mass:** 43876 **Score:** 44 **Matches:** 1(1) **Sequences:** 1(1)
>tr|A0A3S7SW56|A0A3S7SW56_9POXV Uncharacterized protein OS=Moosepox virus GoldyGopher14 OX=2340996 PE=4 SV=1
2::Q08F76 **Mass:** 43876 **Score:** 44 **Matches:** 1(1) **Sequences:** 1(1)
>tr|Q08F76|Q08F76_DPV84 Uncharacterized protein OS=Deerpox virus (strain W-1170-84) OX=305676 GN=DpV84gp106 PE=4 SV=1
2::Q08FP6 **Mass:** 43876 **Score:** 44 **Matches:** 1(1) **Sequences:** 1(1)
>tr|Q08FP6|Q08FP6_DPV83 Uncharacterized protein OS=Deerpox virus (strain Mule deer/United States/W-848-83/1983) OX=305674 GN=DpV83gp106 PE=4 SV=1

---

43. 2::A0A0G3Y4Q4 **Mass:** 68500 **Score:** 44 **Matches:** 1(0) **Sequences:** 1(0)
>tr|A0A0G3Y4Q4|A0A0G3Y4Q4_OSHV1 ORF67 OS=Ostreid herpesvirus 1 OX=261939 PE=4 SV=1

Query	Observed	Mr(expt)	Mr(calc)	ppm	Miss	Score	Expect	Rank	Unique	Peptide
70	373.2136	744.4126	744.4130	-0.46	0	44	0.065	1	U	R.GGLLTER.C

Proteins matching the same set of peptides:
2::A0A220QKS8 **Mass:** 68472 **Score:** 44 **Matches:** 1(0) **Sequences:** 1(0)
>tr|A0A220QKS8|A0A220QKS8_OSHV1 ORF67 OS=Ostreid herpesvirus 1 OX=261939 PE=4 SV=1
2::A0A220QL46 **Mass:** 68486 **Score:** 44 **Matches:** 1(0) **Sequences:** 1(0)
>tr|A0A220QL46|A0A220QL46_OSHV1 ORF67 OS=Ostreid herpesvirus 1 OX=261939 PE=4 SV=1
2::A0A2R3WI06 **Mass:** 68498 **Score:** 44 **Matches:** 1(0) **Sequences:** 1(0)
>tr|A0A2R3WI06|A0A2R3WI06_OSHV1 ORF67 OS=Ostreid herpesvirus 1 OX=261939 PE=4 SV=1
2::F6J0A1 **Mass:** 68514 **Score:** 44 **Matches:** 1(0) **Sequences:** 1(0)
>tr|F6J0A1|F6J0A1_9VIRU ORF66 OS=Chlamys acute necrobiotic virus OX=741995 PE=4 SV=1
1::Q6R7G1 **Mass:** 68428 **Score:** 44 **Matches:** 1(0) **Sequences:** 1(0)
Uncharacterized protein ORF67 OS=Ostreid herpesvirus 1 (isolate France) OX=654903 GN=ORF67 PE=4 SV=1

---

44. 2::A0A0F6NAG1 **Mass:** 52762 **Score:** 44 **Matches:** 1(1) **Sequences:** 1(1) **emPAI:** 0.08
>tr|A0A0F6NAG1|A0A0F6NAG1_9POTV Helper component protein (Fragment) OS=Banana bract mosaic virus OX=45661 PE=4 SV=1

Query	Observed	Mr(expt)	Mr(calc)	ppm	Miss	Score	Expect	Rank	Unique	Peptide
49	365.2169	728.4192	728.4181	1.56	0	44	0.059	1	U	K.IDVINR.T

Proteins matching the same set of peptides:
2::A0A0F6NAG2 **Mass:** 52733 **Score:** 44 **Matches:** 1(1) **Sequences:** 1(1)
>tr|A0A0F6NAG2|A0A0F6NAG2_9POTV Helper component protein (Fragment) OS=Banana bract mosaic virus OX=45661 PE=4 SV=1
2::A0A0F6NAG3 **Mass:** 52752 **Score:** 44 **Matches:** 1(1) **Sequences:** 1(1)
>tr|A0A0F6NAG3|A0A0F6NAG3_9POTV Helper component protein (Fragment) OS=Banana bract mosaic virus OX=45661 PE=4 SV=1
2::A0A0F6NAG4 **Mass:** 52632 **Score:** 44 **Matches:** 1(1) **Sequences:** 1(1)
>tr|A0A0F6NAG4|A0A0F6NAG4_9POTV Helper component protein (Fragment) OS=Banana bract mosaic virus OX=45661 PE=4 SV=1
2::A0A0F6NAG5 **Mass:** 52752 **Score:** 44 **Matches:** 1(1) **Sequences:** 1(1)
>tr|A0A0F6NAG5|A0A0F6NAG5_9POTV Helper component protein (Fragment) OS=Banana bract mosaic virus OX=45661 PE=4 SV=1
2::A0A0F6NAG6 **Mass:** 52722 **Score:** 44 **Matches:** 1(1) **Sequences:** 1(1)
>tr|A0A0F6NAG6|A0A0F6NAG6_9POTV Helper component protein (Fragment) OS=Banana bract mosaic virus OX=45661 PE=4 SV=1
2::A0A0F6NAL6 **Mass:** 52778 **Score:** 44 **Matches:** 1(1) **Sequences:** 1(1)
>tr|A0A0F6NAL6|A0A0F6NAL6_9POTV Helper component protein (Fragment) OS=Banana bract mosaic virus OX=45661 PE=4 SV=1
2::A0A0F6NAL9 **Mass:** 52714 **Score:** 44 **Matches:** 1(1) **Sequences:** 1(1)
>tr|A0A0F6NAL9|A0A0F6NAL9_9POTV Helper component protein (Fragment) OS=Banana bract mosaic virus OX=45661 PE=4 SV=1
2::A0A0F6NAQ3 **Mass:** 52877 **Score:** 44 **Matches:** 1(1) **Sequences:** 1(1)
>tr|A0A0F6NAQ3|A0A0F6NAQ3_9POTV Helper component protein (Fragment) OS=Banana bract mosaic virus OX=45661 PE=4 SV=1
2::A0A0F6NAQ7 **Mass:** 52851 **Score:** 44 **Matches:** 1(1) **Sequences:** 1(1)
>tr|A0A0F6NAQ7|A0A0F6NAQ7_9POTV Helper component protein (Fragment) OS=Banana bract mosaic virus OX=45661 PE=4 SV=1
2::A0A0F6NAQ9 **Mass:** 52749 **Score:** 44 **Matches:** 1(1) **Sequences:** 1(1)
>tr|A0A0F6NAQ9|A0A0F6NAQ9_9POTV Helper component protein (Fragment) OS=Banana bract mosaic virus OX=45661 PE=4 SV=1
2::A0A0F6NAY7 **Mass:** 52748 **Score:** 44 **Matches:** 1(1) **Sequences:** 1(1)
>tr|A0A0F6NAY7|A0A0F6NAY7_9POTV Helper component protein (Fragment) OS=Banana bract mosaic virus OX=45661 PE=4 SV=1
2::A0A0F6NAY8 **Mass:** 52767 **Score:** 44 **Matches:** 1(1) **Sequences:** 1(1)
>tr|A0A0F6NAY8|A0A0F6NAY8_9POTV Helper component protein (Fragment) OS=Banana bract mosaic virus OX=45661 PE=4 SV=1
2::A0A0G2R267 **Mass:** 52730 **Score:** 44 **Matches:** 1(1) **Sequences:** 1(1)
>tr|A0A0G2R267|A0A0G2R267_9POTV Helper component protein (Fragment) OS=Banana bract mosaic virus OX=45661 PE=4 SV=1
2::A0A0G2R272 **Mass:** 52730 **Score:** 44 **Matches:** 1(1) **Sequences:** 1(1)
>tr|A0A0G2R272|A0A0G2R272_9POTV Helper component protein (Fragment) OS=Banana bract mosaic virus OX=45661 PE=4 SV=1
2::A0A0G2R273 **Mass:** 52722 **Score:** 44 **Matches:** 1(1) **Sequences:** 1(1)
>tr|A0A0G2R273|A0A0G2R273_9POTV Helper component protein (Fragment) OS=Banana bract mosaic virus OX=45661 PE=4 SV=1
2::A0A0G2R287 **Mass:** 52710 **Score:** 44 **Matches:** 1(1) **Sequences:** 1(1)

>tr|A0A0G2R287|A0A0G2R287_9POTV Helper component protein (Fragment) OS=Banana bract mosaic virus OX=45661 PE=4 SV=1
2::A0A0G2R288 **Mass:** 52752 **Score:** 44 **Matches:** 1(1) **Sequences:** 1(1)
>tr|A0A0G2R288|A0A0G2R288_9POTV Helper component protein (Fragment) OS=Banana bract mosaic virus OX=45661 PE=4 SV=1
2::A0A0G2R2A5 **Mass:** 52696 **Score:** 44 **Matches:** 1(1) **Sequences:** 1(1)
>tr|A0A0G2R2A5|A0A0G2R2A5_9POTV Helper component protein (Fragment) OS=Banana bract mosaic virus OX=45661 PE=4 SV=1
2::A0A0G2R2A6 **Mass:** 52830 **Score:** 44 **Matches:** 1(1) **Sequences:** 1(1)
>tr|A0A0G2R2A6|A0A0G2R2A6_9POTV Helper component protein (Fragment) OS=Banana bract mosaic virus OX=45661 PE=4 SV=1
2::A0A0G2R2E7 **Mass:** 52648 **Score:** 44 **Matches:** 1(1) **Sequences:** 1(1)
>tr|A0A0G2R2E7|A0A0G2R2E7_9POTV Helper component protein (Fragment) OS=Banana bract mosaic virus OX=45661 PE=4 SV=1
2::A0A0G2R2F0 **Mass:** 52660 **Score:** 44 **Matches:** 1(1) **Sequences:** 1(1)
>tr|A0A0G2R2F0|A0A0G2R2F0_9POTV Helper component protein (Fragment) OS=Banana bract mosaic virus OX=45661 PE=4 SV=1
2::A0A166EG72 **Mass:** 357784 **Score:** 44 **Matches:** 1(1) **Sequences:** 1(1)
>tr|A0A166EG72|A0A166EG72_9POTV Polyprotein OS=Banana bract mosaic virus OX=45661 PE=3 SV=1
2::A0A1X9I7T3 **Mass:** 17491 **Score:** 44 **Matches:** 1(1) **Sequences:** 1(1)
>tr|A0A1X9I7T3|A0A1X9I7T3_9POTV Helper component protein (Fragment) OS=Banana bract mosaic virus OX=45661 PE=4 SV=1
2::A0A343U5W8 **Mass:** 357746 **Score:** 44 **Matches:** 1(1) **Sequences:** 1(1)
>tr|A0A343U5W8|A0A343U5W8_9POTV Polyprotein OS=Banana bract mosaic virus OX=45661 PE=3 SV=1
2::A6XMW6 **Mass:** 357726 **Score:** 44 **Matches:** 1(1) **Sequences:** 1(1)
>tr|A6XMW6|A6XMW6_9POTV Polyprotein OS=Banana bract mosaic virus OX=45661 PE=3 SV=1
2::G9B189 **Mass:** 357800 **Score:** 44 **Matches:** 1(1) **Sequences:** 1(1)
>tr|G9B189|G9B189_9POTV Polyprotein OS=Banana bract mosaic virus OX=45661 PE=3 SV=1

---

45. 2::A0A385IRN7 **Mass:** 95529 **Score:** 44 **Matches:** 1(1) **Sequences:** 1(1) **emPAI:** 0.05
>tr|A0A385IRN7|A0A385IRN7_9CAUD Putative colanic acid-degrading protein OS=Escherichia phage vB_EcoM_IME392 OX=2301649 PE=4 SV=1

Query	Observed	Mr(expt)	Mr(calc)	ppm	Miss	Score	Expect	Rank	Unique	Peptide
77	374.2282	746.4419	746.4439	-2.74	0	44	0.0076	1	U	R.LFVGGVR.L

---

46. 2::A0A2D2W767 **Mass:** 12740 **Score:** 44 **Matches:** 1(1) **Sequences:** 1(1) **emPAI:** 0.38
>tr|A0A2D2W767|A0A2D2W767_9CAUD Uncharacterized protein OS=Pectobacterium phage DU_PP_V OX=2041492 GN=P13BB106kb_p046 PE=4 SV=1

Query	Observed	Mr(expt)	Mr(calc)	ppm	Miss	Score	Expect	Rank	Unique	Peptide
223	414.7514	827.4882	827.4865	2.15	0	44	0.041	1	U	K.IANLELR.A

---

47. 2::A0A1W6QMP8 **Mass:** 88440 **Score:** 44 **Matches:** 1(1) **Sequences:** 1(1) **emPAI:** 0.05
>tr|A0A1W6QMP8|A0A1W6QMP8_9INFB Polymerase basic protein 2 OS=Influenza B virus (B/Minnesota/05/2017) OX=1985038 GN=PB2 PE=3 SV=1

Query	Observed	Mr(expt)	Mr(calc)	ppm	Miss	Score	Expect	Rank	Unique	Peptide
703	517.7610	1033.5075	1033.5080	-0.48	1	44	0.013	1	U	K.KDPDLGDFK.T

Proteins matching the same set of peptides:
2::A0A482CPQ1 **Mass:** 88454 **Score:** 44 **Matches:** 1(1) **Sequences:** 1(1)
>tr|A0A482CPQ1|A0A482CPQ1_9INFB Polymerase basic protein 2 OS=Influenza B virus OX=11520 GN=PB2 PE=3 SV=1

---

48. 2::A0A0F6R7E5 **Mass:** 12860 **Score:** 43 **Matches:** 1(1) **Sequences:** 1(1) **emPAI:** 0.38
>tr|A0A0F6R7E5|A0A0F6R7E5_9CAUD Uncharacterized protein OS=Escherichia coli O157 typing phage 8 OX=1508683 GN=ECTP8_01430 PE=4 SV=1

Query	Observed	Mr(expt)	Mr(calc)	ppm	Miss	Score	Expect	Rank	Unique	Peptide
145	393.7576	785.5007	785.5011	-0.49	0	43	0.0097	1	U	K.IAAAVTIK.V

Proteins matching the same set of peptides:
2::A0A0F6TJL0 **Mass:** 12860 **Score:** 43 **Matches:** 1(1) **Sequences:** 1(1)
>tr|A0A0F6TJL0|A0A0F6TJL0_9CAUD Uncharacterized protein OS=Escherichia coli O157 typing phage 12 OX=1508674 GN=ECTP12_01880 PE=4 SV=1
2::A0A0F6TK27 **Mass:** 12860 **Score:** 43 **Matches:** 1(1) **Sequences:** 1(1)
>tr|A0A0F6TK27|A0A0F6TK27_9CAUD Uncharacterized protein OS=Escherichia coli O157 typing phage 11 OX=1508673 GN=ECTP11_01820 PE=4 SV=1
2::C5H7R7 **Mass:** 12860 **Score:** 43 **Matches:** 1(1) **Sequences:** 1(1)
>tr|C5H7R7|C5H7R7_9CAUD Uncharacterized protein 137 OS=Escherichia phage wV8 OX=576791 GN=137 PE=4 SV=1

---

49. 2::A0A0N6YRF3 **Mass:** 8469 **Score:** 43 **Matches:** 1(1) **Sequences:** 1(1) **emPAI:** 0.62
>tr|A0A0N6YRF3|A0A0N6YRF3_9CAUD Uncharacterized protein OS=Dickeya phage phiD3 OX=1542131 GN=HQ80_0150 PE=4 SV=1

Query	Observed	Mr(expt)	Mr(calc)	ppm	Miss	Score	Expect	Rank	Unique	Peptide
179	404.2215	806.4285	806.4286	-0.22	0	43	0.031	1	U	K.YILAGDR.S

Proteins matching the same set of peptides:
2::A0A140XB41 **Mass:** 8469 **Score:** 43 **Matches:** 1(1) **Sequences:** 1(1)
>tr|A0A140XB41|A0A140XB41_9CAUD Uncharacterized protein OS=Dickeya phage phiDP10.3 OX=1542132 GN=HQ82_0205 PE=4 SV=1
2::A0A140XBH8 **Mass:** 8469 **Score:** 43 **Matches:** 1(1) **Sequences:** 1(1)
>tr|A0A140XBH8|A0A140XBH8_9CAUD Uncharacterized protein OS=Dickeya phage phiDP23.1 OX=1542133 GN=HQ81_0129 PE=4 SV=1
2::A0A248H1V8 **Mass:** 8469 **Score:** 43 **Matches:** 1(1) **Sequences:** 1(1)
>tr|A0A248H1V8|A0A248H1V8_9CAUD Uncharacterized protein OS=Dickeya phage JA15 OX=1983655 PE=4 SV=1
2::A0A2H4PHZ7 **Mass:** 8469 **Score:** 43 **Matches:** 1(1) **Sequences:** 1(1)
>tr|A0A2H4PHZ7|A0A2H4PHZ7_9CAUD Uncharacterized protein OS=Dickeya phage PP35 OX=2053675 PE=4 SV=1
2::A0A3G2K9U6 **Mass:** 8469 **Score:** 43 **Matches:** 1(1) **Sequences:** 1(1)
>tr|A0A3G2K9U6|A0A3G2K9U6_9CAUD Uncharacterized protein OS=Dickeya phage Kamild OX=2320190 PE=4 SV=1
2::I0J327 **Mass:** 8469 **Score:** 43 **Matches:** 1(1) **Sequences:** 1(1)
>tr|I0J327|I0J327_9CAUD Uncharacterized protein OS=Dickeya virus Limestone OX=1091052 PE=4 SV=1

---

50. 2::A0A1L3KJF4 **Mass:** 265678 **Score:** 42 **Matches:** 1(1) **Sequences:** 1(1) **emPAI:** 0.02
>tr|A0A1L3KJF4|A0A1L3KJF4_9VIRU RdRp OS=Beihai hepe-like virus 2 OX=1922383 PE=4 SV=1

Query	Observed	Mr(expt)	Mr(calc)	ppm	Miss	Score	Expect	Rank	Unique	Peptide
319	435.7557	869.4968	869.4971	-0.30	0	42	0.0092	1	U	K.VTIIGDPR.Q

---

51. 2::A0A345MNE4 **Mass:** 73419 **Score:** 42 **Matches:** 1(1) **Sequences:** 1(1) **emPAI:** 0.06
>tr|A0A345MNE4|A0A345MNE4_9CAUD Helicase domain protein OS=Myoviridae sp. OX=2202564 PE=4 SV=1

Query	Observed	Mr(expt)	Mr(calc)	ppm	Miss	Score	Expect	Rank	Unique	Peptide
1801	687.3982	1372.7818	1372.7814	0.33	0	42	0.0055	1	U	K.LISDVTGLTDIVK.V

---

52. 2::A0A3S9UCE2 **Mass:** 66154 **Score:** 41 **Matches:** 1(1) **Sequences:** 1(1) **emPAI:** 0.07
>tr|A0A3S9UCE2|A0A3S9UCE2_9CAUD CobT-like cobalamin biosynthesis protein OS=Gordonia phage Gray OX=2499024 GN=73 PE=4 SV=1

Query	Observed	Mr(expt)	Mr(calc)	ppm	Miss	Score	Expect	Rank	Unique	Peptide
178	404.2038	806.3930	806.3923	0.94	0	41	0.033	1	U	K.LAADFDR.V

---

53. 2::G8XUC3 **Mass:** 242144 **Score:** 41 **Matches:** 1(0) **Sequences:** 1(0)
>tr|G8XUC3|G8XUC3_9BETA Large tegument protein OS=Aotine betaherpesvirus 1 OX=50290 GN=UL48 PE=3 SV=1

Query	Observed	Mr(expt)	Mr(calc)	ppm	Miss	Score	Expect	Rank	Unique	Peptide
853	544.8016	1087.5887	1087.5873	1.23	1	41	0.073	1	U	K.VEAVSKAAADK.N

---

54. 2::A0A516LXB0 **Mass:** 188227 **Score:** 41 **Matches:** 1(1) **Sequences:** 1(1) **emPAI:** 0.02
>tr|A0A516LXB0|A0A516LXB0_9VIRU Uncharacterized protein OS=Prokaryotic dsDNA virus sp. OX=2591644 GN=Unbinned175contig1000_19 PE=4 SV=1

Query	Observed	Mr(expt)	Mr(calc)	ppm	Miss	Score	Expect	Rank	Unique	Peptide
432	464.2082	926.4018	926.4021	-0.40	0	41	0.013	1	U	K.FDPEEYK.G

---

55. 2::A0A160DHM9 **Mass:** 9089 **Score:** 41 **Matches:** 1(1) **Sequences:** 1(1) **emPAI:** 0.56
>tr|A0A160DHM9|A0A160DHM9_9CAUD Uncharacterized protein OS=Gordonia phage Jeanie OX=1838068 GN=3 PE=4 SV=1

Query	Observed	Mr(expt)	Mr(calc)	ppm	Miss	Score	Expect	Rank	Unique	Peptide
209	412.2416	822.4686	822.4712	-3.19	0	41	0.0068	1	U	R.LVADHLR.H

Proteins matching the same set of peptides:

2::A0A160DHP6 **Mass:** 9089 **Score:** 41 **Matches:** 1(1) **Sequences:** 1(1)
>tr|A0A160DHP6|A0A160DHP6_9CAUD Uncharacterized protein OS=Gordonia phage McGonagall OX=1838072 GN=3 PE=4 SV=1
2::A0A4Y6EJ81 **Mass:** 8962 **Score:** 41 **Matches:** 1(1) **Sequences:** 1(1)
>tr|A0A4Y6EJ81|A0A4Y6EJ81_9CAUD Uncharacterized protein OS=Gordonia phage Coeur OX=2571246 GN=3 PE=4 SV=1

---

56. 2::Q75491 **Mass:** 13951 **Score:** 41 **Matches:** 1(1) **Sequences:** 1(1) **emPAI:** 0.34
>tr|Q75491|Q75491_9HIV1 Envelope glycoprotein (Fragment) OS=Human immunodeficiency virus 1 OX=11676 GN=env PE=4 SV=1

Query	Observed	Mr(expt)	Mr(calc)	ppm	Miss	Score	Expect	Rank	Unique	Peptide
41	362.7083	723.4020	723.4028	-1.03	0	41	0.014	1	U	K.IADHLR.E

---

57. 2::A0A3G4ZXG6 **Mass:** 9639 **Score:** 41 **Matches:** 1(1) **Sequences:** 1(1) **emPAI:** 0.53
>tr|A0A3G4ZXG6|A0A3G4ZXG6_9VIRU Uncharacterized protein OS=Edafosvirus sp. OX=2487765 GN=Edafosvirus5_27 PE=4 SV=1

Query	Observed	Mr(expt)	Mr(calc)	ppm	Miss	Score	Expect	Rank	Unique	Peptide
78	374.2289	746.4433	746.4439	-0.85	0	41	0.016	1	U	K.VNVFLR.T

---

58. 2::A0A2H4UVE2 **Mass:** 39272 **Score:** 41 **Matches:** 1(1) **Sequences:** 1(1) **emPAI:** 0.11
>tr|A0A2H4UVE2|A0A2H4UVE2_9VIRU Serine/threonine-protein kinase OS=Bodo saltans virus OX=2024608 GN=BMW23_0847 PE=4 SV=1

Query	Observed	Mr(expt)	Mr(calc)	ppm	Miss	Score	Expect	Rank	Unique	Peptide
575	493.7976	985.5806	985.5808	-0.22	0	41	0.032	1	U	K.ITNDIVALK.T

---

59. 2::A0A1D6Y7D3 **Mass:** 87661 **Score:** 41 **Matches:** 1(1) **Sequences:** 1(1) **emPAI:** 0.05
>tr|A0A1D6Y7D3|A0A1D6Y7D3_9VIRU Putative mRNA-capping enzyme OS=Golden Marseillevirus OX=1720526 GN=GMAR_ORF208 PE=4 SV=1

Query	Observed	Mr(expt)	Mr(calc)	ppm	Miss	Score	Expect	Rank	Unique	Peptide
660	509.7616	1017.5087	1017.5131	-4.34	0	41	0.052	1	U	K.IEDLFEPR.L

---

60. 2::A0A2Z4QKJ4 **Mass:** 62494 **Score:** 40 **Matches:** 1(0) **Sequences:** 1(0)
>tr|A0A2Z4QKJ4|A0A2Z4QKJ4_9VIRU Putative RNA-dependent RNA polymerase OS=Galbut virus OX=1654579 PE=4 SV=1

Query	Observed	Mr(expt)	Mr(calc)	ppm	Miss	Score	Expect	Rank	Unique	Peptide
243	420.1995	838.3844	838.3861	-2.07	0	40	0.17	1	U	K.YWEDVK.D

Proteins matching the same set of peptides:

2::A0A2Z4QKJ8 **Mass:** 62436 **Score:** 40 **Matches:** 1(0) **Sequences:** 1(0)
>tr|A0A2Z4QKJ8|A0A2Z4QKJ8_9VIRU Putative RNA-dependent RNA polymerase OS=Galbut virus OX=1654579 PE=4 SV=1

Mascot: http://www.matrixscience.com/

MATRIX SCIENCE Mascot Search Results

User	Asia Zebrowska
Email	asia.zebrowska87@gmail.com
Search title	Copy of Zebrowska cal viruses sheep
MS data file	\\212.87.29.243\Dane\USERS\Agata\tmp\00229312zebr_BL_depol-Q2.mgf
Database 1	Sprot 2020_01 (561911 sequences; 202173710 residues)
Taxonomy 1	Viruses (16712 sequences)
Database 2	Trembl 2019_11 (179250561 sequences; 60319985647 residues)
Taxonomy 2	Viruses (4300387 sequences)
Timestamp	11 Mar 2020 at 13:22:57 GMT
Enzyme	Trypsin
Fixed modifications	Carbamidomethyl (C)
Variable modifications	Oxidation (M)
Mass values	Monoisotopic
Protein Mass	Unrestricted
Peptide Mass Tolerance	± 5 ppm
Fragment Mass Tolerance	± 0.01 Da
Max Missed Cleavages	1
Instrument type	HCD
Number of queries	5069

Protein hits :

2::A0A1U9WQS0	>tr|A0A1U9WQS0|A0A1U9WQS0_9CAUD Glycosylase OS=Geobacillus phage TP-84 OX=1965361 PE=4 SV=1
2::W6LP63	>tr|W6LP63|W6LP63_9CAUD Enolase OS=Streptococcus phage 20617 OX=1392231 GN=eno PE=3 SV=1
2::A0A076FL24	>tr|A0A076FL24|A0A076FL24_9MONO Enhanced green fluorescent protein OS=Measles morbillivirus MeV-eGFP_Edm-tag OX=1524961 GN=eGFP PE=4 SV=1
2::A0A1U9WQN1	>tr|A0A1U9WQN1|A0A1U9WQN1_9CAUD Thymidylate synthase OS=Geobacillus phage TP-84 OX=1965361 PE=4 SV=1
2::A0A514C8V0	>tr|A0A514C8V0|A0A514C8V0_9GAMR Putative gag protein (Fragment) OS=Porcine endogenous retrovirus OX=61673 PE=4 SV=1
2::A0A0Y0AS84	>tr|A0A0Y0AS84|A0A0Y0AS84_9CAUD Ribonucleoside-diphosphate reductase OS=Bacillus phage Eldridge OX=1776293 GN=Eldridge_0108 PE=3 SV=1
2::A0A060D5Q0	>tr|A0A060D5Q0|A0A060D5Q0_9CAUD Uncharacterized protein OS=Salmonella phage 9NA OX=1113547 GN=9NA_029 PE=4 SV=1
2::A0A1U9WQP5	>tr|A0A1U9WQP5|A0A1U9WQP5_9CAUD Capsid portal protein OS=Geobacillus phage TP-84 OX=1965361 PE=4 SV=1
2::A0A097DAM9	>tr|A0A097DAM9|A0A097DAM9_9BBAC Ubiquitin-like protein OS=Erinnyis ello granulovirus OX=307444 GN=v-ubq PE=4 SV=1
2::A0A345AYC4	>tr|A0A345AYC4|A0A345AYC4_9CAUD Uncharacterized protein OS=Synechococcus T7-like virus S-TIP37 OX=1332145 GN=STIP37_44 PE=4 SV=1
2::A0A068EKL1	>tr|A0A068EKL1|A0A068EKL1_9POXV Ankyrin repeat protein OS=Pigeonpox virus OX=10264 GN=fep_016 PE=4 SV=1
2::A0A0M4AGG4	>tr|A0A0M4AGG4|A0A0M4AGG4_9HIV1 Envelope glycoprotein gp160 OS=Human immunodeficiency virus 1 OX=11676 GN=env PE=3 SV=1
2::A0A0A8J9R3	>tr|A0A0A8J9R3|A0A0A8J9R3_9CAUD Uncharacterized protein OS=Klebsiella phage K64-1 OX=1439894 PE=4 SV=1
2::A0A1U9WQR1	>tr|A0A1U9WQR1|A0A1U9WQR1_9CAUD Tail protein OS=Geobacillus phage TP-84 OX=1965361 PE=4 SV=1
2::A0A345BP79	>tr|A0A345BP79|A0A345BP79_9VIRU Uncharacterized protein OS=CRESS virus sp. OX=2202563 PE=4 SV=1
2::A0A1X9Q0Z7	>tr|A0A1X9Q0Z7|A0A1X9Q0Z7_9VIRU Putative VP4 OS=Human gut gokushovirus OX=1986031 PE=4 SV=1
2::A0A0M3SUP6	>tr|A0A0M3SUP6|A0A0M3SUP6_9VIRU RNA-dependent RNA polymerase (Fragment) OS=Rhizoctonia solani beny-like virus 1 OX=1708385 PE=4 SV=1
2::D7SGS7	>tr|D7SGS7|D7SGS7_9MONO Polymerase (Fragment) OS=Paramyxovirus bat/GH47/2008 OX=589524 GN=L PE=4 SV=1
2::A0A146JG10	>tr|A0A146JG10|A0A146JG10_9VIRU Conserved putative translation elongation factor 1-alpha OS=Tokyovirus A1 OX=1826170 PE=4 SV=1
2::A0A510C2I1	>tr|A0A510C2I1|A0A510C2I1_9RHAB Putative G protein OS=Red clover associated cytorhabdovirus 1 OX=2448906 PE=4 SV=1
2::A0A482JV29	>tr|A0A482JV29|A0A482JV29_9RHAB RNA-directed RNA polymerase L OS=Lampyris noctiluca rhabdo-like virus 1 OX=2552994 PE=3 SV=1
2::A0A089FY51	>tr|A0A089FY51|A0A089FY51_9NIDO Putative 1b protein OS=Mikumi yellow baboon virus 1 OX=1546177 GN=ORF1ab PE=4 SV=1
2::A0A0M5M5R9	>tr|A0A0M5M5R9|A0A0M5M5R9_9CAUD SsDNA-binding protein OS=Citrobacter phage Margaery OX=1701810 GN=CPT_Margaery222 PE=4 SV=1
2::A0A1L2CVK2	>tr|A0A1L2CVK2|A0A1L2CVK2_9CAUD Uncharacterized protein OS=Pectobacterium phage CBB OX=1792242 GN=CBB_463 PE=4 SV=1
2::A0A516L0H2	>tr|A0A516L0H2|A0A516L0H2_9VIRU Uncharacterized protein OS=Prokaryotic dsDNA virus sp. OX=2591644 GN=GOVbin2950_25 PE=4 SV=1
2::A0A3Q8J4E7	>tr|A0A3Q8J4E7|A0A3Q8J4E7_9GAMA Glycoprotein B OS=Vombatid gammaherpesvirus 1 OX=2052651 GN=ORF8 PE=4 SV=1
2::A0A097BY06	>tr|A0A097BY06|A0A097BY06_9CAUD Minor tail protein OS=Mycobacterium phage Power OX=1552733 GN=PBI_POWER_31 PE=4 SV=1
2::A0A5J6T603	>tr|A0A5J6T603|A0A5J6T603_9CAUD Tape measure protein OS=Bacillus phage 280BB001 OX=2601665 GN=29 PE=4 SV=1
2::A0A023JAL3	>tr|A0A023JAL3|A0A023JAL3_9HIV1 Envelope glycoprotein gp160 (Fragment) OS=Human immunodeficiency virus 1 OX=11676 GN=env PE=3 SV=1
2::A0A096XT44	>tr|A0A096XT44|A0A096XT44_9CAUD Uncharacterized protein OS=Enterococcus phage ECP3 OX=1498168 PE=4 SV=1
2::A0A516LXB0	>tr|A0A516LXB0|A0A516LXB0_9VIRU Uncharacterized protein OS=Prokaryotic dsDNA virus sp. OX=2591644 GN=Unbinned175contig1000_19 PE=4 SV=1
2::Q6RU22	>tr|Q6RU22|Q6RU22_9HIV1 Gag protein (Fragment) OS=Human immunodeficiency virus 1 OX=11676 GN=gag PE=4 SV=1
2::S4VSF6	>tr|S4VSF6|S4VSF6_9VIRU Uncharacterized protein OS=Pandoravirus dulcis OX=1349409 GN=pdul_cds_330 PE=4 SV=1
2::O90019	>tr|O90019|O90019_9HIV1 Envelope glycoprotein (Fragment) OS=Human immunodeficiency virus 1 OX=11676 GN=env PE=4 SV=1
2::A0A0G2Y0A6	>tr|A0A0G2Y0A6|A0A0G2Y0A6_MIMIV Heat shock protein 70-like protein OS=Acanthamoeba polyphaga mimivirus OX=212035 PE=4 SV=1
2::A0A3S7SGD4	>tr|A0A3S7SGD4|A0A3S7SGD4_9CAUD Uncharacterized protein OS=Aerococcus phage vB_AviM_AVP OX=2495576 GN=AVP_16 PE=4 SV=1
2::A0A4Y5FP44	>tr|A0A4Y5FP44|A0A4Y5FP44_TSWV RNA dependent RNA polymerase OS=Tomato spotted wilt virus OX=1933298 GN=RdRp PE=4 SV=1
2::A0A345MNF6	>tr|A0A345MNF6|A0A345MNF6_9CAUD Isoleucyl-tRNA synthetase OS=Myoviridae sp. OX=2202564 PE=4 SV=1
2::A0A1U9WQR2	>tr|A0A1U9WQR2|A0A1U9WQR2_9CAUD Uncharacterized protein OS=Geobacillus phage TP-84 OX=1965361 PE=4 SV=1
2::A0A3G5A6F4	>tr|A0A3G5A6F4|A0A3G5A6F4_9VIRU ANK_REP_REGION domain-containing protein OS=Hyperionvirus sp. OX=2487770 GN=Hyperionvirus2_195 PE=4 SV=1
2::A0A1V0DZB4	>tr|A0A1V0DZB4|A0A1V0DZB4_9CAUD Replicative DNA helicase OS=Acinetobacter phage WCHABP12 OX=1965454 GN=dnaB PE=4 SV=1
2::A0A160DHM9	>tr|A0A160DHM9|A0A160DHM9_9CAUD Uncharacterized protein OS=Gordonia phage Jeanie OX=1838068 GN=3 PE=4 SV=1
2::Q6R4V1	>tr|Q6R4V1|Q6R4V1_9CAUD Putative replication protein RepA OS=Vibrio virus VP882 OX=261726 PE=4 SV=1
2::A0A076JU09	>tr|A0A076JU09|A0A076JU09_9ALPH Portal protein OS=Equid alphaherpesvirus 1 OX=10326 PE=3 SV=1
2::A0A1L7DSB0	>tr|A0A1L7DSB0|A0A1L7DSB0_9CAUD Tail tubular B OS=Ralstonia virus phiAp1 OX=1932865 GN=phiAp1_42 PE=4 SV=1
2::A0A1S5RFA4	>tr|A0A1S5RFA4|A0A1S5RFA4_9CAUD Putative terminase OS=Helicobacter phage SwA626G OX=1852684 PE=4 SV=1
2::A0A514D6G7	>tr|A0A514D6G7|A0A514D6G7_9VIRU Uncharacterized protein OS=Leviviridae sp. OX=2027243 GN=H2RhizoLitter493661_000002 PE=4 SV=1
2::Q75491	>tr|Q75491|Q75491_9HIV1 Envelope glycoprotein (Fragment) OS=Human immunodeficiency virus 1 OX=11676 GN=env PE=4 SV=1
2::A0A345MUY6	>tr|A0A345MUY6|A0A345MUY6_9VIRU Putative replication initiation protein OS=Microviridae sp. OX=2202644 PE=4 SV=1
2::A0A059UHZ7	>tr|A0A059UHZ7|A0A059UHZ7_9RETR Pol protein (Fragment) OS=Feline immunodeficiency virus OX=11673 GN=pol PE=3 SV=1
2::A0A345MNE4	>tr|A0A345MNE4|A0A345MNE4_9CAUD Helicase domain protein OS=Myoviridae sp. OX=2202564 PE=4 SV=1
2::A0A075W7P3	>tr|A0A075W7P3|A0A075W7P3_9ENTO VP1 (Fragment) OS=Enterovirus B93 OX=991902 PE=4 SV=1
2::A0A0S3QM10	>tr|A0A0S3QM10|A0A0S3QM10_9HIV1 Protein Nef OS=Human immunodeficiency virus 1 OX=11676 GN=nef PE=3 SV=1
2::A0A3G5A8B3	>tr|A0A3G5A8B3|A0A3G5A8B3_9VIRU FkbM family methyltransferase OS=Hyperionvirus sp. OX=2487770 GN=Hyperionvirus7_78 PE=4 SV=1
2::A0A059T7Q4	>tr|A0A059T7Q4|A0A059T7Q4_9CAUD Tape measure protein OS=Staphylococcus phage DW2 OX=1464127 GN=DW2_52 PE=4 SV=1
2::A0A2D2W767	>tr|A0A2D2W767|A0A2D2W767_9CAUD Uncharacterized protein OS=Pectobacterium phage DU_PP_V OX=2041492 GN=P13BB106kb_p046 PE=4 SV=1
2::A0A1W5J0S3	>tr|A0A1W5J0S3|A0A1W5J0S3_9MONO RNA-dependent RNA polymerase (Fragment) OS=Paramyxovirus PREDICT_PMV-18 OX=1647218 PE=4 SV=1
2::A0A0D3MVZ4	>tr|A0A0D3MVZ4|A0A0D3MVZ4_9ADEN Ig-like domain-containing protein OS=Duck adenovirus 1 OX=130329 PE=4 SV=1
2::A0A0G2XZL2	>tr|A0A0G2XZL2|A0A0G2XZL2_MIMIV Putative sel1-like repeat-containing protein OS=Acanthamoeba polyphaga mimivirus OX=212035 PE=4 SV=1
1::C1JCT2	Polyprotein-FSD OS=Solenopsis invicta virus 3 OX=631345 PE=1 SV=2
2::A0A1X9IA00	>tr|A0A1X9IA00|A0A1X9IA00_9CAUD Uncharacterized protein OS=Staphylococcus phage vB_SscM-1 OX=1868844 GN=vB_SscM-1_182 PE=4 SV=1
2::A0A0K0KVG1	>tr|A0A0K0KVG1|A0A0K0KVG1_9CAUD Putative neck protein OS=Prochlorococcus phage P-TIM68 OX=1542477 PE=4 SV=1
2::A0A2H4UVE2	>tr|A0A2H4UVE2|A0A2H4UVE2_9VIRU Serine/threonine-protein kinase OS=Bodo saltans virus OX=2024608 GN=BMW23_0847 PE=4 SV=1
2::A0A1V0SKH4	>tr|A0A1V0SKH4|A0A1V0SKH4_9VIRU AAA family ATPase OS=Klosneuvirus KNV1 OX=1977640 GN=Klosneuvirus_4_37 PE=4 SV=1
2::A0A023VZE8	>tr|A0A023VZE8|A0A023VZE8_BSMV Beta-B protein OS=Barley stripe mosaic virus OX=12327 PE=4 SV=1
2::A0A1S5NQC2	>tr|A0A1S5NQC2|A0A1S5NQC2_9VIRU Glycoprotein (Fragment) OS=Chim orthonairovirus OX=2170062 PE=4 SV=1
2::A0A023NH97	>tr|A0A023NH97|A0A023NH97_9CAUD Putative ribonucleoside-diphosphate redutase OS=Dinoroseobacter phage DFL12phi1 OX=1477404 GN=DFL12P1_0034 PE=4 SV=1

	Target	Decoy	False discovery rate
Peptide matches above identity threshold	162	97	59.88 %
Peptide matches above homology or identity threshold	245	160	65.31 %

Select Summary Report

Format As: Select Summary (protein hits) — Help — Help

Significance threshold p< 0.05 — Max. number of hits AUTO — Show Percolator scores ☐

Standard scoring ○ MudPIT scoring ◉ — Ions score or expect cut-off 40 — Show sub-sets 0

Show pop-ups ○ Suppress pop-ups ◉ — Require bold red ☑

Preferred taxonomy: All entries

Re-Search — ◉ All queries ○ Unassigned ○ Below homology threshold ○ Below identity threshold

1. 2::A0A1U9WQS0 **Mass:** 112893 **Score:** 2271 **Matches:** 36(35) **Sequences:** 23(22) **emPAI:** 1.37

>tr|A0A1U9WQS0|A0A1U9WQS0_9CAUD Glycosylase OS=Geobacillus phage TP-84 OX=1965361 PE=4 SV=1

Query	Observed	Mr(expt)	Mr(calc)	ppm	Miss	Score	Expect	Rank	Unique	Peptide
233	423.2293	844.4440	844.4443	-0.38	0	41	0.084	1	U	R.NVAEWVK.Q
299	438.7429	875.4713	875.4712	0.05	0	78	7e-06	1	U	K.LSIGTTER.W 298
369	455.7293	909.4440	909.4444	-0.37	0	52	0.0012	1	U	K.LFAESTDK.N
549	494.2976	986.5807	986.5801	0.63	0	61	0.0013	1	U	R.VPYVPTAIK.D
601	501.3043	1000.5940	1000.5917	2.28	0	98	2.9e-08	1	U	K.ISGLAGVTGVK.G 600
842	539.8016	1077.5887	1077.5859	2.62	0	87	1.3e-06	1	U	K.LIDAGLWYK.Q 841
1095	582.3086	1162.6026	1162.6022	0.35	0	60	0.00043	1	U	R.TLWPNYIEK.D 1096
1134	587.2643	1172.5140	1172.5132	0.64	0	82	4.2e-07	1	U	R.YDSMGQTTVR.V
1163	590.8094	1179.6042	1179.6023	1.61	0	87	6e-07	1	U	R.ADYDELITLK.V
1436	415.8831	1244.6273	1244.6295	-1.76	0	45	0.014	1	U	K.MGEISRPGINR.D
1620	649.8357	1297.6568	1297.6554	1.06	0	104	1.6e-08	1	U	R.FSVIIDGQEYK.M 1619
1737	663.8226	1325.6306	1325.6286	1.54	0	77	6.5e-06	1	U	K.TTNISCEVQFK.V
1764	668.8596	1335.7046	1335.7034	0.93	1	61	0.00031	1	U	R.RADYDELITLK.V
2549	808.3806	1614.7466	1614.7460	0.34	0	114	6.5e-09	1	U	K.CTIDNVYLGGAGYGR.R 2550

2551	808.4378	1614.8611	1614.8617	-0.35	1	98	5.9e-08	1	U	R.IKNEVIIDANPDFK.L
2723	847.8987	1693.7829	1693.7835	-0.35	0	138	4.5e-12	1	U	R.VSEYNGIEYATAYSK.T
2985	597.6327	1789.8762	1789.8748	0.80	0	(83)	1.4e-06	1	U	K.ITSIGFHEFNVDAQGR.I
2987	895.9465	1789.8784	1789.8748	2.05	0	133	1e-11	1	U	K.ITSIGFHEFNVDAQGR.I 2986
3087	917.4698	1832.9251	1832.9268	-0.91	0	167	8.2e-15	1	U	R.LLDSQTAQDNFINNLK.T 3088
3089	611.9832	1832.9278	1832.9268	0.53	0	(86)	9.9e-07	1	U	R.LLDSQTAQDNFINNLK.T
3173	626.9589	1877.8549	1877.8519	1.61	0	(64)	7.2e-05	1	U	R.INAHAMWGDGIPDYYR.F
3214	632.2909	1893.8508	1893.8468	2.13	0	75	7.3e-06	1	U	R.INAHAMWGDGIPDYYR.F
3310	967.4451	1932.8756	1932.8755	0.04	0	85	4.3e-07	1	U	R.WPIHSDDNWGSTVTYR.D
3311	645.2996	1932.8770	1932.8755	0.77	0	(54)	0.0015	1	U	R.WPIHSDDNWGSTVTYR.D
3651	707.0097	2118.0074	2118.0058	0.73	0	50	0.005	1	U	R.IFETLPDVVPWHPDPNEN.-
4251	1251.0846	2500.1547	2500.1546	0.00	0	127	3.4e-11	1	U	R.QYLNDEFVQQYGLEATTYYR.L
4252	834.3926	2500.1561	2500.1546	0.57	0	(77)	3.2e-06	1	U	R.QYLNDEFVQQYGLEATTYYR.L
4734	1082.5377	3244.5914	3244.5962	-1.49	0	100	5.1e-08	1	U	K.ITGYACQLSEQPETVLTYTLNVPQAGTYK.I

---

2. 2::W6LP63 **Mass:** 47022 **Score:** 679 **Matches:** 6(6) **Sequences:** 2(2) **emPAI:** 0.31

>tr|W6LP63|W6LP63_9CAUD Enolase OS=Streptococcus phage 20617 OX=1392231 GN=eno PE=3 SV=1

Query	Observed	Mr(expt)	Mr(calc)	ppm	Miss	Score	Expect	Rank	Unique	Peptide
3419	990.9996	1979.9846	1979.9874	-1.41	0	136	6.7e-12	1	U	K.VNQIGTLTETFDAIEMAK.E
3454	998.9968	1995.9790	1995.9823	-1.64	0	(108)	5.2e-09	1	U	K.VNQIGTLTETFDAIEMAK.E 3453
3762	731.0292	2190.0658	2190.0652	0.30	0	(141)	1e-11	1	U	R.SGETEDSTIADIAVATNAGQIK.T
3763	1096.0406	2190.0667	2190.0652	0.70	0	158	2e-13	1	U	R.SGETEDSTIADIAVATNAGQIK.T 3761

---

3. 2::A0A076FL24 **Mass:** 27039 **Score:** 594 **Matches:** 11(11) **Sequences:** 6(6) **emPAI:** 1.95

>tr|A0A076FL24|A0A076FL24_9MONO Enhanced green fluorescent protein OS=Measles morbillivirus MeV-eGFP_Edm-tag OX=1524961 GN=eGFP PE=4 SV=1

Query	Observed	Mr(expt)	Mr(calc)	ppm	Miss	Score	Expect	Rank	Unique	Peptide
1526	633.7923	1265.5700	1265.5710	-0.78	0	(55)	0.00056	1	U	K.SAMPEGYVQER.T
1558	641.7905	1281.5665	1281.5659	0.45	0	86	3.7e-07	1	U	K.SAMPEGYVQER.T 1559
1802	674.3319	1346.6493	1346.6507	-0.99	1	70	2.5e-05	1	U	R.TIFFKDDGNYK.T
2381	514.9356	1541.7849	1541.7838	0.74	1	66	0.0001	1	U	K.GIDFKEDGNILGHK.L
4199	813.0926	2436.2559	2436.2537	0.91	0	87	3.1e-07	1	U	K.GEELFTGVVPILVELDGDVNGHK.F
4421	917.8240	2750.4501	2750.4491	0.36	1	(62)	0.00011	1	U	M.VSKGEELFTGVVPILVELDGDVNGHK.F
4422	688.6199	2750.4505	2750.4491	0.53	1	65	5.6e-05	1	U	M.VSKGEELFTGVVPILVELDGDVNGHK.F
4474	966.8354	2897.4842	2897.4845	-0.08	1	109	2.7e-09	1	U	-.MVSKGEELFTGVVPILVELDGDVNGHK.F
4476	725.3788	2897.4861	2897.4845	0.57	1	(74)	8.9e-06	1	U	-.MVSKGEELFTGVVPILVELDGDVNGHK.F 4475

Proteins matching the same set of peptides:

2::A0A076V611 **Mass:** 27039 **Score:** 594 **Matches:** 11(11) **Sequences:** 6(6)
>tr|A0A076V611|A0A076V611_9VIRU EGFP OS=synthetic recombinant Chikungunya virus strain LS3-GFP OX=1529938 PE=4 SV=1
2::A0A0U3A3D8 **Mass:** 27039 **Score:** 594 **Matches:** 11(11) **Sequences:** 6(6)
>tr|A0A0U3A3D8|A0A0U3A3D8_9MONO EGFP OS=Zaire ebolavirus OX=186538 PE=4 SV=1
2::A0A1V0D974 **Mass:** 27010 **Score:** 594 **Matches:** 11(11) **Sequences:** 6(6)
>tr|A0A1V0D974|A0A1V0D974_9POXV EGFP OS=Vaccinia virus OX=10245 GN=EGFP PE=4 SV=1
2::A0A348GST9 **Mass:** 27039 **Score:** 594 **Matches:** 11(11) **Sequences:** 6(6)
>tr|A0A348GST9|A0A348GST9_9MONO Enhanced green fluorescent protein OS=Measles morbillivirus OX=11234 GN=EGFP PE=4 SV=1
2::A0A3Q8WKS4 **Mass:** 27039 **Score:** 594 **Matches:** 11(11) **Sequences:** 6(6)
>tr|A0A3Q8WKS4|A0A3Q8WKS4_9VIRU Enhanced green fluorescence protein OS=Sacbrood virus OX=89463 PE=4 SV=1
2::A0A5J6XWW3 **Mass:** 27089 **Score:** 594 **Matches:** 11(11) **Sequences:** 6(6)
>tr|A0A5J6XWW3|A0A5J6XWW3_9GAMA YFP OS=Macacine gammaherpesvirus 5 OX=154334 PE=4 SV=1
2::C5MKY7 **Mass:** 27039 **Score:** 594 **Matches:** 11(11) **Sequences:** 6(6)
>tr|C5MKY7|C5MKY7_HCMV Enhanced green fluorescent protein OS=Human cytomegalovirus OX=10359 GN=egfp PE=4 SV=1

---

4. 2::A0A1U9WQN1 **Mass:** 38540 **Score:** 281 **Matches:** 5(5) **Sequences:** 3(3) **emPAI:** 0.55

>tr|A0A1U9WQN1|A0A1U9WQN1_9CAUD Thymidylate synthase OS=Geobacillus phage TP-84 OX=1965361 PE=4 SV=1

Query	Observed	Mr(expt)	Mr(calc)	ppm	Miss	Score	Expect	Rank	Unique	Peptide
1005	568.8060	1135.5974	1135.5985	-1.05	1	56	0.00094	1	U	K.KGEISGNYIR.D 1004
1766	669.8406	1337.6666	1337.6649	1.29	0	86	2.3e-06	1	U	K.VAGYEEAIMSLR.M
1816	677.8396	1353.6646	1353.6598	3.56	0	(82)	6.4e-06	1	U	K.VAGYEEAIMSLR.M
3323	968.9863	1935.9581	1935.9578	0.14	0	129	4.7e-11	1	U	R.LANIQETVLSEWYQDK.I

---

5. 2::A0A514C8V0 **Mass:** 13178 **Score:** 257 **Matches:** 4(4) **Sequences:** 2(2) **emPAI:** 1.55

>tr|A0A514C8V0|A0A514C8V0_9GAMR Putative gag protein (Fragment) OS=Porcine endogenous retrovirus OX=61673 PE=4 SV=1

Query	Observed	Mr(expt)	Mr(calc)	ppm	Miss	Score	Expect	Rank	Unique	Peptide
1125	391.1940	1170.5602	1170.5638	-3.06	0	(51)	0.0025	1	U	R.HQGVMVGMGQK.D
1126	586.2894	1170.5642	1170.5638	0.35	0	(89)	3.2e-07	1	U	R.HQGVMVGMGQK.D
1200	594.2859	1186.5572	1186.5587	-1.30	0	92	1.1e-07	1	U	R.HQGVMVGMGQK.D
1250	599.7659	1197.5173	1197.5150	1.92	0	99	5.2e-07	1	U	K.DSYVGDEAQSK.R

Proteins matching the same set of peptides:

2::A0A514C8Y6 **Mass:** 12725 **Score:** 257 **Matches:** 4(4) **Sequences:** 2(2)
>tr|A0A514C8Y6|A0A514C8Y6_9GAMR Putative gag protein (Fragment) OS=Porcine endogenous retrovirus OX=61673 PE=4 SV=1
1::P00544 **Mass:** 62047 **Score:** 257 **Matches:** 4(4) **Sequences:** 2(2)
Tyrosine-protein kinase transforming protein Fgr OS=Feline sarcoma virus (strain Gardner-Rasheed) OX=11775 GN=V-FGR PE=3 SV=1

---

6. 2::A0A0Y0AS84 **Mass:** 86377 **Score:** 117 **Matches:** 1(1) **Sequences:** 1(1) **emPAI:** 0.05

>tr|A0A0Y0AS84|A0A0Y0AS84_9CAUD Ribonucleoside-diphosphate reductase OS=Bacillus phage Eldridge OX=1776293 GN=Eldridge_0108 PE=3 SV=1

Query	Observed	Mr(expt)	Mr(calc)	ppm	Miss	Score	Expect	Rank	Unique	Peptide
2622	821.9284	1641.8422	1641.8434	-0.75	0	117	8.4e-10	1	U	K.QLNNTAVSVDQLGQR.Q

Proteins matching the same set of peptides:

2::A0A127AW21 **Mass:** 89526 **Score:** 117 **Matches:** 1(1) **Sequences:** 1(1)
>tr|A0A127AW21|A0A127AW21_9CAUD Ribonucleoside-diphosphate reductase OS=Bacillus phage SP-15 OX=1792032 GN=SP15_029 PE=3 SV=1
2::A0A5H2QC56 **Mass:** 86079 **Score:** 117 **Matches:** 1(1) **Sequences:** 1(1)
>tr|A0A5H2QC56|A0A5H2QC56_9VIRU Ribonucleoside-diphosphate reductase subunit alpha OS=Bacillus phage BSP7 OX=2301661 GN=BSP7_062 PE=4 SV=1

---

7. 2::A0A060D5Q0 **Mass:** 30617 **Score:** 83 **Matches:** 2(2) **Sequences:** 1(1) **emPAI:** 0.15

>tr|A0A060D5Q0|A0A060D5Q0_9CAUD Uncharacterized protein OS=Salmonella phage 9NA OX=1113547 GN=9NA_029 PE=4 SV=1

Query	Observed	Mr(expt)	Mr(calc)	ppm	Miss	Score	Expect	Rank	Unique	Peptide
5	351.7142	701.4139	701.4146	-0.91	0	(54)	6.6e-05	1	U	K.LIMTPK.A 6

---

8. 2::A0A1U9WQP5 **Mass:** 62259 **Score:** 81 **Matches:** 1(1) **Sequences:** 1(1) **emPAI:** 0.07

>tr|A0A1U9WQP5|A0A1U9WQP5_9CAUD Capsid portal protein OS=Geobacillus phage TP-84 OX=1965361 PE=4 SV=1

Query	Observed	Mr(expt)	Mr(calc)	ppm	Miss	Score	Expect	Rank	Unique	Peptide
1666	655.8282	1309.6418	1309.6401	1.26	0	81	3.7e-06	1	U	R.ELIEQESTAYK.N

---

9. 2::A0A097DAM9 **Mass:** 10827 **Score:** 76 **Matches:** 1(1) **Sequences:** 1(1) **emPAI:** 0.46

>tr|A0A097DAM9|A0A097DAM9_9BBAC Ubiquitin-like protein OS=Erinnyis ello granulovirus OX=307444 GN=v-ubq PE=4 SV=1

Query	Observed	Mr(expt)	Mr(calc)	ppm	Miss	Score	Expect	Rank	Unique	Peptide
853	541.2805	1080.5465	1080.5451	1.30	0	76	1.1e-05	1	U	R.TLSDYNIQK.E

Proteins matching the same set of peptides:
2::A0A0K0WS54 **Mass:** 10788 **Score:** 76 **Matches:** 1(1) **Sequences:** 1(1)
>tr|A0A0K0WS54|A0A0K0WS54_9BBAC Ubiquitin OS=Clostera anastomosis granulovirus B OX=1986290 GN=clas42 PE=4 SV=1
2::A0A0N9QAB0 **Mass:** 8649 **Score:** 76 **Matches:** 1(1) **Sequences:** 1(1)
>tr|A0A0N9QAB0|A0A0N9QAB0_CEV01 Ubiquitin OS=Chrysochromulina ericina virus OX=455364 GN=ceV_156 PE=4 SV=1
2::A0A0R7EYX9 **Mass:** 13787 **Score:** 76 **Matches:** 1(1) **Sequences:** 1(1)
>tr|A0A0R7EYX9|A0A0R7EYX9_9BBAC Ubiquitin-like protein OS=Diatraea saccharalis granulovirus OX=1675862 GN=v-ubq PE=4 SV=1
2::A0A1D8QL82 **Mass:** 8687 **Score:** 76 **Matches:** 1(1) **Sequences:** 1(1)
>tr|A0A1D8QL82|A0A1D8QL82_GVTN V-ubiquitin OS=Trichoplusia ni granulovirus LBIV-12 OX=1916701 PE=4 SV=1
2::A0A1S5YE59 **Mass:** 9002 **Score:** 76 **Matches:** 1(1) **Sequences:** 1(1)
>tr|A0A1S5YE59|A0A1S5YE59_9BBAC Ubiquitin OS=Mythimna unipuncta granulovirus B OX=2169746 PE=4 SV=1
2::A0A1V0QG65 **Mass:** 9615 **Score:** 76 **Matches:** 1(1) **Sequences:** 1(1)
>tr|A0A1V0QG65|A0A1V0QG65_CNPV SWPV2-ORF091 OS=Shearwaterpox virus OX=1974596 GN=SWPV2-091 PE=4 SV=1
2::A0A1V0QGT2 **Mass:** 8674 **Score:** 76 **Matches:** 1(1) **Sequences:** 1(1)
>tr|A0A1V0QGT2|A0A1V0QGT2_CNPV SWPV1-086 OS=Shearwaterpox virus OX=1974596 GN=SWPV1-086 PE=4 SV=1
2::A0A1X9VNV0 **Mass:** 25499 **Score:** 76 **Matches:** 1(1) **Sequences:** 1(1)
>tr|A0A1X9VNV0|A0A1X9VNV0_9VIRU Putative ubiquitin OS=Mimivirus AB-566-O17 OX=1988039 GN=SAGO17_0082 PE=4 SV=1
2::A0A2H4X282 **Mass:** 8716 **Score:** 76 **Matches:** 1(1) **Sequences:** 1(1)
>tr|A0A2H4X282|A0A2H4X282_9POXV Ubiquitin family protein OS=Flamingopox virus FGPVKD09 OX=2059380 GN=fgpv_071 PE=4 SV=1
2::A0A343RRG4 **Mass:** 9057 **Score:** 76 **Matches:** 1(1) **Sequences:** 1(1)
>tr|A0A343RRG4|A0A343RRG4_NPVMN Ubiquitin-protein OS=Malacosoma neustria nuclear polyhedrosis virus OX=38012 GN=ManeNPV_00026 PE=4 SV=1
2::A0A346FEF7 **Mass:** 63610 **Score:** 76 **Matches:** 1(1) **Sequences:** 1(1)
>tr|A0A346FEF7|A0A346FEF7_9POXV Breast cancer specific artificial polyepitopic immunogen OS=Vaccinia virus OX=10245 GN=11 PE=4 SV=1
2::A0A346FF48 **Mass:** 66401 **Score:** 76 **Matches:** 1(1) **Sequences:** 1(1)
>tr|A0A346FF48|A0A346FF48_9POXV Melanoma-specific artificial polyepitopic immunogen OS=Vaccinia virus OX=10245 GN=11 PE=4 SV=1
2::A0A346FGF8 **Mass:** 70547 **Score:** 76 **Matches:** 1(1) **Sequences:** 1(1)
>tr|A0A346FGF8|A0A346FGF8_9POXV Ovarian cancer specific artificial polyepitopic immunogen OS=Vaccinia virus OX=10245 GN=230 PE=4 SV=1
2::A0A3G3CB22 **Mass:** 8674 **Score:** 76 **Matches:** 1(1) **Sequences:** 1(1)
>tr|A0A3G3CB22|A0A3G3CB22_FOWPV Ubiquitin OS=Fowlpox virus OX=10261 PE=4 SV=1
2::A0A3G4ZM02 **Mass:** 8958 **Score:** 76 **Matches:** 1(1) **Sequences:** 1(1)
>tr|A0A3G4ZM02|A0A3G4ZM02_9VIRU Polyubiquitin OS=Terrestrivirus sp. OX=2487775 GN=Terrestrivirus3_146 PE=4 SV=1
2::A0A3G4ZYU7 **Mass:** 18001 **Score:** 76 **Matches:** 1(1) **Sequences:** 1(1)
>tr|A0A3G4ZYU7|A0A3G4ZYU7_9VIRU Polyubiquitin OS=Gaeavirus sp. OX=2487767 GN=Gaeavirus10_2 PE=4 SV=1
2::A0A5B8HX40 **Mass:** 8489 **Score:** 76 **Matches:** 1(1) **Sequences:** 1(1)
>tr|A0A5B8HX40|A0A5B8HX40_9VIRU Ubiquitin family OS=Mimiviridae sp. ChoanoV1 OX=2596887 GN=1_145 PE=4 SV=1
2::A0A5J6VMF0 **Mass:** 11553 **Score:** 76 **Matches:** 1(1) **Sequences:** 1(1)
>tr|A0A5J6VMF0|A0A5J6VMF0_9VIRU Ubiquitin family protein OS=Megaviridae environmental sample OX=1737588 PE=4 SV=1
2::A0PDJ3 **Mass:** 69512 **Score:** 76 **Matches:** 1(1) **Sequences:** 1(1)
>tr|A0PDJ3|A0PDJ3_BVDV Polyprotein (Fragment) OS=Bovine viral diarrhea virus OX=11099 PE=4 SV=1
2::A0PDJ4 **Mass:** 68332 **Score:** 76 **Matches:** 1(1) **Sequences:** 1(1)
>tr|A0PDJ4|A0PDJ4_BVDV Polyprotein (Fragment) OS=Bovine viral diarrhea virus OX=11099 PE=4 SV=1
2::A0PDJ5 **Mass:** 66874 **Score:** 76 **Matches:** 1(1) **Sequences:** 1(1)
>tr|A0PDJ5|A0PDJ5_BVDV Polyprotein (Fragment) OS=Bovine viral diarrhea virus OX=11099 PE=4 SV=1
2::A0PDJ6 **Mass:** 69295 **Score:** 76 **Matches:** 1(1) **Sequences:** 1(1)
>tr|A0PDJ6|A0PDJ6_BVDV Polyprotein (Fragment) OS=Bovine viral diarrhea virus OX=11099 PE=4 SV=1
2::A0PDJ7 **Mass:** 64029 **Score:** 76 **Matches:** 1(1) **Sequences:** 1(1)
>tr|A0PDJ7|A0PDJ7_BVDV Polyprotein (Fragment) OS=Bovine viral diarrhea virus OX=11099 PE=4 SV=1
2::A0PDJ8 **Mass:** 61370 **Score:** 76 **Matches:** 1(1) **Sequences:** 1(1)
>tr|A0PDJ8|A0PDJ8_BVDV Polyprotein (Fragment) OS=Bovine viral diarrhea virus OX=11099 PE=4 SV=1
2::A0PDJ9 **Mass:** 49334 **Score:** 76 **Matches:** 1(1) **Sequences:** 1(1)
>tr|A0PDJ9|A0PDJ9_BVDV Polyprotein (Fragment) OS=Bovine viral diarrhea virus OX=11099 PE=4 SV=1
2::M1HBK7 **Mass:** 11414 **Score:** 76 **Matches:** 1(1) **Sequences:** 1(1)
>tr|M1HBK7|M1HBK7_9PHYC Ubiquitin-60S ribosomal protein L40 OS=Acanthocystis turfacea Chlorella virus Br0604L OX=1278268 GN=Br0604L_251L PE=4 SV=1
2::M1HCF0 **Mass:** 8605 **Score:** 76 **Matches:** 1(1) **Sequences:** 1(1)
>tr|M1HCF0|M1HCF0_9PHYC Ubiquitin-60S ribosomal protein L40 OS=Acanthocystis turfacea Chlorella virus Can0610SP OX=1278269 GN=Can0610SP_243L PE=4 SV=1
2::M1HXG4 **Mass:** 8621 **Score:** 76 **Matches:** 1(1) **Sequences:** 1(1)
>tr|M1HXG4|M1HXG4_9PHYC Ubiquitin-60S ribosomal protein L40 OS=Acanthocystis turfacea Chlorella virus NE-JV-2 OX=1278274 GN=NE-JV-2_257L PE=4 SV=1
2::M1I0A3 **Mass:** 8605 **Score:** 76 **Matches:** 1(1) **Sequences:** 1(1)
>tr|M1I0A3|M1I0A3_9PHYC Ubiquitin-60S ribosomal protein L40 OS=Acanthocystis turfacea Chlorella virus MO0605SPH OX=1278273 GN=MO0605SPH_256L PE=4 SV=1
2::M1I328 **Mass:** 8605 **Score:** 76 **Matches:** 1(1) **Sequences:** 1(1)
>tr|M1I328|M1I328_9PHYC Ubiquitin-60S ribosomal protein L40 OS=Acanthocystis turfacea Chlorella virus NE-JV-3 OX=1278275 GN=NE-JV-3_243L PE=4 SV=1
2::M1I713 **Mass:** 8605 **Score:** 76 **Matches:** 1(1) **Sequences:** 1(1)
>tr|M1I713|M1I713_9PHYC Ubiquitin-60S ribosomal protein L40 OS=Acanthocystis turfacea Chlorella virus WI0606 OX=1278279 GN=WI0606_261L PE=4 SV=1
2::M1IA08 **Mass:** 8621 **Score:** 76 **Matches:** 1(1) **Sequences:** 1(1)
>tr|M1IA08|M1IA08_9PHYC Ubiquitin-60S ribosomal protein L40 OS=Acanthocystis turfacea Chlorella virus OR0704.3 OX=1278277 GN=OR0704.3_250L PE=4 SV=1
2::M1IAW7 **Mass:** 13598 **Score:** 76 **Matches:** 1(1) **Sequences:** 1(1)
>tr|M1IAW7|M1IAW7_9PHYC Ubiquitin-60S ribosomal protein L40 OS=Acanthocystis turfacea Chlorella virus TN603.4.2 OX=1278278 GN=TN603.4.2_251L PE=4 SV=1
2::V5LLV7 **Mass:** 9048 **Score:** 76 **Matches:** 1(1) **Sequences:** 1(1)
>tr|V5LLV7|V5LLV7_9PHYC Ubiquitin-like domain-containing protein OS=Emiliania huxleyi virus 18 OX=1421424 GN=EhV18_00108 PE=4 SV=1
2::V5LQ53 **Mass:** 9048 **Score:** 76 **Matches:** 1(1) **Sequences:** 1(1)
>tr|V5LQ53|V5LQ53_9PHYC Ubiquitin-like domain-containing protein OS=Emiliania huxleyi virus 156 OX=1421422 GN=EhV156_00104 PE=4 SV=1
2::B6S6R9 **Mass:** 8687 **Score:** 76 **Matches:** 1(1) **Sequences:** 1(1)
>tr|B6S6R9|B6S6R9_GVPU Ubiquitin OS=Pseudalatia unipuncta granulosis virus OX=36355 PE=4 SV=1
2::Q6VZQ1 **Mass:** 9615 **Score:** 76 **Matches:** 1(1) **Sequences:** 1(1)
>tr|Q6VZQ1|Q6VZQ1_CNPV CNPV096 ubiquitin OS=Canarypox virus OX=44088 GN=CNPV096 PE=4 SV=1
2::A7K8G3 **Mass:** 8605 **Score:** 76 **Matches:** 1(1) **Sequences:** 1(1)
>tr|A7K8G3|A7K8G3_9PHYC Uncharacterized protein Z203L OS=Acanthocystis turfacea chlorella virus 1 OX=322019 GN=Z203L PE=4 SV=1
2::G8DFN2 **Mass:** 9048 **Score:** 76 **Matches:** 1(1) **Sequences:** 1(1)
>tr|G8DFN2|G8DFN2_9PHYC Polyubiquitin OS=Emiliania huxleyi virus 202 OX=181211 GN=EXVG_00227 PE=4 SV=1
2::Q9WR78 **Mass:** 40006 **Score:** 76 **Matches:** 1(1) **Sequences:** 1(1)
>tr|Q9WR78|Q9WR78_9FLAV Polyprotein (Fragment) OS=Bovine viral diarrhea virus 2 OX=54315 PE=2 SV=1
2::Q9WR79 **Mass:** 29287 **Score:** 76 **Matches:** 1(1) **Sequences:** 1(1)
>tr|Q9WR79|Q9WR79_9FLAV Polyprotein (Fragment) OS=Bovine viral diarrhea virus 2 OX=54315 PE=2 SV=1
2::Q9WR80 **Mass:** 24263 **Score:** 76 **Matches:** 1(1) **Sequences:** 1(1)
>tr|Q9WR80|Q9WR80_9FLAV Polyprotein (Fragment) OS=Bovine viral diarrhea virus 2 OX=54315 PE=2 SV=1
2::Q9WR83 **Mass:** 34922 **Score:** 76 **Matches:** 1(1) **Sequences:** 1(1)
>tr|Q9WR83|Q9WR83_9FLAV Polyprotein (Fragment) OS=Bovine viral diarrhea virus 2 OX=54315 PE=2 SV=1
2::Q9WR85 **Mass:** 20043 **Score:** 76 **Matches:** 1(1) **Sequences:** 1(1)
>tr|Q9WR85|Q9WR85_9FLAV Polyprotein (Fragment) OS=Bovine viral diarrhea virus 2 OX=54315 PE=2 SV=1
2::Q9WR87 **Mass:** 38917 **Score:** 76 **Matches:** 1(1) **Sequences:** 1(1)
>tr|Q9WR87|Q9WR87_9FLAV Polyprotein (Fragment) OS=Bovine viral diarrhea virus 2 OX=54315 PE=2 SV=1
2::Q9YWK8 **Mass:** 113132 **Score:** 76 **Matches:** 1(1) **Sequences:** 1(1)
>tr|Q9YWK8|Q9YWK8_9FLAV Genome polyprotein (Fragment) OS=Bovine viral diarrhea virus strain Rit 4350 OX=83397 PE=4 SV=1
2::Q76B24 **Mass:** 270818 **Score:** 76 **Matches:** 1(1) **Sequences:** 1(1)
>tr|Q76B24|Q76B24_9FLAV Genome polyprotein (Fragment) OS=Bovine viral diarrhea virus T-20 OX=233889 PE=4 SV=1
2::Q76B25 **Mass:** 337981 **Score:** 76 **Matches:** 1(1) **Sequences:** 1(1)
>tr|Q76B25|Q76B25_9FLAV Genome polyprotein (Fragment) OS=Bovine viral diarrhea virus 190cp OX=233888 PE=4 SV=1
2::Q91N41 **Mass:** 39506 **Score:** 76 **Matches:** 1(1) **Sequences:** 1(1)
>tr|Q91N41|Q91N41_BVDV Polyprotein (Fragment) OS=Bovine viral diarrhea virus OX=11099 PE=4 SV=1
2::Q91N42 **Mass:** 40582 **Score:** 76 **Matches:** 1(1) **Sequences:** 1(1)
>tr|Q91N42|Q91N42_BVDV Polyprotein (Fragment) OS=Bovine viral diarrhea virus OX=11099 PE=4 SV=1
2::Q91N43 **Mass:** 63503 **Score:** 76 **Matches:** 1(1) **Sequences:** 1(1)
>tr|Q91N43|Q91N43_BVDV Polyprotein (Fragment) OS=Bovine viral diarrhea virus OX=11099 PE=4 SV=1
2::Q91N44 **Mass:** 63235 **Score:** 76 **Matches:** 1(1) **Sequences:** 1(1)
>tr|Q91N44|Q91N44_BVDV Polyprotein (Fragment) OS=Bovine viral diarrhea virus OX=11099 PE=4 SV=1
2::Q91N45 **Mass:** 68140 **Score:** 76 **Matches:** 1(1) **Sequences:** 1(1)
>tr|Q91N45|Q91N45_BVDV Polyprotein (Fragment) OS=Bovine viral diarrhea virus OX=11099 PE=4 SV=1
2::Q91N49 **Mass:** 110038 **Score:** 76 **Matches:** 1(1) **Sequences:** 1(1)
>tr|Q91N49|Q91N49_BVDV Genome polyprotein (Fragment) OS=Bovine viral diarrhea virus OX=11099 PE=4 SV=1

2::O11993 Mass: 563508 Score: 76 Matches: 1(1) Sequences: 1(1)
>tr|O11993|O11993_BVDV Uncharacterized protein OS=Bovine viral diarrhea virus OX=11099 PE=4 SV=1
2::A0A2C9C261 Mass: 457871 Score: 76 Matches: 1(1) Sequences: 1(1)
>tr|A0A2C9C261|A0A2C9C261_BVDV Genome polyprotein OS=Bovine viral diarrhea virus OX=11099 PE=4 SV=1
2::Q9E2W4 Mass: 72602 Score: 76 Matches: 1(1) Sequences: 1(1)
>tr|Q9E2W4|Q9E2W4_9FLAV Polyprotein (Fragment) OS=Bovine viral diarrhea virus 2 OX=54315 PE=4 SV=1
2::Q9WR77 Mass: 44830 Score: 76 Matches: 1(1) Sequences: 1(1)
>tr|Q9WR77|Q9WR77_9FLAV Polyprotein (Fragment) OS=Bovine viral diarrhea virus 2 OX=54315 PE=2 SV=1
2::Q9WR81 Mass: 48623 Score: 76 Matches: 1(1) Sequences: 1(1)
>tr|Q9WR81|Q9WR81_9FLAV Polyprotein (Fragment) OS=Bovine viral diarrhea virus 2 OX=54315 PE=2 SV=1
2::Q9WR82 Mass: 41184 Score: 76 Matches: 1(1) Sequences: 1(1)
>tr|Q9WR82|Q9WR82_9FLAV Polyprotein (Fragment) OS=Bovine viral diarrhea virus 2 OX=54315 PE=2 SV=1
2::Q9WR84 Mass: 25963 Score: 76 Matches: 1(1) Sequences: 1(1)
>tr|Q9WR84|Q9WR84_9FLAV Polyprotein (Fragment) OS=Bovine viral diarrhea virus 2 OX=54315 PE=2 SV=1
2::Q9WR86 Mass: 35517 Score: 76 Matches: 1(1) Sequences: 1(1)
>tr|Q9WR86|Q9WR86_9FLAV Polyprotein (Fragment) OS=Bovine viral diarrhea virus 2 OX=54315 PE=2 SV=1
2::Q04232 Mass: 211514 Score: 76 Matches: 1(1) Sequences: 1(1)
>tr|Q04232|Q04232_BVDV Genome polyprotein (Fragment) OS=Bovine viral diarrhea virus OX=11099 PE=4 SV=1
2::Q65810 Mass: 119120 Score: 76 Matches: 1(1) Sequences: 1(1)
>tr|Q65810|Q65810_BVDV Genome polyprotein (Fragment) OS=Bovine viral diarrhea virus OX=11099 PE=4 SV=1

---

10. 2::A0A345AYC4 **Mass:** 6048 **Score:** 75 **Matches:** 2(2) **Sequences:** 1(1) **emPAI:** 0.94
>tr|A0A345AYC4|A0A345AYC4_9CAUD Uncharacterized protein OS=Synechococcus T7-like virus S-TIP37 OX=1332145 GN=STIP37_44 PE=4 SV=1

Query	Observed	Mr(expt)	Mr(calc)	ppm	Miss	Score	Expect	Rank	Unique	Peptide
263	430.2572	858.4999	858.4997	0.29	0	57	0.00062	1	U	R.MIVDLLR.A 264

---

11. 2::A0A068EKL1 **Mass:** 46526 **Score:** 71 **Matches:** 2(2) **Sequences:** 1(1) **emPAI:** 0.09
>tr|A0A068EKL1|A0A068EKL1_9POXV Ankyrin repeat protein OS=Pigeonpox virus OX=10264 GN=fep_016 PE=4 SV=1

Query	Observed	Mr(expt)	Mr(calc)	ppm	Miss	Score	Expect	Rank	Unique	Peptide
63	376.7129	751.4112	751.4116	-0.48	0	54	0.0012	1	U	K.LSYEIK.Y 64

Proteins matching the same set of peptides:
2::A0A068EL77 Mass: 46093 Score: 71 Matches: 2(2) Sequences: 1(1)
>tr|A0A068EL77|A0A068EL77_9POXV Ankyrin repeat protein OS=Penguinpox virus OX=648998 GN=pepv_016 PE=4 SV=1
2::A0A1X9Q118 Mass: 56950 Score: 71 Matches: 2(2) Sequences: 1(1)
>tr|A0A1X9Q118|A0A1X9Q118_9VIRU Putative VP4 OS=Human gut microviridae SH-CHD8 OX=1986033 PE=4 SV=1

---

12. 2::A0A0M4AGG4 **Mass:** 97073 **Score:** 70 **Matches:** 2(2) **Sequences:** 1(1) **emPAI:** 0.04
>tr|A0A0M4AGG4|A0A0M4AGG4_9HIV1 Envelope glycoprotein gp160 OS=Human immunodeficiency virus 1 OX=11676 GN=env PE=3 SV=1

Query	Observed	Mr(expt)	Mr(calc)	ppm	Miss	Score	Expect	Rank	Unique	Peptide
89	387.2288	772.4430	772.4443	-1.69	0	59	0.0012	1	U	R.TVELVGR.S 88

Proteins matching the same set of peptides:
2::A0A0R6ACR6 Mass: 98513 Score: 70 Matches: 2(2) Sequences: 1(1)
>tr|A0A0R6ACR6|A0A0R6ACR6_9HIV1 Envelope glycoprotein gp160 OS=Human immunodeficiency virus 1 OX=11676 GN=env PE=3 SV=1
2::A0A0R6BNW3 Mass: 98651 Score: 70 Matches: 2(2) Sequences: 1(1)
>tr|A0A0R6BNW3|A0A0R6BNW3_9HIV1 Envelope glycoprotein gp160 OS=Human immunodeficiency virus 1 OX=11676 GN=env PE=3 SV=1
2::A0A0R6C436 Mass: 98597 Score: 70 Matches: 2(2) Sequences: 1(1)
>tr|A0A0R6C436|A0A0R6C436_9HIV1 Envelope glycoprotein gp160 OS=Human immunodeficiency virus 1 OX=11676 GN=env PE=3 SV=1
2::A0A0R6C449 Mass: 98493 Score: 70 Matches: 2(2) Sequences: 1(1)
>tr|A0A0R6C449|A0A0R6C449_9HIV1 Envelope glycoprotein gp160 OS=Human immunodeficiency virus 1 OX=11676 GN=env PE=3 SV=1
2::A0A0R6C463 Mass: 98609 Score: 70 Matches: 2(2) Sequences: 1(1)
>tr|A0A0R6C463|A0A0R6C463_9HIV1 Envelope glycoprotein gp160 OS=Human immunodeficiency virus 1 OX=11676 GN=env PE=3 SV=1
2::A0A0R6CNE9 Mass: 98491 Score: 70 Matches: 2(2) Sequences: 1(1)
>tr|A0A0R6CNE9|A0A0R6CNE9_9HIV1 Envelope glycoprotein gp160 OS=Human immunodeficiency virus 1 OX=11676 GN=env PE=3 SV=1
2::A0A0R6CNG2 Mass: 98585 Score: 70 Matches: 2(2) Sequences: 1(1)
>tr|A0A0R6CNG2|A0A0R6CNG2_9HIV1 Envelope glycoprotein gp160 OS=Human immunodeficiency virus 1 OX=11676 GN=env PE=3 SV=1
2::A0A0R6CNH2 Mass: 98593 Score: 70 Matches: 2(2) Sequences: 1(1)
>tr|A0A0R6CNH2|A0A0R6CNH2_9HIV1 Envelope glycoprotein gp160 OS=Human immunodeficiency virus 1 OX=11676 GN=env PE=3 SV=1
2::A0A0R6CNI4 Mass: 98521 Score: 70 Matches: 2(2) Sequences: 1(1)
>tr|A0A0R6CNI4|A0A0R6CNI4_9HIV1 Envelope glycoprotein gp160 OS=Human immunodeficiency virus 1 OX=11676 GN=env PE=3 SV=1
2::A0A0R6CXC9 Mass: 98550 Score: 70 Matches: 2(2) Sequences: 1(1)
>tr|A0A0R6CXC9|A0A0R6CXC9_9HIV1 Envelope glycoprotein gp160 OS=Human immunodeficiency virus 1 OX=11676 GN=env PE=3 SV=1
2::A0A0R6CY26 Mass: 98621 Score: 70 Matches: 2(2) Sequences: 1(1)
>tr|A0A0R6CY26|A0A0R6CY26_9HIV1 Envelope glycoprotein gp160 OS=Human immunodeficiency virus 1 OX=11676 GN=env PE=3 SV=1
2::A0A0R6CY34 Mass: 98547 Score: 70 Matches: 2(2) Sequences: 1(1)
>tr|A0A0R6CY34|A0A0R6CY34_9HIV1 Envelope glycoprotein gp160 OS=Human immunodeficiency virus 1 OX=11676 GN=env PE=3 SV=1
2::A0A0R6CY42 Mass: 98593 Score: 70 Matches: 2(2) Sequences: 1(1)
>tr|A0A0R6CY42|A0A0R6CY42_9HIV1 Envelope glycoprotein gp160 OS=Human immunodeficiency virus 1 OX=11676 GN=env PE=3 SV=1
2::A0A0R6CYA9 Mass: 98656 Score: 70 Matches: 2(2) Sequences: 1(1)
>tr|A0A0R6CYA9|A0A0R6CYA9_9HIV1 Envelope glycoprotein gp160 OS=Human immunodeficiency virus 1 OX=11676 GN=env PE=3 SV=1
2::A0A0R6D370 Mass: 98579 Score: 70 Matches: 2(2) Sequences: 1(1)
>tr|A0A0R6D370|A0A0R6D370_9HIV1 Envelope glycoprotein gp160 OS=Human immunodeficiency virus 1 OX=11676 GN=env PE=3 SV=1
2::A0A0R6D3B1 Mass: 98539 Score: 70 Matches: 2(2) Sequences: 1(1)
>tr|A0A0R6D3B1|A0A0R6D3B1_9HIV1 Envelope glycoprotein gp160 OS=Human immunodeficiency virus 1 OX=11676 GN=env PE=3 SV=1
2::A0A0R6D3D0 Mass: 98625 Score: 70 Matches: 2(2) Sequences: 1(1)
>tr|A0A0R6D3D0|A0A0R6D3D0_9HIV1 Envelope glycoprotein gp160 OS=Human immunodeficiency virus 1 OX=11676 GN=env PE=3 SV=1
2::A0A0R6D631 Mass: 98637 Score: 70 Matches: 2(2) Sequences: 1(1)
>tr|A0A0R6D631|A0A0R6D631_9HIV1 Envelope glycoprotein gp160 OS=Human immunodeficiency virus 1 OX=11676 GN=env PE=3 SV=1
2::A0A0R6D650 Mass: 98684 Score: 70 Matches: 2(2) Sequences: 1(1)
>tr|A0A0R6D650|A0A0R6D650_9HIV1 Envelope glycoprotein gp160 OS=Human immunodeficiency virus 1 OX=11676 GN=env PE=3 SV=1
2::A0A0R6D6L9 Mass: 98566 Score: 70 Matches: 2(2) Sequences: 1(1)
>tr|A0A0R6D6L9|A0A0R6D6L9_9HIV1 Envelope glycoprotein gp160 OS=Human immunodeficiency virus 1 OX=11676 GN=env PE=3 SV=1
2::A0A0R6D7T1 Mass: 98621 Score: 70 Matches: 2(2) Sequences: 1(1)
>tr|A0A0R6D7T1|A0A0R6D7T1_9HIV1 Envelope glycoprotein gp160 OS=Human immunodeficiency virus 1 OX=11676 GN=env PE=3 SV=1
2::A0A0R6D7W2 Mass: 98593 Score: 70 Matches: 2(2) Sequences: 1(1)
>tr|A0A0R6D7W2|A0A0R6D7W2_9HIV1 Envelope glycoprotein gp160 OS=Human immunodeficiency virus 1 OX=11676 GN=env PE=3 SV=1
2::B0YP87 Mass: 97441 Score: 70 Matches: 2(2) Sequences: 1(1)
>tr|B0YP87|B0YP87_9HIV1 Envelope glycoprotein gp160 (Fragment) OS=Human immunodeficiency virus 1 OX=11676 GN=env PE=3 SV=1
2::B0YP89 Mass: 98074 Score: 70 Matches: 2(2) Sequences: 1(1)
>tr|B0YP89|B0YP89_9HIV1 Envelope glycoprotein gp160 (Fragment) OS=Human immunodeficiency virus 1 OX=11676 GN=env PE=3 SV=1

---

13. 2::A0A0A8J9R3 **Mass:** 382428 **Score:** 69 **Matches:** 2(2) **Sequences:** 1(1) **emPAI:** 0.01
>tr|A0A0A8J9R3|A0A0A8J9R3_9CAUD Uncharacterized protein OS=Klebsiella phage K64-1 OX=1439894 PE=4 SV=1

Query	Observed	Mr(expt)	Mr(calc)	ppm	Miss	Score	Expect	Rank	Unique	Peptide
25	361.6944	721.3743	721.3759	-2.15	0	54	0.0016	1	U	K.YGDLVR.T 26

Proteins matching the same set of peptides:
2::H6X3M7 Mass: 382912 Score: 69 Matches: 2(2) Sequences: 1(1)
>tr|H6X3M7|H6X3M7_9CAUD Uncharacterized protein OS=Enterobacteria phage vB_KleM-RaK2 OX=1147094 GN=RaK2_0070 PE=4 SV=1

---

14. 2::A0A1U9WQR1 **Mass:** 99414 **Score:** 66 **Matches:** 1(1) **Sequences:** 1(1) **emPAI:** 0.04
>tr|A0A1U9WQR1|A0A1U9WQR1_9CAUD Tail protein OS=Geobacillus phage TP-84 OX=1965361 PE=4 SV=1

Query	Observed	Mr(expt)	Mr(calc)	ppm	Miss	Score	Expect	Rank	Unique	Peptide
777	529.2800	1056.5454	1056.5451	0.28	0	66	8.5e-05	1	U	K.TEVELNQPK.K

15. 2::A0A345BP79 **Mass:** 17137 **Score:** 65 **Matches:** 2(2) **Sequences:** 1(1) **emPAI:** 0.27
>tr|A0A345BP79|A0A345BP79_9VIRU Uncharacterized protein OS=CRESS virus sp. OX=2202563 PE=4 SV=1

Query	Observed	Mr(expt)	Mr(calc)	ppm	Miss	Score	Expect	Rank	Unique	Peptide
100	391.2687	780.5228	780.5221	0.79	0	48	0.00039	1	U	R.LLPLLGR.G 99

16. 2::A0A1X9Q0Z7 **Mass:** 44225 **Score:** 64 **Matches:** 1(1) **Sequences:** 1(1) **emPAI:** 0.10
>tr|A0A1X9Q0Z7|A0A1X9Q0Z7_9VIRU Putative VP4 OS=Human gut gokushovirus OX=1986031 PE=4 SV=1

Query	Observed	Mr(expt)	Mr(calc)	ppm	Miss	Score	Expect	Rank	Unique	Peptide
871	545.8161	1089.6177	1089.6182	-0.47	1	64	0.00021	1	U	R.KLANDALFAK.L

17. 2::A0A0M3SUP6 **Mass:** 49172 **Score:** 64 **Matches:** 2(2) **Sequences:** 1(1) **emPAI:** 0.09
>tr|A0A0M3SUP6|A0A0M3SUP6_9VIRU RNA-dependent RNA polymerase (Fragment) OS=Rhizoctonia solani beny-like virus 1 OX=1708385 PE=4 SV=1

Query	Observed	Mr(expt)	Mr(calc)	ppm	Miss	Score	Expect	Rank	Unique	Peptide
15	358.7120	715.4094	715.4116	-3.11	0	54	0.0029	1	U	R.IVEEVK.V 14

Proteins matching the same set of peptides:
2::A0A127AW26 **Mass:** 20044 **Score:** 64 **Matches:** 2(2) **Sequences:** 1(1)
>tr|A0A127AW26|A0A127AW26_9CAUD Uncharacterized protein OS=Bacillus phage SP-15 OX=1792032 GN=SP15_039 PE=4 SV=1
2::A0A1L3KLR4 **Mass:** 49735 **Score:** 64 **Matches:** 2(2) **Sequences:** 1(1)
>tr|A0A1L3KLR4|A0A1L3KLR4_9VIRU RdRp OS=Wenling partiti-like virus 13 OX=1923519 PE=4 SV=1
2::A0A3G5AHH2 **Mass:** 53785 **Score:** 64 **Matches:** 2(2) **Sequences:** 1(1)
>tr|A0A3G5AHH2|A0A3G5AHH2_9VIRU Uncharacterized protein OS=Sylvanvirus sp. OX=2487774 GN=Sylvanvirus4_2 PE=4 SV=1
2::A0MNA8 **Mass:** 17169 **Score:** 64 **Matches:** 2(2) **Sequences:** 1(1)
>tr|A0MNA8|A0MNA8_9CAUD Uncharacterized protein OS=Thermus phage phiYS40 OX=407392 GN=YS40_090 PE=4 SV=1
2::G0T5E5 **Mass:** 52997 **Score:** 64 **Matches:** 2(2) **Sequences:** 1(1)
>tr|G0T5E5|G0T5E5_IRV9 Uncharacterized protein OS=Wiseana iridescent virus OX=68347 PE=4 SV=1
2::J9PV61 **Mass:** 25120 **Score:** 64 **Matches:** 2(2) **Sequences:** 1(1)
>tr|J9PV61|J9PV61_9CAUD Putative radical activating enzyme OS=Bacillus phage BCD7 OX=1136534 GN=BCD7_0027 PE=3 SV=1

18. 2::D7SGS7 **Mass:** 46496 **Score:** 63 **Matches:** 2(2) **Sequences:** 1(1) **emPAI:** 0.09
>tr|D7SGS7|D7SGS7_9MONO Polymerase (Fragment) OS=Paramyxovirus bat/GH47/2008 OX=589524 GN=L PE=4 SV=1

Query	Observed	Mr(expt)	Mr(calc)	ppm	Miss	Score	Expect	Rank	Unique	Peptide
697	516.3037	1030.5928	1030.5910	1.72	0	49	0.0048	1	U	K.VIDLETTLK.T 696

Proteins matching the same set of peptides:
2::K7XAQ9 **Mass:** 259901 **Score:** 63 **Matches:** 2(2) **Sequences:** 1(1)
>tr|K7XAQ9|K7XAQ9_9MONO RNA-directed RNA polymerase L OS=Achimota virus 1 OX=1261100 GN=L PE=3 SV=1

19. 2::A0A146JG10 **Mass:** 47119 **Score:** 63 **Matches:** 1(1) **Sequences:** 1(1) **emPAI:** 0.09
>tr|A0A146JG10|A0A146JG10_9VIRU Conserved putative translation elongation factor 1-alpha OS=Tokyovirus A1 OX=1826170 PE=4 SV=1

Query	Observed	Mr(expt)	Mr(calc)	ppm	Miss	Score	Expect	Rank	Unique	Peptide
192	416.7679	831.5212	831.5218	-0.70	0	63	8.4e-05	1	U	R.LVVFINK.M

20. 2::A0A510C2I1 **Mass:** 62569 **Score:** 62 **Matches:** 2(2) **Sequences:** 1(1) **emPAI:** 0.07
>tr|A0A510C2I1|A0A510C2I1_9RHAB Putative G protein OS=Red clover associated cytorhabdovirus 1 OX=2448906 PE=4 SV=1

Query	Observed	Mr(expt)	Mr(calc)	ppm	Miss	Score	Expect	Rank	Unique	Peptide
53	372.7099	743.4052	743.4079	-3.60	1	49	0.003	1	U	K.KGFHAGK.V 52

21. 2::A0A482JV29 **Mass:** 224784 **Score:** 61 **Matches:** 2(2) **Sequences:** 1(1) **emPAI:** 0.02
>tr|A0A482JV29|A0A482JV29_9RHAB RNA-directed RNA polymerase L OS=Lampyris noctiluca rhabdo-like virus 1 OX=2552994 PE=3 SV=1

Query	Observed	Mr(expt)	Mr(calc)	ppm	Miss	Score	Expect	Rank	Unique	Peptide
321	445.7223	889.4300	889.4294	0.75	0	49	0.0035	1	U	K.EWIATDR.A 320

22. 2::A0A089FY51 **Mass:** 391692 **Score:** 61 **Matches:** 2(2) **Sequences:** 1(1) **emPAI:** 0.01
>tr|A0A089FY51|A0A089FY51_9NIDO Putative 1b protein OS=Mikumi yellow baboon virus 1 OX=1546177 GN=ORF1ab PE=4 SV=1

Query	Observed	Mr(expt)	Mr(calc)	ppm	Miss	Score	Expect	Rank	Unique	Peptide
354	452.2608	902.5070	902.5073	-0.32	0	50	0.0031	1	U	K.IISSVEGAK.L 355

Proteins matching the same set of peptides:
2::A0A089FY76 **Mass:** 225997 **Score:** 61 **Matches:** 2(2) **Sequences:** 1(1)
>tr|A0A089FY76|A0A089FY76_9NIDO 1a replicase OS=Mikumi yellow baboon virus 1 OX=1546177 GN=ORF1a PE=4 SV=1
2::A0A089G0A8 **Mass:** 391618 **Score:** 61 **Matches:** 2(2) **Sequences:** 1(1)
>tr|A0A089G0A8|A0A089G0A8_9NIDO Putative 1b protein OS=Mikumi yellow baboon virus 1 OX=1546177 GN=ORF1ab PE=4 SV=1
2::A0A089G181 **Mass:** 226058 **Score:** 61 **Matches:** 2(2) **Sequences:** 1(1)
>tr|A0A089G181|A0A089G181_9NIDO 1a replicase OS=Mikumi yellow baboon virus 1 OX=1546177 GN=ORF1a PE=4 SV=1
2::A0A089G1V1 **Mass:** 226140 **Score:** 61 **Matches:** 2(2) **Sequences:** 1(1)
>tr|A0A089G1V1|A0A089G1V1_9NIDO 1a replicase OS=Mikumi yellow baboon virus 1 OX=1546177 GN=ORF1a PE=4 SV=1
2::A0A089H395 **Mass:** 391764 **Score:** 61 **Matches:** 2(2) **Sequences:** 1(1)
>tr|A0A089H395|A0A089H395_9NIDO Putative 1b protein OS=Mikumi yellow baboon virus 1 OX=1546177 GN=ORF1ab PE=4 SV=1

23. 2::A0A0M5M5R9 **Mass:** 16061 **Score:** 60 **Matches:** 2(2) **Sequences:** 1(1) **emPAI:** 0.29
>tr|A0A0M5M5R9|A0A0M5M5R9_9CAUD SsDNA-binding protein OS=Citrobacter phage Margaery OX=1701810 GN=CPT_Margaery222 PE=4 SV=1

Query	Observed	Mr(expt)	Mr(calc)	ppm	Miss	Score	Expect	Rank	Unique	Peptide
281	435.2480	868.4815	868.4807	0.94	0	49	0.0023	1	U	R.LLFYTGR.H 282

Proteins matching the same set of peptides:
2::A0A1W5N0M8 **Mass:** 16061 **Score:** 60 **Matches:** 2(2) **Sequences:** 1(1)
>tr|A0A1W5N0M8|A0A1W5N0M8_9CAUD Recombination, repair and ssDNA binding protein OS=Cronobacter phage vB_CsaM_leB OX=1885242 GN=B_221 PE=4 SV=1
2::A0A1W5P0W1 **Mass:** 16070 **Score:** 60 **Matches:** 2(2) **Sequences:** 1(1)
>tr|A0A1W5P0W1|A0A1W5P0W1_9CAUD Recombination, repair and ssDNA binding protein OS=Cronobacter phage vB_CsaM_leE OX=1873954 GN=E_226 PE=4 SV=1
2::A0A1X8VCA5 **Mass:** 16061 **Score:** 60 **Matches:** 2(2) **Sequences:** 1(1)
>tr|A0A1X8VCA5|A0A1X8VCA5_9CAUD Recombination, repair and sssDNA binding protein OS=Cronobacter phage vB_CsaM_leN OX=1885245 GN=N_227 PE=4 SV=1
2::A0A3B8DIS1 **Mass:** 16061 **Score:** 60 **Matches:** 2(2) **Sequences:** 1(1)
>tr|A0A3B8DIS1|A0A3B8DIS1_9CAUD UvsY-like protein OS=Citrobacter phage Maroon OX=2315469 GN=CPT_Maroon_218 PE=4 SV=1
2::K4F7S7 **Mass:** 16061 **Score:** 60 **Matches:** 2(2) **Sequences:** 1(1)
>tr|K4F7S7|K4F7S7_9CAUD Recombination, repair and ssDNA binding protein OS=Cronobacter phage vB_CsaM_GAP161 OX=1141138 GN=GAP161_213 PE=4 SV=1

24. 2::A0A1L2CVK2 **Mass:** 19336 **Score:** 60 **Matches:** 2(2) **Sequences:** 1(1) **emPAI:** 0.24
>tr|A0A1L2CVK2|A0A1L2CVK2_9CAUD Uncharacterized protein OS=Pectobacterium phage CBB OX=1792242 GN=CBB_463 PE=4 SV=1

Query	Observed	Mr(expt)	Mr(calc)	ppm	Miss	Score	Expect	Rank	Unique	Peptide
38	365.2343	728.4540	728.4545	-0.64	0	51	0.0065	1	U	R.TLQVLR.M 37

Proteins matching the same set of peptides:
2::K4F7S2 **Mass:** 19272 **Score:** 60 **Matches:** 2(2) **Sequences:** 1(1)
>tr|K4F7S2|K4F7S2_9CAUD Uncharacterized protein OS=Cronobacter phage vB_CsaM_GAP32 OX=1141136 GN=GAP32_442 PE=4 SV=1

25. 2::A0A516L0H2 **Mass:** 13576 **Score:** 60 **Matches:** 1(1) **Sequences:** 1(1) **emPAI:** 0.36
>tr|A0A516L0H2|A0A516L0H2_9VIRU Uncharacterized protein OS=Prokaryotic dsDNA virus sp. OX=2591644 GN=GOVbin2950_25 PE=4 SV=1

Query	Observed	Mr(expt)	Mr(calc)	ppm	Miss	Score	Expect	Rank	Unique	Peptide
315	443.7664	885.5182	885.5171	1.29	0	60	0.00067	1	U	K.ALLEALEK.S

Proteins matching the same set of peptides:
2::A0A516L865 **Mass:** 13665 **Score:** 60 **Matches:** 1(1) **Sequences:** 1(1)
>tr|A0A516L865|A0A516L865_9VIRU Uncharacterized protein OS=Prokaryotic dsDNA virus sp. OX=2591644 GN=Unbinned4311contig1001_30 PE=4 SV=1
2::A0A516LUY4 **Mass:** 13201 **Score:** 60 **Matches:** 1(1) **Sequences:** 1(1)
>tr|A0A516LUY4|A0A516LUY4_9VIRU HTH_8 domain-containing protein OS=Prokaryotic dsDNA virus sp. OX=2591644 GN=Unbinned92contig1004_18 PE=4 SV=1

---

26. 2::A0A3Q8J4E7 **Mass:** 91595 **Score:** 59 **Matches:** 1(1) **Sequences:** 1(1) **emPAI:** 0.05
>tr|A0A3Q8J4E7|A0A3Q8J4E7_9GAMA Glycoprotein B OS=Vombatid gammaherpesvirus 1 OX=2052651 GN=ORF8 PE=4 SV=1

Query	Observed	Mr(expt)	Mr(calc)	ppm	Miss	Score	Expect	Rank	Unique	Peptide
16	358.7184	715.4223	715.4228	-0.69	0	59	0.0016	1	U	K.TVIIDR.T

Proteins matching the same set of peptides:
2::V5K6X0 **Mass:** 65777 **Score:** 59 **Matches:** 1(1) **Sequences:** 1(1)
>tr|V5K6X0|V5K6X0_9MONO Transmembrane protein OS=Miniopterus schreibersii paramyxovirus OX=1387879 GN=TM PE=4 SV=1

---

27. 2::A0A097BY06 **Mass:** 77954 **Score:** 59 **Matches:** 1(1) **Sequences:** 1(1) **emPAI:** 0.06
>tr|A0A097BY06|A0A097BY06_9CAUD Minor tail protein OS=Mycobacterium phage Power OX=1552733 GN=PBI_POWER_31 PE=4 SV=1

Query	Observed	Mr(expt)	Mr(calc)	ppm	Miss	Score	Expect	Rank	Unique	Peptide
79	385.2439	768.4732	768.4745	-1.69	0	59	8.3e-05	1	U	R.LPLDALK.T

Proteins matching the same set of peptides:
2::A0A097EWN0 **Mass:** 78208 **Score:** 59 **Matches:** 1(1) **Sequences:** 1(1)
>tr|A0A097EWN0|A0A097EWN0_9CAUD Minor tail protein OS=Mycobacterium phage SweetiePie OX=1555235 GN=31 PE=4 SV=1
2::A0A0M3UL68 **Mass:** 84250 **Score:** 59 **Matches:** 1(1) **Sequences:** 1(1)
>tr|A0A0M3UL68|A0A0M3UL68_9CAUD Minor tail protein OS=Mycobacterium phage LadyBird OX=1718166 GN=SEA_LADYBIRD_31 PE=4 SV=1
2::A0A1C9LZW9 **Mass:** 77898 **Score:** 59 **Matches:** 1(1) **Sequences:** 1(1)
>tr|A0A1C9LZW9|A0A1C9LZW9_9CAUD Minor tail protein OS=Mycobacterium phage DudeLittle OX=1897766 GN=SEA_DUDELITTLE_31 PE=4 SV=1
2::A0A220NSE9 **Mass:** 63313 **Score:** 59 **Matches:** 1(1) **Sequences:** 1(1)
>tr|A0A220NSE9|A0A220NSE9_9CAUD Minor tail protein OS=Mycobacterium phage Heffalump OX=1983575 GN=33 PE=4 SV=1
2::A0A2D1G5J0 **Mass:** 77933 **Score:** 59 **Matches:** 1(1) **Sequences:** 1(1)
>tr|A0A2D1G5J0|A0A2D1G5J0_9CAUD Minor tail protein OS=Mycobacterium phage Anselm OX=2041517 GN=SEA_ANSELM_31 PE=4 SV=1
2::A0A2D1G9Y3 **Mass:** 78257 **Score:** 59 **Matches:** 1(1) **Sequences:** 1(1)
>tr|A0A2D1G9Y3|A0A2D1G9Y3_9CAUD Minor tail protein OS=Mycobacterium phage Dalmatian OX=2041527 GN=SEA_DALMATIAN_31 PE=4 SV=1
2::A0A2D1GJ93 **Mass:** 78238 **Score:** 59 **Matches:** 1(1) **Sequences:** 1(1)
>tr|A0A2D1GJ93|A0A2D1GJ93_9CAUD Minor tail protein OS=Mycobacterium phage SnapTap OX=2041549 GN=SEA_SNAPTAP_31 PE=4 SV=1
2::A0A2R4AP72 **Mass:** 78230 **Score:** 59 **Matches:** 1(1) **Sequences:** 1(1)
>tr|A0A2R4AP72|A0A2R4AP72_9CAUD Minor tail protein OS=Mycobacterium phage Fameo OX=2126943 GN=31 PE=4 SV=1
2::A0A2Z5H0E5 **Mass:** 78208 **Score:** 59 **Matches:** 1(1) **Sequences:** 1(1)
>tr|A0A2Z5H0E5|A0A2Z5H0E5_9CAUD Minor tail protein OS=Mycobacterium phage Crucio OX=2234072 GN=31 PE=4 SV=1
2::A0A385D5H4 **Mass:** 77889 **Score:** 59 **Matches:** 1(1) **Sequences:** 1(1)
>tr|A0A385D5H4|A0A385D5H4_9CAUD Minor tail protein OS=Mycobacterium phage QueenBeesly OX=2301544 GN=31 PE=4 SV=1
2::A0A5J6TLJ6 **Mass:** 78367 **Score:** 59 **Matches:** 1(1) **Sequences:** 1(1)
>tr|A0A5J6TLJ6|A0A5J6TLJ6_9CAUD Minor tail protein OS=Mycobacterium phage SemperFi OX=2599879 GN=33 PE=4 SV=1
2::M4HZ75 **Mass:** 84118 **Score:** 59 **Matches:** 1(1) **Sequences:** 1(1)
>tr|M4HZ75|M4HZ75_9CAUD Minor tail subunit OS=Mycobacterium phage First OX=1245814 GN=First_0031 PE=4 SV=1
2::W8E958 **Mass:** 84119 **Score:** 59 **Matches:** 1(1) **Sequences:** 1(1)
>tr|W8E958|W8E958_9CAUD Minor tail subunit OS=Mycobacterium phage 20ES OX=1458726 GN=20ES_31 PE=4 SV=1

---

28. 2::A0A5J6T603 **Mass:** 86865 **Score:** 59 **Matches:** 1(1) **Sequences:** 1(1) **emPAI:** 0.05
>tr|A0A5J6T603|A0A5J6T603_9CAUD Tape measure protein OS=Bacillus phage 280BB001 OX=2601665 GN=29 PE=4 SV=1

Query	Observed	Mr(expt)	Mr(calc)	ppm	Miss	Score	Expect	Rank	Unique	Peptide
12	358.2207	714.4268	714.4276	-1.03	0	59	0.00088	1	U	K.IIDAGVK.L

Proteins matching the same set of peptides:
2::A0A5J6T604 **Mass:** 86865 **Score:** 59 **Matches:** 1(1) **Sequences:** 1(1)
>tr|A0A5J6T604|A0A5J6T604_9CAUD Tape measure protein OS=Bacillus phage 019DV002 OX=2601653 GN=27 PE=4 SV=1
2::A0A5J6T6Y7 **Mass:** 86865 **Score:** 59 **Matches:** 1(1) **Sequences:** 1(1)
>tr|A0A5J6T6Y7|A0A5J6T6Y7_9CAUD Tape measure protein OS=Bacillus phage 276BB001 OX=2601664 GN=29 PE=4 SV=1
2::A0A5J6TAH8 **Mass:** 86865 **Score:** 59 **Matches:** 1(1) **Sequences:** 1(1)
>tr|A0A5J6TAH8|A0A5J6TAH8_9CAUD Tape measure protein OS=Bacillus phage 019DV004 OX=2601654 GN=27 PE=4 SV=1

---

29. 2::A0A023JAL3 **Mass:** 92933 **Score:** 58 **Matches:** 1(1) **Sequences:** 1(1) **emPAI:** 0.05
>tr|A0A023JAL3|A0A023JAL3_9HIV1 Envelope glycoprotein gp160 (Fragment) OS=Human immunodeficiency virus 1 OX=11676 GN=env PE=3 SV=1

Query	Observed	Mr(expt)	Mr(calc)	ppm	Miss	Score	Expect	Rank	Unique	Peptide
35	365.2155	728.4165	728.4181	-2.08	0	58	0.0025	1	U	R.AAELLGR.S

Proteins matching the same set of peptides:
2::A0A023JAS6 **Mass:** 92521 **Score:** 58 **Matches:** 1(1) **Sequences:** 1(1)
>tr|A0A023JAS6|A0A023JAS6_9HIV1 Envelope glycoprotein gp160 (Fragment) OS=Human immunodeficiency virus 1 OX=11676 GN=env PE=3 SV=1
2::A0A023JB08 **Mass:** 92555 **Score:** 58 **Matches:** 1(1) **Sequences:** 1(1)
>tr|A0A023JB08|A0A023JB08_9HIV1 Envelope glycoprotein gp160 (Fragment) OS=Human immunodeficiency virus 1 OX=11676 GN=env PE=3 SV=1
2::A0A023JB17 **Mass:** 92213 **Score:** 58 **Matches:** 1(1) **Sequences:** 1(1)
>tr|A0A023JB17|A0A023JB17_9HIV1 Envelope glycoprotein gp160 (Fragment) OS=Human immunodeficiency virus 1 OX=11676 GN=env PE=3 SV=1
2::A0A023JB77 **Mass:** 92635 **Score:** 58 **Matches:** 1(1) **Sequences:** 1(1)
>tr|A0A023JB77|A0A023JB77_9HIV1 Envelope glycoprotein gp160 (Fragment) OS=Human immunodeficiency virus 1 OX=11676 GN=env PE=3 SV=1
2::A0A023JBC8 **Mass:** 92857 **Score:** 58 **Matches:** 1(1) **Sequences:** 1(1)
>tr|A0A023JBC8|A0A023JBC8_9HIV1 Envelope glycoprotein gp160 (Fragment) OS=Human immunodeficiency virus 1 OX=11676 GN=env PE=3 SV=1
2::A0A023JBE4 **Mass:** 92565 **Score:** 58 **Matches:** 1(1) **Sequences:** 1(1)
>tr|A0A023JBE4|A0A023JBE4_9HIV1 Envelope glycoprotein gp160 (Fragment) OS=Human immunodeficiency virus 1 OX=11676 GN=env PE=3 SV=1
2::A0A023JBH9 **Mass:** 92862 **Score:** 58 **Matches:** 1(1) **Sequences:** 1(1)
>tr|A0A023JBH9|A0A023JBH9_9HIV1 Envelope glycoprotein gp160 (Fragment) OS=Human immunodeficiency virus 1 OX=11676 GN=env PE=3 SV=1
2::A0A023JBI3 **Mass:** 92975 **Score:** 58 **Matches:** 1(1) **Sequences:** 1(1)
>tr|A0A023JBI3|A0A023JBI3_9HIV1 Envelope glycoprotein gp160 (Fragment) OS=Human immunodeficiency virus 1 OX=11676 GN=env PE=3 SV=1
2::A0A023JBL6 **Mass:** 92728 **Score:** 58 **Matches:** 1(1) **Sequences:** 1(1)
>tr|A0A023JBL6|A0A023JBL6_9HIV1 Envelope glycoprotein gp160 (Fragment) OS=Human immunodeficiency virus 1 OX=11676 GN=env PE=3 SV=1
2::A0A023JBM1 **Mass:** 92804 **Score:** 58 **Matches:** 1(1) **Sequences:** 1(1)
>tr|A0A023JBM1|A0A023JBM1_9HIV1 Envelope glycoprotein gp160 (Fragment) OS=Human immunodeficiency virus 1 OX=11676 GN=env PE=3 SV=1
2::A0A023JBQ7 **Mass:** 92489 **Score:** 58 **Matches:** 1(1) **Sequences:** 1(1)
>tr|A0A023JBQ7|A0A023JBQ7_9HIV1 Envelope glycoprotein gp160 (Fragment) OS=Human immunodeficiency virus 1 OX=11676 GN=env PE=3 SV=1
2::A0A023JBU4 **Mass:** 92639 **Score:** 58 **Matches:** 1(1) **Sequences:** 1(1)
>tr|A0A023JBU4|A0A023JBU4_9HIV1 Envelope glycoprotein gp160 (Fragment) OS=Human immunodeficiency virus 1 OX=11676 GN=env PE=3 SV=1
2::A0A023NEN9 **Mass:** 54686 **Score:** 58 **Matches:** 1(1) **Sequences:** 1(1)
>tr|A0A023NEN9|A0A023NEN9_9HIV1 Envelope glycoprotein gp160 (Fragment) OS=Human immunodeficiency virus 1 OX=11676 GN=env PE=4 SV=1
2::A0A0B5KVT5 **Mass:** 98184 **Score:** 58 **Matches:** 1(1) **Sequences:** 1(1)
>tr|A0A0B5KVT5|A0A0B5KVT5_9HIV1 Envelope glycoprotein gp160 OS=Human immunodeficiency virus 1 OX=11676 GN=env PE=3 SV=1
2::A0A0B5LCY3 **Mass:** 99397 **Score:** 58 **Matches:** 1(1) **Sequences:** 1(1)
>tr|A0A0B5LCY3|A0A0B5LCY3_9HIV1 Envelope glycoprotein gp160 OS=Human immunodeficiency virus 1 OX=11676 GN=env PE=3 SV=1
2::A0A0G3IA07 **Mass:** 99395 **Score:** 58 **Matches:** 1(1) **Sequences:** 1(1)
>tr|A0A0G3IA07|A0A0G3IA07_9HIV1 Envelope glycoprotein gp160 OS=Human immunodeficiency virus 1 OX=11676 GN=env PE=3 SV=1
2::A0A0G3IA55 **Mass:** 100936 **Score:** 58 **Matches:** 1(1) **Sequences:** 1(1)
>tr|A0A0G3IA55|A0A0G3IA55_9HIV1 Envelope glycoprotein gp160 OS=Human immunodeficiency virus 1 OX=11676 GN=env PE=3 SV=1
2::A0A0G3IA71 **Mass:** 100891 **Score:** 58 **Matches:** 1(1) **Sequences:** 1(1)
>tr|A0A0G3IA71|A0A0G3IA71_9HIV1 Envelope glycoprotein gp160 OS=Human immunodeficiency virus 1 OX=11676 GN=env PE=3 SV=1
2::A0A0G3IBT4 **Mass:** 100895 **Score:** 58 **Matches:** 1(1) **Sequences:** 1(1)
>tr|A0A0G3IBT4|A0A0G3IBT4_9HIV1 Envelope glycoprotein gp160 OS=Human immunodeficiency virus 1 OX=11676 GN=env PE=3 SV=1
2::A0A0G3IBU7 **Mass:** 100806 **Score:** 58 **Matches:** 1(1) **Sequences:** 1(1)
>tr|A0A0G3IBU7|A0A0G3IBU7_9HIV1 Envelope glycoprotein gp160 OS=Human immunodeficiency virus 1 OX=11676 GN=env PE=3 SV=1

```
2::A0A0G3IBW5    Mass: 99205    Score: 58    Matches: 1(1)  Sequences: 1(1)
>tr|A0A0G3IBW5|A0A0G3IBW5_9HIV1 Envelope glycoprotein gp160 OS=Human immunodeficiency virus 1 OX=11676 GN=env PE=3 SV=1
2::A0A0G3IDS2    Mass: 97965    Score: 58    Matches: 1(1)  Sequences: 1(1)
>tr|A0A0G3IDS2|A0A0G3IDS2_9HIV1 Envelope glycoprotein gp160 OS=Human immunodeficiency virus 1 OX=11676 GN=env PE=3 SV=1
2::A0A0G3IFV7    Mass: 100848   Score: 58    Matches: 1(1)  Sequences: 1(1)
>tr|A0A0G3IFV7|A0A0G3IFV7_9HIV1 Envelope glycoprotein gp160 OS=Human immunodeficiency virus 1 OX=11676 GN=env PE=3 SV=1
2::A0A0H3Y9F9    Mass: 98408    Score: 58    Matches: 1(1)  Sequences: 1(1)
>tr|A0A0H3Y9F9|A0A0H3Y9F9_9HIV1 Envelope glycoprotein gp160 OS=Human immunodeficiency virus 1 OX=11676 GN=env PE=3 SV=1
2::A0A0H3Y9R1    Mass: 98165    Score: 58    Matches: 1(1)  Sequences: 1(1)
>tr|A0A0H3Y9R1|A0A0H3Y9R1_9HIV1 Envelope glycoprotein gp160 OS=Human immunodeficiency virus 1 OX=11676 GN=env PE=3 SV=1
2::A0A0H3YA85    Mass: 97508    Score: 58    Matches: 1(1)  Sequences: 1(1)
>tr|A0A0H3YA85|A0A0H3YA85_9HIV1 Envelope glycoprotein gp160 OS=Human immunodeficiency virus 1 OX=11676 GN=env PE=3 SV=1
2::A0A0H3YAM2    Mass: 96522    Score: 58    Matches: 1(1)  Sequences: 1(1)
>tr|A0A0H3YAM2|A0A0H3YAM2_9HIV1 Envelope glycoprotein gp160 OS=Human immunodeficiency virus 1 OX=11676 GN=env PE=3 SV=1
2::A0A0H3YAQ3    Mass: 99318    Score: 58    Matches: 1(1)  Sequences: 1(1)
>tr|A0A0H3YAQ3|A0A0H3YAQ3_9HIV1 Envelope glycoprotein gp160 OS=Human immunodeficiency virus 1 OX=11676 GN=env PE=3 SV=1
2::A0A0H3YCZ6    Mass: 98367    Score: 58    Matches: 1(1)  Sequences: 1(1)
>tr|A0A0H3YCZ6|A0A0H3YCZ6_9HIV1 Envelope glycoprotein gp160 OS=Human immunodeficiency virus 1 OX=11676 GN=env PE=3 SV=1
2::A0A0H3YD29    Mass: 96413    Score: 58    Matches: 1(1)  Sequences: 1(1)
>tr|A0A0H3YD29|A0A0H3YD29_9HIV1 Envelope glycoprotein gp160 OS=Human immunodeficiency virus 1 OX=11676 GN=env PE=3 SV=1
2::A0A0H3YD33    Mass: 97914    Score: 58    Matches: 1(1)  Sequences: 1(1)
>tr|A0A0H3YD33|A0A0H3YD33_9HIV1 Envelope glycoprotein gp160 OS=Human immunodeficiency virus 1 OX=11676 GN=env PE=3 SV=1
2::A0A0K1H5N5    Mass: 98715    Score: 58    Matches: 1(1)  Sequences: 1(1)
>tr|A0A0K1H5N5|A0A0K1H5N5_9HIV1 Envelope glycoprotein gp160 OS=Human immunodeficiency virus 1 OX=11676 GN=env PE=3 SV=1
2::A0A0K1H5Q2    Mass: 98759    Score: 58    Matches: 1(1)  Sequences: 1(1)
>tr|A0A0K1H5Q2|A0A0K1H5Q2_9HIV1 Envelope glycoprotein gp160 OS=Human immunodeficiency virus 1 OX=11676 GN=env PE=3 SV=1
2::A0A0K1H5R9    Mass: 98683    Score: 58    Matches: 1(1)  Sequences: 1(1)
>tr|A0A0K1H5R9|A0A0K1H5R9_9HIV1 Envelope glycoprotein gp160 OS=Human immunodeficiency virus 1 OX=11676 GN=env PE=3 SV=1
2::A0A0K1H5T2    Mass: 98635    Score: 58    Matches: 1(1)  Sequences: 1(1)
>tr|A0A0K1H5T2|A0A0K1H5T2_9HIV1 Envelope glycoprotein gp160 OS=Human immunodeficiency virus 1 OX=11676 GN=env PE=3 SV=1
2::A0A0K1H655    Mass: 97399    Score: 58    Matches: 1(1)  Sequences: 1(1)
>tr|A0A0K1H655|A0A0K1H655_9HIV1 Envelope glycoprotein gp160 OS=Human immunodeficiency virus 1 OX=11676 GN=env PE=3 SV=1
2::A0A0K1H690    Mass: 97355    Score: 58    Matches: 1(1)  Sequences: 1(1)
>tr|A0A0K1H690|A0A0K1H690_9HIV1 Envelope glycoprotein gp160 OS=Human immunodeficiency virus 1 OX=11676 GN=env PE=3 SV=1
2::A0A0K1H6B8    Mass: 97481    Score: 58    Matches: 1(1)  Sequences: 1(1)
>tr|A0A0K1H6B8|A0A0K1H6B8_9HIV1 Envelope glycoprotein gp160 OS=Human immunodeficiency virus 1 OX=11676 GN=env PE=3 SV=1
2::A0A0K1H6L3    Mass: 97425    Score: 58    Matches: 1(1)  Sequences: 1(1)
>tr|A0A0K1H6L3|A0A0K1H6L3_9HIV1 Envelope glycoprotein gp160 OS=Human immunodeficiency virus 1 OX=11676 GN=env PE=3 SV=1
2::A0A0K1H6R7    Mass: 97423    Score: 58    Matches: 1(1)  Sequences: 1(1)
>tr|A0A0K1H6R7|A0A0K1H6R7_9HIV1 Envelope glycoprotein gp160 OS=Human immunodeficiency virus 1 OX=11676 GN=env PE=3 SV=1
2::A0A0K1H7A6    Mass: 97799    Score: 58    Matches: 1(1)  Sequences: 1(1)
>tr|A0A0K1H7A6|A0A0K1H7A6_9HIV1 Envelope glycoprotein gp160 OS=Human immunodeficiency virus 1 OX=11676 GN=env PE=3 SV=1
2::A0A0K1H7F0    Mass: 97733    Score: 58    Matches: 1(1)  Sequences: 1(1)
>tr|A0A0K1H7F0|A0A0K1H7F0_9HIV1 Envelope glycoprotein gp160 OS=Human immunodeficiency virus 1 OX=11676 GN=env PE=3 SV=1
2::A0A0K1H7I0    Mass: 97838    Score: 58    Matches: 1(1)  Sequences: 1(1)
>tr|A0A0K1H7I0|A0A0K1H7I0_9HIV1 Envelope glycoprotein gp160 OS=Human immunodeficiency virus 1 OX=11676 GN=env PE=3 SV=1
2::A0A0K1H7L6    Mass: 98174    Score: 58    Matches: 1(1)  Sequences: 1(1)
>tr|A0A0K1H7L6|A0A0K1H7L6_9HIV1 Envelope glycoprotein gp160 OS=Human immunodeficiency virus 1 OX=11676 GN=env PE=3 SV=1
2::A0A0K1H7M0    Mass: 97825    Score: 58    Matches: 1(1)  Sequences: 1(1)
>tr|A0A0K1H7M0|A0A0K1H7M0_9HIV1 Envelope glycoprotein gp160 OS=Human immunodeficiency virus 1 OX=11676 GN=env PE=3 SV=1
2::A0A0K1H877    Mass: 97345    Score: 58    Matches: 1(1)  Sequences: 1(1)
>tr|A0A0K1H877|A0A0K1H877_9HIV1 Envelope glycoprotein gp160 OS=Human immunodeficiency virus 1 OX=11676 GN=env PE=3 SV=1
2::A0A0K1H8L3    Mass: 97754    Score: 58    Matches: 1(1)  Sequences: 1(1)
>tr|A0A0K1H8L3|A0A0K1H8L3_9HIV1 Envelope glycoprotein gp160 OS=Human immunodeficiency virus 1 OX=11676 GN=env PE=3 SV=1
2::A0A0K1H8T5    Mass: 97415    Score: 58    Matches: 1(1)  Sequences: 1(1)
>tr|A0A0K1H8T5|A0A0K1H8T5_9HIV1 Envelope glycoprotein gp160 OS=Human immunodeficiency virus 1 OX=11676 GN=env PE=3 SV=1
2::A0A0K1H8T8    Mass: 98358    Score: 58    Matches: 1(1)  Sequences: 1(1)
>tr|A0A0K1H8T8|A0A0K1H8T8_9HIV1 Envelope glycoprotein gp160 OS=Human immunodeficiency virus 1 OX=11676 GN=env PE=3 SV=1
2::A0A0K1H9A0    Mass: 98215    Score: 58    Matches: 1(1)  Sequences: 1(1)
>tr|A0A0K1H9A0|A0A0K1H9A0_9HIV1 Envelope glycoprotein gp160 OS=Human immunodeficiency virus 1 OX=11676 GN=env PE=3 SV=1
2::A0A0K1HBC2    Mass: 100453   Score: 58    Matches: 1(1)  Sequences: 1(1)
>tr|A0A0K1HBC2|A0A0K1HBC2_9HIV1 Envelope glycoprotein gp160 OS=Human immunodeficiency virus 1 OX=11676 GN=env PE=3 SV=1
2::A0A0K1HBE7    Mass: 100439   Score: 58    Matches: 1(1)  Sequences: 1(1)
>tr|A0A0K1HBE7|A0A0K1HBE7_9HIV1 Envelope glycoprotein gp160 OS=Human immunodeficiency virus 1 OX=11676 GN=env PE=3 SV=1
2::A0A0K1HBE9    Mass: 100354   Score: 58    Matches: 1(1)  Sequences: 1(1)
>tr|A0A0K1HBE9|A0A0K1HBE9_9HIV1 Envelope glycoprotein gp160 OS=Human immunodeficiency virus 1 OX=11676 GN=env PE=3 SV=1
2::A0A0K1HBG0    Mass: 100593   Score: 58    Matches: 1(1)  Sequences: 1(1)
>tr|A0A0K1HBG0|A0A0K1HBG0_9HIV1 Envelope glycoprotein gp160 OS=Human immunodeficiency virus 1 OX=11676 GN=env PE=3 SV=1
2::A0A0K1HBJ0    Mass: 100436   Score: 58    Matches: 1(1)  Sequences: 1(1)
>tr|A0A0K1HBJ0|A0A0K1HBJ0_9HIV1 Envelope glycoprotein gp160 OS=Human immunodeficiency virus 1 OX=11676 GN=env PE=3 SV=1
2::A0A0K1HBJ3    Mass: 100534   Score: 58    Matches: 1(1)  Sequences: 1(1)
>tr|A0A0K1HBJ3|A0A0K1HBJ3_9HIV1 Envelope glycoprotein gp160 OS=Human immunodeficiency virus 1 OX=11676 GN=env PE=3 SV=1
2::A0A0K1HBM3    Mass: 100366   Score: 58    Matches: 1(1)  Sequences: 1(1)
>tr|A0A0K1HBM3|A0A0K1HBM3_9HIV1 Envelope glycoprotein gp160 OS=Human immunodeficiency virus 1 OX=11676 GN=env PE=3 SV=1
2::A0A0K2DML6    Mass: 98895    Score: 58    Matches: 1(1)  Sequences: 1(1)
>tr|A0A0K2DML6|A0A0K2DML6_9HIV1 Envelope glycoprotein gp160 OS=Human immunodeficiency virus 1 OX=11676 GN=env PE=3 SV=1
2::A0A0K2DMP8    Mass: 97960    Score: 58    Matches: 1(1)  Sequences: 1(1)
>tr|A0A0K2DMP8|A0A0K2DMP8_9HIV1 Envelope glycoprotein gp160 OS=Human immunodeficiency virus 1 OX=11676 GN=env PE=3 SV=1
2::A0A0K2DMQ1    Mass: 98240    Score: 58    Matches: 1(1)  Sequences: 1(1)
>tr|A0A0K2DMQ1|A0A0K2DMQ1_9HIV1 Envelope glycoprotein gp160 OS=Human immunodeficiency virus 1 OX=11676 GN=env PE=3 SV=1
2::A0A0K2DMS1    Mass: 98213    Score: 58    Matches: 1(1)  Sequences: 1(1)
>tr|A0A0K2DMS1|A0A0K2DMS1_9HIV1 Envelope glycoprotein gp160 OS=Human immunodeficiency virus 1 OX=11676 GN=env PE=3 SV=1
2::A0A0K2DMS4    Mass: 97967    Score: 58    Matches: 1(1)  Sequences: 1(1)
>tr|A0A0K2DMS4|A0A0K2DMS4_9HIV1 Envelope glycoprotein gp160 OS=Human immunodeficiency virus 1 OX=11676 GN=env PE=3 SV=1
2::A0A0K2DMV0    Mass: 98273    Score: 58    Matches: 1(1)  Sequences: 1(1)
>tr|A0A0K2DMV0|A0A0K2DMV0_9HIV1 Envelope glycoprotein gp160 OS=Human immunodeficiency virus 1 OX=11676 GN=env PE=3 SV=1
2::A0A0K2DMV4    Mass: 98901    Score: 58    Matches: 1(1)  Sequences: 1(1)
>tr|A0A0K2DMV4|A0A0K2DMV4_9HIV1 Envelope glycoprotein gp160 OS=Human immunodeficiency virus 1 OX=11676 GN=env PE=3 SV=1
2::A0A0K2DMX4    Mass: 98963    Score: 58    Matches: 1(1)  Sequences: 1(1)
>tr|A0A0K2DMX4|A0A0K2DMX4_9HIV1 Envelope glycoprotein gp160 OS=Human immunodeficiency virus 1 OX=11676 GN=env PE=3 SV=1
2::A0A0K2DMX8    Mass: 98130    Score: 58    Matches: 1(1)  Sequences: 1(1)
>tr|A0A0K2DMX8|A0A0K2DMX8_9HIV1 Envelope glycoprotein gp160 OS=Human immunodeficiency virus 1 OX=11676 GN=env PE=3 SV=1
2::A0A0K2DMY2    Mass: 98768    Score: 58    Matches: 1(1)  Sequences: 1(1)
>tr|A0A0K2DMY2|A0A0K2DMY2_9HIV1 Envelope glycoprotein gp160 OS=Human immunodeficiency virus 1 OX=11676 GN=env PE=3 SV=1
2::A0A0K2DN10    Mass: 98961    Score: 58    Matches: 1(1)  Sequences: 1(1)
>tr|A0A0K2DN10|A0A0K2DN10_9HIV1 Envelope glycoprotein gp160 OS=Human immunodeficiency virus 1 OX=11676 GN=env PE=3 SV=1
2::A0A0K2DN12    Mass: 99581    Score: 58    Matches: 1(1)  Sequences: 1(1)
>tr|A0A0K2DN12|A0A0K2DN12_9HIV1 Envelope glycoprotein gp160 OS=Human immunodeficiency virus 1 OX=11676 GN=env PE=3 SV=1
2::A0A0K2DN35    Mass: 98733    Score: 58    Matches: 1(1)  Sequences: 1(1)
>tr|A0A0K2DN35|A0A0K2DN35_9HIV1 Envelope glycoprotein gp160 OS=Human immunodeficiency virus 1 OX=11676 GN=env PE=3 SV=1
2::A0A0K2DN46    Mass: 98416    Score: 58    Matches: 1(1)  Sequences: 1(1)
>tr|A0A0K2DN46|A0A0K2DN46_9HIV1 Envelope glycoprotein gp160 OS=Human immunodeficiency virus 1 OX=11676 GN=env PE=3 SV=1
2::A0A0K2DN82    Mass: 97943    Score: 58    Matches: 1(1)  Sequences: 1(1)
>tr|A0A0K2DN82|A0A0K2DN82_9HIV1 Envelope glycoprotein gp160 OS=Human immunodeficiency virus 1 OX=11676 GN=env PE=3 SV=1
2::A0A0K2DNA3    Mass: 98975    Score: 58    Matches: 1(1)  Sequences: 1(1)
>tr|A0A0K2DNA3|A0A0K2DNA3_9HIV1 Envelope glycoprotein gp160 OS=Human immunodeficiency virus 1 OX=11676 GN=env PE=3 SV=1
2::A0A0K2DNH7    Mass: 99611    Score: 58    Matches: 1(1)  Sequences: 1(1)
>tr|A0A0K2DNH7|A0A0K2DNH7_9HIV1 Envelope glycoprotein gp160 OS=Human immunodeficiency virus 1 OX=11676 GN=env PE=3 SV=1
2::A0A0U3VN65    Mass: 97759    Score: 58    Matches: 1(1)  Sequences: 1(1)
>tr|A0A0U3VN65|A0A0U3VN65_9HIV1 Envelope glycoprotein gp160 OS=Human immunodeficiency virus 1 OX=11676 GN=env PE=3 SV=1
```

2::A0A191TGE3 **Mass:** 98955 **Score:** 58 **Matches:** 1(1) **Sequences:** 1(1)
>tr|A0A191TGE3|A0A191TGE3_9HIV1 Envelope glycoprotein gp160 OS=Human immunodeficiency virus 1 OX=11676 GN=env PE=2 SV=1
2::A0A1D9IZ71 **Mass:** 98602 **Score:** 58 **Matches:** 1(1) **Sequences:** 1(1)
>tr|A0A1D9IZ71|A0A1D9IZ71_9HIV1 Envelope glycoprotein gp160 OS=Human immunodeficiency virus 1 OX=11676 GN=env PE=3 SV=1
2::A0A1D9IZ77 **Mass:** 98641 **Score:** 58 **Matches:** 1(1) **Sequences:** 1(1)
>tr|A0A1D9IZ77|A0A1D9IZ77_9HIV1 Envelope glycoprotein gp160 OS=Human immunodeficiency virus 1 OX=11676 GN=env PE=3 SV=1
2::A0A1D9IZ78 **Mass:** 98572 **Score:** 58 **Matches:** 1(1) **Sequences:** 1(1)
>tr|A0A1D9IZ78|A0A1D9IZ78_9HIV1 Envelope glycoprotein gp160 OS=Human immunodeficiency virus 1 OX=11676 GN=env PE=3 SV=1
2::A0A1D9IZ81 **Mass:** 98634 **Score:** 58 **Matches:** 1(1) **Sequences:** 1(1)
>tr|A0A1D9IZ81|A0A1D9IZ81_9HIV1 Envelope glycoprotein gp160 OS=Human immunodeficiency virus 1 OX=11676 GN=env PE=3 SV=1
2::A0A1D9IZ82 **Mass:** 98575 **Score:** 58 **Matches:** 1(1) **Sequences:** 1(1)
>tr|A0A1D9IZ82|A0A1D9IZ82_9HIV1 Envelope glycoprotein gp160 OS=Human immunodeficiency virus 1 OX=11676 GN=env PE=3 SV=1
2::A0A1D9IZ83 **Mass:** 98639 **Score:** 58 **Matches:** 1(1) **Sequences:** 1(1)
>tr|A0A1D9IZ83|A0A1D9IZ83_9HIV1 Envelope glycoprotein gp160 OS=Human immunodeficiency virus 1 OX=11676 GN=env PE=3 SV=1
2::A0A1D9IZ85 **Mass:** 98649 **Score:** 58 **Matches:** 1(1) **Sequences:** 1(1)
>tr|A0A1D9IZ85|A0A1D9IZ85_9HIV1 Envelope glycoprotein gp160 OS=Human immunodeficiency virus 1 OX=11676 GN=env PE=3 SV=1
2::A0A1D9IZ86 **Mass:** 98595 **Score:** 58 **Matches:** 1(1) **Sequences:** 1(1)
>tr|A0A1D9IZ86|A0A1D9IZ86_9HIV1 Envelope glycoprotein gp160 OS=Human immunodeficiency virus 1 OX=11676 GN=env PE=3 SV=1
2::A0A1D9IZ87 **Mass:** 98594 **Score:** 58 **Matches:** 1(1) **Sequences:** 1(1)
>tr|A0A1D9IZ87|A0A1D9IZ87_9HIV1 Envelope glycoprotein gp160 OS=Human immunodeficiency virus 1 OX=11676 GN=env PE=3 SV=1
2::A0A1D9IZ90 **Mass:** 98661 **Score:** 58 **Matches:** 1(1) **Sequences:** 1(1)
>tr|A0A1D9IZ90|A0A1D9IZ90_9HIV1 Envelope glycoprotein gp160 OS=Human immunodeficiency virus 1 OX=11676 GN=env PE=3 SV=1
2::A0A1D9IZ91 **Mass:** 98664 **Score:** 58 **Matches:** 1(1) **Sequences:** 1(1)
>tr|A0A1D9IZ91|A0A1D9IZ91_9HIV1 Envelope glycoprotein gp160 OS=Human immunodeficiency virus 1 OX=11676 GN=env PE=3 SV=1
2::A0A1D9IZ92 **Mass:** 98742 **Score:** 58 **Matches:** 1(1) **Sequences:** 1(1)
>tr|A0A1D9IZ92|A0A1D9IZ92_9HIV1 Envelope glycoprotein gp160 OS=Human immunodeficiency virus 1 OX=11676 GN=env PE=3 SV=1
2::A0A1D9IZ94 **Mass:** 98379 **Score:** 58 **Matches:** 1(1) **Sequences:** 1(1)
>tr|A0A1D9IZ94|A0A1D9IZ94_9HIV1 Envelope glycoprotein gp160 OS=Human immunodeficiency virus 1 OX=11676 GN=env PE=3 SV=1
2::A0A1D9IZ95 **Mass:** 98574 **Score:** 58 **Matches:** 1(1) **Sequences:** 1(1)
>tr|A0A1D9IZ95|A0A1D9IZ95_9HIV1 Envelope glycoprotein gp160 OS=Human immunodeficiency virus 1 OX=11676 GN=env PE=3 SV=1
2::A0A1D9IZ98 **Mass:** 98669 **Score:** 58 **Matches:** 1(1) **Sequences:** 1(1)
>tr|A0A1D9IZ98|A0A1D9IZ98_9HIV1 Envelope glycoprotein gp160 OS=Human immunodeficiency virus 1 OX=11676 GN=env PE=3 SV=1
2::A0A1D9IZ99 **Mass:** 98247 **Score:** 58 **Matches:** 1(1) **Sequences:** 1(1)
>tr|A0A1D9IZ99|A0A1D9IZ99_9HIV1 Envelope glycoprotein gp160 OS=Human immunodeficiency virus 1 OX=11676 GN=env PE=3 SV=1
2::A0A1D9IZA0 **Mass:** 98717 **Score:** 58 **Matches:** 1(1) **Sequences:** 1(1)
>tr|A0A1D9IZA0|A0A1D9IZA0_9HIV1 Envelope glycoprotein gp160 OS=Human immunodeficiency virus 1 OX=11676 GN=env PE=3 SV=1
2::A0A1D9IZA2 **Mass:** 98657 **Score:** 58 **Matches:** 1(1) **Sequences:** 1(1)
>tr|A0A1D9IZA2|A0A1D9IZA2_9HIV1 Envelope glycoprotein gp160 OS=Human immunodeficiency virus 1 OX=11676 GN=env PE=3 SV=1
2::A0A1D9IZA4 **Mass:** 98652 **Score:** 58 **Matches:** 1(1) **Sequences:** 1(1)
>tr|A0A1D9IZA4|A0A1D9IZA4_9HIV1 Envelope glycoprotein gp160 OS=Human immunodeficiency virus 1 OX=11676 GN=env PE=3 SV=1
2::A0A1D9IZA5 **Mass:** 98641 **Score:** 58 **Matches:** 1(1) **Sequences:** 1(1)
>tr|A0A1D9IZA5|A0A1D9IZA5_9HIV1 Envelope glycoprotein gp160 OS=Human immunodeficiency virus 1 OX=11676 GN=env PE=3 SV=1
2::A0A1D9IZA7 **Mass:** 98448 **Score:** 58 **Matches:** 1(1) **Sequences:** 1(1)
>tr|A0A1D9IZA7|A0A1D9IZA7_9HIV1 Envelope glycoprotein gp160 OS=Human immunodeficiency virus 1 OX=11676 GN=env PE=3 SV=1
2::A0A1D9IZA9 **Mass:** 98703 **Score:** 58 **Matches:** 1(1) **Sequences:** 1(1)
>tr|A0A1D9IZA9|A0A1D9IZA9_9HIV1 Envelope glycoprotein gp160 OS=Human immunodeficiency virus 1 OX=11676 GN=env PE=3 SV=1
2::A0A1D9IZB2 **Mass:** 98568 **Score:** 58 **Matches:** 1(1) **Sequences:** 1(1)
>tr|A0A1D9IZB2|A0A1D9IZB2_9HIV1 Envelope glycoprotein gp160 OS=Human immunodeficiency virus 1 OX=11676 GN=env PE=3 SV=1
2::A0A1D9IZB8 **Mass:** 98650 **Score:** 58 **Matches:** 1(1) **Sequences:** 1(1)
>tr|A0A1D9IZB8|A0A1D9IZB8_9HIV1 Envelope glycoprotein gp160 OS=Human immunodeficiency virus 1 OX=11676 GN=env PE=3 SV=1
2::A0A1D9IZC7 **Mass:** 98673 **Score:** 58 **Matches:** 1(1) **Sequences:** 1(1)
>tr|A0A1D9IZC7|A0A1D9IZC7_9HIV1 Envelope glycoprotein gp160 OS=Human immunodeficiency virus 1 OX=11676 GN=env PE=3 SV=1
2::A0A1I9WB21 **Mass:** 97906 **Score:** 58 **Matches:** 1(1) **Sequences:** 1(1)
>tr|A0A1I9WB21|A0A1I9WB21_9HIV1 Envelope glycoprotein gp160 OS=Human immunodeficiency virus 1 OX=11676 GN=env PE=3 SV=1
2::A0A1I9WBC8 **Mass:** 97457 **Score:** 58 **Matches:** 1(1) **Sequences:** 1(1)
>tr|A0A1I9WBC8|A0A1I9WBC8_9HIV1 Envelope glycoprotein gp160 OS=Human immunodeficiency virus 1 OX=11676 GN=env PE=3 SV=1
2::A0A1I9WBC9 **Mass:** 97443 **Score:** 58 **Matches:** 1(1) **Sequences:** 1(1)
>tr|A0A1I9WBC9|A0A1I9WBC9_9HIV1 Envelope glycoprotein gp160 OS=Human immunodeficiency virus 1 OX=11676 GN=env PE=3 SV=1
2::A0A1I9WBD0 **Mass:** 85945 **Score:** 58 **Matches:** 1(1) **Sequences:** 1(1)
>tr|A0A1I9WBD0|A0A1I9WBD0_9HIV1 Envelope glycoprotein gp160 OS=Human immunodeficiency virus 1 OX=11676 GN=env PE=4 SV=1
2::A0A1I9WBD1 **Mass:** 85951 **Score:** 58 **Matches:** 1(1) **Sequences:** 1(1)
>tr|A0A1I9WBD1|A0A1I9WBD1_9HIV1 Envelope glycoprotein gp160 OS=Human immunodeficiency virus 1 OX=11676 GN=env PE=4 SV=1
2::A0A1L4ANE7 **Mass:** 99593 **Score:** 58 **Matches:** 1(1) **Sequences:** 1(1)
>tr|A0A1L4ANE7|A0A1L4ANE7_9HIV1 Envelope glycoprotein gp160 OS=Human immunodeficiency virus 1 OX=11676 GN=env PE=3 SV=1
2::A0A1L4CSI4 **Mass:** 99788 **Score:** 58 **Matches:** 1(1) **Sequences:** 1(1)
>tr|A0A1L4CSI4|A0A1L4CSI4_9HIV1 Envelope glycoprotein gp160 OS=Human immunodeficiency virus 1 OX=11676 GN=env PE=3 SV=1
2::A0A1L4CSJ4 **Mass:** 98219 **Score:** 58 **Matches:** 1(1) **Sequences:** 1(1)
>tr|A0A1L4CSJ4|A0A1L4CSJ4_9HIV1 Envelope glycoprotein gp160 OS=Human immunodeficiency virus 1 OX=11676 GN=env PE=3 SV=1
2::A0A1L7DTK2 **Mass:** 98641 **Score:** 58 **Matches:** 1(1) **Sequences:** 1(1)
>tr|A0A1L7DTK2|A0A1L7DTK2_9HIV1 Envelope glycoprotein gp160 OS=Human immunodeficiency virus 1 OX=11676 GN=env PE=3 SV=1
2::A0A1L7DTN4 **Mass:** 98753 **Score:** 58 **Matches:** 1(1) **Sequences:** 1(1)
>tr|A0A1L7DTN4|A0A1L7DTN4_9HIV1 Envelope glycoprotein gp160 OS=Human immunodeficiency virus 1 OX=11676 GN=env PE=3 SV=1
2::A0A1L7DTQ5 **Mass:** 97449 **Score:** 58 **Matches:** 1(1) **Sequences:** 1(1)
>tr|A0A1L7DTQ5|A0A1L7DTQ5_9HIV1 Envelope glycoprotein gp160 OS=Human immunodeficiency virus 1 OX=11676 GN=env PE=3 SV=1
2::A0A1L7DTR0 **Mass:** 97336 **Score:** 58 **Matches:** 1(1) **Sequences:** 1(1)
>tr|A0A1L7DTR0|A0A1L7DTR0_9HIV1 Envelope glycoprotein gp160 OS=Human immunodeficiency virus 1 OX=11676 GN=env PE=3 SV=1
2::A0A1L7DTR1 **Mass:** 97101 **Score:** 58 **Matches:** 1(1) **Sequences:** 1(1)
>tr|A0A1L7DTR1|A0A1L7DTR1_9HIV1 Envelope glycoprotein gp160 OS=Human immunodeficiency virus 1 OX=11676 GN=env PE=3 SV=1
2::A0A1L7DTT0 **Mass:** 97230 **Score:** 58 **Matches:** 1(1) **Sequences:** 1(1)
>tr|A0A1L7DTT0|A0A1L7DTT0_9HIV1 Envelope glycoprotein gp160 OS=Human immunodeficiency virus 1 OX=11676 GN=env PE=3 SV=1
2::A0A1L7DTT1 **Mass:** 97312 **Score:** 58 **Matches:** 1(1) **Sequences:** 1(1)
>tr|A0A1L7DTT1|A0A1L7DTT1_9HIV1 Envelope glycoprotein gp160 OS=Human immunodeficiency virus 1 OX=11676 GN=env PE=3 SV=1
2::A0A1L7DTT6 **Mass:** 97159 **Score:** 58 **Matches:** 1(1) **Sequences:** 1(1)
>tr|A0A1L7DTT6|A0A1L7DTT6_9HIV1 Envelope glycoprotein gp160 OS=Human immunodeficiency virus 1 OX=11676 GN=env PE=3 SV=1
2::A0A1L7DTU3 **Mass:** 97350 **Score:** 58 **Matches:** 1(1) **Sequences:** 1(1)
>tr|A0A1L7DTU3|A0A1L7DTU3_9HIV1 Envelope glycoprotein gp160 OS=Human immunodeficiency virus 1 OX=11676 GN=env PE=3 SV=1
2::A0A1L7DTV1 **Mass:** 98005 **Score:** 58 **Matches:** 1(1) **Sequences:** 1(1)
>tr|A0A1L7DTV1|A0A1L7DTV1_9HIV1 Envelope glycoprotein gp160 OS=Human immunodeficiency virus 1 OX=11676 GN=env PE=3 SV=1
2::A0A1L7DTW5 **Mass:** 97407 **Score:** 58 **Matches:** 1(1) **Sequences:** 1(1)
>tr|A0A1L7DTW5|A0A1L7DTW5_9HIV1 Envelope glycoprotein gp160 OS=Human immunodeficiency virus 1 OX=11676 GN=env PE=3 SV=1
2::A0A1L7DTW9 **Mass:** 98242 **Score:** 58 **Matches:** 1(1) **Sequences:** 1(1)
>tr|A0A1L7DTW9|A0A1L7DTW9_9HIV1 Envelope glycoprotein gp160 OS=Human immunodeficiency virus 1 OX=11676 GN=env PE=3 SV=1
2::A0A1L7DTZ7 **Mass:** 97121 **Score:** 58 **Matches:** 1(1) **Sequences:** 1(1)
>tr|A0A1L7DTZ7|A0A1L7DTZ7_9HIV1 Envelope glycoprotein gp160 OS=Human immunodeficiency virus 1 OX=11676 GN=env PE=3 SV=1
2::A0A1L7E2G1 **Mass:** 98838 **Score:** 58 **Matches:** 1(1) **Sequences:** 1(1)
>tr|A0A1L7E2G1|A0A1L7E2G1_9HIV1 Envelope glycoprotein gp160 OS=Human immunodeficiency virus 1 OX=11676 GN=env PE=3 SV=1
2::A0A1L7E2J0 **Mass:** 98804 **Score:** 58 **Matches:** 1(1) **Sequences:** 1(1)
>tr|A0A1L7E2J0|A0A1L7E2J0_9HIV1 Envelope glycoprotein gp160 OS=Human immunodeficiency virus 1 OX=11676 GN=env PE=3 SV=1
2::A0A1L7E2J3 **Mass:** 97489 **Score:** 58 **Matches:** 1(1) **Sequences:** 1(1)
>tr|A0A1L7E2J3|A0A1L7E2J3_9HIV1 Envelope glycoprotein gp160 OS=Human immunodeficiency virus 1 OX=11676 GN=env PE=3 SV=1
2::A0A1L7E2K1 **Mass:** 97445 **Score:** 58 **Matches:** 1(1) **Sequences:** 1(1)
>tr|A0A1L7E2K1|A0A1L7E2K1_9HIV1 Envelope glycoprotein gp160 OS=Human immunodeficiency virus 1 OX=11676 GN=env PE=3 SV=1
2::A0A1L7E2N1 **Mass:** 98063 **Score:** 58 **Matches:** 1(1) **Sequences:** 1(1)
>tr|A0A1L7E2N1|A0A1L7E2N1_9HIV1 Envelope glycoprotein gp160 OS=Human immunodeficiency virus 1 OX=11676 GN=env PE=3 SV=1
2::A0A1L7E2N4 **Mass:** 96921 **Score:** 58 **Matches:** 1(1) **Sequences:** 1(1)
>tr|A0A1L7E2N4|A0A1L7E2N4_9HIV1 Envelope glycoprotein gp160 OS=Human immunodeficiency virus 1 OX=11676 GN=env PE=3 SV=1
2::A0A1L7E2N6 **Mass:** 97315 **Score:** 58 **Matches:** 1(1) **Sequences:** 1(1)
>tr|A0A1L7E2N6|A0A1L7E2N6_9HIV1 Envelope glycoprotein gp160 OS=Human immunodeficiency virus 1 OX=11676 GN=env PE=3 SV=1
2::A0A1L7E2N7 **Mass:** 97135 **Score:** 58 **Matches:** 1(1) **Sequences:** 1(1)
>tr|A0A1L7E2N7|A0A1L7E2N7_9HIV1 Envelope glycoprotein gp160 OS=Human immunodeficiency virus 1 OX=11676 GN=env PE=3 SV=1

2::A0A1L7E2S0 **Mass:** 98324 **Score:** 58 **Matches:** 1(1) **Sequences:** 1(1)
>tr|A0A1L7E2S0|A0A1L7E2S0_9HIV1 Envelope glycoprotein gp160 OS=Human immunodeficiency virus 1 OX=11676 GN=env PE=3 SV=1
2::A0A1L7E2S1 **Mass:** 98492 **Score:** 58 **Matches:** 1(1) **Sequences:** 1(1)
>tr|A0A1L7E2S1|A0A1L7E2S1_9HIV1 Envelope glycoprotein gp160 OS=Human immunodeficiency virus 1 OX=11676 GN=env PE=3 SV=1
2::A0A1L7E2S4 **Mass:** 98732 **Score:** 58 **Matches:** 1(1) **Sequences:** 1(1)
>tr|A0A1L7E2S4|A0A1L7E2S4_9HIV1 Envelope glycoprotein gp160 OS=Human immunodeficiency virus 1 OX=11676 GN=env PE=3 SV=1
2::A0A1L7E2S6 **Mass:** 97731 **Score:** 58 **Matches:** 1(1) **Sequences:** 1(1)
>tr|A0A1L7E2S6|A0A1L7E2S6_9HIV1 Envelope glycoprotein gp160 OS=Human immunodeficiency virus 1 OX=11676 GN=env PE=3 SV=1
2::A0A1L7E2S8 **Mass:** 97204 **Score:** 58 **Matches:** 1(1) **Sequences:** 1(1)
>tr|A0A1L7E2S8|A0A1L7E2S8_9HIV1 Envelope glycoprotein gp160 OS=Human immunodeficiency virus 1 OX=11676 GN=env PE=3 SV=1
2::A0A1L7E2V7 **Mass:** 98062 **Score:** 58 **Matches:** 1(1) **Sequences:** 1(1)
>tr|A0A1L7E2V7|A0A1L7E2V7_9HIV1 Envelope glycoprotein gp160 OS=Human immunodeficiency virus 1 OX=11676 GN=env PE=3 SV=1
2::A0A1L7E2W1 **Mass:** 98588 **Score:** 58 **Matches:** 1(1) **Sequences:** 1(1)
>tr|A0A1L7E2W1|A0A1L7E2W1_9HIV1 Envelope glycoprotein gp160 OS=Human immunodeficiency virus 1 OX=11676 GN=env PE=3 SV=1
2::A0A1L7E2W6 **Mass:** 97386 **Score:** 58 **Matches:** 1(1) **Sequences:** 1(1)
>tr|A0A1L7E2W6|A0A1L7E2W6_9HIV1 Envelope glycoprotein gp160 OS=Human immunodeficiency virus 1 OX=11676 GN=env PE=3 SV=1
2::A0A1L7E2X2 **Mass:** 97141 **Score:** 58 **Matches:** 1(1) **Sequences:** 1(1)
>tr|A0A1L7E2X2|A0A1L7E2X2_9HIV1 Envelope glycoprotein gp160 OS=Human immunodeficiency virus 1 OX=11676 GN=env PE=3 SV=1
2::A0A1L7E310 **Mass:** 98418 **Score:** 58 **Matches:** 1(1) **Sequences:** 1(1)
>tr|A0A1L7E310|A0A1L7E310_9HIV1 Envelope glycoprotein gp160 OS=Human immunodeficiency virus 1 OX=11676 GN=env PE=3 SV=1
2::A0A1L7E339 **Mass:** 96909 **Score:** 58 **Matches:** 1(1) **Sequences:** 1(1)
>tr|A0A1L7E339|A0A1L7E339_9HIV1 Envelope glycoprotein gp160 OS=Human immunodeficiency virus 1 OX=11676 GN=env PE=3 SV=1
2::A0A1L7E357 **Mass:** 98135 **Score:** 58 **Matches:** 1(1) **Sequences:** 1(1)
>tr|A0A1L7E357|A0A1L7E357_9HIV1 Envelope glycoprotein gp160 OS=Human immunodeficiency virus 1 OX=11676 GN=env PE=3 SV=1
2::A0A1L7E3A3 **Mass:** 97323 **Score:** 58 **Matches:** 1(1) **Sequences:** 1(1)
>tr|A0A1L7E3A3|A0A1L7E3A3_9HIV1 Envelope glycoprotein gp160 OS=Human immunodeficiency virus 1 OX=11676 GN=env PE=3 SV=1
2::A0A1P8DG81 **Mass:** 97859 **Score:** 58 **Matches:** 1(1) **Sequences:** 1(1)
>tr|A0A1P8DG81|A0A1P8DG81_9HIV1 Envelope glycoprotein gp160 OS=Human immunodeficiency virus 1 OX=11676 GN=env PE=3 SV=1
2::A0A1P8DGJ1 **Mass:** 99267 **Score:** 58 **Matches:** 1(1) **Sequences:** 1(1)
>tr|A0A1P8DGJ1|A0A1P8DGJ1_9HIV1 Envelope glycoprotein gp160 OS=Human immunodeficiency virus 1 OX=11676 GN=env PE=3 SV=1
2::A0A1P8DGS2 **Mass:** 98393 **Score:** 58 **Matches:** 1(1) **Sequences:** 1(1)
>tr|A0A1P8DGS2|A0A1P8DGS2_9HIV1 Envelope glycoprotein gp160 OS=Human immunodeficiency virus 1 OX=11676 GN=env PE=3 SV=1
2::A0A1P8DHU0 **Mass:** 97753 **Score:** 58 **Matches:** 1(1) **Sequences:** 1(1)
>tr|A0A1P8DHU0|A0A1P8DHU0_9HIV1 Envelope glycoprotein gp160 OS=Human immunodeficiency virus 1 OX=11676 GN=env PE=3 SV=1
2::A0A1Q1G4P2 **Mass:** 98780 **Score:** 58 **Matches:** 1(1) **Sequences:** 1(1)
>tr|A0A1Q1G4P2|A0A1Q1G4P2_9HIV1 Envelope glycoprotein gp160 OS=Human immunodeficiency virus 1 OX=11676 GN=env PE=3 SV=1
2::A0A1V0CQA4 **Mass:** 97742 **Score:** 58 **Matches:** 1(1) **Sequences:** 1(1)
>tr|A0A1V0CQA4|A0A1V0CQA4_9HIV1 Envelope glycoprotein gp160 OS=Human immunodeficiency virus 1 OX=11676 GN=env PE=3 SV=1
2::A0A1V0CQA9 **Mass:** 98415 **Score:** 58 **Matches:** 1(1) **Sequences:** 1(1)
>tr|A0A1V0CQA9|A0A1V0CQA9_9HIV1 Envelope glycoprotein gp160 OS=Human immunodeficiency virus 1 OX=11676 GN=env PE=3 SV=1
2::A0A218MY24 **Mass:** 97327 **Score:** 58 **Matches:** 1(1) **Sequences:** 1(1)
>tr|A0A218MY24|A0A218MY24_9HIV1 Envelope glycoprotein gp160 OS=Human immunodeficiency virus 1 OX=11676 GN=env PE=3 SV=1
2::A0A218MY28 **Mass:** 97340 **Score:** 58 **Matches:** 1(1) **Sequences:** 1(1)
>tr|A0A218MY28|A0A218MY28_9HIV1 Envelope glycoprotein gp160 OS=Human immunodeficiency virus 1 OX=11676 GN=env PE=3 SV=1
2::A0A218MY29 **Mass:** 96889 **Score:** 58 **Matches:** 1(1) **Sequences:** 1(1)
>tr|A0A218MY29|A0A218MY29_9HIV1 Envelope glycoprotein gp160 OS=Human immunodeficiency virus 1 OX=11676 GN=env PE=3 SV=1
2::A0A218MY30 **Mass:** 96960 **Score:** 58 **Matches:** 1(1) **Sequences:** 1(1)
>tr|A0A218MY30|A0A218MY30_9HIV1 Envelope glycoprotein gp160 OS=Human immunodeficiency virus 1 OX=11676 GN=env PE=3 SV=1
2::A0A218MY31 **Mass:** 98048 **Score:** 58 **Matches:** 1(1) **Sequences:** 1(1)
>tr|A0A218MY31|A0A218MY31_9HIV1 Envelope glycoprotein gp160 OS=Human immunodeficiency virus 1 OX=11676 GN=env PE=3 SV=1
2::A0A218MY32 **Mass:** 97804 **Score:** 58 **Matches:** 1(1) **Sequences:** 1(1)
>tr|A0A218MY32|A0A218MY32_9HIV1 Envelope glycoprotein gp160 OS=Human immunodeficiency virus 1 OX=11676 GN=env PE=3 SV=1
2::A0A218MY33 **Mass:** 98075 **Score:** 58 **Matches:** 1(1) **Sequences:** 1(1)
>tr|A0A218MY33|A0A218MY33_9HIV1 Envelope glycoprotein gp160 OS=Human immunodeficiency virus 1 OX=11676 GN=env PE=3 SV=1
2::A0A218MY34 **Mass:** 97977 **Score:** 58 **Matches:** 1(1) **Sequences:** 1(1)
>tr|A0A218MY34|A0A218MY34_9HIV1 Envelope glycoprotein gp160 OS=Human immunodeficiency virus 1 OX=11676 GN=env PE=3 SV=1
2::A0A218MY35 **Mass:** 96862 **Score:** 58 **Matches:** 1(1) **Sequences:** 1(1)
>tr|A0A218MY35|A0A218MY35_9HIV1 Envelope glycoprotein gp160 OS=Human immunodeficiency virus 1 OX=11676 GN=env PE=3 SV=1
2::A0A218MY36 **Mass:** 97937 **Score:** 58 **Matches:** 1(1) **Sequences:** 1(1)
>tr|A0A218MY36|A0A218MY36_9HIV1 Envelope glycoprotein gp160 OS=Human immunodeficiency virus 1 OX=11676 GN=env PE=3 SV=1
2::A0A218MY38 **Mass:** 98401 **Score:** 58 **Matches:** 1(1) **Sequences:** 1(1)
>tr|A0A218MY38|A0A218MY38_9HIV1 Envelope glycoprotein gp160 OS=Human immunodeficiency virus 1 OX=11676 GN=env PE=3 SV=1
2::A0A218MY40 **Mass:** 97910 **Score:** 58 **Matches:** 1(1) **Sequences:** 1(1)
>tr|A0A218MY40|A0A218MY40_9HIV1 Envelope glycoprotein gp160 OS=Human immunodeficiency virus 1 OX=11676 GN=env PE=3 SV=1
2::A0A218MY42 **Mass:** 97777 **Score:** 58 **Matches:** 1(1) **Sequences:** 1(1)
>tr|A0A218MY42|A0A218MY42_9HIV1 Envelope glycoprotein gp160 OS=Human immunodeficiency virus 1 OX=11676 GN=env PE=3 SV=1
2::A0A218MY45 **Mass:** 98374 **Score:** 58 **Matches:** 1(1) **Sequences:** 1(1)
>tr|A0A218MY45|A0A218MY45_9HIV1 Envelope glycoprotein gp160 OS=Human immunodeficiency virus 1 OX=11676 GN=env PE=3 SV=1
2::A0A248SMG4 **Mass:** 28562 **Score:** 58 **Matches:** 1(1) **Sequences:** 1(1)
>tr|A0A248SMG4|A0A248SMG4_9HIV1 Envelope glycoprotein (Fragment) OS=Human immunodeficiency virus 1 OX=11676 GN=env PE=4 SV=1
2::A0A286LG32 **Mass:** 98289 **Score:** 58 **Matches:** 1(1) **Sequences:** 1(1)
>tr|A0A286LG32|A0A286LG32_9HIV1 Envelope glycoprotein gp160 OS=Human immunodeficiency virus 1 OX=11676 GN=env PE=3 SV=1
2::A0A286LGN8 **Mass:** 99031 **Score:** 58 **Matches:** 1(1) **Sequences:** 1(1)
>tr|A0A286LGN8|A0A286LGN8_9HIV1 Envelope glycoprotein gp160 OS=Human immunodeficiency virus 1 OX=11676 GN=env PE=3 SV=1
2::A0A286LGP7 **Mass:** 99561 **Score:** 58 **Matches:** 1(1) **Sequences:** 1(1)
>tr|A0A286LGP7|A0A286LGP7_9HIV1 Envelope glycoprotein gp160 OS=Human immunodeficiency virus 1 OX=11676 GN=env PE=3 SV=1
2::A0A286LGT3 **Mass:** 99231 **Score:** 58 **Matches:** 1(1) **Sequences:** 1(1)
>tr|A0A286LGT3|A0A286LGT3_9HIV1 Envelope glycoprotein gp160 OS=Human immunodeficiency virus 1 OX=11676 GN=env PE=3 SV=1
2::A0A286LHF4 **Mass:** 98164 **Score:** 58 **Matches:** 1(1) **Sequences:** 1(1)
>tr|A0A286LHF4|A0A286LHF4_9HIV1 Envelope glycoprotein gp160 OS=Human immunodeficiency virus 1 OX=11676 GN=env PE=3 SV=1
2::A0A286MFF6 **Mass:** 93919 **Score:** 58 **Matches:** 1(1) **Sequences:** 1(1)
>tr|A0A286MFF6|A0A286MFF6_9HIV1 Envelope glycoprotein gp160 (Fragment) OS=Human immunodeficiency virus 1 OX=11676 GN=env PE=4 SV=1
2::A0A286MFF9 **Mass:** 95470 **Score:** 58 **Matches:** 1(1) **Sequences:** 1(1)
>tr|A0A286MFF9|A0A286MFF9_9HIV1 Envelope glycoprotein gp160 (Fragment) OS=Human immunodeficiency virus 1 OX=11676 GN=env PE=3 SV=1
2::A0A286MFG4 **Mass:** 94941 **Score:** 58 **Matches:** 1(1) **Sequences:** 1(1)
>tr|A0A286MFG4|A0A286MFG4_9HIV1 Envelope glycoprotein gp160 (Fragment) OS=Human immunodeficiency virus 1 OX=11676 GN=env PE=4 SV=1
2::A0A286RRQ6 **Mass:** 92360 **Score:** 58 **Matches:** 1(1) **Sequences:** 1(1)
>tr|A0A286RRQ6|A0A286RRQ6_9HIV1 Envelope glycoprotein gp160 (Fragment) OS=Human immunodeficiency virus 1 OX=11676 GN=env PE=4 SV=1
2::A0A286RRQ8 **Mass:** 92517 **Score:** 58 **Matches:** 1(1) **Sequences:** 1(1)
>tr|A0A286RRQ8|A0A286RRQ8_9HIV1 Envelope glycoprotein gp160 (Fragment) OS=Human immunodeficiency virus 1 OX=11676 GN=env PE=4 SV=1
2::A0A286RRR7 **Mass:** 92354 **Score:** 58 **Matches:** 1(1) **Sequences:** 1(1)
>tr|A0A286RRR7|A0A286RRR7_9HIV1 Envelope glycoprotein gp160 (Fragment) OS=Human immunodeficiency virus 1 OX=11676 GN=env PE=4 SV=1
2::A0A286RRR9 **Mass:** 90802 **Score:** 58 **Matches:** 1(1) **Sequences:** 1(1)
>tr|A0A286RRR9|A0A286RRR9_9HIV1 Envelope glycoprotein gp160 (Fragment) OS=Human immunodeficiency virus 1 OX=11676 GN=env PE=4 SV=1
2::A0A286RRS0 **Mass:** 91227 **Score:** 58 **Matches:** 1(1) **Sequences:** 1(1)
>tr|A0A286RRS0|A0A286RRS0_9HIV1 Envelope glycoprotein gp160 (Fragment) OS=Human immunodeficiency virus 1 OX=11676 GN=env PE=4 SV=1
2::A0A286RRS1 **Mass:** 91184 **Score:** 58 **Matches:** 1(1) **Sequences:** 1(1)
>tr|A0A286RRS1|A0A286RRS1_9HIV1 Envelope glycoprotein gp160 (Fragment) OS=Human immunodeficiency virus 1 OX=11676 GN=env PE=4 SV=1
2::A0A286RRS3 **Mass:** 91257 **Score:** 58 **Matches:** 1(1) **Sequences:** 1(1)
>tr|A0A286RRS3|A0A286RRS3_9HIV1 Envelope glycoprotein gp160 (Fragment) OS=Human immunodeficiency virus 1 OX=11676 GN=env PE=4 SV=1
2::A0A286RRS6 **Mass:** 90535 **Score:** 58 **Matches:** 1(1) **Sequences:** 1(1)
>tr|A0A286RRS6|A0A286RRS6_9HIV1 Envelope glycoprotein gp160 (Fragment) OS=Human immunodeficiency virus 1 OX=11676 GN=env PE=4 SV=1
2::A0A286RRS7 **Mass:** 92386 **Score:** 58 **Matches:** 1(1) **Sequences:** 1(1)
>tr|A0A286RRS7|A0A286RRS7_9HIV1 Envelope glycoprotein gp160 (Fragment) OS=Human immunodeficiency virus 1 OX=11676 GN=env PE=4 SV=1
2::A0A286RRS8 **Mass:** 91153 **Score:** 58 **Matches:** 1(1) **Sequences:** 1(1)
>tr|A0A286RRS8|A0A286RRS8_9HIV1 Envelope glycoprotein gp160 (Fragment) OS=Human immunodeficiency virus 1 OX=11676 GN=env PE=4 SV=1
2::A0A286RRS9 **Mass:** 91142 **Score:** 58 **Matches:** 1(1) **Sequences:** 1(1)
>tr|A0A286RRS9|A0A286RRS9_9HIV1 Envelope glycoprotein gp160 (Fragment) OS=Human immunodeficiency virus 1 OX=11676 GN=env PE=4 SV=1
2::A0A286RRT0 **Mass:** 91145 **Score:** 58 **Matches:** 1(1) **Sequences:** 1(1)
>tr|A0A286RRT0|A0A286RRT0_9HIV1 Envelope glycoprotein gp160 (Fragment) OS=Human immunodeficiency virus 1 OX=11676 GN=env PE=4 SV=1

2::A0A286RRT1 **Mass:** 92325 **Score:** 58 **Matches:** 1(1) **Sequences:** 1(1)
>tr|A0A286RRT1|A0A286RRT1_9HIV1 Envelope glycoprotein gp160 (Fragment) OS=Human immunodeficiency virus 1 OX=11676 GN=env PE=4 SV=1
2::A0A286RRT2 **Mass:** 92353 **Score:** 58 **Matches:** 1(1) **Sequences:** 1(1)
>tr|A0A286RRT2|A0A286RRT2_9HIV1 Envelope glycoprotein gp160 (Fragment) OS=Human immunodeficiency virus 1 OX=11676 GN=env PE=4 SV=1
2::A0A286RRT3 **Mass:** 92343 **Score:** 58 **Matches:** 1(1) **Sequences:** 1(1)
>tr|A0A286RRT3|A0A286RRT3_9HIV1 Envelope glycoprotein gp160 (Fragment) OS=Human immunodeficiency virus 1 OX=11676 GN=env PE=4 SV=1
2::A0A286RRT4 **Mass:** 91160 **Score:** 58 **Matches:** 1(1) **Sequences:** 1(1)
>tr|A0A286RRT4|A0A286RRT4_9HIV1 Envelope glycoprotein gp160 (Fragment) OS=Human immunodeficiency virus 1 OX=11676 GN=env PE=4 SV=1
2::A0A286RRT5 **Mass:** 91256 **Score:** 58 **Matches:** 1(1) **Sequences:** 1(1)
>tr|A0A286RRT5|A0A286RRT5_9HIV1 Envelope glycoprotein gp160 (Fragment) OS=Human immunodeficiency virus 1 OX=11676 GN=env PE=4 SV=1
2::A0A286RRT6 **Mass:** 92359 **Score:** 58 **Matches:** 1(1) **Sequences:** 1(1)
>tr|A0A286RRT6|A0A286RRT6_9HIV1 Envelope glycoprotein gp160 (Fragment) OS=Human immunodeficiency virus 1 OX=11676 GN=env PE=4 SV=1
2::A0A286RRT7 **Mass:** 92384 **Score:** 58 **Matches:** 1(1) **Sequences:** 1(1)
>tr|A0A286RRT7|A0A286RRT7_9HIV1 Envelope glycoprotein gp160 (Fragment) OS=Human immunodeficiency virus 1 OX=11676 GN=env PE=4 SV=1
2::A0A286RRT9 **Mass:** 91173 **Score:** 58 **Matches:** 1(1) **Sequences:** 1(1)
>tr|A0A286RRT9|A0A286RRT9_9HIV1 Envelope glycoprotein gp160 (Fragment) OS=Human immunodeficiency virus 1 OX=11676 GN=env PE=4 SV=1
2::A0A286RRU0 **Mass:** 91037 **Score:** 58 **Matches:** 1(1) **Sequences:** 1(1)
>tr|A0A286RRU0|A0A286RRU0_9HIV1 Envelope glycoprotein gp160 (Fragment) OS=Human immunodeficiency virus 1 OX=11676 GN=env PE=4 SV=1
2::A0A286RRU1 **Mass:** 92385 **Score:** 58 **Matches:** 1(1) **Sequences:** 1(1)
>tr|A0A286RRU1|A0A286RRU1_9HIV1 Envelope glycoprotein gp160 (Fragment) OS=Human immunodeficiency virus 1 OX=11676 GN=env PE=4 SV=1
2::A0A286RRU2 **Mass:** 92018 **Score:** 58 **Matches:** 1(1) **Sequences:** 1(1)
>tr|A0A286RRU2|A0A286RRU2_9HIV1 Envelope glycoprotein gp160 (Fragment) OS=Human immunodeficiency virus 1 OX=11676 GN=env PE=4 SV=1
2::A0A286RRU3 **Mass:** 91256 **Score:** 58 **Matches:** 1(1) **Sequences:** 1(1)
>tr|A0A286RRU3|A0A286RRU3_9HIV1 Envelope glycoprotein gp160 (Fragment) OS=Human immunodeficiency virus 1 OX=11676 GN=env PE=4 SV=1
2::A0A286RRU4 **Mass:** 91244 **Score:** 58 **Matches:** 1(1) **Sequences:** 1(1)
>tr|A0A286RRU4|A0A286RRU4_9HIV1 Envelope glycoprotein gp160 (Fragment) OS=Human immunodeficiency virus 1 OX=11676 GN=env PE=4 SV=1
2::A0A286RRU5 **Mass:** 91198 **Score:** 58 **Matches:** 1(1) **Sequences:** 1(1)
>tr|A0A286RRU5|A0A286RRU5_9HIV1 Envelope glycoprotein gp160 (Fragment) OS=Human immunodeficiency virus 1 OX=11676 GN=env PE=4 SV=1
2::A0A286RRU6 **Mass:** 91183 **Score:** 58 **Matches:** 1(1) **Sequences:** 1(1)
>tr|A0A286RRU6|A0A286RRU6_9HIV1 Envelope glycoprotein gp160 (Fragment) OS=Human immunodeficiency virus 1 OX=11676 GN=env PE=4 SV=1
2::A0A286RRU7 **Mass:** 91166 **Score:** 58 **Matches:** 1(1) **Sequences:** 1(1)
>tr|A0A286RRU7|A0A286RRU7_9HIV1 Envelope glycoprotein gp160 (Fragment) OS=Human immunodeficiency virus 1 OX=11676 GN=env PE=4 SV=1
2::A0A286RRU8 **Mass:** 91198 **Score:** 58 **Matches:** 1(1) **Sequences:** 1(1)
>tr|A0A286RRU8|A0A286RRU8_9HIV1 Envelope glycoprotein gp160 (Fragment) OS=Human immunodeficiency virus 1 OX=11676 GN=env PE=4 SV=1
2::A0A286RRV0 **Mass:** 91110 **Score:** 58 **Matches:** 1(1) **Sequences:** 1(1)
>tr|A0A286RRV0|A0A286RRV0_9HIV1 Envelope glycoprotein gp160 (Fragment) OS=Human immunodeficiency virus 1 OX=11676 GN=env PE=4 SV=1
2::A0A286RRV1 **Mass:** 92303 **Score:** 58 **Matches:** 1(1) **Sequences:** 1(1)
>tr|A0A286RRV1|A0A286RRV1_9HIV1 Envelope glycoprotein gp160 (Fragment) OS=Human immunodeficiency virus 1 OX=11676 GN=env PE=4 SV=1
2::A0A286RRV4 **Mass:** 91172 **Score:** 58 **Matches:** 1(1) **Sequences:** 1(1)
>tr|A0A286RRV4|A0A286RRV4_9HIV1 Envelope glycoprotein gp160 (Fragment) OS=Human immunodeficiency virus 1 OX=11676 GN=env PE=4 SV=1
2::A0A286RRV6 **Mass:** 91226 **Score:** 58 **Matches:** 1(1) **Sequences:** 1(1)
>tr|A0A286RRV6|A0A286RRV6_9HIV1 Envelope glycoprotein gp160 (Fragment) OS=Human immunodeficiency virus 1 OX=11676 GN=env PE=4 SV=1
2::A0A286RRV8 **Mass:** 91041 **Score:** 58 **Matches:** 1(1) **Sequences:** 1(1)
>tr|A0A286RRV8|A0A286RRV8_9HIV1 Envelope glycoprotein gp160 (Fragment) OS=Human immunodeficiency virus 1 OX=11676 GN=env PE=4 SV=1
2::A0A2L1KX23 **Mass:** 93187 **Score:** 58 **Matches:** 1(1) **Sequences:** 1(1)
>tr|A0A2L1KX23|A0A2L1KX23_9HIV1 Envelope glycoprotein gp160 OS=Human immunodeficiency virus 1 OX=11676 GN=env PE=3 SV=1
2::A0A2L1KXU2 **Mass:** 92699 **Score:** 58 **Matches:** 1(1) **Sequences:** 1(1)
>tr|A0A2L1KXU2|A0A2L1KXU2_9HIV1 Envelope glycoprotein gp160 OS=Human immunodeficiency virus 1 OX=11676 GN=env PE=3 SV=1
2::A0A2S1CN40 **Mass:** 98269 **Score:** 58 **Matches:** 1(1) **Sequences:** 1(1)
>tr|A0A2S1CN40|A0A2S1CN40_9HIV1 Envelope glycoprotein gp160 OS=Human immunodeficiency virus 1 OX=11676 GN=env PE=3 SV=1
2::A0A2S1CN47 **Mass:** 97793 **Score:** 58 **Matches:** 1(1) **Sequences:** 1(1)
>tr|A0A2S1CN47|A0A2S1CN47_9HIV1 Envelope glycoprotein gp160 OS=Human immunodeficiency virus 1 OX=11676 GN=env PE=3 SV=1
2::A0A2S1CN48 **Mass:** 97782 **Score:** 58 **Matches:** 1(1) **Sequences:** 1(1)
>tr|A0A2S1CN48|A0A2S1CN48_9HIV1 Envelope glycoprotein gp160 OS=Human immunodeficiency virus 1 OX=11676 GN=env PE=3 SV=1
2::A0A2S1CN51 **Mass:** 97744 **Score:** 58 **Matches:** 1(1) **Sequences:** 1(1)
>tr|A0A2S1CN51|A0A2S1CN51_9HIV1 Envelope glycoprotein gp160 OS=Human immunodeficiency virus 1 OX=11676 GN=env PE=3 SV=1
2::A0A2S1CN55 **Mass:** 97733 **Score:** 58 **Matches:** 1(1) **Sequences:** 1(1)
>tr|A0A2S1CN55|A0A2S1CN55_9HIV1 Envelope glycoprotein gp160 OS=Human immunodeficiency virus 1 OX=11676 GN=env PE=3 SV=1
2::A0A2S1CN58 **Mass:** 97870 **Score:** 58 **Matches:** 1(1) **Sequences:** 1(1)
>tr|A0A2S1CN58|A0A2S1CN58_9HIV1 Envelope glycoprotein gp160 OS=Human immunodeficiency virus 1 OX=11676 GN=env PE=3 SV=1
2::A0A2S1CN59 **Mass:** 97817 **Score:** 58 **Matches:** 1(1) **Sequences:** 1(1)
>tr|A0A2S1CN59|A0A2S1CN59_9HIV1 Envelope glycoprotein gp160 OS=Human immunodeficiency virus 1 OX=11676 GN=env PE=3 SV=1
2::A0A2S1CN60 **Mass:** 97842 **Score:** 58 **Matches:** 1(1) **Sequences:** 1(1)
>tr|A0A2S1CN60|A0A2S1CN60_9HIV1 Envelope glycoprotein gp160 OS=Human immunodeficiency virus 1 OX=11676 GN=env PE=3 SV=1
2::A0A2S1CN61 **Mass:** 97730 **Score:** 58 **Matches:** 1(1) **Sequences:** 1(1)
>tr|A0A2S1CN61|A0A2S1CN61_9HIV1 Envelope glycoprotein gp160 OS=Human immunodeficiency virus 1 OX=11676 GN=env PE=3 SV=1
2::A0A2S1CN62 **Mass:** 97815 **Score:** 58 **Matches:** 1(1) **Sequences:** 1(1)
>tr|A0A2S1CN62|A0A2S1CN62_9HIV1 Envelope glycoprotein gp160 OS=Human immunodeficiency virus 1 OX=11676 GN=env PE=3 SV=1
2::A0A2S1CN63 **Mass:** 97783 **Score:** 58 **Matches:** 1(1) **Sequences:** 1(1)
>tr|A0A2S1CN63|A0A2S1CN63_9HIV1 Envelope glycoprotein gp160 OS=Human immunodeficiency virus 1 OX=11676 GN=env PE=3 SV=1
2::A0A2S1CN64 **Mass:** 97805 **Score:** 58 **Matches:** 1(1) **Sequences:** 1(1)
>tr|A0A2S1CN64|A0A2S1CN64_9HIV1 Envelope glycoprotein gp160 OS=Human immunodeficiency virus 1 OX=11676 GN=env PE=3 SV=1
2::A0A2S1CN66 **Mass:** 97856 **Score:** 58 **Matches:** 1(1) **Sequences:** 1(1)
>tr|A0A2S1CN66|A0A2S1CN66_9HIV1 Envelope glycoprotein gp160 OS=Human immunodeficiency virus 1 OX=11676 GN=env PE=3 SV=1
2::A0A2S1CN67 **Mass:** 97704 **Score:** 58 **Matches:** 1(1) **Sequences:** 1(1)
>tr|A0A2S1CN67|A0A2S1CN67_9HIV1 Envelope glycoprotein gp160 OS=Human immunodeficiency virus 1 OX=11676 GN=env PE=3 SV=1
2::A0A2S1CN68 **Mass:** 97726 **Score:** 58 **Matches:** 1(1) **Sequences:** 1(1)
>tr|A0A2S1CN68|A0A2S1CN68_9HIV1 Envelope glycoprotein gp160 OS=Human immunodeficiency virus 1 OX=11676 GN=env PE=3 SV=1
2::A0A2S1CN69 **Mass:** 98028 **Score:** 58 **Matches:** 1(1) **Sequences:** 1(1)
>tr|A0A2S1CN69|A0A2S1CN69_9HIV1 Envelope glycoprotein gp160 OS=Human immunodeficiency virus 1 OX=11676 GN=env PE=3 SV=1
2::A0A2S1CN71 **Mass:** 97619 **Score:** 58 **Matches:** 1(1) **Sequences:** 1(1)
>tr|A0A2S1CN71|A0A2S1CN71_9HIV1 Envelope glycoprotein gp160 OS=Human immunodeficiency virus 1 OX=11676 GN=env PE=3 SV=1
2::A0A2S1CN72 **Mass:** 97830 **Score:** 58 **Matches:** 1(1) **Sequences:** 1(1)
>tr|A0A2S1CN72|A0A2S1CN72_9HIV1 Envelope glycoprotein gp160 OS=Human immunodeficiency virus 1 OX=11676 GN=env PE=3 SV=1
2::A0A2S1CN73 **Mass:** 97865 **Score:** 58 **Matches:** 1(1) **Sequences:** 1(1)
>tr|A0A2S1CN73|A0A2S1CN73_9HIV1 Envelope glycoprotein gp160 OS=Human immunodeficiency virus 1 OX=11676 GN=env PE=3 SV=1
2::A0A2S1CN74 **Mass:** 98328 **Score:** 58 **Matches:** 1(1) **Sequences:** 1(1)
>tr|A0A2S1CN74|A0A2S1CN74_9HIV1 Envelope glycoprotein gp160 OS=Human immunodeficiency virus 1 OX=11676 GN=env PE=3 SV=1
2::A0A2S1CN75 **Mass:** 97793 **Score:** 58 **Matches:** 1(1) **Sequences:** 1(1)
>tr|A0A2S1CN75|A0A2S1CN75_9HIV1 Envelope glycoprotein gp160 OS=Human immunodeficiency virus 1 OX=11676 GN=env PE=3 SV=1
2::A0A2S1CN76 **Mass:** 97747 **Score:** 58 **Matches:** 1(1) **Sequences:** 1(1)
>tr|A0A2S1CN76|A0A2S1CN76_9HIV1 Envelope glycoprotein gp160 OS=Human immunodeficiency virus 1 OX=11676 GN=env PE=3 SV=1
2::A0A2S1CN79 **Mass:** 97745 **Score:** 58 **Matches:** 1(1) **Sequences:** 1(1)
>tr|A0A2S1CN79|A0A2S1CN79_9HIV1 Envelope glycoprotein gp160 OS=Human immunodeficiency virus 1 OX=11676 GN=env PE=3 SV=1
2::A0A2S1CN81 **Mass:** 97413 **Score:** 58 **Matches:** 1(1) **Sequences:** 1(1)
>tr|A0A2S1CN81|A0A2S1CN81_9HIV1 Envelope glycoprotein gp160 OS=Human immunodeficiency virus 1 OX=11676 GN=env PE=3 SV=1
2::A0A2S1CN82 **Mass:** 97671 **Score:** 58 **Matches:** 1(1) **Sequences:** 1(1)
>tr|A0A2S1CN82|A0A2S1CN82_9HIV1 Envelope glycoprotein gp160 OS=Human immunodeficiency virus 1 OX=11676 GN=env PE=3 SV=1
2::A0A2S1CN83 **Mass:** 97744 **Score:** 58 **Matches:** 1(1) **Sequences:** 1(1)
>tr|A0A2S1CN83|A0A2S1CN83_9HIV1 Envelope glycoprotein gp160 OS=Human immunodeficiency virus 1 OX=11676 GN=env PE=3 SV=1
2::A0A3G2KT52 **Mass:** 98832 **Score:** 58 **Matches:** 1(1) **Sequences:** 1(1)
>tr|A0A3G2KT52|A0A3G2KT52_9HIV1 Envelope glycoprotein gp160 OS=Human immunodeficiency virus 1 OX=11676 GN=env PE=3 SV=1
2::A0A3Q8Q0Z3 **Mass:** 96360 **Score:** 58 **Matches:** 1(1) **Sequences:** 1(1)
>tr|A0A3Q8Q0Z3|A0A3Q8Q0Z3_9HIV1 Envelope glycoprotein gp160 OS=Human immunodeficiency virus 1 OX=11676 GN=env PE=3 SV=1
2::A0A3Q8Q102 **Mass:** 96422 **Score:** 58 **Matches:** 1(1) **Sequences:** 1(1)
>tr|A0A3Q8Q102|A0A3Q8Q102_9HIV1 Envelope glycoprotein gp160 OS=Human immunodeficiency virus 1 OX=11676 GN=env PE=3 SV=1
2::A0A3Q8Q103 **Mass:** 96416 **Score:** 58 **Matches:** 1(1) **Sequences:** 1(1)
>tr|A0A3Q8Q103|A0A3Q8Q103_9HIV1 Envelope glycoprotein gp160 OS=Human immunodeficiency virus 1 OX=11676 GN=env PE=3 SV=1
2::A0A3Q8Q106 **Mass:** 96350 **Score:** 58 **Matches:** 1(1) **Sequences:** 1(1)
>tr|A0A3Q8Q106|A0A3Q8Q106_9HIV1 Envelope glycoprotein gp160 OS=Human immunodeficiency virus 1 OX=11676 GN=env PE=3 SV=1

2::A0A3Q8Q115 **Mass:** 92128 **Score:** 58 **Matches:** 1(1) **Sequences:** 1(1)
>tr|A0A3Q8Q115|A0A3Q8Q115_9HIV1 Envelope glycoprotein gp160 (Fragment) OS=Human immunodeficiency virus 1 OX=11676 GN=env PE=3 SV=1
2::A0A3Q8Q116 **Mass:** 96413 **Score:** 58 **Matches:** 1(1) **Sequences:** 1(1)
>tr|A0A3Q8Q116|A0A3Q8Q116_9HIV1 Envelope glycoprotein gp160 OS=Human immunodeficiency virus 1 OX=11676 GN=env PE=3 SV=1
2::A0A3Q8Q117 **Mass:** 96202 **Score:** 58 **Matches:** 1(1) **Sequences:** 1(1)
>tr|A0A3Q8Q117|A0A3Q8Q117_9HIV1 Envelope glycoprotein gp160 (Fragment) OS=Human immunodeficiency virus 1 OX=11676 GN=env PE=3 SV=1
2::A0A3Q8Q121 **Mass:** 96381 **Score:** 58 **Matches:** 1(1) **Sequences:** 1(1)
>tr|A0A3Q8Q121|A0A3Q8Q121_9HIV1 Envelope glycoprotein gp160 OS=Human immunodeficiency virus 1 OX=11676 GN=env PE=3 SV=1
2::A0A3Q8Q122 **Mass:** 96571 **Score:** 58 **Matches:** 1(1) **Sequences:** 1(1)
>tr|A0A3Q8Q122|A0A3Q8Q122_9HIV1 Envelope glycoprotein gp160 (Fragment) OS=Human immunodeficiency virus 1 OX=11676 GN=env PE=3 SV=1
2::A0A3Q8Q126 **Mass:** 96482 **Score:** 58 **Matches:** 1(1) **Sequences:** 1(1)
>tr|A0A3Q8Q126|A0A3Q8Q126_9HIV1 Envelope glycoprotein gp160 OS=Human immunodeficiency virus 1 OX=11676 GN=env PE=3 SV=1
2::A0A3Q8Q127 **Mass:** 96485 **Score:** 58 **Matches:** 1(1) **Sequences:** 1(1)
>tr|A0A3Q8Q127|A0A3Q8Q127_9HIV1 Envelope glycoprotein gp160 OS=Human immunodeficiency virus 1 OX=11676 GN=env PE=3 SV=1
2::A0A3Q8Q128 **Mass:** 96814 **Score:** 58 **Matches:** 1(1) **Sequences:** 1(1)
>tr|A0A3Q8Q128|A0A3Q8Q128_9HIV1 Envelope glycoprotein gp160 (Fragment) OS=Human immunodeficiency virus 1 OX=11676 GN=env PE=3 SV=1
2::A0A3Q8Q133 **Mass:** 95884 **Score:** 58 **Matches:** 1(1) **Sequences:** 1(1)
>tr|A0A3Q8Q133|A0A3Q8Q133_9HIV1 Envelope glycoprotein gp160 OS=Human immunodeficiency virus 1 OX=11676 GN=env PE=3 SV=1
2::A0A3Q8Q134 **Mass:** 95811 **Score:** 58 **Matches:** 1(1) **Sequences:** 1(1)
>tr|A0A3Q8Q134|A0A3Q8Q134_9HIV1 Envelope glycoprotein gp160 OS=Human immunodeficiency virus 1 OX=11676 GN=env PE=3 SV=1
2::A0A3Q8Q137 **Mass:** 97191 **Score:** 58 **Matches:** 1(1) **Sequences:** 1(1)
>tr|A0A3Q8Q137|A0A3Q8Q137_9HIV1 Envelope glycoprotein gp160 OS=Human immunodeficiency virus 1 OX=11676 GN=env PE=3 SV=1
2::A0A3Q8Q140 **Mass:** 96722 **Score:** 58 **Matches:** 1(1) **Sequences:** 1(1)
>tr|A0A3Q8Q140|A0A3Q8Q140_9HIV1 Envelope glycoprotein gp160 OS=Human immunodeficiency virus 1 OX=11676 GN=env PE=3 SV=1
2::A0A3Q8Q141 **Mass:** 95810 **Score:** 58 **Matches:** 1(1) **Sequences:** 1(1)
>tr|A0A3Q8Q141|A0A3Q8Q141_9HIV1 Envelope glycoprotein gp160 OS=Human immunodeficiency virus 1 OX=11676 GN=env PE=3 SV=1
2::A0A3Q8Q142 **Mass:** 96770 **Score:** 58 **Matches:** 1(1) **Sequences:** 1(1)
>tr|A0A3Q8Q142|A0A3Q8Q142_9HIV1 Envelope glycoprotein gp160 OS=Human immunodeficiency virus 1 OX=11676 GN=env PE=3 SV=1
2::A0A3Q8Q145 **Mass:** 97462 **Score:** 58 **Matches:** 1(1) **Sequences:** 1(1)
>tr|A0A3Q8Q145|A0A3Q8Q145_9HIV1 Envelope glycoprotein gp160 OS=Human immunodeficiency virus 1 OX=11676 GN=env PE=3 SV=1
2::A0A3Q8Q146 **Mass:** 97228 **Score:** 58 **Matches:** 1(1) **Sequences:** 1(1)
>tr|A0A3Q8Q146|A0A3Q8Q146_9HIV1 Envelope glycoprotein gp160 OS=Human immunodeficiency virus 1 OX=11676 GN=env PE=3 SV=1
2::A0A3Q8Q149 **Mass:** 97986 **Score:** 58 **Matches:** 1(1) **Sequences:** 1(1)
>tr|A0A3Q8Q149|A0A3Q8Q149_9HIV1 Envelope glycoprotein gp160 OS=Human immunodeficiency virus 1 OX=11676 GN=env PE=3 SV=1
2::A0A3Q8Q150 **Mass:** 96496 **Score:** 58 **Matches:** 1(1) **Sequences:** 1(1)
>tr|A0A3Q8Q150|A0A3Q8Q150_9HIV1 Envelope glycoprotein gp160 OS=Human immunodeficiency virus 1 OX=11676 GN=env PE=3 SV=1
2::A0A3Q8Q151 **Mass:** 97980 **Score:** 58 **Matches:** 1(1) **Sequences:** 1(1)
>tr|A0A3Q8Q151|A0A3Q8Q151_9HIV1 Envelope glycoprotein gp160 OS=Human immunodeficiency virus 1 OX=11676 GN=env PE=3 SV=1
2::A0A3Q8Q154 **Mass:** 96626 **Score:** 58 **Matches:** 1(1) **Sequences:** 1(1)
>tr|A0A3Q8Q154|A0A3Q8Q154_9HIV1 Envelope glycoprotein gp160 OS=Human immunodeficiency virus 1 OX=11676 GN=env PE=3 SV=1
2::A0A3Q8Q155 **Mass:** 97986 **Score:** 58 **Matches:** 1(1) **Sequences:** 1(1)
>tr|A0A3Q8Q155|A0A3Q8Q155_9HIV1 Envelope glycoprotein gp160 OS=Human immunodeficiency virus 1 OX=11676 GN=env PE=3 SV=1
2::A0A3Q8Q157 **Mass:** 97517 **Score:** 58 **Matches:** 1(1) **Sequences:** 1(1)
>tr|A0A3Q8Q157|A0A3Q8Q157_9HIV1 Envelope glycoprotein gp160 OS=Human immunodeficiency virus 1 OX=11676 GN=env PE=3 SV=1
2::A0A3Q8Q160 **Mass:** 98271 **Score:** 58 **Matches:** 1(1) **Sequences:** 1(1)
>tr|A0A3Q8Q160|A0A3Q8Q160_9HIV1 Envelope glycoprotein gp160 OS=Human immunodeficiency virus 1 OX=11676 GN=env PE=3 SV=1
2::A0A3Q8Q161 **Mass:** 97735 **Score:** 58 **Matches:** 1(1) **Sequences:** 1(1)
>tr|A0A3Q8Q161|A0A3Q8Q161_9HIV1 Envelope glycoprotein gp160 OS=Human immunodeficiency virus 1 OX=11676 GN=env PE=3 SV=1
2::A0A3Q8Q163 **Mass:** 97324 **Score:** 58 **Matches:** 1(1) **Sequences:** 1(1)
>tr|A0A3Q8Q163|A0A3Q8Q163_9HIV1 Envelope glycoprotein gp160 OS=Human immunodeficiency virus 1 OX=11676 GN=env PE=3 SV=1
2::A0A3Q8Q164 **Mass:** 97995 **Score:** 58 **Matches:** 1(1) **Sequences:** 1(1)
>tr|A0A3Q8Q164|A0A3Q8Q164_9HIV1 Envelope glycoprotein gp160 OS=Human immunodeficiency virus 1 OX=11676 GN=env PE=3 SV=1
2::A0A3Q8Q165 **Mass:** 98200 **Score:** 58 **Matches:** 1(1) **Sequences:** 1(1)
>tr|A0A3Q8Q165|A0A3Q8Q165_9HIV1 Envelope glycoprotein gp160 OS=Human immunodeficiency virus 1 OX=11676 GN=env PE=3 SV=1
2::A0A3Q8Q168 **Mass:** 97454 **Score:** 58 **Matches:** 1(1) **Sequences:** 1(1)
>tr|A0A3Q8Q168|A0A3Q8Q168_9HIV1 Envelope glycoprotein gp160 OS=Human immunodeficiency virus 1 OX=11676 GN=env PE=3 SV=1
2::A0A3Q8Q170 **Mass:** 98006 **Score:** 58 **Matches:** 1(1) **Sequences:** 1(1)
>tr|A0A3Q8Q170|A0A3Q8Q170_9HIV1 Envelope glycoprotein gp160 OS=Human immunodeficiency virus 1 OX=11676 GN=env PE=3 SV=1
2::A0A3Q8Q176 **Mass:** 97945 **Score:** 58 **Matches:** 1(1) **Sequences:** 1(1)
>tr|A0A3Q8Q176|A0A3Q8Q176_9HIV1 Envelope glycoprotein gp160 OS=Human immunodeficiency virus 1 OX=11676 GN=env PE=3 SV=1
2::A0A3Q8Q180 **Mass:** 97091 **Score:** 58 **Matches:** 1(1) **Sequences:** 1(1)
>tr|A0A3Q8Q180|A0A3Q8Q180_9HIV1 Envelope glycoprotein gp160 OS=Human immunodeficiency virus 1 OX=11676 GN=env PE=3 SV=1
2::A0A3Q8Q184 **Mass:** 98137 **Score:** 58 **Matches:** 1(1) **Sequences:** 1(1)
>tr|A0A3Q8Q184|A0A3Q8Q184_9HIV1 Envelope glycoprotein gp160 OS=Human immunodeficiency virus 1 OX=11676 GN=env PE=3 SV=1
2::A0A3Q8Q4G9 **Mass:** 96405 **Score:** 58 **Matches:** 1(1) **Sequences:** 1(1)
>tr|A0A3Q8Q4G9|A0A3Q8Q4G9_9HIV1 Envelope glycoprotein gp160 OS=Human immunodeficiency virus 1 OX=11676 GN=env PE=3 SV=1
2::A0A3Q8Q4K2 **Mass:** 96364 **Score:** 58 **Matches:** 1(1) **Sequences:** 1(1)
>tr|A0A3Q8Q4K2|A0A3Q8Q4K2_9HIV1 Envelope glycoprotein gp160 (Fragment) OS=Human immunodeficiency virus 1 OX=11676 GN=env PE=3 SV=1
2::A0A3Q8Q4P8 **Mass:** 98070 **Score:** 58 **Matches:** 1(1) **Sequences:** 1(1)
>tr|A0A3Q8Q4P8|A0A3Q8Q4P8_9HIV1 Envelope glycoprotein gp160 OS=Human immunodeficiency virus 1 OX=11676 GN=env PE=3 SV=1
2::A0A3Q8Q4S0 **Mass:** 97417 **Score:** 58 **Matches:** 1(1) **Sequences:** 1(1)
>tr|A0A3Q8Q4S0|A0A3Q8Q4S0_9HIV1 Envelope glycoprotein gp160 OS=Human immunodeficiency virus 1 OX=11676 GN=env PE=3 SV=1
2::A0A3Q8Q700 **Mass:** 96497 **Score:** 58 **Matches:** 1(1) **Sequences:** 1(1)
>tr|A0A3Q8Q700|A0A3Q8Q700_9HIV1 Envelope glycoprotein gp160 OS=Human immunodeficiency virus 1 OX=11676 GN=env PE=3 SV=1
2::A0A3Q8Q715 **Mass:** 97219 **Score:** 58 **Matches:** 1(1) **Sequences:** 1(1)
>tr|A0A3Q8Q715|A0A3Q8Q715_9HIV1 Envelope glycoprotein gp160 (Fragment) OS=Human immunodeficiency virus 1 OX=11676 GN=env PE=3 SV=1
2::A0A3Q8Q734 **Mass:** 96605 **Score:** 58 **Matches:** 1(1) **Sequences:** 1(1)
>tr|A0A3Q8Q734|A0A3Q8Q734_9HIV1 Envelope glycoprotein gp160 OS=Human immunodeficiency virus 1 OX=11676 GN=env PE=3 SV=1
2::A0A3Q8Q752 **Mass:** 97220 **Score:** 58 **Matches:** 1(1) **Sequences:** 1(1)
>tr|A0A3Q8Q752|A0A3Q8Q752_9HIV1 Envelope glycoprotein gp160 OS=Human immunodeficiency virus 1 OX=11676 GN=env PE=3 SV=1
2::A0A3Q8Q772 **Mass:** 97831 **Score:** 58 **Matches:** 1(1) **Sequences:** 1(1)
>tr|A0A3Q8Q772|A0A3Q8Q772_9HIV1 Envelope glycoprotein gp160 OS=Human immunodeficiency virus 1 OX=11676 GN=env PE=3 SV=1
2::A0A3Q8Q7A5 **Mass:** 96490 **Score:** 58 **Matches:** 1(1) **Sequences:** 1(1)
>tr|A0A3Q8Q7A5|A0A3Q8Q7A5_9HIV1 Envelope glycoprotein gp160 OS=Human immunodeficiency virus 1 OX=11676 GN=env PE=3 SV=1
2::A0A3Q8Q7B9 **Mass:** 96374 **Score:** 58 **Matches:** 1(1) **Sequences:** 1(1)
>tr|A0A3Q8Q7B9|A0A3Q8Q7B9_9HIV1 Envelope glycoprotein gp160 (Fragment) OS=Human immunodeficiency virus 1 OX=11676 GN=env PE=3 SV=1
2::A0A3Q8Q7D2 **Mass:** 96893 **Score:** 58 **Matches:** 1(1) **Sequences:** 1(1)
>tr|A0A3Q8Q7D2|A0A3Q8Q7D2_9HIV1 Envelope glycoprotein gp160 OS=Human immunodeficiency virus 1 OX=11676 GN=env PE=3 SV=1
2::A0A3Q8Q7E4 **Mass:** 96681 **Score:** 58 **Matches:** 1(1) **Sequences:** 1(1)
>tr|A0A3Q8Q7E4|A0A3Q8Q7E4_9HIV1 Envelope glycoprotein gp160 OS=Human immunodeficiency virus 1 OX=11676 GN=env PE=3 SV=1
2::A0A3Q8Q7G3 **Mass:** 97749 **Score:** 58 **Matches:** 1(1) **Sequences:** 1(1)
>tr|A0A3Q8Q7G3|A0A3Q8Q7G3_9HIV1 Envelope glycoprotein gp160 OS=Human immunodeficiency virus 1 OX=11676 GN=env PE=3 SV=1
2::A0A3Q8Q7I5 **Mass:** 98232 **Score:** 58 **Matches:** 1(1) **Sequences:** 1(1)
>tr|A0A3Q8Q7I5|A0A3Q8Q7I5_9HIV1 Envelope glycoprotein gp160 OS=Human immunodeficiency virus 1 OX=11676 GN=env PE=3 SV=1
2::A0A3S8NDG2 **Mass:** 96442 **Score:** 58 **Matches:** 1(1) **Sequences:** 1(1)
>tr|A0A3S8NDG2|A0A3S8NDG2_9HIV1 Envelope glycoprotein gp160 OS=Human immunodeficiency virus 1 OX=11676 GN=env PE=3 SV=1
2::A0A3S8NDH0 **Mass:** 96372 **Score:** 58 **Matches:** 1(1) **Sequences:** 1(1)
>tr|A0A3S8NDH0|A0A3S8NDH0_9HIV1 Envelope glycoprotein gp160 OS=Human immunodeficiency virus 1 OX=11676 GN=env PE=3 SV=1
2::A0A3S8NDH1 **Mass:** 96501 **Score:** 58 **Matches:** 1(1) **Sequences:** 1(1)
>tr|A0A3S8NDH1|A0A3S8NDH1_9HIV1 Envelope glycoprotein gp160 OS=Human immunodeficiency virus 1 OX=11676 GN=env PE=3 SV=1
2::A0A3S8NDH2 **Mass:** 96506 **Score:** 58 **Matches:** 1(1) **Sequences:** 1(1)
>tr|A0A3S8NDH2|A0A3S8NDH2_9HIV1 Envelope glycoprotein gp160 OS=Human immunodeficiency virus 1 OX=11676 GN=env PE=3 SV=1
2::A0A3S8NDH5 **Mass:** 96431 **Score:** 58 **Matches:** 1(1) **Sequences:** 1(1)
>tr|A0A3S8NDH5|A0A3S8NDH5_9HIV1 Envelope glycoprotein gp160 OS=Human immunodeficiency virus 1 OX=11676 GN=env PE=3 SV=1
2::A0A3S8NDH8 **Mass:** 96930 **Score:** 58 **Matches:** 1(1) **Sequences:** 1(1)
>tr|A0A3S8NDH8|A0A3S8NDH8_9HIV1 Envelope glycoprotein gp160 (Fragment) OS=Human immunodeficiency virus 1 OX=11676 GN=env PE=3 SV=1
2::A0A3S8NDI0 **Mass:** 96362 **Score:** 58 **Matches:** 1(1) **Sequences:** 1(1)
>tr|A0A3S8NDI0|A0A3S8NDI0_9HIV1 Envelope glycoprotein gp160 OS=Human immunodeficiency virus 1 OX=11676 GN=env PE=3 SV=1
2::A0A3S8NDI2 **Mass:** 96232 **Score:** 58 **Matches:** 1(1) **Sequences:** 1(1)
>tr|A0A3S8NDI2|A0A3S8NDI2_9HIV1 Envelope glycoprotein gp160 (Fragment) OS=Human immunodeficiency virus 1 OX=11676 GN=env PE=3 SV=1

2::A0A3S8NDI3 **Mass:** 96677 **Score:** 58 **Matches:** 1(1) **Sequences:** 1(1)
>tr|A0A3S8NDI3|A0A3S8NDI3_9HIV1 Envelope glycoprotein gp160 OS=Human immunodeficiency virus 1 OX=11676 GN=env PE=3 SV=1
2::A0A3S8NDI4 **Mass:** 97266 **Score:** 58 **Matches:** 1(1) **Sequences:** 1(1)
>tr|A0A3S8NDI4|A0A3S8NDI4_9HIV1 Envelope glycoprotein gp160 OS=Human immunodeficiency virus 1 OX=11676 GN=env PE=3 SV=1
2::A0A3S8NDI5 **Mass:** 96374 **Score:** 58 **Matches:** 1(1) **Sequences:** 1(1)
>tr|A0A3S8NDI5|A0A3S8NDI5_9HIV1 Envelope glycoprotein gp160 OS=Human immunodeficiency virus 1 OX=11676 GN=env PE=3 SV=1
2::A0A3S8NDI7 **Mass:** 97390 **Score:** 58 **Matches:** 1(1) **Sequences:** 1(1)
>tr|A0A3S8NDI7|A0A3S8NDI7_9HIV1 Envelope glycoprotein gp160 OS=Human immunodeficiency virus 1 OX=11676 GN=env PE=3 SV=1
2::A0A3S8NDI8 **Mass:** 96316 **Score:** 58 **Matches:** 1(1) **Sequences:** 1(1)
>tr|A0A3S8NDI8|A0A3S8NDI8_9HIV1 Envelope glycoprotein gp160 OS=Human immunodeficiency virus 1 OX=11676 GN=env PE=3 SV=1
2::A0A3S8NDI9 **Mass:** 96589 **Score:** 58 **Matches:** 1(1) **Sequences:** 1(1)
>tr|A0A3S8NDI9|A0A3S8NDI9_9HIV1 Envelope glycoprotein gp160 OS=Human immunodeficiency virus 1 OX=11676 GN=env PE=3 SV=1
2::A0A3S8NDJ0 **Mass:** 96597 **Score:** 58 **Matches:** 1(1) **Sequences:** 1(1)
>tr|A0A3S8NDJ0|A0A3S8NDJ0_9HIV1 Envelope glycoprotein gp160 OS=Human immunodeficiency virus 1 OX=11676 GN=env PE=3 SV=1
2::A0A3S8NDJ2 **Mass:** 96485 **Score:** 58 **Matches:** 1(1) **Sequences:** 1(1)
>tr|A0A3S8NDJ2|A0A3S8NDJ2_9HIV1 Envelope glycoprotein gp160 OS=Human immunodeficiency virus 1 OX=11676 GN=env PE=3 SV=1
2::A0A3S8NDJ5 **Mass:** 96446 **Score:** 58 **Matches:** 1(1) **Sequences:** 1(1)
>tr|A0A3S8NDJ5|A0A3S8NDJ5_9HIV1 Envelope glycoprotein gp160 OS=Human immunodeficiency virus 1 OX=11676 GN=env PE=3 SV=1
2::A0A3S8NDJ6 **Mass:** 96511 **Score:** 58 **Matches:** 1(1) **Sequences:** 1(1)
>tr|A0A3S8NDJ6|A0A3S8NDJ6_9HIV1 Envelope glycoprotein gp160 OS=Human immunodeficiency virus 1 OX=11676 GN=env PE=3 SV=1
2::A0A3S8NDJ7 **Mass:** 96694 **Score:** 58 **Matches:** 1(1) **Sequences:** 1(1)
>tr|A0A3S8NDJ7|A0A3S8NDJ7_9HIV1 Envelope glycoprotein gp160 OS=Human immunodeficiency virus 1 OX=11676 GN=env PE=3 SV=1
2::A0A3S8NDJ8 **Mass:** 97161 **Score:** 58 **Matches:** 1(1) **Sequences:** 1(1)
>tr|A0A3S8NDJ8|A0A3S8NDJ8_9HIV1 Envelope glycoprotein gp160 OS=Human immunodeficiency virus 1 OX=11676 GN=env PE=3 SV=1
2::A0A3S8NDK2 **Mass:** 97958 **Score:** 58 **Matches:** 1(1) **Sequences:** 1(1)
>tr|A0A3S8NDK2|A0A3S8NDK2_9HIV1 Envelope glycoprotein gp160 OS=Human immunodeficiency virus 1 OX=11676 GN=env PE=3 SV=1
2::A0A3S8NDK3 **Mass:** 98277 **Score:** 58 **Matches:** 1(1) **Sequences:** 1(1)
>tr|A0A3S8NDK3|A0A3S8NDK3_9HIV1 Envelope glycoprotein gp160 OS=Human immunodeficiency virus 1 OX=11676 GN=env PE=3 SV=1
2::A0A3S8NDK4 **Mass:** 98456 **Score:** 58 **Matches:** 1(1) **Sequences:** 1(1)
>tr|A0A3S8NDK4|A0A3S8NDK4_9HIV1 Envelope glycoprotein gp160 OS=Human immunodeficiency virus 1 OX=11676 GN=env PE=3 SV=1
2::A0A3S8NDK6 **Mass:** 96554 **Score:** 58 **Matches:** 1(1) **Sequences:** 1(1)
>tr|A0A3S8NDK6|A0A3S8NDK6_9HIV1 Envelope glycoprotein gp160 OS=Human immunodeficiency virus 1 OX=11676 GN=env PE=3 SV=1
2::A0A3S8NDK7 **Mass:** 96364 **Score:** 58 **Matches:** 1(1) **Sequences:** 1(1)
>tr|A0A3S8NDK7|A0A3S8NDK7_9HIV1 Envelope glycoprotein gp160 (Fragment) OS=Human immunodeficiency virus 1 OX=11676 GN=env PE=3 SV=1
2::A0A3S8NDK9 **Mass:** 97625 **Score:** 58 **Matches:** 1(1) **Sequences:** 1(1)
>tr|A0A3S8NDK9|A0A3S8NDK9_9HIV1 Envelope glycoprotein gp160 OS=Human immunodeficiency virus 1 OX=11676 GN=env PE=3 SV=1
2::A0A3S8NDL1 **Mass:** 98106 **Score:** 58 **Matches:** 1(1) **Sequences:** 1(1)
>tr|A0A3S8NDL1|A0A3S8NDL1_9HIV1 Envelope glycoprotein gp160 OS=Human immunodeficiency virus 1 OX=11676 GN=env PE=3 SV=1
2::A0A3S8NDL3 **Mass:** 97445 **Score:** 58 **Matches:** 1(1) **Sequences:** 1(1)
>tr|A0A3S8NDL3|A0A3S8NDL3_9HIV1 Envelope glycoprotein gp160 OS=Human immunodeficiency virus 1 OX=11676 GN=env PE=3 SV=1
2::A0A3S8NDL7 **Mass:** 97384 **Score:** 58 **Matches:** 1(1) **Sequences:** 1(1)
>tr|A0A3S8NDL7|A0A3S8NDL7_9HIV1 Envelope glycoprotein gp160 OS=Human immunodeficiency virus 1 OX=11676 GN=env PE=3 SV=1
2::A0A3S8NDM0 **Mass:** 96352 **Score:** 58 **Matches:** 1(1) **Sequences:** 1(1)
>tr|A0A3S8NDM0|A0A3S8NDM0_9HIV1 Envelope glycoprotein gp160 (Fragment) OS=Human immunodeficiency virus 1 OX=11676 GN=env PE=3 SV=1
2::A0A3S8NDM1 **Mass:** 96963 **Score:** 58 **Matches:** 1(1) **Sequences:** 1(1)
>tr|A0A3S8NDM1|A0A3S8NDM1_9HIV1 Envelope glycoprotein gp160 OS=Human immunodeficiency virus 1 OX=11676 GN=env PE=3 SV=1
2::A0A3S8NDM2 **Mass:** 98845 **Score:** 58 **Matches:** 1(1) **Sequences:** 1(1)
>tr|A0A3S8NDM2|A0A3S8NDM2_9HIV1 Envelope glycoprotein gp160 OS=Human immunodeficiency virus 1 OX=11676 GN=env PE=3 SV=1
2::A0A3S8NDM6 **Mass:** 97751 **Score:** 58 **Matches:** 1(1) **Sequences:** 1(1)
>tr|A0A3S8NDM6|A0A3S8NDM6_9HIV1 Envelope glycoprotein gp160 OS=Human immunodeficiency virus 1 OX=11676 GN=env PE=3 SV=1
2::A0A3S8NDN0 **Mass:** 95839 **Score:** 58 **Matches:** 1(1) **Sequences:** 1(1)
>tr|A0A3S8NDN0|A0A3S8NDN0_9HIV1 Envelope glycoprotein gp160 OS=Human immunodeficiency virus 1 OX=11676 GN=env PE=3 SV=1
2::A0A3S8NDN1 **Mass:** 97384 **Score:** 58 **Matches:** 1(1) **Sequences:** 1(1)
>tr|A0A3S8NDN1|A0A3S8NDN1_9HIV1 Envelope glycoprotein gp160 OS=Human immunodeficiency virus 1 OX=11676 GN=env PE=3 SV=1
2::A0A3S8NDN2 **Mass:** 98393 **Score:** 58 **Matches:** 1(1) **Sequences:** 1(1)
>tr|A0A3S8NDN2|A0A3S8NDN2_9HIV1 Envelope glycoprotein gp160 OS=Human immunodeficiency virus 1 OX=11676 GN=env PE=3 SV=1
2::A0A3S8NDP1 **Mass:** 98400 **Score:** 58 **Matches:** 1(1) **Sequences:** 1(1)
>tr|A0A3S8NDP1|A0A3S8NDP1_9HIV1 Envelope glycoprotein gp160 OS=Human immunodeficiency virus 1 OX=11676 GN=env PE=3 SV=1
2::A0A499T153 **Mass:** 97836 **Score:** 58 **Matches:** 1(1) **Sequences:** 1(1)
>tr|A0A499T153|A0A499T153_9HIV1 Envelope glycoprotein gp160 OS=Human immunodeficiency virus 1 OX=11676 GN=env PE=3 SV=1
2::A0A499T3W8 **Mass:** 99325 **Score:** 58 **Matches:** 1(1) **Sequences:** 1(1)
>tr|A0A499T3W8|A0A499T3W8_9HIV1 Envelope glycoprotein gp160 OS=Human immunodeficiency virus 1 OX=11676 GN=env PE=3 SV=1
2::A0A517EQ66 **Mass:** 98419 **Score:** 58 **Matches:** 1(1) **Sequences:** 1(1)
>tr|A0A517EQ66|A0A517EQ66_9HIV1 Envelope glycoprotein gp160 OS=Human immunodeficiency virus 1 OX=11676 GN=env PE=3 SV=1
2::A0A517EQ79 **Mass:** 97467 **Score:** 58 **Matches:** 1(1) **Sequences:** 1(1)
>tr|A0A517EQ79|A0A517EQ79_9HIV1 Envelope glycoprotein gp160 OS=Human immunodeficiency virus 1 OX=11676 GN=env PE=3 SV=1
2::A0A517EQA3 **Mass:** 97212 **Score:** 58 **Matches:** 1(1) **Sequences:** 1(1)
>tr|A0A517EQA3|A0A517EQA3_9HIV1 Envelope glycoprotein gp160 OS=Human immunodeficiency virus 1 OX=11676 GN=env PE=3 SV=1
2::A0A517EQA9 **Mass:** 97329 **Score:** 58 **Matches:** 1(1) **Sequences:** 1(1)
>tr|A0A517EQA9|A0A517EQA9_9HIV1 Envelope glycoprotein gp160 OS=Human immunodeficiency virus 1 OX=11676 GN=env PE=3 SV=1
2::A0A517EQC6 **Mass:** 97967 **Score:** 58 **Matches:** 1(1) **Sequences:** 1(1)
>tr|A0A517EQC6|A0A517EQC6_9HIV1 Envelope glycoprotein gp160 OS=Human immunodeficiency virus 1 OX=11676 GN=env PE=3 SV=1
2::A0A517EQD0 **Mass:** 97497 **Score:** 58 **Matches:** 1(1) **Sequences:** 1(1)
>tr|A0A517EQD0|A0A517EQD0_9HIV1 Envelope glycoprotein gp160 OS=Human immunodeficiency virus 1 OX=11676 GN=env PE=3 SV=1
2::A0A5B8H6Z8 **Mass:** 97850 **Score:** 58 **Matches:** 1(1) **Sequences:** 1(1)
>tr|A0A5B8H6Z8|A0A5B8H6Z8_9HIV1 Envelope glycoprotein gp160 OS=Human immunodeficiency virus 1 OX=11676 GN=env PE=3 SV=1
2::A0A5B8H714 **Mass:** 98041 **Score:** 58 **Matches:** 1(1) **Sequences:** 1(1)
>tr|A0A5B8H714|A0A5B8H714_9HIV1 Envelope glycoprotein gp160 OS=Human immunodeficiency virus 1 OX=11676 GN=env PE=3 SV=1
2::A0A5B8H729 **Mass:** 97995 **Score:** 58 **Matches:** 1(1) **Sequences:** 1(1)
>tr|A0A5B8H729|A0A5B8H729_9HIV1 Envelope glycoprotein gp160 OS=Human immunodeficiency virus 1 OX=11676 GN=env PE=3 SV=1
2::A0A5B8H742 **Mass:** 98755 **Score:** 58 **Matches:** 1(1) **Sequences:** 1(1)
>tr|A0A5B8H742|A0A5B8H742_9HIV1 Envelope glycoprotein gp160 OS=Human immunodeficiency virus 1 OX=11676 GN=env PE=3 SV=1
2::A0A5B8H755 **Mass:** 98243 **Score:** 58 **Matches:** 1(1) **Sequences:** 1(1)
>tr|A0A5B8H755|A0A5B8H755_9HIV1 Envelope glycoprotein gp160 OS=Human immunodeficiency virus 1 OX=11676 GN=env PE=3 SV=1
2::A0A5B8H771 **Mass:** 98058 **Score:** 58 **Matches:** 1(1) **Sequences:** 1(1)
>tr|A0A5B8H771|A0A5B8H771_9HIV1 Envelope glycoprotein gp160 OS=Human immunodeficiency virus 1 OX=11676 GN=env PE=3 SV=1
2::A0A5B8H7M4 **Mass:** 97663 **Score:** 58 **Matches:** 1(1) **Sequences:** 1(1)
>tr|A0A5B8H7M4|A0A5B8H7M4_9HIV1 Envelope glycoprotein gp160 OS=Human immunodeficiency virus 1 OX=11676 GN=env PE=3 SV=1
2::A0A5B8H7N8 **Mass:** 97991 **Score:** 58 **Matches:** 1(1) **Sequences:** 1(1)
>tr|A0A5B8H7N8|A0A5B8H7N8_9HIV1 Envelope glycoprotein gp160 OS=Human immunodeficiency virus 1 OX=11676 GN=env PE=3 SV=1
2::A0A5B8H7Q1 **Mass:** 98146 **Score:** 58 **Matches:** 1(1) **Sequences:** 1(1)
>tr|A0A5B8H7Q1|A0A5B8H7Q1_9HIV1 Envelope glycoprotein gp160 OS=Human immunodeficiency virus 1 OX=11676 GN=env PE=3 SV=1
2::A0A5B8H7Q2 **Mass:** 97846 **Score:** 58 **Matches:** 1(1) **Sequences:** 1(1)
>tr|A0A5B8H7Q2|A0A5B8H7Q2_9HIV1 Envelope glycoprotein gp160 OS=Human immunodeficiency virus 1 OX=11676 GN=env PE=3 SV=1
2::A0A5B8H7R0 **Mass:** 97965 **Score:** 58 **Matches:** 1(1) **Sequences:** 1(1)
>tr|A0A5B8H7R0|A0A5B8H7R0_9HIV1 Envelope glycoprotein gp160 OS=Human immunodeficiency virus 1 OX=11676 GN=env PE=3 SV=1
2::A0A5B8H7S5 **Mass:** 98019 **Score:** 58 **Matches:** 1(1) **Sequences:** 1(1)
>tr|A0A5B8H7S5|A0A5B8H7S5_9HIV1 Envelope glycoprotein gp160 OS=Human immunodeficiency virus 1 OX=11676 GN=env PE=3 SV=1
2::A0A5B8H7Z0 **Mass:** 97322 **Score:** 58 **Matches:** 1(1) **Sequences:** 1(1)
>tr|A0A5B8H7Z0|A0A5B8H7Z0_9HIV1 Envelope glycoprotein gp160 OS=Human immunodeficiency virus 1 OX=11676 GN=env PE=3 SV=1
2::A0A5B8HRC2 **Mass:** 98644 **Score:** 58 **Matches:** 1(1) **Sequences:** 1(1)
>tr|A0A5B8HRC2|A0A5B8HRC2_9HIV1 Envelope glycoprotein gp160 OS=Human immunodeficiency virus 1 OX=11676 GN=env PE=3 SV=1
2::A0A5B8HRD4 **Mass:** 97455 **Score:** 58 **Matches:** 1(1) **Sequences:** 1(1)
>tr|A0A5B8HRD4|A0A5B8HRD4_9HIV1 Envelope glycoprotein gp160 OS=Human immunodeficiency virus 1 OX=11676 GN=env PE=3 SV=1
2::A0A5B8HRE4 **Mass:** 98218 **Score:** 58 **Matches:** 1(1) **Sequences:** 1(1)
>tr|A0A5B8HRE4|A0A5B8HRE4_9HIV1 Envelope glycoprotein gp160 OS=Human immunodeficiency virus 1 OX=11676 GN=env PE=3 SV=1
2::A0A5B8HRF6 **Mass:** 98113 **Score:** 58 **Matches:** 1(1) **Sequences:** 1(1)
>tr|A0A5B8HRF6|A0A5B8HRF6_9HIV1 Envelope glycoprotein gp160 OS=Human immunodeficiency virus 1 OX=11676 GN=env PE=3 SV=1
2::A0A5B8HRG7 **Mass:** 98034 **Score:** 58 **Matches:** 1(1) **Sequences:** 1(1)
>tr|A0A5B8HRG7|A0A5B8HRG7_9HIV1 Envelope glycoprotein gp160 OS=Human immunodeficiency virus 1 OX=11676 GN=env PE=3 SV=1

2::A0A5B8HSM6 Mass: 98584 Score: 58 Matches: 1(1) Sequences: 1(1)
>tr|A0A5B8HSM6|A0A5B8HSM6_9HIV1 Envelope glycoprotein gp160 OS=Human immunodeficiency virus 1 OX=11676 GN=env PE=3 SV=1
2::A0A5B8HST0 Mass: 97835 Score: 58 Matches: 1(1) Sequences: 1(1)
>tr|A0A5B8HST0|A0A5B8HST0_9HIV1 Envelope glycoprotein gp160 OS=Human immunodeficiency virus 1 OX=11676 GN=env PE=3 SV=1
2::A0A5B8HSU2 Mass: 97742 Score: 58 Matches: 1(1) Sequences: 1(1)
>tr|A0A5B8HSU2|A0A5B8HSU2_9HIV1 Envelope glycoprotein gp160 OS=Human immunodeficiency virus 1 OX=11676 GN=env PE=3 SV=1
2::A0A5B8HSW0 Mass: 98031 Score: 58 Matches: 1(1) Sequences: 1(1)
>tr|A0A5B8HSW0|A0A5B8HSW0_9HIV1 Envelope glycoprotein gp160 OS=Human immunodeficiency virus 1 OX=11676 GN=env PE=3 SV=1
2::A0A5B8HSW3 Mass: 98877 Score: 58 Matches: 1(1) Sequences: 1(1)
>tr|A0A5B8HSW3|A0A5B8HSW3_9HIV1 Envelope glycoprotein gp160 OS=Human immunodeficiency virus 1 OX=11676 GN=env PE=3 SV=1
2::A0A5B8HSX4 Mass: 98085 Score: 58 Matches: 1(1) Sequences: 1(1)
>tr|A0A5B8HSX4|A0A5B8HSX4_9HIV1 Envelope glycoprotein gp160 OS=Human immunodeficiency virus 1 OX=11676 GN=env PE=3 SV=1
2::A0A5B8HSX6 Mass: 98117 Score: 58 Matches: 1(1) Sequences: 1(1)
>tr|A0A5B8HSX6|A0A5B8HSX6_9HIV1 Envelope glycoprotein gp160 OS=Human immunodeficiency virus 1 OX=11676 GN=env PE=3 SV=1
2::A0A5B8HSY8 Mass: 98261 Score: 58 Matches: 1(1) Sequences: 1(1)
>tr|A0A5B8HSY8|A0A5B8HSY8_9HIV1 Envelope glycoprotein gp160 OS=Human immunodeficiency virus 1 OX=11676 GN=env PE=3 SV=1
2::A0A5B8HT01 Mass: 98004 Score: 58 Matches: 1(1) Sequences: 1(1)
>tr|A0A5B8HT01|A0A5B8HT01_9HIV1 Envelope glycoprotein gp160 OS=Human immunodeficiency virus 1 OX=11676 GN=env PE=3 SV=1
2::A0A5B8HT14 Mass: 97988 Score: 58 Matches: 1(1) Sequences: 1(1)
>tr|A0A5B8HT14|A0A5B8HT14_9HIV1 Envelope glycoprotein gp160 OS=Human immunodeficiency virus 1 OX=11676 GN=env PE=3 SV=1
2::A0A5B8HT44 Mass: 97844 Score: 58 Matches: 1(1) Sequences: 1(1)
>tr|A0A5B8HT44|A0A5B8HT44_9HIV1 Envelope glycoprotein gp160 OS=Human immunodeficiency virus 1 OX=11676 GN=env PE=3 SV=1
2::A0A5B8HT56 Mass: 98255 Score: 58 Matches: 1(1) Sequences: 1(1)
>tr|A0A5B8HT56|A0A5B8HT56_9HIV1 Envelope glycoprotein gp160 OS=Human immunodeficiency virus 1 OX=11676 GN=env PE=3 SV=1
2::A0A5B8HT69 Mass: 97589 Score: 58 Matches: 1(1) Sequences: 1(1)
>tr|A0A5B8HT69|A0A5B8HT69_9HIV1 Envelope glycoprotein gp160 OS=Human immunodeficiency virus 1 OX=11676 GN=env PE=3 SV=1
2::A0A5B8HT82 Mass: 98790 Score: 58 Matches: 1(1) Sequences: 1(1)
>tr|A0A5B8HT82|A0A5B8HT82_9HIV1 Envelope glycoprotein gp160 OS=Human immunodeficiency virus 1 OX=11676 GN=env PE=3 SV=1
2::A0A5B8HT92 Mass: 98006 Score: 58 Matches: 1(1) Sequences: 1(1)
>tr|A0A5B8HT92|A0A5B8HT92_9HIV1 Envelope glycoprotein gp160 OS=Human immunodeficiency virus 1 OX=11676 GN=env PE=3 SV=1
2::A0A5B8HTA4 Mass: 98498 Score: 58 Matches: 1(1) Sequences: 1(1)
>tr|A0A5B8HTA4|A0A5B8HTA4_9HIV1 Envelope glycoprotein gp160 OS=Human immunodeficiency virus 1 OX=11676 GN=env PE=3 SV=1
2::A0A5B8HTB0 Mass: 97547 Score: 58 Matches: 1(1) Sequences: 1(1)
>tr|A0A5B8HTB0|A0A5B8HTB0_9HIV1 Envelope glycoprotein gp160 OS=Human immunodeficiency virus 1 OX=11676 GN=env PE=3 SV=1
2::A0A5B8HTB7 Mass: 97739 Score: 58 Matches: 1(1) Sequences: 1(1)
>tr|A0A5B8HTB7|A0A5B8HTB7_9HIV1 Envelope glycoprotein gp160 OS=Human immunodeficiency virus 1 OX=11676 GN=env PE=3 SV=1
2::A0A5B8HTD0 Mass: 97931 Score: 58 Matches: 1(1) Sequences: 1(1)
>tr|A0A5B8HTD0|A0A5B8HTD0_9HIV1 Envelope glycoprotein gp160 OS=Human immunodeficiency virus 1 OX=11676 GN=env PE=3 SV=1
2::A0A5B8HTE3 Mass: 97856 Score: 58 Matches: 1(1) Sequences: 1(1)
>tr|A0A5B8HTE3|A0A5B8HTE3_9HIV1 Envelope glycoprotein gp160 OS=Human immunodeficiency virus 1 OX=11676 GN=env PE=3 SV=1
2::A0A5B8HTG0 Mass: 97949 Score: 58 Matches: 1(1) Sequences: 1(1)
>tr|A0A5B8HTG0|A0A5B8HTG0_9HIV1 Envelope glycoprotein gp160 OS=Human immunodeficiency virus 1 OX=11676 GN=env PE=3 SV=1
2::A0A5B8HTH4 Mass: 98122 Score: 58 Matches: 1(1) Sequences: 1(1)
>tr|A0A5B8HTH4|A0A5B8HTH4_9HIV1 Envelope glycoprotein gp160 OS=Human immunodeficiency virus 1 OX=11676 GN=env PE=3 SV=1
2::A0A5B8HXQ9 Mass: 97934 Score: 58 Matches: 1(1) Sequences: 1(1)
>tr|A0A5B8HXQ9|A0A5B8HXQ9_9HIV1 Envelope glycoprotein gp160 OS=Human immunodeficiency virus 1 OX=11676 GN=env PE=3 SV=1
2::A0A5B8HXT1 Mass: 97910 Score: 58 Matches: 1(1) Sequences: 1(1)
>tr|A0A5B8HXT1|A0A5B8HXT1_9HIV1 Envelope glycoprotein gp160 OS=Human immunodeficiency virus 1 OX=11676 GN=env PE=3 SV=1
2::A0A5B8HXU6 Mass: 97971 Score: 58 Matches: 1(1) Sequences: 1(1)
>tr|A0A5B8HXU6|A0A5B8HXU6_9HIV1 Envelope glycoprotein gp160 OS=Human immunodeficiency virus 1 OX=11676 GN=env PE=3 SV=1
2::A0A5B8HXW4 Mass: 98060 Score: 58 Matches: 1(1) Sequences: 1(1)
>tr|A0A5B8HXW4|A0A5B8HXW4_9HIV1 Envelope glycoprotein gp160 OS=Human immunodeficiency virus 1 OX=11676 GN=env PE=3 SV=1
2::A0A5B8HXY4 Mass: 97568 Score: 58 Matches: 1(1) Sequences: 1(1)
>tr|A0A5B8HXY4|A0A5B8HXY4_9HIV1 Envelope glycoprotein gp160 OS=Human immunodeficiency virus 1 OX=11676 GN=env PE=3 SV=1
2::A0A5B8HXZ7 Mass: 98049 Score: 58 Matches: 1(1) Sequences: 1(1)
>tr|A0A5B8HXZ7|A0A5B8HXZ7_9HIV1 Envelope glycoprotein gp160 OS=Human immunodeficiency virus 1 OX=11676 GN=env PE=3 SV=1
2::A0A5B8HY45 Mass: 97806 Score: 58 Matches: 1(1) Sequences: 1(1)
>tr|A0A5B8HY45|A0A5B8HY45_9HIV1 Envelope glycoprotein gp160 OS=Human immunodeficiency virus 1 OX=11676 GN=env PE=3 SV=1
2::A0A5B8HY63 Mass: 98755 Score: 58 Matches: 1(1) Sequences: 1(1)
>tr|A0A5B8HY63|A0A5B8HY63_9HIV1 Envelope glycoprotein gp160 OS=Human immunodeficiency virus 1 OX=11676 GN=env PE=3 SV=1
2::A0A5B8HY78 Mass: 97880 Score: 58 Matches: 1(1) Sequences: 1(1)
>tr|A0A5B8HY78|A0A5B8HY78_9HIV1 Envelope glycoprotein gp160 OS=Human immunodeficiency virus 1 OX=11676 GN=env PE=3 SV=1
2::A0A5B8HYA3 Mass: 97979 Score: 58 Matches: 1(1) Sequences: 1(1)
>tr|A0A5B8HYA3|A0A5B8HYA3_9HIV1 Envelope glycoprotein gp160 OS=Human immunodeficiency virus 1 OX=11676 GN=env PE=3 SV=1
2::A0A5B8HYC3 Mass: 98189 Score: 58 Matches: 1(1) Sequences: 1(1)
>tr|A0A5B8HYC3|A0A5B8HYC3_9HIV1 Envelope glycoprotein gp160 OS=Human immunodeficiency virus 1 OX=11676 GN=env PE=3 SV=1
2::A0A5B8HYE1 Mass: 98004 Score: 58 Matches: 1(1) Sequences: 1(1)
>tr|A0A5B8HYE1|A0A5B8HYE1_9HIV1 Envelope glycoprotein gp160 OS=Human immunodeficiency virus 1 OX=11676 GN=env PE=3 SV=1
2::A0A5J6SBD6 Mass: 36965 Score: 58 Matches: 1(1) Sequences: 1(1)
>tr|A0A5J6SBD6|A0A5J6SBD6_9HIV1 Envelope glycoprotein (Fragment) OS=Human immunodeficiency virus 1 OX=11676 GN=env PE=4 SV=1
2::B0KZ62 Mass: 99557 Score: 58 Matches: 1(1) Sequences: 1(1)
>tr|B0KZ62|B0KZ62_9HIV1 Envelope glycoprotein gp160 OS=Human immunodeficiency virus 1 OX=11676 GN=env PE=3 SV=1
2::B0KZ64 Mass: 99583 Score: 58 Matches: 1(1) Sequences: 1(1)
>tr|B0KZ64|B0KZ64_9HIV1 Envelope glycoprotein gp160 OS=Human immunodeficiency virus 1 OX=11676 GN=env PE=3 SV=1
2::B0YWK2 Mass: 96661 Score: 58 Matches: 1(1) Sequences: 1(1)
>tr|B0YWK2|B0YWK2_9HIV1 Envelope glycoprotein gp160 OS=Human immunodeficiency virus 1 OX=11676 GN=env PE=3 SV=1
2::B0YWK7 Mass: 96714 Score: 58 Matches: 1(1) Sequences: 1(1)
>tr|B0YWK7|B0YWK7_9HIV1 Envelope glycoprotein gp160 OS=Human immunodeficiency virus 1 OX=11676 GN=env PE=3 SV=1
2::B0YWL6 Mass: 96698 Score: 58 Matches: 1(1) Sequences: 1(1)
>tr|B0YWL6|B0YWL6_9HIV1 Envelope glycoprotein gp160 OS=Human immunodeficiency virus 1 OX=11676 GN=env PE=3 SV=1
2::B0YWM1 Mass: 96675 Score: 58 Matches: 1(1) Sequences: 1(1)
>tr|B0YWM1|B0YWM1_9HIV1 Envelope glycoprotein gp160 OS=Human immunodeficiency virus 1 OX=11676 GN=env PE=3 SV=1
2::B0YWN4 Mass: 96604 Score: 58 Matches: 1(1) Sequences: 1(1)
>tr|B0YWN4|B0YWN4_9HIV1 Envelope glycoprotein gp160 OS=Human immunodeficiency virus 1 OX=11676 GN=env PE=3 SV=1
2::B0YWQ1 Mass: 96587 Score: 58 Matches: 1(1) Sequences: 1(1)
>tr|B0YWQ1|B0YWQ1_9HIV1 Envelope glycoprotein gp160 OS=Human immunodeficiency virus 1 OX=11676 GN=env PE=3 SV=1
2::B0YWR1 Mass: 98593 Score: 58 Matches: 1(1) Sequences: 1(1)
>tr|B0YWR1|B0YWR1_9HIV1 Envelope glycoprotein gp160 OS=Human immunodeficiency virus 1 OX=11676 GN=env PE=3 SV=1
2::B0YWS0 Mass: 96688 Score: 58 Matches: 1(1) Sequences: 1(1)
>tr|B0YWS0|B0YWS0_9HIV1 Envelope glycoprotein gp160 OS=Human immunodeficiency virus 1 OX=11676 GN=env PE=3 SV=1
2::B0YWW3 Mass: 99005 Score: 58 Matches: 1(1) Sequences: 1(1)
>tr|B0YWW3|B0YWW3_9HIV1 Envelope glycoprotein gp160 OS=Human immunodeficiency virus 1 OX=11676 GN=env PE=3 SV=1
2::B0YWX4 Mass: 96673 Score: 58 Matches: 1(1) Sequences: 1(1)
>tr|B0YWX4|B0YWX4_9HIV1 Envelope glycoprotein gp160 OS=Human immunodeficiency virus 1 OX=11676 GN=env PE=3 SV=1
2::B0YWX8 Mass: 96701 Score: 58 Matches: 1(1) Sequences: 1(1)
>tr|B0YWX8|B0YWX8_9HIV1 Envelope glycoprotein gp160 OS=Human immunodeficiency virus 1 OX=11676 GN=env PE=3 SV=1
2::B0YWY2 Mass: 96687 Score: 58 Matches: 1(1) Sequences: 1(1)
>tr|B0YWY2|B0YWY2_9HIV1 Envelope glycoprotein gp160 OS=Human immunodeficiency virus 1 OX=11676 GN=env PE=3 SV=1
2::B0YWY6 Mass: 96657 Score: 58 Matches: 1(1) Sequences: 1(1)
>tr|B0YWY6|B0YWY6_9HIV1 Envelope glycoprotein gp160 OS=Human immunodeficiency virus 1 OX=11676 GN=env PE=3 SV=1
2::B0YWZ0 Mass: 96683 Score: 58 Matches: 1(1) Sequences: 1(1)
>tr|B0YWZ0|B0YWZ0_9HIV1 Envelope glycoprotein gp160 OS=Human immunodeficiency virus 1 OX=11676 GN=env PE=3 SV=1
2::B0YX22 Mass: 96715 Score: 58 Matches: 1(1) Sequences: 1(1)
>tr|B0YX22|B0YX22_9HIV1 Envelope glycoprotein gp160 OS=Human immunodeficiency virus 1 OX=11676 GN=env PE=3 SV=1
2::B0YX38 Mass: 96701 Score: 58 Matches: 1(1) Sequences: 1(1)
>tr|B0YX38|B0YX38_9HIV1 Envelope glycoprotein gp160 OS=Human immunodeficiency virus 1 OX=11676 GN=env PE=3 SV=1
2::B0YX46 Mass: 96661 Score: 58 Matches: 1(1) Sequences: 1(1)
>tr|B0YX46|B0YX46_9HIV1 Envelope glycoprotein gp160 OS=Human immunodeficiency virus 1 OX=11676 GN=env PE=3 SV=1
2::B0YX50 Mass: 96587 Score: 58 Matches: 1(1) Sequences: 1(1)
>tr|B0YX50|B0YX50_9HIV1 Envelope glycoprotein gp160 OS=Human immunodeficiency virus 1 OX=11676 GN=env PE=3 SV=1

2::B0YX58 **Mass:** 96686 **Score:** 58 **Matches:** 1(1) **Sequences:** 1(1)
>tr|B0YX58|B0YX58_9HIV1 Envelope glycoprotein gp160 OS=Human immunodeficiency virus 1 OX=11676 GN=env PE=3 SV=1
2::B0YX82 **Mass:** 96695 **Score:** 58 **Matches:** 1(1) **Sequences:** 1(1)
>tr|B0YX82|B0YX82_9HIV1 Envelope glycoprotein gp160 OS=Human immunodeficiency virus 1 OX=11676 GN=env PE=3 SV=1
2::B0YX86 **Mass:** 96687 **Score:** 58 **Matches:** 1(1) **Sequences:** 1(1)
>tr|B0YX86|B0YX86_9HIV1 Envelope glycoprotein gp160 OS=Human immunodeficiency virus 1 OX=11676 GN=env PE=3 SV=1
2::B0YX90 **Mass:** 96674 **Score:** 58 **Matches:** 1(1) **Sequences:** 1(1)
>tr|B0YX90|B0YX90_9HIV1 Envelope glycoprotein gp160 OS=Human immunodeficiency virus 1 OX=11676 GN=env PE=3 SV=1
2::B0YX94 **Mass:** 96702 **Score:** 58 **Matches:** 1(1) **Sequences:** 1(1)
>tr|B0YX94|B0YX94_9HIV1 Envelope glycoprotein gp160 OS=Human immunodeficiency virus 1 OX=11676 GN=env PE=3 SV=1
2::B0YX98 **Mass:** 96663 **Score:** 58 **Matches:** 1(1) **Sequences:** 1(1)
>tr|B0YX98|B0YX98_9HIV1 Envelope glycoprotein gp160 OS=Human immunodeficiency virus 1 OX=11676 GN=env PE=3 SV=1
2::B0YXA2 **Mass:** 96616 **Score:** 58 **Matches:** 1(1) **Sequences:** 1(1)
>tr|B0YXA2|B0YXA2_9HIV1 Envelope glycoprotein gp160 OS=Human immunodeficiency virus 1 OX=11676 GN=env PE=3 SV=1
2::B0YXB3 **Mass:** 96770 **Score:** 58 **Matches:** 1(1) **Sequences:** 1(1)
>tr|B0YXB3|B0YXB3_9HIV1 Envelope glycoprotein gp160 OS=Human immunodeficiency virus 1 OX=11676 GN=env PE=3 SV=1
2::B0YXB7 **Mass:** 96672 **Score:** 58 **Matches:** 1(1) **Sequences:** 1(1)
>tr|B0YXB7|B0YXB7_9HIV1 Envelope glycoprotein gp160 OS=Human immunodeficiency virus 1 OX=11676 GN=env PE=3 SV=1
2::B0YXC1 **Mass:** 96753 **Score:** 58 **Matches:** 1(1) **Sequences:** 1(1)
>tr|B0YXC1|B0YXC1_9HIV1 Envelope glycoprotein gp160 OS=Human immunodeficiency virus 1 OX=11676 GN=env PE=3 SV=1
2::B0YXC5 **Mass:** 96674 **Score:** 58 **Matches:** 1(1) **Sequences:** 1(1)
>tr|B0YXC5|B0YXC5_9HIV1 Envelope glycoprotein gp160 OS=Human immunodeficiency virus 1 OX=11676 GN=env PE=3 SV=1
2::B0YXC9 **Mass:** 96603 **Score:** 58 **Matches:** 1(1) **Sequences:** 1(1)
>tr|B0YXC9|B0YXC9_9HIV1 Envelope glycoprotein gp160 OS=Human immunodeficiency virus 1 OX=11676 GN=env PE=3 SV=1
2::B0YXD3 **Mass:** 96736 **Score:** 58 **Matches:** 1(1) **Sequences:** 1(1)
>tr|B0YXD3|B0YXD3_9HIV1 Envelope glycoprotein gp160 OS=Human immunodeficiency virus 1 OX=11676 GN=env PE=3 SV=1
2::D0VLR6 **Mass:** 98133 **Score:** 58 **Matches:** 1(1) **Sequences:** 1(1)
>tr|D0VLR6|D0VLR6_9HIV1 Envelope glycoprotein gp160 OS=Human immunodeficiency virus 1 OX=11676 GN=env PE=3 SV=1
2::D0VLS2 **Mass:** 98257 **Score:** 58 **Matches:** 1(1) **Sequences:** 1(1)
>tr|D0VLS2|D0VLS2_9HIV1 Envelope glycoprotein gp160 OS=Human immunodeficiency virus 1 OX=11676 GN=env PE=3 SV=1
2::D0VLT0 **Mass:** 98544 **Score:** 58 **Matches:** 1(1) **Sequences:** 1(1)
>tr|D0VLT0|D0VLT0_9HIV1 Envelope glycoprotein gp160 OS=Human immunodeficiency virus 1 OX=11676 GN=env PE=3 SV=1
2::G0WNZ5 **Mass:** 97619 **Score:** 58 **Matches:** 1(1) **Sequences:** 1(1)
>tr|G0WNZ5|G0WNZ5_9HIV1 Envelope glycoprotein gp160 OS=Human immunodeficiency virus 1 OX=11676 GN=env PE=3 SV=1
2::G0WP03 **Mass:** 98795 **Score:** 58 **Matches:** 1(1) **Sequences:** 1(1)
>tr|G0WP03|G0WP03_9HIV1 Envelope glycoprotein gp160 OS=Human immunodeficiency virus 1 OX=11676 GN=env PE=3 SV=1
2::G0WP09 **Mass:** 97743 **Score:** 58 **Matches:** 1(1) **Sequences:** 1(1)
>tr|G0WP09|G0WP09_9HIV1 Envelope glycoprotein gp160 OS=Human immunodeficiency virus 1 OX=11676 GN=env PE=3 SV=1
2::G0WP20 **Mass:** 100869 **Score:** 58 **Matches:** 1(1) **Sequences:** 1(1)
>tr|G0WP20|G0WP20_9HIV1 Envelope glycoprotein gp160 OS=Human immunodeficiency virus 1 OX=11676 GN=env PE=3 SV=1
2::G0WP23 **Mass:** 97712 **Score:** 58 **Matches:** 1(1) **Sequences:** 1(1)
>tr|G0WP23|G0WP23_9HIV1 Envelope glycoprotein gp160 OS=Human immunodeficiency virus 1 OX=11676 GN=env PE=3 SV=1
2::G0WP44 **Mass:** 97604 **Score:** 58 **Matches:** 1(1) **Sequences:** 1(1)
>tr|G0WP44|G0WP44_9HIV1 Envelope glycoprotein gp160 OS=Human immunodeficiency virus 1 OX=11676 GN=env PE=3 SV=1
2::G0WP45 **Mass:** 97202 **Score:** 58 **Matches:** 1(1) **Sequences:** 1(1)
>tr|G0WP45|G0WP45_9HIV1 Envelope glycoprotein gp160 OS=Human immunodeficiency virus 1 OX=11676 GN=env PE=3 SV=1
2::L0HQV6 **Mass:** 95410 **Score:** 58 **Matches:** 1(1) **Sequences:** 1(1)
>tr|L0HQV6|L0HQV6_9HIV1 Envelope glycoprotein gp160 OS=Human immunodeficiency virus 1 OX=11676 GN=env PE=3 SV=1
2::C1KK43 **Mass:** 97661 **Score:** 58 **Matches:** 1(1) **Sequences:** 1(1)
>tr|C1KK43|C1KK43_9HIV1 Envelope glycoprotein gp160 OS=Human immunodeficiency virus 1 OX=11676 GN=env PE=3 SV=1
2::C1KK44 **Mass:** 97826 **Score:** 58 **Matches:** 1(1) **Sequences:** 1(1)
>tr|C1KK44|C1KK44_9HIV1 Envelope glycoprotein gp160 OS=Human immunodeficiency virus 1 OX=11676 GN=env PE=3 SV=1
2::C1KK45 **Mass:** 97582 **Score:** 58 **Matches:** 1(1) **Sequences:** 1(1)
>tr|C1KK45|C1KK45_9HIV1 Envelope glycoprotein gp160 OS=Human immunodeficiency virus 1 OX=11676 GN=env PE=3 SV=1
2::C1KK46 **Mass:** 97763 **Score:** 58 **Matches:** 1(1) **Sequences:** 1(1)
>tr|C1KK46|C1KK46_9HIV1 Envelope glycoprotein gp160 OS=Human immunodeficiency virus 1 OX=11676 GN=env PE=3 SV=1
2::C1KK47 **Mass:** 97669 **Score:** 58 **Matches:** 1(1) **Sequences:** 1(1)
>tr|C1KK47|C1KK47_9HIV1 Envelope glycoprotein gp160 OS=Human immunodeficiency virus 1 OX=11676 GN=env PE=3 SV=1
2::E1AEE3 **Mass:** 96413 **Score:** 58 **Matches:** 1(1) **Sequences:** 1(1)
>tr|E1AEE3|E1AEE3_9HIV1 Envelope glycoprotein gp160 OS=Human immunodeficiency virus 1 OX=11676 GN=env PE=3 SV=1
2::E1AEE4 **Mass:** 97144 **Score:** 58 **Matches:** 1(1) **Sequences:** 1(1)
>tr|E1AEE4|E1AEE4_9HIV1 Envelope glycoprotein gp160 OS=Human immunodeficiency virus 1 OX=11676 GN=env PE=3 SV=1
2::E1AEE5 **Mass:** 98300 **Score:** 58 **Matches:** 1(1) **Sequences:** 1(1)
>tr|E1AEE5|E1AEE5_9HIV1 Envelope glycoprotein gp160 OS=Human immunodeficiency virus 1 OX=11676 GN=env PE=3 SV=1
2::E1AEE6 **Mass:** 96866 **Score:** 58 **Matches:** 1(1) **Sequences:** 1(1)
>tr|E1AEE6|E1AEE6_9HIV1 Envelope glycoprotein gp160 OS=Human immunodeficiency virus 1 OX=11676 GN=env PE=3 SV=1
2::E1AEE7 **Mass:** 97316 **Score:** 58 **Matches:** 1(1) **Sequences:** 1(1)
>tr|E1AEE7|E1AEE7_9HIV1 Envelope glycoprotein gp160 OS=Human immunodeficiency virus 1 OX=11676 GN=env PE=3 SV=1
2::E1AEE8 **Mass:** 97095 **Score:** 58 **Matches:** 1(1) **Sequences:** 1(1)
>tr|E1AEE8|E1AEE8_9HIV1 Envelope glycoprotein gp160 OS=Human immunodeficiency virus 1 OX=11676 GN=env PE=3 SV=1
2::E1AEF1 **Mass:** 96385 **Score:** 58 **Matches:** 1(1) **Sequences:** 1(1)
>tr|E1AEF1|E1AEF1_9HIV1 Envelope glycoprotein gp160 OS=Human immunodeficiency virus 1 OX=11676 GN=env PE=3 SV=1
2::E1AEF2 **Mass:** 97296 **Score:** 58 **Matches:** 1(1) **Sequences:** 1(1)
>tr|E1AEF2|E1AEF2_9HIV1 Envelope glycoprotein gp160 OS=Human immunodeficiency virus 1 OX=11676 GN=env PE=3 SV=1
2::E1AEF3 **Mass:** 97348 **Score:** 58 **Matches:** 1(1) **Sequences:** 1(1)
>tr|E1AEF3|E1AEF3_9HIV1 Envelope glycoprotein gp160 OS=Human immunodeficiency virus 1 OX=11676 GN=env PE=3 SV=1
2::E1AEF4 **Mass:** 97316 **Score:** 58 **Matches:** 1(1) **Sequences:** 1(1)
>tr|E1AEF4|E1AEF4_9HIV1 Envelope glycoprotein gp160 OS=Human immunodeficiency virus 1 OX=11676 GN=env PE=3 SV=1
2::E1AEF5 **Mass:** 96556 **Score:** 58 **Matches:** 1(1) **Sequences:** 1(1)
>tr|E1AEF5|E1AEF5_9HIV1 Envelope glycoprotein gp160 OS=Human immunodeficiency virus 1 OX=11676 GN=env PE=3 SV=1
2::E1AEF6 **Mass:** 98254 **Score:** 58 **Matches:** 1(1) **Sequences:** 1(1)
>tr|E1AEF6|E1AEF6_9HIV1 Envelope glycoprotein gp160 OS=Human immunodeficiency virus 1 OX=11676 GN=env PE=3 SV=1
2::E1AEF7 **Mass:** 97828 **Score:** 58 **Matches:** 1(1) **Sequences:** 1(1)
>tr|E1AEF7|E1AEF7_9HIV1 Envelope glycoprotein gp160 OS=Human immunodeficiency virus 1 OX=11676 GN=env PE=3 SV=1
2::E1AEF9 **Mass:** 97342 **Score:** 58 **Matches:** 1(1) **Sequences:** 1(1)
>tr|E1AEF9|E1AEF9_9HIV1 Envelope glycoprotein gp160 OS=Human immunodeficiency virus 1 OX=11676 GN=env PE=3 SV=1
2::E1AEG1 **Mass:** 97903 **Score:** 58 **Matches:** 1(1) **Sequences:** 1(1)
>tr|E1AEG1|E1AEG1_9HIV1 Envelope glycoprotein gp160 OS=Human immunodeficiency virus 1 OX=11676 GN=env PE=3 SV=1
2::E1AEG2 **Mass:** 97479 **Score:** 58 **Matches:** 1(1) **Sequences:** 1(1)
>tr|E1AEG2|E1AEG2_9HIV1 Envelope glycoprotein gp160 OS=Human immunodeficiency virus 1 OX=11676 GN=env PE=3 SV=1
2::E1AEG4 **Mass:** 97123 **Score:** 58 **Matches:** 1(1) **Sequences:** 1(1)
>tr|E1AEG4|E1AEG4_9HIV1 Envelope glycoprotein gp160 OS=Human immunodeficiency virus 1 OX=11676 GN=env PE=3 SV=1
2::E1AEG5 **Mass:** 97698 **Score:** 58 **Matches:** 1(1) **Sequences:** 1(1)
>tr|E1AEG5|E1AEG5_9HIV1 Envelope glycoprotein gp160 OS=Human immunodeficiency virus 1 OX=11676 GN=env PE=3 SV=1
2::E1AEG8 **Mass:** 97917 **Score:** 58 **Matches:** 1(1) **Sequences:** 1(1)
>tr|E1AEG8|E1AEG8_9HIV1 Envelope glycoprotein gp160 OS=Human immunodeficiency virus 1 OX=11676 GN=env PE=3 SV=1
2::E1AEH0 **Mass:** 97614 **Score:** 58 **Matches:** 1(1) **Sequences:** 1(1)
>tr|E1AEH0|E1AEH0_9HIV1 Envelope glycoprotein gp160 OS=Human immunodeficiency virus 1 OX=11676 GN=env PE=3 SV=1
2::E1AEH1 **Mass:** 97626 **Score:** 58 **Matches:** 1(1) **Sequences:** 1(1)
>tr|E1AEH1|E1AEH1_9HIV1 Envelope glycoprotein gp160 OS=Human immunodeficiency virus 1 OX=11676 GN=env PE=3 SV=1
2::E1AEH3 **Mass:** 97122 **Score:** 58 **Matches:** 1(1) **Sequences:** 1(1)
>tr|E1AEH3|E1AEH3_9HIV1 Envelope glycoprotein gp160 OS=Human immunodeficiency virus 1 OX=11676 GN=env PE=3 SV=1
2::E1AEH4 **Mass:** 97543 **Score:** 58 **Matches:** 1(1) **Sequences:** 1(1)
>tr|E1AEH4|E1AEH4_9HIV1 Envelope glycoprotein gp160 OS=Human immunodeficiency virus 1 OX=11676 GN=env PE=3 SV=1
2::E1AEH5 **Mass:** 97024 **Score:** 58 **Matches:** 1(1) **Sequences:** 1(1)
>tr|E1AEH5|E1AEH5_9HIV1 Envelope glycoprotein gp160 OS=Human immunodeficiency virus 1 OX=11676 GN=env PE=3 SV=1
2::E1AEH8 **Mass:** 97857 **Score:** 58 **Matches:** 1(1) **Sequences:** 1(1)
>tr|E1AEH8|E1AEH8_9HIV1 Envelope glycoprotein gp160 OS=Human immunodeficiency virus 1 OX=11676 GN=env PE=3 SV=1
2::E1AEI1 **Mass:** 97063 **Score:** 58 **Matches:** 1(1) **Sequences:** 1(1)
>tr|E1AEI1|E1AEI1_9HIV1 Envelope glycoprotein gp160 OS=Human immunodeficiency virus 1 OX=11676 GN=env PE=3 SV=1

2::E1AEI4 **Mass:** 97972 **Score:** 58 **Matches:** 1(1) **Sequences:** 1(1)
>tr|E1AEI4|E1AEI4_9HIV1 Envelope glycoprotein gp160 OS=Human immunodeficiency virus 1 OX=11676 GN=env PE=3 SV=1
2::E1AEI5 **Mass:** 97144 **Score:** 58 **Matches:** 1(1) **Sequences:** 1(1)
>tr|E1AEI5|E1AEI5_9HIV1 Envelope glycoprotein gp160 OS=Human immunodeficiency virus 1 OX=11676 GN=env PE=3 SV=1
2::E1AEI7 **Mass:** 98288 **Score:** 58 **Matches:** 1(1) **Sequences:** 1(1)
>tr|E1AEI7|E1AEI7_9HIV1 Envelope glycoprotein gp160 OS=Human immunodeficiency virus 1 OX=11676 GN=env PE=3 SV=1
2::E1AEI8 **Mass:** 96881 **Score:** 58 **Matches:** 1(1) **Sequences:** 1(1)
>tr|E1AEI8|E1AEI8_9HIV1 Envelope glycoprotein gp160 OS=Human immunodeficiency virus 1 OX=11676 GN=env PE=3 SV=1
2::E1AEI9 **Mass:** 97320 **Score:** 58 **Matches:** 1(1) **Sequences:** 1(1)
>tr|E1AEI9|E1AEI9_9HIV1 Envelope glycoprotein gp160 OS=Human immunodeficiency virus 1 OX=11676 GN=env PE=3 SV=1
2::E1AEJ0 **Mass:** 97686 **Score:** 58 **Matches:** 1(1) **Sequences:** 1(1)
>tr|E1AEJ0|E1AEJ0_9HIV1 Envelope glycoprotein gp160 OS=Human immunodeficiency virus 1 OX=11676 GN=env PE=3 SV=1
2::E1AEJ1 **Mass:** 97648 **Score:** 58 **Matches:** 1(1) **Sequences:** 1(1)
>tr|E1AEJ1|E1AEJ1_9HIV1 Envelope glycoprotein gp160 OS=Human immunodeficiency virus 1 OX=11676 GN=env PE=3 SV=1
2::E1AEJ2 **Mass:** 97621 **Score:** 58 **Matches:** 1(1) **Sequences:** 1(1)
>tr|E1AEJ2|E1AEJ2_9HIV1 Envelope glycoprotein gp160 OS=Human immunodeficiency virus 1 OX=11676 GN=env PE=3 SV=1
2::E1AEJ3 **Mass:** 97570 **Score:** 58 **Matches:** 1(1) **Sequences:** 1(1)
>tr|E1AEJ3|E1AEJ3_9HIV1 Envelope glycoprotein gp160 OS=Human immunodeficiency virus 1 OX=11676 GN=env PE=3 SV=1
2::E1AEJ4 **Mass:** 96851 **Score:** 58 **Matches:** 1(1) **Sequences:** 1(1)
>tr|E1AEJ4|E1AEJ4_9HIV1 Envelope glycoprotein gp160 OS=Human immunodeficiency virus 1 OX=11676 GN=env PE=3 SV=1
2::E1AEJ5 **Mass:** 97411 **Score:** 58 **Matches:** 1(1) **Sequences:** 1(1)
>tr|E1AEJ5|E1AEJ5_9HIV1 Envelope glycoprotein gp160 OS=Human immunodeficiency virus 1 OX=11676 GN=env PE=3 SV=1
2::E1AEJ6 **Mass:** 98137 **Score:** 58 **Matches:** 1(1) **Sequences:** 1(1)
>tr|E1AEJ6|E1AEJ6_9HIV1 Envelope glycoprotein gp160 OS=Human immunodeficiency virus 1 OX=11676 GN=env PE=3 SV=1
2::E1AEJ7 **Mass:** 97156 **Score:** 58 **Matches:** 1(1) **Sequences:** 1(1)
>tr|E1AEJ7|E1AEJ7_9HIV1 Envelope glycoprotein gp160 OS=Human immunodeficiency virus 1 OX=11676 GN=env PE=3 SV=1
2::E1AEJ8 **Mass:** 97781 **Score:** 58 **Matches:** 1(1) **Sequences:** 1(1)
>tr|E1AEJ8|E1AEJ8_9HIV1 Envelope glycoprotein gp160 OS=Human immunodeficiency virus 1 OX=11676 GN=env PE=3 SV=1
2::E1AEJ9 **Mass:** 97201 **Score:** 58 **Matches:** 1(1) **Sequences:** 1(1)
>tr|E1AEJ9|E1AEJ9_9HIV1 Envelope glycoprotein gp160 OS=Human immunodeficiency virus 1 OX=11676 GN=env PE=3 SV=1
2::E1AEK1 **Mass:** 96974 **Score:** 58 **Matches:** 1(1) **Sequences:** 1(1)
>tr|E1AEK1|E1AEK1_9HIV1 Envelope glycoprotein gp160 OS=Human immunodeficiency virus 1 OX=11676 GN=env PE=3 SV=1
2::E1AEK2 **Mass:** 97597 **Score:** 58 **Matches:** 1(1) **Sequences:** 1(1)
>tr|E1AEK2|E1AEK2_9HIV1 Envelope glycoprotein gp160 OS=Human immunodeficiency virus 1 OX=11676 GN=env PE=3 SV=1
2::E1AEK3 **Mass:** 96829 **Score:** 58 **Matches:** 1(1) **Sequences:** 1(1)
>tr|E1AEK3|E1AEK3_9HIV1 Envelope glycoprotein gp160 OS=Human immunodeficiency virus 1 OX=11676 GN=env PE=3 SV=1
2::E1AEK4 **Mass:** 97711 **Score:** 58 **Matches:** 1(1) **Sequences:** 1(1)
>tr|E1AEK4|E1AEK4_9HIV1 Envelope glycoprotein gp160 OS=Human immunodeficiency virus 1 OX=11676 GN=env PE=3 SV=1
2::E1AEK6 **Mass:** 97367 **Score:** 58 **Matches:** 1(1) **Sequences:** 1(1)
>tr|E1AEK6|E1AEK6_9HIV1 Envelope glycoprotein gp160 OS=Human immunodeficiency virus 1 OX=11676 GN=env PE=3 SV=1
2::E1AEK7 **Mass:** 97266 **Score:** 58 **Matches:** 1(1) **Sequences:** 1(1)
>tr|E1AEK7|E1AEK7_9HIV1 Envelope glycoprotein gp160 OS=Human immunodeficiency virus 1 OX=11676 GN=env PE=3 SV=1
2::E1AEK8 **Mass:** 97084 **Score:** 58 **Matches:** 1(1) **Sequences:** 1(1)
>tr|E1AEK8|E1AEK8_9HIV1 Envelope glycoprotein gp160 OS=Human immunodeficiency virus 1 OX=11676 GN=env PE=3 SV=1
2::E1AEK9 **Mass:** 97884 **Score:** 58 **Matches:** 1(1) **Sequences:** 1(1)
>tr|E1AEK9|E1AEK9_9HIV1 Envelope glycoprotein gp160 OS=Human immunodeficiency virus 1 OX=11676 GN=env PE=3 SV=1
2::E1AEL0 **Mass:** 97661 **Score:** 58 **Matches:** 1(1) **Sequences:** 1(1)
>tr|E1AEL0|E1AEL0_9HIV1 Envelope glycoprotein gp160 OS=Human immunodeficiency virus 1 OX=11676 GN=env PE=3 SV=1
2::E1AEL2 **Mass:** 98197 **Score:** 58 **Matches:** 1(1) **Sequences:** 1(1)
>tr|E1AEL2|E1AEL2_9HIV1 Envelope glycoprotein gp160 OS=Human immunodeficiency virus 1 OX=11676 GN=env PE=3 SV=1
2::E1AEL3 **Mass:** 96784 **Score:** 58 **Matches:** 1(1) **Sequences:** 1(1)
>tr|E1AEL3|E1AEL3_9HIV1 Envelope glycoprotein gp160 OS=Human immunodeficiency virus 1 OX=11676 GN=env PE=3 SV=1
2::E1AEL4 **Mass:** 97114 **Score:** 58 **Matches:** 1(1) **Sequences:** 1(1)
>tr|E1AEL4|E1AEL4_9HIV1 Envelope glycoprotein gp160 OS=Human immunodeficiency virus 1 OX=11676 GN=env PE=3 SV=1
2::E1AEL5 **Mass:** 96931 **Score:** 58 **Matches:** 1(1) **Sequences:** 1(1)
>tr|E1AEL5|E1AEL5_9HIV1 Envelope glycoprotein gp160 OS=Human immunodeficiency virus 1 OX=11676 GN=env PE=3 SV=1
2::E1AEL6 **Mass:** 97163 **Score:** 58 **Matches:** 1(1) **Sequences:** 1(1)
>tr|E1AEL6|E1AEL6_9HIV1 Envelope glycoprotein gp160 OS=Human immunodeficiency virus 1 OX=11676 GN=env PE=3 SV=1
2::E1AEL7 **Mass:** 97620 **Score:** 58 **Matches:** 1(1) **Sequences:** 1(1)
>tr|E1AEL7|E1AEL7_9HIV1 Envelope glycoprotein gp160 OS=Human immunodeficiency virus 1 OX=11676 GN=env PE=3 SV=1
2::M1K578 **Mass:** 98737 **Score:** 58 **Matches:** 1(1) **Sequences:** 1(1)
>tr|M1K578|M1K578_9HIV1 Envelope glycoprotein gp160 OS=Human immunodeficiency virus 1 OX=11676 GN=env PE=3 SV=1
2::M1KQF9 **Mass:** 98766 **Score:** 58 **Matches:** 1(1) **Sequences:** 1(1)
>tr|M1KQF9|M1KQF9_9HIV1 Envelope glycoprotein gp160 OS=Human immunodeficiency virus 1 OX=11676 GN=env PE=3 SV=1
2::M1KQK0 **Mass:** 97118 **Score:** 58 **Matches:** 1(1) **Sequences:** 1(1)
>tr|M1KQK0|M1KQK0_9HIV1 Envelope glycoprotein gp160 OS=Human immunodeficiency virus 1 OX=11676 GN=env PE=3 SV=1
2::M1L091 **Mass:** 97574 **Score:** 58 **Matches:** 1(1) **Sequences:** 1(1)
>tr|M1L091|M1L091_9HIV1 Envelope glycoprotein gp160 OS=Human immunodeficiency virus 1 OX=11676 GN=env PE=3 SV=1
2::M1LBP6 **Mass:** 97992 **Score:** 58 **Matches:** 1(1) **Sequences:** 1(1)
>tr|M1LBP6|M1LBP6_9HIV1 Envelope glycoprotein gp160 OS=Human immunodeficiency virus 1 OX=11676 GN=env PE=3 SV=1
2::Q1KW40 **Mass:** 97944 **Score:** 58 **Matches:** 1(1) **Sequences:** 1(1)
>tr|Q1KW40|Q1KW40_9HIV1 Envelope glycoprotein gp160 OS=Human immunodeficiency virus 1 OX=11676 GN=env PE=3 SV=1
2::Q1KWJ9 **Mass:** 98451 **Score:** 58 **Matches:** 1(1) **Sequences:** 1(1)
>tr|Q1KWJ9|Q1KWJ9_9HIV1 Envelope glycoprotein gp160 OS=Human immunodeficiency virus 1 OX=11676 GN=env PE=3 SV=1
2::Q1KWT9 **Mass:** 98633 **Score:** 58 **Matches:** 1(1) **Sequences:** 1(1)
>tr|Q1KWT9|Q1KWT9_9HIV1 Envelope glycoprotein gp160 OS=Human immunodeficiency virus 1 OX=11676 GN=env PE=3 SV=1
2::Q1PDE4 **Mass:** 100375 **Score:** 58 **Matches:** 1(1) **Sequences:** 1(1)
>tr|Q1PDE4|Q1PDE4_9HIV1 Envelope glycoprotein gp160 OS=Human immunodeficiency virus 1 OX=11676 GN=env PE=3 SV=1
2::D2ISZ7 **Mass:** 98829 **Score:** 58 **Matches:** 1(1) **Sequences:** 1(1)
>tr|D2ISZ7|D2ISZ7_9HIV1 Envelope glycoprotein gp160 OS=Human immunodeficiency virus 1 OX=11676 GN=env PE=3 SV=1
2::D2X6P2 **Mass:** 99586 **Score:** 58 **Matches:** 1(1) **Sequences:** 1(1)
>tr|D2X6P2|D2X6P2_9HIV1 Envelope glycoprotein gp160 OS=Human immunodeficiency virus 1 OX=11676 GN=env PE=3 SV=1
2::Q2EQE3 **Mass:** 99497 **Score:** 58 **Matches:** 1(1) **Sequences:** 1(1)
>tr|Q2EQE3|Q2EQE3_9HIV1 Envelope glycoprotein gp160 OS=Human immunodeficiency virus 1 OX=11676 GN=env PE=3 SV=1
2::Q2L7N9 **Mass:** 97375 **Score:** 58 **Matches:** 1(1) **Sequences:** 1(1)
>tr|Q2L7N9|Q2L7N9_9HIV1 Envelope glycoprotein gp160 OS=Human immunodeficiency virus 1 OX=11676 GN=env PE=3 SV=1
2::Q2L841 **Mass:** 97544 **Score:** 58 **Matches:** 1(1) **Sequences:** 1(1)
>tr|Q2L841|Q2L841_9HIV1 Envelope glycoprotein gp160 OS=Human immunodeficiency virus 1 OX=11676 GN=env PE=3 SV=1
2::Q2VAP7 **Mass:** 98619 **Score:** 58 **Matches:** 1(1) **Sequences:** 1(1)
>tr|Q2VAP7|Q2VAP7_9HIV1 Envelope glycoprotein gp160 OS=Human immunodeficiency virus 1 OX=11676 GN=env PE=3 SV=1
2::T2CI98 **Mass:** 98123 **Score:** 58 **Matches:** 1(1) **Sequences:** 1(1)
>tr|T2CI98|T2CI98_9HIV1 Envelope glycoprotein gp160 OS=Human immunodeficiency virus 1 OX=11676 GN=env PE=3 SV=1
2::T2CII4 **Mass:** 97211 **Score:** 58 **Matches:** 1(1) **Sequences:** 1(1)
>tr|T2CII4|T2CII4_9HIV1 Envelope glycoprotein gp160 OS=Human immunodeficiency virus 1 OX=11676 GN=env PE=3 SV=1
2::T2CIQ1 **Mass:** 97575 **Score:** 58 **Matches:** 1(1) **Sequences:** 1(1)
>tr|T2CIQ1|T2CIQ1_9HIV1 Envelope glycoprotein gp160 OS=Human immunodeficiency virus 1 OX=11676 GN=env PE=3 SV=1
2::T2CJA5 **Mass:** 98298 **Score:** 58 **Matches:** 1(1) **Sequences:** 1(1)
>tr|T2CJA5|T2CJA5_9HIV1 Envelope glycoprotein gp160 OS=Human immunodeficiency virus 1 OX=11676 GN=env PE=3 SV=1
2::T2CN10 **Mass:** 98682 **Score:** 58 **Matches:** 1(1) **Sequences:** 1(1)
>tr|T2CN10|T2CN10_9HIV1 Envelope glycoprotein gp160 OS=Human immunodeficiency virus 1 OX=11676 GN=env PE=3 SV=1
2::T2CNI3 **Mass:** 100321 **Score:** 58 **Matches:** 1(1) **Sequences:** 1(1)
>tr|T2CNI3|T2CNI3_9HIV1 Envelope glycoprotein gp160 OS=Human immunodeficiency virus 1 OX=11676 GN=env PE=3 SV=1
2::T2CPN3 **Mass:** 100275 **Score:** 58 **Matches:** 1(1) **Sequences:** 1(1)
>tr|T2CPN3|T2CPN3_9HIV1 Envelope glycoprotein gp160 OS=Human immunodeficiency virus 1 OX=11676 GN=env PE=3 SV=1
2::T2CPP7 **Mass:** 97714 **Score:** 58 **Matches:** 1(1) **Sequences:** 1(1)
>tr|T2CPP7|T2CPP7_9HIV1 Envelope glycoprotein gp160 OS=Human immunodeficiency virus 1 OX=11676 GN=env PE=3 SV=1
2::T2CPQ3 **Mass:** 98529 **Score:** 58 **Matches:** 1(1) **Sequences:** 1(1)
>tr|T2CPQ3|T2CPQ3_9HIV1 Envelope glycoprotein gp160 OS=Human immunodeficiency virus 1 OX=11676 GN=env PE=3 SV=1
2::T2CPT0 **Mass:** 100252 **Score:** 58 **Matches:** 1(1) **Sequences:** 1(1)
>tr|T2CPT0|T2CPT0_9HIV1 Envelope glycoprotein gp160 OS=Human immunodeficiency virus 1 OX=11676 GN=env PE=3 SV=1

```
2::T2CQ99    Mass: 100496   Score: 58     Matches: 1(1)  Sequences: 1(1)
>tr|T2CQ99|T2CQ99_9HIV1 Envelope glycoprotein gp160 OS=Human immunodeficiency virus 1 OX=11676 GN=env PE=3 SV=1
2::T2CQY7    Mass: 99303    Score: 58     Matches: 1(1)  Sequences: 1(1)
>tr|T2CQY7|T2CQY7_9HIV1 Envelope glycoprotein gp160 OS=Human immunodeficiency virus 1 OX=11676 GN=env PE=3 SV=1
2::T2CYM6    Mass: 98761    Score: 58     Matches: 1(1)  Sequences: 1(1)
>tr|T2CYM6|T2CYM6_9HIV1 Envelope glycoprotein gp160 OS=Human immunodeficiency virus 1 OX=11676 GN=env PE=3 SV=1
2::A3QWF0    Mass: 96946    Score: 58     Matches: 1(1)  Sequences: 1(1)
>tr|A3QWF0|A3QWF0_9HIV1 Envelope glycoprotein gp160 (Fragment) OS=Human immunodeficiency virus 1 OX=11676 GN=env PE=3 SV=1
2::A3QWF7    Mass: 97551    Score: 58     Matches: 1(1)  Sequences: 1(1)
>tr|A3QWF7|A3QWF7_9HIV1 Envelope glycoprotein gp160 (Fragment) OS=Human immunodeficiency virus 1 OX=11676 GN=env PE=3 SV=1
2::A3QWF8    Mass: 97517    Score: 58     Matches: 1(1)  Sequences: 1(1)
>tr|A3QWF8|A3QWF8_9HIV1 Envelope glycoprotein gp160 (Fragment) OS=Human immunodeficiency virus 1 OX=11676 GN=env PE=3 SV=1
2::A3QWF9    Mass: 98368    Score: 58     Matches: 1(1)  Sequences: 1(1)
>tr|A3QWF9|A3QWF9_9HIV1 Envelope glycoprotein gp160 (Fragment) OS=Human immunodeficiency virus 1 OX=11676 GN=env PE=3 SV=1
2::A3QWG0    Mass: 98366    Score: 58     Matches: 1(1)  Sequences: 1(1)
>tr|A3QWG0|A3QWG0_9HIV1 Envelope glycoprotein gp160 (Fragment) OS=Human immunodeficiency virus 1 OX=11676 GN=env PE=3 SV=1
2::A3QWG1    Mass: 97386    Score: 58     Matches: 1(1)  Sequences: 1(1)
>tr|A3QWG1|A3QWG1_9HIV1 Envelope glycoprotein gp160 (Fragment) OS=Human immunodeficiency virus 1 OX=11676 GN=env PE=3 SV=1
2::A3QWG2    Mass: 97528    Score: 58     Matches: 1(1)  Sequences: 1(1)
>tr|A3QWG2|A3QWG2_9HIV1 Envelope glycoprotein gp160 (Fragment) OS=Human immunodeficiency virus 1 OX=11676 GN=env PE=3 SV=1
2::A3QWG3    Mass: 97646    Score: 58     Matches: 1(1)  Sequences: 1(1)
>tr|A3QWG3|A3QWG3_9HIV1 Envelope glycoprotein gp160 (Fragment) OS=Human immunodeficiency virus 1 OX=11676 GN=env PE=3 SV=1
2::C3U4F1    Mass: 96432    Score: 58     Matches: 1(1)  Sequences: 1(1)
>tr|C3U4F1|C3U4F1_9HIV1 Envelope glycoprotein gp160 OS=Human immunodeficiency virus 1 OX=11676 GN=env PE=3 SV=1
2::C3U4G4    Mass: 96450    Score: 58     Matches: 1(1)  Sequences: 1(1)
>tr|C3U4G4|C3U4G4_9HIV1 Envelope glycoprotein gp160 OS=Human immunodeficiency virus 1 OX=11676 GN=env PE=3 SV=1
2::C3U4G6    Mass: 96431    Score: 58     Matches: 1(1)  Sequences: 1(1)
>tr|C3U4G6|C3U4G6_9HIV1 Envelope glycoprotein gp160 OS=Human immunodeficiency virus 1 OX=11676 GN=env PE=3 SV=1
2::D3GFS3    Mass: 97936    Score: 58     Matches: 1(1)  Sequences: 1(1)
>tr|D3GFS3|D3GFS3_9HIV1 Envelope glycoprotein gp160 OS=Human immunodeficiency virus 1 OX=11676 GN=env PE=3 SV=2
2::D3X561    Mass: 99368    Score: 58     Matches: 1(1)  Sequences: 1(1)
>tr|D3X561|D3X561_9HIV1 Envelope glycoprotein gp160 OS=Human immunodeficiency virus 1 OX=11676 GN=env PE=3 SV=1
2::Q3S5W7    Mass: 98837    Score: 58     Matches: 1(1)  Sequences: 1(1)
>tr|Q3S5W7|Q3S5W7_9HIV1 Envelope glycoprotein gp160 OS=Human immunodeficiency virus 1 OX=11676 GN=env PE=3 SV=1
2::U3MY28    Mass: 96952    Score: 58     Matches: 1(1)  Sequences: 1(1)
>tr|U3MY28|U3MY28_9HIV1 Envelope glycoprotein gp160 OS=Human immunodeficiency virus 1 OX=11676 GN=env PE=3 SV=1
2::U3MYB4    Mass: 96901    Score: 58     Matches: 1(1)  Sequences: 1(1)
>tr|U3MYB4|U3MYB4_9HIV1 Envelope glycoprotein gp160 OS=Human immunodeficiency virus 1 OX=11676 GN=env PE=3 SV=1
2::U3MZR6    Mass: 96920    Score: 58     Matches: 1(1)  Sequences: 1(1)
>tr|U3MZR6|U3MZR6_9HIV1 Envelope glycoprotein gp160 OS=Human immunodeficiency virus 1 OX=11676 GN=env PE=3 SV=1
2::U3N0I6    Mass: 97114    Score: 58     Matches: 1(1)  Sequences: 1(1)
>tr|U3N0I6|U3N0I6_9HIV1 Envelope glycoprotein gp160 OS=Human immunodeficiency virus 1 OX=11676 GN=env PE=3 SV=1
2::U3N551    Mass: 96940    Score: 58     Matches: 1(1)  Sequences: 1(1)
>tr|U3N551|U3N551_9HIV1 Envelope glycoprotein gp160 OS=Human immunodeficiency virus 1 OX=11676 GN=env PE=3 SV=1
2::D4NUI1    Mass: 98143    Score: 58     Matches: 1(1)  Sequences: 1(1)
>tr|D4NUI1|D4NUI1_9HIV1 Envelope glycoprotein gp160 OS=Human immunodeficiency virus 1 OX=11676 GN=env PE=3 SV=1
2::D4NUI2    Mass: 98269    Score: 58     Matches: 1(1)  Sequences: 1(1)
>tr|D4NUI2|D4NUI2_9HIV1 Envelope glycoprotein gp160 OS=Human immunodeficiency virus 1 OX=11676 GN=env PE=3 SV=1
2::D4NUI3    Mass: 97907    Score: 58     Matches: 1(1)  Sequences: 1(1)
>tr|D4NUI3|D4NUI3_9HIV1 Envelope glycoprotein gp160 OS=Human immunodeficiency virus 1 OX=11676 GN=env PE=3 SV=1
2::D4NUI5    Mass: 98018    Score: 58     Matches: 1(1)  Sequences: 1(1)
>tr|D4NUI5|D4NUI5_9HIV1 Envelope glycoprotein gp160 OS=Human immunodeficiency virus 1 OX=11676 GN=env PE=3 SV=1
2::D4NUI6    Mass: 97726    Score: 58     Matches: 1(1)  Sequences: 1(1)
>tr|D4NUI6|D4NUI6_9HIV1 Envelope glycoprotein gp160 OS=Human immunodeficiency virus 1 OX=11676 GN=env PE=3 SV=1
2::D4NUI7    Mass: 97728    Score: 58     Matches: 1(1)  Sequences: 1(1)
>tr|D4NUI7|D4NUI7_9HIV1 Envelope glycoprotein gp160 OS=Human immunodeficiency virus 1 OX=11676 GN=env PE=3 SV=1
2::D4NUI8    Mass: 98033    Score: 58     Matches: 1(1)  Sequences: 1(1)
>tr|D4NUI8|D4NUI8_9HIV1 Envelope glycoprotein gp160 OS=Human immunodeficiency virus 1 OX=11676 GN=env PE=3 SV=1
2::D4NUI9    Mass: 98389    Score: 58     Matches: 1(1)  Sequences: 1(1)
>tr|D4NUI9|D4NUI9_9HIV1 Envelope glycoprotein gp160 OS=Human immunodeficiency virus 1 OX=11676 GN=env PE=3 SV=1
2::D4NUJ0    Mass: 97782    Score: 58     Matches: 1(1)  Sequences: 1(1)
>tr|D4NUJ0|D4NUJ0_9HIV1 Envelope glycoprotein gp160 OS=Human immunodeficiency virus 1 OX=11676 GN=env PE=3 SV=1
2::D4NUJ1    Mass: 98376    Score: 58     Matches: 1(1)  Sequences: 1(1)
>tr|D4NUJ1|D4NUJ1_9HIV1 Envelope glycoprotein gp160 OS=Human immunodeficiency virus 1 OX=11676 GN=env PE=3 SV=1
2::D4NUJ2    Mass: 97661    Score: 58     Matches: 1(1)  Sequences: 1(1)
>tr|D4NUJ2|D4NUJ2_9HIV1 Envelope glycoprotein gp160 OS=Human immunodeficiency virus 1 OX=11676 GN=env PE=3 SV=1
2::D4NUJ3    Mass: 97921    Score: 58     Matches: 1(1)  Sequences: 1(1)
>tr|D4NUJ3|D4NUJ3_9HIV1 Envelope glycoprotein gp160 OS=Human immunodeficiency virus 1 OX=11676 GN=env PE=3 SV=1
2::D4NUJ4    Mass: 97643    Score: 58     Matches: 1(1)  Sequences: 1(1)
>tr|D4NUJ4|D4NUJ4_9HIV1 Envelope glycoprotein gp160 OS=Human immunodeficiency virus 1 OX=11676 GN=env PE=3 SV=1
2::D4NUJ5    Mass: 98184    Score: 58     Matches: 1(1)  Sequences: 1(1)
>tr|D4NUJ5|D4NUJ5_9HIV1 Envelope glycoprotein gp160 OS=Human immunodeficiency virus 1 OX=11676 GN=env PE=3 SV=1
2::D4NUJ6    Mass: 98153    Score: 58     Matches: 1(1)  Sequences: 1(1)
>tr|D4NUJ6|D4NUJ6_9HIV1 Envelope glycoprotein gp160 OS=Human immunodeficiency virus 1 OX=11676 GN=env PE=3 SV=1
2::D4NUJ7    Mass: 96949    Score: 58     Matches: 1(1)  Sequences: 1(1)
>tr|D4NUJ7|D4NUJ7_9HIV1 Envelope glycoprotein gp160 OS=Human immunodeficiency virus 1 OX=11676 GN=env PE=3 SV=1
2::D4NUJ8    Mass: 98314    Score: 58     Matches: 1(1)  Sequences: 1(1)
>tr|D4NUJ8|D4NUJ8_9HIV1 Envelope glycoprotein gp160 OS=Human immunodeficiency virus 1 OX=11676 GN=env PE=3 SV=1
2::D4NUJ9    Mass: 97694    Score: 58     Matches: 1(1)  Sequences: 1(1)
>tr|D4NUJ9|D4NUJ9_9HIV1 Envelope glycoprotein gp160 OS=Human immunodeficiency virus 1 OX=11676 GN=env PE=3 SV=1
2::D4NUK0    Mass: 97916    Score: 58     Matches: 1(1)  Sequences: 1(1)
>tr|D4NUK0|D4NUK0_9HIV1 Envelope glycoprotein gp160 OS=Human immunodeficiency virus 1 OX=11676 GN=env PE=3 SV=1
2::D4NUK1    Mass: 98155    Score: 58     Matches: 1(1)  Sequences: 1(1)
>tr|D4NUK1|D4NUK1_9HIV1 Envelope glycoprotein gp160 OS=Human immunodeficiency virus 1 OX=11676 GN=env PE=3 SV=1
2::D4NUK2    Mass: 97626    Score: 58     Matches: 1(1)  Sequences: 1(1)
>tr|D4NUK2|D4NUK2_9HIV1 Envelope glycoprotein gp160 OS=Human immunodeficiency virus 1 OX=11676 GN=env PE=3 SV=1
2::D4NUK3    Mass: 98185    Score: 58     Matches: 1(1)  Sequences: 1(1)
>tr|D4NUK3|D4NUK3_9HIV1 Envelope glycoprotein gp160 OS=Human immunodeficiency virus 1 OX=11676 GN=env PE=3 SV=1
2::D4NUS6    Mass: 96444    Score: 58     Matches: 1(1)  Sequences: 1(1)
>tr|D4NUS6|D4NUS6_9HIV1 Envelope glycoprotein gp160 OS=Human immunodeficiency virus 1 OX=11676 GN=env PE=3 SV=1
2::D4NUS7    Mass: 96616    Score: 58     Matches: 1(1)  Sequences: 1(1)
>tr|D4NUS7|D4NUS7_9HIV1 Envelope glycoprotein gp160 OS=Human immunodeficiency virus 1 OX=11676 GN=env PE=3 SV=1
2::D4NUS9    Mass: 96328    Score: 58     Matches: 1(1)  Sequences: 1(1)
>tr|D4NUS9|D4NUS9_9HIV1 Envelope glycoprotein gp160 OS=Human immunodeficiency virus 1 OX=11676 GN=env PE=3 SV=1
2::D4NUT0    Mass: 96581    Score: 58     Matches: 1(1)  Sequences: 1(1)
>tr|D4NUT0|D4NUT0_9HIV1 Envelope glycoprotein gp160 OS=Human immunodeficiency virus 1 OX=11676 GN=env PE=3 SV=1
2::M4P246    Mass: 98738    Score: 58     Matches: 1(1)  Sequences: 1(1)
>tr|M4P246|M4P246_9HIV1 Envelope glycoprotein gp160 OS=Human immunodeficiency virus 1 OX=11676 GN=env PE=3 SV=1
2::M4P2B9    Mass: 98756    Score: 58     Matches: 1(1)  Sequences: 1(1)
>tr|M4P2B9|M4P2B9_9HIV1 Envelope glycoprotein gp160 OS=Human immunodeficiency virus 1 OX=11676 GN=env PE=3 SV=1
2::M4P2C3    Mass: 98639    Score: 58     Matches: 1(1)  Sequences: 1(1)
>tr|M4P2C3|M4P2C3_9HIV1 Envelope glycoprotein gp160 OS=Human immunodeficiency virus 1 OX=11676 GN=env PE=3 SV=1
2::M4P2C9    Mass: 98763    Score: 58     Matches: 1(1)  Sequences: 1(1)
>tr|M4P2C9|M4P2C9_9HIV1 Envelope glycoprotein gp160 OS=Human immunodeficiency virus 1 OX=11676 GN=env PE=3 SV=1
2::M4P2F2    Mass: 98704    Score: 58     Matches: 1(1)  Sequences: 1(1)
>tr|M4P2F2|M4P2F2_9HIV1 Envelope glycoprotein gp160 OS=Human immunodeficiency virus 1 OX=11676 GN=env PE=3 SV=1
2::M4P2F4    Mass: 98798    Score: 58     Matches: 1(1)  Sequences: 1(1)
>tr|M4P2F4|M4P2F4_9HIV1 Envelope glycoprotein gp160 OS=Human immunodeficiency virus 1 OX=11676 GN=env PE=3 SV=1
2::M4P2H1    Mass: 98694    Score: 58     Matches: 1(1)  Sequences: 1(1)
>tr|M4P2H1|M4P2H1_9HIV1 Envelope glycoprotein gp160 OS=Human immunodeficiency virus 1 OX=11676 GN=env PE=3 SV=1
```

2::M4P2I4 **Mass:** 98717 **Score:** 58 **Matches:** 1(1) **Sequences:** 1(1)
>tr|M4P2I4|M4P2I4_9HIV1 Envelope glycoprotein gp160 OS=Human immunodeficiency virus 1 OX=11676 GN=env PE=3 SV=1
2::M4P2I8 **Mass:** 98668 **Score:** 58 **Matches:** 1(1) **Sequences:** 1(1)
>tr|M4P2I8|M4P2I8_9HIV1 Envelope glycoprotein gp160 OS=Human immunodeficiency virus 1 OX=11676 GN=env PE=3 SV=1
2::M4P2M0 **Mass:** 98682 **Score:** 58 **Matches:** 1(1) **Sequences:** 1(1)
>tr|M4P2M0|M4P2M0_9HIV1 Envelope glycoprotein gp160 OS=Human immunodeficiency virus 1 OX=11676 GN=env PE=3 SV=1
2::M4P2U7 **Mass:** 98696 **Score:** 58 **Matches:** 1(1) **Sequences:** 1(1)
>tr|M4P2U7|M4P2U7_9HIV1 Envelope glycoprotein gp160 OS=Human immunodeficiency virus 1 OX=11676 GN=env PE=3 SV=1
2::M4P2W1 **Mass:** 98739 **Score:** 58 **Matches:** 1(1) **Sequences:** 1(1)
>tr|M4P2W1|M4P2W1_9HIV1 Envelope glycoprotein gp160 OS=Human immunodeficiency virus 1 OX=11676 GN=env PE=3 SV=1
2::M4P2Y3 **Mass:** 98744 **Score:** 58 **Matches:** 1(1) **Sequences:** 1(1)
>tr|M4P2Y3|M4P2Y3_9HIV1 Envelope glycoprotein gp160 OS=Human immunodeficiency virus 1 OX=11676 GN=env PE=3 SV=1
2::M4P2Z9 **Mass:** 98712 **Score:** 58 **Matches:** 1(1) **Sequences:** 1(1)
>tr|M4P2Z9|M4P2Z9_9HIV1 Envelope glycoprotein gp160 OS=Human immunodeficiency virus 1 OX=11676 GN=env PE=3 SV=1
2::M4P332 **Mass:** 98728 **Score:** 58 **Matches:** 1(1) **Sequences:** 1(1)
>tr|M4P332|M4P332_9HIV1 Envelope glycoprotein gp160 OS=Human immunodeficiency virus 1 OX=11676 GN=env PE=3 SV=1
2::M4P3A2 **Mass:** 98664 **Score:** 58 **Matches:** 1(1) **Sequences:** 1(1)
>tr|M4P3A2|M4P3A2_9HIV1 Envelope glycoprotein gp160 OS=Human immunodeficiency virus 1 OX=11676 GN=env PE=3 SV=1
2::M4P3H0 **Mass:** 98159 **Score:** 58 **Matches:** 1(1) **Sequences:** 1(1)
>tr|M4P3H0|M4P3H0_9HIV1 Envelope glycoprotein gp160 OS=Human immunodeficiency virus 1 OX=11676 GN=env PE=3 SV=1
2::M4P3P5 **Mass:** 98528 **Score:** 58 **Matches:** 1(1) **Sequences:** 1(1)
>tr|M4P3P5|M4P3P5_9HIV1 Envelope glycoprotein gp160 OS=Human immunodeficiency virus 1 OX=11676 GN=env PE=3 SV=1
2::M4P3R0 **Mass:** 98505 **Score:** 58 **Matches:** 1(1) **Sequences:** 1(1)
>tr|M4P3R0|M4P3R0_9HIV1 Envelope glycoprotein gp160 OS=Human immunodeficiency virus 1 OX=11676 GN=env PE=3 SV=1
2::M4P3V5 **Mass:** 98096 **Score:** 58 **Matches:** 1(1) **Sequences:** 1(1)
>tr|M4P3V5|M4P3V5_9HIV1 Envelope glycoprotein gp160 OS=Human immunodeficiency virus 1 OX=11676 GN=env PE=3 SV=1
2::M4P5B2 **Mass:** 98668 **Score:** 58 **Matches:** 1(1) **Sequences:** 1(1)
>tr|M4P5B2|M4P5B2_9HIV1 Envelope glycoprotein gp160 OS=Human immunodeficiency virus 1 OX=11676 GN=env PE=3 SV=1
2::M4P5I2 **Mass:** 98748 **Score:** 58 **Matches:** 1(1) **Sequences:** 1(1)
>tr|M4P5I2|M4P5I2_9HIV1 Envelope glycoprotein gp160 OS=Human immunodeficiency virus 1 OX=11676 GN=env PE=3 SV=1
2::M4P5R1 **Mass:** 98682 **Score:** 58 **Matches:** 1(1) **Sequences:** 1(1)
>tr|M4P5R1|M4P5R1_9HIV1 Envelope glycoprotein gp160 OS=Human immunodeficiency virus 1 OX=11676 GN=env PE=3 SV=1
2::M4P5T1 **Mass:** 97144 **Score:** 58 **Matches:** 1(1) **Sequences:** 1(1)
>tr|M4P5T1|M4P5T1_9HIV1 Envelope glycoprotein gp160 OS=Human immunodeficiency virus 1 OX=11676 GN=env PE=3 SV=1
2::M4P645 **Mass:** 97158 **Score:** 58 **Matches:** 1(1) **Sequences:** 1(1)
>tr|M4P645|M4P645_9HIV1 Envelope glycoprotein gp160 OS=Human immunodeficiency virus 1 OX=11676 GN=env PE=3 SV=1
2::M4P656 **Mass:** 96174 **Score:** 58 **Matches:** 1(1) **Sequences:** 1(1)
>tr|M4P656|M4P656_9HIV1 Envelope glycoprotein gp160 OS=Human immunodeficiency virus 1 OX=11676 GN=env PE=3 SV=1
2::M4P684 **Mass:** 97098 **Score:** 58 **Matches:** 1(1) **Sequences:** 1(1)
>tr|M4P684|M4P684_9HIV1 Envelope glycoprotein gp160 OS=Human immunodeficiency virus 1 OX=11676 GN=env PE=3 SV=1
2::M4P692 **Mass:** 97137 **Score:** 58 **Matches:** 1(1) **Sequences:** 1(1)
>tr|M4P692|M4P692_9HIV1 Envelope glycoprotein gp160 OS=Human immunodeficiency virus 1 OX=11676 GN=env PE=3 SV=1
2::M4P6C6 **Mass:** 96961 **Score:** 58 **Matches:** 1(1) **Sequences:** 1(1)
>tr|M4P6C6|M4P6C6_9HIV1 Envelope glycoprotein gp160 OS=Human immunodeficiency virus 1 OX=11676 GN=env PE=3 SV=1
2::M4P6C7 **Mass:** 98668 **Score:** 58 **Matches:** 1(1) **Sequences:** 1(1)
>tr|M4P6C7|M4P6C7_9HIV1 Envelope glycoprotein gp160 OS=Human immunodeficiency virus 1 OX=11676 GN=env PE=3 SV=1
2::M4P6C9 **Mass:** 97179 **Score:** 58 **Matches:** 1(1) **Sequences:** 1(1)
>tr|M4P6C9|M4P6C9_9HIV1 Envelope glycoprotein gp160 OS=Human immunodeficiency virus 1 OX=11676 GN=env PE=3 SV=1
2::M4P6F2 **Mass:** 98557 **Score:** 58 **Matches:** 1(1) **Sequences:** 1(1)
>tr|M4P6F2|M4P6F2_9HIV1 Envelope glycoprotein gp160 OS=Human immunodeficiency virus 1 OX=11676 GN=env PE=3 SV=1
2::M4P6F3 **Mass:** 98705 **Score:** 58 **Matches:** 1(1) **Sequences:** 1(1)
>tr|M4P6F3|M4P6F3_9HIV1 Envelope glycoprotein gp160 OS=Human immunodeficiency virus 1 OX=11676 GN=env PE=3 SV=1
2::M4P6G3 **Mass:** 98695 **Score:** 58 **Matches:** 1(1) **Sequences:** 1(1)
>tr|M4P6G3|M4P6G3_9HIV1 Envelope glycoprotein gp160 OS=Human immunodeficiency virus 1 OX=11676 GN=env PE=3 SV=1
2::M4P6H5 **Mass:** 97187 **Score:** 58 **Matches:** 1(1) **Sequences:** 1(1)
>tr|M4P6H5|M4P6H5_9HIV1 Envelope glycoprotein gp160 OS=Human immunodeficiency virus 1 OX=11676 GN=env PE=3 SV=1
2::M4P6I3 **Mass:** 97204 **Score:** 58 **Matches:** 1(1) **Sequences:** 1(1)
>tr|M4P6I3|M4P6I3_9HIV1 Envelope glycoprotein gp160 OS=Human immunodeficiency virus 1 OX=11676 GN=env PE=3 SV=1
2::M4P6K6 **Mass:** 97103 **Score:** 58 **Matches:** 1(1) **Sequences:** 1(1)
>tr|M4P6K6|M4P6K6_9HIV1 Envelope glycoprotein gp160 OS=Human immunodeficiency virus 1 OX=11676 GN=env PE=3 SV=1
2::M4P6M9 **Mass:** 98766 **Score:** 58 **Matches:** 1(1) **Sequences:** 1(1)
>tr|M4P6M9|M4P6M9_9HIV1 Envelope glycoprotein gp160 OS=Human immunodeficiency virus 1 OX=11676 GN=env PE=3 SV=1
2::M4P6P7 **Mass:** 97242 **Score:** 58 **Matches:** 1(1) **Sequences:** 1(1)
>tr|M4P6P7|M4P6P7_9HIV1 Envelope glycoprotein gp160 OS=Human immunodeficiency virus 1 OX=11676 GN=env PE=3 SV=1
2::M4P6T9 **Mass:** 97772 **Score:** 58 **Matches:** 1(1) **Sequences:** 1(1)
>tr|M4P6T9|M4P6T9_9HIV1 Envelope glycoprotein gp160 OS=Human immunodeficiency virus 1 OX=11676 GN=env PE=3 SV=1
2::M4P6X8 **Mass:** 98937 **Score:** 58 **Matches:** 1(1) **Sequences:** 1(1)
>tr|M4P6X8|M4P6X8_9HIV1 Envelope glycoprotein gp160 OS=Human immunodeficiency virus 1 OX=11676 GN=env PE=3 SV=1
2::M4P708 **Mass:** 98694 **Score:** 58 **Matches:** 1(1) **Sequences:** 1(1)
>tr|M4P708|M4P708_9HIV1 Envelope glycoprotein gp160 OS=Human immunodeficiency virus 1 OX=11676 GN=env PE=3 SV=1
2::M4P750 **Mass:** 98654 **Score:** 58 **Matches:** 1(1) **Sequences:** 1(1)
>tr|M4P750|M4P750_9HIV1 Envelope glycoprotein gp160 OS=Human immunodeficiency virus 1 OX=11676 GN=env PE=3 SV=1
2::M4P781 **Mass:** 94662 **Score:** 58 **Matches:** 1(1) **Sequences:** 1(1)
>tr|M4P781|M4P781_9HIV1 Envelope glycoprotein gp160 OS=Human immunodeficiency virus 1 OX=11676 GN=env PE=3 SV=1
2::M4P7F9 **Mass:** 98375 **Score:** 58 **Matches:** 1(1) **Sequences:** 1(1)
>tr|M4P7F9|M4P7F9_9HIV1 Envelope glycoprotein gp160 OS=Human immunodeficiency virus 1 OX=11676 GN=env PE=3 SV=1
2::M4P7H4 **Mass:** 98390 **Score:** 58 **Matches:** 1(1) **Sequences:** 1(1)
>tr|M4P7H4|M4P7H4_9HIV1 Envelope glycoprotein gp160 OS=Human immunodeficiency virus 1 OX=11676 GN=env PE=3 SV=1
2::M4P7T3 **Mass:** 98387 **Score:** 58 **Matches:** 1(1) **Sequences:** 1(1)
>tr|M4P7T3|M4P7T3_9HIV1 Envelope glycoprotein gp160 OS=Human immunodeficiency virus 1 OX=11676 GN=env PE=3 SV=1
2::M4P8M5 **Mass:** 97046 **Score:** 58 **Matches:** 1(1) **Sequences:** 1(1)
>tr|M4P8M5|M4P8M5_9HIV1 Envelope glycoprotein gp160 OS=Human immunodeficiency virus 1 OX=11676 GN=env PE=3 SV=1
2::M4P8T1 **Mass:** 97076 **Score:** 58 **Matches:** 1(1) **Sequences:** 1(1)
>tr|M4P8T1|M4P8T1_9HIV1 Envelope glycoprotein gp160 OS=Human immunodeficiency virus 1 OX=11676 GN=env PE=3 SV=1
2::M4P8W9 **Mass:** 97216 **Score:** 58 **Matches:** 1(1) **Sequences:** 1(1)
>tr|M4P8W9|M4P8W9_9HIV1 Envelope glycoprotein gp160 OS=Human immunodeficiency virus 1 OX=11676 GN=env PE=3 SV=1
2::M4P951 **Mass:** 97163 **Score:** 58 **Matches:** 1(1) **Sequences:** 1(1)
>tr|M4P951|M4P951_9HIV1 Envelope glycoprotein gp160 OS=Human immunodeficiency virus 1 OX=11676 GN=env PE=3 SV=1
2::M4P9F6 **Mass:** 97254 **Score:** 58 **Matches:** 1(1) **Sequences:** 1(1)
>tr|M4P9F6|M4P9F6_9HIV1 Envelope glycoprotein gp160 OS=Human immunodeficiency virus 1 OX=11676 GN=env PE=3 SV=1
2::M4P9G8 **Mass:** 97627 **Score:** 58 **Matches:** 1(1) **Sequences:** 1(1)
>tr|M4P9G8|M4P9G8_9HIV1 Envelope glycoprotein gp160 OS=Human immunodeficiency virus 1 OX=11676 GN=env PE=3 SV=1
2::M4P9L1 **Mass:** 97662 **Score:** 58 **Matches:** 1(1) **Sequences:** 1(1)
>tr|M4P9L1|M4P9L1_9HIV1 Envelope glycoprotein gp160 OS=Human immunodeficiency virus 1 OX=11676 GN=env PE=3 SV=1
2::M4P9M6 **Mass:** 97909 **Score:** 58 **Matches:** 1(1) **Sequences:** 1(1)
>tr|M4P9M6|M4P9M6_9HIV1 Envelope glycoprotein gp160 OS=Human immunodeficiency virus 1 OX=11676 GN=env PE=3 SV=1
2::M4P9V6 **Mass:** 97105 **Score:** 58 **Matches:** 1(1) **Sequences:** 1(1)
>tr|M4P9V6|M4P9V6_9HIV1 Envelope glycoprotein gp160 OS=Human immunodeficiency virus 1 OX=11676 GN=env PE=3 SV=1
2::M4PA43 **Mass:** 97164 **Score:** 58 **Matches:** 1(1) **Sequences:** 1(1)
>tr|M4PA43|M4PA43_9HIV1 Envelope glycoprotein gp160 OS=Human immunodeficiency virus 1 OX=11676 GN=env PE=3 SV=1
2::M4PAB3 **Mass:** 97244 **Score:** 58 **Matches:** 1(1) **Sequences:** 1(1)
>tr|M4PAB3|M4PAB3_9HIV1 Envelope glycoprotein gp160 OS=Human immunodeficiency virus 1 OX=11676 GN=env PE=3 SV=1
2::M4PAE8 **Mass:** 96711 **Score:** 58 **Matches:** 1(1) **Sequences:** 1(1)
>tr|M4PAE8|M4PAE8_9HIV1 Envelope glycoprotein gp160 OS=Human immunodeficiency virus 1 OX=11676 GN=env PE=3 SV=1
2::M4PAM2 **Mass:** 97177 **Score:** 58 **Matches:** 1(1) **Sequences:** 1(1)
>tr|M4PAM2|M4PAM2_9HIV1 Envelope glycoprotein gp160 OS=Human immunodeficiency virus 1 OX=11676 GN=env PE=3 SV=1
2::M4PCU2 **Mass:** 98767 **Score:** 58 **Matches:** 1(1) **Sequences:** 1(1)
>tr|M4PCU2|M4PCU2_9HIV1 Envelope glycoprotein gp160 OS=Human immunodeficiency virus 1 OX=11676 GN=env PE=3 SV=1
2::M4PD48 **Mass:** 98758 **Score:** 58 **Matches:** 1(1) **Sequences:** 1(1)
>tr|M4PD48|M4PD48_9HIV1 Envelope glycoprotein gp160 OS=Human immunodeficiency virus 1 OX=11676 GN=env PE=3 SV=1

2::M4PD62 **Mass:** 98656 **Score:** 58 **Matches:** 1(1) **Sequences:** 1(1)
>tr|M4PD62|M4PD62_9HIV1 Envelope glycoprotein gp160 OS=Human immunodeficiency virus 1 OX=11676 GN=env PE=3 SV=1
2::M4PD92 **Mass:** 98707 **Score:** 58 **Matches:** 1(1) **Sequences:** 1(1)
>tr|M4PD92|M4PD92_9HIV1 Envelope glycoprotein gp160 OS=Human immunodeficiency virus 1 OX=11676 GN=env PE=3 SV=1
2::M4PDB8 **Mass:** 98596 **Score:** 58 **Matches:** 1(1) **Sequences:** 1(1)
>tr|M4PDB8|M4PDB8_9HIV1 Envelope glycoprotein gp160 OS=Human immunodeficiency virus 1 OX=11676 GN=env PE=3 SV=1
2::M4PDH3 **Mass:** 98640 **Score:** 58 **Matches:** 1(1) **Sequences:** 1(1)
>tr|M4PDH3|M4PDH3_9HIV1 Envelope glycoprotein gp160 OS=Human immunodeficiency virus 1 OX=11676 GN=env PE=3 SV=1
2::M4PDV2 **Mass:** 98670 **Score:** 58 **Matches:** 1(1) **Sequences:** 1(1)
>tr|M4PDV2|M4PDV2_9HIV1 Envelope glycoprotein gp160 OS=Human immunodeficiency virus 1 OX=11676 GN=env PE=3 SV=1
2::M4PDW5 **Mass:** 98749 **Score:** 58 **Matches:** 1(1) **Sequences:** 1(1)
>tr|M4PDW5|M4PDW5_9HIV1 Envelope glycoprotein gp160 OS=Human immunodeficiency virus 1 OX=11676 GN=env PE=3 SV=1
2::M4PDX9 **Mass:** 98496 **Score:** 58 **Matches:** 1(1) **Sequences:** 1(1)
>tr|M4PDX9|M4PDX9_9HIV1 Envelope glycoprotein gp160 OS=Human immunodeficiency virus 1 OX=11676 GN=env PE=3 SV=1
2::M4PE38 **Mass:** 98576 **Score:** 58 **Matches:** 1(1) **Sequences:** 1(1)
>tr|M4PE38|M4PE38_9HIV1 Envelope glycoprotein gp160 OS=Human immunodeficiency virus 1 OX=11676 GN=env PE=3 SV=1
2::M4PE80 **Mass:** 98432 **Score:** 58 **Matches:** 1(1) **Sequences:** 1(1)
>tr|M4PE80|M4PE80_9HIV1 Envelope glycoprotein gp160 OS=Human immunodeficiency virus 1 OX=11676 GN=env PE=3 SV=1
2::M4PEF5 **Mass:** 98320 **Score:** 58 **Matches:** 1(1) **Sequences:** 1(1)
>tr|M4PEF5|M4PEF5_9HIV1 Envelope glycoprotein gp160 OS=Human immunodeficiency virus 1 OX=11676 GN=env PE=3 SV=1
2::M4PEZ3 **Mass:** 98667 **Score:** 58 **Matches:** 1(1) **Sequences:** 1(1)
>tr|M4PEZ3|M4PEZ3_9HIV1 Envelope glycoprotein gp160 OS=Human immunodeficiency virus 1 OX=11676 GN=env PE=3 SV=1
2::M4PG60 **Mass:** 97119 **Score:** 58 **Matches:** 1(1) **Sequences:** 1(1)
>tr|M4PG60|M4PG60_9HIV1 Envelope glycoprotein gp160 OS=Human immunodeficiency virus 1 OX=11676 GN=env PE=3 SV=1
2::M4PGH7 **Mass:** 97112 **Score:** 58 **Matches:** 1(1) **Sequences:** 1(1)
>tr|M4PGH7|M4PGH7_9HIV1 Envelope glycoprotein gp160 OS=Human immunodeficiency virus 1 OX=11676 GN=env PE=3 SV=1
2::M4PGL4 **Mass:** 97202 **Score:** 58 **Matches:** 1(1) **Sequences:** 1(1)
>tr|M4PGL4|M4PGL4_9HIV1 Envelope glycoprotein gp160 OS=Human immunodeficiency virus 1 OX=11676 GN=env PE=3 SV=1
2::M4PGQ2 **Mass:** 97204 **Score:** 58 **Matches:** 1(1) **Sequences:** 1(1)
>tr|M4PGQ2|M4PGQ2_9HIV1 Envelope glycoprotein gp160 OS=Human immunodeficiency virus 1 OX=11676 GN=env PE=3 SV=1
2::M4PGV0 **Mass:** 81666 **Score:** 58 **Matches:** 1(1) **Sequences:** 1(1)
>tr|M4PGV0|M4PGV0_9HIV1 Envelope glycoprotein gp160 OS=Human immunodeficiency virus 1 OX=11676 GN=env PE=4 SV=1
2::M4PGZ8 **Mass:** 97148 **Score:** 58 **Matches:** 1(1) **Sequences:** 1(1)
>tr|M4PGZ8|M4PGZ8_9HIV1 Envelope glycoprotein gp160 OS=Human immunodeficiency virus 1 OX=11676 GN=env PE=3 SV=1
2::Q4JG30 **Mass:** 99653 **Score:** 58 **Matches:** 1(1) **Sequences:** 1(1)
>tr|Q4JG30|Q4JG30_9HIV1 Envelope glycoprotein gp160 OS=Human immunodeficiency virus 1 OX=11676 GN=env PE=3 SV=1
2::Q4JGJ0 **Mass:** 97120 **Score:** 58 **Matches:** 1(1) **Sequences:** 1(1)
>tr|Q4JGJ0|Q4JGJ0_9HIV1 Envelope glycoprotein gp160 OS=Human immunodeficiency virus 1 OX=11676 GN=env PE=3 SV=1
2::Q4PT53 **Mass:** 97311 **Score:** 58 **Matches:** 1(1) **Sequences:** 1(1)
>tr|Q4PT53|Q4PT53_9HIV1 Envelope glycoprotein gp160 OS=Human immunodeficiency virus 1 OX=11676 GN=env PE=3 SV=1
2::R4UYM6 **Mass:** 100453 **Score:** 58 **Matches:** 1(1) **Sequences:** 1(1)
>tr|R4UYM6|R4UYM6_9HIV1 Envelope glycoprotein gp160 OS=Human immunodeficiency virus 1 OX=11676 GN=env PE=3 SV=1
2::R4V966 **Mass:** 97427 **Score:** 58 **Matches:** 1(1) **Sequences:** 1(1)
>tr|R4V966|R4V966_9HIV1 Envelope glycoprotein gp160 OS=Human immunodeficiency virus 1 OX=11676 GN=env PE=3 SV=1
2::R4VGS0 **Mass:** 99548 **Score:** 58 **Matches:** 1(1) **Sequences:** 1(1)
>tr|R4VGS0|R4VGS0_9HIV1 Envelope glycoprotein gp160 OS=Human immunodeficiency virus 1 OX=11676 GN=env PE=3 SV=1
2::R4VGW5 **Mass:** 97311 **Score:** 58 **Matches:** 1(1) **Sequences:** 1(1)
>tr|R4VGW5|R4VGW5_9HIV1 Envelope glycoprotein gp160 OS=Human immunodeficiency virus 1 OX=11676 GN=env PE=3 SV=1
2::B5ANK0 **Mass:** 96535 **Score:** 58 **Matches:** 1(1) **Sequences:** 1(1)
>tr|B5ANK0|B5ANK0_9HIV1 Envelope glycoprotein gp160 (Fragment) OS=Human immunodeficiency virus 1 OX=11676 GN=env PE=3 SV=1
2::B5ANK3 **Mass:** 96835 **Score:** 58 **Matches:** 1(1) **Sequences:** 1(1)
>tr|B5ANK3|B5ANK3_9HIV1 Envelope glycoprotein gp160 (Fragment) OS=Human immunodeficiency virus 1 OX=11676 GN=env PE=3 SV=1
2::B5ANK5 **Mass:** 96356 **Score:** 58 **Matches:** 1(1) **Sequences:** 1(1)
>tr|B5ANK5|B5ANK5_9HIV1 Envelope glycoprotein gp160 (Fragment) OS=Human immunodeficiency virus 1 OX=11676 GN=env PE=3 SV=1
2::C5HSY0 **Mass:** 96641 **Score:** 58 **Matches:** 1(1) **Sequences:** 1(1)
>tr|C5HSY0|C5HSY0_9HIV1 Envelope glycoprotein gp160 OS=Human immunodeficiency virus 1 OX=11676 GN=env PE=3 SV=1
2::C5HT06 **Mass:** 96701 **Score:** 58 **Matches:** 1(1) **Sequences:** 1(1)
>tr|C5HT06|C5HT06_9HIV1 Envelope glycoprotein gp160 OS=Human immunodeficiency virus 1 OX=11676 GN=env PE=3 SV=1
2::C5HT15 **Mass:** 96671 **Score:** 58 **Matches:** 1(1) **Sequences:** 1(1)
>tr|C5HT15|C5HT15_9HIV1 Envelope glycoprotein gp160 OS=Human immunodeficiency virus 1 OX=11676 GN=env PE=3 SV=1
2::D5LLR3 **Mass:** 99595 **Score:** 58 **Matches:** 1(1) **Sequences:** 1(1)
>tr|D5LLR3|D5LLR3_9HIV1 Envelope glycoprotein gp160 OS=Human immunodeficiency virus 1 OX=11676 GN=env PE=3 SV=1
2::D5LLS3 **Mass:** 100058 **Score:** 58 **Matches:** 1(1) **Sequences:** 1(1)
>tr|D5LLS3|D5LLS3_9HIV1 Envelope glycoprotein gp160 OS=Human immunodeficiency virus 1 OX=11676 GN=env PE=3 SV=1
2::D5LLT0 **Mass:** 100116 **Score:** 58 **Matches:** 1(1) **Sequences:** 1(1)
>tr|D5LLT0|D5LLT0_9HIV1 Envelope glycoprotein gp160 OS=Human immunodeficiency virus 1 OX=11676 GN=env PE=3 SV=1
2::D5LLT1 **Mass:** 99918 **Score:** 58 **Matches:** 1(1) **Sequences:** 1(1)
>tr|D5LLT1|D5LLT1_9HIV1 Envelope glycoprotein gp160 OS=Human immunodeficiency virus 1 OX=11676 GN=env PE=3 SV=1
2::D5LLT2 **Mass:** 99836 **Score:** 58 **Matches:** 1(1) **Sequences:** 1(1)
>tr|D5LLT2|D5LLT2_9HIV1 Envelope glycoprotein gp160 OS=Human immunodeficiency virus 1 OX=11676 GN=env PE=3 SV=1
2::D5LLU2 **Mass:** 100337 **Score:** 58 **Matches:** 1(1) **Sequences:** 1(1)
>tr|D5LLU2|D5LLU2_9HIV1 Envelope glycoprotein gp160 OS=Human immunodeficiency virus 1 OX=11676 GN=env PE=3 SV=1
2::D5LLU3 **Mass:** 98927 **Score:** 58 **Matches:** 1(1) **Sequences:** 1(1)
>tr|D5LLU3|D5LLU3_9HIV1 Envelope glycoprotein gp160 OS=Human immunodeficiency virus 1 OX=11676 GN=env PE=3 SV=1
2::D5LLU5 **Mass:** 100228 **Score:** 58 **Matches:** 1(1) **Sequences:** 1(1)
>tr|D5LLU5|D5LLU5_9HIV1 Envelope glycoprotein gp160 OS=Human immunodeficiency virus 1 OX=11676 GN=env PE=3 SV=1
2::D5LLU6 **Mass:** 100115 **Score:** 58 **Matches:** 1(1) **Sequences:** 1(1)
>tr|D5LLU6|D5LLU6_9HIV1 Envelope glycoprotein gp160 OS=Human immunodeficiency virus 1 OX=11676 GN=env PE=3 SV=1
2::D5LLV1 **Mass:** 98654 **Score:** 58 **Matches:** 1(1) **Sequences:** 1(1)
>tr|D5LLV1|D5LLV1_9HIV1 Envelope glycoprotein gp160 OS=Human immunodeficiency virus 1 OX=11676 GN=env PE=3 SV=1
2::D5LLV2 **Mass:** 95228 **Score:** 58 **Matches:** 1(1) **Sequences:** 1(1)
>tr|D5LLV2|D5LLV2_9HIV1 Envelope glycoprotein gp160 OS=Human immunodeficiency virus 1 OX=11676 GN=env PE=3 SV=1
2::D5LLV4 **Mass:** 98342 **Score:** 58 **Matches:** 1(1) **Sequences:** 1(1)
>tr|D5LLV4|D5LLV4_9HIV1 Envelope glycoprotein gp160 OS=Human immunodeficiency virus 1 OX=11676 GN=env PE=3 SV=1
2::D5LLV5 **Mass:** 99363 **Score:** 58 **Matches:** 1(1) **Sequences:** 1(1)
>tr|D5LLV5|D5LLV5_9HIV1 Envelope glycoprotein gp160 OS=Human immunodeficiency virus 1 OX=11676 GN=env PE=3 SV=1
2::D5LLV7 **Mass:** 98702 **Score:** 58 **Matches:** 1(1) **Sequences:** 1(1)
>tr|D5LLV7|D5LLV7_9HIV1 Envelope glycoprotein gp160 OS=Human immunodeficiency virus 1 OX=11676 GN=env PE=3 SV=1
2::D5LLV8 **Mass:** 98415 **Score:** 58 **Matches:** 1(1) **Sequences:** 1(1)
>tr|D5LLV8|D5LLV8_9HIV1 Envelope glycoprotein gp160 OS=Human immunodeficiency virus 1 OX=11676 GN=env PE=3 SV=1
2::D5LLW0 **Mass:** 97568 **Score:** 58 **Matches:** 1(1) **Sequences:** 1(1)
>tr|D5LLW0|D5LLW0_9HIV1 Envelope glycoprotein gp160 OS=Human immunodeficiency virus 1 OX=11676 GN=env PE=3 SV=1
2::D5LLW5 **Mass:** 98748 **Score:** 58 **Matches:** 1(1) **Sequences:** 1(1)
>tr|D5LLW5|D5LLW5_9HIV1 Envelope glycoprotein gp160 OS=Human immunodeficiency virus 1 OX=11676 GN=env PE=3 SV=1
2::E5RER5 **Mass:** 100654 **Score:** 58 **Matches:** 1(1) **Sequences:** 1(1)
>tr|E5RER5|E5RER5_9HIV1 Envelope glycoprotein gp160 OS=Human immunodeficiency virus 1 OX=11676 GN=env PE=3 SV=1
2::E5REV4 **Mass:** 99530 **Score:** 58 **Matches:** 1(1) **Sequences:** 1(1)
>tr|E5REV4|E5REV4_9HIV1 Envelope glycoprotein gp160 OS=Human immunodeficiency virus 1 OX=11676 GN=env PE=3 SV=1
2::E5REV8 **Mass:** 99516 **Score:** 58 **Matches:** 1(1) **Sequences:** 1(1)
>tr|E5REV8|E5REV8_9HIV1 Envelope glycoprotein gp160 OS=Human immunodeficiency virus 1 OX=11676 GN=env PE=3 SV=1
2::F5AYA0 **Mass:** 98134 **Score:** 58 **Matches:** 1(1) **Sequences:** 1(1)
>tr|F5AYA0|F5AYA0_9HIV1 Envelope glycoprotein gp160 OS=Human immunodeficiency virus 1 OX=11676 GN=env PE=3 SV=1
2::F5AYA4 **Mass:** 98277 **Score:** 58 **Matches:** 1(1) **Sequences:** 1(1)
>tr|F5AYA4|F5AYA4_9HIV1 Envelope glycoprotein gp160 OS=Human immunodeficiency virus 1 OX=11676 GN=env PE=3 SV=1
2::F5AYA8 **Mass:** 98459 **Score:** 58 **Matches:** 1(1) **Sequences:** 1(1)
>tr|F5AYA8|F5AYA8_9HIV1 Envelope glycoprotein gp160 OS=Human immunodeficiency virus 1 OX=11676 GN=env PE=3 SV=1
2::F5AYB2 **Mass:** 98032 **Score:** 58 **Matches:** 1(1) **Sequences:** 1(1)
>tr|F5AYB2|F5AYB2_9HIV1 Envelope glycoprotein gp160 OS=Human immunodeficiency virus 1 OX=11676 GN=env PE=3 SV=1
2::F5AYB6 **Mass:** 99028 **Score:** 58 **Matches:** 1(1) **Sequences:** 1(1)
>tr|F5AYB6|F5AYB6_9HIV1 Envelope glycoprotein gp160 OS=Human immunodeficiency virus 1 OX=11676 GN=env PE=3 SV=1

2::F5AYC0 **Mass:** 98570 **Score:** 58 **Matches:** 1(1) **Sequences:** 1(1)
>tr|F5AYC0|F5AYC0_9HIV1 Envelope glycoprotein gp160 OS=Human immunodeficiency virus 1 OX=11676 GN=env PE=3 SV=1
2::F5AYC4 **Mass:** 98709 **Score:** 58 **Matches:** 1(1) **Sequences:** 1(1)
>tr|F5AYC4|F5AYC4_9HIV1 Envelope glycoprotein gp160 OS=Human immunodeficiency virus 1 OX=11676 GN=env PE=3 SV=1
2::F5AYD2 **Mass:** 98088 **Score:** 58 **Matches:** 1(1) **Sequences:** 1(1)
>tr|F5AYD2|F5AYD2_9HIV1 Envelope glycoprotein gp160 OS=Human immunodeficiency virus 1 OX=11676 GN=env PE=3 SV=1
2::F5AYD6 **Mass:** 98440 **Score:** 58 **Matches:** 1(1) **Sequences:** 1(1)
>tr|F5AYD6|F5AYD6_9HIV1 Envelope glycoprotein gp160 OS=Human immunodeficiency virus 1 OX=11676 GN=env PE=3 SV=1
2::F5AYE0 **Mass:** 98077 **Score:** 58 **Matches:** 1(1) **Sequences:** 1(1)
>tr|F5AYE0|F5AYE0_9HIV1 Envelope glycoprotein gp160 OS=Human immunodeficiency virus 1 OX=11676 GN=env PE=3 SV=1
2::F5AYE4 **Mass:** 98091 **Score:** 58 **Matches:** 1(1) **Sequences:** 1(1)
>tr|F5AYE4|F5AYE4_9HIV1 Envelope glycoprotein gp160 OS=Human immunodeficiency virus 1 OX=11676 GN=env PE=3 SV=1
2::F5AYE8 **Mass:** 98239 **Score:** 58 **Matches:** 1(1) **Sequences:** 1(1)
>tr|F5AYE8|F5AYE8_9HIV1 Envelope glycoprotein gp160 OS=Human immunodeficiency virus 1 OX=11676 GN=env PE=3 SV=1
2::F5AYF2 **Mass:** 98349 **Score:** 58 **Matches:** 1(1) **Sequences:** 1(1)
>tr|F5AYF2|F5AYF2_9HIV1 Envelope glycoprotein gp160 OS=Human immunodeficiency virus 1 OX=11676 GN=env PE=3 SV=1
2::F5AYF6 **Mass:** 98457 **Score:** 58 **Matches:** 1(1) **Sequences:** 1(1)
>tr|F5AYF6|F5AYF6_9HIV1 Envelope glycoprotein gp160 OS=Human immunodeficiency virus 1 OX=11676 GN=env PE=3 SV=1
2::F5AYG0 **Mass:** 97912 **Score:** 58 **Matches:** 1(1) **Sequences:** 1(1)
>tr|F5AYG0|F5AYG0_9HIV1 Envelope glycoprotein gp160 OS=Human immunodeficiency virus 1 OX=11676 GN=env PE=3 SV=1
2::F5AYG4 **Mass:** 98141 **Score:** 58 **Matches:** 1(1) **Sequences:** 1(1)
>tr|F5AYG4|F5AYG4_9HIV1 Envelope glycoprotein gp160 OS=Human immunodeficiency virus 1 OX=11676 GN=env PE=3 SV=1
2::F5AYH5 **Mass:** 98504 **Score:** 58 **Matches:** 1(1) **Sequences:** 1(1)
>tr|F5AYH5|F5AYH5_9HIV1 Envelope glycoprotein gp160 OS=Human immunodeficiency virus 1 OX=11676 GN=env PE=3 SV=1
2::F5AYH9 **Mass:** 98340 **Score:** 58 **Matches:** 1(1) **Sequences:** 1(1)
>tr|F5AYH9|F5AYH9_9HIV1 Envelope glycoprotein gp160 OS=Human immunodeficiency virus 1 OX=11676 GN=env PE=3 SV=1
2::F5AYI3 **Mass:** 97804 **Score:** 58 **Matches:** 1(1) **Sequences:** 1(1)
>tr|F5AYI3|F5AYI3_9HIV1 Envelope glycoprotein gp160 OS=Human immunodeficiency virus 1 OX=11676 GN=env PE=3 SV=1
2::F5AYJ1 **Mass:** 98558 **Score:** 58 **Matches:** 1(1) **Sequences:** 1(1)
>tr|F5AYJ1|F5AYJ1_9HIV1 Envelope glycoprotein gp160 OS=Human immunodeficiency virus 1 OX=11676 GN=env PE=3 SV=1
2::F5AYJ5 **Mass:** 98430 **Score:** 58 **Matches:** 1(1) **Sequences:** 1(1)
>tr|F5AYJ5|F5AYJ5_9HIV1 Envelope glycoprotein gp160 OS=Human immunodeficiency virus 1 OX=11676 GN=env PE=3 SV=1
2::F5AYJ9 **Mass:** 98473 **Score:** 58 **Matches:** 1(1) **Sequences:** 1(1)
>tr|F5AYJ9|F5AYJ9_9HIV1 Envelope glycoprotein gp160 OS=Human immunodeficiency virus 1 OX=11676 GN=env PE=3 SV=1
2::F5AYL4 **Mass:** 98232 **Score:** 58 **Matches:** 1(1) **Sequences:** 1(1)
>tr|F5AYL4|F5AYL4_9HIV1 Envelope glycoprotein gp160 OS=Human immunodeficiency virus 1 OX=11676 GN=env PE=3 SV=1
2::F5AYL8 **Mass:** 98474 **Score:** 58 **Matches:** 1(1) **Sequences:** 1(1)
>tr|F5AYL8|F5AYL8_9HIV1 Envelope glycoprotein gp160 OS=Human immunodeficiency virus 1 OX=11676 GN=env PE=3 SV=1
2::F5AYM2 **Mass:** 98460 **Score:** 58 **Matches:** 1(1) **Sequences:** 1(1)
>tr|F5AYM2|F5AYM2_9HIV1 Envelope glycoprotein gp160 OS=Human immunodeficiency virus 1 OX=11676 GN=env PE=3 SV=1
2::F5AYM6 **Mass:** 98435 **Score:** 58 **Matches:** 1(1) **Sequences:** 1(1)
>tr|F5AYM6|F5AYM6_9HIV1 Envelope glycoprotein gp160 OS=Human immunodeficiency virus 1 OX=11676 GN=env PE=3 SV=1
2::F5AYN0 **Mass:** 98521 **Score:** 58 **Matches:** 1(1) **Sequences:** 1(1)
>tr|F5AYN0|F5AYN0_9HIV1 Envelope glycoprotein gp160 OS=Human immunodeficiency virus 1 OX=11676 GN=env PE=3 SV=1
2::F5AYN7 **Mass:** 98719 **Score:** 58 **Matches:** 1(1) **Sequences:** 1(1)
>tr|F5AYN7|F5AYN7_9HIV1 Envelope glycoprotein gp160 OS=Human immunodeficiency virus 1 OX=11676 GN=env PE=3 SV=1
2::F5AYP5 **Mass:** 98450 **Score:** 58 **Matches:** 1(1) **Sequences:** 1(1)
>tr|F5AYP5|F5AYP5_9HIV1 Envelope glycoprotein gp160 OS=Human immunodeficiency virus 1 OX=11676 GN=env PE=3 SV=1
2::F5AYQ3 **Mass:** 98476 **Score:** 58 **Matches:** 1(1) **Sequences:** 1(1)
>tr|F5AYQ3|F5AYQ3_9HIV1 Envelope glycoprotein gp160 OS=Human immunodeficiency virus 1 OX=11676 GN=env PE=3 SV=1
2::F5AYQ7 **Mass:** 98513 **Score:** 58 **Matches:** 1(1) **Sequences:** 1(1)
>tr|F5AYQ7|F5AYQ7_9HIV1 Envelope glycoprotein gp160 OS=Human immunodeficiency virus 1 OX=11676 GN=env PE=3 SV=1
2::F5AYR5 **Mass:** 98444 **Score:** 58 **Matches:** 1(1) **Sequences:** 1(1)
>tr|F5AYR5|F5AYR5_9HIV1 Envelope glycoprotein gp160 OS=Human immunodeficiency virus 1 OX=11676 GN=env PE=3 SV=1
2::F5AYR9 **Mass:** 98540 **Score:** 58 **Matches:** 1(1) **Sequences:** 1(1)
>tr|F5AYR9|F5AYR9_9HIV1 Envelope glycoprotein gp160 OS=Human immunodeficiency virus 1 OX=11676 GN=env PE=3 SV=1
2::G5DRJ5 **Mass:** 97681 **Score:** 58 **Matches:** 1(1) **Sequences:** 1(1)
>tr|G5DRJ5|G5DRJ5_9HIV1 Envelope glycoprotein gp160 OS=Human immunodeficiency virus 1 OX=11676 GN=env PE=3 SV=1
2::G5DRJ9 **Mass:** 97828 **Score:** 58 **Matches:** 1(1) **Sequences:** 1(1)
>tr|G5DRJ9|G5DRJ9_9HIV1 Envelope glycoprotein gp160 OS=Human immunodeficiency virus 1 OX=11676 GN=env PE=3 SV=1
2::G5DRR9 **Mass:** 97972 **Score:** 58 **Matches:** 1(1) **Sequences:** 1(1)
>tr|G5DRR9|G5DRR9_9HIV1 Envelope glycoprotein gp160 OS=Human immunodeficiency virus 1 OX=11676 GN=env PE=3 SV=1
2::Q5EEG4 **Mass:** 97227 **Score:** 58 **Matches:** 1(1) **Sequences:** 1(1)
>tr|Q5EEG4|Q5EEG4_9HIV1 Envelope glycoprotein gp160 OS=Human immunodeficiency virus 1 OX=11676 GN=env PE=3 SV=1
2::Q5FXR3 **Mass:** 98644 **Score:** 58 **Matches:** 1(1) **Sequences:** 1(1)
>tr|Q5FXR3|Q5FXR3_9HIV1 Envelope glycoprotein gp160 OS=Human immunodeficiency virus 1 OX=11676 GN=env PE=3 SV=1
2::Q5FXW6 **Mass:** 100358 **Score:** 58 **Matches:** 1(1) **Sequences:** 1(1)
>tr|Q5FXW6|Q5FXW6_9HIV1 Envelope glycoprotein gp160 OS=Human immunodeficiency virus 1 OX=11676 GN=env PE=3 SV=1
2::Q5FXY4 **Mass:** 97020 **Score:** 58 **Matches:** 1(1) **Sequences:** 1(1)
>tr|Q5FXY4|Q5FXY4_9HIV1 Envelope glycoprotein gp160 OS=Human immunodeficiency virus 1 OX=11676 GN=env PE=3 SV=1
2::Q5GI23 **Mass:** 95908 **Score:** 58 **Matches:** 1(1) **Sequences:** 1(1)
>tr|Q5GI23|Q5GI23_9HIV1 Envelope glycoprotein gp160 OS=Human immunodeficiency virus 1 OX=11676 GN=env PE=3 SV=1
2::Q5PZV4 **Mass:** 96161 **Score:** 58 **Matches:** 1(1) **Sequences:** 1(1)
>tr|Q5PZV4|Q5PZV4_9HIV1 Envelope glycoprotein gp160 OS=Human immunodeficiency virus 1 OX=11676 GN=env PE=3 SV=1
2::Q5U953 **Mass:** 96546 **Score:** 58 **Matches:** 1(1) **Sequences:** 1(1)
>tr|Q5U953|Q5U953_9HIV1 Envelope glycoprotein gp160 OS=Human immunodeficiency virus 1 OX=11676 GN=env PE=3 SV=1
2::Q5U993 **Mass:** 97390 **Score:** 58 **Matches:** 1(1) **Sequences:** 1(1)
>tr|Q5U993|Q5U993_9HIV1 Envelope glycoprotein gp160 OS=Human immunodeficiency virus 1 OX=11676 GN=env PE=3 SV=1
2::B6V2C3 **Mass:** 40365 **Score:** 58 **Matches:** 1(1) **Sequences:** 1(1)
>tr|B6V2C3|B6V2C3_9HIV1 Envelope glycoprotein (Fragment) OS=Human immunodeficiency virus 1 OX=11676 GN=env PE=4 SV=1
2::B6V2E4 **Mass:** 40104 **Score:** 58 **Matches:** 1(1) **Sequences:** 1(1)
>tr|B6V2E4|B6V2E4_9HIV1 Envelope glycoprotein (Fragment) OS=Human immunodeficiency virus 1 OX=11676 GN=env PE=4 SV=1
2::B6V2E7 **Mass:** 40362 **Score:** 58 **Matches:** 1(1) **Sequences:** 1(1)
>tr|B6V2E7|B6V2E7_9HIV1 Envelope glycoprotein (Fragment) OS=Human immunodeficiency virus 1 OX=11676 GN=env PE=4 SV=1
2::B6VH71 **Mass:** 99774 **Score:** 58 **Matches:** 1(1) **Sequences:** 1(1)
>tr|B6VH71|B6VH71_9HIV1 Envelope glycoprotein gp160 OS=Human immunodeficiency virus 1 OX=11676 GN=env PE=3 SV=1
2::B6VH72 **Mass:** 99715 **Score:** 58 **Matches:** 1(1) **Sequences:** 1(1)
>tr|B6VH72|B6VH72_9HIV1 Envelope glycoprotein gp160 OS=Human immunodeficiency virus 1 OX=11676 GN=env PE=3 SV=1
2::B6VH73 **Mass:** 99833 **Score:** 58 **Matches:** 1(1) **Sequences:** 1(1)
>tr|B6VH73|B6VH73_9HIV1 Envelope glycoprotein gp160 OS=Human immunodeficiency virus 1 OX=11676 GN=env PE=3 SV=1
2::B6VH74 **Mass:** 99850 **Score:** 58 **Matches:** 1(1) **Sequences:** 1(1)
>tr|B6VH74|B6VH74_9HIV1 Envelope glycoprotein gp160 OS=Human immunodeficiency virus 1 OX=11676 GN=env PE=3 SV=1
2::C6FXU0 **Mass:** 99042 **Score:** 58 **Matches:** 1(1) **Sequences:** 1(1)
>tr|C6FXU0|C6FXU0_9HIV1 Envelope glycoprotein gp160 OS=Human immunodeficiency virus 1 OX=11676 GN=env PE=3 SV=1
2::C6FXU1 **Mass:** 99227 **Score:** 58 **Matches:** 1(1) **Sequences:** 1(1)
>tr|C6FXU1|C6FXU1_9HIV1 Envelope glycoprotein gp160 OS=Human immunodeficiency virus 1 OX=11676 GN=env PE=3 SV=1
2::C6FXU6 **Mass:** 98564 **Score:** 58 **Matches:** 1(1) **Sequences:** 1(1)
>tr|C6FXU6|C6FXU6_9HIV1 Envelope glycoprotein gp160 OS=Human immunodeficiency virus 1 OX=11676 GN=env PE=3 SV=1
2::C6FXU8 **Mass:** 99209 **Score:** 58 **Matches:** 1(1) **Sequences:** 1(1)
>tr|C6FXU8|C6FXU8_9HIV1 Envelope glycoprotein gp160 OS=Human immunodeficiency virus 1 OX=11676 GN=env PE=3 SV=1
2::C6FY07 **Mass:** 98092 **Score:** 58 **Matches:** 1(1) **Sequences:** 1(1)
>tr|C6FY07|C6FY07_9HIV1 Envelope glycoprotein gp160 OS=Human immunodeficiency virus 1 OX=11676 GN=env PE=3 SV=1
2::C6FY14 **Mass:** 98080 **Score:** 58 **Matches:** 1(1) **Sequences:** 1(1)
>tr|C6FY14|C6FY14_9HIV1 Envelope glycoprotein gp160 OS=Human immunodeficiency virus 1 OX=11676 GN=env PE=3 SV=1
2::C6FY16 **Mass:** 98106 **Score:** 58 **Matches:** 1(1) **Sequences:** 1(1)
>tr|C6FY16|C6FY16_9HIV1 Envelope glycoprotein gp160 OS=Human immunodeficiency virus 1 OX=11676 GN=env PE=3 SV=1
2::C6FY17 **Mass:** 98122 **Score:** 58 **Matches:** 1(1) **Sequences:** 1(1)
>tr|C6FY17|C6FY17_9HIV1 Envelope glycoprotein gp160 OS=Human immunodeficiency virus 1 OX=11676 GN=env PE=3 SV=1
2::C6FY21 **Mass:** 98122 **Score:** 58 **Matches:** 1(1) **Sequences:** 1(1)
>tr|C6FY21|C6FY21_9HIV1 Envelope glycoprotein gp160 OS=Human immunodeficiency virus 1 OX=11676 GN=env PE=3 SV=1

2::C6FY24 **Mass:** 98065 **Score:** 58 **Matches:** 1(1) **Sequences:** 1(1)
>tr|C6FY24|C6FY24_9HIV1 Envelope glycoprotein gp160 OS=Human immunodeficiency virus 1 OX=11676 GN=env PE=3 SV=1
2::C6FY25 **Mass:** 98093 **Score:** 58 **Matches:** 1(1) **Sequences:** 1(1)
>tr|C6FY25|C6FY25_9HIV1 Envelope glycoprotein gp160 OS=Human immunodeficiency virus 1 OX=11676 GN=env PE=3 SV=1
2::C6FY59 **Mass:** 99296 **Score:** 58 **Matches:** 1(1) **Sequences:** 1(1)
>tr|C6FY59|C6FY59_9HIV1 Envelope glycoprotein gp160 OS=Human immunodeficiency virus 1 OX=11676 GN=env PE=3 SV=1
2::C6FYA3 **Mass:** 97092 **Score:** 58 **Matches:** 1(1) **Sequences:** 1(1)
>tr|C6FYA3|C6FYA3_9HIV1 Envelope glycoprotein gp160 OS=Human immunodeficiency virus 1 OX=11676 GN=env PE=3 SV=1
2::C6FYA4 **Mass:** 97121 **Score:** 58 **Matches:** 1(1) **Sequences:** 1(1)
>tr|C6FYA4|C6FYA4_9HIV1 Envelope glycoprotein gp160 OS=Human immunodeficiency virus 1 OX=11676 GN=env PE=3 SV=1
2::C6FYA5 **Mass:** 97107 **Score:** 58 **Matches:** 1(1) **Sequences:** 1(1)
>tr|C6FYA5|C6FYA5_9HIV1 Envelope glycoprotein gp160 OS=Human immunodeficiency virus 1 OX=11676 GN=env PE=3 SV=1
2::C6FYA6 **Mass:** 97083 **Score:** 58 **Matches:** 1(1) **Sequences:** 1(1)
>tr|C6FYA6|C6FYA6_9HIV1 Envelope glycoprotein gp160 OS=Human immunodeficiency virus 1 OX=11676 GN=env PE=3 SV=1
2::C6FYA7 **Mass:** 97093 **Score:** 58 **Matches:** 1(1) **Sequences:** 1(1)
>tr|C6FYA7|C6FYA7_9HIV1 Envelope glycoprotein gp160 OS=Human immunodeficiency virus 1 OX=11676 GN=env PE=3 SV=1
2::C6FYA8 **Mass:** 97150 **Score:** 58 **Matches:** 1(1) **Sequences:** 1(1)
>tr|C6FYA8|C6FYA8_9HIV1 Envelope glycoprotein gp160 OS=Human immunodeficiency virus 1 OX=11676 GN=env PE=3 SV=1
2::C6FYB1 **Mass:** 97148 **Score:** 58 **Matches:** 1(1) **Sequences:** 1(1)
>tr|C6FYB1|C6FYB1_9HIV1 Envelope glycoprotein gp160 OS=Human immunodeficiency virus 1 OX=11676 GN=env PE=3 SV=1
2::C6FYB3 **Mass:** 97108 **Score:** 58 **Matches:** 1(1) **Sequences:** 1(1)
>tr|C6FYB3|C6FYB3_9HIV1 Envelope glycoprotein gp160 OS=Human immunodeficiency virus 1 OX=11676 GN=env PE=3 SV=1
2::C6FYB4 **Mass:** 97120 **Score:** 58 **Matches:** 1(1) **Sequences:** 1(1)
>tr|C6FYB4|C6FYB4_9HIV1 Envelope glycoprotein gp160 OS=Human immunodeficiency virus 1 OX=11676 GN=env PE=3 SV=1
2::C6FYB5 **Mass:** 97120 **Score:** 58 **Matches:** 1(1) **Sequences:** 1(1)
>tr|C6FYB5|C6FYB5_9HIV1 Envelope glycoprotein gp160 OS=Human immunodeficiency virus 1 OX=11676 GN=env PE=3 SV=1
2::C6FYB6 **Mass:** 97148 **Score:** 58 **Matches:** 1(1) **Sequences:** 1(1)
>tr|C6FYB6|C6FYB6_9HIV1 Envelope glycoprotein gp160 OS=Human immunodeficiency virus 1 OX=11676 GN=env PE=3 SV=1
2::C6FYB9 **Mass:** 97106 **Score:** 58 **Matches:** 1(1) **Sequences:** 1(1)
>tr|C6FYB9|C6FYB9_9HIV1 Envelope glycoprotein gp160 OS=Human immunodeficiency virus 1 OX=11676 GN=env PE=3 SV=1
2::C6FYC0 **Mass:** 97132 **Score:** 58 **Matches:** 1(1) **Sequences:** 1(1)
>tr|C6FYC0|C6FYC0_9HIV1 Envelope glycoprotein gp160 OS=Human immunodeficiency virus 1 OX=11676 GN=env PE=3 SV=1
2::C6FZA5 **Mass:** 97136 **Score:** 58 **Matches:** 1(1) **Sequences:** 1(1)
>tr|C6FZA5|C6FZA5_9HIV1 Envelope glycoprotein gp160 OS=Human immunodeficiency virus 1 OX=11676 GN=env PE=3 SV=1
2::C6FZV7 **Mass:** 98727 **Score:** 58 **Matches:** 1(1) **Sequences:** 1(1)
>tr|C6FZV7|C6FZV7_9HIV1 Envelope glycoprotein gp160 OS=Human immunodeficiency virus 1 OX=11676 GN=env PE=3 SV=1
2::C6FZW5 **Mass:** 98755 **Score:** 58 **Matches:** 1(1) **Sequences:** 1(1)
>tr|C6FZW5|C6FZW5_9HIV1 Envelope glycoprotein gp160 OS=Human immunodeficiency virus 1 OX=11676 GN=env PE=3 SV=1
2::C6FZW8 **Mass:** 98757 **Score:** 58 **Matches:** 1(1) **Sequences:** 1(1)
>tr|C6FZW8|C6FZW8_9HIV1 Envelope glycoprotein gp160 OS=Human immunodeficiency virus 1 OX=11676 GN=env PE=3 SV=1
2::C6FZX4 **Mass:** 98737 **Score:** 58 **Matches:** 1(1) **Sequences:** 1(1)
>tr|C6FZX4|C6FZX4_9HIV1 Envelope glycoprotein gp160 OS=Human immunodeficiency virus 1 OX=11676 GN=env PE=3 SV=1
2::C6G0H3 **Mass:** 98026 **Score:** 58 **Matches:** 1(1) **Sequences:** 1(1)
>tr|C6G0H3|C6G0H3_9HIV1 Envelope glycoprotein gp160 OS=Human immunodeficiency virus 1 OX=11676 GN=env PE=3 SV=1
2::C6G0H4 **Mass:** 99148 **Score:** 58 **Matches:** 1(1) **Sequences:** 1(1)
>tr|C6G0H4|C6G0H4_9HIV1 Envelope glycoprotein gp160 OS=Human immunodeficiency virus 1 OX=11676 GN=env PE=3 SV=1
2::C6G0H5 **Mass:** 98010 **Score:** 58 **Matches:** 1(1) **Sequences:** 1(1)
>tr|C6G0H5|C6G0H5_9HIV1 Envelope glycoprotein gp160 OS=Human immunodeficiency virus 1 OX=11676 GN=env PE=3 SV=1
2::C6G0H6 **Mass:** 98060 **Score:** 58 **Matches:** 1(1) **Sequences:** 1(1)
>tr|C6G0H6|C6G0H6_9HIV1 Envelope glycoprotein gp160 OS=Human immunodeficiency virus 1 OX=11676 GN=env PE=3 SV=1
2::C6G0H7 **Mass:** 97967 **Score:** 58 **Matches:** 1(1) **Sequences:** 1(1)
>tr|C6G0H7|C6G0H7_9HIV1 Envelope glycoprotein gp160 OS=Human immunodeficiency virus 1 OX=11676 GN=env PE=3 SV=1
2::C6G0H8 **Mass:** 99119 **Score:** 58 **Matches:** 1(1) **Sequences:** 1(1)
>tr|C6G0H8|C6G0H8_9HIV1 Envelope glycoprotein gp160 OS=Human immunodeficiency virus 1 OX=11676 GN=env PE=3 SV=1
2::C6G0I1 **Mass:** 97905 **Score:** 58 **Matches:** 1(1) **Sequences:** 1(1)
>tr|C6G0I1|C6G0I1_9HIV1 Envelope glycoprotein gp160 OS=Human immunodeficiency virus 1 OX=11676 GN=env PE=3 SV=1
2::C6G0I3 **Mass:** 99077 **Score:** 58 **Matches:** 1(1) **Sequences:** 1(1)
>tr|C6G0I3|C6G0I3_9HIV1 Envelope glycoprotein gp160 OS=Human immunodeficiency virus 1 OX=11676 GN=env PE=3 SV=1
2::C6G0I8 **Mass:** 98054 **Score:** 58 **Matches:** 1(1) **Sequences:** 1(1)
>tr|C6G0I8|C6G0I8_9HIV1 Envelope glycoprotein gp160 OS=Human immunodeficiency virus 1 OX=11676 GN=env PE=3 SV=1
2::H6T9S0 **Mass:** 99564 **Score:** 58 **Matches:** 1(1) **Sequences:** 1(1)
>tr|H6T9S0|H6T9S0_9HIV1 Envelope glycoprotein gp160 OS=Human immunodeficiency virus 1 OX=11676 GN=env PE=3 SV=1
2::H6T9S6 **Mass:** 99371 **Score:** 58 **Matches:** 1(1) **Sequences:** 1(1)
>tr|H6T9S6|H6T9S6_9HIV1 Envelope glycoprotein gp160 OS=Human immunodeficiency virus 1 OX=11676 GN=env PE=3 SV=1
2::H6T9T3 **Mass:** 98117 **Score:** 58 **Matches:** 1(1) **Sequences:** 1(1)
>tr|H6T9T3|H6T9T3_9HIV1 Envelope glycoprotein gp160 OS=Human immunodeficiency virus 1 OX=11676 GN=env PE=3 SV=1
2::I6P2D6 **Mass:** 99529 **Score:** 58 **Matches:** 1(1) **Sequences:** 1(1)
>tr|I6P2D6|I6P2D6_9HIV1 Envelope glycoprotein gp160 OS=Human immunodeficiency virus 1 OX=11676 GN=env PE=3 SV=1
2::I6P5K5 **Mass:** 99174 **Score:** 58 **Matches:** 1(1) **Sequences:** 1(1)
>tr|I6P5K5|I6P5K5_9HIV1 Envelope glycoprotein gp160 OS=Human immunodeficiency virus 1 OX=11676 GN=env PE=3 SV=1
2::I6P6C8 **Mass:** 99204 **Score:** 58 **Matches:** 1(1) **Sequences:** 1(1)
>tr|I6P6C8|I6P6C8_9HIV1 Envelope glycoprotein gp160 OS=Human immunodeficiency virus 1 OX=11676 GN=env PE=3 SV=1
2::I6P6T1 **Mass:** 99520 **Score:** 58 **Matches:** 1(1) **Sequences:** 1(1)
>tr|I6P6T1|I6P6T1_9HIV1 Envelope glycoprotein gp160 OS=Human immunodeficiency virus 1 OX=11676 GN=env PE=3 SV=1
2::I6P6X1 **Mass:** 99590 **Score:** 58 **Matches:** 1(1) **Sequences:** 1(1)
>tr|I6P6X1|I6P6X1_9HIV1 Envelope glycoprotein gp160 OS=Human immunodeficiency virus 1 OX=11676 GN=env PE=3 SV=1
2::I6ZDJ4 **Mass:** 97801 **Score:** 58 **Matches:** 1(1) **Sequences:** 1(1)
>tr|I6ZDJ4|I6ZDJ4_9HIV1 Envelope glycoprotein gp160 OS=Human immunodeficiency virus 1 OX=11676 GN=env PE=3 SV=1
2::I6ZDL5 **Mass:** 97819 **Score:** 58 **Matches:** 1(1) **Sequences:** 1(1)
>tr|I6ZDL5|I6ZDL5_9HIV1 Envelope glycoprotein gp160 OS=Human immunodeficiency virus 1 OX=11676 GN=env PE=3 SV=1
2::I6ZDP9 **Mass:** 97007 **Score:** 58 **Matches:** 1(1) **Sequences:** 1(1)
>tr|I6ZDP9|I6ZDP9_9HIV1 Envelope glycoprotein gp160 OS=Human immunodeficiency virus 1 OX=11676 GN=env PE=3 SV=1
2::I6ZDS8 **Mass:** 97930 **Score:** 58 **Matches:** 1(1) **Sequences:** 1(1)
>tr|I6ZDS8|I6ZDS8_9HIV1 Envelope glycoprotein gp160 OS=Human immunodeficiency virus 1 OX=11676 GN=env PE=3 SV=1
2::I6ZE65 **Mass:** 100211 **Score:** 58 **Matches:** 1(1) **Sequences:** 1(1)
>tr|I6ZE65|I6ZE65_9HIV1 Envelope glycoprotein gp160 OS=Human immunodeficiency virus 1 OX=11676 GN=env PE=3 SV=1
2::I6ZEC1 **Mass:** 99204 **Score:** 58 **Matches:** 1(1) **Sequences:** 1(1)
>tr|I6ZEC1|I6ZEC1_9HIV1 Envelope glycoprotein gp160 (Fragment) OS=Human immunodeficiency virus 1 OX=11676 GN=env PE=3 SV=1
2::I6ZEG3 **Mass:** 100391 **Score:** 58 **Matches:** 1(1) **Sequences:** 1(1)
>tr|I6ZEG3|I6ZEG3_9HIV1 Envelope glycoprotein gp160 OS=Human immunodeficiency virus 1 OX=11676 GN=env PE=3 SV=1
2::I6ZEQ2 **Mass:** 99206 **Score:** 58 **Matches:** 1(1) **Sequences:** 1(1)
>tr|I6ZEQ2|I6ZEQ2_9HIV1 Envelope glycoprotein gp160 OS=Human immunodeficiency virus 1 OX=11676 GN=env PE=3 SV=1
2::I6ZNZ7 **Mass:** 97845 **Score:** 58 **Matches:** 1(1) **Sequences:** 1(1)
>tr|I6ZNZ7|I6ZNZ7_9HIV1 Envelope glycoprotein gp160 OS=Human immunodeficiency virus 1 OX=11676 GN=env PE=3 SV=1
2::I6ZP18 **Mass:** 97944 **Score:** 58 **Matches:** 1(1) **Sequences:** 1(1)
>tr|I6ZP18|I6ZP18_9HIV1 Envelope glycoprotein gp160 OS=Human immunodeficiency virus 1 OX=11676 GN=env PE=3 SV=1
2::I6ZP51 **Mass:** 97468 **Score:** 58 **Matches:** 1(1) **Sequences:** 1(1)
>tr|I6ZP51|I6ZP51_9HIV1 Envelope glycoprotein gp160 OS=Human immunodeficiency virus 1 OX=11676 GN=env PE=3 SV=1
2::I6ZP82 **Mass:** 97964 **Score:** 58 **Matches:** 1(1) **Sequences:** 1(1)
>tr|I6ZP82|I6ZP82_9HIV1 Envelope glycoprotein gp160 OS=Human immunodeficiency virus 1 OX=11676 GN=env PE=3 SV=1
2::I6ZPM6 **Mass:** 98349 **Score:** 58 **Matches:** 1(1) **Sequences:** 1(1)
>tr|I6ZPM6|I6ZPM6_9HIV1 Envelope glycoprotein gp160 (Fragment) OS=Human immunodeficiency virus 1 OX=11676 GN=env PE=3 SV=1
2::I6ZPU1 **Mass:** 99408 **Score:** 58 **Matches:** 1(1) **Sequences:** 1(1)
>tr|I6ZPU1|I6ZPU1_9HIV1 Envelope glycoprotein gp160 (Fragment) OS=Human immunodeficiency virus 1 OX=11676 GN=env PE=3 SV=1
2::I6ZPW0 **Mass:** 100164 **Score:** 58 **Matches:** 1(1) **Sequences:** 1(1)
>tr|I6ZPW0|I6ZPW0_9HIV1 Envelope glycoprotein gp160 OS=Human immunodeficiency virus 1 OX=11676 GN=env PE=3 SV=1
2::I6ZPY9 **Mass:** 98504 **Score:** 58 **Matches:** 1(1) **Sequences:** 1(1)
>tr|I6ZPY9|I6ZPY9_9HIV1 Envelope glycoprotein gp160 OS=Human immunodeficiency virus 1 OX=11676 GN=env PE=3 SV=1
2::Q6E7I2 **Mass:** 98999 **Score:** 58 **Matches:** 1(1) **Sequences:** 1(1)
>tr|Q6E7I2|Q6E7I2_9HIV1 Envelope glycoprotein gp160 OS=Human immunodeficiency virus 1 OX=11676 GN=env PE=3 SV=1

2::Q6S7Y1 **Mass:** 97555 **Score:** 58 **Matches:** 1(1) **Sequences:** 1(1)
>tr|Q6S7Y1|Q6S7Y1_9HIV1 Envelope glycoprotein gp160 OS=Human immunodeficiency virus 1 OX=11676 GN=env PE=3 SV=1
2::Q6S822 **Mass:** 96786 **Score:** 58 **Matches:** 1(1) **Sequences:** 1(1)
>tr|Q6S822|Q6S822_9HIV1 Envelope glycoprotein gp160 OS=Human immunodeficiency virus 1 OX=11676 GN=env PE=3 SV=1
2::Q6X4Q0 **Mass:** 97942 **Score:** 58 **Matches:** 1(1) **Sequences:** 1(1)
>tr|Q6X4Q0|Q6X4Q0_9HIV1 Envelope glycoprotein gp160 OS=Human immunodeficiency virus 1 OX=11676 GN=env PE=3 SV=1
2::Q6X6K8 **Mass:** 98098 **Score:** 58 **Matches:** 1(1) **Sequences:** 1(1)
>tr|Q6X6K8|Q6X6K8_9HIV1 Envelope glycoprotein gp160 OS=Human immunodeficiency virus 1 OX=11676 GN=env PE=3 SV=1
2::U6BGL2 **Mass:** 99838 **Score:** 58 **Matches:** 1(1) **Sequences:** 1(1)
>tr|U6BGL2|U6BGL2_9HIV1 Envelope glycoprotein gp160 OS=Human immunodeficiency virus 1 OX=11676 GN=env PE=3 SV=1
2::U6BGN5 **Mass:** 99020 **Score:** 58 **Matches:** 1(1) **Sequences:** 1(1)
>tr|U6BGN5|U6BGN5_9HIV1 Envelope glycoprotein gp160 OS=Human immunodeficiency virus 1 OX=11676 GN=env PE=3 SV=1
2::U6BQ88 **Mass:** 97620 **Score:** 58 **Matches:** 1(1) **Sequences:** 1(1)
>tr|U6BQ88|U6BQ88_9HIV1 Envelope glycoprotein gp160 OS=Human immunodeficiency virus 1 OX=11676 GN=env PE=3 SV=1
2::U6BQD6 **Mass:** 96696 **Score:** 58 **Matches:** 1(1) **Sequences:** 1(1)
>tr|U6BQD6|U6BQD6_9HIV1 Envelope glycoprotein gp160 OS=Human immunodeficiency virus 1 OX=11676 GN=env PE=3 SV=1
2::U6BQV1 **Mass:** 96943 **Score:** 58 **Matches:** 1(1) **Sequences:** 1(1)
>tr|U6BQV1|U6BQV1_9HIV1 Envelope glycoprotein gp160 OS=Human immunodeficiency virus 1 OX=11676 GN=env PE=3 SV=1
2::U6BR17 **Mass:** 98325 **Score:** 58 **Matches:** 1(1) **Sequences:** 1(1)
>tr|U6BR17|U6BR17_9HIV1 Envelope glycoprotein gp160 OS=Human immunodeficiency virus 1 OX=11676 GN=env PE=3 SV=1
2::U6BRD1 **Mass:** 97277 **Score:** 58 **Matches:** 1(1) **Sequences:** 1(1)
>tr|U6BRD1|U6BRD1_9HIV1 Envelope glycoprotein gp160 OS=Human immunodeficiency virus 1 OX=11676 GN=env PE=3 SV=1
2::U6BRF9 **Mass:** 96634 **Score:** 58 **Matches:** 1(1) **Sequences:** 1(1)
>tr|U6BRF9|U6BRF9_9HIV1 Envelope glycoprotein gp160 OS=Human immunodeficiency virus 1 OX=11676 GN=env PE=3 SV=1
2::U6BRT5 **Mass:** 97262 **Score:** 58 **Matches:** 1(1) **Sequences:** 1(1)
>tr|U6BRT5|U6BRT5_9HIV1 Envelope glycoprotein gp160 OS=Human immunodeficiency virus 1 OX=11676 GN=env PE=3 SV=1
2::U6BRZ5 **Mass:** 99054 **Score:** 58 **Matches:** 1(1) **Sequences:** 1(1)
>tr|U6BRZ5|U6BRZ5_9HIV1 Envelope glycoprotein gp160 OS=Human immunodeficiency virus 1 OX=11676 GN=env PE=3 SV=1
2::U6BTY4 **Mass:** 98415 **Score:** 58 **Matches:** 1(1) **Sequences:** 1(1)
>tr|U6BTY4|U6BTY4_9HIV1 Envelope glycoprotein gp160 OS=Human immunodeficiency virus 1 OX=11676 GN=env PE=3 SV=1
2::U6BU16 **Mass:** 96806 **Score:** 58 **Matches:** 1(1) **Sequences:** 1(1)
>tr|U6BU16|U6BU16_9HIV1 Envelope glycoprotein gp160 (Fragment) OS=Human immunodeficiency virus 1 OX=11676 GN=env PE=3 SV=1
2::U6BUA8 **Mass:** 98420 **Score:** 58 **Matches:** 1(1) **Sequences:** 1(1)
>tr|U6BUA8|U6BUA8_9HIV1 Envelope glycoprotein gp160 OS=Human immunodeficiency virus 1 OX=11676 GN=env PE=3 SV=1
2::U6BUX2 **Mass:** 94708 **Score:** 58 **Matches:** 1(1) **Sequences:** 1(1)
>tr|U6BUX2|U6BUX2_9HIV1 Envelope glycoprotein gp160 OS=Human immunodeficiency virus 1 OX=11676 GN=env PE=3 SV=1
2::B7SGW0 **Mass:** 99044 **Score:** 58 **Matches:** 1(1) **Sequences:** 1(1)
>tr|B7SGW0|B7SGW0_9HIV1 Envelope glycoprotein gp160 OS=Human immunodeficiency virus 1 OX=11676 GN=env PE=3 SV=1
2::C7G295 **Mass:** 98547 **Score:** 58 **Matches:** 1(1) **Sequences:** 1(1)
>tr|C7G295|C7G295_9HIV1 Envelope glycoprotein gp160 OS=Human immunodeficiency virus 1 OX=11676 GN=env PE=3 SV=1
2::C7G2A4 **Mass:** 98462 **Score:** 58 **Matches:** 1(1) **Sequences:** 1(1)
>tr|C7G2A4|C7G2A4_9HIV1 Envelope glycoprotein gp160 OS=Human immunodeficiency virus 1 OX=11676 GN=env PE=3 SV=1
2::C7G2B3 **Mass:** 98474 **Score:** 58 **Matches:** 1(1) **Sequences:** 1(1)
>tr|C7G2B3|C7G2B3_9HIV1 Envelope glycoprotein gp160 OS=Human immunodeficiency virus 1 OX=11676 GN=env PE=3 SV=1
2::D7R5J2 **Mass:** 95749 **Score:** 58 **Matches:** 1(1) **Sequences:** 1(1)
>tr|D7R5J2|D7R5J2_9HIV1 Envelope glycoprotein gp160 OS=Human immunodeficiency virus 1 OX=11676 GN=env PE=3 SV=1
2::D7R5J3 **Mass:** 96454 **Score:** 58 **Matches:** 1(1) **Sequences:** 1(1)
>tr|D7R5J3|D7R5J3_9HIV1 Envelope glycoprotein gp160 OS=Human immunodeficiency virus 1 OX=11676 GN=env PE=3 SV=1
2::D7R5J4 **Mass:** 95949 **Score:** 58 **Matches:** 1(1) **Sequences:** 1(1)
>tr|D7R5J4|D7R5J4_9HIV1 Envelope glycoprotein gp160 OS=Human immunodeficiency virus 1 OX=11676 GN=env PE=3 SV=1
2::D7R5J5 **Mass:** 95986 **Score:** 58 **Matches:** 1(1) **Sequences:** 1(1)
>tr|D7R5J5|D7R5J5_9HIV1 Envelope glycoprotein gp160 OS=Human immunodeficiency virus 1 OX=11676 GN=env PE=3 SV=1
2::D7R5J6 **Mass:** 96175 **Score:** 58 **Matches:** 1(1) **Sequences:** 1(1)
>tr|D7R5J6|D7R5J6_9HIV1 Envelope glycoprotein gp160 OS=Human immunodeficiency virus 1 OX=11676 GN=env PE=3 SV=1
2::D7R5J8 **Mass:** 98479 **Score:** 58 **Matches:** 1(1) **Sequences:** 1(1)
>tr|D7R5J8|D7R5J8_9HIV1 Envelope glycoprotein gp160 OS=Human immunodeficiency virus 1 OX=11676 GN=env PE=3 SV=1
2::D7R5J9 **Mass:** 98419 **Score:** 58 **Matches:** 1(1) **Sequences:** 1(1)
>tr|D7R5J9|D7R5J9_9HIV1 Envelope glycoprotein gp160 OS=Human immunodeficiency virus 1 OX=11676 GN=env PE=3 SV=1
2::D7R5K0 **Mass:** 96221 **Score:** 58 **Matches:** 1(1) **Sequences:** 1(1)
>tr|D7R5K0|D7R5K0_9HIV1 Envelope glycoprotein gp160 OS=Human immunodeficiency virus 1 OX=11676 GN=env PE=3 SV=1
2::D7R5K1 **Mass:** 96099 **Score:** 58 **Matches:** 1(1) **Sequences:** 1(1)
>tr|D7R5K1|D7R5K1_9HIV1 Envelope glycoprotein gp160 OS=Human immunodeficiency virus 1 OX=11676 GN=env PE=3 SV=1
2::D7R5K2 **Mass:** 96048 **Score:** 58 **Matches:** 1(1) **Sequences:** 1(1)
>tr|D7R5K2|D7R5K2_9HIV1 Envelope glycoprotein gp160 OS=Human immunodeficiency virus 1 OX=11676 GN=env PE=3 SV=1
2::D7R5K3 **Mass:** 98030 **Score:** 58 **Matches:** 1(1) **Sequences:** 1(1)
>tr|D7R5K3|D7R5K3_9HIV1 Envelope glycoprotein gp160 OS=Human immunodeficiency virus 1 OX=11676 GN=env PE=3 SV=1
2::D7R5K4 **Mass:** 96206 **Score:** 58 **Matches:** 1(1) **Sequences:** 1(1)
>tr|D7R5K4|D7R5K4_9HIV1 Envelope glycoprotein gp160 (Fragment) OS=Human immunodeficiency virus 1 OX=11676 GN=env PE=3 SV=1
2::D7R5K5 **Mass:** 98287 **Score:** 58 **Matches:** 1(1) **Sequences:** 1(1)
>tr|D7R5K5|D7R5K5_9HIV1 Envelope glycoprotein gp160 OS=Human immunodeficiency virus 1 OX=11676 GN=env PE=3 SV=1
2::D7R5K6 **Mass:** 98322 **Score:** 58 **Matches:** 1(1) **Sequences:** 1(1)
>tr|D7R5K6|D7R5K6_9HIV1 Envelope glycoprotein gp160 OS=Human immunodeficiency virus 1 OX=11676 GN=env PE=3 SV=1
2::D7R5K7 **Mass:** 98297 **Score:** 58 **Matches:** 1(1) **Sequences:** 1(1)
>tr|D7R5K7|D7R5K7_9HIV1 Envelope glycoprotein gp160 OS=Human immunodeficiency virus 1 OX=11676 GN=env PE=3 SV=1
2::D7R5K8 **Mass:** 98204 **Score:** 58 **Matches:** 1(1) **Sequences:** 1(1)
>tr|D7R5K8|D7R5K8_9HIV1 Envelope glycoprotein gp160 OS=Human immunodeficiency virus 1 OX=11676 GN=env PE=3 SV=1
2::D7R5K9 **Mass:** 98109 **Score:** 58 **Matches:** 1(1) **Sequences:** 1(1)
>tr|D7R5K9|D7R5K9_9HIV1 Envelope glycoprotein gp160 OS=Human immunodeficiency virus 1 OX=11676 GN=env PE=3 SV=1
2::D7R5L0 **Mass:** 98290 **Score:** 58 **Matches:** 1(1) **Sequences:** 1(1)
>tr|D7R5L0|D7R5L0_9HIV1 Envelope glycoprotein gp160 OS=Human immunodeficiency virus 1 OX=11676 GN=env PE=3 SV=1
2::D7RZ63 **Mass:** 97618 **Score:** 58 **Matches:** 1(1) **Sequences:** 1(1)
>tr|D7RZ63|D7RZ63_9HIV1 Envelope glycoprotein gp160 OS=Human immunodeficiency virus 1 OX=11676 GN=env PE=3 SV=1
2::D7RZA2 **Mass:** 98889 **Score:** 58 **Matches:** 1(1) **Sequences:** 1(1)
>tr|D7RZA2|D7RZA2_9HIV1 Envelope glycoprotein gp160 (Fragment) OS=Human immunodeficiency virus 1 OX=11676 GN=env PE=3 SV=1
2::D7RZA6 **Mass:** 100890 **Score:** 58 **Matches:** 1(1) **Sequences:** 1(1)
>tr|D7RZA6|D7RZA6_9HIV1 Envelope glycoprotein gp160 OS=Human immunodeficiency virus 1 OX=11676 GN=env PE=3 SV=1
2::D7S0Z5 **Mass:** 97923 **Score:** 58 **Matches:** 1(1) **Sequences:** 1(1)
>tr|D7S0Z5|D7S0Z5_9HIV1 Envelope glycoprotein gp160 OS=Human immunodeficiency virus 1 OX=11676 GN=env PE=3 SV=1
2::D7S130 **Mass:** 99254 **Score:** 58 **Matches:** 1(1) **Sequences:** 1(1)
>tr|D7S130|D7S130_9HIV1 Envelope glycoprotein gp160 OS=Human immunodeficiency virus 1 OX=11676 GN=env PE=3 SV=1
2::D7S1C8 **Mass:** 98144 **Score:** 58 **Matches:** 1(1) **Sequences:** 1(1)
>tr|D7S1C8|D7S1C8_9HIV1 Envelope glycoprotein gp160 OS=Human immunodeficiency virus 1 OX=11676 GN=env PE=3 SV=1
2::D7S1D5 **Mass:** 99293 **Score:** 58 **Matches:** 1(1) **Sequences:** 1(1)
>tr|D7S1D5|D7S1D5_9HIV1 Envelope glycoprotein gp160 (Fragment) OS=Human immunodeficiency virus 1 OX=11676 GN=env PE=3 SV=1
2::E7EFG5 **Mass:** 100224 **Score:** 58 **Matches:** 1(1) **Sequences:** 1(1)
>tr|E7EFG5|E7EFG5_9HIV1 Envelope glycoprotein gp160 OS=Human immunodeficiency virus 1 OX=11676 GN=env PE=3 SV=1
2::E7EFG8 **Mass:** 99509 **Score:** 58 **Matches:** 1(1) **Sequences:** 1(1)
>tr|E7EFG8|E7EFG8_9HIV1 Envelope glycoprotein gp160 OS=Human immunodeficiency virus 1 OX=11676 GN=env PE=3 SV=1
2::E7EFH1 **Mass:** 100167 **Score:** 58 **Matches:** 1(1) **Sequences:** 1(1)
>tr|E7EFH1|E7EFH1_9HIV1 Envelope glycoprotein gp160 OS=Human immunodeficiency virus 1 OX=11676 GN=env PE=3 SV=1
2::E7EFH8 **Mass:** 100293 **Score:** 58 **Matches:** 1(1) **Sequences:** 1(1)
>tr|E7EFH8|E7EFH8_9HIV1 Envelope glycoprotein gp160 OS=Human immunodeficiency virus 1 OX=11676 GN=env PE=3 SV=1
2::E7EFI1 **Mass:** 100252 **Score:** 58 **Matches:** 1(1) **Sequences:** 1(1)
>tr|E7EFI1|E7EFI1_9HIV1 Envelope glycoprotein gp160 OS=Human immunodeficiency virus 1 OX=11676 GN=env PE=3 SV=1
2::E7EFI4 **Mass:** 99354 **Score:** 58 **Matches:** 1(1) **Sequences:** 1(1)
>tr|E7EFI4|E7EFI4_9HIV1 Envelope glycoprotein gp160 OS=Human immunodeficiency virus 1 OX=11676 GN=env PE=3 SV=1
2::E7EFI7 **Mass:** 100296 **Score:** 58 **Matches:** 1(1) **Sequences:** 1(1)
>tr|E7EFI7|E7EFI7_9HIV1 Envelope glycoprotein gp160 OS=Human immunodeficiency virus 1 OX=11676 GN=env PE=3 SV=1
2::E7EFJ3 **Mass:** 100181 **Score:** 58 **Matches:** 1(1) **Sequences:** 1(1)
>tr|E7EFJ3|E7EFJ3_9HIV1 Envelope glycoprotein gp160 OS=Human immunodeficiency virus 1 OX=11676 GN=env PE=3 SV=1

2::E7EFJ7 **Mass:** 100095 **Score:** 58 **Matches:** 1(1) **Sequences:** 1(1)
>tr|E7EFJ7|E7EFJ7_9HIV1 Envelope glycoprotein gp160 OS=Human immunodeficiency virus 1 OX=11676 GN=env PE=3 SV=1

2::E7EI33 **Mass:** 97895 **Score:** 58 **Matches:** 1(1) **Sequences:** 1(1)
>tr|E7EI33|E7EI33_9HIV1 Envelope glycoprotein gp160 OS=Human immunodeficiency virus 1 OX=11676 GN=env PE=3 SV=1

2::E7EI34 **Mass:** 97806 **Score:** 58 **Matches:** 1(1) **Sequences:** 1(1)
>tr|E7EI34|E7EI34_9HIV1 Envelope glycoprotein gp160 OS=Human immunodeficiency virus 1 OX=11676 GN=env PE=3 SV=1

2::E7EI35 **Mass:** 97981 **Score:** 58 **Matches:** 1(1) **Sequences:** 1(1)
>tr|E7EI35|E7EI35_9HIV1 Envelope glycoprotein gp160 OS=Human immunodeficiency virus 1 OX=11676 GN=env PE=3 SV=1

2::E7EI36 **Mass:** 97909 **Score:** 58 **Matches:** 1(1) **Sequences:** 1(1)
>tr|E7EI36|E7EI36_9HIV1 Envelope glycoprotein gp160 OS=Human immunodeficiency virus 1 OX=11676 GN=env PE=3 SV=1

2::E7EI37 **Mass:** 98008 **Score:** 58 **Matches:** 1(1) **Sequences:** 1(1)
>tr|E7EI37|E7EI37_9HIV1 Envelope glycoprotein gp160 OS=Human immunodeficiency virus 1 OX=11676 GN=env PE=3 SV=1

2::E7EI39 **Mass:** 97908 **Score:** 58 **Matches:** 1(1) **Sequences:** 1(1)
>tr|E7EI39|E7EI39_9HIV1 Envelope glycoprotein gp160 OS=Human immunodeficiency virus 1 OX=11676 GN=env PE=3 SV=1

2::E7EI40 **Mass:** 97981 **Score:** 58 **Matches:** 1(1) **Sequences:** 1(1)
>tr|E7EI40|E7EI40_9HIV1 Envelope glycoprotein gp160 OS=Human immunodeficiency virus 1 OX=11676 GN=env PE=3 SV=1

2::E7EI41 **Mass:** 97909 **Score:** 58 **Matches:** 1(1) **Sequences:** 1(1)
>tr|E7EI41|E7EI41_9HIV1 Envelope glycoprotein gp160 OS=Human immunodeficiency virus 1 OX=11676 GN=env PE=3 SV=1

2::E7EI42 **Mass:** 97923 **Score:** 58 **Matches:** 1(1) **Sequences:** 1(1)
>tr|E7EI42|E7EI42_9HIV1 Envelope glycoprotein gp160 OS=Human immunodeficiency virus 1 OX=11676 GN=env PE=3 SV=1

2::E7EI44 **Mass:** 97921 **Score:** 58 **Matches:** 1(1) **Sequences:** 1(1)
>tr|E7EI44|E7EI44_9HIV1 Envelope glycoprotein gp160 OS=Human immunodeficiency virus 1 OX=11676 GN=env PE=3 SV=1

2::E7EI45 **Mass:** 97981 **Score:** 58 **Matches:** 1(1) **Sequences:** 1(1)
>tr|E7EI45|E7EI45_9HIV1 Envelope glycoprotein gp160 OS=Human immunodeficiency virus 1 OX=11676 GN=env PE=3 SV=1

2::E7EI46 **Mass:** 97865 **Score:** 58 **Matches:** 1(1) **Sequences:** 1(1)
>tr|E7EI46|E7EI46_9HIV1 Envelope glycoprotein gp160 OS=Human immunodeficiency virus 1 OX=11676 GN=env PE=3 SV=1

2::E7EI47 **Mass:** 97923 **Score:** 58 **Matches:** 1(1) **Sequences:** 1(1)
>tr|E7EI47|E7EI47_9HIV1 Envelope glycoprotein gp160 OS=Human immunodeficiency virus 1 OX=11676 GN=env PE=3 SV=1

2::E7EI48 **Mass:** 97967 **Score:** 58 **Matches:** 1(1) **Sequences:** 1(1)
>tr|E7EI48|E7EI48_9HIV1 Envelope glycoprotein gp160 OS=Human immunodeficiency virus 1 OX=11676 GN=env PE=3 SV=1

2::E7EI49 **Mass:** 97908 **Score:** 58 **Matches:** 1(1) **Sequences:** 1(1)
>tr|E7EI49|E7EI49_9HIV1 Envelope glycoprotein gp160 OS=Human immunodeficiency virus 1 OX=11676 GN=env PE=3 SV=1

2::E7EI50 **Mass:** 97923 **Score:** 58 **Matches:** 1(1) **Sequences:** 1(1)
>tr|E7EI50|E7EI50_9HIV1 Envelope glycoprotein gp160 OS=Human immunodeficiency virus 1 OX=11676 GN=env PE=3 SV=1

2::I7A9E7 **Mass:** 97788 **Score:** 58 **Matches:** 1(1) **Sequences:** 1(1)
>tr|I7A9E7|I7A9E7_9HIV1 Envelope glycoprotein gp160 OS=Human immunodeficiency virus 1 OX=11676 GN=env PE=3 SV=1

2::I7A9G7 **Mass:** 97862 **Score:** 58 **Matches:** 1(1) **Sequences:** 1(1)
>tr|I7A9G7|I7A9G7_9HIV1 Envelope glycoprotein gp160 OS=Human immunodeficiency virus 1 OX=11676 GN=env PE=3 SV=1

2::I7A9Q8 **Mass:** 97058 **Score:** 58 **Matches:** 1(1) **Sequences:** 1(1)
>tr|I7A9Q8|I7A9Q8_9HIV1 Envelope glycoprotein gp160 (Fragment) OS=Human immunodeficiency virus 1 OX=11676 GN=env PE=3 SV=1

2::I7A9S6 **Mass:** 97333 **Score:** 58 **Matches:** 1(1) **Sequences:** 1(1)
>tr|I7A9S6|I7A9S6_9HIV1 Envelope glycoprotein gp160 OS=Human immunodeficiency virus 1 OX=11676 GN=env PE=3 SV=1

2::I7AA65 **Mass:** 99244 **Score:** 58 **Matches:** 1(1) **Sequences:** 1(1)
>tr|I7AA65|I7AA65_9HIV1 Envelope glycoprotein gp160 (Fragment) OS=Human immunodeficiency virus 1 OX=11676 GN=env PE=3 SV=1

2::I7AA88 **Mass:** 100320 **Score:** 58 **Matches:** 1(1) **Sequences:** 1(1)
>tr|I7AA88|I7AA88_9HIV1 Envelope glycoprotein gp160 (Fragment) OS=Human immunodeficiency virus 1 OX=11676 GN=env PE=3 SV=1

2::I7AAE2 **Mass:** 99738 **Score:** 58 **Matches:** 1(1) **Sequences:** 1(1)
>tr|I7AAE2|I7AAE2_9HIV1 Envelope glycoprotein gp160 OS=Human immunodeficiency virus 1 OX=11676 GN=env PE=3 SV=1

2::I7AAJ5 **Mass:** 98288 **Score:** 58 **Matches:** 1(1) **Sequences:** 1(1)
>tr|I7AAJ5|I7AAJ5_9HIV1 Envelope glycoprotein gp160 OS=Human immunodeficiency virus 1 OX=11676 GN=env PE=3 SV=1

2::I7AIE9 **Mass:** 95098 **Score:** 58 **Matches:** 1(1) **Sequences:** 1(1)
>tr|I7AIE9|I7AIE9_9HIV1 Envelope glycoprotein gp160 OS=Human immunodeficiency virus 1 OX=11676 GN=env PE=3 SV=1

2::I7AIR5 **Mass:** 99361 **Score:** 58 **Matches:** 1(1) **Sequences:** 1(1)
>tr|I7AIR5|I7AIR5_9HIV1 Envelope glycoprotein gp160 (Fragment) OS=Human immunodeficiency virus 1 OX=11676 GN=env PE=3 SV=1

2::I7AIU1 **Mass:** 100150 **Score:** 58 **Matches:** 1(1) **Sequences:** 1(1)
>tr|I7AIU1|I7AIU1_9HIV1 Envelope glycoprotein gp160 OS=Human immunodeficiency virus 1 OX=11676 GN=env PE=3 SV=1

2::I7AIZ2 **Mass:** 99839 **Score:** 58 **Matches:** 1(1) **Sequences:** 1(1)
>tr|I7AIZ2|I7AIZ2_9HIV1 Envelope glycoprotein gp160 OS=Human immunodeficiency virus 1 OX=11676 GN=env PE=3 SV=1

2::I7ALM5 **Mass:** 97844 **Score:** 58 **Matches:** 1(1) **Sequences:** 1(1)
>tr|I7ALM5|I7ALM5_9HIV1 Envelope glycoprotein gp160 OS=Human immunodeficiency virus 1 OX=11676 GN=env PE=3 SV=1

2::I7ALQ1 **Mass:** 97818 **Score:** 58 **Matches:** 1(1) **Sequences:** 1(1)
>tr|I7ALQ1|I7ALQ1_9HIV1 Envelope glycoprotein gp160 OS=Human immunodeficiency virus 1 OX=11676 GN=env PE=3 SV=1

2::I7ALS6 **Mass:** 97871 **Score:** 58 **Matches:** 1(1) **Sequences:** 1(1)
>tr|I7ALS6|I7ALS6_9HIV1 Envelope glycoprotein gp160 OS=Human immunodeficiency virus 1 OX=11676 GN=env PE=3 SV=1

2::I7AM10 **Mass:** 98232 **Score:** 58 **Matches:** 1(1) **Sequences:** 1(1)
>tr|I7AM10|I7AM10_9HIV1 Envelope glycoprotein gp160 OS=Human immunodeficiency virus 1 OX=11676 GN=env PE=3 SV=1

2::I7AMH2 **Mass:** 99412 **Score:** 58 **Matches:** 1(1) **Sequences:** 1(1)
>tr|I7AMH2|I7AMH2_9HIV1 Envelope glycoprotein gp160 (Fragment) OS=Human immunodeficiency virus 1 OX=11676 GN=env PE=3 SV=1

2::I7AMM9 **Mass:** 99813 **Score:** 58 **Matches:** 1(1) **Sequences:** 1(1)
>tr|I7AMM9|I7AMM9_9HIV1 Envelope glycoprotein gp160 (Fragment) OS=Human immunodeficiency virus 1 OX=11676 GN=env PE=3 SV=1

2::I7AMQ4 **Mass:** 99980 **Score:** 58 **Matches:** 1(1) **Sequences:** 1(1)
>tr|I7AMQ4|I7AMQ4_9HIV1 Envelope glycoprotein gp160 OS=Human immunodeficiency virus 1 OX=11676 GN=env PE=3 SV=1

2::L7WD59 **Mass:** 97012 **Score:** 58 **Matches:** 1(1) **Sequences:** 1(1)
>tr|L7WD59|L7WD59_9HIV1 Envelope glycoprotein gp160 OS=Human immunodeficiency virus 1 OX=11676 GN=env PE=3 SV=1

2::L7WD64 **Mass:** 96990 **Score:** 58 **Matches:** 1(1) **Sequences:** 1(1)
>tr|L7WD64|L7WD64_9HIV1 Envelope glycoprotein gp160 OS=Human immunodeficiency virus 1 OX=11676 GN=env PE=3 SV=1

2::L7WD70 **Mass:** 97038 **Score:** 58 **Matches:** 1(1) **Sequences:** 1(1)
>tr|L7WD70|L7WD70_9HIV1 Envelope glycoprotein gp160 OS=Human immunodeficiency virus 1 OX=11676 GN=env PE=3 SV=1

2::L7WD76 **Mass:** 97012 **Score:** 58 **Matches:** 1(1) **Sequences:** 1(1)
>tr|L7WD76|L7WD76_9HIV1 Envelope glycoprotein gp160 OS=Human immunodeficiency virus 1 OX=11676 GN=env PE=3 SV=1

2::L7WD82 **Mass:** 97052 **Score:** 58 **Matches:** 1(1) **Sequences:** 1(1)
>tr|L7WD82|L7WD82_9HIV1 Envelope glycoprotein gp160 OS=Human immunodeficiency virus 1 OX=11676 GN=env PE=3 SV=1

2::L7WD88 **Mass:** 96978 **Score:** 58 **Matches:** 1(1) **Sequences:** 1(1)
>tr|L7WD88|L7WD88_9HIV1 Envelope glycoprotein gp160 OS=Human immunodeficiency virus 1 OX=11676 GN=env PE=3 SV=1

2::L7WD93 **Mass:** 96981 **Score:** 58 **Matches:** 1(1) **Sequences:** 1(1)
>tr|L7WD93|L7WD93_9HIV1 Envelope glycoprotein gp160 OS=Human immunodeficiency virus 1 OX=11676 GN=env PE=3 SV=1

2::L7WG68 **Mass:** 98446 **Score:** 58 **Matches:** 1(1) **Sequences:** 1(1)
>tr|L7WG68|L7WG68_9HIV1 Envelope glycoprotein gp160 OS=Human immunodeficiency virus 1 OX=11676 GN=env PE=3 SV=1

2::L7WG74 **Mass:** 98471 **Score:** 58 **Matches:** 1(1) **Sequences:** 1(1)
>tr|L7WG74|L7WG74_9HIV1 Envelope glycoprotein gp160 OS=Human immunodeficiency virus 1 OX=11676 GN=env PE=3 SV=1

2::L7WG80 **Mass:** 98494 **Score:** 58 **Matches:** 1(1) **Sequences:** 1(1)
>tr|L7WG80|L7WG80_9HIV1 Envelope glycoprotein gp160 OS=Human immunodeficiency virus 1 OX=11676 GN=env PE=3 SV=1

2::L7WG85 **Mass:** 99009 **Score:** 58 **Matches:** 1(1) **Sequences:** 1(1)
>tr|L7WG85|L7WG85_9HIV1 Envelope glycoprotein gp160 OS=Human immunodeficiency virus 1 OX=11676 GN=env PE=3 SV=1

2::L7WG92 **Mass:** 99143 **Score:** 58 **Matches:** 1(1) **Sequences:** 1(1)
>tr|L7WG92|L7WG92_9HIV1 Envelope glycoprotein gp160 OS=Human immunodeficiency virus 1 OX=11676 GN=env PE=3 SV=1

2::L7WGE1 **Mass:** 96967 **Score:** 58 **Matches:** 1(1) **Sequences:** 1(1)
>tr|L7WGE1|L7WGE1_9HIV1 Envelope glycoprotein gp160 OS=Human immunodeficiency virus 1 OX=11676 GN=env PE=3 SV=1

2::L7WGN5 **Mass:** 95388 **Score:** 58 **Matches:** 1(1) **Sequences:** 1(1)
>tr|L7WGN5|L7WGN5_9HIV1 Envelope glycoprotein gp160 OS=Human immunodeficiency virus 1 OX=11676 GN=env PE=3 SV=1

2::L7WH08 **Mass:** 97096 **Score:** 58 **Matches:** 1(1) **Sequences:** 1(1)
>tr|L7WH08|L7WH08_9HIV1 Envelope glycoprotein gp160 OS=Human immunodeficiency virus 1 OX=11676 GN=env PE=3 SV=1

2::L7WH13 **Mass:** 96953 **Score:** 58 **Matches:** 1(1) **Sequences:** 1(1)
>tr|L7WH13|L7WH13_9HIV1 Envelope glycoprotein gp160 OS=Human immunodeficiency virus 1 OX=11676 GN=env PE=3 SV=1

2::L7WH18 **Mass:** 96986 **Score:** 58 **Matches:** 1(1) **Sequences:** 1(1)
>tr|L7WH18|L7WH18_9HIV1 Envelope glycoprotein gp160 OS=Human immunodeficiency virus 1 OX=11676 GN=env PE=3 SV=1

2::L7WH23 **Mass:** 97124 **Score:** 58 **Matches:** 1(1) **Sequences:** 1(1)
>tr|L7WH23|L7WH23_9HIV1 Envelope glycoprotein gp160 OS=Human immunodeficiency virus 1 OX=11676 GN=env PE=3 SV=1

2::L7WH27 **Mass:** 97083 **Score:** 58 **Matches:** 1(1) **Sequences:** 1(1)
>tr|L7WH27|L7WH27_9HIV1 Envelope glycoprotein gp160 OS=Human immunodeficiency virus 1 OX=11676 GN=env PE=3 SV=1

2::L7WH32 **Mass:** 97034 **Score:** 58 **Matches:** 1(1) **Sequences:** 1(1)
>tr|L7WH32|L7WH32_9HIV1 Envelope glycoprotein gp160 OS=Human immunodeficiency virus 1 OX=11676 GN=env PE=3 SV=1
2::L7WH43 **Mass:** 97238 **Score:** 58 **Matches:** 1(1) **Sequences:** 1(1)
>tr|L7WH43|L7WH43_9HIV1 Envelope glycoprotein gp160 OS=Human immunodeficiency virus 1 OX=11676 GN=env PE=3 SV=1
2::L7WHE4 **Mass:** 97036 **Score:** 58 **Matches:** 1(1) **Sequences:** 1(1)
>tr|L7WHE4|L7WHE4_9HIV1 Envelope glycoprotein gp160 OS=Human immunodeficiency virus 1 OX=11676 GN=env PE=3 SV=1
2::L7WHF1 **Mass:** 97039 **Score:** 58 **Matches:** 1(1) **Sequences:** 1(1)
>tr|L7WHF1|L7WHF1_9HIV1 Envelope glycoprotein gp160 OS=Human immunodeficiency virus 1 OX=11676 GN=env PE=3 SV=1
2::L7WHF7 **Mass:** 96992 **Score:** 58 **Matches:** 1(1) **Sequences:** 1(1)
>tr|L7WHF7|L7WHF7_9HIV1 Envelope glycoprotein gp160 OS=Human immunodeficiency virus 1 OX=11676 GN=env PE=3 SV=1
2::L7WHG4 **Mass:** 97156 **Score:** 58 **Matches:** 1(1) **Sequences:** 1(1)
>tr|L7WHG4|L7WHG4_9HIV1 Envelope glycoprotein gp160 OS=Human immunodeficiency virus 1 OX=11676 GN=env PE=3 SV=1
2::L7WHG8 **Mass:** 97024 **Score:** 58 **Matches:** 1(1) **Sequences:** 1(1)
>tr|L7WHG8|L7WHG8_9HIV1 Envelope glycoprotein gp160 OS=Human immunodeficiency virus 1 OX=11676 GN=env PE=3 SV=1
2::L7WHH4 **Mass:** 96994 **Score:** 58 **Matches:** 1(1) **Sequences:** 1(1)
>tr|L7WHH4|L7WHH4_9HIV1 Envelope glycoprotein gp160 OS=Human immunodeficiency virus 1 OX=11676 GN=env PE=3 SV=1
2::L7WHI0 **Mass:** 97148 **Score:** 58 **Matches:** 1(1) **Sequences:** 1(1)
>tr|L7WHI0|L7WHI0_9HIV1 Envelope glycoprotein gp160 OS=Human immunodeficiency virus 1 OX=11676 GN=env PE=3 SV=1
2::L7WHU5 **Mass:** 96975 **Score:** 58 **Matches:** 1(1) **Sequences:** 1(1)
>tr|L7WHU5|L7WHU5_9HIV1 Envelope glycoprotein gp160 OS=Human immunodeficiency virus 1 OX=11676 GN=env PE=3 SV=1
2::L7WHU8 **Mass:** 97003 **Score:** 58 **Matches:** 1(1) **Sequences:** 1(1)
>tr|L7WHU8|L7WHU8_9HIV1 Envelope glycoprotein gp160 OS=Human immunodeficiency virus 1 OX=11676 GN=env PE=3 SV=1
2::L7WHV3 **Mass:** 97038 **Score:** 58 **Matches:** 1(1) **Sequences:** 1(1)
>tr|L7WHV3|L7WHV3_9HIV1 Envelope glycoprotein gp160 OS=Human immunodeficiency virus 1 OX=11676 GN=env PE=3 SV=1
2::L7WHV6 **Mass:** 97009 **Score:** 58 **Matches:** 1(1) **Sequences:** 1(1)
>tr|L7WHV6|L7WHV6_9HIV1 Envelope glycoprotein gp160 OS=Human immunodeficiency virus 1 OX=11676 GN=env PE=3 SV=1
2::L7WHW0 **Mass:** 96946 **Score:** 58 **Matches:** 1(1) **Sequences:** 1(1)
>tr|L7WHW0|L7WHW0_9HIV1 Envelope glycoprotein gp160 OS=Human immunodeficiency virus 1 OX=11676 GN=env PE=3 SV=1
2::L7WHW5 **Mass:** 96933 **Score:** 58 **Matches:** 1(1) **Sequences:** 1(1)
>tr|L7WHW5|L7WHW5_9HIV1 Envelope glycoprotein gp160 OS=Human immunodeficiency virus 1 OX=11676 GN=env PE=3 SV=1
2::L7WHW8 **Mass:** 96989 **Score:** 58 **Matches:** 1(1) **Sequences:** 1(1)
>tr|L7WHW8|L7WHW8_9HIV1 Envelope glycoprotein gp160 OS=Human immunodeficiency virus 1 OX=11676 GN=env PE=3 SV=1
2::L7WJS5 **Mass:** 98271 **Score:** 58 **Matches:** 1(1) **Sequences:** 1(1)
>tr|L7WJS5|L7WJS5_9HIV1 Envelope glycoprotein gp160 OS=Human immunodeficiency virus 1 OX=11676 GN=env PE=3 SV=1
2::L7WJT0 **Mass:** 98969 **Score:** 58 **Matches:** 1(1) **Sequences:** 1(1)
>tr|L7WJT0|L7WJT0_9HIV1 Envelope glycoprotein gp160 OS=Human immunodeficiency virus 1 OX=11676 GN=env PE=3 SV=1
2::L7WJT4 **Mass:** 98838 **Score:** 58 **Matches:** 1(1) **Sequences:** 1(1)
>tr|L7WJT4|L7WJT4_9HIV1 Envelope glycoprotein gp160 OS=Human immunodeficiency virus 1 OX=11676 GN=env PE=3 SV=1
2::L7WJT7 **Mass:** 98891 **Score:** 58 **Matches:** 1(1) **Sequences:** 1(1)
>tr|L7WJT7|L7WJT7_9HIV1 Envelope glycoprotein gp160 OS=Human immunodeficiency virus 1 OX=11676 GN=env PE=3 SV=1
2::L7WJU2 **Mass:** 98860 **Score:** 58 **Matches:** 1(1) **Sequences:** 1(1)
>tr|L7WJU2|L7WJU2_9HIV1 Envelope glycoprotein gp160 OS=Human immunodeficiency virus 1 OX=11676 GN=env PE=3 SV=1
2::L7WK70 **Mass:** 98620 **Score:** 58 **Matches:** 1(1) **Sequences:** 1(1)
>tr|L7WK70|L7WK70_9HIV1 Envelope glycoprotein gp160 OS=Human immunodeficiency virus 1 OX=11676 GN=env PE=3 SV=1
2::L7WK74 **Mass:** 98187 **Score:** 58 **Matches:** 1(1) **Sequences:** 1(1)
>tr|L7WK74|L7WK74_9HIV1 Envelope glycoprotein gp160 OS=Human immunodeficiency virus 1 OX=11676 GN=env PE=3 SV=1
2::L7WK79 **Mass:** 98468 **Score:** 58 **Matches:** 1(1) **Sequences:** 1(1)
>tr|L7WK79|L7WK79_9HIV1 Envelope glycoprotein gp160 OS=Human immunodeficiency virus 1 OX=11676 GN=env PE=3 SV=1
2::L7WK84 **Mass:** 99166 **Score:** 58 **Matches:** 1(1) **Sequences:** 1(1)
>tr|L7WK84|L7WK84_9HIV1 Envelope glycoprotein gp160 OS=Human immunodeficiency virus 1 OX=11676 GN=env PE=3 SV=1
2::L7WK88 **Mass:** 98287 **Score:** 58 **Matches:** 1(1) **Sequences:** 1(1)
>tr|L7WK88|L7WK88_9HIV1 Envelope glycoprotein gp160 OS=Human immunodeficiency virus 1 OX=11676 GN=env PE=3 SV=1
2::L7WKE3 **Mass:** 97184 **Score:** 58 **Matches:** 1(1) **Sequences:** 1(1)
>tr|L7WKE3|L7WKE3_9HIV1 Envelope glycoprotein gp160 OS=Human immunodeficiency virus 1 OX=11676 GN=env PE=3 SV=1
2::L7WKE9 **Mass:** 97011 **Score:** 58 **Matches:** 1(1) **Sequences:** 1(1)
>tr|L7WKE9|L7WKE9_9HIV1 Envelope glycoprotein gp160 OS=Human immunodeficiency virus 1 OX=11676 GN=env PE=3 SV=1
2::L7WKF4 **Mass:** 97038 **Score:** 58 **Matches:** 1(1) **Sequences:** 1(1)
>tr|L7WKF4|L7WKF4_9HIV1 Envelope glycoprotein gp160 OS=Human immunodeficiency virus 1 OX=11676 GN=env PE=3 SV=1
2::L7WKF9 **Mass:** 97108 **Score:** 58 **Matches:** 1(1) **Sequences:** 1(1)
>tr|L7WKF9|L7WKF9_9HIV1 Envelope glycoprotein gp160 OS=Human immunodeficiency virus 1 OX=11676 GN=env PE=3 SV=1
2::L7WKG5 **Mass:** 97016 **Score:** 58 **Matches:** 1(1) **Sequences:** 1(1)
>tr|L7WKG5|L7WKG5_9HIV1 Envelope glycoprotein gp160 OS=Human immunodeficiency virus 1 OX=11676 GN=env PE=3 SV=1
2::L7WKN3 **Mass:** 98213 **Score:** 58 **Matches:** 1(1) **Sequences:** 1(1)
>tr|L7WKN3|L7WKN3_9HIV1 Envelope glycoprotein gp160 OS=Human immunodeficiency virus 1 OX=11676 GN=env PE=3 SV=1
2::L7WKN7 **Mass:** 98953 **Score:** 58 **Matches:** 1(1) **Sequences:** 1(1)
>tr|L7WKN7|L7WKN7_9HIV1 Envelope glycoprotein gp160 OS=Human immunodeficiency virus 1 OX=11676 GN=env PE=3 SV=1
2::L7WKP1 **Mass:** 98938 **Score:** 58 **Matches:** 1(1) **Sequences:** 1(1)
>tr|L7WKP1|L7WKP1_9HIV1 Envelope glycoprotein gp160 OS=Human immunodeficiency virus 1 OX=11676 GN=env PE=3 SV=1
2::L7WKP6 **Mass:** 98858 **Score:** 58 **Matches:** 1(1) **Sequences:** 1(1)
>tr|L7WKP6|L7WKP6_9HIV1 Envelope glycoprotein gp160 OS=Human immunodeficiency virus 1 OX=11676 GN=env PE=3 SV=1
2::L7WN45 **Mass:** 99054 **Score:** 58 **Matches:** 1(1) **Sequences:** 1(1)
>tr|L7WN45|L7WN45_9HIV1 Envelope glycoprotein gp160 OS=Human immunodeficiency virus 1 OX=11676 GN=env PE=3 SV=1
2::L7WN50 **Mass:** 99166 **Score:** 58 **Matches:** 1(1) **Sequences:** 1(1)
>tr|L7WN50|L7WN50_9HIV1 Envelope glycoprotein gp160 OS=Human immunodeficiency virus 1 OX=11676 GN=env PE=3 SV=1
2::L7WN56 **Mass:** 98426 **Score:** 58 **Matches:** 1(1) **Sequences:** 1(1)
>tr|L7WN56|L7WN56_9HIV1 Envelope glycoprotein gp160 OS=Human immunodeficiency virus 1 OX=11676 GN=env PE=3 SV=1
2::L7WN60 **Mass:** 98795 **Score:** 58 **Matches:** 1(1) **Sequences:** 1(1)
>tr|L7WN60|L7WN60_9HIV1 Envelope glycoprotein gp160 OS=Human immunodeficiency virus 1 OX=11676 GN=env PE=3 SV=1
2::L7WN68 **Mass:** 98838 **Score:** 58 **Matches:** 1(1) **Sequences:** 1(1)
>tr|L7WN68|L7WN68_9HIV1 Envelope glycoprotein gp160 OS=Human immunodeficiency virus 1 OX=11676 GN=env PE=3 SV=1
2::Q7ZAP9 **Mass:** 97017 **Score:** 58 **Matches:** 1(1) **Sequences:** 1(1)
>tr|Q7ZAP9|Q7ZAP9_9HIV1 Envelope glycoprotein gp160 OS=Human immunodeficiency virus 1 OX=11676 GN=env PE=3 SV=1
2::A8VEH8 **Mass:** 96768 **Score:** 58 **Matches:** 1(1) **Sequences:** 1(1)
>tr|A8VEH8|A8VEH8_9HIV1 Envelope glycoprotein gp160 OS=Human immunodeficiency virus 1 OX=11676 GN=env PE=3 SV=1
2::F8S9V3 **Mass:** 98594 **Score:** 58 **Matches:** 1(1) **Sequences:** 1(1)
>tr|F8S9V3|F8S9V3_9HIV1 Envelope glycoprotein gp160 OS=Human immunodeficiency virus 1 OX=11676 GN=env PE=3 SV=1
2::F8S9X0 **Mass:** 96575 **Score:** 58 **Matches:** 1(1) **Sequences:** 1(1)
>tr|F8S9X0|F8S9X0_9HIV1 Envelope glycoprotein gp160 OS=Human immunodeficiency virus 1 OX=11676 GN=env PE=3 SV=1
2::F8S9X6 **Mass:** 92967 **Score:** 58 **Matches:** 1(1) **Sequences:** 1(1)
>tr|F8S9X6|F8S9X6_9HIV1 Envelope glycoprotein gp160 OS=Human immunodeficiency virus 1 OX=11676 GN=env PE=3 SV=1
2::F8SX53 **Mass:** 98039 **Score:** 58 **Matches:** 1(1) **Sequences:** 1(1)
>tr|F8SX53|F8SX53_9HIV1 Envelope glycoprotein gp160 (Fragment) OS=Human immunodeficiency virus 1 OX=11676 GN=env PE=3 SV=1
2::F8SX54 **Mass:** 97919 **Score:** 58 **Matches:** 1(1) **Sequences:** 1(1)
>tr|F8SX54|F8SX54_9HIV1 Envelope glycoprotein gp160 (Fragment) OS=Human immunodeficiency virus 1 OX=11676 GN=env PE=3 SV=1
2::F8SX55 **Mass:** 97974 **Score:** 58 **Matches:** 1(1) **Sequences:** 1(1)
>tr|F8SX55|F8SX55_9HIV1 Envelope glycoprotein gp160 (Fragment) OS=Human immunodeficiency virus 1 OX=11676 GN=env PE=3 SV=1
2::F8SX56 **Mass:** 96303 **Score:** 58 **Matches:** 1(1) **Sequences:** 1(1)
>tr|F8SX56|F8SX56_9HIV1 Envelope glycoprotein gp160 (Fragment) OS=Human immunodeficiency virus 1 OX=11676 GN=env PE=3 SV=1
2::F8SX57 **Mass:** 98078 **Score:** 58 **Matches:** 1(1) **Sequences:** 1(1)
>tr|F8SX57|F8SX57_9HIV1 Envelope glycoprotein gp160 (Fragment) OS=Human immunodeficiency virus 1 OX=11676 GN=env PE=3 SV=1
2::F8SX58 **Mass:** 97675 **Score:** 58 **Matches:** 1(1) **Sequences:** 1(1)
>tr|F8SX58|F8SX58_9HIV1 Envelope glycoprotein gp160 (Fragment) OS=Human immunodeficiency virus 1 OX=11676 GN=env PE=3 SV=1
2::F8SX59 **Mass:** 98054 **Score:** 58 **Matches:** 1(1) **Sequences:** 1(1)
>tr|F8SX59|F8SX59_9HIV1 Envelope glycoprotein gp160 OS=Human immunodeficiency virus 1 OX=11676 GN=env PE=3 SV=1
2::F8SX60 **Mass:** 98144 **Score:** 58 **Matches:** 1(1) **Sequences:** 1(1)
>tr|F8SX60|F8SX60_9HIV1 Envelope glycoprotein gp160 (Fragment) OS=Human immunodeficiency virus 1 OX=11676 GN=env PE=3 SV=1
2::F8SX61 **Mass:** 98185 **Score:** 58 **Matches:** 1(1) **Sequences:** 1(1)
>tr|F8SX61|F8SX61_9HIV1 Envelope glycoprotein gp160 (Fragment) OS=Human immunodeficiency virus 1 OX=11676 GN=env PE=3 SV=1
2::F8SX62 **Mass:** 97976 **Score:** 58 **Matches:** 1(1) **Sequences:** 1(1)
>tr|F8SX62|F8SX62_9HIV1 Envelope glycoprotein gp160 (Fragment) OS=Human immunodeficiency virus 1 OX=11676 GN=env PE=3 SV=1

2::F8SX63 **Mass:** 97946 **Score:** 58 **Matches:** 1(1) **Sequences:** 1(1)
>tr|F8SX63|F8SX63_9HIV1 Envelope glycoprotein gp160 (Fragment) OS=Human immunodeficiency virus 1 OX=11676 GN=env PE=3 SV=1
2::Q08JX3 **Mass:** 99172 **Score:** 58 **Matches:** 1(1) **Sequences:** 1(1)
>tr|Q08JX3|Q08JX3_9HIV1 Envelope glycoprotein gp160 OS=Human immunodeficiency virus 1 OX=11676 GN=env PE=3 SV=1
2::Q08JY2 **Mass:** 99358 **Score:** 58 **Matches:** 1(1) **Sequences:** 1(1)
>tr|Q08JY2|Q08JY2_9HIV1 Envelope glycoprotein gp160 OS=Human immunodeficiency virus 1 OX=11676 GN=env PE=3 SV=1
2::Q08K36 **Mass:** 100346 **Score:** 58 **Matches:** 1(1) **Sequences:** 1(1)
>tr|Q08K36|Q08K36_9HIV1 Envelope glycoprotein gp160 OS=Human immunodeficiency virus 1 OX=11676 GN=env PE=3 SV=1
2::Q8UTT0 **Mass:** 99277 **Score:** 58 **Matches:** 1(1) **Sequences:** 1(1)
>tr|Q8UTT0|Q8UTT0_9HIV1 Envelope glycoprotein gp160 OS=Human immunodeficiency virus 1 OX=11676 GN=env PE=3 SV=1
2::E9JST7 **Mass:** 98176 **Score:** 58 **Matches:** 1(1) **Sequences:** 1(1)
>tr|E9JST7|E9JST7_9HIV1 Envelope glycoprotein gp160 OS=Human immunodeficiency virus 1 OX=11676 GN=env PE=3 SV=1
2::E9JTE1 **Mass:** 99775 **Score:** 58 **Matches:** 1(1) **Sequences:** 1(1)
>tr|E9JTE1|E9JTE1_9HIV1 Envelope glycoprotein gp160 OS=Human immunodeficiency virus 1 OX=11676 GN=env PE=3 SV=1
2::E9JTE2 **Mass:** 99949 **Score:** 58 **Matches:** 1(1) **Sequences:** 1(1)
>tr|E9JTE2|E9JTE2_9HIV1 Envelope glycoprotein gp160 OS=Human immunodeficiency virus 1 OX=11676 GN=env PE=3 SV=1
2::E9JTE3 **Mass:** 100503 **Score:** 58 **Matches:** 1(1) **Sequences:** 1(1)
>tr|E9JTE3|E9JTE3_9HIV1 Envelope glycoprotein gp160 OS=Human immunodeficiency virus 1 OX=11676 GN=env PE=3 SV=1
2::E9JTE4 **Mass:** 101443 **Score:** 58 **Matches:** 1(1) **Sequences:** 1(1)
>tr|E9JTE4|E9JTE4_9HIV1 Envelope glycoprotein gp160 OS=Human immunodeficiency virus 1 OX=11676 GN=env PE=3 SV=1
2::E9JTE6 **Mass:** 100906 **Score:** 58 **Matches:** 1(1) **Sequences:** 1(1)
>tr|E9JTE6|E9JTE6_9HIV1 Envelope glycoprotein gp160 OS=Human immunodeficiency virus 1 OX=11676 GN=env PE=3 SV=1
2::E9JTE7 **Mass:** 99668 **Score:** 58 **Matches:** 1(1) **Sequences:** 1(1)
>tr|E9JTE7|E9JTE7_9HIV1 Envelope glycoprotein gp160 OS=Human immunodeficiency virus 1 OX=11676 GN=env PE=3 SV=1
2::E9JTE8 **Mass:** 99546 **Score:** 58 **Matches:** 1(1) **Sequences:** 1(1)
>tr|E9JTE8|E9JTE8_9HIV1 Envelope glycoprotein gp160 OS=Human immunodeficiency virus 1 OX=11676 GN=env PE=3 SV=1
2::E9JTS5 **Mass:** 96335 **Score:** 58 **Matches:** 1(1) **Sequences:** 1(1)
>tr|E9JTS5|E9JTS5_9HIV1 Envelope glycoprotein gp160 OS=Human immunodeficiency virus 1 OX=11676 GN=env PE=3 SV=1
2::E9JTS6 **Mass:** 97749 **Score:** 58 **Matches:** 1(1) **Sequences:** 1(1)
>tr|E9JTS6|E9JTS6_9HIV1 Envelope glycoprotein gp160 OS=Human immunodeficiency virus 1 OX=11676 GN=env PE=3 SV=1
2::E9JTT0 **Mass:** 97556 **Score:** 58 **Matches:** 1(1) **Sequences:** 1(1)
>tr|E9JTT0|E9JTT0_9HIV1 Envelope glycoprotein gp160 OS=Human immunodeficiency virus 1 OX=11676 GN=env PE=3 SV=1
2::E9JTT1 **Mass:** 97763 **Score:** 58 **Matches:** 1(1) **Sequences:** 1(1)
>tr|E9JTT1|E9JTT1_9HIV1 Envelope glycoprotein gp160 OS=Human immunodeficiency virus 1 OX=11676 GN=env PE=3 SV=1
2::G9JVH3 **Mass:** 97855 **Score:** 58 **Matches:** 1(1) **Sequences:** 1(1)
>tr|G9JVH3|G9JVH3_9HIV1 Envelope glycoprotein gp160 OS=Human immunodeficiency virus 1 OX=11676 GN=env PE=3 SV=1
2::H9TM84 **Mass:** 96633 **Score:** 58 **Matches:** 1(1) **Sequences:** 1(1)
>tr|H9TM84|H9TM84_9HIV1 Envelope glycoprotein gp160 OS=Human immunodeficiency virus 1 OX=11676 GN=env PE=3 SV=1
2::H9TM88 **Mass:** 96528 **Score:** 58 **Matches:** 1(1) **Sequences:** 1(1)
>tr|H9TM88|H9TM88_9HIV1 Envelope glycoprotein gp160 OS=Human immunodeficiency virus 1 OX=11676 GN=env PE=3 SV=1
2::H9TM92 **Mass:** 100212 **Score:** 58 **Matches:** 1(1) **Sequences:** 1(1)
>tr|H9TM92|H9TM92_9HIV1 Envelope glycoprotein gp160 OS=Human immunodeficiency virus 1 OX=11676 GN=env PE=3 SV=1
2::H9TM96 **Mass:** 96459 **Score:** 58 **Matches:** 1(1) **Sequences:** 1(1)
>tr|H9TM96|H9TM96_9HIV1 Envelope glycoprotein gp160 OS=Human immunodeficiency virus 1 OX=11676 GN=env PE=3 SV=1
2::H9TMA0 **Mass:** 100068 **Score:** 58 **Matches:** 1(1) **Sequences:** 1(1)
>tr|H9TMA0|H9TMA0_9HIV1 Envelope glycoprotein gp160 OS=Human immunodeficiency virus 1 OX=11676 GN=env PE=3 SV=1
2::H9TMA4 **Mass:** 100981 **Score:** 58 **Matches:** 1(1) **Sequences:** 1(1)
>tr|H9TMA4|H9TMA4_9HIV1 Envelope glycoprotein gp160 OS=Human immunodeficiency virus 1 OX=11676 GN=env PE=3 SV=1
2::H9TMA8 **Mass:** 100092 **Score:** 58 **Matches:** 1(1) **Sequences:** 1(1)
>tr|H9TMA8|H9TMA8_9HIV1 Envelope glycoprotein gp160 OS=Human immunodeficiency virus 1 OX=11676 GN=env PE=3 SV=1
2::H9TMB2 **Mass:** 100244 **Score:** 58 **Matches:** 1(1) **Sequences:** 1(1)
>tr|H9TMB2|H9TMB2_9HIV1 Envelope glycoprotein gp160 OS=Human immunodeficiency virus 1 OX=11676 GN=env PE=3 SV=1
2::H9TMB6 **Mass:** 96760 **Score:** 58 **Matches:** 1(1) **Sequences:** 1(1)
>tr|H9TMB6|H9TMB6_9HIV1 Envelope glycoprotein gp160 OS=Human immunodeficiency virus 1 OX=11676 GN=env PE=3 SV=1
2::H9TMC3 **Mass:** 100095 **Score:** 58 **Matches:** 1(1) **Sequences:** 1(1)
>tr|H9TMC3|H9TMC3_9HIV1 Envelope glycoprotein gp160 OS=Human immunodeficiency virus 1 OX=11676 GN=env PE=3 SV=1
2::H9TMC7 **Mass:** 100095 **Score:** 58 **Matches:** 1(1) **Sequences:** 1(1)
>tr|H9TMC7|H9TMC7_9HIV1 Envelope glycoprotein gp160 OS=Human immunodeficiency virus 1 OX=11676 GN=env PE=3 SV=1
2::H9TMD1 **Mass:** 100902 **Score:** 58 **Matches:** 1(1) **Sequences:** 1(1)
>tr|H9TMD1|H9TMD1_9HIV1 Envelope glycoprotein gp160 OS=Human immunodeficiency virus 1 OX=11676 GN=env PE=3 SV=1
2::H9TMD5 **Mass:** 96660 **Score:** 58 **Matches:** 1(1) **Sequences:** 1(1)
>tr|H9TMD5|H9TMD5_9HIV1 Envelope glycoprotein gp160 OS=Human immunodeficiency virus 1 OX=11676 GN=env PE=3 SV=1
2::H9TMD9 **Mass:** 100233 **Score:** 58 **Matches:** 1(1) **Sequences:** 1(1)
>tr|H9TMD9|H9TMD9_9HIV1 Envelope glycoprotein gp160 OS=Human immunodeficiency virus 1 OX=11676 GN=env PE=3 SV=1
2::H9TME3 **Mass:** 100053 **Score:** 58 **Matches:** 1(1) **Sequences:** 1(1)
>tr|H9TME3|H9TME3_9HIV1 Envelope glycoprotein gp160 OS=Human immunodeficiency virus 1 OX=11676 GN=env PE=3 SV=1
2::H9TMF0 **Mass:** 96647 **Score:** 58 **Matches:** 1(1) **Sequences:** 1(1)
>tr|H9TMF0|H9TMF0_9HIV1 Envelope glycoprotein gp160 OS=Human immunodeficiency virus 1 OX=11676 GN=env PE=3 SV=1
2::H9TMF4 **Mass:** 96518 **Score:** 58 **Matches:** 1(1) **Sequences:** 1(1)
>tr|H9TMF4|H9TMF4_9HIV1 Envelope glycoprotein gp160 OS=Human immunodeficiency virus 1 OX=11676 GN=env PE=3 SV=1
2::H9TMF8 **Mass:** 96697 **Score:** 58 **Matches:** 1(1) **Sequences:** 1(1)
>tr|H9TMF8|H9TMF8_9HIV1 Envelope glycoprotein gp160 OS=Human immunodeficiency virus 1 OX=11676 GN=env PE=3 SV=1
2::H9TMG2 **Mass:** 96453 **Score:** 58 **Matches:** 1(1) **Sequences:** 1(1)
>tr|H9TMG2|H9TMG2_9HIV1 Envelope glycoprotein gp160 OS=Human immunodeficiency virus 1 OX=11676 GN=env PE=3 SV=1
2::H9TMG6 **Mass:** 96497 **Score:** 58 **Matches:** 1(1) **Sequences:** 1(1)
>tr|H9TMG6|H9TMG6_9HIV1 Envelope glycoprotein gp160 OS=Human immunodeficiency virus 1 OX=11676 GN=env PE=3 SV=1
2::H9TMH0 **Mass:** 100132 **Score:** 58 **Matches:** 1(1) **Sequences:** 1(1)
>tr|H9TMH0|H9TMH0_9HIV1 Envelope glycoprotein gp160 OS=Human immunodeficiency virus 1 OX=11676 GN=env PE=3 SV=1
2::H9TMH4 **Mass:** 96545 **Score:** 58 **Matches:** 1(1) **Sequences:** 1(1)
>tr|H9TMH4|H9TMH4_9HIV1 Envelope glycoprotein gp160 OS=Human immunodeficiency virus 1 OX=11676 GN=env PE=3 SV=1
2::H9TMH8 **Mass:** 96393 **Score:** 58 **Matches:** 1(1) **Sequences:** 1(1)
>tr|H9TMH8|H9TMH8_9HIV1 Envelope glycoprotein gp160 OS=Human immunodeficiency virus 1 OX=11676 GN=env PE=3 SV=1
2::H9TMI2 **Mass:** 96375 **Score:** 58 **Matches:** 1(1) **Sequences:** 1(1)
>tr|H9TMI2|H9TMI2_9HIV1 Envelope glycoprotein gp160 OS=Human immunodeficiency virus 1 OX=11676 GN=env PE=3 SV=1
2::H9TMU3 **Mass:** 96013 **Score:** 58 **Matches:** 1(1) **Sequences:** 1(1)
>tr|H9TMU3|H9TMU3_9HIV1 Envelope glycoprotein gp160 OS=Human immunodeficiency virus 1 OX=11676 GN=env PE=3 SV=1
2::H9TMU6 **Mass:** 98090 **Score:** 58 **Matches:** 1(1) **Sequences:** 1(1)
>tr|H9TMU6|H9TMU6_9HIV1 Envelope glycoprotein gp160 OS=Human immunodeficiency virus 1 OX=11676 GN=env PE=3 SV=1
2::H9TMU9 **Mass:** 97983 **Score:** 58 **Matches:** 1(1) **Sequences:** 1(1)
>tr|H9TMU9|H9TMU9_9HIV1 Envelope glycoprotein gp160 OS=Human immunodeficiency virus 1 OX=11676 GN=env PE=3 SV=1
2::H9TMV5 **Mass:** 97870 **Score:** 58 **Matches:** 1(1) **Sequences:** 1(1)
>tr|H9TMV5|H9TMV5_9HIV1 Envelope glycoprotein gp160 (Fragment) OS=Human immunodeficiency virus 1 OX=11676 GN=env PE=3 SV=1
2::H9TMW3 **Mass:** 98037 **Score:** 58 **Matches:** 1(1) **Sequences:** 1(1)
>tr|H9TMW3|H9TMW3_9HIV1 Envelope glycoprotein gp160 OS=Human immunodeficiency virus 1 OX=11676 GN=env PE=3 SV=1
2::H9TMX3 **Mass:** 97350 **Score:** 58 **Matches:** 1(1) **Sequences:** 1(1)
>tr|H9TMX3|H9TMX3_9HIV1 Envelope glycoprotein gp160 OS=Human immunodeficiency virus 1 OX=11676 GN=env PE=3 SV=1
2::H9TMX6 **Mass:** 97983 **Score:** 58 **Matches:** 1(1) **Sequences:** 1(1)
>tr|H9TMX6|H9TMX6_9HIV1 Envelope glycoprotein gp160 OS=Human immunodeficiency virus 1 OX=11676 GN=env PE=3 SV=1
2::H9TMX9 **Mass:** 97588 **Score:** 58 **Matches:** 1(1) **Sequences:** 1(1)
>tr|H9TMX9|H9TMX9_9HIV1 Envelope glycoprotein gp160 OS=Human immunodeficiency virus 1 OX=11676 GN=env PE=3 SV=1
2::H9TMY2 **Mass:** 98123 **Score:** 58 **Matches:** 1(1) **Sequences:** 1(1)
>tr|H9TMY2|H9TMY2_9HIV1 Envelope glycoprotein gp160 OS=Human immunodeficiency virus 1 OX=11676 GN=env PE=3 SV=1
2::H9TMY5 **Mass:** 97547 **Score:** 58 **Matches:** 1(1) **Sequences:** 1(1)
>tr|H9TMY5|H9TMY5_9HIV1 Envelope glycoprotein gp160 OS=Human immunodeficiency virus 1 OX=11676 GN=env PE=3 SV=1
2::H9TMY8 **Mass:** 97538 **Score:** 58 **Matches:** 1(1) **Sequences:** 1(1)
>tr|H9TMY8|H9TMY8_9HIV1 Envelope glycoprotein gp160 OS=Human immunodeficiency virus 1 OX=11676 GN=env PE=3 SV=1
2::H9TMZ3 **Mass:** 97878 **Score:** 58 **Matches:** 1(1) **Sequences:** 1(1)
>tr|H9TMZ3|H9TMZ3_9HIV1 Envelope glycoprotein gp160 OS=Human immunodeficiency virus 1 OX=11676 GN=env PE=3 SV=1
2::H9TMZ8 **Mass:** 97507 **Score:** 58 **Matches:** 1(1) **Sequences:** 1(1)
>tr|H9TMZ8|H9TMZ8_9HIV1 Envelope glycoprotein gp160 OS=Human immunodeficiency virus 1 OX=11676 GN=env PE=3 SV=1

2::H9TN01 **Mass:** 97445 **Score:** 58 **Matches:** 1(1) **Sequences:** 1(1)
>tr|H9TN01|H9TN01_9HIV1 Envelope glycoprotein gp160 (Fragment) OS=Human immunodeficiency virus 1 OX=11676 GN=env PE=3 SV=1
2::H9TN04 **Mass:** 98051 **Score:** 58 **Matches:** 1(1) **Sequences:** 1(1)
>tr|H9TN04|H9TN04_9HIV1 Envelope glycoprotein gp160 OS=Human immunodeficiency virus 1 OX=11676 GN=env PE=3 SV=1
2::H9TN07 **Mass:** 97551 **Score:** 58 **Matches:** 1(1) **Sequences:** 1(1)
>tr|H9TN07|H9TN07_9HIV1 Envelope glycoprotein gp160 OS=Human immunodeficiency virus 1 OX=11676 GN=env PE=3 SV=1
2::H9TN10 **Mass:** 97889 **Score:** 58 **Matches:** 1(1) **Sequences:** 1(1)
>tr|H9TN10|H9TN10_9HIV1 Envelope glycoprotein gp160 OS=Human immunodeficiency virus 1 OX=11676 GN=env PE=3 SV=1
2::H9TN13 **Mass:** 97962 **Score:** 58 **Matches:** 1(1) **Sequences:** 1(1)
>tr|H9TN13|H9TN13_9HIV1 Envelope glycoprotein gp160 OS=Human immunodeficiency virus 1 OX=11676 GN=env PE=3 SV=1
2::H9TN16 **Mass:** 97621 **Score:** 58 **Matches:** 1(1) **Sequences:** 1(1)
>tr|H9TN16|H9TN16_9HIV1 Envelope glycoprotein gp160 OS=Human immunodeficiency virus 1 OX=11676 GN=env PE=3 SV=1
2::H9TN19 **Mass:** 97939 **Score:** 58 **Matches:** 1(1) **Sequences:** 1(1)
>tr|H9TN19|H9TN19_9HIV1 Envelope glycoprotein gp160 OS=Human immunodeficiency virus 1 OX=11676 GN=env PE=3 SV=1
2::H9TN22 **Mass:** 98033 **Score:** 58 **Matches:** 1(1) **Sequences:** 1(1)
>tr|H9TN22|H9TN22_9HIV1 Envelope glycoprotein gp160 OS=Human immunodeficiency virus 1 OX=11676 GN=env PE=3 SV=1
2::H9TN25 **Mass:** 98003 **Score:** 58 **Matches:** 1(1) **Sequences:** 1(1)
>tr|H9TN25|H9TN25_9HIV1 Envelope glycoprotein gp160 OS=Human immunodeficiency virus 1 OX=11676 GN=env PE=3 SV=1
2::K9MUE6 **Mass:** 98076 **Score:** 58 **Matches:** 1(1) **Sequences:** 1(1)
>tr|K9MUE6|K9MUE6_9HIV1 Envelope glycoprotein gp160 OS=Human immunodeficiency virus 1 OX=11676 GN=env PE=3 SV=1
2::K9MUF0 **Mass:** 97798 **Score:** 58 **Matches:** 1(1) **Sequences:** 1(1)
>tr|K9MUF0|K9MUF0_9HIV1 Envelope glycoprotein gp160 OS=Human immunodeficiency virus 1 OX=11676 GN=env PE=3 SV=1
2::K9MUN8 **Mass:** 97457 **Score:** 58 **Matches:** 1(1) **Sequences:** 1(1)
>tr|K9MUN8|K9MUN8_9HIV1 Envelope glycoprotein gp160 OS=Human immunodeficiency virus 1 OX=11676 GN=env PE=3 SV=1
2::K9MVH4 **Mass:** 97668 **Score:** 58 **Matches:** 1(1) **Sequences:** 1(1)
>tr|K9MVH4|K9MVH4_9HIV1 Envelope glycoprotein gp160 OS=Human immunodeficiency virus 1 OX=11676 GN=env PE=3 SV=1
2::K9MVH5 **Mass:** 97640 **Score:** 58 **Matches:** 1(1) **Sequences:** 1(1)
>tr|K9MVH5|K9MVH5_9HIV1 Envelope glycoprotein gp160 OS=Human immunodeficiency virus 1 OX=11676 GN=env PE=3 SV=1
2::M9QTE0 **Mass:** 98351 **Score:** 58 **Matches:** 1(1) **Sequences:** 1(1)
>tr|M9QTE0|M9QTE0_9HIV1 Envelope glycoprotein gp160 OS=Human immunodeficiency virus 1 OX=11676 GN=env PE=3 SV=1
2::Q9YKU1 **Mass:** 100610 **Score:** 58 **Matches:** 1(1) **Sequences:** 1(1)
>tr|Q9YKU1|Q9YKU1_9HIV1 Envelope glycoprotein gp160 OS=Human immunodeficiency virus 1 OX=11676 GN=env PE=3 SV=1
2::Q9YKU7 **Mass:** 98062 **Score:** 58 **Matches:** 1(1) **Sequences:** 1(1)
>tr|Q9YKU7|Q9YKU7_9HIV1 Envelope glycoprotein gp160 OS=Human immunodeficiency virus 1 OX=11676 GN=env PE=3 SV=1
2::Q9YKV1 **Mass:** 98432 **Score:** 58 **Matches:** 1(1) **Sequences:** 1(1)
>tr|Q9YKV1|Q9YKV1_9HIV1 Envelope glycoprotein gp160 OS=Human immunodeficiency virus 1 OX=11676 GN=env PE=3 SV=1
2::Q9YKV7 **Mass:** 98046 **Score:** 58 **Matches:** 1(1) **Sequences:** 1(1)
>tr|Q9YKV7|Q9YKV7_9HIV1 Envelope glycoprotein gp160 OS=Human immunodeficiency virus 1 OX=11676 GN=env PE=3 SV=1
2::Q30BW6 **Mass:** 99144 **Score:** 58 **Matches:** 1(1) **Sequences:** 1(1)
>tr|Q30BW6|Q30BW6_9HIV1 Envelope glycoprotein gp160 OS=Human immunodeficiency virus 1 OX=11676 GN=env PE=3 SV=1
2::Q90CV3 **Mass:** 98680 **Score:** 58 **Matches:** 1(1) **Sequences:** 1(1)
>tr|Q90CV3|Q90CV3_9HIV1 Envelope glycoprotein gp160 OS=Human immunodeficiency virus 1 OX=11676 GN=env PE=3 SV=1
2::Q202J5 **Mass:** 98437 **Score:** 58 **Matches:** 1(1) **Sequences:** 1(1)
>tr|Q202J5|Q202J5_9HIV1 Envelope glycoprotein gp160 OS=Human immunodeficiency virus 1 OX=11676 GN=env PE=3 SV=1
2::Q901X2 **Mass:** 98425 **Score:** 58 **Matches:** 1(1) **Sequences:** 1(1)
>tr|Q901X2|Q901X2_9HIV1 Envelope glycoprotein gp160 OS=Human immunodeficiency virus 1 OX=11676 GN=env PE=3 SV=1
2::Q901Z0 **Mass:** 96957 **Score:** 58 **Matches:** 1(1) **Sequences:** 1(1)
>tr|Q901Z0|Q901Z0_9HIV1 Envelope glycoprotein gp160 OS=Human immunodeficiency virus 1 OX=11676 GN=env PE=3 SV=1
2::Q73358 **Mass:** 97200 **Score:** 58 **Matches:** 1(1) **Sequences:** 1(1)
>tr|Q73358|Q73358_9HIV1 Envelope glycoprotein gp160 OS=Human immunodeficiency virus 1 OX=11676 GN=env PE=3 SV=1
2::Q73359 **Mass:** 96690 **Score:** 58 **Matches:** 1(1) **Sequences:** 1(1)
>tr|Q73359|Q73359_9HIV1 Envelope glycoprotein gp160 OS=Human immunodeficiency virus 1 OX=11676 GN=env PE=3 SV=1

---

30. 2::A0A096XT44 **Mass:** 11896 **Score:** 55 **Matches:** 2(2) **Sequences:** 1(1) **emPAI:** 0.41
>tr|A0A096XT44|A0A096XT44_9CAUD Uncharacterized protein OS=Enterococcus phage ECP3 OX=1498168 PE=4 SV=1

Query	Observed	Mr(expt)	Mr(calc)	ppm	Miss	Score	Expect	Rank	Unique	Peptide
120	397.2258	792.4371	792.4381	-1.24	0	51	0.021	1	U	K.LYELQK.S 119

Proteins matching the same set of peptides:
2::A0A097C039 **Mass:** 266621 **Score:** 55 **Matches:** 2(2) **Sequences:** 1(1)
>tr|A0A097C039|A0A097C039_9VIRU RNA-dependent RNA polymerase OS=Gamboa virus OX=35313 PE=4 SV=1
2::A0A097C040 **Mass:** 266625 **Score:** 55 **Matches:** 2(2) **Sequences:** 1(1)
>tr|A0A097C040|A0A097C040_9VIRU RNA-dependent RNA polymerase OS=Gamboa virus OX=35313 PE=4 SV=1
2::A0A097C041 **Mass:** 266640 **Score:** 55 **Matches:** 2(2) **Sequences:** 1(1)
>tr|A0A097C041|A0A097C041_9VIRU RNA-dependent RNA polymerase OS=Gamboa virus OX=35313 PE=4 SV=1
2::A0A097C042 **Mass:** 266593 **Score:** 55 **Matches:** 2(2) **Sequences:** 1(1)
>tr|A0A097C042|A0A097C042_9VIRU RNA-dependent RNA polymerase OS=Alajuela virus OX=1552846 PE=4 SV=1
2::A0A097C045 **Mass:** 263960 **Score:** 55 **Matches:** 2(2) **Sequences:** 1(1)
>tr|A0A097C045|A0A097C045_9VIRU RNA-dependent RNA polymerase (Fragment) OS=Calchaqui virus OX=1552845 PE=4 SV=1
2::A0A109W3L6 **Mass:** 266619 **Score:** 55 **Matches:** 2(2) **Sequences:** 1(1)
>tr|A0A109W3L6|A0A109W3L6_9VIRU RNA-dependent RNA polymerase OS=Gamboa virus OX=35313 PE=4 SV=1
2::A0A120KLG7 **Mass:** 266666 **Score:** 55 **Matches:** 2(2) **Sequences:** 1(1)
>tr|A0A120KLG7|A0A120KLG7_9VIRU RNA-dependent RNA polymerase OS=Gamboa virus OX=35313 PE=4 SV=1
2::A0A125RZ39 **Mass:** 266573 **Score:** 55 **Matches:** 2(2) **Sequences:** 1(1)
>tr|A0A125RZ39|A0A125RZ39_9VIRU RNA-dependent RNA polymerase OS=Gamboa virus OX=35313 PE=4 SV=1
2::A0A125RZ40 **Mass:** 266607 **Score:** 55 **Matches:** 2(2) **Sequences:** 1(1)
>tr|A0A125RZ40|A0A125RZ40_9VIRU RNA-dependent RNA polymerase OS=Gamboa virus OX=35313 PE=4 SV=1
2::A0A2K8HPR5 **Mass:** 266710 **Score:** 55 **Matches:** 2(2) **Sequences:** 1(1)
>tr|A0A2K8HPR5|A0A2K8HPR5_9VIRU RNA-dependent RNA polymerase OS=Gamboa virus OX=35313 PE=4 SV=1
2::A0A2K8HQL7 **Mass:** 266464 **Score:** 55 **Matches:** 2(2) **Sequences:** 1(1)
>tr|A0A2K8HQL7|A0A2K8HQL7_9VIRU RNA-dependent RNA polymerase OS=Gamboa virus OX=35313 PE=4 SV=1
2::A0A2K8HTC8 **Mass:** 266548 **Score:** 55 **Matches:** 2(2) **Sequences:** 1(1)
>tr|A0A2K8HTC8|A0A2K8HTC8_9VIRU RNA-dependent RNA polymerase OS=Gamboa virus OX=35313 PE=4 SV=1
2::A0A2K8HZ72 **Mass:** 266524 **Score:** 55 **Matches:** 2(2) **Sequences:** 1(1)
>tr|A0A2K8HZ72|A0A2K8HZ72_9VIRU RNA-dependent RNA polymerase OS=Gamboa virus OX=35313 PE=4 SV=1
2::A0A2K8I2I5 **Mass:** 266755 **Score:** 55 **Matches:** 2(2) **Sequences:** 1(1)
>tr|A0A2K8I2I5|A0A2K8I2I5_9VIRU RNA-dependent RNA polymerase OS=Gamboa virus OX=35313 PE=4 SV=1
2::A0A2K8I602 **Mass:** 266627 **Score:** 55 **Matches:** 2(2) **Sequences:** 1(1)
>tr|A0A2K8I602|A0A2K8I602_9VIRU RNA-dependent RNA polymerase OS=Gamboa virus OX=35313 PE=4 SV=1
2::A0A2K8IBG0 **Mass:** 266652 **Score:** 55 **Matches:** 2(2) **Sequences:** 1(1)
>tr|A0A2K8IBG0|A0A2K8IBG0_9VIRU RNA-dependent RNA polymerase OS=Pueblo Viejo virus OX=2036955 PE=4 SV=1
2::A0A2L2FNS1 **Mass:** 266682 **Score:** 55 **Matches:** 2(2) **Sequences:** 1(1)
>tr|A0A2L2FNS1|A0A2L2FNS1_9VIRU RNA-dependent RNA polymerase OS=Gamboa virus OX=35313 PE=4 SV=1
2::A0A2Z6FZT6 **Mass:** 11896 **Score:** 55 **Matches:** 2(2) **Sequences:** 1(1)
>tr|A0A2Z6FZT6|A0A2Z6FZT6_9CAUD Uncharacterized protein OS=Enterococcus phage phiEF17H OX=2218497 GN=PHIEF17H_1980 PE=4 SV=1
2::A0A2Z6G0A8 **Mass:** 11896 **Score:** 55 **Matches:** 2(2) **Sequences:** 1(1)
>tr|A0A2Z6G0A8|A0A2Z6G0A8_9CAUD Uncharacterized protein OS=Enterococcus phage phiM1EF22 OX=2218498 GN=PHIM1EF22_1980 PE=4 SV=1
2::A0A3P5VTY4 **Mass:** 11896 **Score:** 55 **Matches:** 2(2) **Sequences:** 1(1)
>tr|A0A3P5VTY4|A0A3P5VTY4_9CAUD Scaffolding protein, putative previously named ORF B1 OS=Enterococcus phage 156 OX=2315797 GN=PHI156_201 PE=4 SV=1
2::A0A4D6DU27 **Mass:** 11896 **Score:** 55 **Matches:** 2(2) **Sequences:** 1(1)
>tr|A0A4D6DU27|A0A4D6DU27_9CAUD Prohead assembly (Scaffolding) protein OS=Enterococcus phage vB_EfaM_Ef2.1 OX=2546633 PE=4 SV=1
2::A0A4D6DV50 **Mass:** 11896 **Score:** 55 **Matches:** 2(2) **Sequences:** 1(1)
>tr|A0A4D6DV50|A0A4D6DV50_9CAUD Prohead assembly (Scaffolding) protein OS=Enterococcus phage vB_EfaM_Ef2.3 OX=2546634 PE=4 SV=1
2::A8E2R4 **Mass:** 11895 **Score:** 55 **Matches:** 2(2) **Sequences:** 1(1)
>tr|A8E2R4|A8E2R4_BPPHE Uncharacterized protein OS=Enterococcus phage phiEF24C OX=442493 GN=EFP_212 PE=4 SV=1

31. 2::A0A516LXB0 **Mass:** 188227 **Score:** 54 **Matches:** 2(2) **Sequences:** 1(1) **emPAI:** 0.02
>tr|A0A516LXB0|A0A516LXB0_9VIRU Uncharacterized protein OS=Prokaryotic dsDNA virus sp. OX=2591644 GN=Unbinned175contig1000_19 PE=4 SV=1

Query	Observed	Mr(expt)	Mr(calc)	ppm	Miss	Score	Expect	Rank	Unique	Peptide
404	464.2079	926.4013	926.4021	-0.88	0	41	0.013	1	U	K.FDPEEYK.G 405

---

32. 2::Q6RU22 **Mass:** 17943 **Score:** 53 **Matches:** 1(1) **Sequences:** 1(1) **emPAI:** 0.26
>tr|Q6RU22|Q6RU22_9HIV1 Gag protein (Fragment) OS=Human immunodeficiency virus 1 OX=11676 GN=gag PE=4 SV=1

Query	Observed	Mr(expt)	Mr(calc)	ppm	Miss	Score	Expect	Rank	Unique	Peptide
566	496.7640	991.5135	991.5127	0.79	0	53	0.0042	1	U	-.DFLNAWVK.V

---

33. 2::S4VSF6 **Mass:** 53177 **Score:** 52 **Matches:** 1(1) **Sequences:** 1(1) **emPAI:** 0.08
>tr|S4VSF6|S4VSF6_9VIRU Uncharacterized protein OS=Pandoravirus dulcis OX=1349409 GN=pdul_cds_330 PE=4 SV=1

Query	Observed	Mr(expt)	Mr(calc)	ppm	Miss	Score	Expect	Rank	Unique	Peptide
121	398.2398	794.4651	794.4650	0.10	0	52	0.0033	1	U	R.LIAPEPR.A

---

34. 2::O90019 **Mass:** 23170 **Score:** 52 **Matches:** 2(2) **Sequences:** 1(1) **emPAI:** 0.20
>tr|O90019|O90019_9HIV1 Envelope glycoprotein (Fragment) OS=Human immunodeficiency virus 1 OX=11676 GN=env PE=4 SV=1

Query	Observed	Mr(expt)	Mr(calc)	ppm	Miss	Score	Expect	Rank	Unique	Peptide
324	446.2506	890.4866	890.4862	0.48	0	48	0.021	1	U	-.EFEVVIR.S 323

Proteins matching the same set of peptides:
2::O90021 **Mass:** 23019 **Score:** 52 **Matches:** 2(2) **Sequences:** 1(1)
>tr|O90021|O90021_9HIV1 Envelope glycoprotein (Fragment) OS=Human immunodeficiency virus 1 OX=11676 GN=env PE=4 SV=1

---

35. 2::A0A0G2Y0A6 **Mass:** 107753 **Score:** 52 **Matches:** 1(1) **Sequences:** 1(1) **emPAI:** 0.04
>tr|A0A0G2Y0A6|A0A0G2Y0A6_MIMIV Heat shock protein 70-like protein OS=Acanthamoeba polyphaga mimivirus OX=212035 PE=4 SV=1

Query	Observed	Mr(expt)	Mr(calc)	ppm	Miss	Score	Expect	Rank	Unique	Peptide
348	451.2769	900.5393	900.5393	-0.02	0	52	0.006	1	U	K.VILVGGSTR.I

Proteins matching the same set of peptides:
2::A0A0G2Y835 **Mass:** 107638 **Score:** 52 **Matches:** 1(1) **Sequences:** 1(1)
>tr|A0A0G2Y835|A0A0G2Y835_MIMIV Heat shock protein 70-like protein OS=Acanthamoeba polyphaga mimivirus OX=212035 PE=4 SV=1
2::A0A0G2YCR2 **Mass:** 107765 **Score:** 52 **Matches:** 1(1) **Sequences:** 1(1)
>tr|A0A0G2YCR2|A0A0G2YCR2_MIMIV Heat shock 70Kd HSP OS=Acanthamoeba polyphaga mimivirus OX=212035 GN=L254 PE=4 SV=1
2::A0A0U2K379 **Mass:** 107765 **Score:** 52 **Matches:** 1(1) **Sequences:** 1(1)
>tr|A0A0U2K379|A0A0U2K379_9VIRU Heat shock 70Kd HSP OS=Niemeyer virus OX=1766765 PE=4 SV=1
2::A0A140E0K5 **Mass:** 107765 **Score:** 52 **Matches:** 1(1) **Sequences:** 1(1)
>tr|A0A140E0K5|A0A140E0K5_MIMIV Heat shock protein 70-like protein OS=Samba virus OX=1461100 PE=4 SV=1
2::A0A165XD09 **Mass:** 107765 **Score:** 52 **Matches:** 1(1) **Sequences:** 1(1)
>tr|A0A165XD09|A0A165XD09_MIMIV Heat shock protein 70-like protein OS=Mimivirus Bombay OX=1835008 PE=4 SV=1
2::A0A1E1EVQ4 **Mass:** 107765 **Score:** 52 **Matches:** 1(1) **Sequences:** 1(1)
>tr|A0A1E1EVQ4|A0A1E1EVQ4_9VIRU Heat shock protein 70-like protein OS=Acanthamoeba castellanii mimivirus OX=1899318 PE=4 SV=1
2::J2YWD0 **Mass:** 107765 **Score:** 52 **Matches:** 1(1) **Sequences:** 1(1)
>tr|J2YWD0|J2YWD0_9VIRU Heat shock 70Kd HSP OS=Acanthamoeba polyphaga lentillevirus OX=1077221 GN=L206 PE=4 SV=1
1::Q5UPU0 **Mass:** 107765 **Score:** 52 **Matches:** 1(1) **Sequences:** 1(1)
Heat shock protein 70 homolog OS=Acanthamoeba polyphaga mimivirus OX=212035 GN=MIMI_L254 PE=3 SV=1
2::V5L598 **Mass:** 107765 **Score:** 52 **Matches:** 1(1) **Sequences:** 1(1)
>tr|V5L598|V5L598_9VIRU 70 kDa heat shock protein-like protein OS=Hirudovirus strain Sangsue OX=1420594 GN=HIRU_S708 PE=4 SV=1
2::G8ECW7 **Mass:** 107765 **Score:** 52 **Matches:** 1(1) **Sequences:** 1(1)
>tr|G8ECW7|G8ECW7_9VIRU Heat shock 70Kd HSP OS=Acanthamoeba castellanii mamavirus OX=554168 GN=MAMA_L321 PE=4 SV=1

---

36. 2::A0A3S7SGD4 **Mass:** 7434 **Score:** 51 **Matches:** 2(2) **Sequences:** 1(1) **emPAI:** 0.72
>tr|A0A3S7SGD4|A0A3S7SGD4_9CAUD Uncharacterized protein OS=Aerococcus phage vB_AviM_AVP OX=2495576 GN=AVP_16 PE=4 SV=1

Query	Observed	Mr(expt)	Mr(calc)	ppm	Miss	Score	Expect	Rank	Unique	Peptide
612	503.2840	1004.5534	1004.5542	-0.80	0	46	0.0087	1	U	K.LDGLEFALK.T 611

---

37. 2::A0A4Y5FP44 **Mass:** 334087 **Score:** 51 **Matches:** 2(2) **Sequences:** 1(1) **emPAI:** 0.01
>tr|A0A4Y5FP44|A0A4Y5FP44_TSWV RNA dependent RNA polymerase OS=Tomato spotted wilt virus OX=1933298 GN=RdRp PE=4 SV=1

Query	Observed	Mr(expt)	Mr(calc)	ppm	Miss	Score	Expect	Rank	Unique	Peptide
648	508.7864	1015.5582	1015.5550	3.14	0	45	0.012	1	U	K.ITADGEGLIK.G 647

Proteins matching the same set of peptides:
2::A0A4Y5FP57 **Mass:** 334084 **Score:** 51 **Matches:** 2(2) **Sequences:** 1(1)
>tr|A0A4Y5FP57|A0A4Y5FP57_TSWV RNA dependent RNA polymerase OS=Tomato spotted wilt virus OX=1933298 GN=RdRp PE=4 SV=1

---

38. 2::A0A345MNF6 **Mass:** 105559 **Score:** 51 **Matches:** 1(1) **Sequences:** 1(1) **emPAI:** 0.04
>tr|A0A345MNF6|A0A345MNF6_9CAUD Isoleucyl-tRNA synthetase OS=Myoviridae sp. OX=2202564 PE=4 SV=1

Query	Observed	Mr(expt)	Mr(calc)	ppm	Miss	Score	Expect	Rank	Unique	Peptide
76	381.7293	761.4441	761.4436	0.70	0	51	0.003	1	U	K.TPLLYR.T

---

39. 2::A0A1U9WQR2 **Mass:** 19335 **Score:** 51 **Matches:** 1(1) **Sequences:** 1(1) **emPAI:** 0.24
>tr|A0A1U9WQR2|A0A1U9WQR2_9CAUD Uncharacterized protein OS=Geobacillus phage TP-84 OX=1965361 PE=4 SV=1

Query	Observed	Mr(expt)	Mr(calc)	ppm	Miss	Score	Expect	Rank	Unique	Peptide
949	562.2668	1122.5191	1122.5193	-0.14	0	51	0.0037	1	U	K.ESQQFEIDK.I

---

40. 2::A0A3G5A6F4 **Mass:** 24080 **Score:** 51 **Matches:** 2(1) **Sequences:** 1(1) **emPAI:** 0.19
>tr|A0A3G5A6F4|A0A3G5A6F4_9VIRU ANK_REP_REGION domain-containing protein OS=Hyperionvirus sp. OX=2487770 GN=Hyperionvirus2_195 PE=4 SV=1

Query	Observed	Mr(expt)	Mr(calc)	ppm	Miss	Score	Expect	Rank	Unique	Peptide
57	373.7152	745.4159	745.4156	0.42	0	51	0.024	1	U	K.LVMIDR.V
75	381.7128	761.4110	761.4106	0.55	0	(46)	0.08	1	U	K.LVMIDR.V

---

41. 2::A0A1V0DZB4 **Mass:** 48404 **Score:** 50 **Matches:** 1(1) **Sequences:** 1(1) **emPAI:** 0.09
>tr|A0A1V0DZB4|A0A1V0DZB4_9CAUD Replicative DNA helicase OS=Acinetobacter phage WCHABP12 OX=1965454 GN=dnaB PE=4 SV=1

Query	Observed	Mr(expt)	Mr(calc)	ppm	Miss	Score	Expect	Rank	Unique	Peptide
4	351.7058	701.3970	701.3959	1.45	0	50	0.0024	1	U	K.LDIVDK.A

Proteins matching the same set of peptides:

2::A0A1X6WG70 **Mass:** 156443 **Score:** 50 **Matches:** 1(1) **Sequences:** 1(1)
>tr|A0A1X6WG70|A0A1X6WG70_9VIRU Uncharacterized protein OS=Pacmanvirus A23 OX=1932881 GN=PACV_169 PE=4 SV=1
2::A0A1X9SFL1 **Mass:** 48404 **Score:** 50 **Matches:** 1(1) **Sequences:** 1(1)
>tr|A0A1X9SFL1|A0A1X9SFL1_9CAUD Replicative DNA helicase OS=Acinetobacter phage WCHABP1 OX=1983409 GN=dnaB PE=4 SV=1
2::A0A220NQM0 **Mass:** 48501 **Score:** 50 **Matches:** 1(1) **Sequences:** 1(1)
>tr|A0A220NQM0|A0A220NQM0_9CAUD Replicative DNA helicase OS=Acinetobacter phage AbP2 OX=2015804 GN=ABP2_079 PE=4 SV=1
2::A0A2L0UZ96 **Mass:** 87135 **Score:** 50 **Matches:** 1(1) **Sequences:** 1(1)
>tr|A0A2L0UZ96|A0A2L0UZ96_9CAUD Zeta_toxin domain-containing protein OS=Agrobacterium phage Atu_ph07 OX=2024264 PE=4 SV=1
2::A0A3G3BXJ7 **Mass:** 48461 **Score:** 50 **Matches:** 1(1) **Sequences:** 1(1)
>tr|A0A3G3BXJ7|A0A3G3BXJ7_9CAUD Replicative DNA helicase OS=Acinetobacter phage vB_KpnM_IME284 OX=2419625 PE=4 SV=1
2::A0A3G3BXU9 **Mass:** 48404 **Score:** 50 **Matches:** 1(1) **Sequences:** 1(1)
>tr|A0A3G3BXU9|A0A3G3BXU9_9CAUD Replicative DNA helicase OS=Acinetobacter phage vB_KpnM_IME512 OX=2419627 PE=4 SV=1
2::A0A482MGD6 **Mass:** 93180 **Score:** 50 **Matches:** 1(1) **Sequences:** 1(1)
>tr|A0A482MGD6|A0A482MGD6_9CAUD Tape measure protein OS=Serratia phage Serbin OX=2562181 GN=CPT_Serbin_021 PE=4 SV=1
2::A0A516LKU8 **Mass:** 7405 **Score:** 50 **Matches:** 1(1) **Sequences:** 1(1)
>tr|A0A516LKU8|A0A516LKU8_9VIRU Uncharacterized protein OS=Prokaryotic dsDNA virus sp. OX=2591644 GN=Tp118DCM00d2C30442581_17 PE=4 SV=1
2::A7DYD4 **Mass:** 35730 **Score:** 50 **Matches:** 1(1) **Sequences:** 1(1)
>tr|A7DYD4|A7DYD4_9CAUD Uncharacterized protein OS=Streptococcus phage M102 OX=372457 PE=4 SV=1

---

42. 2::A0A160DHM9 **Mass:** 9089 **Score:** 50 **Matches:** 2(2) **Sequences:** 1(1) **emPAI:** 0.56
>tr|A0A160DHM9|A0A160DHM9_9CAUD Uncharacterized protein OS=Gordonia phage Jeanie OX=1838068 GN=3 PE=4 SV=1

Query	Observed	Mr(expt)	Mr(calc)	ppm	Miss	Score	Expect	Rank	Unique	Peptide
168	412.2418	822.4691	822.4712	-2.52	0	41	0.0068	1	U	R.LVADHLR.H 169

Proteins matching the same set of peptides:

2::A0A160DHP6 **Mass:** 9089 **Score:** 50 **Matches:** 2(2) **Sequences:** 1(1)
>tr|A0A160DHP6|A0A160DHP6_9CAUD Uncharacterized protein OS=Gordonia phage McGonagall OX=1838072 GN=3 PE=4 SV=1
2::A0A4Y6EJ81 **Mass:** 8962 **Score:** 50 **Matches:** 2(2) **Sequences:** 1(1)
>tr|A0A4Y6EJ81|A0A4Y6EJ81_9CAUD Uncharacterized protein OS=Gordonia phage Coeur OX=2571246 GN=3 PE=4 SV=1

---

43. 2::Q6R4V1 **Mass:** 140130 **Score:** 50 **Matches:** 1(1) **Sequences:** 1(1) **emPAI:** 0.03
>tr|Q6R4V1|Q6R4V1_9CAUD Putative replication protein RepA OS=Vibrio virus VP882 OX=261726 PE=4 SV=1

Query	Observed	Mr(expt)	Mr(calc)	ppm	Miss	Score	Expect	Rank	Unique	Peptide
618	503.7832	1005.5519	1005.5495	2.40	0	50	0.0072	1	U	K.ALLEIYER.E

---

44. 2::A0A076JU09 **Mass:** 84694 **Score:** 49 **Matches:** 1(1) **Sequences:** 1(1) **emPAI:** 0.05
>tr|A0A076JU09|A0A076JU09_9ALPH Portal protein OS=Equid alphaherpesvirus 1 OX=10326 PE=3 SV=1

Query	Observed	Mr(expt)	Mr(calc)	ppm	Miss	Score	Expect	Rank	Unique	Peptide
56	373.7060	745.3975	745.3970	0.67	0	49	0.0074	1	U	K.TIEDLR.T

Proteins matching the same set of peptides:

2::A0A0A7D9M5 **Mass:** 84568 **Score:** 49 **Matches:** 1(1) **Sequences:** 1(1)
>tr|A0A0A7D9M5|A0A0A7D9M5_9ALPH Portal protein OS=Equid alphaherpesvirus 1 OX=10326 GN=ORF56 PE=3 SV=1
2::A0A0A7DAD3 **Mass:** 84554 **Score:** 49 **Matches:** 1(1) **Sequences:** 1(1)
>tr|A0A0A7DAD3|A0A0A7DAD3_9ALPH Portal protein OS=Equid alphaherpesvirus 1 OX=10326 GN=ORF56 PE=3 SV=1
2::A0A0X9ZHI2 **Mass:** 84184 **Score:** 49 **Matches:** 1(1) **Sequences:** 1(1)
>tr|A0A0X9ZHI2|A0A0X9ZHI2_9ALPH Portal protein OS=Equid alphaherpesvirus 4 OX=10331 GN=ORF56 PE=3 SV=1
2::A0A0Y0A4E8 **Mass:** 84170 **Score:** 49 **Matches:** 1(1) **Sequences:** 1(1)
>tr|A0A0Y0A4E8|A0A0Y0A4E8_9ALPH Portal protein OS=Equid alphaherpesvirus 4 OX=10331 GN=ORF56 PE=3 SV=1
2::A0A1J1E0I6 **Mass:** 84210 **Score:** 49 **Matches:** 1(1) **Sequences:** 1(1)
>tr|A0A1J1E0I6|A0A1J1E0I6_9ALPH Portal protein OS=Equid alphaherpesvirus 4 OX=10331 GN=ORF56 PE=3 SV=1
2::A0A2K9QRI7 **Mass:** 84599 **Score:** 49 **Matches:** 1(1) **Sequences:** 1(1)
>tr|A0A2K9QRI7|A0A2K9QRI7_9ALPH Portal protein OS=Equid alphaherpesvirus 8 OX=39637 GN=ORF56 PE=3 SV=1
2::A0A2K9QRR7 **Mass:** 84666 **Score:** 49 **Matches:** 1(1) **Sequences:** 1(1)
>tr|A0A2K9QRR7|A0A2K9QRR7_9ALPH Portal protein OS=Equid alphaherpesvirus 8 OX=39637 GN=ORF56 PE=3 SV=1
2::A0A2P0N522 **Mass:** 84610 **Score:** 49 **Matches:** 1(1) **Sequences:** 1(1)
>tr|A0A2P0N522|A0A2P0N522_9ALPH Portal protein OS=Equid alphaherpesvirus 1 OX=10326 GN=ORF56 PE=3 SV=1
2::A0A2P0N7P0 **Mass:** 84568 **Score:** 49 **Matches:** 1(1) **Sequences:** 1(1)
>tr|A0A2P0N7P0|A0A2P0N7P0_9ALPH Portal protein OS=Equid alphaherpesvirus 1 OX=10326 GN=ORF56 PE=3 SV=1
2::A0A2P0N8A0 **Mass:** 84525 **Score:** 49 **Matches:** 1(1) **Sequences:** 1(1)
>tr|A0A2P0N8A0|A0A2P0N8A0_9ALPH Portal protein OS=Equid alphaherpesvirus 1 OX=10326 GN=ORF56 PE=3 SV=1
2::A0A516LDA7 **Mass:** 97041 **Score:** 49 **Matches:** 1(1) **Sequences:** 1(1)
>tr|A0A516LDA7|A0A516LDA7_9VIRU Putative DNA ligase OS=Prokaryotic dsDNA virus sp. OX=2591644 GN=GOVbin4206_77 PE=4 SV=1
2::I1V8H0 **Mass:** 77644 **Score:** 49 **Matches:** 1(1) **Sequences:** 1(1)
>tr|I1V8H0|I1V8H0_9ALPH Portal protein OS=Equid alphaherpesvirus 8 OX=39637 GN=ORF56 PE=3 SV=1
2::M4JG74 **Mass:** 157566 **Score:** 49 **Matches:** 1(1) **Sequences:** 1(1)
>tr|M4JG74|M4JG74_9VIRU Capsid protein VP1 OS=Haloarcula hispanica virus PH1 OX=1282967 GN=HhPH1_gp12 PE=4 SV=1
2::B7FEJ8 **Mass:** 77689 **Score:** 49 **Matches:** 1(1) **Sequences:** 1(1)
>tr|B7FEJ8|B7FEJ8_9ALPH Portal protein OS=Equid alphaherpesvirus 9 OX=55744 PE=3 SV=1
2::L8B1V9 **Mass:** 84640 **Score:** 49 **Matches:** 1(1) **Sequences:** 1(1)
>tr|L8B1V9|L8B1V9_9ALPH Portal protein OS=Equid alphaherpesvirus 1 OX=10326 GN=ORF56 PE=3 SV=1
1::P28944 **Mass:** 84568 **Score:** 49 **Matches:** 1(1) **Sequences:** 1(1)
Portal protein OS=Equine herpesvirus 1 (strain Ab4p) OX=31520 GN=56 PE=3 SV=1
2::O39296 **Mass:** 84212 **Score:** 49 **Matches:** 1(1) **Sequences:** 1(1)
>tr|O39296|O39296_EHV4 Portal protein OS=Equine herpesvirus 4 (strain 1942) OX=10333 GN=56 PE=3 SV=1
1::P84458 **Mass:** 84568 **Score:** 49 **Matches:** 1(1) **Sequences:** 1(1)
Portal protein OS=Equine herpesvirus 1 (strain V592) OX=310273 GN=56 PE=3 SV=1

---

45. 2::A0A1L7DSB0 **Mass:** 85533 **Score:** 48 **Matches:** 1(1) **Sequences:** 1(1) **emPAI:** 0.05
>tr|A0A1L7DSB0|A0A1L7DSB0_9CAUD Tail tubular B OS=Ralstonia virus phiAp1 OX=1932865 GN=phiAp1_42 PE=4 SV=1

Query	Observed	Mr(expt)	Mr(calc)	ppm	Miss	Score	Expect	Rank	Unique	Peptide
213	421.2665	840.5184	840.5181	0.34	0	48	0.0021	1	U	R.LVVLAGNR.V

---

46. 2::A0A1S5RFA4 **Mass:** 61068 **Score:** 48 **Matches:** 2(2) **Sequences:** 1(1) **emPAI:** 0.07
>tr|A0A1S5RFA4|A0A1S5RFA4_9CAUD Putative terminase OS=Helicobacter phage SwA626G OX=1852684 PE=4 SV=1

Query	Observed	Mr(expt)	Mr(calc)	ppm	Miss	Score	Expect	Rank	Unique	Peptide
947	560.8228	1119.6310	1119.6288	1.92	0	43	0.012	1	U	R.QGGGLFVTTLK.S 946

---

47. 2::A0A514D6G7 **Mass:** 7907 **Score:** 47 **Matches:** 1(1) **Sequences:** 1(1) **emPAI:** 0.67
>tr|A0A514D6G7|A0A514D6G7_9VIRU Uncharacterized protein OS=Leviviridae sp. OX=2027243 GN=H2RhizoLitter493661_000002 PE=4 SV=1

Query	Observed	Mr(expt)	Mr(calc)	ppm	Miss	Score	Expect	Rank	Unique	Peptide
73	380.6871	759.3597	759.3585	1.60	0	47	0.0022	1	U	R.MPEDLR.K

Proteins matching the same set of peptides:

2::A0A5B8RQV3 **Mass:** 85639 **Score:** 47 **Matches:** 1(1) **Sequences:** 1(1)
>tr|A0A5B8RQV3|A0A5B8RQV3_9CAUD DNA polymerase OS=Salmonella phage Shemara OX=2596714 GN=CPT_Shemara_044 PE=4 SV=1

---

48. 2::Q75491 **Mass:** 13951 **Score:** 46 **Matches:** 1(1) **Sequences:** 1(1) **emPAI:** 0.34
>tr|Q75491|Q75491_9HIV1 Envelope glycoprotein (Fragment) OS=Human immunodeficiency virus 1 OX=11676 GN=env PE=4 SV=1

Query	Observed	Mr(expt)	Mr(calc)	ppm	Miss	Score	Expect	Rank	Unique	Peptide
31	362.7084	723.4023	723.4028	-0.65	0	46	0.0039	1	U	K.IADHLR.E

49. 2::A0A345MUY6 **Mass:** 32986 **Score:** 45 **Matches:** 1(1) **Sequences:** 1(1) **emPAI:** 0.14
>tr|A0A345MUY6|A0A345MUY6_9VIRU Putative replication initiation protein OS=Microviridae sp. OX=2202644 PE=4 SV=1

Query	Observed	Mr(expt)	Mr(calc)	ppm	Miss	Score	Expect	Rank	Unique	Peptide
101	391.6967	781.3789	781.3792	-0.42	0	45	0.0071	1	U	R.MLEAYR.K

Proteins matching the same set of peptides:
2::V5Q7L8 **Mass:** 23777 **Score:** 45 **Matches:** 1(1) **Sequences:** 1(1)
>tr|V5Q7L8|V5Q7L8_9CAUD Uncharacterized protein OS=Xylella phage Paz OX=1415145 GN=Paz_22 PE=4 SV=1

---

50. 2::A0A059UHZ7 **Mass:** 130888 **Score:** 45 **Matches:** 1(1) **Sequences:** 1(1) **emPAI:** 0.03
>tr|A0A059UHZ7|A0A059UHZ7_9RETR Pol protein (Fragment) OS=Feline immunodeficiency virus OX=11673 GN=pol PE=3 SV=1

Query	Observed	Mr(expt)	Mr(calc)	ppm	Miss	Score	Expect	Rank	Unique	Peptide
380	458.2612	914.5078	914.5073	0.60	0	45	0.054	1	U	K.QVADEIIK.K

---

51. 2::A0A345MNE4 **Mass:** 73419 **Score:** 45 **Matches:** 1(1) **Sequences:** 1(1) **emPAI:** 0.06
>tr|A0A345MNE4|A0A345MNE4_9CAUD Helicase domain protein OS=Myoviridae sp. OX=2202564 PE=4 SV=1

Query	Observed	Mr(expt)	Mr(calc)	ppm	Miss	Score	Expect	Rank	Unique	Peptide
1886	687.3987	1372.7829	1372.7814	1.13	0	45	0.0026	1	U	K.LISDVTGLTDIVK.V

---

52. 2::A0A075W7P3 **Mass:** 11545 **Score:** 44 **Matches:** 1(1) **Sequences:** 1(1) **emPAI:** 0.43
>tr|A0A075W7P3|A0A075W7P3_9ENTO VP1 (Fragment) OS=Enterovirus B93 OX=991902 PE=4 SV=1

Query	Observed	Mr(expt)	Mr(calc)	ppm	Miss	Score	Expect	Rank	Unique	Peptide
11	358.2206	714.4267	714.4276	-1.17	0	44	0.023	1	U	R.DLVQLK.R

Proteins matching the same set of peptides:
2::A0A2I7RNA2 **Mass:** 10198 **Score:** 44 **Matches:** 1(1) **Sequences:** 1(1)
>tr|A0A2I7RNA2|A0A2I7RNA2_9CAUD Coil containing protein OS=Vibrio phage 1.201.B._10N.286.55.F1 OX=1881261 GN=NVP1201B_20 PE=4 SV=1
2::A0A2P1I0P7 **Mass:** 74154 **Score:** 44 **Matches:** 1(1) **Sequences:** 1(1)
>tr|A0A2P1I0P7|A0A2P1I0P7_HPV16 Replication protein E1 OS=Human papillomavirus type 16 OX=333760 GN=E1 PE=3 SV=1
2::A0A2P1IEL5 **Mass:** 74163 **Score:** 44 **Matches:** 1(1) **Sequences:** 1(1)
>tr|A0A2P1IEL5|A0A2P1IEL5_HPV16 Replication protein E1 OS=Human papillomavirus type 16 OX=333760 GN=E1 PE=3 SV=1
2::A0A514D3X3 **Mass:** 58466 **Score:** 44 **Matches:** 1(1) **Sequences:** 1(1)
>tr|A0A514D3X3|A0A514D3X3_9VIRU RNA-dependent RNA polymerase OS=Riboviria sp. OX=2585031 GN=H4BulkLitter231857_000002 PE=4 SV=1

---

53. 2::A0A0S3QM10 **Mass:** 23371 **Score:** 44 **Matches:** 1(1) **Sequences:** 1(1) **emPAI:** 0.20
>tr|A0A0S3QM10|A0A0S3QM10_9HIV1 Protein Nef OS=Human immunodeficiency virus 1 OX=11676 GN=nef PE=3 SV=1

Query	Observed	Mr(expt)	Mr(calc)	ppm	Miss	Score	Expect	Rank	Unique	Peptide
30	362.7082	723.4018	723.4028	-1.34	0	44	0.0062	1	U	K.HIAVER.H

Proteins matching the same set of peptides:
2::A0A0S3QMI2 **Mass:** 23928 **Score:** 44 **Matches:** 1(1) **Sequences:** 1(1)
>tr|A0A0S3QMI2|A0A0S3QMI2_9HIV1 Protein Nef OS=Human immunodeficiency virus 1 OX=11676 GN=nef PE=2 SV=1

---

54. 2::A0A3G5A8B3 **Mass:** 49883 **Score:** 44 **Matches:** 1(1) **Sequences:** 1(1) **emPAI:** 0.09
>tr|A0A3G5A8B3|A0A3G5A8B3_9VIRU FkbM family methyltransferase OS=Hyperionvirus sp. OX=2487770 GN=Hyperionvirus7_78 PE=4 SV=1

Query	Observed	Mr(expt)	Mr(calc)	ppm	Miss	Score	Expect	Rank	Unique	Peptide
316	444.2635	886.5125	886.5123	0.18	1	44	0.016	1	U	K.KIADAIEK.I

---

55. 2::A0A059T7Q4 **Mass:** 113372 **Score:** 44 **Matches:** 1(1) **Sequences:** 1(1) **emPAI:** 0.04
>tr|A0A059T7Q4|A0A059T7Q4_9CAUD Tape measure protein OS=Staphylococcus phage DW2 OX=1464127 GN=DW2_52 PE=4 SV=1

Query	Observed	Mr(expt)	Mr(calc)	ppm	Miss	Score	Expect	Rank	Unique	Peptide
40	365.7271	729.4397	729.4385	1.67	0	44	0.036	1	U	R.ILDLTR.I

Proteins matching the same set of peptides:
2::A0A076VAE9 **Mass:** 266299 **Score:** 44 **Matches:** 1(1) **Sequences:** 1(1)
>tr|A0A076VAE9|A0A076VAE9_9PICO Genome polyprotein OS=Chicken picornavirus 1 OX=1534544 PE=4 SV=1
2::A0A0E3XC73 **Mass:** 103700 **Score:** 44 **Matches:** 1(1) **Sequences:** 1(1)
>tr|A0A0E3XC73|A0A0E3XC73_9CAUD Tape measure protein OS=Staphylococcus phage B236 OX=1636205 PE=4 SV=1
2::A0A0H3U2Z1 **Mass:** 102958 **Score:** 44 **Matches:** 1(1) **Sequences:** 1(1)
>tr|A0A0H3U2Z1|A0A0H3U2Z1_9CAUD Tapemeasure OS=Staphylococcus phage 3MRA OX=1505026 PE=4 SV=1
2::A0A0N9BAY4 **Mass:** 113835 **Score:** 44 **Matches:** 1(1) **Sequences:** 1(1)
>tr|A0A0N9BAY4|A0A0N9BAY4_9CAUD Tape measure protein OS=Staphylococcus phage phiJB OX=1698421 GN=tmp PE=4 SV=1
2::A0A2P1IP96 **Mass:** 135732 **Score:** 44 **Matches:** 1(1) **Sequences:** 1(1)
>tr|A0A2P1IP96|A0A2P1IP96_9PICO Polyprotein (Fragment) OS=Sicinivirus sp. OX=1930506 PE=4 SV=1
2::A0A482MGI8 **Mass:** 103352 **Score:** 44 **Matches:** 1(1) **Sequences:** 1(1)
>tr|A0A482MGI8|A0A482MGI8_9CAUD Tape measure protein OS=Staphylococcus phage Sebago OX=2557555 GN=CPT_Sebago_017 PE=4 SV=1
2::A0EX72 **Mass:** 113835 **Score:** 44 **Matches:** 1(1) **Sequences:** 1(1)
>tr|A0EX72|A0EX72_9CAUD Phage tape measure protein OS=Staphylococcus virus phiNM4 OX=387910 PE=4 SV=1
2::A1KXA6 **Mass:** 103686 **Score:** 44 **Matches:** 1(1) **Sequences:** 1(1)
>tr|A1KXA6|A1KXA6_9CAUD Uncharacterized protein OS=Staphylococcus virus phiETA3 OX=326037 PE=4 SV=1
2::Q4ZBQ5 **Mass:** 113835 **Score:** 44 **Matches:** 1(1) **Sequences:** 1(1)
>tr|Q4ZBQ5|Q4ZBQ5_9CAUD ORF001 OS=Staphylococcus phage ROSA OX=320843 PE=4 SV=1
2::R4IG82 **Mass:** 106540 **Score:** 44 **Matches:** 1(1) **Sequences:** 1(1)
>tr|R4IG82|R4IG82_9CAUD Putative tape measure protein OS=Staphylococcus phage SA13 OX=1195086 GN=SA13_49 PE=4 SV=1
2::M9NRJ5 **Mass:** 102336 **Score:** 44 **Matches:** 1(1) **Sequences:** 1(1)
>tr|M9NRJ5|M9NRJ5_9CAUD Uncharacterized protein OS=Staphylococcus phage StauST398-3 OX=1195071 GN=StauST398-3_0055 PE=4 SV=1

---

56. 2::A0A2D2W767 **Mass:** 12740 **Score:** 43 **Matches:** 1(1) **Sequences:** 1(1) **emPAI:** 0.38
>tr|A0A2D2W767|A0A2D2W767_9CAUD Uncharacterized protein OS=Pectobacterium phage DU_PP_V OX=2041492 GN=P13BB106kb_p046 PE=4 SV=1

Query	Observed	Mr(expt)	Mr(calc)	ppm	Miss	Score	Expect	Rank	Unique	Peptide
177	414.7509	827.4873	827.4865	0.96	0	43	0.046	1	U	K.IANLELR.A

---

57. 2::A0A1W5J0S3 **Mass:** 20347 **Score:** 43 **Matches:** 1(1) **Sequences:** 1(1) **emPAI:** 0.23
>tr|A0A1W5J0S3|A0A1W5J0S3_9MONO RNA-dependent RNA polymerase (Fragment) OS=Paramyxovirus PREDICT_PMV-18 OX=1647218 PE=4 SV=1

Query	Observed	Mr(expt)	Mr(calc)	ppm	Miss	Score	Expect	Rank	Unique	Peptide
87	386.7371	771.4596	771.4603	-0.89	0	43	0.033	1	U	-.QAIAITR.R

Proteins matching the same set of peptides:

2::A0A1W5J0V7 **Mass:** 18895 **Score:** 43 **Matches:** 1(1) **Sequences:** 1(1)
>tr|A0A1W5J0V7|A0A1W5J0V7_9MONO RNA-dependent RNA polymerase (Fragment) OS=Paramyxovirus PREDICT_PMV-53 OX=1647253 PE=4 SV=1
2::A0A1W5J0X9 **Mass:** 20257 **Score:** 43 **Matches:** 1(1) **Sequences:** 1(1)
>tr|A0A1W5J0X9|A0A1W5J0X9_9MONO RNA-dependent RNA polymerase (Fragment) OS=Paramyxovirus PREDICT_PMV-13 OX=1647213 PE=4 SV=1
2::A0A1W5J1C7 **Mass:** 18748 **Score:** 43 **Matches:** 1(1) **Sequences:** 1(1)
>tr|A0A1W5J1C7|A0A1W5J1C7_9MONO RNA-dependent RNA polymerase (Fragment) OS=Paramyxovirus PREDICT_PMV-53 OX=1647253 PE=4 SV=1
2::A0A1W5J1E8 **Mass:** 20247 **Score:** 43 **Matches:** 1(1) **Sequences:** 1(1)
>tr|A0A1W5J1E8|A0A1W5J1E8_9MONO RNA-dependent RNA polymerase (Fragment) OS=Paramyxovirus PREDICT_PMV-13 OX=1647213 PE=4 SV=1
2::A0A1W5J1G0 **Mass:** 20187 **Score:** 43 **Matches:** 1(1) **Sequences:** 1(1)
>tr|A0A1W5J1G0|A0A1W5J1G0_9MONO RNA-dependent RNA polymerase (Fragment) OS=Paramyxovirus PREDICT_PMV-66 OX=1647265 PE=4 SV=1
2::A0A1W5J265 **Mass:** 20196 **Score:** 43 **Matches:** 1(1) **Sequences:** 1(1)
>tr|A0A1W5J265|A0A1W5J265_9MONO RNA-dependent RNA polymerase (Fragment) OS=Paramyxovirus PREDICT_PMV-32 OX=1647232 PE=4 SV=1
2::A0A1W5J273 **Mass:** 18677 **Score:** 43 **Matches:** 1(1) **Sequences:** 1(1)
>tr|A0A1W5J273|A0A1W5J273_9MONO RNA-dependent RNA polymerase (Fragment) OS=Paramyxovirus PREDICT_PMV-13 OX=1647213 PE=4 SV=1
2::A0A1W5J2A1 **Mass:** 18447 **Score:** 43 **Matches:** 1(1) **Sequences:** 1(1)
>tr|A0A1W5J2A1|A0A1W5J2A1_9MONO RNA-dependent RNA polymerase (Fragment) OS=Paramyxovirus PREDICT_PMV-46 OX=1647246 PE=4 SV=1
2::A0A1W5J2A8 **Mass:** 18118 **Score:** 43 **Matches:** 1(1) **Sequences:** 1(1)
>tr|A0A1W5J2A8|A0A1W5J2A8_9MONO RNA-dependent RNA polymerase (Fragment) OS=Paramyxovirus PREDICT_PMV-19 OX=1647219 PE=4 SV=1
2::A0A1W5J2G1 **Mass:** 18543 **Score:** 43 **Matches:** 1(1) **Sequences:** 1(1)
>tr|A0A1W5J2G1|A0A1W5J2G1_9MONO RNA-dependent RNA polymerase (Fragment) OS=Paramyxovirus PREDICT_PMV-52 OX=1647252 PE=4 SV=1
2::A0A1W5J2H1 **Mass:** 18895 **Score:** 43 **Matches:** 1(1) **Sequences:** 1(1)
>tr|A0A1W5J2H1|A0A1W5J2H1_9MONO RNA-dependent RNA polymerase (Fragment) OS=Paramyxovirus PREDICT_PMV-53 OX=1647253 PE=4 SV=1
2::A0A1W5J2U5 **Mass:** 20196 **Score:** 43 **Matches:** 1(1) **Sequences:** 1(1)
>tr|A0A1W5J2U5|A0A1W5J2U5_9MONO RNA-dependent RNA polymerase (Fragment) OS=Paramyxovirus PREDICT_PMV-32 OX=1647232 PE=4 SV=1
2::A0A1W5J350 **Mass:** 18689 **Score:** 43 **Matches:** 1(1) **Sequences:** 1(1)
>tr|A0A1W5J350|A0A1W5J350_9MONO RNA-dependent RNA polymerase (Fragment) OS=Paramyxovirus PREDICT_PMV-51 OX=1647251 PE=4 SV=1
2::A0A1W5J360 **Mass:** 18895 **Score:** 43 **Matches:** 1(1) **Sequences:** 1(1)
>tr|A0A1W5J360|A0A1W5J360_9MONO RNA-dependent RNA polymerase (Fragment) OS=Paramyxovirus PREDICT_PMV-53 OX=1647253 PE=4 SV=1
2::A0A1W5J3F1 **Mass:** 18251 **Score:** 43 **Matches:** 1(1) **Sequences:** 1(1)
>tr|A0A1W5J3F1|A0A1W5J3F1_9MONO RNA-dependent RNA polymerase (Fragment) OS=Paramyxovirus PREDICT_PMV-60 OX=1647260 PE=4 SV=1
2::A0A1W5J3G6 **Mass:** 18236 **Score:** 43 **Matches:** 1(1) **Sequences:** 1(1)
>tr|A0A1W5J3G6|A0A1W5J3G6_9MONO RNA-dependent RNA polymerase (Fragment) OS=Paramyxovirus PREDICT_PMV-29 OX=1647229 PE=4 SV=1
2::A0A1W5J3H2 **Mass:** 18895 **Score:** 43 **Matches:** 1(1) **Sequences:** 1(1)
>tr|A0A1W5J3H2|A0A1W5J3H2_9MONO RNA-dependent RNA polymerase (Fragment) OS=Paramyxovirus PREDICT_PMV-53 OX=1647253 PE=4 SV=1
2::A0A1W5J3J2 **Mass:** 20247 **Score:** 43 **Matches:** 1(1) **Sequences:** 1(1)
>tr|A0A1W5J3J2|A0A1W5J3J2_9MONO RNA-dependent RNA polymerase (Fragment) OS=Paramyxovirus PREDICT_PMV-13 OX=1647213 PE=4 SV=1
2::A0A1W5J3K3 **Mass:** 20229 **Score:** 43 **Matches:** 1(1) **Sequences:** 1(1)
>tr|A0A1W5J3K3|A0A1W5J3K3_9MONO RNA-dependent RNA polymerase (Fragment) OS=Paramyxovirus PREDICT_PMV-66 OX=1647265 PE=4 SV=1
2::A0A1W5J3M3 **Mass:** 20321 **Score:** 43 **Matches:** 1(1) **Sequences:** 1(1)
>tr|A0A1W5J3M3|A0A1W5J3M3_9MONO RNA-dependent RNA polymerase (Fragment) OS=Paramyxovirus PREDICT_PMV-18 OX=1647218 PE=4 SV=1
2::A0A1W5J3Q2 **Mass:** 18895 **Score:** 43 **Matches:** 1(1) **Sequences:** 1(1)
>tr|A0A1W5J3Q2|A0A1W5J3Q2_9MONO RNA-dependent RNA polymerase (Fragment) OS=Paramyxovirus PREDICT_PMV-53 OX=1647253 PE=4 SV=1
2::A0A1W5J4P8 **Mass:** 20196 **Score:** 43 **Matches:** 1(1) **Sequences:** 1(1)
>tr|A0A1W5J4P8|A0A1W5J4P8_9MONO RNA-dependent RNA polymerase (Fragment) OS=Paramyxovirus PREDICT_PMV-32 OX=1647232 PE=4 SV=1
2::A0A1W5J4T0 **Mass:** 18408 **Score:** 43 **Matches:** 1(1) **Sequences:** 1(1)
>tr|A0A1W5J4T0|A0A1W5J4T0_9MONO RNA-dependent RNA polymerase (Fragment) OS=Paramyxovirus PREDICT_PMV-49 OX=1647249 PE=4 SV=1
2::A0A1W5J4Z0 **Mass:** 18895 **Score:** 43 **Matches:** 1(1) **Sequences:** 1(1)
>tr|A0A1W5J4Z0|A0A1W5J4Z0_9MONO RNA-dependent RNA polymerase (Fragment) OS=Paramyxovirus PREDICT_PMV-53 OX=1647253 PE=4 SV=1
2::A0A1W5J508 **Mass:** 20247 **Score:** 43 **Matches:** 1(1) **Sequences:** 1(1)
>tr|A0A1W5J508|A0A1W5J508_9MONO RNA-dependent RNA polymerase (Fragment) OS=Paramyxovirus PREDICT_PMV-13 OX=1647213 PE=4 SV=1
2::A0A1W5J517 **Mass:** 20229 **Score:** 43 **Matches:** 1(1) **Sequences:** 1(1)
>tr|A0A1W5J517|A0A1W5J517_9MONO RNA-dependent RNA polymerase (Fragment) OS=Paramyxovirus PREDICT_PMV-66 OX=1647265 PE=4 SV=1
2::A0A1W5J5K7 **Mass:** 20169 **Score:** 43 **Matches:** 1(1) **Sequences:** 1(1)
>tr|A0A1W5J5K7|A0A1W5J5K7_9MONO RNA-dependent RNA polymerase (Fragment) OS=Paramyxovirus PREDICT_PMV-32 OX=1647232 PE=4 SV=1
2::A0A1W5J5U9 **Mass:** 18895 **Score:** 43 **Matches:** 1(1) **Sequences:** 1(1)
>tr|A0A1W5J5U9|A0A1W5J5U9_9MONO RNA-dependent RNA polymerase (Fragment) OS=Paramyxovirus PREDICT_PMV-53 OX=1647253 PE=4 SV=1
2::A0A1W5J5V8 **Mass:** 18895 **Score:** 43 **Matches:** 1(1) **Sequences:** 1(1)
>tr|A0A1W5J5V8|A0A1W5J5V8_9MONO RNA-dependent RNA polymerase (Fragment) OS=Paramyxovirus PREDICT_PMV-53 OX=1647253 PE=4 SV=1
2::A0A1W5J5W5 **Mass:** 20247 **Score:** 43 **Matches:** 1(1) **Sequences:** 1(1)
>tr|A0A1W5J5W5|A0A1W5J5W5_9MONO RNA-dependent RNA polymerase (Fragment) OS=Paramyxovirus PREDICT_PMV-13 OX=1647213 PE=4 SV=1
2::A0A1W5J5X5 **Mass:** 20164 **Score:** 43 **Matches:** 1(1) **Sequences:** 1(1)
>tr|A0A1W5J5X5|A0A1W5J5X5_9MONO RNA-dependent RNA polymerase (Fragment) OS=Paramyxovirus PREDICT_PMV-66 OX=1647265 PE=4 SV=1
2::A0A1W5J6W0 **Mass:** 20196 **Score:** 43 **Matches:** 1(1) **Sequences:** 1(1)
>tr|A0A1W5J6W0|A0A1W5J6W0_9MONO RNA-dependent RNA polymerase (Fragment) OS=Paramyxovirus PREDICT_PMV-32 OX=1647232 PE=4 SV=1
2::A0A1W5J6W8 **Mass:** 18677 **Score:** 43 **Matches:** 1(1) **Sequences:** 1(1)
>tr|A0A1W5J6W8|A0A1W5J6W8_9MONO RNA-dependent RNA polymerase (Fragment) OS=Paramyxovirus PREDICT_PMV-13 OX=1647213 PE=4 SV=1
2::A0A1W5J754 **Mass:** 18895 **Score:** 43 **Matches:** 1(1) **Sequences:** 1(1)
>tr|A0A1W5J754|A0A1W5J754_9MONO RNA-dependent RNA polymerase (Fragment) OS=Paramyxovirus PREDICT_PMV-53 OX=1647253 PE=4 SV=1
2::A0A1W5J772 **Mass:** 20247 **Score:** 43 **Matches:** 1(1) **Sequences:** 1(1)
>tr|A0A1W5J772|A0A1W5J772_9MONO RNA-dependent RNA polymerase (Fragment) OS=Paramyxovirus PREDICT_PMV-13 OX=1647213 PE=4 SV=1
2::A0A1W5J782 **Mass:** 20213 **Score:** 43 **Matches:** 1(1) **Sequences:** 1(1)
>tr|A0A1W5J782|A0A1W5J782_9MONO RNA-dependent RNA polymerase (Fragment) OS=Paramyxovirus PREDICT_PMV-66 OX=1647265 PE=4 SV=1
2::A0A1W5J8T3 **Mass:** 20196 **Score:** 43 **Matches:** 1(1) **Sequences:** 1(1)
>tr|A0A1W5J8T3|A0A1W5J8T3_9MONO RNA-dependent RNA polymerase (Fragment) OS=Paramyxovirus PREDICT_PMV-32 OX=1647232 PE=4 SV=1
2::A0A1W5J928 **Mass:** 18480 **Score:** 43 **Matches:** 1(1) **Sequences:** 1(1)
>tr|A0A1W5J928|A0A1W5J928_9MONO RNA-dependent RNA polymerase (Fragment) OS=Paramyxovirus PREDICT_PMV-50 OX=1647250 PE=4 SV=1
2::A0A1W5J938 **Mass:** 18895 **Score:** 43 **Matches:** 1(1) **Sequences:** 1(1)
>tr|A0A1W5J938|A0A1W5J938_9MONO RNA-dependent RNA polymerase (Fragment) OS=Paramyxovirus PREDICT_PMV-53 OX=1647253 PE=4 SV=1
2::A0A1Z1W2A4 **Mass:** 20087 **Score:** 43 **Matches:** 1(1) **Sequences:** 1(1)
>tr|A0A1Z1W2A4|A0A1Z1W2A4_9MONO Large polymerase-like protein (Fragment) OS=Bat paramyxovirus OX=1300978 GN=L PE=4 SV=1

---

58. 2::A0A0D3MVZ4 **Mass:** 20513 **Score:** 43 **Matches:** 1(1) **Sequences:** 1(1) **emPAI:** 0.23
>tr|A0A0D3MVZ4|A0A0D3MVZ4_9ADEN Ig-like domain-containing protein OS=Duck adenovirus 1 OX=130329 PE=4 SV=1

Query	Observed	Mr(expt)	Mr(calc)	ppm	Miss	Score	Expect	Rank	Unique	Peptide
140	404.2218	806.4290	806.4286	0.45	0	43	0.033	1	U	K.LLDYQR.L

Proteins matching the same set of peptides:

2::O11432 **Mass:** 20527 **Score:** 43 **Matches:** 1(1) **Sequences:** 1(1)
>tr|O11432|O11432_9ADEN Ig-like domain-containing protein OS=Duck adenovirus 1 OX=130329 PE=4 SV=1

---

59. 2::A0A0G2XZL2 **Mass:** 64703 **Score:** 43 **Matches:** 1(1) **Sequences:** 1(1) **emPAI:** 0.07
>tr|A0A0G2XZL2|A0A0G2XZL2_MIMIV Putative sel1-like repeat-containing protein OS=Acanthamoeba polyphaga mimivirus OX=212035 PE=4 SV=1

Query	Observed	Mr(expt)	Mr(calc)	ppm	Miss	Score	Expect	Rank	Unique	Peptide
7	353.7340	705.4534	705.4537	-0.43	0	43	0.00033	1	U	K.IHPIVK.Q

Proteins matching the same set of peptides:

2::A0A0G2Y9Z1 **Mass:** 64102 **Score:** 43 **Matches:** 1(1) **Sequences:** 1(1)
>tr|A0A0G2Y9Z1|A0A0G2Y9Z1_MIMIV Putative sel1-like repeat-containing protein OS=Acanthamoeba polyphaga mimivirus OX=212035 GN=L21 PE=4 SV=1
2::A0A0U2UGR2 **Mass:** 64102 **Score:** 43 **Matches:** 1(1) **Sequences:** 1(1)
>tr|A0A0U2UGR2|A0A0U2UGR2_9VIRU Uncharacterized protein OS=Niemeyer virus OX=1766765 PE=4 SV=1
2::A0A140E086 **Mass:** 64102 **Score:** 43 **Matches:** 1(1) **Sequences:** 1(1)
>tr|A0A140E086|A0A140E086_MIMIV Sel1-like repeat-containing protein OS=Samba virus OX=1461100 PE=4 SV=1
2::A0A165X5V1 **Mass:** 64102 **Score:** 43 **Matches:** 1(1) **Sequences:** 1(1)
>tr|A0A165X5V1|A0A165X5V1_MIMIV Putative sel1-like repeat-containing protein OS=Mimivirus Bombay OX=1835008 PE=4 SV=1
2::A0A1E1ES63 **Mass:** 64102 **Score:** 43 **Matches:** 1(1) **Sequences:** 1(1)
>tr|A0A1E1ES63|A0A1E1ES63_9VIRU Putative sel1-like repeat-containing protein OS=Acanthamoeba castellanii mimivirus OX=1899318 PE=4 SV=1
2::J3IZN9 **Mass:** 64102 **Score:** 43 **Matches:** 1(1) **Sequences:** 1(1)
>tr|J3IZN9|J3IZN9_9VIRU Uncharacterized protein OS=Acanthamoeba polyphaga lentillevirus OX=1077221 GN=L4 PE=4 SV=1
1::Q5UP97 **Mass:** 64102 **Score:** 43 **Matches:** 1(1) **Sequences:** 1(1)
Putative sel1-like repeat-containing protein L21 OS=Acanthamoeba polyphaga mimivirus OX=212035 GN=MIMI_L21 PE=4 SV=1
2::V5L439 **Mass:** 64102 **Score:** 43 **Matches:** 1(1) **Sequences:** 1(1)
>tr|V5L439|V5L439_9VIRU Putative sel1-like repeat-containing protein OS=Hirudovirus strain Sangsue OX=1420594 GN=HIRU_S965 PE=4 SV=1
2::G8EC62 **Mass:** 64102 **Score:** 43 **Matches:** 1(1) **Sequences:** 1(1)
>tr|G8EC62|G8EC62_9VIRU Uncharacterized protein MAMA_L53 OS=Acanthamoeba castellanii mamavirus OX=554168 GN=MAMA_L53 PE=4 SV=1

---

60. 1::C1JCT2 **Mass:** 392767 **Score:** 43 **Matches:** 1(1) **Sequences:** 1(1) **emPAI:** 0.01
Polyprotein-FSD OS=Solenopsis invicta virus 3 OX=631345 PE=1 SV=2

Query	Observed	Mr(expt)	Mr(calc)	ppm	Miss	Score	Expect	Rank	Unique	Peptide
932	557.8145	1113.6145	1113.6142	0.29	0	43	0.029	1	U	K.VIQTGNNLAGK.H

---

61. 2::A0A1X9IA00 **Mass:** 18329 **Score:** 43 **Matches:** 1(1) **Sequences:** 1(1) **emPAI:** 0.25
>tr|A0A1X9IA00|A0A1X9IA00_9CAUD Uncharacterized protein OS=Staphylococcus phage vB_SscM-1 OX=1868844 GN=vB_SscM-1_182 PE=4 SV=1

Query	Observed	Mr(expt)	Mr(calc)	ppm	Miss	Score	Expect	Rank	Unique	Peptide
90	387.2289	772.4433	772.4443	-1.24	1	43	0.046	1	U	K.DKELIR.I

Proteins matching the same set of peptides:

2::A0A1X9IAJ6 **Mass:** 18329 **Score:** 43 **Matches:** 1(1) **Sequences:** 1(1)
>tr|A0A1X9IAJ6|A0A1X9IAJ6_9CAUD Uncharacterized protein OS=Staphylococcus phage vB_SscM-2 OX=1868845 GN=vB_SscM-2_181 PE=4 SV=1
2::A0A4D5XEX7 **Mass:** 47382 **Score:** 43 **Matches:** 1(1) **Sequences:** 1(1)
>tr|A0A4D5XEX7|A0A4D5XEX7_9VIRU Uncharacterized protein OS=Mimivirus LCMiAC02 OX=2506609 GN=LCMiAC02_03920 PE=4 SV=1

---

62. 2::A0A0K0KVG1 **Mass:** 284825 **Score:** 42 **Matches:** 1(1) **Sequences:** 1(1) **emPAI:** 0.02
>tr|A0A0K0KVG1|A0A0K0KVG1_9CAUD Putative neck protein OS=Prochlorococcus phage P-TIM68 OX=1542477 PE=4 SV=1

Query	Observed	Mr(expt)	Mr(calc)	ppm	Miss	Score	Expect	Rank	Unique	Peptide
86	386.7215	771.4285	771.4279	0.76	0	42	0.022	1	U	K.FDIHIK.D

---

63. 2::A0A2H4UVE2 **Mass:** 39272 **Score:** 42 **Matches:** 1(1) **Sequences:** 1(1) **emPAI:** 0.11
>tr|A0A2H4UVE2|A0A2H4UVE2_9VIRU Serine/threonine-protein kinase OS=Bodo saltans virus OX=2024608 GN=BMW23_0847 PE=4 SV=1

Query	Observed	Mr(expt)	Mr(calc)	ppm	Miss	Score	Expect	Rank	Unique	Peptide
546	493.7986	985.5826	985.5808	1.81	0	42	0.024	1	U	K.ITNDIVALK.T

---

64. 2::A0A1V0SKH4 **Mass:** 66751 **Score:** 41 **Matches:** 1(1) **Sequences:** 1(1) **emPAI:** 0.07
>tr|A0A1V0SKH4|A0A1V0SKH4_9VIRU AAA family ATPase OS=Klosneuvirus KNV1 OX=1977640 GN=Klosneuvirus_4_37 PE=4 SV=1

Query	Observed	Mr(expt)	Mr(calc)	ppm	Miss	Score	Expect	Rank	Unique	Peptide
2190	484.9332	1451.7778	1451.7847	-4.74	0	41	0.035	1	U	K.LPMFIVDLSVFR.N

---

65. 2::A0A023VZE8 **Mass:** 58864 **Score:** 41 **Matches:** 1(1) **Sequences:** 1(1) **emPAI:** 0.07
>tr|A0A023VZE8|A0A023VZE8_BSMV Beta-B protein OS=Barley stripe mosaic virus OX=12327 PE=4 SV=1

Query	Observed	Mr(expt)	Mr(calc)	ppm	Miss	Score	Expect	Rank	Unique	Peptide
144	405.2229	808.4312	808.4330	-2.32	0	41	0.025	1	U	R.LASLYDK.H

Proteins matching the same set of peptides:

2::A0A0G2RAA1 **Mass:** 56933 **Score:** 41 **Matches:** 1(1) **Sequences:** 1(1)
>tr|A0A0G2RAA1|A0A0G2RAA1_BSMV Beta b protein OS=Barley stripe mosaic virus OX=12327 PE=4 SV=1
2::A0A2K9YRQ0 **Mass:** 57069 **Score:** 41 **Matches:** 1(1) **Sequences:** 1(1)
>tr|A0A2K9YRQ0|A0A2K9YRQ0_BSMV Beta b protein OS=Barley stripe mosaic virus OX=12327 PE=4 SV=1
2::A0A2K9YS21 **Mass:** 56872 **Score:** 41 **Matches:** 1(1) **Sequences:** 1(1)
>tr|A0A2K9YS21|A0A2K9YS21_BSMV Beta b protein OS=Barley stripe mosaic virus OX=12327 PE=4 SV=1
2::A0A2K9YS53 **Mass:** 57104 **Score:** 41 **Matches:** 1(1) **Sequences:** 1(1)
>tr|A0A2K9YS53|A0A2K9YS53_BSMV Beta b protein OS=Barley stripe mosaic virus OX=12327 PE=4 SV=1
2::A0A2K9YS64 **Mass:** 57097 **Score:** 41 **Matches:** 1(1) **Sequences:** 1(1)
>tr|A0A2K9YS64|A0A2K9YS64_BSMV Beta b protein OS=Barley stripe mosaic virus OX=12327 PE=4 SV=1
2::Q5Q1D8 **Mass:** 57255 **Score:** 41 **Matches:** 1(1) **Sequences:** 1(1)
>tr|Q5Q1D8|Q5Q1D8_BSMV Beta-B protein OS=Barley stripe mosaic virus OX=12327 PE=2 SV=1
1::P04867 **Mass:** 58458 **Score:** 41 **Matches:** 1(1) **Sequences:** 1(1)
Movement protein TGB1 OS=Barley stripe mosaic virus OX=12327 PE=1 SV=1
2::Q65722 **Mass:** 56975 **Score:** 41 **Matches:** 1(1) **Sequences:** 1(1)
>tr|Q65722|Q65722_BSMV Beta-b protein OS=Barley stripe mosaic virus OX=12327 PE=2 SV=1
2::Q65723 **Mass:** 56931 **Score:** 41 **Matches:** 1(1) **Sequences:** 1(1)
>tr|Q65723|Q65723_BSMV Beta-b protein OS=Barley stripe mosaic virus OX=12327 PE=2 SV=1
2::Q65725 **Mass:** 57005 **Score:** 41 **Matches:** 1(1) **Sequences:** 1(1)
>tr|Q65725|Q65725_BSMV Beta-b protein OS=Barley stripe mosaic virus OX=12327 PE=2 SV=1

---

66. 2::A0A1S5NQC2 **Mass:** 186899 **Score:** 41 **Matches:** 1(1) **Sequences:** 1(1) **emPAI:** 0.02
>tr|A0A1S5NQC2|A0A1S5NQC2_9VIRU Glycoprotein (Fragment) OS=Chim orthonairovirus OX=2170062 PE=4 SV=1

Query	Observed	Mr(expt)	Mr(calc)	ppm	Miss	Score	Expect	Rank	Unique	Peptide
200	418.6901	835.3657	835.3647	1.28	0	41	0.0083	1	U	R.DWCISR.G

Proteins matching the same set of peptides:

2::A0A1V0S951 **Mass:** 82501 **Score:** 41 **Matches:** 1(1) **Sequences:** 1(1)
>tr|A0A1V0S951|A0A1V0S951_9VIRU Valyl-tRNA synthetase OS=Catovirus CTV1 OX=1977631 GN=Catovirus_1_287 PE=4 SV=1

---

67. 2::A0A023NH97 **Mass:** 88819 **Score:** 40 **Matches:** 1(1) **Sequences:** 1(1) **emPAI:** 0.05
>tr|A0A023NH97|A0A023NH97_9CAUD Putative ribonucleoside-diphosphate redutase OS=Dinoroseobacter phage DFL12phi1 OX=1477404 GN=DFL12P1_0034 PE=4 SV=1

Query	Observed	Mr(expt)	Mr(calc)	ppm	Miss	Score	Expect	Rank	Unique	Peptide
393	461.2381	920.4616	920.4603	1.39	0	40	0.047	1	U	K.WVDSSISK.T

Proteins matching the same set of peptides:

2::A0A0A7CHE9 **Mass:** 88820 **Score:** 40 **Matches:** 1(1) **Sequences:** 1(1)
>tr|A0A0A7CHE9|A0A0A7CHE9_9CAUD Putative ribonucleoside-diphosphate redutase OS=Dinoroseobacter phage vBDshPR2C OX=1498169 GN=vBDshPR2C_50 PE=4 SV=1
2::A0A2Z4QFE2 **Mass:** 88352 **Score:** 40 **Matches:** 1(1) **Sequences:** 1(1)
>tr|A0A2Z4QFE2|A0A2Z4QFE2_9CAUD Putative ribonucleoside-diphosphate reductase OS=Ruegeria phage vB_RpoP-V12 OX=2218611 GN=vBRpoPV12_50 PE=4 SV=1
2::A0A2Z4QFG0 **Mass:** 88352 **Score:** 40 **Matches:** 1(1) **Sequences:** 1(1)
>tr|A0A2Z4QFG0|A0A2Z4QFG0_9CAUD Putative ribonucleoside-diphosphate reductase OS=Ruegeria phage vB_RpoP-V21 OX=2218615 GN=vBRpoPV21_47 PE=4 SV=1
2::A0A2Z4QHC9 **Mass:** 89077 **Score:** 40 **Matches:** 1(1) **Sequences:** 1(1)
>tr|A0A2Z4QHC9|A0A2Z4QHC9_9CAUD Ribonucleotide reductase of class II OS=Ruegeria phage vB_RpoP-V13 OX=2218612 GN=vBRpoPV13_42 PE=4 SV=1
2::A0A2Z4QIG9 **Mass:** 88352 **Score:** 40 **Matches:** 1(1) **Sequences:** 1(1)
>tr|A0A2Z4QIG9|A0A2Z4QIG9_9CAUD Putative ribonucleoside-diphosphate reductase OS=Ruegeria phage vB_RpoP-V17 OX=2218614 GN=vBRpoPV17_47 PE=4 SV=1
2::A0A345AYJ0 **Mass:** 88352 **Score:** 40 **Matches:** 1(1) **Sequences:** 1(1)
>tr|A0A345AYJ0|A0A345AYJ0_9CAUD Ribonucleotide reductase of class II OS=Ruegeria phage vB_RpoP-V14 OX=2218613 GN=vBRpoPV14_52 PE=4 SV=1
2::W0NY41 **Mass:** 89169 **Score:** 40 **Matches:** 1(1) **Sequences:** 1(1)
>tr|W0NY41|W0NY41_9CAUD Ribonucleoside-diphosphate reductase OS=Sulfitobacter phage phiCB2047-B OX=754046 GN=SUFG_00031 PE=4 SV=1
2::E3PZ93 **Mass:** 88256 **Score:** 40 **Matches:** 1(1) **Sequences:** 1(1)
>tr|E3PZ93|E3PZ93_9CAUD Hypothetical host-like ribonucleoside diphosphate reductase OS=Roseovarius sp. 217 phage 1 OX=874471 PE=4 SV=1
2::C4NT50 **Mass:** 88352 **Score:** 40 **Matches:** 1(1) **Sequences:** 1(1)
>tr|C4NT50|C4NT50_9CAUD Putative ribonucleoside-diphosphate redutase OS=Silicibacter phage DSS3phi2 OX=490912 PE=4 SV=1
2::C4NTC8 **Mass:** 88160 **Score:** 40 **Matches:** 1(1) **Sequences:** 1(1)
>tr|C4NTC8|C4NTC8_9CAUD Putative ribonucleoside-diphosphate redutase OS=Sulfitobacter phage EE36phi1 OX=490913 PE=4 SV=1
2::K4Q4Y8 **Mass:** 88171 **Score:** 40 **Matches:** 1(1) **Sequences:** 1(1)
>tr|K4Q4Y8|K4Q4Y8_9CAUD Hypothetical host-like protein OS=Roseovarius Plymouth podovirus 1 OX=926474 PE=4 SV=1

Mascot: http://www.matrixscience.com/

Mascot Search Results

User	: Asia Zebrowska
Email	: asia.zebrowska87@gmail.com
Search title	: Copy of Zebrowska cal viruses sheep
MS data file	: \\212.87.29.243\Dane\USERS\Agata\tmp\00229308zebr_GP_TP84_G.mgf
Database 1	: Sprot 2020_01 (561911 sequences; 202173710 residues)
Taxonomy 1	: Viruses (16712 sequences)
Database 2	: Trembl 2019_11 (179250561 sequences; 60319985647 residues)
Taxonomy 2	: Viruses (4300387 sequences)
Timestamp	: 11 Mar 2020 at 12:24:34 GMT
Enzyme	: Trypsin
Fixed modifications	: Carbamidomethyl (C)
Variable modifications	: Oxidation (M)
Mass values	: Monoisotopic
Protein Mass	: Unrestricted
Peptide Mass Tolerance	: ± 5.92 ppm
Fragment Mass Tolerance	: ± 0.01 Da
Max Missed Cleavages	: 1
Instrument type	: HCD
Number of queries	: 5179
Protein hits	: 2::A0A1U9WQS0 >tr|A0A1U9WQS0|A0A1U9WQS0_9CAUD Glycosylase OS=Geobacillus phage TP-84 OX=1965361 PE=4 SV=1
	2::A0A076FL24 >tr|A0A076FL24|A0A076FL24_9MONO Enhanced green fluorescent protein OS=Measles morbillivirus MeV-eGFP_Edm-tag OX=1524961 GN=eGFP PE=4 SV=1
	2::A0A1U9WQR1 >tr|A0A1U9WQR1|A0A1U9WQR1_9CAUD Tail protein OS=Geobacillus phage TP-84 OX=1965361 PE=4 SV=1
	2::A0A218MLN2 >tr|A0A218MLN2|A0A218MLN2_9VIRU Putative structural protein OS=uncultured virus OX=340016 PE=4 SV=1
	2::A0A3Q8J4E7 >tr|A0A3Q8J4E7|A0A3Q8J4E7_9GAMA Glycoprotein B OS=Vombatid gammaherpesvirus 1 OX=2052651 GN=ORF8 PE=4 SV=1
	2::G8XUC3 >tr|G8XUC3|G8XUC3_9BETA Large tegument protein OS=Aotine betaherpesvirus 1 OX=50290 GN=UL48 PE=3 SV=1
	2::A0A345AYC4 >tr|A0A345AYC4|A0A345AYC4_9CAUD Uncharacterized protein OS=Synechococcus T7-like virus S-TIP37 OX=1332145 GN=STIP37_44 PE=4 SV=1
	2::C8CJF1 >tr|C8CJF1|C8CJF1_9RHAB RNA-directed RNA polymerase L OS=Drosophila obscura sigmavirus 10A OX=666962 GN=L PE=3 SV=1
	2::A0A1L2CVK2 >tr|A0A1L2CVK2|A0A1L2CVK2_9CAUD Uncharacterized protein OS=Pectobacterium phage CBB OX=1792242 GN=CBB_463 PE=4 SV=1
	2::A0A1E1GE69 >tr|A0A1E1GE69|A0A1E1GE69_9CAUD Putative primase OS=Vibrio phage CKB-S1 OX=1903259 PE=4 SV=1
	2::A0A068EKL1 >tr|A0A068EKL1|A0A068EKL1_9POXV Ankyrin repeat protein OS=Pigeonpox virus OX=10264 GN=fep_016 PE=4 SV=1
	2::A0A1V0SKV6 >tr|A0A1V0SKV6|A0A1V0SKV6_9VIRU Uncharacterized protein OS=Klosneuvirus KNV1 OX=1977640 GN=Klosneuvirus_5_30 PE=4 SV=1
	2::A0A3S9UCE2 >tr|A0A3S9UCE2|A0A3S9UCE2_9CAUD CobT-like cobalamin biosynthesis protein OS=Gordonia phage Gray OX=2499024 GN=73 PE=4 SV=1
	2::A0A1X9SH86 >tr|A0A1X9SH86|A0A1X9SH86_9CAUD HNHc domain-containing protein OS=Bradyrhizobium phage BDU-MI-1 OX=1983459 PE=4 SV=1
	2::A0A223LF67 >tr|A0A223LF67|A0A223LF67_9CAUD Tail sheath protein OS=Aeromonas phage AS-gz OX=2026082 PE=4 SV=1
	2::A0A1D8KUC6 >tr|A0A1D8KUC6|A0A1D8KUC6_9CAUD DEAD-like helicase superfamily domain containing protein OS=Synechococcus phage S-CAM7 OX=1883368 GN=S420910_056 PE=4 SV=1
	2::A0A0S1M392 >tr|A0A0S1M392|A0A0S1M392_9PICO Genome polyprotein OS=Hedgehog hepatovirus OX=1745249 PE=4 SV=1
	2::A0A2Z2CKJ5 >tr|A0A2Z2CKJ5|A0A2Z2CKJ5_9VIRU Glycoprotein OS=Pidgey virus OX=1911436 PE=4 SV=1
	2::A0A1W6JEN0 >tr|A0A1W6JEN0|A0A1W6JEN0_9BETA Uncharacterized protein OS=Human betaherpesvirus 6 OX=10368 PE=4 SV=1
	2::E5E431 >tr|E5E431|E5E431_9CAUD Uncharacterized protein OS=Acinetobacter phage Acj61 OX=760732 GN=Acj61p050 PE=4 SV=1
	2::A0A401IPN0 >tr|A0A401IPN0|A0A401IPN0_9VIRU Wsv514-like protein OS=Sesarmops intermedium nimavirus OX=2133796 PE=4 SV=1
	2::A0A1B0VV78 >tr|A0A1B0VV78|A0A1B0VV78_9CAUD RIIA protein OS=Salmonella phage vB_SnwM_CGG4-1 OX=1815631 GN=CGG41_001 PE=4 SV=1
	2::A0A0C5IA69 >tr|A0A0C5IA69|A0A0C5IA69_9VIRU Capsid protein OS=Simian torque teno virus 33 OX=1629656 PE=3 SV=1
	2::A0A1Z1DA46 >tr|A0A1Z1DA46|A0A1Z1DA46_9CAUD Uncharacterized protein OS=Bacillus phage vB_BsuM-Goe3 OX=1933063 GN=Goe3_c21400 PE=4 SV=1
	2::A0A1D8EZ70 >tr|A0A1D8EZ70|A0A1D8EZ70_9CAUD Lysin A OS=Mycobacterium phage Badger OX=1881140 GN=SEA_BADGER_8 PE=4 SV=1
	2::L0H229 >tr|L0H229|L0H229_9HIV1 Envelope glycoprotein (Fragment) OS=Human immunodeficiency virus 1 OX=11676 GN=env PE=4 SV=1
	2::A0A0A7RU81 >tr|A0A0A7RU81|A0A0A7RU81_9CAUD Tail fiber protein OS=Clostridium phage phiCT19406B OX=1567010 GN=phiCT19406B_53 PE=4 SV=1
	2::Q9J4F7 >tr|Q9J4F7|Q9J4F7_9HIV1 Envelope glycoprotein (Fragment) OS=Human immunodeficiency virus 1 OX=11676 GN=env PE=4 SV=1
	2::A0A023VZE8 >tr|A0A023VZE8|A0A023VZE8_BSMV Beta-B protein OS=Barley stripe mosaic virus OX=12327 PE=4 SV=1
	2::A0A290FZR5 >tr|A0A290FZR5|A0A290FZR5_9CAUD Uncharacterized protein OS=Caldibacillus phage CBP1 OX=2036768 PE=4 SV=1
	2::B7SQB8 >tr|B7SQB8|B7SQB8_9VIRU Nucleoprotein (Fragment) OS=Central Plata virus OX=374423 GN=N PE=2 SV=1

	Target	Decoy	False discovery rate
Peptide matches above identity threshold	63	56	88.89 %
Peptide matches above homology or identity threshold	122	98	80.33 %

Select Summary Report

Format As	Select Summary (protein hits)	Help	Help
	Significance threshold p< 0.05	Max. number of hits AUTO	Show Percolator scores ☐
	Standard scoring ○ MudPIT scoring ⦿	Ions score or expect cut-off 40	Show sub-sets 0
	Show pop-ups ○ Suppress pop-ups ⦿		Require bold red ☑
	Preferred taxonomy All entries		

Re-Search ⦿ All queries ○ Unassigned ○ Below homology threshold ○ Below identity threshold

1. 2::A0A1U9WQS0 **Mass:** 112893 **Score:** 274 **Matches:** 4(4) **Sequences:** 3(3) **emPAI:** 0.12
>tr|A0A1U9WQS0|A0A1U9WQS0_9CAUD Glycosylase OS=Geobacillus phage TP-84 OX=1965361 PE=4 SV=1

Query	Observed	Mr(expt)	Mr(calc)	ppm	Miss	Score	Expect	Rank	Unique	Peptide
436	501.3036	1000.5926	1000.5917	0.86	0	88	3.1e-07	1	U	K.ISGLAGVTGVK.G
1358	649.8342	1297.6539	1297.6554	-1.20	0	50	0.0036	1	U	R.FSVIIDGQEYK.M
2831	917.4688	1832.9230	1832.9268	-2.07	0	108	7.2e-09	1	U	R.LLDSQTAQDNFINNLK.T 2832

2. 2::A0A076FL24 **Mass:** 27039 **Score:** 271 **Matches:** 5(5) **Sequences:** 4(4) **emPAI:** 0.86
>tr|A0A076FL24|A0A076FL24_9MONO Enhanced green fluorescent protein OS=Measles morbillivirus MeV-eGFP_Edm-tag OX=1524961 GN=eGFP PE=4 SV=1

Query	Observed	Mr(expt)	Mr(calc)	ppm	Miss	Score	Expect	Rank	Unique	Peptide
1309	641.7900	1281.5655	1281.5659	-0.33	0	46	0.006	1	U	K.SAMPEGYVQER.T
3844	813.0939	2436.2600	2436.2537	2.59	0	96	4.7e-08	1	U	K.GEELFTGVVPILVELDGDVNGHK.F
4094	688.6199	2750.4507	2750.4491	0.59	1	72	1.3e-05	1	U	M.VSKGEELFTGVVPILVELDGDVNGHK.F
4213	725.3784	2897.4846	2897.4845	0.04	1	68	4.7e-05	1	U	-.MVSKGEELFTGVVPILVELDGDVNGHK.F 4212

Proteins matching the same set of peptides:
2::A0A076V611 **Mass:** 27039 **Score:** 271 **Matches:** 5(5) **Sequences:** 4(4)
>tr|A0A076V611|A0A076V611_9VIRU EGFP OS=synthetic recombinant Chikungunya virus strain LS3-GFP OX=1529938 PE=4 SV=1
2::A0A0U3A3D8 **Mass:** 27039 **Score:** 271 **Matches:** 5(5) **Sequences:** 4(4)
>tr|A0A0U3A3D8|A0A0U3A3D8_9MONO EGFP OS=Zaire ebolavirus OX=186538 PE=4 SV=1
2::A0A1V0D974 **Mass:** 27010 **Score:** 271 **Matches:** 5(5) **Sequences:** 4(4)
>tr|A0A1V0D974|A0A1V0D974_9POXV EGFP OS=Vaccinia virus OX=10245 GN=EGFP PE=4 SV=1
2::A0A348GST9 **Mass:** 27039 **Score:** 271 **Matches:** 5(5) **Sequences:** 4(4)
>tr|A0A348GST9|A0A348GST9_9MONO Enhanced green fluorescent protein OS=Measles morbillivirus OX=11234 GN=EGFP PE=4 SV=1
2::A0A3Q8WKS4 **Mass:** 27039 **Score:** 271 **Matches:** 5(5) **Sequences:** 4(4)
>tr|A0A3Q8WKS4|A0A3Q8WKS4_9VIRU Enhanced green fluorescence protein OS=Sacbrood virus OX=89463 PE=4 SV=1
2::A0A5J6XWW3 **Mass:** 27089 **Score:** 271 **Matches:** 5(5) **Sequences:** 4(4)
>tr|A0A5J6XWW3|A0A5J6XWW3_9GAMA YFP OS=Macacine gammaherpesvirus 5 OX=154334 PE=4 SV=1
2::C5MKY7 **Mass:** 27039 **Score:** 271 **Matches:** 5(5) **Sequences:** 4(4)
>tr|C5MKY7|C5MKY7_HCMV Enhanced green fluorescent protein OS=Human cytomegalovirus OX=10359 GN=egfp PE=4 SV=1

3. 2::A0A1U9WQR1 **Mass:** 99414 **Score:** 92 **Matches:** 2(2) **Sequences:** 2(2) **emPAI:** 0.09
>tr|A0A1U9WQR1|A0A1U9WQR1_9CAUD Tail protein OS=Geobacillus phage TP-84 OX=1965361 PE=4 SV=1

Query	Observed	Mr(expt)	Mr(calc)	ppm	Miss	Score	Expect	Rank	Unique	Peptide
780	561.2956	1120.5767	1120.5764	0.28	0	60	0.00028	1	U	K.VINYEQVEK.T
1626	690.3416	1378.6687	1378.6688	-0.13	1	61	0.00029	1	U	K.TSVDGTINSSDKR.Y

4. 2::A0A218MLN2 **Mass:** 43019 **Score:** 79 **Matches:** 3(3) **Sequences:** 2(2) **emPAI:** 0.22
>tr|A0A218MLN2|A0A218MLN2_9VIRU Putative structural protein OS=uncultured virus OX=340016 PE=4 SV=1

Query	Observed	Mr(expt)	Mr(calc)	ppm	Miss	Score	Expect	Rank	Unique	Peptide
1	351.7046	701.3946	701.3959	-1.91	0	58	0.0007	1	U	K.VEEVVK.E 2
103	415.7519	829.4892	829.4909	-2.06	1	45	0.015	1	U	K.KVEEVVK.E

5. 2::A0A3Q8J4E7 **Mass:** 91595 **Score:** 70 **Matches:** 2(2) **Sequences:** 1(1) **emPAI:** 0.05
>tr|A0A3Q8J4E7|A0A3Q8J4E7_9GAMA Glycoprotein B OS=Vombatid gammaherpesvirus 1 OX=2052651 GN=ORF8 PE=4 SV=1

Query	Observed	Mr(expt)	Mr(calc)	ppm	Miss	Score	Expect	Rank	Unique	Peptide
8	358.7180	715.4215	715.4228	-1.82	0	59	0.0016	1	U	K.TVIIDR.T 7

Proteins matching the same set of peptides:
2::V5K6X0 **Mass:** 65777 **Score:** 70 **Matches:** 2(2) **Sequences:** 1(1)
>tr|V5K6X0|V5K6X0_9MONO Transmembrane protein OS=Miniopterus schreibersii paramyxovirus OX=1387879 GN=TM PE=4 SV=1

6. 2::G8XUC3 **Mass:** 242144 **Score:** 63 **Matches:** 4(4) **Sequences:** 1(1) **emPAI:** 0.02
>tr|G8XUC3|G8XUC3_9BETA Large tegument protein OS=Aotine betaherpesvirus 1 OX=50290 GN=UL48 PE=3 SV=1

Query	Observed	Mr(expt)	Mr(calc)	ppm	Miss	Score	Expect	Rank	Unique	Peptide
666	544.7993	1087.5840	1087.5873	-3.02	1	49	0.015	1	U	K.VEAVSKAAADK.N 668 669 670

7. 2::A0A345AYC4 **Mass:** 6048 **Score:** 61 **Matches:** 1(1) **Sequences:** 1(1) **emPAI:** 0.94
>tr|A0A345AYC4|A0A345AYC4_9CAUD Uncharacterized protein OS=Synechococcus T7-like virus S-TIP37 OX=1332145 GN=STIP37_44 PE=4 SV=1

Query	Observed	Mr(expt)	Mr(calc)	ppm	Miss	Score	Expect	Rank	Unique	Peptide
212	438.2533	874.4920	874.4946	-2.95	0	61	0.001	1	U	R.MIVDLLR.A

8. 2::C8CJF1 **Mass:** 247398 **Score:** 60 **Matches:** 2(2) **Sequences:** 1(1) **emPAI:** 0.02
>tr|C8CJF1|C8CJF1_9RHAB RNA-directed RNA polymerase L OS=Drosophila obscura sigmavirus 10A OX=666962 GN=L PE=3 SV=1

Query	Observed	Mr(expt)	Mr(calc)	ppm	Miss	Score	Expect	Rank	Unique	Peptide
41	381.2192	760.4238	760.4232	0.80	0	46	0.017	1	U	K.QPTLFR.T 40

9. 2::A0A1L2CVK2 **Mass:** 19336 **Score:** 58 **Matches:** 2(2) **Sequences:** 1(1) **emPAI:** 0.24
>tr|A0A1L2CVK2|A0A1L2CVK2_9CAUD Uncharacterized protein OS=Pectobacterium phage CBB OX=1792242 GN=CBB_463 PE=4 SV=1

Query	Observed	Mr(expt)	Mr(calc)	ppm	Miss	Score	Expect	Rank	Unique	Peptide
19	365.2344	728.4543	728.4545	-0.21	0	51	0.0067	1	U	R.TLQVLR.M 18

Proteins matching the same set of peptides:
2::K4F7S2 **Mass:** 19272 **Score:** 58 **Matches:** 2(2) **Sequences:** 1(1)
>tr|K4F7S2|K4F7S2_9CAUD Uncharacterized protein OS=Cronobacter phage vB_CsaM_GAP32 OX=1141136 GN=GAP32_442 PE=4 SV=1

10. 2::A0A1E1GE69 **Mass:** 88638 **Score:** 56 **Matches:** 2(2) **Sequences:** 1(1) **emPAI:** 0.05
>tr|A0A1E1GE69|A0A1E1GE69_9CAUD Putative primase OS=Vibrio phage CKB-S1 OX=1903259 PE=4 SV=1

Query	Observed	Mr(expt)	Mr(calc)	ppm	Miss	Score	Expect	Rank	Unique	Peptide
399	494.2764	986.5383	986.5397	-1.42	0	49	0.0053	1	U	R.AAVSAGVGDLK.G 398

11. 2::A0A068EKL1 **Mass:** 46526 **Score:** 54 **Matches:** 1(1) **Sequences:** 1(1) **emPAI:** 0.09
>tr|A0A068EKL1|A0A068EKL1_9POXV Ankyrin repeat protein OS=Pigeonpox virus OX=10264 GN=fep_016 PE=4 SV=1

Query	Observed	Mr(expt)	Mr(calc)	ppm	Miss	Score	Expect	Rank	Unique	Peptide
32	376.7123	751.4100	751.4116	-2.15	0	54	0.0012	1	U	K.LSYEIK.Y

Proteins matching the same set of peptides:
2::A0A068EL77 **Mass:** 46093 **Score:** 54 **Matches:** 1(1) **Sequences:** 1(1)
>tr|A0A068EL77|A0A068EL77_9POXV Ankyrin repeat protein OS=Penguinpox virus OX=648998 GN=pepv_016 PE=4 SV=1
2::A0A1X9Q118 **Mass:** 56950 **Score:** 54 **Matches:** 1(1) **Sequences:** 1(1)
>tr|A0A1X9Q118|A0A1X9Q118_9VIRU Putative VP4 OS=Human gut microviridae SH-CHD8 OX=1986033 PE=4 SV=1

12. 2::A0A1V0SKV6 **Mass:** 61089 **Score:** 54 **Matches:** 1(1) **Sequences:** 1(1) **emPAI:** 0.07
>tr|A0A1V0SKV6|A0A1V0SKV6_9VIRU Uncharacterized protein OS=Klosneuvirus KNV1 OX=1977640 GN=Klosneuvirus_5_30 PE=4 SV=1

Query	Observed	Mr(expt)	Mr(calc)	ppm	Miss	Score	Expect	Rank	Unique	Peptide
9	360.7178	719.4211	719.4218	-0.89	0	54	0.00078	1	U	R.IPYSLK.Y

Proteins matching the same set of peptides:
2::A0A385I731 **Mass:** 203583 **Score:** 54 **Matches:** 1(1) **Sequences:** 1(1)
>tr|A0A385I731|A0A385I731_9RETR Glycoprotein OS=Saesbyeol virus OX=2320522 PE=4 SV=1
2::A0A5J6VJE5 **Mass:** 45156 **Score:** 54 **Matches:** 1(1) **Sequences:** 1(1)
>tr|A0A5J6VJE5|A0A5J6VJE5_9VIRU Elongation factor Tu GTP binding domain protein OS=Megaviridae environmental sample OX=1737588 PE=4 SV=1

13. 2::A0A3S9UCE2 **Mass:** 66154 **Score:** 52 **Matches:** 2(2) **Sequences:** 1(1) **emPAI:** 0.07
>tr|A0A3S9UCE2|A0A3S9UCE2_9CAUD CobT-like cobalamin biosynthesis protein OS=Gordonia phage Gray OX=2499024 GN=73 PE=4 SV=1

Query	Observed	Mr(expt)	Mr(calc)	ppm	Miss	Score	Expect	Rank	Unique	Peptide
78	404.2022	806.3898	806.3923	-3.10	0	43	0.0076	1	U	K.LAADFDR.V 79

14. 2::A0A1X9SH86 **Mass:** 24714 **Score:** 52 **Matches:** 2(2) **Sequences:** 1(1) **emPAI:** 0.18
>tr|A0A1X9SH86|A0A1X9SH86_9CAUD HNHc domain-containing protein OS=Bradyrhizobium phage BDU-MI-1 OX=1983459 PE=4 SV=1

Query	Observed	Mr(expt)	Mr(calc)	ppm	Miss	Score	Expect	Rank	Unique	Peptide
46	387.2287	772.4429	772.4443	-1.86	0	48	0.013	1	U	R.ASVILDR.G 45

Proteins matching the same set of peptides:
2::B7SYF4 **Mass:** 24200 **Score:** 52 **Matches:** 2(2) **Sequences:** 1(1)
>tr|B7SYF4|B7SYF4_9CAUD Gp24 OS=Stenotrophomonas phage S1 OX=573591 PE=4 SV=1

15. 2::A0A223LF67 **Mass:** 71904 **Score:** 50 **Matches:** 1(1) **Sequences:** 1(1) **emPAI:** 0.06
>tr|A0A223LF67|A0A223LF67_9CAUD Tail sheath protein OS=Aeromonas phage AS-gz OX=2026082 PE=4 SV=1

Query	Observed	Mr(expt)	Mr(calc)	ppm	Miss	Score	Expect	Rank	Unique	Peptide
138	423.2411	844.4677	844.4654	2.71	1	50	0.0057	1	U	K.VDADGKIK.A

Proteins matching the same set of peptides:
2::A0A2S1PEG1 **Mass:** 71953 **Score:** 50 **Matches:** 1(1) **Sequences:** 1(1)
>tr|A0A2S1PEG1|A0A2S1PEG1_9CAUD Tail sheath protein OS=Aeromonas phage 50AhydR13PP OX=2163978 PE=4 SV=1
2::A0A2S1PG39 **Mass:** 71904 **Score:** 50 **Matches:** 1(1) **Sequences:** 1(1)
>tr|A0A2S1PG39|A0A2S1PG39_9CAUD Tail sheath protein OS=Aeromonas phage 60AhydR15PP OX=2163979 PE=4 SV=1
2::E1A1G3 **Mass:** 28558 **Score:** 50 **Matches:** 1(1) **Sequences:** 1(1)
>tr|E1A1G3|E1A1G3_9CAUD Tail sheath monomer-like protein OS=Aeromonas phage phiAS4 OX=879628 GN=phiAS4_ORF0115 PE=4 SV=1
2::I6ZI77 **Mass:** 71966 **Score:** 50 **Matches:** 1(1) **Sequences:** 1(1)
>tr|I6ZI77|I6ZI77_9CAUD Tail sheath protein OS=Aeromonas phage Aes012 OX=1198014 GN=Aes012_153 PE=4 SV=1
2::J7HXR1 **Mass:** 71881 **Score:** 50 **Matches:** 1(1) **Sequences:** 1(1)
>tr|J7HXR1|J7HXR1_9CAUD Tail sheath monomer OS=Stenotrophomonas phage IME13 OX=1211280 PE=4 SV=1
2::J7KLQ6 **Mass:** 71908 **Score:** 50 **Matches:** 1(1) **Sequences:** 1(1)
>tr|J7KLQ6|J7KLQ6_9CAUD Tail sheath protein OS=Aeromonas phage Aes508 OX=1198013 GN=Aes508_142 PE=4 SV=1
2::Q19CM1 **Mass:** 71950 **Score:** 50 **Matches:** 1(1) **Sequences:** 1(1)
>tr|Q19CM1|Q19CM1_9CAUD Gp18 tail sheath monomer OS=Aeromonas virus 25 OX=233894 GN=18 PE=4 SV=1

16. 2::A0A1D8KUC6 **Mass:** 53669 **Score:** 50 **Matches:** 1(1) **Sequences:** 1(1) **emPAI:** 0.08
>tr|A0A1D8KUC6|A0A1D8KUC6_9CAUD DEAD-like helicase superfamily domain containing protein OS=Synechococcus phage S-CAM7 OX=1883368 GN=S420910_056 PE=4 SV=1

Query	Observed	Mr(expt)	Mr(calc)	ppm	Miss	Score	Expect	Rank	Unique	Peptide
31	375.1842	748.3538	748.3538	0.05	0	50	0.0039	1	U	K.TINDMR.T

17. 2::A0A0S1M392 **Mass:** 255098 **Score:** 49 **Matches:** 2(2) **Sequences:** 1(1) **emPAI:** 0.02
>tr|A0A0S1M392|A0A0S1M392_9PICO Genome polyprotein OS=Hedgehog hepatovirus OX=1745249 PE=4 SV=1

Query	Observed	Mr(expt)	Mr(calc)	ppm	Miss	Score	Expect	Rank	Unique	Peptide
4	352.6813	703.3480	703.3500	-2.83	0	46	0.024	1	U	K.SLNENK.I 3

Proteins matching the same set of peptides:
2::A0A0S1M3C0 **Mass:** 254956 **Score:** 49 **Matches:** 2(2) **Sequences:** 1(1)
>tr|A0A0S1M3C0|A0A0S1M3C0_9PICO Genome polyprotein OS=Hedgehog hepatovirus OX=1745249 PE=4 SV=1
2::A0A0S1M3C5 **Mass:** 255134 **Score:** 49 **Matches:** 2(2) **Sequences:** 1(1)
>tr|A0A0S1M3C5|A0A0S1M3C5_9PICO Genome polyprotein OS=Hedgehog hepatovirus Igel8Erieur2014 OX=1987144 PE=4 SV=1
2::A0A4V1F5G8 **Mass:** 8213 **Score:** 49 **Matches:** 2(2) **Sequences:** 1(1)
>tr|A0A4V1F5G8|A0A4V1F5G8_9VIRU Uncharacterized protein OS=Blackfly microvirus SF02 OX=2576452 PE=4 SV=1

18. 2::A0A2Z2CKJ5 **Mass:** 134247 **Score:** 48 **Matches:** 1(1) **Sequences:** 1(1) **emPAI:** 0.03
>tr|A0A2Z2CKJ5|A0A2Z2CKJ5_9VIRU Glycoprotein OS=Pidgey virus OX=1911436 PE=4 SV=1

Query	Observed	Mr(expt)	Mr(calc)	ppm	Miss	Score	Expect	Rank	Unique	Peptide
28	373.2129	744.4112	744.4130	-2.34	0	48	0.024	1	U	R.EVTQLR.A

Proteins matching the same set of peptides:
2::Q90QL4 **Mass:** 12443 **Score:** 48 **Matches:** 1(1) **Sequences:** 1(1)
>tr|Q90QL4|Q90QL4_9HIV1 Envelope glycoprotein (Fragment) OS=Human immunodeficiency virus 1 OX=11676 GN=env PE=4 SV=1

19. 2::A0A1W6JEN0 **Mass:** 48576 **Score:** 48 **Matches:** 1(1) **Sequences:** 1(1) **emPAI:** 0.09
>tr|A0A1W6JEN0|A0A1W6JEN0_9BETA Uncharacterized protein OS=Human betaherpesvirus 6 OX=10368 PE=4 SV=1

Query	Observed	Mr(expt)	Mr(calc)	ppm	Miss	Score	Expect	Rank	Unique	Peptide
373	487.2687	972.5228	972.5240	-1.22	0	48	0.0087	1	U	R.IESLENLR.N

20. 2::E5E431 **Mass:** 16863 **Score:** 48 **Matches:** 2(2) **Sequences:** 1(1) **emPAI:** 0.28
>tr|E5E431|E5E431_9CAUD Uncharacterized protein OS=Acinetobacter phage Acj61 OX=760732 GN=Acj61p050 PE=4 SV=1

Query	Observed	Mr(expt)	Mr(calc)	ppm	Miss	Score	Expect	Rank	Unique	Peptide
93	408.7126	815.4106	815.4137	-3.75	0	42	0.017	1	U	R.AEAQLER.A 94

21. 2::A0A401IPN0 **Mass:** 255005 **Score:** 47 **Matches:** 1(1) **Sequences:** 1(1) **emPAI:** 0.02
>tr|A0A401IPN0|A0A401IPN0_9VIRU Wsv514-like protein OS=Sesarmops intermedium nimavirus OX=2133796 PE=4 SV=1

Query	Observed	Mr(expt)	Mr(calc)	ppm	Miss	Score	Expect	Rank	Unique	Peptide
90	406.2183	810.4220	810.4236	-1.96	0	47	0.0029	1	U	K.IGEHIDK.F

22. 2::A0A1B0VV78 **Mass:** 84210 **Score:** 46 **Matches:** 2(1) **Sequences:** 1(1) **emPAI:** 0.05
>tr|A0A1B0VV78|A0A1B0VV78_9CAUD RIIA protein OS=Salmonella phage vB_SnwM_CGG4-1 OX=1815631 GN=CGG41_001 PE=4 SV=1

Query	Observed	Mr(expt)	Mr(calc)	ppm	Miss	Score	Expect	Rank	Unique	Peptide
21	366.6972	731.3799	731.3813	-1.97	0	46	0.041	1	U	K.LDSELR.W 22

Proteins matching the same set of peptides:
2::A0A1B1MQW2 **Mass:** 93388 **Score:** 46 **Matches:** 2(1) **Sequences:** 1(1)
>tr|A0A1B1MQW2|A0A1B1MQW2_NPVLD Desmoplakin OS=Lymantria dispar multicapsid nuclear polyhedrosis virus OX=10449 PE=4 SV=1
2::A0A2D1GLZ8 **Mass:** 84449 **Score:** 46 **Matches:** 2(1) **Sequences:** 1(1)
>tr|A0A2D1GLZ8|A0A2D1GLZ8_9CAUD RIIa-like protector from prophage-induced early lysis OS=Salmonella phage Melville OX=2041413 GN=CPT_Melville_001 PE=4 SV=1
2::A0A401IPB9 **Mass:** 95369 **Score:** 46 **Matches:** 2(1) **Sequences:** 1(1)
>tr|A0A401IPB9|A0A401IPB9_9VIRU Wsv327-like protein OS=Metapenaeus ensis nimavirus OX=2133794 PE=4 SV=1
2::K4I2L6 **Mass:** 84514 **Score:** 46 **Matches:** 2(1) **Sequences:** 1(1)
>tr|K4I2L6|K4I2L6_9CAUD Uncharacterized protein OS=Salmonella phage STML-198 OX=1204531 PE=4 SV=1

23. 2::A0A0C5IA69 **Mass:** 92945 **Score:** 46 **Matches:** 1(1) **Sequences:** 1(1) **emPAI:** 0.05
>tr|A0A0C5IA69|A0A0C5IA69_9VIRU Capsid protein OS=Simian torque teno virus 33 OX=1629656 PE=3 SV=1

Query	Observed	Mr(expt)	Mr(calc)	ppm	Miss	Score	Expect	Rank	Unique	Peptide
5	352.6949	703.3752	703.3752	-0.05	0	46	0.011	1	U	R.LDSEIK.G

Proteins matching the same set of peptides:
2::A0A0C5IBK0 **Mass:** 54261 **Score:** 46 **Matches:** 1(1) **Sequences:** 1(1)
>tr|A0A0C5IBK0|A0A0C5IBK0_9VIRU Capsid protein (Fragment) OS=Simian torque teno virus OX=1619217 PE=3 SV=1
2::A0A0C5IC23 **Mass:** 93443 **Score:** 46 **Matches:** 1(1) **Sequences:** 1(1)
>tr|A0A0C5IC23|A0A0C5IC23_9VIRU Capsid protein (Fragment) OS=Simian torque teno virus OX=1619217 PE=3 SV=1
2::A0A0C5IC38 **Mass:** 84969 **Score:** 46 **Matches:** 1(1) **Sequences:** 1(1)
>tr|A0A0C5IC38|A0A0C5IC38_9VIRU Capsid protein (Fragment) OS=Simian torque teno virus OX=1619217 PE=3 SV=1
2::A0A0C5IMU2 **Mass:** 92923 **Score:** 46 **Matches:** 1(1) **Sequences:** 1(1)
>tr|A0A0C5IMU2|A0A0C5IMU2_9VIRU Capsid protein (Fragment) OS=Simian torque teno virus OX=1619217 PE=3 SV=1
2::A0A3G5A3D2 **Mass:** 26663 **Score:** 46 **Matches:** 1(1) **Sequences:** 1(1)
>tr|A0A3G5A3D2|A0A3G5A3D2_9VIRU Uncharacterized protein OS=Harvfovirus sp. OX=2487768 GN=Harvfovirus57_7 PE=4 SV=1
2::A0A3G8GB07 **Mass:** 6970 **Score:** 46 **Matches:** 1(1) **Sequences:** 1(1)
>tr|A0A3G8GB07|A0A3G8GB07_9VIRU Uncharacterized protein OS=Sulfolobus spindle-shaped virus OX=2491899 PE=4 SV=1

24. 2::A0A1Z1DA46 **Mass:** 9887 **Score:** 45 **Matches:** 1(1) **Sequences:** 1(1) **emPAI:** 0.51
>tr|A0A1Z1DA46|A0A1Z1DA46_9CAUD Uncharacterized protein OS=Bacillus phage vB_BsuM-Goe3 OX=1933063 GN=Goe3_c21400 PE=4 SV=1

Query	Observed	Mr(expt)	Mr(calc)	ppm	Miss	Score	Expect	Rank	Unique	Peptide
273	458.2601	914.5057	914.5073	-1.75	0	45	0.047	1	U	R.NIAEDIIK.A

Proteins matching the same set of peptides:
2::A0A516KNT8 **Mass:** 9887 **Score:** 45 **Matches:** 1(1) **Sequences:** 1(1)
>tr|A0A516KNT8|A0A516KNT8_9CAUD Uncharacterized protein OS=Bacillus phage vB_BveM-Goe7 OX=2593639 GN=Goe7_c02160 PE=4 SV=1

25. 2::A0A1D8EZ70 **Mass:** 56951 **Score:** 45 **Matches:** 2(2) **Sequences:** 1(1) **emPAI:** 0.08
>tr|A0A1D8EZ70|A0A1D8EZ70_9CAUD Lysin A OS=Mycobacterium phage Badger OX=1881140 GN=SEA_BADGER_8 PE=4 SV=1

Query	Observed	Mr(expt)	Mr(calc)	ppm	Miss	Score	Expect	Rank	Unique	Peptide
62	393.7445	785.4745	785.4759	-1.89	0	44	0.031	1	U	R.GTLAGVIR.A 63

Proteins matching the same set of peptides:
2::A0A1J0MAF1 **Mass:** 56951 **Score:** 45 **Matches:** 2(2) **Sequences:** 1(1)
>tr|A0A1J0MAF1|A0A1J0MAF1_9CAUD Lysin A OS=Mycobacterium phage Achebe OX=1916113 GN=8 PE=4 SV=1
2::A0A3S9UMP6 **Mass:** 56951 **Score:** 45 **Matches:** 2(2) **Sequences:** 1(1)
>tr|A0A3S9UMP6|A0A3S9UMP6_9CAUD Lysin A OS=Mycobacterium phage Cici OX=2499034 GN=8 PE=4 SV=1
2::A0A411BUR2 **Mass:** 56951 **Score:** 45 **Matches:** 2(2) **Sequences:** 1(1)
>tr|A0A411BUR2|A0A411BUR2_9CAUD Lysin A OS=Mycobacterium phage Katalie136 OX=2502428 GN=8 PE=4 SV=1
2::A0A411BZ85 **Mass:** 56951 **Score:** 45 **Matches:** 2(2) **Sequences:** 1(1)
>tr|A0A411BZ85|A0A411BZ85_9CAUD Lysin A OS=Mycobacterium phage Datway OX=2502440 GN=8 PE=4 SV=1
2::A0A4Y5P096 **Mass:** 56951 **Score:** 45 **Matches:** 2(2) **Sequences:** 1(1)
>tr|A0A4Y5P096|A0A4Y5P096_9CAUD Lysin A OS=Mycobacterium phage Xena OX=2584499 GN=8 PE=4 SV=1
2::G1BKY1 **Mass:** 56951 **Score:** 45 **Matches:** 2(2) **Sequences:** 1(1)
>tr|G1BKY1|G1BKY1_9CAUD Lysin A OS=Mycobacterium virus Backyardigan OX=1034129 GN=8 PE=4 SV=1
2::G1FN95 **Mass:** 56951 **Score:** 45 **Matches:** 2(2) **Sequences:** 1(1)
>tr|G1FN95|G1FN95_9CAUD Lysin A OS=Mycobacterium phage Wile OX=1056833 GN=8 PE=4 SV=1

26. 2::L0H229 **Mass:** 38097 **Score:** 44 **Matches:** 1(1) **Sequences:** 1(1) **emPAI:** 0.12
>tr|L0H229|L0H229_9HIV1 Envelope glycoprotein (Fragment) OS=Human immunodeficiency virus 1 OX=11676 GN=env PE=4 SV=1

Query	Observed	Mr(expt)	Mr(calc)	ppm	Miss	Score	Expect	Rank	Unique	Peptide
296	465.2648	928.5151	928.5130	2.19	0	44	0.0083	1	U	R.WLEVVQR.T

Proteins matching the same set of peptides:
2::L0H3M9 **Mass:** 37274 **Score:** 44 **Matches:** 1(1) **Sequences:** 1(1)
>tr|L0H3M9|L0H3M9_9HIV1 Envelope glycoprotein (Fragment) OS=Human immunodeficiency virus 1 OX=11676 GN=env PE=4 SV=1

27. 2::A0A0A7RU81 **Mass:** 47134 **Score:** 42 **Matches:** 2(2) **Sequences:** 1(1) **emPAI:** 0.09
>tr|A0A0A7RU81|A0A0A7RU81_9CAUD Tail fiber protein OS=Clostridium phage phiCT19406B OX=1567010 GN=phiCT19406B_53 PE=4 SV=1

Query	Observed	Mr(expt)	Mr(calc)	ppm	Miss	Score	Expect	Rank	Unique	Peptide
204	437.2409	872.4673	872.4715	-4.82	1	41	0.043	1	U	R.AGNLENKK.S 205

Proteins matching the same set of peptides:
2::A0A0A7S1G0 **Mass:** 47134 **Score:** 42 **Matches:** 2(2) **Sequences:** 1(1)
>tr|A0A0A7S1G0|A0A0A7S1G0_9CAUD Tail fiber protein OS=Clostridium phage phiCTC2B OX=1567016 GN=phiCTC2B_53 PE=4 SV=1

28. 2::Q9J4F7 **Mass:** 13468 **Score:** 42 **Matches:** 1(0) **Sequences:** 1(0)
>tr|Q9J4F7|Q9J4F7_9HIV1 Envelope glycoprotein (Fragment) OS=Human immunodeficiency virus 1 OX=11676 GN=env PE=4 SV=1

Query	Observed	Mr(expt)	Mr(calc)	ppm	Miss	Score	Expect	Rank	Unique	Peptide
278	458.7476	915.4807	915.4814	-0.79	0	42	0.061	1	U	K.LGELWGNK.T

Proteins matching the same set of peptides:
2::Q9J4F8 **Mass:** 13480 **Score:** 42 **Matches:** 1(0) **Sequences:** 1(0)
>tr|Q9J4F8|Q9J4F8_9HIV1 Envelope glycoprotein (Fragment) OS=Human immunodeficiency virus 1 OX=11676 GN=env PE=4 SV=1
2::Q9J4F9 **Mass:** 13481 **Score:** 42 **Matches:** 1(0) **Sequences:** 1(0)
>tr|Q9J4F9|Q9J4F9_9HIV1 Envelope glycoprotein (Fragment) OS=Human immunodeficiency virus 1 OX=11676 GN=env PE=4 SV=1
2::Q9J4G0 **Mass:** 13483 **Score:** 42 **Matches:** 1(0) **Sequences:** 1(0)
>tr|Q9J4G0|Q9J4G0_9HIV1 Envelope glycoprotein (Fragment) OS=Human immunodeficiency virus 1 OX=11676 GN=env PE=4 SV=1
2::Q9J4G1 **Mass:** 13441 **Score:** 42 **Matches:** 1(0) **Sequences:** 1(0)
>tr|Q9J4G1|Q9J4G1_9HIV1 Envelope glycoprotein (Fragment) OS=Human immunodeficiency virus 1 OX=11676 GN=env PE=4 SV=1
2::Q9J4G2 **Mass:** 13485 **Score:** 42 **Matches:** 1(0) **Sequences:** 1(0)
>tr|Q9J4G2|Q9J4G2_9HIV1 Envelope glycoprotein (Fragment) OS=Human immunodeficiency virus 1 OX=11676 GN=env PE=4 SV=1
2::Q9J4G3 **Mass:** 13485 **Score:** 42 **Matches:** 1(0) **Sequences:** 1(0)
>tr|Q9J4G3|Q9J4G3_9HIV1 Envelope glycoprotein (Fragment) OS=Human immunodeficiency virus 1 OX=11676 GN=env PE=4 SV=1
2::Q9J4G4 **Mass:** 13497 **Score:** 42 **Matches:** 1(0) **Sequences:** 1(0)
>tr|Q9J4G4|Q9J4G4_9HIV1 Envelope glycoprotein (Fragment) OS=Human immunodeficiency virus 1 OX=11676 GN=env PE=4 SV=1
2::Q9J4G5 **Mass:** 13513 **Score:** 42 **Matches:** 1(0) **Sequences:** 1(0)
>tr|Q9J4G5|Q9J4G5_9HIV1 Envelope glycoprotein (Fragment) OS=Human immunodeficiency virus 1 OX=11676 GN=env PE=4 SV=1

29. 2::A0A023VZE8 **Mass:** 58864 **Score:** 42 **Matches:** 1(1) **Sequences:** 1(1) **emPAI:** 0.07
>tr|A0A023VZE8|A0A023VZE8_BSMV Beta-B protein OS=Barley stripe mosaic virus OX=12327 PE=4 SV=1

Query	Observed	Mr(expt)	Mr(calc)	ppm	Miss	Score	Expect	Rank	Unique	Peptide
85	405.2241	808.4337	808.4330	0.75	0	42	0.022	1	U	R.LASLYDK.H

Proteins matching the same set of peptides:
2::A0A0G2RAA1 **Mass:** 56933 **Score:** 42 **Matches:** 1(1) **Sequences:** 1(1)
>tr|A0A0G2RAA1|A0A0G2RAA1_BSMV Beta b protein OS=Barley stripe mosaic virus OX=12327 PE=4 SV=1
2::A0A2K9YRQ0 **Mass:** 57069 **Score:** 42 **Matches:** 1(1) **Sequences:** 1(1)
>tr|A0A2K9YRQ0|A0A2K9YRQ0_BSMV Beta b protein OS=Barley stripe mosaic virus OX=12327 PE=4 SV=1
2::A0A2K9YS21 **Mass:** 56872 **Score:** 42 **Matches:** 1(1) **Sequences:** 1(1)
>tr|A0A2K9YS21|A0A2K9YS21_BSMV Beta b protein OS=Barley stripe mosaic virus OX=12327 PE=4 SV=1
2::A0A2K9YS53 **Mass:** 57104 **Score:** 42 **Matches:** 1(1) **Sequences:** 1(1)
>tr|A0A2K9YS53|A0A2K9YS53_BSMV Beta b protein OS=Barley stripe mosaic virus OX=12327 PE=4 SV=1
2::A0A2K9YS64 **Mass:** 57097 **Score:** 42 **Matches:** 1(1) **Sequences:** 1(1)
>tr|A0A2K9YS64|A0A2K9YS64_BSMV Beta b protein OS=Barley stripe mosaic virus OX=12327 PE=4 SV=1
2::Q5Q1D8 **Mass:** 57255 **Score:** 42 **Matches:** 1(1) **Sequences:** 1(1)
>tr|Q5Q1D8|Q5Q1D8_BSMV Beta-B protein OS=Barley stripe mosaic virus OX=12327 PE=2 SV=1
1::P04867 **Mass:** 58458 **Score:** 42 **Matches:** 1(1) **Sequences:** 1(1)
Movement protein TGB1 OS=Barley stripe mosaic virus OX=12327 PE=1 SV=1
2::Q65722 **Mass:** 56975 **Score:** 42 **Matches:** 1(1) **Sequences:** 1(1)
>tr|Q65722|Q65722_BSMV Beta-b protein OS=Barley stripe mosaic virus OX=12327 PE=2 SV=1
2::Q65723 **Mass:** 56931 **Score:** 42 **Matches:** 1(1) **Sequences:** 1(1)
>tr|Q65723|Q65723_BSMV Beta-b protein OS=Barley stripe mosaic virus OX=12327 PE=2 SV=1
2::Q65725 **Mass:** 57005 **Score:** 42 **Matches:** 1(1) **Sequences:** 1(1)
>tr|Q65725|Q65725_BSMV Beta-b protein OS=Barley stripe mosaic virus OX=12327 PE=2 SV=1

30. 2::A0A290FZR5 **Mass:** 18830 **Score:** 41 **Matches:** 1(1) **Sequences:** 1(1) **emPAI:** 0.25
>tr|A0A290FZR5|A0A290FZR5_9CAUD Uncharacterized protein OS=Caldibacillus phage CBP1 OX=2036768 PE=4 SV=1

Query	Observed	Mr(expt)	Mr(calc)	ppm	Miss	Score	Expect	Rank	Unique	Peptide
418	498.2633	994.5121	994.5123	-0.25	1	41	0.034	1	U	K.KIEWYEK.A

31. 2::B7SQB8 **Mass:** 13678 **Score:** 40 **Matches:** 1(1) **Sequences:** 1(1) **emPAI:** 0.35
>tr|B7SQB8|B7SQB8_9VIRU Nucleoprotein (Fragment) OS=Central Plata virus OX=374423 GN=N PE=2 SV=1

Query	Observed	Mr(expt)	Mr(calc)	ppm	Miss	Score	Expect	Rank	Unique	Peptide
339	473.7342	945.4538	945.4589	-5.44	0	40	0.03	1	U	K.AEEITPMR.F
